# Regulation of Alternative Splicing of Lipid Metabolism Genes in Sepsis-Induced Liver Damage by RNA-Binding Proteins

**DOI:** 10.1007/s10753-024-02017-2

**Published:** 2024-05-09

**Authors:** Buzukela Abuduaini, Zhang Jiyuan, Aliya Rehati, Zhao Liang, Song Yunlin

**Affiliations:** 1 Department of Intensive Care Unit, The First Affiliated Hospital of Xinjiang Medical University, 393 South Li Yu Shan Road, Ürümqi, 830054, Xinjiang China; 2 First Clinical institute of Xinjiang Medical University, Ürümqi, 830054 Xinjiang China; 3 Department of Gastroenternology, The First Affiliated Hospital of Xinjiang Medical University, 393 South Li Yu Shan Road, Ürümqi, 830054 Xinjiang China; 4 Department of General Surgery, The First Affiliated Hospital of Xinjiang Medical University, Ürümqi, 830054 Xinjiang, China; 5 Department of Intensive Care Unit, The First Affiliated Hospital of Xinjiang Medical University,State Key Laboratory of Pathogenesis, Prevention and Treatment of high Incidence Diseases in Central Asia, 393 South Li Yu Shan Road, Ürümqi, 830054 Xinjiang China

**Keywords:** dichloroacetate, sepsis-induced liver injury, RNA binding proteins, lipid metabolism.

## Abstract

RNA binding proteins (RBPs) have the potential for transcriptional regulation in sepsis-induced liver injury, but precise functions remain unclear. Our aim is to conduct a genome-wide expression analysis of RBPs and illuminate changes in the regulation of alternative splicing in sepsis-induced liver injury. RNA-seq data on “sepsis and liver” from the publicly available NCBI data set was analyzed, and differentially expressed RBPs and alternative splicing events (ASEs) in the healthy and septic liver were identified. Co-expression analyses of sepsis-regulated RBPs and ASEs were performed. Models of sepsis were established to validate hepatic RBP gene expression patterns with different treatments. Pairwise analysis of gene expression profiles of sham, cecum ligation puncture (CLP), and CLP with dichloroacetate (CLPDCA) mice allowed 1208 differentially expressed genes (DEGs), of which 800 were up-regulated and 408 down-regulated, to be identified. DEGs were similar in both Sham and CLPDCA mice. The KEGG analysis showed that up-regulated genes as being involved in cytokine-cytokine receptor interaction and IL-17 signaling pathway and down-regulated genes in metabolic pathways. Differences in lipid metabolism–related alternative splicing events, including A3SS, were also found in CLP and CLPDCA compared with sham mice. Thirty-seven RBPs, including S100a11, Ads2, Fndc3b, Fn1, Ddx28, Car2, Cisd1, and Ptms, were differentially expressed in CLP mice and the regulated alternative splicing genes(RASG) with the RBP shown to be enriched in lipid metabolic and oxidation-reduction-related processes by GO functional analysis. In KEEG analysis the RASG mainly enriched in metabolic pathway. The models of sepsis were constructed with different treatment groups, and S100a11 expression in the CLP group found to be higher than in the sham group, a change that was reversed by DCA. The alternative splicing ratio of Srebf1 and Cers2 decreased compared with the sham group increased after DCA treatment. Abnormal profiles of gene expression and alternative splicing were associated with sepsis-induced liver injury. Unusual expression of RBPs, such as S100a11, may regulate alternative splicing of lipid metabolism–associated genes, such as Srebf1 and Cers2, in the septic liver. RBPs may constitute potential treatment targets for sepsis-induced liver injury.

**Supplementary Information:**

The online version contains supplementary material available at 10.1007/s10753-024-02017-2.

## INTRODUCTION

Sepsis describes a dysregulated immune response to an infection that leads to multiple organ dysfunction. A clearer understanding of its pathophysiology is crucial for the optimization of sepsis management [1]. The metabolic and immune functions of the liver place this organ at the center of the body’s response to the systemic infection underlying sepsis, during which it participates in the bacterial clearance, production of acute-phase proteins and cytokines, and adaptation of metabolism to inflammation. It is acknowledged that sepsis-induced hepatic dysfunction aggravates the septic prognosis and is an independent predictor of mortality in the intensive care unit [2]. However, work remains to be done on mechanistic aspects of sepsis-induced liver injury to enable identification of molecular targets for diagnosis and treatment.

The current study reveals that propofol can inhibit hepatic oxidative stress, lipid peroxidation, and inflammation, which ultimately helps protect the liver from sepsis [3]. Liver LD (lipid droplet) overload is associated with increased sepsis severity and liver injury. The synthesis of hepatic LDs can be reduced by inhibiting DGAT1, which can lead to a decrease in inflammation, lipid peroxidation, and improvement in liver function [4]. During sepsis, there is a starvation response which is worsened by the rapid decline of hepatic peroxisome proliferator activated receptor alpha(PPARa) and PGC-1a levels, leading to poor mitochondria function, excess free fatty acids, lipotoxicity, and glycerol. Mice treated with the PPARa agonist pemafibrate are protected against bacterial sepsis as it improves hepatic PPARa function and reduces lipotoxicity and tissue damage [5]. Aberrations in fatty acid metabolism, including lipolysis, beta-oxidation, and lipogenesis, contribute to the pathogenesis of sepsis. Clinically, this phenomenon is addressed as steatosis in the liver after the onset of sepsis [6]. These findings confirm that dysregulated lipid metabolism is a critical factor in the hepatic pathology of sepsis and lipid metabolism in liver damage associated with sepsis.

RNA binding proteins (RBPs) regulate gene expression by binding RNA during transcription, splicing, modification, transportation, translation, and degradation. Abnormal RBP expression or altered function has suggested therapeutic targets for a number of disorders [7]. Recent studies have indicated that abnormal RBP expression has been implicated in the immune response to sepsis, and targeting has been shown to reduce inflammation and organ injury [8, 9]. RBP dysfunction might be expected to impact splicing of downstream genes with implications for the septic liver [10, 11]. Therefore, a systematic genome-wide analysis of abnormal RBP expression may illuminate splicing modifications specific to septic liver injury.

The GEO database was searched with the keywords “sepsis or severe sepsis or septic shock,” “RNA binding protein or RBP,” and “liver,” and GSE167127 data was selected to analyze RBP expression and alternative splicing. Relevant publications had 12 mice divided into sham operation (Sham), cecal ligation and puncture (CLP), and cecal ligation and puncture treatment with dichloroacetic acid (CLPDCA) groups. Transcriptomic analysis revealed that differentially expressed genes (DEGs) in CLP mice were reversed by dichloroacetic acid (DCA) therapy but by an unknown mechanism. DCA is used to treat lactic acidosis, inborn errors of mitochondrial metabolism, and diabetes [12]. It has been shown to improve sepsis survival in animal models [13] but effects on RBP and sepsis-induced liver injury remain unknown. The current study explored the impact of DCA treatment in reversing RBP and alternative splicing changes and attenuation of septic liver damage. Underlying mechanisms require further investigation.

## MATERIAL AND METHODS

### Retrieval and Processing of Public Data

We obtained publicly available data files from the Sequence Read Archive (SRA) and converted them to fastq format using the NCBI SRA Tool, fastq-dump, To ensure high quality reads, we used a FASTX-Toolkit to remove low-quality bases and evaluated the resulting clean reads with FastQC. The whole liver RNA-seq was conducted 30 hours after cecal ligation puncture(CLP) and the mice were euthanized 30 hours following surgery to collect liver tissue. Intraperitoneal administration of Dichloroacetate (DCA) was given 24 hours after surgery, with tissue collected 6 hours after DCA administration [30, h after surgery].

### Read Alignment and Differentially Expressed Gene (DEG) Analysis

The clean reads were aligned to the mouse GRCm39-M27 genome using HISAT [14], with a tolerance of up to four mismatches. We utilized the uniquely mapped reads to determine the read count and read per kilobase of exon per million fragments mapped (RPKM) for each gene to assess its expression level. To evaluate differential expression, we employed the DEseq2 software and estimated gene dispersion, fitting the negative binomial distribution model. This allowed us to assess differential expression using either the Wald or likelihood ratio test. We represent differential expression as the fold change (FC) with false discovery rate (FDR).

### Alternative Splicing Analysis

The AB Las pipeline was utilized to identify alternative splicing events (ASEs) and regulated alternative splicing events (RASEs), following previously described methods [15, 16]. The pipeline was able to detect ten different types of ASEs from splice junction reads, including exon skipping (ES), alternative 5′ splice site (A5SS), alternative 3′ splice site (A3SS), intron retention (IR), mutually exclusive exons (MXE), mutually exclusive 5′UTRs (5pMXE), mutually exclusive 3′UTRs (3pMXE), cassette exon, A3SS&ES, and A5SS&ES. Fisher’s exact test was employed to determine statistical significance between pairs of samples, using alternative and model reads as input data. The RASE ratio was defined as changes to ratios of alternatively spliced reads and constitutively spliced reads between paired samples, with a threshold of ratio ≥ 0.2 and *p*-value ≤ 0.05 set for RASE detection. Student’s *t*-test was performed to evaluate the significance of the ratio alteration, with a *p*-value of 0.05 indicating RASEs.

### Functional Enrichment Analysis

To analyze enriched terms among the differentially expressed genes (DEGs), we conducted gene ontology (GO) enrichment analysis. This involved utilizing the KOBAS 2.0 server [17] to filter significantly enriched terms. Enrichment was defined using the hypergeometric test and Benjamini-Hochberg FDR controlling procedure. Additionally, we employed Reactome (http://reactome.org) pathway profiling to further investigate functional enrichment of specific genes.

### Co-expression Analysis

To explore the regulatory relationship between RASE and DERBPs, we calculated Pearson’s correlation coefficients (PCCs). Based on the PCCs value, we classified their relationship as positive correlated, negative correlated, and non-correlated.

### Reverse Transcription qPCR Validation of DEGs and ASEs

The study used quantitative reverse-transcription polymerase chain reaction (RT-qPCR) to validate selected DEGs and ASEs in the CLP, CLPDCA, and control groups. The RT-qPCR test was performed 72 hours post cecal ligation puncture (CLP), and liver tissues were collected from the mice 72 hours after the surgery. Dichloroacetate (DCA) was administered intraperitonealy 24 hours after surgery, with tissue collected 48 hours after the DCA administration (72 hours after the surgery). Total RNA was extracted from animal samples and used to transcribe RNA into cDNA using M-MLV Reverse Transcriptase (Vazyme). The Step One Real-Time PCR System was used to perform real-time PCR with HieffTM qPCR SYBR® Green Master Mix (Low Rox Plus; YEASEN, China). The PCR protocol involved denaturation at 95 °C for 5 min, followed by 40 cycles of denaturation at 95 °C for 15 s and annealing/extension at 60 °C for 30 s. PCR amplifications were carried out in triplicate for each sample. RNA expression levels were normalized to GAPDH (Table 1).

**Table 1:** Primers Used in qPCR

Primer	Sequence (5′-3′)
S100a11-F	CATTGAGTCCCTGATTGCT
S100a11-R	AGCCAGCTCTGTGTTCAT
Srebf1-M/AS-F	GGGAAGTCACTGTCTTGGTTG
Srebf1-AS-R	ACATTTGAAGACATGCTCCA
Srebf1-M-R	GACATCGAAGACATGCTCCA
Cers2-M-F	CGGACGCCGAGATGCTCCAG
Cers2-AS-F	TAGTCTCTAGGATGCTCCAG
Cers2-M/AS-R	GAGGCTTTGGCATAGACACG
GAPDH-F	GGAGATGCTCAGTGTTGG
GAPDH-R	TGACAATGAATACGGCTACA

### Statistical Analysis

To demonstrate the grouping of samples based on the first two components, we utilized the factoextra R package to perform principal component analysis (PCA). During this analysis, we normalized the reads for each gene using Tags Per Million (TPM). We also incorporated a script named sogen to visualize next-generation sequence data and genomic annotations. For clustering, we utilized Euclidean distance and generated a heatmap in R. To compare two groups, we employed Student’s *t*-test. The data is presented as mean ± SD (standard deviation of the mean). The statistical differences among the groups were analyzed using the one-way ANOVA tool. The statistical significance was shown as follows: an asterisk (*) indicates *p* < 0.05, two asterisks (**) indicate *p* < 0.01, three asterisks (***) indicate *p* < 0.001, and four asterisks (****) indicate *p* < 0.0001.

## RESULTS

### Liver Gene Expression Profiles in Sham, CLP, and CLPDCA Group

**Fig. 1:**
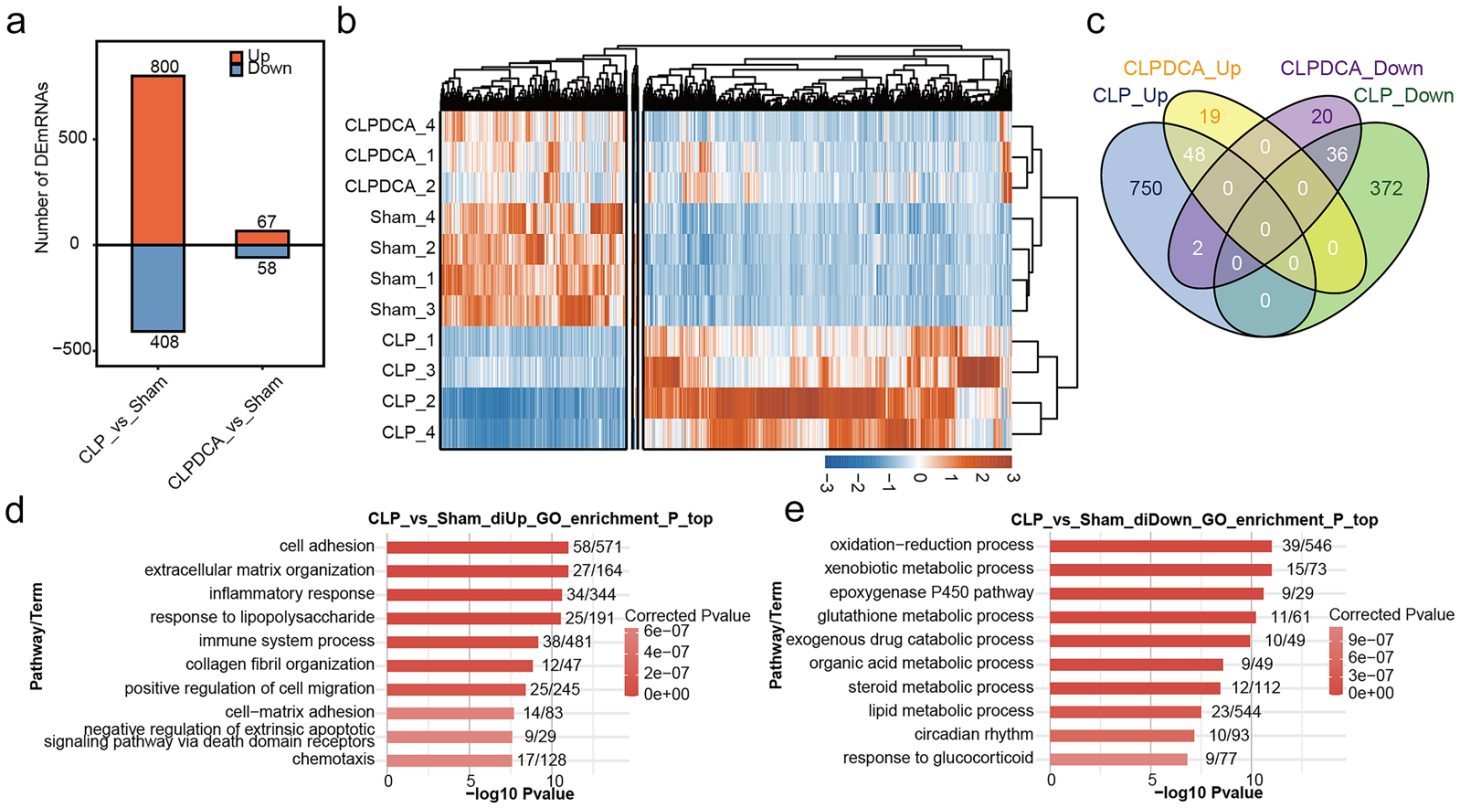
Gene expression profile of mouse liver tissue. **a** Significant DEGs in CLP and CLPDCA septic mice. **b** Expression heatmap of significant DEGs among CLP, CLPDCA, and sham. **c** Venn diagram showing overlap of DEGs in CLP and CLPDCA samples. **d** Bar plot of the most enriched GO biological processes of genes up-regulated in CLP. **e** Bar plot of the most enriched GO biological processes of genes down-regulated in CLP.

A total of 1208 DEGs were identified from 4 sham, 4 CLP, and 3 CLPDCA samples, including 800 up-regulated and 408 down-regulated genes between CLP and sham (Fig. 1a). A further 125 DEGs were identified between the CLPDCA and sham groups, including 67 up-regulated and 58 down-regulated genes. Inspection of Figure 1a shows that treatment with DCA had partially reversed the changes in gene expression found between the sham and CLP groups. Hierarchical cluster analysis of significantly different expression patterns in different groups of samples found DEG patterns to be more similar between CLPDCA and sham groups than between CLP and sham (Fig. 1b). Analysis of DEG overlap identified 48 co-up-regulated and 36 co-down-regulated DEGs in the CLP and CLPDCA treatment groups relative to the sham group. However, a comparison of sham and CLP groups revealed 750 up-and 372 down-regulated genes. The differences in numbers of up- and down-regulated genes support the view that treatment of CLP mice with DCA partially reversed abnormal gene expression induced by CLP (Fig. 1c).

Functional analysis was performed on DEGs between CLP and sham. GO analysis showed co-up-regulated genes to be enriched in the biological processes of cell adhesion, inflammatory response, immune system processes, positive regulation of cell migration, negative regulation of external apoptotic signal pathways through death domain receptors, chemotaxis, and other functional pathways (Fig. 1d). Co-down-regulated DEGs were enriched in oxidation-reduction and lipid metabolic processes (Fig. 1e). Kyoto Encyclopedia of Genes and Genomes (KEGG) analysis identified up-regulated genes as being involved in cytokine-cytokine receptor interaction and IL-17 signaling pathway and down-regulated genes in metabolic pathways. Genes that were up-regulated in the comparison of CLP with CLPDCA were enriched in FoxO signaling and glucagon signaling pathways (supplement1).

The involvement of lipid metabolism–related genes in the impact of sepsis on liver tissue was highlighted by the outcomes of the functional analyses described above. Inflammation/immune and cell apoptotic–related genes, including cytokine-cytokine receptor interaction and the IL-17 signaling pathways, also featured prominently in the differential expression results. It may be suggested that the actions of DCA on signaling of the transcription factor, FoxO, and hormone, glucagon, are involved in reversing abnormal gene expression. Lipid metabolism was also highlighted as a process worthy of further investigation as a potential therapeutic target.

### Alternative Splicing Patterns in Sham, CLP, and CLPDCA Group

**Fig. 2:**
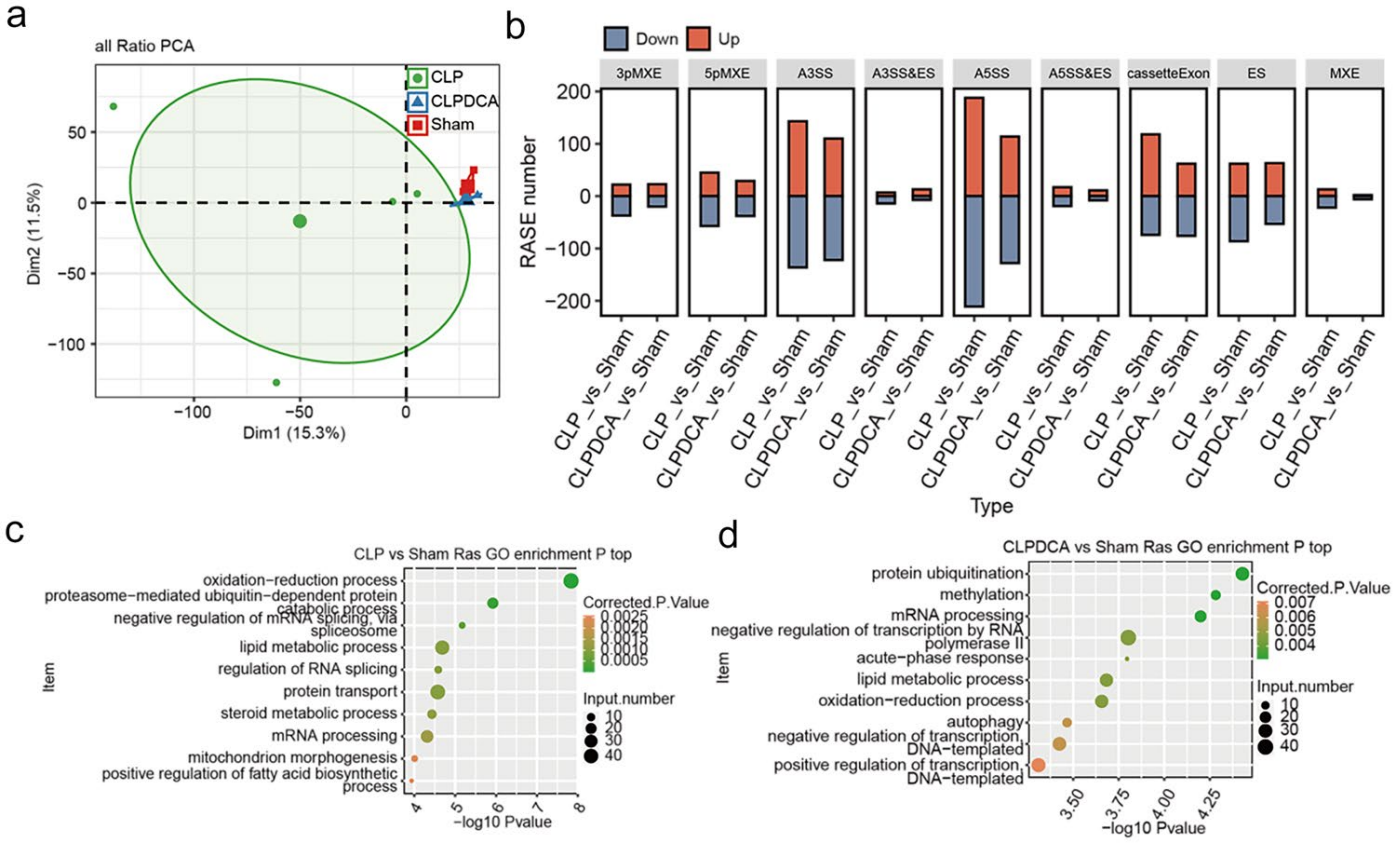
Regulated alternative splicing events in septic mice. **a** PCA of RASE ratios with confidence ellipse for all groups. **b** Bar plot of RASEs in CPL *versus* sham liver and CLPDCA *versus* sham liver. **c** Scatter plot of enriched GO terms relating to ASEGs in CPL *versus* sham liver. **d** Scatter plot of enriched GO terms relating to ASEGs in CPLDCA *versus* sham liver.

AS events at nine variable splicing sites, including A3SS, A5SS, and ES, were found from RNA-Seq data. Hierarchical clustering analysis showed that the 4 CLP samples formed one cluster, and the three sham and three CLPDCA samples formed a second cluster. Thus, the variable splicing patterns of genes in CLPDCA and sham were more similar to one another than either was to CLP (Fig. 2a). RASEs present in CLP and CLPDCA groups relative to sham were identified by *t*-test, and the most significant were A3SS and A5SS with the cassette exon and ES following (Fig. 2b). The implication of the findings above is that the pattern of A3SS and A5SS splicing may be associated with gene expression in the septic liver.

Genes associated with alternative splicing events (ASEGs) were subjected to GO functional analysis. Those differentially expressed between the CLP and sham groups showed enrichment for the processes of oxidation-reduction, lipid metabolism, mitochondrial morphogenesis, and positive regulation of fatty acid biosynthesis (Fig. 2c). Those differentially expressed between CLPDCA and CLP were enriched for protein ubiquitination, lipid metabolism, redox balance, autophagy, DNA template–dependent negative and positive regulation of transcription (Fig. 2d). These results give further confirmation of the involvement of lipid metabolism and oxidation-reduction reactions in sepsis and also show that DCA treatment ameliorates the sepsis-dependent changes by acting on the same pathways.

**Fig. 3:**
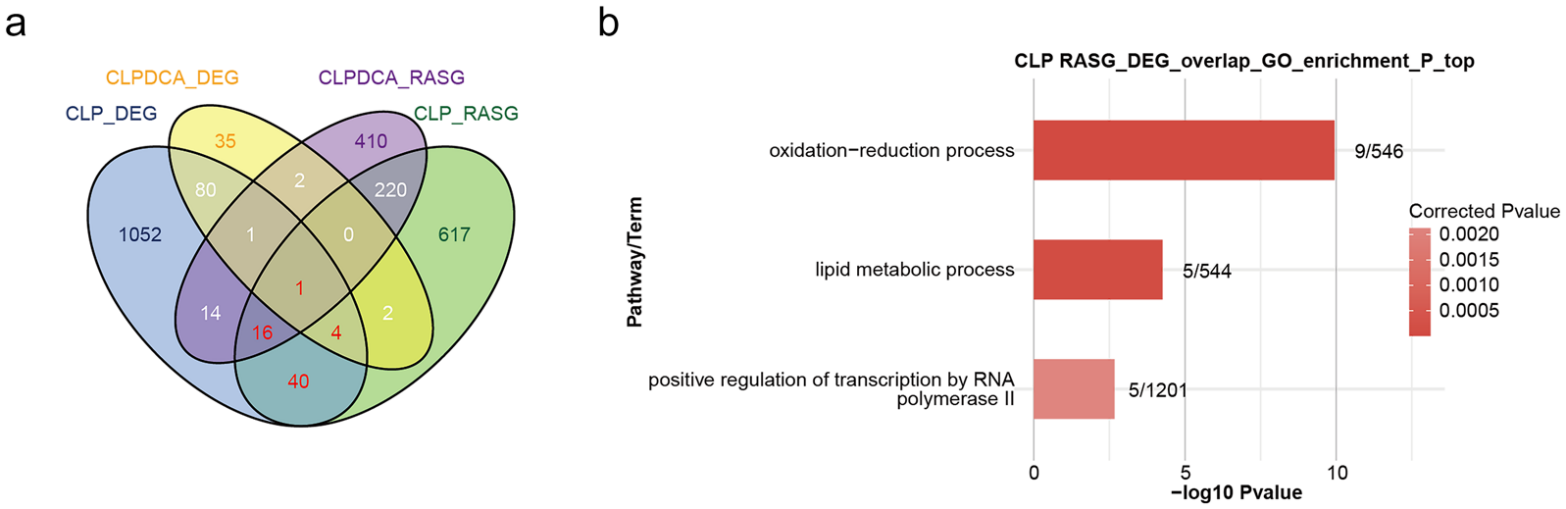
Regulated alternative splicing events. **a** Venn diagram showing the overlap of ASEGs and DEGs. **b** Bar plot of enriched GO terms relating to the overlapping genes in **a**.

Overlap analysis of DEGs and ASEGs between CLP and sham revealed 61 genes were differentially expressed between the two groups. Similar analysis of the CLPDCA and CLP groups showed only four genes with differential expression (Fig. 3a). GO analysis of the differences between CLP and sham showed enrichment for redox processes, lipid metabolism, and regulation of transcription by RNA polymerase II (Fig. 3b).

A comparison of AS event ratios of the CLP and sham groups revealed that 390 events were up-regulated and 396 down-regulated. However, a similar comparison of CLPDCA with sham revealed only 43 up-regulated and 66 down-regulated variable alternative splicing events. Again, it can be seen that the pattern observed for DEGs has been repeated. Abnormal splicing in the septic liver may cause tissue damage, and treatment with DCA reverses the effect by reversing the changes in abnormal splicing.

**Fig. 4:**
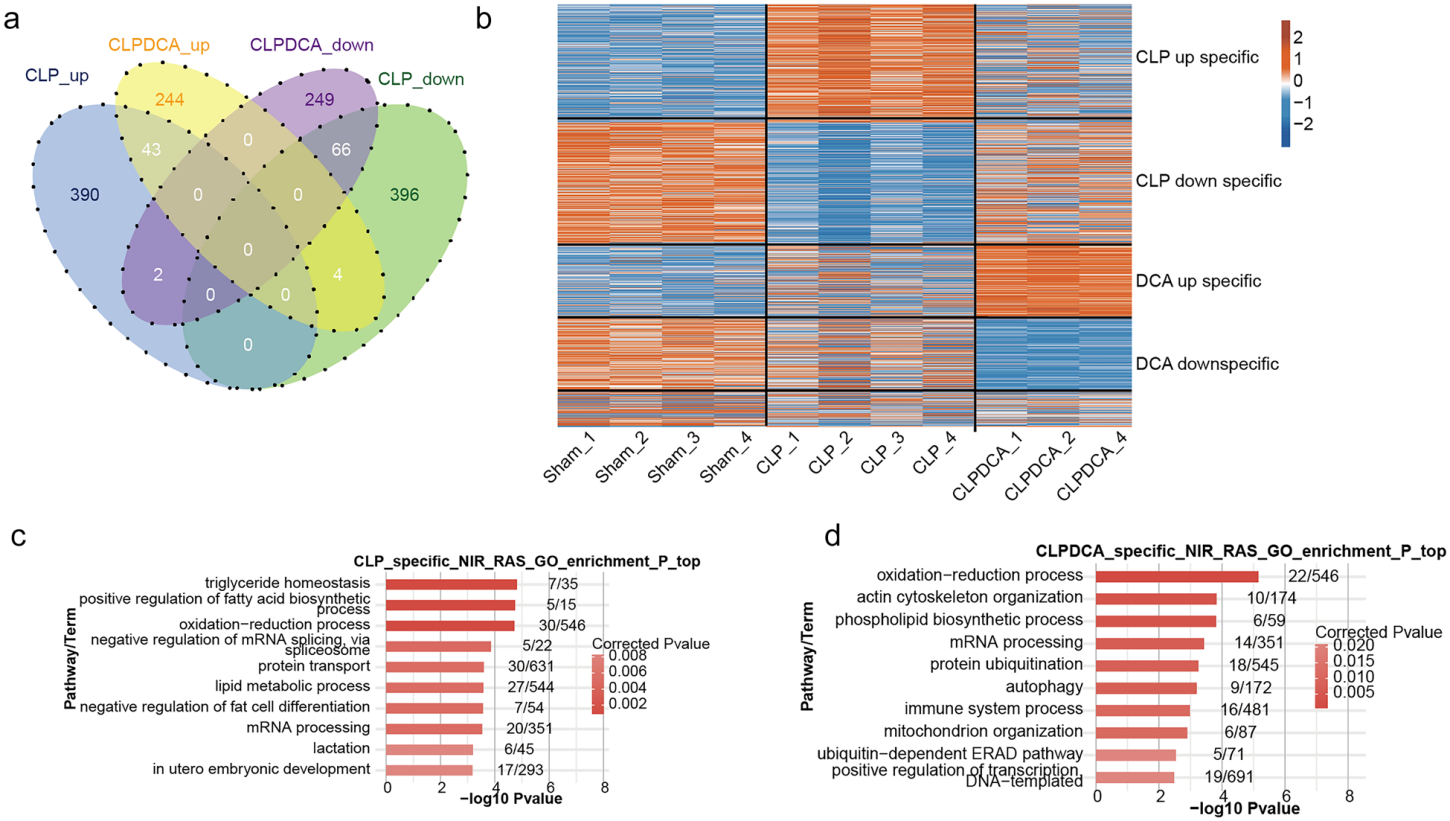
Differential AS events in the CLP- and CLPDCA-treated liver. **a** Venn diagram of overlapping AS events in CLP *versus* sham and CLPDCA *versus* sham liver. **b** Hierarchical clustering heat map of all significant ASEG ratios. The AS filter included all detectable splice junctions with at least 80% of samples having ≥ 10 splice junction supporting reads. **c** Bar plot of the most enriched GO biological processes of the CLP-specific ASEGs from **b**. **d** Bar plot of the most enriched GO biological processes of the CLPDCA-specific ASEGs from **b**.

Up- and down-regulation of differential AS events in CLPDCA tissue were 244 and 249 compared with CLP, indicating that CLPDCA reversed the abnormal gene expression profile (Fig. 4a). Cluster analysis carried out by screening the covariant AS events with read number > 10 in at least 80% of the samples showed that the four CLP samples were clustered in one group and the 3 sham and 3 CLPDCA samples were clustered in a second group (Fig. 4b). Up-regulation of AS events with CLP treatment may be associated with septic liver damage, and up-regulation of AS events with DCA treatment reversed the CLP effect and may indicate a protective effect on the septic liver. GO analysis of the genes involved in the AS events above was performed.

The 10 most enriched pathways involved in biological processes relating to the AS genes identified above included triglyceride homeostasis, positive regulation of fatty acid biosynthesis, redox processes, negative regulation of mRNA splicing by spliceosomes, protein transport, lipid metabolism, negative regulation of adipocyte differentiation, lactation, and *in utero* embryonic development (Fig. 4c). GO analysis of DCA-specific AS events indicated that redox processes, actin cytoskeleton, phospholipid biosynthesis, RNA processing, protein ubiquitination, autophagy, immune system processes, mitochondrion organization, ubiquitin-dependent ERAD pathways, and positive transcriptional regulation of DNA templates (Fig. 4d).

**Fig. 5:**
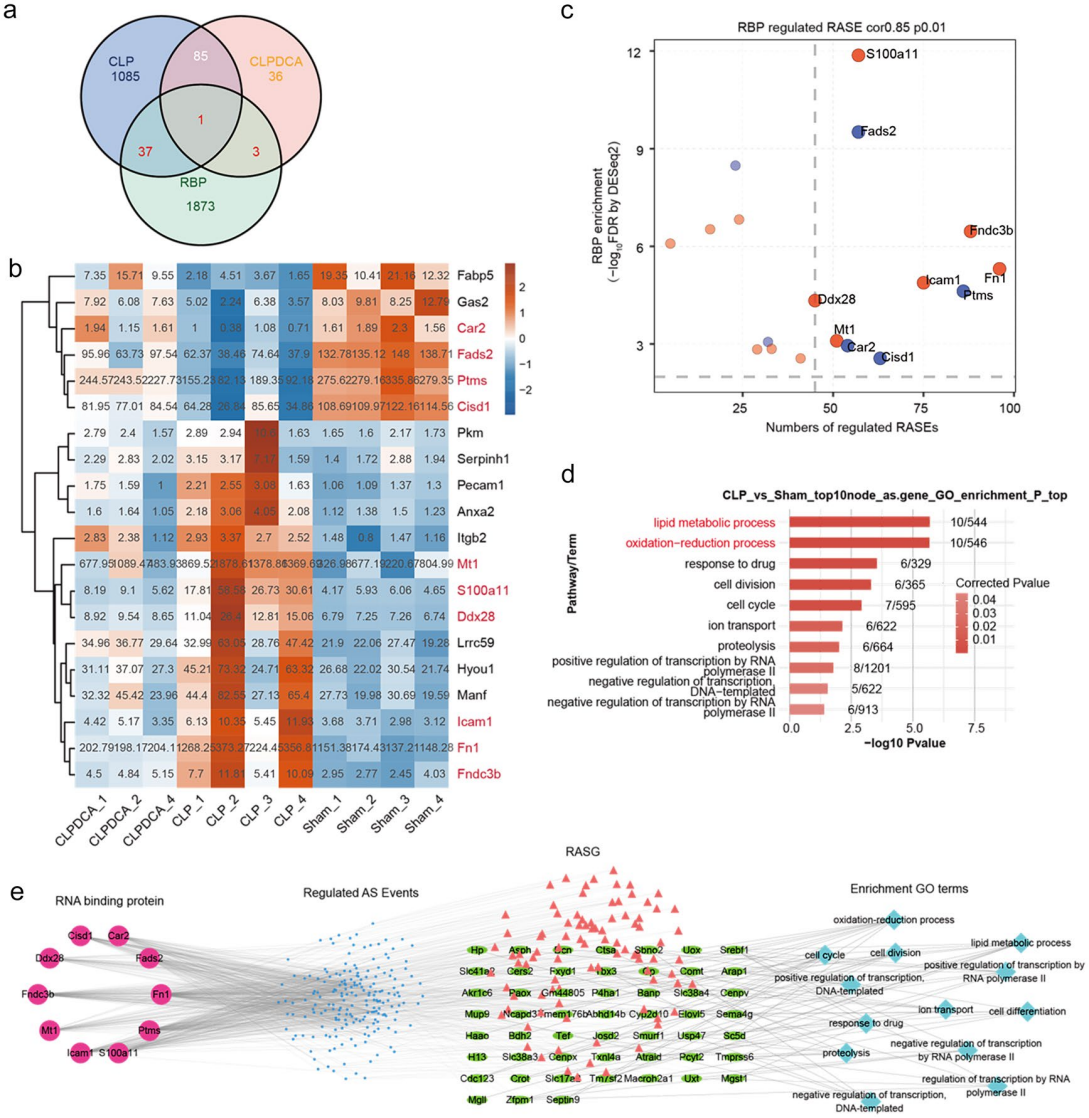
Interaction network of RNA-binding proteins and alternative splicing-associated genes. **a** Venn diagram showing overlapping differentially expressed ASEGs and RBP genes in septic mice. **b** Heatmap of differentially expressed RBP genes in CLP samples. RBPs were filtered by expected fragments per kilobase of transcript per million fragments mapped (FPKM) ≥1 in 80% of samples. The color key from blue to red indicates *z*-score color range. **c** The scatter plot shows ASEGs by CLP *versus* sham co-expressed with differentially expressed RBP genes from **b**. **d** Enriched GO biological processes of ASEGs co-disturbed with the top 10 differentially expressed RBPs. **e** The co-deregulation in CLP-treated liver of AS network and RBPs (left; circle size, number of connections), AS events (middle left), ASEGs (center right), and enriched GO biological process terms of co-disturbed ASEGs and RBP genes (right).

Human RBP genes were intersected with the DEGs found above to be associated with CLP/sham and CLPDCA/CLP differences. A total of 37 RBP genes were up- or down-regulated in the CLP group compared with the sham (Fig. 5a). Treatment with DCA reversed the expression changes of the RBP genes brought about by CLP (Fig. 5a). Some of the RBP genes identified are likely to be involved in AS events. The 20 RBPs showing the greatest degree of up- or down-regulation in the CLP group were screened (Fig. 5b), and a co-expression network of hub RBPs and ASEGs was constructed. RBP S100A11 was found to be likely to regulate multiple differential AS events (Fig. 5c). GO and KEGG analyses of the 10 RBPs co-expressed most frequently with ASEGs were carried out. GO analysis showed enrichment in lipid metabolism, redox processes, drug responses, cell division, cell cycle, ion transport, proteolysis, positive and negative regulation of transcription by RNA polymerase II, and DNA-dependent negative regulation (Fig. 5d).

**Fig. 6:**
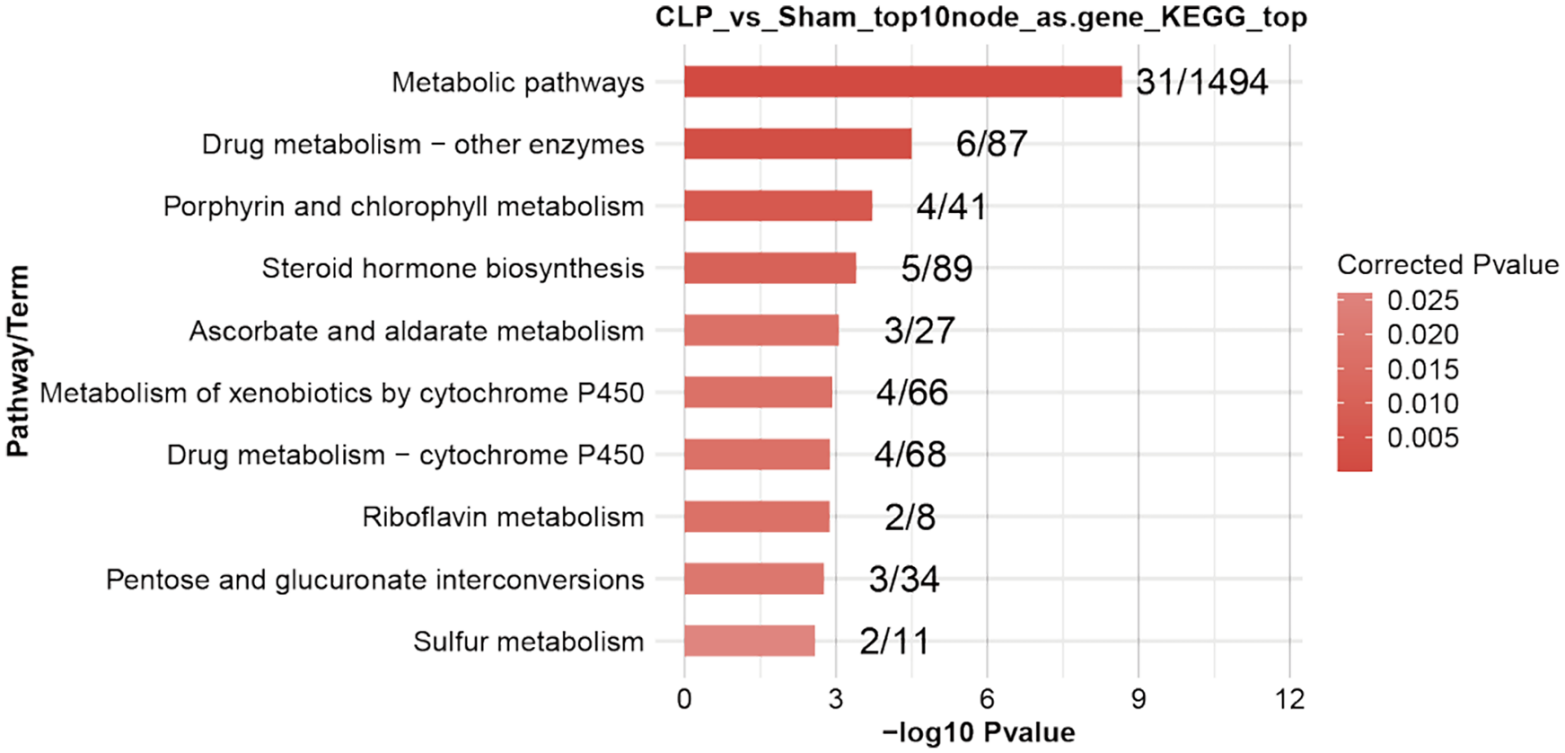
The most enriched KEGG pathways of ASEGs co-disturbed with the top 10 differentially expressed RBPs.

In order to investigate the regulatory relationship between RASE and DERBPs, a co-expression analysis was conducted. Pearson’s correlation coefficients (PCCs) were calculated and used to categorize their association as positively correlated, negatively correlated, or non-correlated. Relationship pairs between DERBPs and RASE that exhibited a correlation coefficient value of at least 0.85 and a *p*-value of no greater than 0.01 were identified through screening. An interaction network of differentially expressed RBPs and ASEGs suggested that CLP-induced up- or down-regulation of liver RBPs could be reversed by DCA treatment, affecting splicing of downstream genes (Fig.5e). A KEGG analysis showed the ASEGs co-disturbed with differentially expressed RBPs enriched in metabolic pathways, drug metabolism, porphyrin and chlorophyll metabolism, steroid biosynthesis, ascorbic acid and uronate metabolism, cytochrome P450 metabolism, riboflavin metabolism, pentose and gluconate interconversion, and sulfur metabolism (Fig. 6).

### Co-expression Analysis Between Sepsis-Regulated RBPs and NIR

**Fig. 7:**
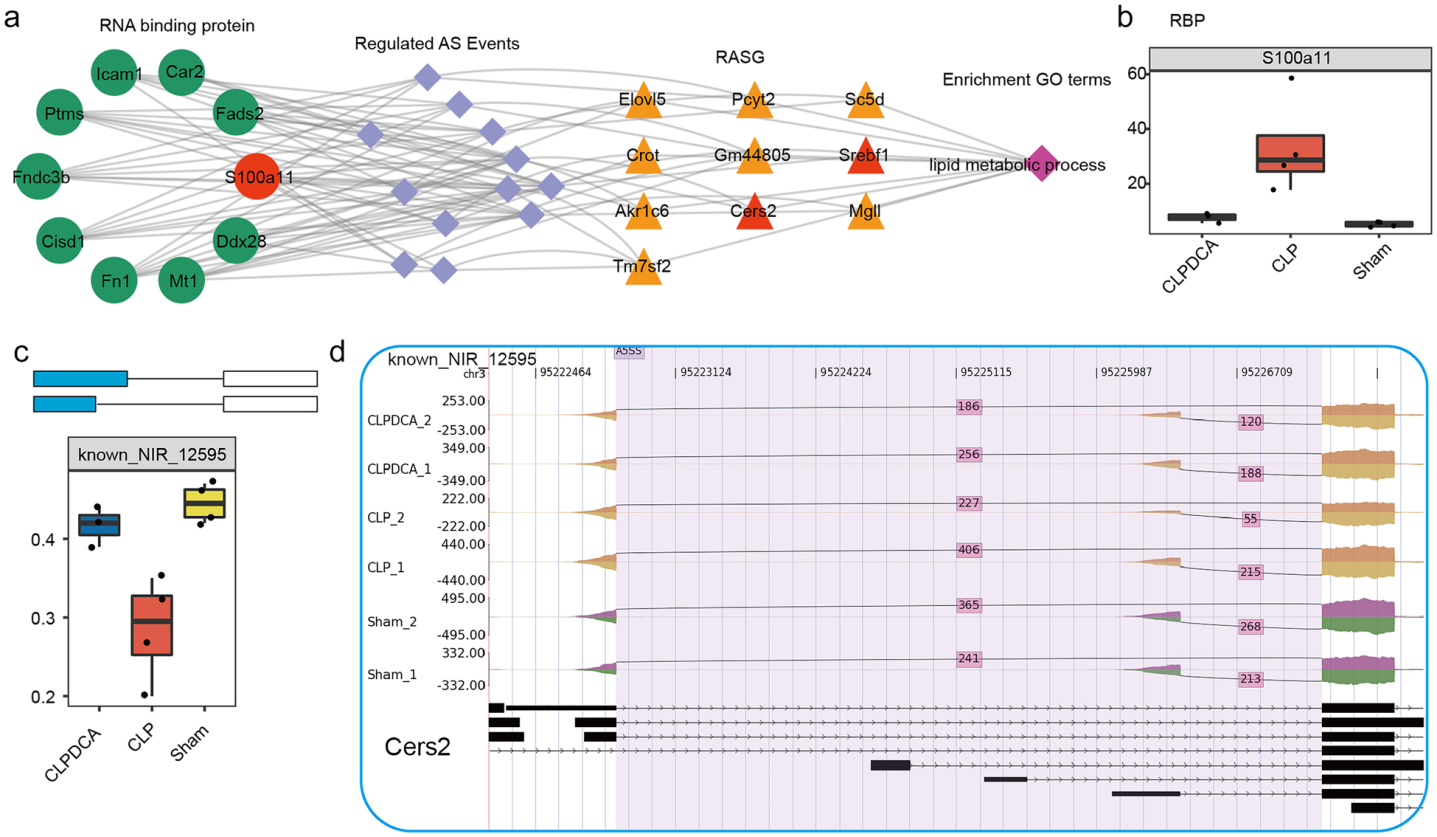
Abnormally expressed RBP genes with effects on lipid metabolism–associated genes. **a** Network showing deregulation of lipid metabolism by abnormal RBP expression. **b** Box plot showing expression of S100a11 in CLP, CLPDCA, and sham samples. **c** Box plot showing the splicing ratio profile of the Cers2 gene across 11 samples. **d** Visualization of junction read distribution of Cers2 gene in samples from different groups. Splice junctions are labeled with SJ read number.

**Fig. 8:**
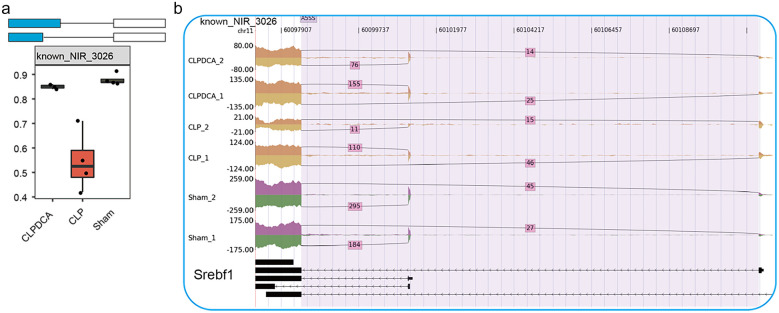
Gene expression and splicing regulation of lipid metabolism–associated genes. **a** Box plot showing the splicing ratio profile of the Serbf1 gene across 11 samples. **b** Visualization of junction reads distribution of Serbf1 gene in samples from different groups. Splice junctions are labeled with SJ read number.

An interaction and co-expression network was constructed to encompass all connections among RBPs, AS events, and ASEGs. The dysregulation of RBP gene expression caused by CLP was found to be reversed by DCA treatment with a likely impact on AS events (Fig. 7a and b). Genes involved in metabolic pathways, in particular, lipid metabolism, which is subject to AS, were the focus of the investigation. The abnormally expressed RBP, S100A11, was found to affect AS of some genes encoding products involved in lipid metabolism, such as SREBF1 and CERS2 (Figs. 7c, d and 8).

### Analysis the Genome-Wide AS in Lipid Metabolism and Oxidation-Reduction–Related Genes

**Fig. 9:**
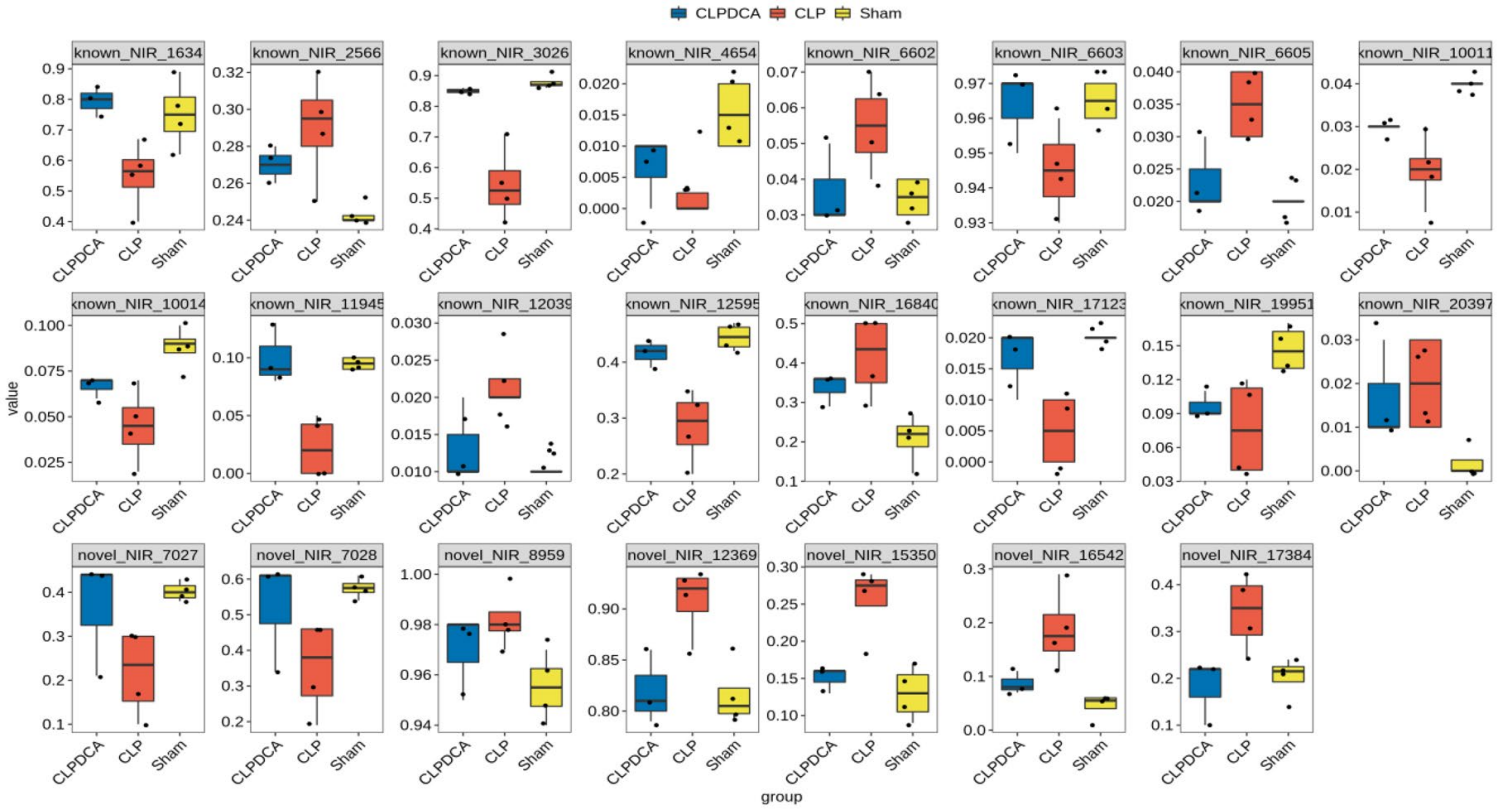
Top ten node lipid metabolic and oxidation-reduction–related alternative splicing genes and relative alternative splicing events ratio in Sham, CLP, and CLPDCA group.

In the CLP group, the top ten alternative splicing event genes were Cers2, Tm7sf2, Crot, Pcyt2, Mgll, Sc5d, Elovl5, Akr1c6, Srebf1, and Gm44805 when compared with the Sham group. These genes were mainly enriched in metabolic lipid processes in the GO enrichment analysis of biological processes. Additionally, Cyp2d10, Tm7sf2, Paox, Uox, Sc5d, P4ha1, Haao, Akr1c6, Cp, and Bdh2 were the alternative splicing event genes that were mainly enriched in the oxidation-reduction process in the GO enrichment analysis of biological processes. The alternative splicing ratio profile of lipid metabolism and oxidation-reduction–related genes is shown in Table 2 and Fig. 9.

**Table 2:** Lipid Metabolic and Oxidation-reduction–related RASG

**Source**	**Target**	**Type**	**Type**
known_NIR_10011	Tm7sf2	RASE	RASG
known_NIR_10014	Tm7sf2	RASE	RASG
known_NIR_11945	Bdh2	RASE	RASG
known_NIR_12039	Uox	RASE	RASG
known_NIR_12595	Cers2	RASE	RASG
known_NIR_1634	P4ha1	RASE	RASG
known_NIR_16840	Paox	RASE	RASG
known_NIR_17123	Gm44805	RASE	RASG
known_NIR_19951	Sc5d	RASE	RASG
known_NIR_20397	Elovl5	RASE	RASG
known_NIR_2566	Pcyt2	RASE	RASG
known_NIR_3026	Srebf1	RASE	RASG
known_NIR_4654	Akr1c6	RASE	RASG
known_NIR_6602	Cyp2d10	RASE	RASG
known_NIR_6603	Cyp2d10	RASE	RASG
known_NIR_6605	Cyp2d10	RASE	RASG
novel_NIR_12369	Cp	RASE	RASG
novel_NIR_15350	Crot	RASE	RASG
novel_NIR_16542	Mgll	RASE	RASG
novel_NIR_17384	Gm44805	RASE	RASG
novel_NIR_7027	Cyp2d10	RASE	RASG
novel_NIR_7028	Cyp2d10	RASE	RASG
novel_NIR_8959	Haao	RASE	RASG

**Fig. 10:**
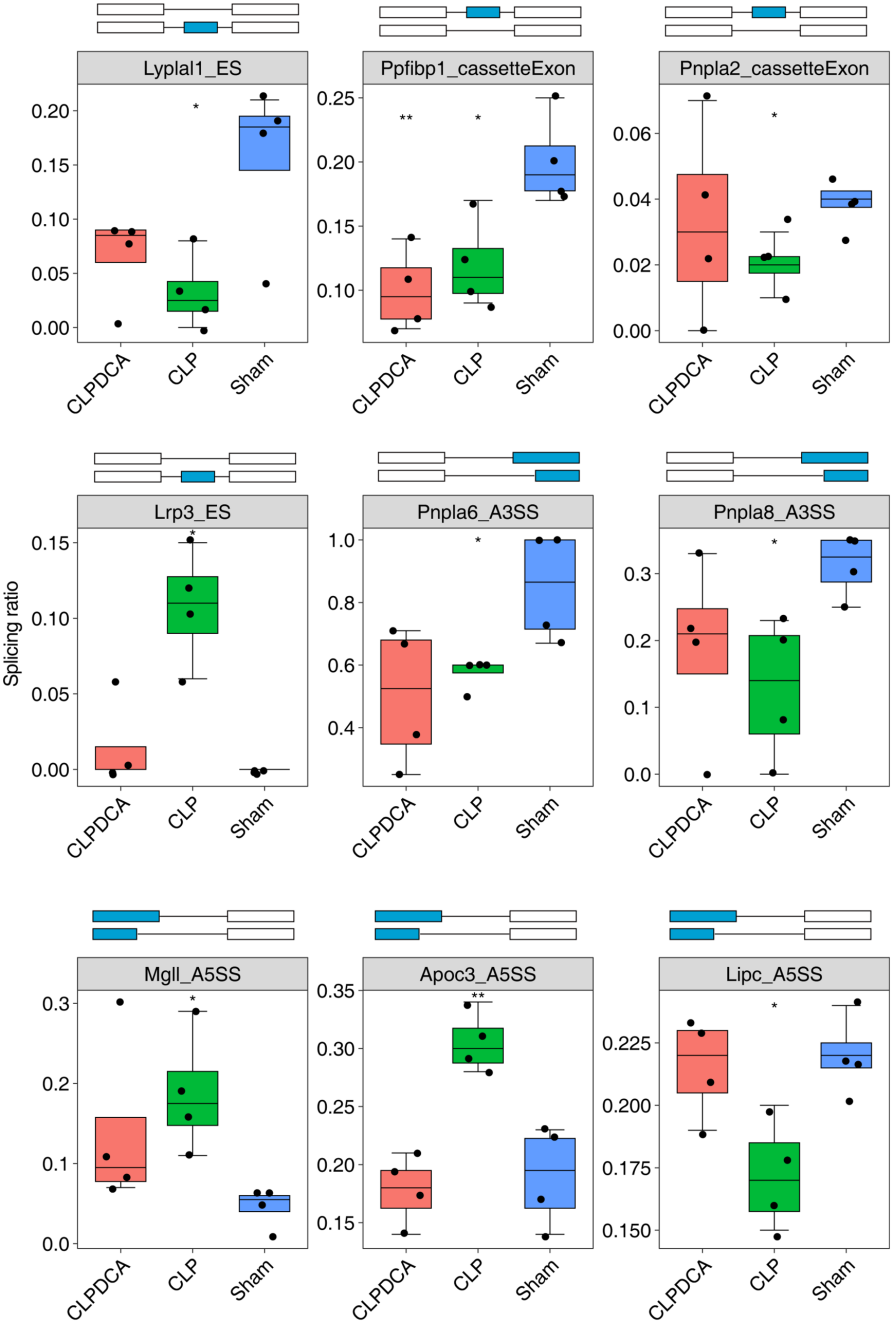
Splicing ratio profile of specific lipid metabolism–related genes in Sham, CLP, and CLPDCA.

Furthermore, in the CLP group, the splicing ratio of Lyplal, Ppfibp1, Pnpla2, Lrps3, Pnpla6, Pnpla8, Mgll, Apoc3, and Lipc exhibits significant differences when compared to the Sham group. Additionally, the splicing ratio of P4ha1, Akr1c6, Cyp2d10, Tm7sf2, Bdh2, Gm44805, and Sc5d decreased in the CLP group compared to the Sham group. On the other hand, the Pcyt2, Uox, Paox, and Elovl5 genes showed an increase in their splicing ratio in the CLP group. The CLP group showed a significant reduction in the reads of ES in Lyplal1 compared to the Sham group. The reads of A3SS were increased in Mgll and Apoc3, and there was a significant reduction in the reads of cassette exon in Ppfibp1 and Pnpla2. as illustrated in Fig. 10. These findings suggest that the alternative splicing of genes related to lipid metabolism and oxidation might be involved in the development of liver damage during sepsis.

### Validation of Co-expressed RBPs and ASEs

**Fig. 11:**
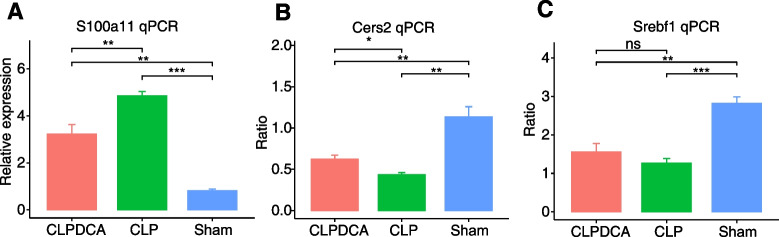
Validation of RASEs and RBP. **a** Box plot showing expression status of S100a11 in CLP, CLPDCA, and Sham samples by qPCR validation. **b** Box plot showing splicing ratio profile of the Cers2 splicing event by qPCR validation. **c** Box plot showing the splicing ratio profile of the Serbf1 splicing event by qPCR validation. Data are shown as mean ± SD, *p < 0.05, **p < 0.01, ****p < 0.0001.

We observed abnormal alternative splicing of genes related to lipid metabolism in the liver tissues of septic animal model. These genes include Cers2, Tm7sf2, Crot, Pcyt2, Mgll, Sc5d, Elovl5, Akr1c6, Srebf1, Gm44805, Lylal1, Pnpla, Lyplal1, Ppfibp1, Pnpla2, Lrp3, Pnpla6, Apoc3, Pnpla8, Mgll, Apoc3, and Lipc. Dysregulated expression of cers2 and srebf1 has been reported in sepsis, and they are considered hub genes of lipid metabolism. However, there is a lack of evidence on the expression and alternative splicing of other lipid metabolism–related genes in sepsis. To address this, Considering the evidence for the genes and alternative splicing linked to lipid metabolism in sepsis, we validated only cers2 and srebf1 in a septic animal model. S100a11 expression levels were significantly greater in the CLP group than in the sham group, although DCA eliminated this difference. Furthermore, the alternative splicing ratio of Srebf1 and Cers2 was reduced compared with the sham group and increased after DCA treatment. These results suggested that S100a11 is relevant to the pathogenesis of sepsis-induced liver damage. Its mechanism may be related to Srebf1 and Cers2 alternative splicing, regulated by S100a11 (Fig. 11).

## DISCUSSION

Transcriptomic sequencing (RNA-seq) has been used to reveal molecular phenotypic changes underlying physiological conditions and disease progression [18]. Variations in gene expression are acknowledged to affect physiological parameters. The current study analyzed RNA-seq data from liver tissue in an animal model of sepsis and found altered gene expression and varying alternative splicing associated with sepsis progression. DCA treatment was found to reverse the aberrant gene expression and ameliorate liver injury [19]. DCA activates pyruvate dehydrogenase, affecting oxidative metabolism [20, 21] and attenuated lactic acidosis during orthotopic liver transplantation (OLT) [22] producing a hepato-protective effect [23]. DCA also protected Nnt−/− mice from developing high-fat diet (HFD)-induced non-alcoholic fatty liver disease (NAFLD) which may be due to the reactivation of pyruvate dehydrogenase, restoring the capacity of the pyruvate-supported liver mitochondria to manage peroxide [24]. Thus, the protective effect of DCA against sepsis-induced liver injury may rely on metabolic reprogramming.

The GO functional analysis showed up-regulation of genes involved in immune/inflammatory response, apoptosis, and cell migration, whereas down-regulated genes tended to be associated with redox reactions and lipid metabolism. The current study’s findings agree with previous reports in indicating that variable gene expression accompanies disease progression. Lipid metabolism has been associated with the development of sepsis [25] and consequent liver damage [26]. However, mechanistic connections between liver injury and sepsis progression remain to be elucidated.

RBPs are involved in liver inflammation and lipid homeostasis, and the expression of immune-associated RBPs is a biomarker for predicting the targeted therapeutic response of liver cancer and patient survival [27]. The current study found that CLP-induced abnormal expression of RBPs, such as S100a11, ads2, Fndc3b, Fn1, Ddx28, Car2, Cisd1, and Ptms, and DCA treatment reversed these effects. Sepsis-related RBPs were found to regulate alternative splicing of downstream genes involved in lipid metabolism. Previous studies have reported that the liver RBP, HuR, regulates lipid homeostasis in response to a high-fat diet [28], and HuR promoted miRNA-mediated up-regulation of NFI-A protein expression in Myeloid-derived suppressor cells (MDSCs) and enhanced resistance to uncontrolled infection in septic mice [29]. HuR deficiency leads to inflammation and fibrosis of the liver [30]. The cold-inducible RBP, CIRP, induces inflammatory responses in hemorrhagic shock and sepsis [31] and activation of splenic T cells dependent on the TLR4 [32]. Thus, targeting of RBPs, such as with anti-peptides of CIRP, reduced sepsis-induced inflammation and organ damage in septic mice [8]. Thus, RBPs are attractive candidates for therapeutic targeting with essential functions in liver immunity, metabolic diseases, and sepsis.

RBPs regulate the development of a number of liver diseases through variable splicing, which is otherwise a source of protein diversity [33, 34]. Degradation of the RBP, SRSF3, led to abnormal splicing in the liver, promoting disease progression [35]. The dysregulation of AS associated with sepsis means that RBPs may be potential targets for the treatment of septic liver injury. Abnormal splicing of acidic sphingomyelinase 1 (SMPD1) mRNA is known to result in altered enzyme activity and an impact on the development of sepsis [10]. Abnormal alternative splicing of the myosin phosphatase gene resulted in reduced enzyme activity, oxidative stress, and altered NO vasodilator reserve in the early and late stages of the mouse model of LPS-induced sepsis, affecting disease progression [36]. The current findings demonstrate that DCA reversed abnormal alternative splicing events in the liver tissue in sepsis with ASEGs enriched in lipid metabolism and oxidation-reduction–related genes. Disordered lipid metabolism is known to affect alternative splicing of mRNA [37-39]. In summary, altered RBP function may lead to AS abnormalities associated with liver injury in sepsis, and RBPs may be a therapeutic target.

The S100a11 calcium–binding member of the S100 family is up-regulated during sepsis [40] and promotes liver steatosis via the RAGE-mediated AKT-mTOR signaling pathway [41] and foxo1-mediated autophagy and lipogenesis [42]. Based on the available evidence, it appears that S100a11 has the potential to significantly affect the activity of SREBF1, which is a vital transcription factor involved in liver lipid metabolism. It has been observed that SREBF1 can enhance lipid synthesis while reducing lipid degradation, leading to the accumulation of lipids in the liver and improper regulation of autophagy. These findings indicate that S100a11 may have a crucial role to play in the regulation of liver lipid metabolism through its impact on SREBF1 [43, 44]. Indeed, KDM1A-mediated attenuation of SREBF1 activity underlies suppression of *de novo* lipogenesis by oxidative stress [45]. Moreover, Down-regulation of PPARG and SREBF1 in response to PER2 silencing highlights the importance of circadian clock signaling for lipogenesis regulation [46].

CerS2 maintains normal cell division through the MAD2‐MKLP2‐CPC axis [47] but down-regulation of CerS2 resulted in LC (long-chain) ceramide accumulation and growth arrest, unaccompanied by apoptosis [48]. Ceramide synthase is known to be enhanced in LPS-mediated septic shock in Cers2-deficient mice [49], and inhibition of ceramide synthesis prevented diabetes, steatosis, and cardiovascular disease in rodents [50]. The current study found SREBF1/CERS2 to be predicted targets of S100a11. In conclusion, lipid metabolism appears to be involved in sepsis-induced liver injury. RBP dysfunction disturbs alternative splicing of lipid metabolism genes, such as SREBF1/CerS2, indicating RBPs as possible therapeutic targets for sepsis-induced liver injury. Further investigations are required to elucidate the mechanisms involved.

We have identified several areas that require improvement in our study. To investigate sepsis liver tissue, we systematically analyzed RNA-seq data from document number one and established a sepsis animal model. We validated the gene expression of S100a11, srebf1, and cers2 using RT-qPCR, but it would be more beneficial to perform additional tests such as immuno-histochemistry and Western blot to detect protein expression levels in the animal model. It is also necessary to continuously and dynamically observe the changes in S100a11, srebf1, cers2, and liver injury markers in the animal model and conduct correlation analysis. Additionally, RNA-binding protein immuno-precipitation experiments would be helpful to elucidate further the intrinsic connections between S100a11, srebf1, cers2, and the pathogenesis of sepsis liver injury.

## CONCLUSION

The liver is a vital organ for metabolism and immunity, and it plays a critical role in sepsis development. Our study focused on septic liver tissue in mice and revealed numerous differentially expressed genes, alternative splicing events, and RNA-binding proteins with abnormal expression. Through bioinformatics analysis, we identified the relationship between abnormal RNA-binding proteins and variably spliced events and found that RNA binding proteins like S100A11 can indirectly impact septic liver injury by regulating downstream genes associated with lipid metabolism, such as SREBF1 and CERS2. These discoveries offer valuable insight into the function and mechanism of RNA-binding proteins in sepsis, and they could lead to the identification of new therapeutic targets for septic liver injury.

## SUPPLEMENTARY INFORMATION

Below is the link to the electronic supplementary material.

Supplementary file1 (ZIP 38655 KB)

## Data Availability

The published article and its additional information files contain all of the data generated or analyzed during this investigation. The datasets supporting the findings of this study are available in the NCBI Gene Expression Omnibus and can be accessed using the GEO series accession number (GSE167127).
