# Supplementary material for: Regulation of Alternative Splicing of Lipid Metabolism Genes in Sepsis-Induced Liver Damage by RNA-Binding Proteins
Source: Inflammation. 2024 May 9;47(6):1952–68. doi: 10.1007/s10753-024-02017-2 (PMC11607010; doi:10.1007/s10753-024-02017-2)
Supplement: Supplementary file 1 — Supplementary file1 (ZIP 38655 KB) [file 10753_2024_2017_MOESM1_ESM.zip › Sup5_AS_GO.zip!/Sup5_AS_GO/ras/CLP_vs_Sham/Ras_GO_analysis/Ras_GO_enrichment_web_P.html]

GO enrichment analyze


# GO enrichment analyze result


| Term | Database | ID | Input number (734) | Background number (20961) | P-Value | Corrected P-Value | Input | Hyperlink |
| --- | --- | --- | --- | --- | --- | --- | --- | --- |
| oxidation-reduction process | Gene Ontology | GO:0055114 | 47 (6.40%) | 546 (2.60%) | 1.46188469108e-08 | 3.26000286111e-06 | Oxnad1  Grhpr  Aox3  Fmo5  Acox2  Idh1  Acox1  Ero1b  Dhrs3  Akr1e1  Sc5d  Fxn  Cyp2c40  Bdh2  Pam  Tm7sf2  Cryz  Paox  Cbr4  Cyp4a31  Ndufs2  Prxl2c  Cyp2r1  Dio1  Dhrs7b  Uox  Cyp3a59  Cmah  P4ha1  Adhfe1  Hsd17b2  Hsd17b6  Akr1c20  Aspdh  Cyp2d10  Cp  Cyb5r3  Maob  Cyp4b1  Rtn4ip1  Haao  Rdh13  Aldh16a1  Akr1c6  Nnt  Alkbh2  Alkbh3 | web link |
| proteasome-mediated ubiquitin-dependent protein catabolic process | Gene Ontology | GO:0043161 | 21 (2.86%) | 178 (0.85%) | 1.20119293423e-06 | 0.000133933012167 | Smurf1  Ube2i  Btrc  Kctd17  Tbl1xr1  Nedd4l  Arrb2  Znrf1  Huwe1  Ube2d3  Fbxw5  Pcnp  Dcaf11  Pcbp2  Ubxn2a  Bag6  Trim3  Ddb1  Derl1  Atxn3  Ppp2r5c | web link |
| negative regulation of mRNA splicing, via spliceosome | Gene Ontology | GO:0048025 | 7 (0.95%) | 22 (0.10%) | 6.75514393611e-06 | 0.000502132365918 | Hnrnpk  Sfswap  Hnrnpa2b1  Ptbp3  Hnrnpl  Akr1c6  Npm1 | web link |
| lipid metabolic process | Gene Ontology | GO:0006629 | 39 (5.31%) | 544 (2.60%) | 2.07032762631e-05 | 0.000994389466223 | Sult1b1  Cers2  Acox2  Mgll  Acox1  Baat  Sc5d  St3gal4  Angptl4  Cyp2c40  Gm44805  Mtmr2  Tm7sf2  Cbr4  4932438A13Rik  Elovl5  Pnpla6  Ptdss1  Gba  Arv1  Acacb  Hdlbp  Gstm4  Insig2  Hsd17b2  Akr1c6  Hsd17b6  Lipc  Pnpla8  Cyb5r3  Crot  Pcyt2  Enpp2  Pnpla2  Srebf1  Lpcat3  Gba2  Nr1h3  Gpcpd1 | web link |
| regulation of RNA splicing | Gene Ontology | GO:0043484 | 9 (1.23%) | 46 (0.22%) | 2.59659351007e-05 | 0.000994389466223 | Son  Esrp2  Hnrnpl  Hnrnph3  Ptbp3  Ptbp2  Snrnp70  Clk4  Hnrnpll | web link |
| protein transport | Gene Ontology | GO:0015031 | 43 (5.86%) | 631 (3.01%) | 2.67548735307e-05 | 0.000994389466223 | Tomm5  Ktn1  Nup98  Dop1b  Synrg  Ccdc91  Entr1  Mon2  Trim3  Derl1  Copa  Snap23  Rhbdf2  Yif1b  Sec24c  Sec24b  Chmp3  Unc50  Lman2l  Washc3  Ubap1  Exoc7  Sec23b  Atg16l1  Atg16l2  Atg4d  Akap8  Bet1  Mcfd2  Tbc1d17  Sec61a2  Timm17b  Fam160a2  Rab4b  Snx10  Dennd10  Myo1c  Arrb2  Tomm22  Eny2  Golph3  Sec31b  Eif5a | web link |
| steroid metabolic process | Gene Ontology | GO:0008202 | 14 (1.91%) | 112 (0.53%) | 3.7073489678e-05 | 0.00118105545688 | Sult1b1  Cyb5r3  Tm7sf2  Gba  Cyp3a59  Akr1c6  Hdlbp  Sc5d  Srebf1  Insig2  Arv1  Gba2  Hsd17b6  Akr1c20 | web link |
| mRNA processing | Gene Ontology | GO:0006397 | 28 (3.81%) | 351 (1.67%) | 4.85965042869e-05 | 0.001354627557 | Srek1  Srsf3  Esrp2  Hnrnpk  Hnrnpl  Hnrnpm  Ptbp3  Ptbp2  Pan3  Tent2  Gemin7  Ppil3  Ybx1  Srrt  Txnl4a  Hnrnpll  Thoc2l  U2af1  Rbm39  Snrpa1  Son  Akap8l  Sfswap  Hnrnpa2b1  Cpsf7  Cpsf6  Thoc7  Cpsf1 | web link |
| mitochondrion morphogenesis | Gene Ontology | GO:0070584 | 7 (0.95%) | 32 (0.15%) | 9.82391243389e-05 | 0.00243414719195 | Bcl2l1  Mff  Polg2  Mup9  Ndufs6  Opa1  Ssbp1 | web link |
| positive regulation of fatty acid biosynthetic process | Gene Ontology | GO:0045723 | 5 (0.68%) | 15 (0.07%) | 0.000116406209827 | 0.00259585847914 | Mlxipl  Gm44805  Elovl5  Srebf1  Nr1h3 | web link |
| phosphorylation | Gene Ontology | GO:0016310 | 41 (5.59%) | 641 (3.06%) | 0.000163478150393 | 0.00307302703357 | Pfkfb2  Uckl1  Snrk  Stk11  Glyctk  Csnk1g2  Csnk1g3  Mapkapk5  Irak1  Adk  Nme6  Rps6ka3  Csnk1a1  Pi4ka  Pnkp  Pip5k1c  Mapkapk2  Mapk15  Clk4  Acvr2b  Ulk1  Akap8  Uhmk1  Map4k2  Mapk6  Fggy  Idnk  Gne  Abl1  Xylb  Mapk9  Papss2  Sgk1  Fgfr2  Tec  Dcakd  Rps6kb2  Akap8l  Mast3  Cdk8  Gk | web link |
| nucleic acid phosphodiester bond hydrolysis | Gene Ontology | GO:0090305 | 14 (1.91%) | 129 (0.62%) | 0.000174362096744 | 0.00307302703357 | Exosc10  Dna2  Rnaseh2a  Rnaset2b  Enpp2  Ercc5  Dclre1c  Apex1  Rexo5  Gm49673  Aen  Alkbh2  Alkbh3  Enpp3 | web link |
| triglyceride homeostasis | Gene Ontology | GO:0070328 | 7 (0.95%) | 35 (0.17%) | 0.000179145073706 | 0.00307302703357 | Lipc  Gckr  Rora  Angptl4  Gm44805  Pnpla8  Nr1h3 | web link |
| chaperone-mediated protein folding | Gene Ontology | GO:0061077 | 6 (0.82%) | 26 (0.12%) | 0.000228177835863 | 0.00363454695696 | Dnajc5  Unc45a  Dffa  Dnajb6  Fkbp4  Clu | web link |
| lipid homeostasis | Gene Ontology | GO:0055088 | 8 (1.09%) | 49 (0.23%) | 0.000275223722295 | 0.00396854581706 | Nr1h3  Acox2  Acox1  Usf2  Pnpla2  Pnpla8  Thada  Cln8 | web link |
| cellular response to DNA damage stimulus | Gene Ontology | GO:0006974 | 32 (4.36%) | 470 (2.24%) | 0.000284738713332 | 0.00396854581706 | Stk11  Wac  Ube2d3  Apex1  Inip  Hdac10  Mapkapk2  Yap1  Sgk1  Usp47  Huwe1  Ercc5  Mapk15  Dclre1c  Atf2  Emsy  Hmces  Chd2  Cenpx  Abl1  Shld2  Aen  Dna2  Parp3  Xiap  Ddb1  Fbxo6  Ddb2  Mrnip  Alkbh2  Alkbh3  Eya3 | web link |
| iron ion homeostasis | Gene Ontology | GO:0055072 | 7 (0.95%) | 39 (0.19%) | 0.00036288407809 | 0.00476018525965 | Gm16867  Slc40a1  Tmprss6  Trf  Fxn  Cp  Bdh2 | web link |
| negative regulation of release of cytochrome c from mitochondria | Gene Ontology | GO:0090201 | 5 (0.68%) | 21 (0.10%) | 0.000662411963754 | 0.00819241553257 | Arrb2  Lmna  Fxn  Higd1a  Opa1 | web link |
| DNA repair | Gene Ontology | GO:0006281 | 25 (3.41%) | 352 (1.68%) | 0.00069800849829 | 0.00819241553257 | Ercc5  Apex1  Inip  Hdac10  Npm1  Pnkp  Kmt5b  Usp47  Huwe1  Ube2d3  Dclre1c  Emsy  Cenpx  Abl1  Shld2  Dna2  Polg2  Parp3  Ddb1  Fbxo6  Ddb2  Mrnip  Alkbh2  Alkbh3  Eya3 | web link |
| ubiquitin-dependent ERAD pathway | Gene Ontology | GO:0030433 | 9 (1.23%) | 71 (0.34%) | 0.000806102557008 | 0.00898804351064 | Bag6  Dnajb12  Man1a  Derl1  Derl2  Ube2g2  Fbxo6  Ubxn4  Psmc6 | web link |
| protein ubiquitination | Gene Ontology | GO:0016567 | 34 (4.63%) | 545 (2.60%) | 0.000890516562031 | 0.00945643777776 | Ube2i  Rnf114  Znrf1  Rspry1  Fbxl12  Ankib1  Ddb2  Trim3  Anapc5  Fbxw5  Trim7  Trim11  Rnf167  Nedd4l  Huwe1  Ube2d3  Uba52  Ubc  Rnf214  Ubox5  Btrc  Dcaf11  Cdk8  Med7  Smurf1  Anapc10  Pcnp  Xiap  Arrb2  Wsb1  Fbxo6  Rnf220  Ddb1  Ube2g2 | web link |
| positive regulation of epithelial cell proliferation | Gene Ontology | GO:0050679 | 9 (1.23%) | 73 (0.35%) | 0.000987718261482 | 0.0100118714687 | Esr1  Hyal1  Azin1  Tcf7l2  Esrp2  Vegfa  Pkhd1  Fgf1  Yap1 | web link |
| metabolic process | Gene Ontology | GO:0008152 | 16 (2.18%) | 190 (0.91%) | 0.00109718093711 | 0.0106378847381 | Dna2  Man1a  Man2c1  Spast  Gba  Ctbs  Hyal1  Pfkfb2  Enpp3  Papss2  Ganc  Acacb  Fbp1  Gne  Gba2  Pam | web link |
| regulation of growth | Gene Ontology | GO:0040008 | 9 (1.23%) | 77 (0.37%) | 0.00145035054251 | 0.0134575837891 | Bcl2l1  Enox2  Selenop  Safb  Epc1  Vegfa  Igfbp4  Igfbp5  Brd8 | web link |
| carbohydrate phosphorylation | Gene Ontology | GO:0046835 | 5 (0.68%) | 25 (0.12%) | 0.00154027563501 | 0.0134575837891 | Pfkfb2  Xylb  Fggy  Khk  Gne | web link |
| response to cytokine | Gene Ontology | GO:0034097 | 9 (1.23%) | 78 (0.37%) | 0.00158965794901 | 0.0134575837891 | Bcl2l1  Acp5  Serpina3m  Ghr  Itih4  Tyms  Slco1b2  Lifr  Mapkapk2 | web link |
| regulation of cell growth | Gene Ontology | GO:0001558 | 7 (0.95%) | 50 (0.24%) | 0.00168974146231 | 0.0134575837891 | Stk11  4932438A13Rik  Epb41  Igfbp4  Igfbp5  Npm1  Lamtor2 | web link |
| response to ionizing radiation | Gene Ontology | GO:0010212 | 7 (0.95%) | 50 (0.24%) | 0.00168974146231 | 0.0134575837891 | Cyp2r1  Stk11  Inip  Mrnip  Dclre1c  Eya3  Aen | web link |
| mRNA export from nucleus | Gene Ontology | GO:0006406 | 6 (0.82%) | 38 (0.18%) | 0.00191521881591 | 0.014236459865 | Srsf3  Hnrnpa2b1  Eny2  Thoc7  Thoc2l  Eif5a | web link |
| protein targeting | Gene Ontology | GO:0006605 | 6 (0.82%) | 38 (0.18%) | 0.00191521881591 | 0.014236459865 | Pan3  Ywhaz  Gm20498  Erbin  Trak1  Myo1c | web link |
| cholesterol metabolic process | Gene Ontology | GO:0008203 | 10 (1.36%) | 98 (0.47%) | 0.00227971616583 | 0.0159617982867 | Lipc  Cyb5r3  Tm7sf2  Gba  Hdlbp  Srebf1  Insig2  Gba2  Arv1  Cln8 | web link |
| positive regulation of transcription by RNA polymerase II | Gene Ontology | GO:0045944 | 61 (8.31%) | 1201 (5.73%) | 0.00229048226536 | 0.0159617982867 | Sbno2  Hsf2  Hsf4  Abhd14b  Apex1  Hnrnpk  Zfp639  Tmprss6  Mdm4  Rfxap  Yap1  Dcn  Rps6ka3  Foxk2  Esr1  Mlxipl  Npm1  Safb  Churc1  Epc1  Usf2  Ss18  Tnip1  Gzf1  Tfdp1  Gtf2a2  Smarca2  Ybx1  Bcas3  Mta2  Cela1  Slc40a1  Stat1  Hax1  Zbtb18  Brd8  Tcf7l2  Tbx3  Per1  Pcbp2  Rora  Vegfa  Ablim2  Zmiz2  Gtf2i  Fgf1  Crem  Tbl1xr1  Zfp780b  Aamdc  Zfpm1  Tef  Mettl23  Akap8l  Srebf1  Camta2  Gmeb2  Atf2  Nr1h3  Zfp62  Vezf1 | web link |
| translation | Gene Ontology | GO:0006412 | 21 (2.86%) | 303 (1.45%) | 0.00242418695491 | 0.0159919372764 | Mrpl36  Rpl22l1  Rbm3  Mrpl30  Mrpl24  Uba52  Eef1d  Aars  Eif5  Cpeb3  Eif3e  Rpl15  Farsa  Mtif3  Kxd1  Mrpl47  Rps19  Eif5a  Rpl18  Mettl17  Rpl35a | web link |
| positive regulation of transcription, DNA-templated | Gene Ontology | GO:0045893 | 39 (5.31%) | 691 (3.30%) | 0.00244428371382 | 0.0159919372764 | Actn1  Nup98  Edrf1  Asph  Npm1  Kdm5a  Tfdp1  Yap1  Banp  Pmf1  Esr1  Mlxipl  Churc1  Usf2  Ss18  Rfxap  Nsd1  Smarca2  Rbm39  Nr1h3  Stat1  Wac  Btrc  Tcf7l2  Trf  Tbx3  Rora  Ccar1  Mapk9  Atxn7l3  Fgf1  Foxk2  Tbl1xr1  Nolc1  Hmbox1  Rai1  Srebf1  Eny2  Arap1 | web link |
| negative regulation of extrinsic apoptotic signaling pathway | Gene Ontology | GO:2001237 | 6 (0.82%) | 40 (0.19%) | 0.00250994531245 | 0.0159919372764 | Cttn  Tcf7l2  Cflar  Itgav  Lmna  Yap1 | web link |
| negative regulation of fat cell differentiation | Gene Ontology | GO:0045599 | 7 (0.95%) | 54 (0.26%) | 0.00265610973375 | 0.016453124184 | Lrp3  Zfpm1  Rora  Vegfa  Tcf7l2  Ncor2  Yap1 | web link |
| autophagy | Gene Ontology | GO:0006914 | 14 (1.91%) | 172 (0.82%) | 0.00299295224016 | 0.0179690471922 | Dram2  Gba  Atg16l1  Atg4d  Stk11  Retreg1  Tmem208  Ulk1  S100a9  Tmbim6  Abl1  Hdac10  Tm9sf1  Tbc1d17 | web link |
| endoplasmic reticulum to Golgi vesicle-mediated transport | Gene Ontology | GO:0006888 | 10 (1.36%) | 102 (0.49%) | 0.0030619901045 | 0.0179690471922 | Trappc8  Bet1  Copa  Spast  Sec23b  Yif1b  Sec24c  Sec24b  Lman2l  Sec31b | web link |
| negative regulation of transcription by RNA polymerase II | Gene Ontology | GO:0000122 | 48 (6.54%) | 913 (4.36%) | 0.00341717880229 | 0.0194932863494 | Ube2i  Hmbox1  Hsf4  Pkig  Macroh2a1  Zbtb42  Hnrnpk  Vegfa  Fbp1  Cnot2  Hdac10  Mdm4  Kdm5a  Yap1  Foxk2  Esr1  Mlxipl  Mier1  Tsc22d3  Tmprss6  Epc1  Mapk15  N4bp2l2  Gzf1  Mbd3  Klf3  Ybx1  Mta2  Cela1  Mier2  Acvr2b  Zbtb14  Zbtb18  Tcf7l2  Tbx3  Per1  Sap130  Uxt  Tbl1xr1  Zfpm1  Cpeb3  Srebf1  Hnrnpa2b1  Atf2  Nr1h3  Ncor2  Ncor1  Crem | web link |
| RNA splicing | Gene Ontology | GO:0008380 | 19 (2.59%) | 272 (1.30%) | 0.00349655360527 | 0.0194932863494 | Sfswap  Srek1  Ybx1  Ppil3  Srsf3  Gemin7  U2af1  Rbm39  Snrpa1  Son  Esrp2  Hnrnpk  Srsf11  Hnrnpa2b1  Ptbp2  Ptbp3  Txnl4a  Thoc7  Hnrnpm | web link |
| in utero embryonic development | Gene Ontology | GO:0001701 | 20 (2.72%) | 293 (1.40%) | 0.00360680773278 | 0.0196175152295 | Bcl2l1  Zbtb18  Tctn1  Mbd3  Tbx3  Prrc2b  Polg2  Kidins220  Tcf7l2  H13  Mgat1  N4bp2l2  Vegfa  Gna13  Sec24c  Smpd4  Hsd17b2  Ybx1  Ncor2  Dusp3 | web link |
| cell cycle | Gene Ontology | GO:0007049 | 34 (4.63%) | 595 (2.84%) | 0.00371744954638 | 0.0197378868772 | Ube2i  Pard3  Ppp2r2d  Stk11  Entr1  Cenpv  Nde1  Cdc123  Anapc5  Tfdp1  Rps6ka3  Banp  Pmf1  Ccnh  Pard6a  Chmp3  Inca1  Ncapd3  Septin2  Mapk6  Cenpx  Septin9  Epb41  Csnk1a1  Rcc1  Zfyve19  Spast  Anapc15  Rabgap1  Anapc10  Son  Pcnp  Ccar1  Txnl4a | web link |
| regulation of apoptotic process | Gene Ontology | GO:0042981 | 15 (2.04%) | 196 (0.94%) | 0.0038894794245 | 0.0201710212015 | Bcl2l1  Madd  Card19  Bag6  Esr1  Rassf5  Dffa  Apex1  Cflar  Akt1s1  Anp32a  Abl1  Clu  Dedd  Gls2 | web link |
| cellular response to hypoxia | Gene Ontology | GO:0071456 | 11 (1.50%) | 123 (0.59%) | 0.00398001057039 | 0.020171417209 | Cflar  Npm1  Higd1a  Rora  Cbs  Vegfa  Gngt1  Opa1  Lmna  Irak1  Mdm4 | web link |
| positive regulation of telomere maintenance via telomerase | Gene Ontology | GO:0032212 | 5 (0.68%) | 31 (0.15%) | 0.00414331676599 | 0.0205324364181 | Mapk15  Mapkapk5  Hmbox1  Tcp1  Pnkp | web link |
| lactation | Gene Ontology | GO:0007595 | 6 (0.82%) | 45 (0.21%) | 0.00459587021861 | 0.022279979538 | Prlr  Pam  Usf2  Aprt  Vegfa  Ncor2 | web link |
| positive regulation of dendritic spine development | Gene Ontology | GO:0060999 | 5 (0.68%) | 32 (0.15%) | 0.00477150124114 | 0.0224755428135 | Mapk6  Mapkapk5  Hnrnpk  Zmynd8  Cpeb3 | web link |
| skeletal muscle tissue development | Gene Ontology | GO:0007519 | 7 (0.95%) | 60 (0.29%) | 0.00483778500021 | 0.0224755428135 | Dcn  Zbtb18  Taz  Myl6  Cflar  Mylpf  Gpcpd1 | web link |
| cell division | Gene Ontology | GO:0051301 | 23 (3.13%) | 365 (1.74%) | 0.00505606202672 | 0.0226547827577 | Ube2i  Pard3  Ppp2r2d  Entr1  Txnl4a  Nde1  Cdc123  Anapc5  Csnk1a1  Pmf1  Pard6a  Chmp3  Cenpv  Epb41  Septin2  Cenpx  Septin9  Ncapd3  Rcc1  Zfyve19  Spast  Anapc15  Anapc10 | web link |
| negative regulation of I-kappaB kinase/NF-kappaB signaling | Gene Ontology | GO:0043124 | 6 (0.82%) | 46 (0.22%) | 0.00513200385818 | 0.0226547827577 | Stat1  Esr1  Rora  Per1  Tnip1  Abl1 | web link |
| protein polyubiquitination | Gene Ontology | GO:0000209 | 13 (1.77%) | 164 (0.78%) | 0.00518113865759 | 0.0226547827577 | Ubox5  Smurf1  Huwe1  Rnf167  Nedd4l  Btrc  Ube2d3  Ddb2  Rnf114  Ube2g2  Ankib1  Trim3  Ube2v2 | web link |
| negative regulation of cysteine-type endopeptidase activity involved in apoptotic process | Gene Ontology | GO:0043154 | 8 (1.09%) | 77 (0.37%) | 0.00545764513958 | 0.0229918030175 | Bcl2l1  Rps6ka3  Usp47  Dnajb6  Cflar  Xiap  Vegfa  Arrb2 | web link |
| positive regulation of telomerase activity | Gene Ontology | GO:0051973 | 5 (0.68%) | 33 (0.16%) | 0.00546441955125 | 0.0229918030175 | Mapk15  Tcp1  Hmbox1  Mapkapk5  Pnkp | web link |
| fatty acid beta-oxidation | Gene Ontology | GO:0006635 | 6 (0.82%) | 47 (0.22%) | 0.00571258235532 | 0.0235908493562 | Acox2  Crot  Acox1  Bdh2  Abcd4  Echdc1 | web link |
| peptidyl-tyrosine dephosphorylation | Gene Ontology | GO:0035335 | 9 (1.23%) | 95 (0.45%) | 0.0060709679018 | 0.0241754614661 | Mtmr14  Acp1  Eya3  Mtmr4  Dusp16  Dusp11  Mtmr2  Dusp3  Dusp12 | web link |
| Golgi organization | Gene Ontology | GO:0007030 | 9 (1.23%) | 95 (0.45%) | 0.0060709679018 | 0.0241754614661 | Lysmd3  Trappc8  Huwe1  Csnk1a1  Golph3  Gak  Ubxn2a  Lman2l  Bcas3 | web link |
| negative regulation of smooth muscle cell proliferation | Gene Ontology | GO:0048662 | 5 (0.68%) | 35 (0.17%) | 0.00705789860679 | 0.0276124805143 | Ndrg2  Igfbp5  Comt  Ralbp1  Esr1 | web link |
| negative regulation of transcription, DNA-templated | Gene Ontology | GO:0045892 | 34 (4.63%) | 622 (2.97%) | 0.00722761771311 | 0.0277889439659 | Ube2i  Pphln1  Hsf4  Sbno2  Zfp639  Hdac10  Irak1  Trim11  Foxk2  Hmbox1  Mlxipl  Usp47  Dnajb6  Tmprss6  Epc1  Mup9  Gzf1  Smarca2  Riox2  Mta2  Nr1h3  Dhrs7b  Btrc  Zmynd8  Zbtb14  Mterf3  Zbtb18  Tcf7l2  Tbx3  Per1  Tbl1xr1  Ccar1  Ncor2  Ncor1 | web link |
| adipose tissue development | Gene Ontology | GO:0060612 | 5 (0.68%) | 36 (0.17%) | 0.00796502159508 | 0.0301050816221 | Dhrs7b  Atf2  4932438A13Rik  Tbl1xr1  Slc25a25 | web link |
| positive regulation of cell growth | Gene Ontology | GO:0030307 | 8 (1.09%) | 84 (0.40%) | 0.00914758833903 | 0.0336431898417 | Sgk1  Hdgfl2  Hyal1  Cep43  Derl2  Fxn  Zfp639  Usp47 | web link |
| glucose homeostasis | Gene Ontology | GO:0042593 | 11 (1.50%) | 138 (0.66%) | 0.00931259620549 | 0.0336431898417 | Slc8b1  Mlxipl  Stk11  Ero1b  Gckr  Tcf7l2  Mup9  Rbp4  Igfbp5  Gk  Ncor2 | web link |
| Rho protein signal transduction | Gene Ontology | GO:0007266 | 6 (0.82%) | 52 (0.25%) | 0.00935371197394 | 0.0336431898417 | Arhgef12  Gm20498  Cdc42ep4  Arhgap32  Arhgdib  Gna13 | web link |
| histone H4 acetylation | Gene Ontology | GO:0043967 | 5 (0.68%) | 38 (0.18%) | 0.0100158547355 | 0.0354529461271 | Atf2  Per1  Naa60  Brd8  Epc1 | web link |
| negative regulation of apoptotic process | Gene Ontology | GO:0043066 | 31 (4.22%) | 570 (2.72%) | 0.0107137736736 | 0.0367273815699 | Fn1  Opa1  Clu  Hnrnpk  Vegfa  Tmbim6  Pkhd1  Proc  Bcl2l1  Sgk1  Cbs  Npm1  Usp47  Golph3  Higd1a  Ybx1  Bag6  Slc40a1  Eif5a  Prlr  Hax1  Tbx3  Fxn  Aamdc  Son  Cflar  Xiap  Angptl4  Mdm4  Ddb1  Cln8 | web link |
| rhythmic process | Gene Ontology | GO:0048511 | 11 (1.50%) | 141 (0.67%) | 0.0108457079714 | 0.0367273815699 | Mapk9  Huwe1  Tef  Btrc  Rora  Rai1  Per1  Enox2  Crem  Ddb1  Kdm5a | web link |
| histone deacetylation | Gene Ontology | GO:0016575 | 5 (0.68%) | 39 (0.19%) | 0.0111656184472 | 0.0367273815699 | Mier1  Mier2  Tbl1xr1  Mta2  Hdac10 | web link |
| outflow tract morphogenesis | Gene Ontology | GO:0003151 | 6 (0.82%) | 54 (0.26%) | 0.0111921133091 | 0.0367273815699 | Zfpm1  Dhrs3  Tbx3  Vegfa  Sec24b  Atf2 | web link |
| regulation of protein stability | Gene Ontology | GO:0031647 | 8 (1.09%) | 87 (0.42%) | 0.0111993809271 | 0.0367273815699 | Ctsa  Usp47  Asph  Usp40  Nedd4l  Srebf1  Lmna  Npm1 | web link |
| positive regulation of protein ubiquitination | Gene Ontology | GO:0031398 | 7 (0.95%) | 72 (0.34%) | 0.0128998327092 | 0.0416907636834 | Derl1  Huwe1  Rassf5  Mapk15  Xiap  Mapk9  Npm1 | web link |
| lipid catabolic process | Gene Ontology | GO:0016042 | 10 (1.36%) | 127 (0.61%) | 0.0138892435072 | 0.0442471614586 | Lipc  Tbl1xr1  Ces3a  Enpp2  Mgll  Pnpla2  Pnpla6  Gm44805  Pnpla8  Ces1f | web link |
| protein dephosphorylation | Gene Ontology | GO:0006470 | 12 (1.63%) | 169 (0.81%) | 0.0160504319383 | 0.0504119200315 | Dusp12  Dusp16  Acp1  Ppp5c  Btrc  Stk11  Dusp3  Mtmr4  Dusp11  Ppp4r3a  Eya3  Ppp2r5c | web link |
| activation of GTPase activity | Gene Ontology | GO:0090630 | 8 (1.09%) | 93 (0.44%) | 0.0162926309595 | 0.0504618986661 | Arhgef19  Eif5  Bcas3  Tiam2  Ndel1  Grtp1  Tbc1d17  Rabgap1 | web link |
| response to lipopolysaccharide | Gene Ontology | GO:0032496 | 13 (1.77%) | 191 (0.91%) | 0.0172712087846 | 0.0527599939586 | Dcn  Acp5  Rps6ka3  Mgst1  Erbin  Comt  Lgals9  Slco1b2  S100a9  Aldoa  Irak1  Fgfr2  Mapkapk2 | web link |
| protein glycosylation | Gene Ontology | GO:0006486 | 12 (1.63%) | 172 (0.82%) | 0.0181874678737 | 0.0548081802139 | Entpd5  Dpagt1  St3gal5  Tmem258  Extl2  St6gal1  Nagpa  Mgat1  St3gal4  Man1a  Pomgnt2  Uggt1 | web link |
| regulation of circadian rhythm | Gene Ontology | GO:0042752 | 6 (0.82%) | 61 (0.29%) | 0.019622575313 | 0.057880728617 | Ddb1  Btrc  Rora  Per1  Crem  Mapk9 | web link |
| protein autophosphorylation | Gene Ontology | GO:0046777 | 12 (1.63%) | 174 (0.83%) | 0.0197261676004 | 0.057880728617 | Ulk1  Csnk1g3  Clk4  Stk11  Uhmk1  Csnk1g2  Mapk15  Abl1  Mapkapk5  Irak1  Fgfr2  Mapkapk2 | web link |
| dephosphorylation | Gene Ontology | GO:0016311 | 12 (1.63%) | 178 (0.85%) | 0.023091741023 | 0.0652553820888 | Acp5  Mtmr14  Pnkp  Dusp16  Pfkfb2  Inpp4a  Fbp1  Mtmr4  Dusp11  Mtmr2  Dusp3  Dusp12 | web link |
| response to unfolded protein | Gene Ontology | GO:0006986 | 5 (0.68%) | 47 (0.22%) | 0.0237026275749 | 0.0652553820888 | Tmbim6  Derl2  Derl1  Fbxo6  Upf3b | web link |
| protein N-linked glycosylation | Gene Ontology | GO:0006487 | 5 (0.68%) | 47 (0.22%) | 0.0237026275749 | 0.0652553820888 | Slc39a8  Mgat1  Entpd5  Dpagt1  Tmem258 | web link |
| retinol metabolic process | Gene Ontology | GO:0042572 | 5 (0.68%) | 47 (0.22%) | 0.0237026275749 | 0.0652553820888 | Pnpla2  Rdh13  Rbp4  Dhrs3  Ces1f | web link |
| SCF-dependent proteasomal ubiquitin-dependent protein catabolic process | Gene Ontology | GO:0031146 | 5 (0.68%) | 47 (0.22%) | 0.0237026275749 | 0.0652553820888 | Fbxl12  Amn1  Btrc  Fbxw5  Fbxo6 | web link |
| positive regulation of translation | Gene Ontology | GO:0045727 | 7 (0.95%) | 82 (0.39%) | 0.024760514287 | 0.0673365205609 | Plxnb2  Rbm3  Eif3e  Cpeb3  Upf3b  Hnrnpl  Npm1 | web link |
| arachidonic acid metabolic process | Gene Ontology | GO:0019369 | 5 (0.68%) | 48 (0.23%) | 0.0257215770643 | 0.069107369703 | Cyp4a31  Pnpla8  Mgll  Cyp2d10  Cyp2c40 | web link |
| positive regulation of cell population proliferation | Gene Ontology | GO:0008284 | 30 (4.09%) | 590 (2.81%) | 0.0271153261609 | 0.0719847349272 | Hsf4  Fn1  Thpo  Derl2  Fxn  Vegfa  Tial1  Pkhd1  Clu  Mdm4  Yap1  Bcl2l1  Mlxipl  Npm1  Itgav  Mapk15  Lifr  Tns3  Ybx1  Prlr  Cep43  Tbx3  Eapp  Fgf1  Fgfr2  Cd47  Enpp2  Ccar1  Nolc1  Eif5a | web link |
| electron transport chain | Gene Ontology | GO:0022900 | 6 (0.82%) | 66 (0.31%) | 0.0277777460388 | 0.0720283414728 | Enox2  Uqcr11  Ndufs2  Aox3  Cox7a2  Etfbl | web link |
| steroid biosynthetic process | Gene Ontology | GO:0006694 | 6 (0.82%) | 66 (0.31%) | 0.0277777460388 | 0.0720283414728 | Cyb5r3  Tm7sf2  Sc5d  Hsd17b2  Akr1c6  Akr1c20 | web link |
| endocytosis | Gene Ontology | GO:0006897 | 14 (1.91%) | 226 (1.08%) | 0.0284664128407 | 0.0725630236675 | Cttn  Ralbp1  Gm20498  Snx10  Pip5k1c  Synrg  Enpp2  Srl  Ghr  Csnk1g2  Csnk1g3  Arrb2  Abl1  Bin1 | web link |
| peptidyl-serine phosphorylation | Gene Ontology | GO:0018105 | 11 (1.50%) | 163 (0.78%) | 0.0286347357971 | 0.0725630236675 | Rps6ka3  Csnk1a1  Sgk1  Uhmk1  Rps6kb2  Csnk1g2  Csnk1g3  Mast3  Mapkapk5  Mapk9  Mapkapk2 | web link |
| RNA processing | Gene Ontology | GO:0006396 | 6 (0.82%) | 67 (0.32%) | 0.0296407363924 | 0.0742683619719 | Exosc10  Prpf39  Rtca  Rcl1  Hnrnpk  Sfswap | web link |
| regulation of cell cycle | Gene Ontology | GO:0051726 | 10 (1.36%) | 145 (0.69%) | 0.0315679793856 | 0.0782184378109 | Madd  Sgk1  Btrc  Son  Abl1  Xiap  Fbxl12  Cdk8  Npm1  Tfdp1 | web link |
| wound healing | Gene Ontology | GO:0042060 | 8 (1.09%) | 106 (0.51%) | 0.032693064404 | 0.0801159710119 | Dcn  Fn1  Arhgef19  Coro1b  Cflar  Fgfr2  Fgf1  Yap1 | web link |
| chromatin organization | Gene Ontology | GO:0006325 | 17 (2.32%) | 298 (1.42%) | 0.0340064827868 | 0.0822400982167 | Emsy  Chd2  Bag6  Banp  Macroh2a1  Kmt5b  Tbl1xr1  Wac  Epc1  Eny2  Brd8  Naa60  Nsd1  Atxn7l3  Kdm5a  Eya3  Brd3 | web link |
| positive regulation of peptidyl-tyrosine phosphorylation | Gene Ontology | GO:0050731 | 8 (1.09%) | 107 (0.51%) | 0.0342974400635 | 0.0822400982167 | Ghr  Enpp2  Tec  Hax1  Arrb2  Vegfa  Abl1  Gfra1 | web link |
| ubiquitin-dependent protein catabolic process | Gene Ontology | GO:0006511 | 15 (2.04%) | 256 (1.22%) | 0.0365729844092 | 0.0867635693963 | Smurf1  Kctd21  Bag6  Usp47  Btrc  Usp40  Ube2d3  Nedd4l  Rnf114  Fbxl12  Ube2g2  Ankib1  Rnf167  Ddb1  Atxn3 | web link |
| fatty acid metabolic process | Gene Ontology | GO:0006631 | 12 (1.63%) | 193 (0.92%) | 0.0395157185028 | 0.0914225492492 | Lipc  Acacb  Acox2  Cbr4  Crot  Acox1  Mgll  Elovl5  Crem  Ndufs6  Pnpla8  Baat | web link |
| carbohydrate metabolic process | Gene Ontology | GO:0005975 | 13 (1.77%) | 215 (1.03%) | 0.0399681493478 | 0.0914225492492 | Man2c1  Ctbs  Hyal1  Man1a  Gckr  Phkb  Ganc  Fggy  Idnk  Gba2  Xylb  Gk  Fbp1 | web link |
| negative regulation of BMP signaling pathway | Gene Ontology | GO:0030514 | 5 (0.68%) | 54 (0.26%) | 0.0401243407631 | 0.0914225492492 | Smurf1  Abl1  Chrd  Tmprss6  Tcf7l2 | web link |
| positive regulation of osteoblast differentiation | Gene Ontology | GO:0045669 | 6 (0.82%) | 72 (0.34%) | 0.040176725679 | 0.0914225492492 | Lrp3  Acvr2b  Atraid  Vegfa  Lmna  Yap1 | web link |
| positive regulation of protein catabolic process | Gene Ontology | GO:0045732 | 6 (0.82%) | 73 (0.35%) | 0.0425343020332 | 0.094851493534 | Smurf1  Nedd4l  Huwe1  Mapk15  Rhbdd3  Ddb1 | web link |
| xenobiotic metabolic process | Gene Ontology | GO:0006805 | 6 (0.82%) | 73 (0.35%) | 0.0425343020332 | 0.094851493534 | Sult1b1  Cyp2d10  Rora  Gsta2  Cyp2c40  Cyp2r1 | web link |
| fatty acid biosynthetic process | Gene Ontology | GO:0006633 | 6 (0.82%) | 74 (0.35%) | 0.0449770203185 | 0.0983321130492 | Lipc  Acacb  Cbr4  Mgll  Elovl5  Nr1h3 | web link |
| roof of mouth development | Gene Ontology | GO:0060021 | 6 (0.82%) | 74 (0.35%) | 0.0449770203185 | 0.0983321130492 | Acvr2b  Asph  Dhrs3  Tbx3  Insig2  Plekha1 | web link |
| amino acid transport | Gene Ontology | GO:0006865 | 5 (0.68%) | 56 (0.27%) | 0.0458230206561 | 0.0992090641389 | Slc38a10  Slc25a44  Slc43a1  Slc38a3  Slc38a4 | web link |
| mRNA transport | Gene Ontology | GO:0051028 | 7 (0.95%) | 94 (0.45%) | 0.0468684162363 | 0.100496700199 | Srsf3  Nup98  Myo1c  Hnrnpa2b1  Eny2  Thoc7  Eif5a | web link |
| mitochondrial respiratory chain complex I assembly | Gene Ontology | GO:0032981 | 5 (0.68%) | 58 (0.28%) | 0.0519792238796 | 0.109352518162 | Ndufs2  Ndufa5  Ndufb2  Taz  Ndufs6 | web link |
| muscle organ development | Gene Ontology | GO:0007517 | 5 (0.68%) | 58 (0.28%) | 0.0519792238796 | 0.109352518162 | Kmt5b  Zbtb42  Chd2  Unc45a  Lmna | web link |
| cell population proliferation | Gene Ontology | GO:0008283 | 10 (1.36%) | 159 (0.76%) | 0.0533859843515 | 0.111262378602 | Bcl2l1  Dazap1  Alkbh3  Tacc2  Thpo  Vegfa  Prkar1a  Naa60  Riox2  Yap1 | web link |
| circadian regulation of gene expression | Gene Ontology | GO:0032922 | 5 (0.68%) | 60 (0.29%) | 0.0585945023835 | 0.117617588274 | Rai1  Per1  Huwe1  Kdm5a  Rora | web link |
| regulation of autophagy | Gene Ontology | GO:0010506 | 5 (0.68%) | 60 (0.29%) | 0.0585945023835 | 0.117617588274 | Mapk15  Mtmr4  Dram2  Iigp1  Rragc | web link |
| ribosome biogenesis | Gene Ontology | GO:0042254 | 7 (0.95%) | 99 (0.47%) | 0.0589162521971 | 0.117617588274 | Mrpl36  Rrp36  Sbds  Mterf3  Rcl1  Ddx56  Riox2 | web link |
| methylation | Gene Ontology | GO:0032259 | 11 (1.50%) | 184 (0.88%) | 0.0594757864207 | 0.117617588274 | Vcpkmt  Kmt5b  Comt  Setd4  Mettl23  Tyms  Trmt5  Nsd1  Hemk1  Mettl17  Icmt | web link |
| microtubule cytoskeleton organization | Gene Ontology | GO:0000226 | 9 (1.23%) | 141 (0.67%) | 0.0600310605401 | 0.117617588274 | Uxt  Pard3  Camsap2  Tacc2  Son  Ss18  Ndel1  Phldb2  Atxn3 | web link |
| actin filament organization | Gene Ontology | GO:0007015 | 9 (1.23%) | 141 (0.67%) | 0.0600310605401 | 0.117617588274 | Actn1  Myo1c  Trf  Coro1b  Mprip  Arhgap12  Rhobtb1  Sorbs2  Elmo3 | web link |
| sodium ion transport | Gene Ontology | GO:0006814 | 8 (1.09%) | 120 (0.57%) | 0.0601273769653 | 0.117617588274 | Slc38a4  Slc8b1  Slc38a3  Fxyd1  Asic5  Slc9a8  Slc17a2  Slc38a10 | web link |
| glutathione metabolic process | Gene Ontology | GO:0006749 | 5 (0.68%) | 61 (0.29%) | 0.0620741387291 | 0.120369851622 | Ggt6  Idh1  Mgst1  Gstm4  Gsta2 | web link |
| response to glucose | Gene Ontology | GO:0009749 | 6 (0.82%) | 81 (0.39%) | 0.0644965584288 | 0.123989073531 | Acvr2b  Khk  Mlxipl  Pfkfb2  Tcf7l2  Srebf1 | web link |
| protein K48-linked ubiquitination | Gene Ontology | GO:0070936 | 5 (0.68%) | 62 (0.30%) | 0.0656680717672 | 0.124679104414 | Nedd4l  Btrc  Znrf1  Ube2g2  Ube2d3 | web link |
| protein phosphorylation | Gene Ontology | GO:0006468 | 29 (3.95%) | 616 (2.94%) | 0.0662593954204 | 0.124679104414 | Snrk  Stk11  Csnk1g2  Csnk1g3  Mapkapk5  Irak1  Mapkapk2  Pan3  Map4k2  Csnk1a1  Sgk1  Mapk15  Gak  Fgfr2  Acvr2b  Ulk1  Uhmk1  Phkb  Rps6ka3  Mapk6  Abl1  Mapk9  Ywhaz  Clk4  Tec  Rps6kb2  Mast3  Prkar1a  Cdk8 | web link |
| apoptotic process | Gene Ontology | GO:0006915 | 27 (3.68%) | 567 (2.71%) | 0.0667407780808 | 0.124679104414 | Dram2  Stk11  Dffa  Tial1  Opa1  Tmbim6  Bcl2l1  Rps6ka3  Sgk1  Ube2d3  Mindy3  Atg4d  Elmo3  Dedd  Madd  Bag6  Nisch  Rassf5  S100a9  Abl1  Dido1  Aen  Uxt  Cflar  Xiap  Ccar1  Eif5a | web link |
| intracellular signal transduction | Gene Ontology | GO:0035556 | 21 (2.86%) | 421 (2.01%) | 0.0670918947518 | 0.124679104414 | Mast3  Arhgef12  Map4k2  Mapk9  Sgk1  Gpr155  Mapk15  Snrk  Tec  Rassf5  Ss18  Tiam2  Rps6ka3  Mapk6  Wsb1  Igfbp5  Tns3  Mapkapk5  Irak1  Mapkapk2  Gna13 | web link |
| positive regulation of canonical Wnt signaling pathway | Gene Ontology | GO:0090263 | 7 (0.95%) | 103 (0.49%) | 0.0698094928317 | 0.128657164475 | Tbl1xr1  Usp47  Csnk1g2  Csnk1g3  Xiap  Rnf220  Yap1 | web link |
| protein folding | Gene Ontology | GO:0006457 | 7 (0.95%) | 106 (0.51%) | 0.0787167980747 | 0.142932930775 | Nudcd2  Nudcd3  Tcp1  Ppil3  Dnajb6  Ahsa2  Pdrg1 | web link |
| regulation of cell shape | Gene Ontology | GO:0008360 | 9 (1.23%) | 149 (0.71%) | 0.0788374461226 | 0.142932930775 | Plxnb2  Arap1  Fn1  Cdc42ep4  Vegfa  Gna13  Rhobtb1  Aldoa  Epb41 | web link |
| positive regulation of GTPase activity | Gene Ontology | GO:0043547 | 16 (2.18%) | 310 (1.48%) | 0.0798219725257 | 0.143550805429 | Arhgef12  Sec23b  Arap1  Arhgef19  Rabgap1  Rgs3  Ralbp1  Arhgap32  Pkp4  Arhgap12  Rin3  Ndel1  Arhgdib  Agap3  Bin1  Bcas3 | web link |
| cellular response to insulin stimulus | Gene Ontology | GO:0032869 | 7 (0.95%) | 107 (0.51%) | 0.0818261531355 | 0.145977857194 | Ghr  Sgk1  Cflar  Aprt  Hnrnpk  Insig2  Srebf1 | web link |
| mitochondrion organization | Gene Ontology | GO:0007005 | 6 (0.82%) | 87 (0.42%) | 0.0846226226437 | 0.149768609917 | Cluh  Tmem11  Taz  Fxn  Opa1  Vps13d | web link |
| chromosome segregation | Gene Ontology | GO:0007059 | 6 (0.82%) | 88 (0.42%) | 0.0882788992934 | 0.153798394863 | Ube2i  Rcc1  Pmf1  Ndel1  Naa60  Nde1 | web link |
| integrin-mediated signaling pathway | Gene Ontology | GO:0007229 | 6 (0.82%) | 88 (0.42%) | 0.0882788992934 | 0.153798394863 | Fn1  Tec  Itgav  Fermt3  Abl1  Zyx | web link |
| response to estrogen | Gene Ontology | GO:0043627 | 5 (0.68%) | 70 (0.33%) | 0.0984530942171 | 0.170194108608 | Aldoa  Akr1c6  Esr1  Comt  Gba | web link |
| lung development | Gene Ontology | GO:0030324 | 7 (0.95%) | 113 (0.54%) | 0.101940276621 | 0.17369080182 | Ywhaz  Acvr2b  Bag6  Srebf1  Rbp4  Vegfa  Fgf1 | web link |
| regulation of translation | Gene Ontology | GO:0006417 | 8 (1.09%) | 135 (0.64%) | 0.102033610038 | 0.17369080182 | Rbm3  Nolc1  Cnot10  Cpeb3  Tyms  S100a9  Cnot2  Mapkapk5 | web link |
| negative regulation of inflammatory response | Gene Ontology | GO:0050728 | 7 (0.95%) | 114 (0.54%) | 0.10553245065 | 0.177836571251 | Acp5  Enpp3  Rora  Lgals9  Lpcat3  Proc  Nr1h3 | web link |
| Wnt signaling pathway | Gene Ontology | GO:0016055 | 11 (1.50%) | 205 (0.98%) | 0.106764523805 | 0.177836571251 | Csnk1a1  Tmem131l  Btrc  Tcf7l2  Csnk1g2  Csnk1g3  Xiap  Gpc4  Ndrg2  Ddb1  Cela1 | web link |
| kidney development | Gene Ontology | GO:0001822 | 9 (1.23%) | 159 (0.76%) | 0.106861437434 | 0.177836571251 | Dcn  Acvr2b  Bag6  Zbtb14  Mpst  Vegfa  Ndufs6  Pkhd1  Cyp4a31 | web link |
| positive regulation of proteasomal ubiquitin-dependent protein catabolic process | Gene Ontology | GO:0032436 | 5 (0.68%) | 72 (0.34%) | 0.107731475285 | 0.177956436952 | Ankib1  Clu  Ube2v2  Csnk1a1  Gba | web link |
| post-embryonic development | Gene Ontology | GO:0009791 | 6 (0.82%) | 94 (0.45%) | 0.111978677721 | 0.1831617582 | Acvr2b  Selenop  Tcf7l2  Plekha1  Abl1  Cela1 | web link |
| cellular response to hydrogen peroxide | Gene Ontology | GO:0070301 | 5 (0.68%) | 73 (0.35%) | 0.112525385082 | 0.1831617582 | Apex1  Ppp5c  Abl1  Fxn  Plekha1 | web link |
| cell migration | Gene Ontology | GO:0016477 | 13 (1.77%) | 255 (1.22%) | 0.113912160418 | 0.184075447632 | Cd47  Golph3  Itgav  Coro1b  Nde1  Gfra1  Arpc5  Gpc4  Ndel1  Tns3  Vegfa  Lcp1  Elmo3 | web link |
| negative regulation of neuron death | Gene Ontology | GO:1901215 | 5 (0.68%) | 75 (0.36%) | 0.122414105229 | 0.1949881819 | Ppp5c  Gba  Vegfa  Ghr  Esr1 | web link |
| negative regulation of NF-kappaB transcription factor activity | Gene Ontology | GO:0032088 | 5 (0.68%) | 75 (0.36%) | 0.122414105229 | 0.1949881819 | Erbin  Irak1  Arrb2  Rwdd3  Pkhd1 | web link |
| liver development | Gene Ontology | GO:0001889 | 6 (0.82%) | 97 (0.46%) | 0.124924580513 | 0.197575754996 | Hp  Baat  Mpst  Slco1b2  Rhbdd3  Proc | web link |
| cellular response to starvation | Gene Ontology | GO:0009267 | 5 (0.68%) | 76 (0.36%) | 0.127505237074 | 0.200237097659 | Angptl4  Srebf1  Rragc  Tcf7l2  Gba | web link |
| aging | Gene Ontology | GO:0007568 | 10 (1.36%) | 190 (0.91%) | 0.131353551081 | 0.204838055183 | Dcn  Kynu  Opa1  Apex1  Tyms  Srebf1  Mbd3  Igfbp5  Irak1  Eif5a | web link |
| fat cell differentiation | Gene Ontology | GO:0045444 | 5 (0.68%) | 77 (0.37%) | 0.132691628733 | 0.205487730607 | Fam120b  4932438A13Rik  Srebf1  Tcf7l2  Atf2 | web link |
| negative regulation of neuron apoptotic process | Gene Ontology | GO:0043524 | 9 (1.23%) | 169 (0.81%) | 0.139794248424 | 0.214993913094 | Bcl2l1  Gba  Dnajc5  Aars  Retreg1  Xiap  Arrb2  Ube2v2  Npm1 | web link |
| retina development in camera-type eye | Gene Ontology | GO:0060041 | 5 (0.68%) | 79 (0.38%) | 0.143341976953 | 0.21894014288 | Clcn2  Opa1  Rbp4  Tent2  Cln8 | web link |
| small GTPase mediated signal transduction | Gene Ontology | GO:0007264 | 6 (0.82%) | 102 (0.49%) | 0.148027245263 | 0.224558338053 | Dock9  Ralbp1  Tiam2  Arhgap32  Rhobtb1  Nucb1 | web link |
| multicellular organism growth | Gene Ontology | GO:0035264 | 6 (0.82%) | 103 (0.49%) | 0.152865456754 | 0.228257448165 | Tbl1xr1  Tcf7l2  Plekha1  Ndufs6  Slc25a25  Cela1 | web link |
| negative regulation of angiogenesis | Gene Ontology | GO:0016525 | 6 (0.82%) | 103 (0.49%) | 0.152865456754 | 0.228257448165 | Dcn  Stat1  Gm20498  Cnmd  Gtf2i  Atf2 | web link |
| positive regulation of protein kinase B signaling | Gene Ontology | GO:0051897 | 7 (0.95%) | 126 (0.60%) | 0.153705639773 | 0.228257448165 | Hax1  Tcf7l2  Thpo  Arrb2  Igfbp5  Vegfa  Mup9 | web link |
| regulation of catalytic activity | Gene Ontology | GO:0050790 | 13 (1.77%) | 270 (1.29%) | 0.154559976112 | 0.228257448165 | Plekhg3  Arhgef12  Dock9  Rcc1  Dennd10  Azin1  Madd  Rin3  Septin2  Arhgdib  Nucb1  Lamtor2  Ppp2r5c | web link |
| transforming growth factor beta receptor signaling pathway | Gene Ontology | GO:0007179 | 5 (0.68%) | 82 (0.39%) | 0.159978849253 | 0.231657684307 | Ltbp3  Mtmr4  Arrb2  Arap1  Zyx | web link |
| negative regulation of translation | Gene Ontology | GO:0017148 | 5 (0.68%) | 82 (0.39%) | 0.159978849253 | 0.231657684307 | Tyms  Cnot10  Ybx1  Cpeb3  Igfbp5 | web link |
| regulation of inflammatory response | Gene Ontology | GO:0050727 | 5 (0.68%) | 82 (0.39%) | 0.159978849253 | 0.231657684307 | Sbno2  Tnip1  Mgll  Zyx  Esr1 | web link |
| negative regulation of ERK1 and ERK2 cascade | Gene Ontology | GO:0070373 | 5 (0.68%) | 83 (0.40%) | 0.165691731167 | 0.238382297099 | Abl1  Ndrg2  Tnip1  Dusp3  Gm20498 | web link |
| lipid transport | Gene Ontology | GO:0006869 | 7 (0.95%) | 130 (0.62%) | 0.171698890484 | 0.245441362678 | Gm44805  Hdlbp  Osbpl9  Atp11c  Apoc3  Arv1  Sigmar1 | web link |
| regulation of GTPase activity | Gene Ontology | GO:0043087 | 5 (0.68%) | 85 (0.41%) | 0.177354104751 | 0.251910607386 | Plxnb2  Rabgap1  Fgd6  Arhgap12  Ralbp1 | web link |
| angiogenesis | Gene Ontology | GO:0001525 | 12 (1.63%) | 254 (1.21%) | 0.1820413933 | 0.25693183991 | Ywhaz  Fn1  Fgf1  Rora  Itgav  Gna13  Angptl4  Vegfa  Vezf1  Bcas3  Nrp2  Pnpla6 | web link |
| transcription by RNA polymerase II | Gene Ontology | GO:0006366 | 6 (0.82%) | 110 (0.52%) | 0.188591066854 | 0.26450193653 | Arrb2  Gtf2a2  Gtf2i  Ccnh  Lcorl  Tfdp1 | web link |
| neuron projection development | Gene Ontology | GO:0031175 | 8 (1.09%) | 159 (0.76%) | 0.195003301989 | 0.271785852147 | Ulk1  Camsap2  Uhmk1  Septin2  Gfra1  Ndel1  Atxn10  Mapk9 | web link |
| negative regulation of peptidase activity | Gene Ontology | GO:0010466 | 6 (0.82%) | 113 (0.54%) | 0.204810666953 | 0.283681855469 | Serpina3m  Serpinb6a  Tfpi2  Fetub  Wfdc2  Itih4 | web link |
| protein homooligomerization | Gene Ontology | GO:0051260 | 6 (0.82%) | 114 (0.54%) | 0.210326123414 | 0.289522997045 | Kctd2  Kctd17  Spast  Gsdmd  Kctd21  Lzts3 | web link |
| cellular response to leukemia inhibitory factor | Gene Ontology | GO:1990830 | 6 (0.82%) | 115 (0.55%) | 0.215893141577 | 0.295363009643 | Sbno2  Srsf3  Snx10  Ubxn2a  Abcd4  Nrp2 | web link |
| response to bacterium | Gene Ontology | GO:0009617 | 9 (1.23%) | 192 (0.92%) | 0.231985535838 | 0.315226924945 | Ociad2  Prlr  Cd47  Gsta2  Trf  Iigp1  Cflar  Hp  Mt2 | web link |
| negative regulation of protein phosphorylation | Gene Ontology | GO:0001933 | 5 (0.68%) | 94 (0.45%) | 0.233239652987 | 0.315226924945 | Ppp5c  Paqr3  Arrb2  Pard6a  Spag9 | web link |
| central nervous system development | Gene Ontology | GO:0007417 | 5 (0.68%) | 95 (0.45%) | 0.239741022826 | 0.322061735483 | Tbx3  Gba2  Chrd  Pam  Gba | web link |
| positive regulation of cell migration | Gene Ontology | GO:0030335 | 11 (1.50%) | 245 (1.17%) | 0.241609071139 | 0.32262768182 | Fn1  Spag9  Myo1c  Cep43  Fermt3  Vegfa  Itgav  Aldoa  Ccar1  Fgf1  Sema4g | web link |
| response to estradiol | Gene Ontology | GO:0032355 | 6 (0.82%) | 120 (0.57%) | 0.244439250989 | 0.324464005776 | Ghr  Esr1  Pam  Mapk15  Mbd3  Ncor2 | web link |
| rRNA processing | Gene Ontology | GO:0006364 | 7 (0.95%) | 145 (0.69%) | 0.246259848097 | 0.324946426779 | Exosc10  Rrp36  Sbds  Ddx56  Rps19  Rcl1  Rpl35a | web link |
| cilium assembly | Gene Ontology | GO:0060271 | 9 (1.23%) | 196 (0.94%) | 0.249947608977 | 0.327872451776 | Nudcd3  Fam149b  Snx10  Pcm1  Tctn1  Septin2  Atxn10  Pkhd1  Gm20695 | web link |
| response to drug | Gene Ontology | GO:0042493 | 14 (1.91%) | 329 (1.57%) | 0.265136167478 | 0.344967042343 | Ywhaz  Mapk9  Crot  Acacb  Usp47  Ss18  Comt  Apex1  Tyms  Mgst1  Slco1b2  Akr1c6  Srebf1  Pam | web link |
| actin cytoskeleton organization | Gene Ontology | GO:0030036 | 8 (1.09%) | 174 (0.83%) | 0.266073234453 | 0.344967042343 | Pdlim5  Nisch  Dnajb6  Epb41  Coro1b  Ablim2  Abl1  Atxn3 | web link |
| negative regulation of cell migration | Gene Ontology | GO:0030336 | 6 (0.82%) | 124 (0.59%) | 0.268020425323 | 0.345482975995 | Zmynd8  Nisch  Mapk15  Chrd  Igfbp5  Dusp3 | web link |
| transcription, DNA-templated | Gene Ontology | GO:0006351 | 5 (0.68%) | 100 (0.48%) | 0.272924340202 | 0.347907415273 | Polrmt  Slc39a8  Ccnh  Dido1  Ablim2 | web link |
| protein phosphopantetheinylation | Gene Ontology | GO:0018215 | 9 (1.23%) | 201 (0.96%) | 0.273021514227 | 0.347907415273 | Ero1b  Pomgnt2  Asph  Mgat1  Uba5  Ggt6  Zdhhc20  Atat1  Pam | web link |
| cellular response to interferon-gamma | Gene Ontology | GO:0071346 | 5 (0.68%) | 101 (0.48%) | 0.279677701002 | 0.350809944808 | Cd47  Myo1c  Zyx  Stat1  Cdc42ep4 | web link |
| skeletal system development | Gene Ontology | GO:0001501 | 5 (0.68%) | 101 (0.48%) | 0.279677701002 | 0.350809944808 | Rai1  Acvr2b  Chrd  Ltbp3  Tbx3 | web link |
| neuron migration | Gene Ontology | GO:0001764 | 6 (0.82%) | 126 (0.60%) | 0.28001870034 | 0.350809944808 | Pcm1  Pomgnt2  Ndel1  Nde1  D130043K22Rik  Nrp2 | web link |
| regulation of transcription by RNA polymerase II | Gene Ontology | GO:0006357 | 56 (7.63%) | 1489 (7.10%) | 0.306428714079 | 0.381751973406 | Hsf2  Hsf4  Hdgfl2  Tcf7l2  Zbtb42  Zbtb43  Zfp639  Crem  Rai1  Ccnh  Pam  Tfdp1  Zfp825  Foxk2  Esr1  Mlxipl  Mdm4  Tsc22d3  Rora  Safb  Epc1  Usf2  Gzf1  Zfp874a  Zfp874b  Klf3  Zfp58  Rbm39  Atf2  Zbtb14  Zbtb18  Hnrnpk  Zfp938  Tbx3  Zfp367  Vezf1  Vegfa  Zmiz2  Rfxap  Atxn7l3  Brd3  Gmeb2  Uxt  Tbl1xr1  Zfp362  Zfp780b  Zfpm1  Tef  Zfp62  Hmgb3  Srebf1  Camta2  Eny2  Zfp235  Lcorl  Med7 | web link |
| regulation of cell migration | Gene Ontology | GO:0030334 | 5 (0.68%) | 107 (0.51%) | 0.320794908144 | 0.397429247312 | Plxnb2  Lmna  Enpp2  Gna13  Fgf1 | web link |
| response to ethanol | Gene Ontology | GO:0045471 | 6 (0.82%) | 133 (0.63%) | 0.322851971134 | 0.397767898138 | Tyms  Gk  Rpl15  Srebf1  Rbp4  Fgfr2 | web link |
| immune system process | Gene Ontology | GO:0002376 | 19 (2.59%) | 481 (2.29%) | 0.327710902853 | 0.401535886463 | Tec  Bag6  Irak1  Cd300c2  Akap8  Hp  C4bp  Gsdmd  Gm49673  Ifi30  Dclre1c  Hmgb3  Lgals9  Map4k2  S100a9  AI182371  Pcbp2  Rnaset2b  Iigp1 | web link |
| cytoskeleton organization | Gene Ontology | GO:0007010 | 6 (0.82%) | 136 (0.65%) | 0.341499829721 | 0.41390868014 | Znrf1  Sh3d19  Epb41  Ss18  Mast3  Ablim2 | web link |
| spermatogenesis | Gene Ontology | GO:0007283 | 18 (2.45%) | 458 (2.19%) | 0.341521063434 | 0.41390868014 | Bcl2l1  Dazap1  4932438A13Rik  Bag6  Acox1  Stk11  Kdm5a  Snrpa1  Dedd  Septin2  Hsf2  Rbp4  Crem  Rnf114  Slc4a2  Atat1  Ggnbp1  Plekha1 | web link |
| transmembrane transport | Gene Ontology | GO:0055085 | 22 (3.00%) | 568 (2.71%) | 0.34396725898 | 0.414619993257 | Slc39a11  Slc25a44  Slc8b1  Ralbp1  Slc22a30  Slc17a2  Slc35b3  Slco1b2  Abcd4  Gpr155  Slc39a8  Slc39a9  Slc25a19  Slc43a1  Slc2a9  Slc4a2  Slc9a8  Slc12a7  Clcn2  Gsdmd  Slc25a23  Slc25a25 | web link |
| positive regulation of neuron projection development | Gene Ontology | GO:0010976 | 7 (0.95%) | 164 (0.78%) | 0.351527534448 | 0.421455054741 | Plxnb2  Ddx56  Kidins220  Cflar  Hnrnpk  S100a9  Ube2v2 | web link |
| mRNA splicing, via spliceosome | Gene Ontology | GO:0000398 | 7 (0.95%) | 167 (0.80%) | 0.368704605752 | 0.439685171566 | Srsf3  U2af1  Snrpa1  Hnrnpa2b1  Txnl4a  Snrnp70  Hnrnpm | web link |
| proteolysis | Gene Ontology | GO:0006508 | 25 (3.41%) | 664 (3.17%) | 0.383369983617 | 0.454742055035 | Tysnd1  Rnpepl1  Proc  Jmjd7  Ctsa  Hp  Usp40  Tmprss6  Rhbdf2  Rhbdd3  Mindy3  Ndel1  Josd2  Cela1  Hmces  H13  Ggt6  Atxn3  Bmp1  Desi1  Senp6  Cflar  Atg4d  Usp47  Nln | web link |
| cell differentiation | Gene Ontology | GO:0030154 | 34 (4.63%) | 918 (4.38%) | 0.392469173927 | 0.463072094104 | Sbno2  Pard3  Stk11  Nde1  Vegfa  Tmem176b  Ptbp3  Bin1  Nrp2  Dazap1  Nedd4l  Fam120b  Huwe1  Atraid  Itgav  Gna13  Ndel1  Ndrg2  Ggnbp1  Nr1h3  Bag6  Prrc2c  Prrc2b  Unc45a  Septin2  Fgf1  Sema4g  Smurf1  Bmp1  Spast  Zfpm1  Cnmd  Rnf114  Eya3 | web link |
| negative regulation of endopeptidase activity | Gene Ontology | GO:0010951 | 7 (0.95%) | 176 (0.84%) | 0.420410935375 | 0.493429676783 | Serpina3m  Serpinb6a  Tfpi2  Fetub  AI182371  Wfdc2  Itih4 | web link |
| ion transport | Gene Ontology | GO:0006811 | 23 (3.13%) | 622 (2.97%) | 0.424379640075 | 0.49547989391 | Slc39a11  Slc8b1  Slc17a2  Slc38a10  Fxn  Slc38a4  Slc38a3  Asic5  Nipa2  Slc39a8  Slc39a9  Slc41a2  Slc40a1  Slc9a8  Trf  Slc12a7  Gm16867  Fxyd1  Clcn2  Atp5j  Slco1b2  Slc4a2  Cp | web link |
| negative regulation of cell growth | Gene Ontology | GO:0030308 | 5 (0.68%) | 123 (0.59%) | 0.432146887576 | 0.499319978909 | Nme6  Hyal1  Stk11  Smarca2  Fbp1 | web link |
| positive regulation of MAPK cascade | Gene Ontology | GO:0043410 | 5 (0.68%) | 123 (0.59%) | 0.432146887576 | 0.499319978909 | Fgfr2  Spag9  Vegfa  Igfbp4  Thpo | web link |
| response to organic cyclic compound | Gene Ontology | GO:0014070 | 6 (0.82%) | 151 (0.72%) | 0.435418144985 | 0.50050642439 | Acacb  Idh1  Comt  Tyms  Srebf1  Mbd3 | web link |
| heart development | Gene Ontology | GO:0007507 | 10 (1.36%) | 263 (1.25%) | 0.440810935546 | 0.50181421461 | Pdlim5  Acvr2b  Fn1  Zfpm1  Taz  Rbp4  Prkar1a  Mbd3  Pam  Nrp2 | web link |
| intracellular protein transport | Gene Ontology | GO:0006886 | 11 (1.50%) | 291 (1.39%) | 0.441275645607 | 0.50181421461 | Cttn  Sec23b  Snx10  Rabgap1  Tomm22  Grtp1  Tbc1d17  Copa  Sec24c  Sec24b  Sec31b | web link |
| protein stabilization | Gene Ontology | GO:0050821 | 7 (0.95%) | 180 (0.86%) | 0.443306727705 | 0.50181421461 | Bag6  Mdm4  Ccnh  Tcp1  Ifi30  Clu  Npm1 | web link |
| cellular response to tumor necrosis factor | Gene Ontology | GO:0071356 | 5 (0.68%) | 126 (0.60%) | 0.452774997916 | 0.509943558259 | Smpd4  Hyal1  Erbin  Rora  Gba | web link |
| cell-cell adhesion | Gene Ontology | GO:0098609 | 6 (0.82%) | 155 (0.74%) | 0.460214521973 | 0.515717780905 | Pard3  Gm20498  Igsf5  Cd47  Nectin3  Pkp4 | web link |
| cell projection organization | Gene Ontology | GO:0030030 | 7 (0.95%) | 186 (0.89%) | 0.4773373179 | 0.532231109459 | Fam149b  Snx10  Pcm1  Tctn1  Cep43  Entr1  Gm20695 | web link |
| regulation of cell population proliferation | Gene Ontology | GO:0042127 | 7 (0.95%) | 190 (0.91%) | 0.499719459647 | 0.554415121897 | Bag6  Tbx3  Abl1  Clu  Yap1  Riox2  Cela1 | web link |
| cytokine-mediated signaling pathway | Gene Ontology | GO:0019221 | 5 (0.68%) | 142 (0.68%) | 0.558299002156 | 0.616339987529 | Prlr  Irak1  Lifr  Ghr  Iigp1 | web link |
| positive regulation of protein phosphorylation | Gene Ontology | GO:0001934 | 8 (1.09%) | 235 (1.12%) | 0.582432684047 | 0.639815214495 | Mapk15  Thpo  Mprip  Arrb2  Abl1  Vegfa  Mapk9  Fgf1 | web link |
| negative regulation of gene expression | Gene Ontology | GO:0010629 | 10 (1.36%) | 295 (1.41%) | 0.586625666385 | 0.641262370608 | Ptrh2  Esr1  Acacb  Lgals9  Hnrnpk  Vegfa  Cnpy2  Npm1  Lrp3  Yap1 | web link |
| vesicle-mediated transport | Gene Ontology | GO:0016192 | 8 (1.09%) | 249 (1.19%) | 0.647312343349 | 0.704149524717 | Bet1  Copa  Sec23b  Spag9  Snap23  Kxd1  Mcfd2  Sec31b | web link |
| positive regulation of ERK1 and ERK2 cascade | Gene Ontology | GO:0070374 | 7 (0.95%) | 223 (1.06%) | 0.668093228325 | 0.723227135517 | Esr1  Thpo  Cflar  Arrb2  Prxl2c  Abl1  Vegfa | web link |
| nervous system development | Gene Ontology | GO:0007399 | 13 (1.77%) | 410 (1.96%) | 0.681860730248 | 0.734564941281 | Fn1  Spast  D130043K22Rik  Atxn10  Gfra1  Ndel1  Nde1  Vegfa  Ndrg2  Cln8  Sigmar1  Nrp2  Sema4g | web link |
| response to hypoxia | Gene Ontology | GO:0001666 | 6 (0.82%) | 197 (0.94%) | 0.691755180891 | 0.741641371821 | Cflar  Alas1  Angptl4  Vegfa  Aldoa  Pam | web link |
| positive regulation of cytosolic calcium ion concentration | Gene Ontology | GO:0007204 | 5 (0.68%) | 167 (0.80%) | 0.699737090225 | 0.746609431197 | Abl1  Esr1  Gna13  Itgav  Eif5a | web link |
| inflammatory response | Gene Ontology | GO:0006954 | 10 (1.36%) | 344 (1.64%) | 0.768459373211 | 0.816030667744 | Hyal1  Gsdmd  Itgav  Cd47  Tnip1  S100a9  Igfbp4  AI182371  Mapkapk2  Cela1 | web link |
| positive regulation of apoptotic process | Gene Ontology | GO:0043065 | 10 (1.36%) | 360 (1.72%) | 0.813644179575 | 0.859917782205 | Bcl2l1  Ccar1  Dffa  Tcf7l2  Tomm22  Abl1  Clu  Mapk9  Bin1  Eif5a | web link |
| cellular response to lipopolysaccharide | Gene Ontology | GO:0071222 | 5 (0.68%) | 197 (0.94%) | 0.823728013235 | 0.866468617696 | Abl1  Sbno2  Irak1  Akap8  Nr1h3 | web link |
| regulation of transcription, DNA-templated | Gene Ontology | GO:0006355 | 43 (5.86%) | 1403 (6.69%) | 0.840672311278 | 0.880140494906 | Sbno2  Hsf2  Zfp825  Taf1d  Emsy  Zkscan3  Crem  Hdac10  Zfp938  Cnot2  Tfdp1  Hsf4  Foxk2  Esr1  Mier1  Safb  Slc39a8  Zfp874a  Zfp874b  Mterf3  Rnf141  Zfp58  Ybx1  Mta2  Nr1h3  Dhrs7b  Acvr2b  Zmynd8  Stat1  Srrt  Rora  Tbx3  Zfp235  Tcf7l2  Gtf2i  Smarca2  Sap130  Lrrfip2  Zfp780b  Tef  Srebf1  Ccar1  Atf2 | web link |
| regulation of gene expression | Gene Ontology | GO:0010468 | 10 (1.36%) | 376 (1.79%) | 0.851881801731 | 0.887708606477 | Dhrs7b  Trim11  Tbl1xr1  Fn1  Atraid  Hnrnpk  Pcbp2  Ubxn2a  Ybx1  Yap1 | web link |
| innate immune response | Gene Ontology | GO:0045087 | 15 (2.04%) | 560 (2.67%) | 0.886664753918 | 0.919656930808 | Trim11  Map4k2  Wfdc2  Hmgb3  Akap8  C4bp  Gsdmd  Irak1  Gm49673  Pcbp2  Ankhd1  AI182371  S100a9  Rnaset2b  Iigp1 | web link |
| brain development | Gene Ontology | GO:0007420 | 6 (0.82%) | 258 (1.23%) | 0.892114203764 | 0.921025312219 | Plxnb2  Bag6  Slc38a3  Selenop  Arrb2  Mbd3 | web link |
| multicellular organism development | Gene Ontology | GO:0007275 | 29 (3.95%) | 1040 (4.96%) | 0.918465174752 | 0.943860525206 | Ube2i  Chrd  Vegfa  Churc1  Nde1  Cnot2  Bin1  Kdm5a  Nrp2  Plxnb2  Dazap1  Banp  Cnmd  Itgav  Ndel1  Ndrg2  Ggnbp1  Tctn1  Zbtb18  Unc45a  Rora  Tbx3  D130043K22Rik  Fgf1  Sema4g  Spast  Bmp1  Rnf114  Eya3 | web link |
| positive regulation of gene expression | Gene Ontology | GO:0010628 | 12 (1.63%) | 525 (2.50%) | 0.958456113009 | 0.980439051381 | Lrp3  Mapk9  Fn1  Mup9  Lgals9  Arrb2  Vegfa  Clu  Lmna  Atf2  Fgf1  Yap1 | web link |
| negative regulation of cell population proliferation | Gene Ontology | GO:0008285 | 8 (1.09%) | 392 (1.87%) | 0.966828030184 | 0.984486989639 | Inca1  Asph  Stk11  Rassf5  Lmna  Npm1  Smarca2  Ppp2r5c | web link |
| cell adhesion | Gene Ontology | GO:0007155 | 11 (1.50%) | 571 (2.72%) | 0.990889859214 | 1.0 | Pard3  Fn1  Fermt3  Pip5k1c  Cd47  Itgav  Nectin3  Pkp4  Abl1  Mpdz  Zyx | web link |
| biological\_process | Gene Ontology | GO:0008150 | 72 (9.81%) | 2834 (13.52%) | 0.999230860815 | 1.0 | Bri3bp  Srek1  Gpank1  Gm826  Zfp825  Taf1d  Ccdc163  Synrg  Szrd1  Haghl  Tmem234  Tmem222  Smim1  Zfand4  Zfand3  1110032A03Rik  Mmaa  1110065P20Rik  C87436  Mcrip2  Smim10l1  Card19  Hemk1  Wdr53  Chchd7  Mrpl30  Slc39a9  Fam204a  Tmem256  Ccdc30  Atraid  Mup9  Iqcc  Golga1  Ttc13  2700062C07Rik  Fam234a  Polr1has  Gm527  Fgd6  Ccdc97  Mettl9  1110008P14Rik  9930021J03Rik  Fbxo34  Wbp1l  Ggt6  Rcbtb2  Susd1  Pcnx3  Cdv3  Tmem185a  Gtpbp8  Msantd2  Ntpcr  Rsrp1  Chchd1  Cep170  Plekha6  Nomo1  Gramd3  Ccdc58  Fance  Pdrg1  Srsf11  Wsb1  Wdr20  Uhrf1bp1l  Mrnip  Zcchc7  Btf3l4  Arl16 | web link |
| G protein-coupled receptor signaling pathway | Gene Ontology | GO:0007186 | 7 (0.95%) | 2101 (10.02%) | 1.0 | 1.0 | Arhgef12  Arrb2  Gna13  Gngt1  Rgs3  P2ry2  Gprc5c | web link |
| signal transduction | Gene Ontology | GO:0007165 | 32 (4.36%) | 2811 (13.41%) | 1.0 | 1.0 | Gngt1  Ralbp1  Pkig  Thpo  Raph1  Arhgap32  Vegfa  Irak1  Plxnb2  Copa  Stat1  Rin3  Gna13  Rgs3  Arhgap5  Ndrg2  Acvr2b  Ulk1  Arap1  Rassf5  Tiam2  Manf  P2ry2  Zyx  Ywhaz  Bmp1  Erbin  Rps6kb2  Agap3  Arrb2  Arhgap12  Ppp2r5c | web link |
