# Supplementary material for: Regulation of Alternative Splicing of Lipid Metabolism Genes in Sepsis-Induced Liver Damage by RNA-Binding Proteins
Source: Inflammation. 2024 May 9;47(6):1952–68. doi: 10.1007/s10753-024-02017-2 (PMC11607010; doi:10.1007/s10753-024-02017-2)
Supplement: Supplementary file 1 — Supplementary file1 (ZIP 38655 KB) [file 10753_2024_2017_MOESM1_ESM.zip › Sup5_AS_GO.zip!/Sup5_AS_GO/ras/CLP_vs_Sham/Ras_GO_analysis/Ras_GO_enrichment_web_C.html]

GO enrichment analyze


# GO enrichment analyze result


| Term | Database | ID | Input number (805) | Background number (20939) | P-Value | Corrected P-Value | Input | Hyperlink |
| --- | --- | --- | --- | --- | --- | --- | --- | --- |
| cytosol | Gene Ontology | GO:0005829 | 235 (29.19%) | 3762 (17.97%) | 1.33226762955e-15 | 1.58539847916e-13 | Ccdc91  Pdlim5  Dop1b  Uox  Usp40  Gemin7  Fbxl12  Mon2  Itgav  Blvrb  Cnot2  Mapkapk5  Tprkb  Naa80  Tfdp1  Synrg  Vcpkmt  Cdv3  Sgk1  Cox19  Pip5k1c  Usp47  Taf1d  Uba52  Mt2  Tcp1  Gak  Apoo  Chmp3  Senp6  Arap1  Zbtb14  Mterf3  Atg16l2  Unc45a  Mri1  Spag9  Mpst  Mapk6  Gne  Abl1  Cenpv  Mapk9  Atxn3  Fgf1  Akr1c20  Exosc10  Upp2  Cttn  Cep170  Spast  Huwe1  Extl2  Desi1  Rpl35a  Riox2  Farsa  Tnip1  Dnajc5  Hnrnpl  Gk  Ncor1  Ptrh2  Ppp5c  Eef1d  Opa1  Ubap1  Sec23b  Rpl15  Lgals9  Uba5  Plekha1  Ubxn4  Gm44805  Irak1  Atat1  Yap1  Bcl2l1  Smg7  Rps6ka3  Ghr  Mthfs  Bin1  Dnajb6  Cpeb3  Adk  Camsap2  Gna13  Sec24c  Sec24b  Ybx1  Thada  Echdc1  S100a16  Madd  Kynu  Slc40a1  Stat1  Prrc2c  Tnrc18  Gstm4  Tcf7l2  Cutc  Per1  Txnl4a  Xylb  Ppp4r3a  Zyx  Ywhaz  Tent2  Dcun1d1  Fkbp4  Sbds  Xiap  Osbpl9  Haao  Ubxn2a  Gba2  Zcchc7  Alkbh3  Nln  Ube2i  Golph3  Sult1b1  Acox2  Pcm1  Acox1  Ces1f  Ifi30  Dffa  Coro1b  Tyms  Aox3  Clu  Bdh2  Mtmr2  Aasdhppt  Trim11  Dazap1  Chchd1  Hmbox1  Mlxipl  Man1a  Comt  Mup9  Ndel1  Tns3  Lmna  Dusp16  Idh1  Nr1h3  Rpl22l1  Bag6  Acp1  Slc66a2  Btrc  Eif3e  Epb41  Mprip  Ahsa2  S100a9  Ube2g2  Phldb2  Akr1e1  Kif21a  Tbc1d17  Smurf1  Fam160a2  Raph1  Rabgap1  Flad1  Gsdmd  Arrb2  Gmeb2  Akr1c6  Dusp12  Nudcd2  Grhpr  Znrf1  Cuedc2  Fmo5  Ppp2r2d  Mgll  Orc4  Stk11  Baat  Abhd14b  Fxn  Fbp1  Lcp1  Npm1  Pam  Dusp3  Exoc7  Rnaseh2a  Csnk1a1  Cryz  Pnpla2  Sh3d19  Rpl18  Helz  Gckr  Hnrnpk  Mocs2  Mocs1  Arhgdib  Ubc  Atxn10  Arhgap5  Josd2  Thoc7  Tacc2  Pnpla6  Lyplal1  Rps10  Atg16l1  Rps19  Hdlbp  Eif5  Alas1  Akt1s1  Uhrf1bp1l  Aldoa  Upf3b  Nisch  Myo1c  Gsta2  Aprt  Rnf114  Ppp6r3  Ppp6r1  Pcbp2  Gpcpd1  Setd4  Ppp2r5c | web link |
| mitochondrion | Gene Ontology | GO:0005739 | 130 (16.15%) | 1745 (8.33%) | 1.15463194561e-13 | 6.87006007638e-12 | Slc8b1  Apoo  Uox  Gm49673  Tomm5  Opa1  Hemk1  Nme6  Mapk9  Sgk1  Esr1  Ctsa  Snap23  Atg4d  Ggnbp1  Atf2  Bri3bp  Mthfs  Mpst  Mrpl47  Abl1  Mrpl48  Nudt9  Nipsnap1  Maob  Polg2  Dcakd  Rpl35a  Taz  Nudt13  Hnrnpk  Mtif3  Eny2  Nmnat3  Gk  Oxnad1  Hoga1  Idh1  Sugct  Slc35b3  Higd1a  Immt  Gm20498  Mrpl36  Foxk2  Mrpl30  Atp5j  Uqcr11  Slc25a19  Ghr  Tnrc18  Tomm22  Hax1  Card19  Ssbp1  Trmt5  Polrmt  Zmiz2  Ywhaz  Mrpl24  Dna2  Rab4b  Mff  Fkbp4  Slc25a23  Mgst1  Mpv17l2  Slc25a25  Alkbh3  Nln  Ndufa5  Slc25a44  Ptrh2  Acox1  Tyms  Clu  Chchd1  Chchd7  Cbr4  Golph3  Comt  Agxt2  Mccc2  Ociad2  Tmem11  Gm16867  P4ha1  Rnaset2b  Etfbl  Smurf1  Naxd  Mettl17  Rtn4ip1  Rdh13  Cox7a2  Clpb  Trak1  Cln8  Aars  Apex1  Fxn  Mmaa  Dbi  Mtfr1l  Pnkp  Ccdc58  Stk11  Gckr  Cox19  Ndufs2  Pnkd  Ndufs6  Mterf3  Gls2  Bcl2l1  Letmd1  Acacb  Akap8  Ndufb2  Alas1  Brd8  Adhfe1  Aldoa  Gtpbp8  Timm17b  Cyb5r3  Crot  Isca2  Glrx2  Pnpla8 | web link |
| nucleus | Gene Ontology | GO:0005634 | 322 (40.00%) | 6031 (28.80%) | 2.46103137869e-12 | 9.76209113546e-11 | Slc39a11  Cers2  Rai1  Taf1b  Safb  Ube2d3  Hnrnpk  Hnrnpl  Hnrnpm  Edc4  Lcorl  Ghr  Mapkapk5  Mcrip2  Anapc5  Mapkapk2  Tfdp1  Dffa  Gm49325  Anp32a  Sgk1  Esr1  Mier1  Mier2  Cnot10  Kdm5a  Uba52  Inpp4a  Epc1  Nap1l4  Pnkd  Klf3  Fgf1  Gtf2a2  Rgs3  Smarca2  Clk4  Bcas3  Rbm3  Atf2  Exosc10  Mindy3  Zbtb14  Glrx2  Tacc2  Inca1  Unc45a  Ncapd3  Septin2  Zfp367  Med7  Cenpx  Abl1  Cenpv  Mapk9  Shld2  Sigmar1  Thoc2l  Fbxl12  Rcc1  Uxt  Spast  Snx10  Fgfr2  Nolc1  Desi1  Ddx56  Zkscan3  Rps10  Helz  Mettl23  Smpd4  Fam120b  Hnrnpa2b1  Ddb2  Nmnat3  Mrnip  Gk  Ncor2  Ncor1  C87436  Actn1  Tial1  Nup98  Ppp5c  Snrk  Pkig  Cpsf6  Rpl15  Lgals9  Uba5  Ptbp3  Crem  Ptbp2  Hdac10  Irak1  Iigp1  Trim7  Yap1  Bcl2l1  Smg7  Rps6ka3  Hnrnpdl  Pmf1  Pcnp  Bin1  4932438A13Rik  Ppp2r5c  Dnajb6  Cpeb3  Mtmr2  Mapk15  Tnip1  Gna13  Mbd3  Son  Arrb2  Ndrg2  Ybx1  Riox2  S100a16  Dhrs7b  Stat1  Gemin7  Rassf5  Hax1  Uhmk1  Tcf7l2  Ddb1  Kxd1  Per1  Trmt5  Cep43  Ccar1  Txnl4a  Gtf2i  Ppp4r3a  Psmc6  Luc7l2  Ywhaz  Dna2  Ints7  Tent2  Zfp58  Zfp780b  Erbin  Parp3  Fkbp4  Sbds  Xiap  Mgst1  Sfswap  Cpsf1  Ubxn2a  Churc1  Cdk8  Zfp235  Nucb1  Zfp62  Rbm39  Sbno2  Rwdd3  Taf1d  Mt2  Tyms  Ss18  Usp40  Csnk1g2  Csnk1g3  Zbtb42  Zbtb43  Vegfa  Pard6a  Wac  Zmiz2  Bivm  Clu  Mdm4  Adk  Zfp825  Trim11  Dazap1  Chchd1  Cnot2  Srsf3  Hmbox1  Mlxipl  Huwe1  Atraid  Arpc5  Mup9  Dnajb12  Gzf1  Zyx  Mri1  Rexo5  Lmna  Dusp16  Dusp11  Orc4  Nr1h3  Dbi  Emsy  Bag6  Srek1  Srrt  Btrc  Azin1  Eif3e  Rora  Epb41  Upf3b  S100a9  Pkp4  Ppp6r1  Eapp  Ccnh  Atxn7l3  Akr1c6  Sap130  Aen  Tbl1xr1  Cutc  U2af1  Senp6  Hdgfl2  Cdk2ap2  Snrpa1  Tef  Cbs  Srebf1  Camta2  Mta2  Zfp639  Gmeb2  Banp  Ube2v2  Dusp12  Rcl1  Trak1  Ercc5  Plekha1  Ube2i  Hnrnpll  Hsf2  Macroh2a1  Hsf4  Cuedc2  Uckl1  Rtca  Stk11  Brd3  Abhd14b  Apex1  Esrp2  Alkbh2  Fbp1  Inip  Tmem176b  Atxn3  Pkhd1  Cpsf7  Rps6kb2  Npm1  N4bp2l2  Dusp3  1110032A03Rik  Rnaseh2a  Csnk1a1  Pnkp  Kmt5b  Eny2  Tsc22d3  Higd1a  Gckr  Usf2  Wdr37  Usp47  Zfp874a  Zfp874b  Ubc  Nsd1  Thoc7  Dclre1c  Ubox5  Eya3  Zbtb18  Jmjd7  Rragc  Acacb  Zmynd8  Dedd  Hdlbp  Akap8  Zfp938  Unc50  Dcun1d1  Akt1s1  Brd8  Tbx3  Foxk2  Rfxap  Nemf  Chd2  Rbm47  Hmgb3  Zcchc7  Dido1  Zfpm1  Myo1c  Vezf1  Alkbh3  Akap8l  Rnf114  Mapk6  Ppp6r3  Hnrnph3  Tprkb  Pcbp2  Snrnp70  Setd4  Eif5a | web link |
| cytoplasm | Gene Ontology | GO:0005737 | 347 (43.11%) | 6677 (31.89%) | 6.66489086143e-12 | 1.98280503128e-10 | Rbm3  Nde1  Naa80  Cdv3  Tent2  Mier2  Cnot10  Rin3  Atg4d  Cyp2r1  Ggnbp1  Kctd17  Arap1  Tacc2  Inca1  Nap1l4  Tiam2  Fgf1  Exosc10  Cep170  Cutc  Tnip1  Hnrnpa2b1  Senp6  Kxd1  Ccar1  Ddb1  Gk  Yap1  Bcl2l1  Ghr  Dnajb6  Ndrg2  Fgfr2  Echdc1  Madd  Stat1  Camsap2  Uhmk1  Mtmr14  Txnl4a  Sorbs2  Ppp4r3a  Psmc6  Ints7  Parp3  Xiap  Trmt5  Mpv17l2  Usp47  Nudcd2  Csad  Tyms  Hsf2  Bivm  Mlxipl  Dffa  Zmynd8  Srrt  Cmah  Azin1  Eif3e  S100a9  Phldb2  Smurf1  Ankrd13a  Rabgap1  Serpinb6a  Cbs  Arrb2  Fbxo6  Gmeb2  Akr1c6  Grhpr  Aars  Apex1  Fxn  Kctd20  Lzts3  Csnk1a1  Cryz  Paox  Tsc22d3  Cox19  Pnkd  Rps10  Jmjd7  Rps19  Uhrf1bp1l  Aldoa  Elmo3  Nucb1  Trak1  Mgll  Sec31b  Aprt  Mapkapk5  Tprkb  Mapkapk2  Bcas3  Atf2  Mthfs  Ubc  Unc45a  Pcbp2  Zdhhc20  Blvrb  Coro1b  Arhgef12  Enpp2  Zbtb42  Slmap  Rai1  Farsa  Cpsf7  Cpsf6  Dram2  Snrk  Rspry1  Asph  Uba5  Plekha1  Atat1  Foxk2  Bdh2  Adk  Mbd3  Ybx1  Gm20498  Rassf5  Gstm4  Per1  Pkp4  Dido1  Dbi  Nedd4l  Haao  Nln  Acox1  Clu  Bin1  Dazap1  Arpc5  Gzf1  Ndel1  Dusp16  Dusp12  Acp1  Btrc  Epb41  Upf3b  Cyp4a31  Zfyve19  Pfkfb2  Srebf1  Uckl1  Stk11  Baat  Akr1e1  Arhgap12  Cyp2c40  Cluh  Rnf167  Gckr  Uba52  Dedd  Rragc  Hyal1  Cdc42ep4  Akt1s1  Pnpla2  Rnf114  Snrnp70  Eif5a  Pcmtd2  Pdlim5  Slc39a11  Fbxl12  Anapc5  Eapp  Tfdp1  Pan3  Vcpkmt  Esr1  Pip5k1c  Inpp4a  Tcp1  Rgs3  Chmp3  Sec23b  Mpst  Ablim2  Cdk2ap2  Upp2  Uxt  Cttn  Spast  Nolc1  Polg2  Mettl23  Sult1b1  Actn1  Ppp5c  Ubap1  Lgals9  Crem  Tmbim6  Hdac10  Irak1  Iigp1  Ces1f  Hnrnpdl  Gemin7  Mcrip2  Mapk15  Fgd6  Mpdz  Ywhaz  Dna2  Aamdc  Fkbp4  Eya3  Mt2  Pcm1  Mtmr4  Mtmr2  Trim11  Septin9  Hmbox1  Mprip  Kif21a  Desi1  Gsdmd  Prkar1a  Erbin  Ube2i  Nudcd3  Pard3  Cuedc2  Pkhd1  Rps6kb2  Npm1  Copa  Sh3d19  Anp32a  Atxn10  Thoc7  Acvr2b  Akap8  Cep43  Dcun1d1  Hmgb3  Akap8l  Albfm1  Ppp6r3  Ppp6r1  Gpcpd1  Hnrnpk  Hnrnpl  Hnrnpm  Tial1  Opa1  Cnot2  Man2c1  Sgk1  Atg16l1  Atg16l2  Septin2  Mapk6  Gne  Abl1  Cenpv  Mapk9  Atxn3  Rcc1  Cyp2d10  Snx10  Dnajc5  Mgat1  Khk  Nudt14  Ppp2r2d  Synrg  Pkig  Ankhd1  Cdc123  Rpl18  Trim7  Smg7  Rps6ka3  Cpeb3  Gna13  S100a16  Kynu  Nisch  Tctn1  Hax1  Rnf220  Gtf2i  Zyx  Spag9  Sbds  Pdrg1  Sult2a8  Prrc2c  Alkbh3  Aox3  Rwdd3  Snap23  Csnk1g2  Csnk1g3  Arhgap32  Vegfa  Ankib1  Fbxw5  Aasdhppt  Huwe1  Comt  Pard6a  Mri1  Idh1  Nr1h3  Bag6  Exoc7  Flad1  Clpb  Itih4  Srsf3  Tmem131l  Entr1  Fbp1  Mmaa  Lcp1  Trim3  Map4k2  Mocs2  Wdr37  Arhgdib  Josd2  Hdlbp  Gmppa  Eif5  Cyb5r3  Myo1c  Anapc10  Cflar  Setd4 | web link |
| nucleoplasm | Gene Ontology | GO:0005654 | 187 (23.23%) | 3156 (15.07%) | 3.17414095008e-10 | 7.55445546119e-09 | Rbm3  Srek1  Dnajb6  Hnrnpk  Hnrnpl  Hnrnpm  Edc4  Opa1  Cnot2  Mapkapk5  Ccnh  Mapkapk2  Tfdp1  Man2c1  Pip5k1c  Mier1  Mier2  Taf1d  Uba52  Inpp4a  Epc1  Klf3  Gtf2a2  Atg4d  Smarca2  Rbm39  Mta2  Atf2  Arap1  Zbtb14  Atg16l2  Zbtb18  Ncapd3  Septin2  Pcbp2  Med7  Abl1  Cenpv  Atxn3  Fgf1  Exosc10  Rcc1  Spast  Inca1  Extl2  Arhgap32  Pcnp  Tnip1  Hnrnpa2b1  Eny2  Cpsf6  Mrnip  Nolc1  Ncor1  C87436  Blvrb  Nup98  Eef1d  Ccdc91  Macroh2a1  Plekha1  1110032A03Rik  Hdac10  Irak1  Rps6ka3  Foxk2  Pmf1  Rai1  Safb  Gemin7  Cpeb3  Mbd3  Riox2  Ncor2  Slc40a1  Stat1  Tnrc18  Uhmk1  Tcf7l2  Dcaf11  Ddb2  Per1  Txnl4a  Gtf2i  Ppp4r3a  Dido1  Luc7l2  Dna2  Vezf1  Alas1  Parp3  Fkbp4  Sbds  Srsf11  Cpsf1  Cdk8  Alkbh2  Alkbh3  Eya3  Taf1b  Snap23  Wac  Zbtb42  Zfp639  Zmiz2  Mdm4  Adk  Trim11  Dazap1  Chchd1  Hmbox1  Mlxipl  Huwe1  Gzf1  Mri1  Lmna  Dusp16  Dusp11  Mettl17  Dusp12  Emsy  Bag6  Srrt  Rora  Upf3b  S100a9  Aen  Sh3d19  Tbl1xr1  Cutc  Senp6  Pfkfb2  Gsdmd  Snrpa1  Srebf1  Arrb2  Gmeb2  Ube2v2  Nr1h3  Ube2i  Hsf2  Cuedc2  Mgll  Rtca  Stk11  Abhd14b  Apex1  Esrp2  Inip  Zmynd8  Npm1  Dusp3  Rnaseh2a  Banp  Rrp36  Pnpla2  Kmt5b  Higd1a  Gckr  Usf2  Mocs2  Ndufs2  Mindy3  Ubc  Dclre1c  Ubox5  Letmd1  Rragc  Rps19  Akap8  Dcun1d1  Aprt  Brd8  Orc4  Hnrnpdl  Nisch  Pnkp  Myo1c  Glrx2  Fam120b  Ddb1  Tacc2  Ppp6r3  Hnrnph3  Zfp367  Snrnp70  Ppp2r5c | web link |
| endoplasmic reticulum | Gene Ontology | GO:0005783 | 102 (12.67%) | 1518 (7.25%) | 1.97665868118e-08 | 3.92037305101e-07 | Cers2  Ktn1  Apoo  Ugt1a6b  Tmbim6  Card19  Tm7sf2  Sgk1  Tmem258  Pigp  Rhbdf2  Pigv  Cyp2r1  Ptdss1  Kctd17  Sec23b  Rer1  Manf  Zdhhc20  Arv1  Sigmar1  Hsd17b6  Cyp2d10  Spast  Snx10  Extl2  Cyp4b1  Smpd4  Atp11c  Ugt1a9  Asph  Retreg1  Sc5d  Uba5  Cnpy2  Ubxn4  Rpl18  Iigp1  Ces1f  Bcl2l1  Hp  BC031181  Elovl5  Derl2  Thada  Lpcat3  Dhrs7b  H13  Hax1  Abl1  Ugt1a7c  Gm49673  Sec61a2  Dbi  Mgst1  Ubxn2a  Gba2  Sec31b  Entpd5  Derl1  Arhgap32  Clu  Proc  Uggt1  Man1a  Dnajb12  Dpagt1  St6gal1  Pomgnt2  Cmah  Vkorc1l1  P4ha1  Ube2g2  Tmem208  Rnaset2b  Bet1  Srebf1  Mcfd2  Cln8  Fmo5  Tmem131l  Ero1b  Calu  Apex1  Cyp2c40  Pkhd1  Abcd4  Pgap2  Gba  Fkbp7  Yif1b  Anp32a  Arhgap5  Lman2l  Icmt  Insig2  Cyb5r3  Ces3a  Nomo1  Pnpla6  Pnpla8  Eif5a | web link |
| endoplasmic reticulum membrane | Gene Ontology | GO:0005789 | 65 (8.07%) | 903 (4.31%) | 9.00253975367e-07 | 1.53043175812e-05 | Cers2  Fmo5  Apoo  Asph  Calu  Lpcat3  Derl1  Derl2  Uba5  Cyp2c40  Tmbim6  Arhgap32  Srebf1  Abcd4  Card19  Pgap2  Tm7sf2  Sgk1  Ero1b  Iigp1  Dnajb12  Rhbdf2  Mgst1  Yif1b  Pigv  Sec24b  Cyp2r1  Lman2l  Sigmar1  Icmt  Dhrs7b  Ptdss1  Dpagt1  Sec24c  Sec23b  Eif5a  Cyp3a59  Pomgnt2  H13  Bet1  Retreg1  Ugt1a7c  Insig2  Ugt1a9  Tmem208  Arv1  Vkorc1l1  Atxn3  Sec61a2  Gm4951  Cyp2d10  Cyb5r3  Spast  Sc5d  Extl2  Cyp4b1  Nomo1  Smpd4  Elovl5  Atp11c  Pnpla6  Gba2  Pnpla8  Sec31b  Cln8 | web link |
| mitochondrial inner membrane | Gene Ontology | GO:0005743 | 36 (4.47%) | 421 (2.01%) | 7.08602177468e-06 | 0.000105404573898 | Ndufa5  Slc25a44  Slc8b1  Apoo  Tyms  Tmem11  Opa1  Clu  Immt  Tmem256  Bcl2l1  Atp5j  Uqcr11  Higd1a  Ndufs2  Ndufs6  Timm17b  Letmd1  Slc25a19  Gm20498  Rdh13  Ndufb2  Mpst  Ugt1a7c  Slc25a25  Ociad2  Gm16867  Nipsnap1  Cyb5r3  Maob  Taz  Slc25a23  Mgst1  Mpv17l2  Tomm22  Cox7a2 | web link |
| nuclear membrane | Gene Ontology | GO:0031965 | 21 (2.61%) | 236 (1.13%) | 0.000326853903053 | 0.00432173494036 | Bcl2l1  Nudt9  Cuedc2  Spast  Nup98  Pcm1  Tnrc18  Hax1  Inpp4a  Epc1  Dnajb12  Rcc1  Txnl4a  Mindy3  Tmem176b  Cenpv  Lmna  Erbin  Ybx1  Iigp1  Aen | web link |
| mitochondrial intermembrane space | Gene Ontology | GO:0005758 | 11 (1.37%) | 95 (0.45%) | 0.00104531873948 | 0.0124392929998 | Rnaset2b  Chchd7  Golph3  Hax1  Immt  Cox19  Opa1  Gm49673  Clpb  Ggnbp1  Nln | web link |
| ER to Golgi transport vesicle membrane | Gene Ontology | GO:0012507 | 5 (0.62%) | 22 (0.11%) | 0.00126585160377 | 0.0135392336421 | Srebf1  Sec23b  Sec31b  Sec24c  Sec24b | web link |
| mitochondrial outer membrane | Gene Ontology | GO:0005741 | 16 (1.99%) | 177 (0.85%) | 0.00136530087147 | 0.0135392336421 | Bcl2l1  Tomm5  Cyb5r3  Gm20498  Mff  Mgst1  Tomm22  Hax1  Letmd1  Maob  Rtn4ip1  Taz  Opa1  Bri3bp  Gk  Atf2 | web link |
| protein-containing complex | Gene Ontology | GO:0032991 | 50 (6.21%) | 869 (4.15%) | 0.00303165653568 | 0.0272777782604 | Ndufa5  Ppp5c  Stk11  Neb  Dffa  Ugt1a6b  Pard3  Clu  Mapkapk5  Irak1  Npm1  Akt1s1  Uggt1  Mettl23  Vcpkmt  Tmem258  Mier1  Mier2  Higd1a  Kidins220  Pard6a  Mbd3  Pcm1  Rbm39  Acvr2b  Stat1  Dazap1  Srrt  Epb41  Cdk8  Slc12a7  Ugt1a7c  Mapk6  Abl1  Ywhaz  Rcc1  Aldoa  Mff  Desi1  Rnf220  Esr1  Polrmt  Nomo1  Fkbp4  Xiap  Srebf1  Prkar1a  Ddb2  Rbp4  Ddb1 | web link |
| Golgi apparatus | Gene Ontology | GO:0005794 | 70 (8.70%) | 1309 (6.25%) | 0.00320915038357 | 0.0272777782604 | Slc39a11  Nedd4l  Golph3  Dop1b  Synrg  Naa60  Ccdc91  Osbpl9  Slc38a10  St3gal4  Uba5  Gfra1  Copa  Pkhd1  Trim3  Yif1b  Proc  Iigp1  Atat1  Pgap2  St3gal5  Pmf1  Esr1  Slc9a8  Golga1  Hp  St6gal1  Mapk15  Tcp1  Gak  Apoo  Man1a  Ndrg2  Lman2l  Ggnbp1  Lysmd3  Nagpa  Zmynd8  Arap1  Gba  Camsap2  Unc45a  Akap8  Uhmk1  Retreg1  Map4k2  Rer1  Zdhhc20  Arv1  Unc50  Arhgap32  Dbi  Bet1  Cttn  Bmp1  Calu  Dnajc5  Paqr3  Enpp2  Slc35b3  Dclre1c  Mgat1  Srebf1  Dram2  Gcc2  Smpd4  Mcfd2  Gba2  Nucb1  Ppp2r5c | web link |
| endoplasmic reticulum-Golgi intermediate compartment | Gene Ontology | GO:0005793 | 9 (1.12%) | 81 (0.39%) | 0.00386018422699 | 0.0276184417774 | Fn1  Nucb1  Man1a  Yif1b  Mcfd2  Rer1  Uggt1  Lman2l  Cln8 | web link |
| nuclear speck | Gene Ontology | GO:0016607 | 27 (3.35%) | 403 (1.92%) | 0.003867403072 | 0.0276184417774 | Srek1  Hsf4  Srsf3  Wac  Apex1  Npm1  Rfxap  Csnk1a1  Sgk1  Mocs2  Lmna  Rbm39  Zbtb18  Unc45a  Ppp4r3a  Sap130  Luc7l2  Snrnp70  Dusp11  U2af1  Erbin  Snrpa1  Son  Akap8l  Srsf11  Cpsf6  Thoc7 | web link |
| ribonucleoprotein complex | Gene Ontology | GO:1990904 | 14 (1.74%) | 162 (0.77%) | 0.00398960070937 | 0.0276184417774 | Dazap1  Csnk1a1  Tial1  Esrp2  Srrt  Nup98  Akap8l  Hnrnpl  Hnrnpa2b1  Hnrnph3  Cpsf6  Npm1  Ybx1  Hnrnpm | web link |
| perinuclear region of cytoplasm | Gene Ontology | GO:0048471 | 44 (5.47%) | 755 (3.61%) | 0.00417757942852 | 0.0276184417774 | Stat1  Synrg  Dnajb6  Apex1  Abl1  Hnrnpl  Clu  Pkhd1  Lcp1  Mtmr2  Mtmr14  Gm20498  Sgk1  Esr1  Atraid  Pam  Golga1  Anp32a  Atxn10  Ndrg2  Ybx1  Sec23b  Unc45a  Cep43  Trf  Septin2  Pkp4  Manf  Zdhhc20  Septin9  Sorbs2  Lmna  Ywhaz  Coro1b  Rab4b  Spast  Ankrd13a  Trak1  Spag9  Enpp3  Fkbp4  Mgat1  Xiap  Ccar1 | web link |
| nuclear body | Gene Ontology | GO:0016604 | 22 (2.73%) | 318 (1.52%) | 0.00597192329646 | 0.037403098541 | Ube2i  Nup98  Retreg1  Raph1  Mrpl36  Banp  Hmbox1  Gemin7  Lmna  Ubox5  Srrt  Inca1  Epb41  Med7  Abl1  Nudt9  Ints7  Myo1c  Snrpa1  Parp3  Pcnp  Ncor2 | web link |
| intracellular membrane-bounded organelle | Gene Ontology | GO:0043231 | 57 (7.08%) | 1061 (5.07%) | 0.00683197075065 | 0.0406502259664 | Dram2  Ugt1a6b  Fmo5  Acox2  Ppp5c  Calu  Ifi30  Zdhhc20  Uba5  Cyp2c40  Ugt2b36  Clu  Srebf1  Mtmr2  Ces1f  Foxk2  Pmf1  Ctsa  Nup98  Cyp4a31  Comt  Epc1  Usf2  Mgst1  Gak  Smarca2  Cyp2r1  Ybx1  Ubap1  Dpagt1  Bag6  Arap1  Cyp3a59  Nisch  Hax1  Srl  P4ha1  Ugt1a7c  Tmem208  Blvrb  Hsd17b2  D130043K22Rik  Hsd17b6  Tm7sf2  Cyp2d10  Cttn  Sc5d  Ugt3a2  Dnajc5  Crot  Cyp4b1  Glrx2  Rragc  Osbpl9  Rhobtb1  Golph3  Pnpla8 | web link |
| mitochondrial membrane | Gene Ontology | GO:0031966 | 14 (1.74%) | 174 (0.83%) | 0.00743911746393 | 0.0421549989623 | Bcl2l1  Card19  Slc25a44  Gm20498  Mff  Trak1  Higd1a  Taz  Tmbim6  Haao  Opa1  Clu  Pnpla8  Atxn3 | web link |
| endosome | Gene Ontology | GO:0005768 | 38 (4.72%) | 662 (3.16%) | 0.0093801087934 | 0.0491278618353 | Znrf1  Dop1b  2610528J11Rik  Entr1  Arhgap32  Mon2  Arpc5  Trim3  Mtmr4  Bin1  Mtmr2  Ankrd13a  Nipa2  Golph3  Ube2d3  Rin3  Lamtor2  Chmp3  Arrb2  Washc3  Ubap1  Slc66a2  Pip4p2  Nisch  Trf  Osbpl9  Uhrf1bp1l  D130043K22Rik  Hsd17b6  Ociad2  Rab4b  Spast  Snx10  Trak1  Dennd10  Nedd4l  Atp11c  Gfra1 | web link |
| lipid droplet | Gene Ontology | GO:0005811 | 9 (1.12%) | 93 (0.44%) | 0.00949530102698 | 0.0491278618353 | Cyb5r3  Spast  Pnpla2  Dhrs3  BC031181  Ube2g2  Irak1  Sigmar1  Ces1f | web link |
| histone deacetylase complex | Gene Ontology | GO:0000118 | 5 (0.62%) | 37 (0.18%) | 0.0130449138439 | 0.0646810311428 | Hdac10  Ncor1  Tbl1xr1  Mta2  Ncor2 | web link |
| site of double-strand break | Gene Ontology | GO:0035861 | 7 (0.87%) | 67 (0.32%) | 0.0142402556747 | 0.0677836170114 | Arpc5  Parp3  Inip  Lmna  Ddb1  Shld2  Atf2 | web link |
| peroxisome | Gene Ontology | GO:0005777 | 11 (1.37%) | 136 (0.65%) | 0.015959989231 | 0.0729438282685 | Dhrs7b  Uox  Mff  Acox2  Tysnd1  Crot  Acox1  Baat  Paox  Pnpla8  Idh1 | web link |
| cell leading edge | Gene Ontology | GO:0031252 | 7 (0.87%) | 69 (0.33%) | 0.0165502803635 | 0.0729438282685 | Ywhaz  Coro1b  Raph1  Ndel1  Abl1  Phldb2  Bcas3 | web link |
| Golgi cisterna membrane | Gene Ontology | GO:0032580 | 7 (0.87%) | 71 (0.34%) | 0.0191133688341 | 0.0748362437653 | Nagpa  Arap1  St3gal4  Golph3  St6gal1  Nucb1  Iigp1 | web link |
| cell cortex | Gene Ontology | GO:0005938 | 13 (1.61%) | 177 (0.85%) | 0.0194727444088 | 0.0748362437653 | Cttn  Trak1  Hax1  Pard3  Pard6a  Epb41  Csnk1g2  Arhgap32  Hnrnpk  Septin2  Rhobtb1  Fgfr2  Fgf1 | web link |
| ribosome | Gene Ontology | GO:0005840 | 14 (1.74%) | 196 (0.94%) | 0.0195398234985 | 0.0748362437653 | Rpl22l1  Mrpl24  Rps10  Mrpl30  Rps19  Mrpl36  Rpl35a  Rpl15  Uba52  Chchd1  Mrpl47  Kxd1  Mrpl48  Rpl18 | web link |
| integral component of Golgi membrane | Gene Ontology | GO:0030173 | 6 (0.75%) | 56 (0.27%) | 0.0201240319369 | 0.0748362437653 | Bet1  Slc35b3  Yif1b  Zdhhc20  Rer1  Unc50 | web link |
| transcription repressor complex | Gene Ontology | GO:0017053 | 6 (0.75%) | 56 (0.27%) | 0.0201240319369 | 0.0748362437653 | Tbl1xr1  Mier1  Zfpm1  N4bp2l2  Ncor2  Ncor1 | web link |
| mitotic spindle | Gene Ontology | GO:0072686 | 9 (1.12%) | 109 (0.52%) | 0.024641001752 | 0.0836617983769 | Nudcd2  Tbl1xr1  Cep170  Taf1d  Epb41  Pkp4  Pkhd1  Atat1  Ncor1 | web link |
| mitochondrial large ribosomal subunit | Gene Ontology | GO:0005762 | 6 (0.75%) | 59 (0.28%) | 0.0253776551278 | 0.0836617983769 | Mrpl36  Mrpl24  Mrpl30  Mrpl47  Mpv17l2  Mrpl48 | web link |
| spindle | Gene Ontology | GO:0005819 | 14 (1.74%) | 203 (0.97%) | 0.0255071339099 | 0.0836617983769 | Pard3  Spast  Cep170  Sbds  Anapc5  Cenpv  Mapk15  Septin2  Pkp4  Ndel1  Nde1  Pkhd1  Atat1  Dido1 | web link |
| actin cytoskeleton | Gene Ontology | GO:0015629 | 15 (1.86%) | 223 (1.06%) | 0.0259172441451 | 0.0836617983769 | Pdlim5  Aldoa  Myo1c  Hax1  Arhgap32  Arpc5  Epb41  Mprip  Septin2  Ablim2  Abl1  Septin9  Lcp1  Cdc42ep4  Zyx | web link |
| intercellular bridge | Gene Ontology | GO:0045171 | 7 (0.87%) | 76 (0.36%) | 0.0267084946452 | 0.0836617983769 | Nudcd2  Nisch  Gstm4  Parp3  Septin2  Epb41  Dusp11 | web link |
| condensed chromosome kinetochore | Gene Ontology | GO:0000777 | 8 (0.99%) | 93 (0.44%) | 0.0267155322548 | 0.0836617983769 | Nudcd2  Csnk1a1  Pmf1  Cenpx  Septin2  Ndel1  Nde1  Cenpv | web link |
| integral component of endoplasmic reticulum membrane | Gene Ontology | GO:0030176 | 9 (1.12%) | 113 (0.54%) | 0.0302063279279 | 0.0911395948889 | Tm7sf2  Ktn1  Dnajb12  Asph  Retreg1  Derl1  Slc35b3  Elovl5  Derl2 | web link |
| respirasome | Gene Ontology | GO:0070469 | 6 (0.75%) | 62 (0.30%) | 0.0314727276644 | 0.0911395948889 | Ndufa5  Uqcr11  Higd1a  Ndufb2  Ndufs2  Ndufs6 | web link |
| microtubule organizing center | Gene Ontology | GO:0005815 | 21 (2.61%) | 353 (1.69%) | 0.0325986693747 | 0.0911395948889 | Nudcd2  Bcl2l1  Uxt  Zfyve19  Exoc7  Spast  Snx10  Pcm1  Tacc2  Cep43  Slmap  Entr1  Tcp1  Pard6a  Csnk1a1  Ndel1  Nde1  Cep170  Npm1  Ppp4r3a  Rabgap1 | web link |
| endoplasmic reticulum lumen | Gene Ontology | GO:0005788 | 9 (1.12%) | 115 (0.55%) | 0.0332942702423 | 0.0911395948889 | Entpd5  Ces3a  Calu  Fkbp7  Manf  Gm49673  P4ha1  Rnaset2b  Uggt1 | web link |
| chromatin | Gene Ontology | GO:0000785 | 20 (2.48%) | 333 (1.59%) | 0.0334149778996 | 0.0911395948889 | Rcc1  Uxt  Hsf4  Stat1  Esr1  Nap1l4  Akap8  Dffa  Tcf7l2  Akap8l  Hnrnpk  Camta2  Hnrnpa2b1  Mbd3  Macroh2a1  Smarca2  Atf2  Ncor2  Ncor1  Nr1h3 | web link |
| SCF ubiquitin ligase complex | Gene Ontology | GO:0019005 | 6 (0.75%) | 63 (0.30%) | 0.0336986737404 | 0.0911395948889 | Usp47  Btrc  Fbxl12  Fbxo6  Amn1  Fbxw5 | web link |
| stress fiber | Gene Ontology | GO:0001725 | 7 (0.87%) | 82 (0.39%) | 0.0382527212453 | 0.101157196182 | Pdlim5  Actn1  Coro1b  Mprip  Septin9  Lcp1  Zyx | web link |
| spliceosomal complex | Gene Ontology | GO:0005681 | 12 (1.49%) | 178 (0.85%) | 0.0425577968485 | 0.110095170108 | Srek1  Rbm3  U2af1  Wac  Snrpa1  Hnrnpk  Ppil3  Hnrnpm  Txnl4a  Ptbp2  Snrnp70  Hnrnpa2b1 | web link |
| nuclear matrix | Gene Ontology | GO:0016363 | 8 (0.99%) | 105 (0.50%) | 0.04937319907 | 0.121142578667 | Akap8  Akap8l  Hnrnpm  Anp32a  Lmna  Npm1  Ncor2  Hnrnpa2b1 | web link |
| chromosome, centromeric region | Gene Ontology | GO:0000775 | 10 (1.24%) | 143 (0.68%) | 0.0495217687291 | 0.121142578667 | Nudcd2  Csnk1a1  Pmf1  Ndel1  Septin2  Cenpx  Nde1  Pkhd1  Cenpv  Ppp2r5c | web link |
| kinetochore | Gene Ontology | GO:0000776 | 9 (1.12%) | 124 (0.59%) | 0.0498822382744 | 0.121142578667 | Nudcd2  Csnk1a1  Pmf1  Nup98  Ndel1  Septin2  Cenpx  Nde1  Cenpv | web link |
| extracellular exosome | Gene Ontology | GO:0070062 | 7 (0.87%) | 89 (0.43%) | 0.0553526291156 | 0.131739257295 | Bag6  Fn1  Cd47  Hnrnpa2b1  Aldoa  Pkhd1  Ybx1 | web link |
| early endosome | Gene Ontology | GO:0005769 | 16 (1.99%) | 274 (1.31%) | 0.0646850750085 | 0.150931841687 | Washc3  Lipc  Entr1  Nisch  Nipa2  Trak1  2610528J11Rik  Rin3  Trf  Derl1  Derl2  Uhrf1bp1l  Chmp3  Trim3  Nucb1  D130043K22Rik | web link |
| nucleolus | Gene Ontology | GO:0005730 | 40 (4.97%) | 813 (3.88%) | 0.0664037468363 | 0.151962420645 | Taf1b  Taf1d  Myo1c  Orc4  Retreg1  Abhd14b  Apex1  Tyms  Letmd1  Cdk8  Rpl18  Kdm5a  Rps6ka3  Rrp36  Pnkp  Sbds  Npm1  Rexo5  Gzf1  Riox2  S100a16  Rps10  Stat1  Zbtb14  Rcl1  Rps19  Akap8  Upf3b  Abl1  Atxn3  Fgf1  Aen  Exosc10  Cutc  Parp3  Nolc1  Ddx56  Dedd  Macroh2a1  Zcchc7 | web link |
| clathrin-coated pit | Gene Ontology | GO:0005905 | 5 (0.62%) | 61 (0.29%) | 0.0848769886546 | 0.185319552543 | Bcl2l1  Arrb2  Cttn  Atat1  Trf | web link |
| Z disc | Gene Ontology | GO:0030018 | 9 (1.12%) | 138 (0.66%) | 0.0850897071554 | 0.185319552543 | Pdlim5  Actn1  Stk11  Neb  Dnajb6  Slmap  Aldoa  Sorbs2  Bin1 | web link |
| trans-Golgi network | Gene Ontology | GO:0005802 | 12 (1.49%) | 200 (0.96%) | 0.0856518940326 | 0.185319552543 | Gba  Arap1  Slc66a2  Smpd4  Dop1b  Golph3  Ccdc91  Golga1  Atp11c  Relch  Nucb1  Pam | web link |
| mitochondrial matrix | Gene Ontology | GO:0005759 | 13 (1.61%) | 224 (1.07%) | 0.0924736159396 | 0.196506433872 | Bcl2l1  Mettl17  Alas1  Mthfs  Cbr4  Glrx2  Mccc2  Tyms  Trmt5  Atg4d  Polrmt  Etfbl  Atxn3 | web link |
| extrinsic component of membrane | Gene Ontology | GO:0019898 | 5 (0.62%) | 63 (0.30%) | 0.0943773401254 | 0.197033394297 | Mgll  Gba2  Gfra1  Ghr  Vps13d | web link |
| focal adhesion | Gene Ontology | GO:0005925 | 11 (1.37%) | 184 (0.88%) | 0.0986927746278 | 0.202490347943 | Ubox5  Actn1  Cttn  Pip5k1c  Itgav  Tns3  Phldb2  Sorbs2  Atat1  Lcp1  Zyx | web link |
| centriolar satellite | Gene Ontology | GO:0034451 | 7 (0.87%) | 103 (0.49%) | 0.101907215774 | 0.205541672494 | Exoc7  Spag9  Pcm1  Taf1d  Pard6a  Upf3b  Rbm39 | web link |
| midbody | Gene Ontology | GO:0030496 | 10 (1.24%) | 166 (0.79%) | 0.107635149403 | 0.213476379649 | Zfyve19  Spast  Safb  Uhmk1  Cpeb3  Entr1  Septin2  Pkp4  Chmp3  Cenpv | web link |
| late endosome | Gene Ontology | GO:0005770 | 9 (1.12%) | 149 (0.71%) | 0.121056820059 | 0.235868948626 | Lipc  Ankrd13a  Dennd10  Kidins220  Trf  Derl1  Derl2  Chmp3  Lamtor2 | web link |
| cytoplasmic vesicle | Gene Ontology | GO:0031410 | 37 (4.60%) | 789 (3.77%) | 0.123752980887 | 0.235868948626 | Trappc8  Znrf1  Snap23  Synrg  Tm9sf1  Clu  Pam  Bcl2l1  Copa  Arap1  Golga1  Man1a  Rin3  Mpdz  Mapk15  Sec24c  Sec24b  Chmp3  Fgfr2  Sigmar1  Sec23b  Pip4p2  Hyal1  Hax1  Trf  Rcbtb2  Tbc1d17  Spast  Mff  Dnajc5  Trak1  Myo1c  Srebf1  Arrb2  Rhobtb1  D130043K22Rik  Sec31b | web link |
| Golgi membrane | Gene Ontology | GO:0000139 | 24 (2.98%) | 485 (2.32%) | 0.124871796331 | 0.235868948626 | Man1a  Dop1b  Arhgap32  Naa60  Copa  Smpd4  Huwe1  Yif1b  Apoo  Unc50  Lman2l  St3gal5  St6gal1  Slc9a8  Map4k2  Rer1  Zdhhc20  Bet1  Paqr3  Slc35b3  Mgat1  Srebf1  Gba2  Pgap2 | web link |
| adherens junction | Gene Ontology | GO:0005912 | 9 (1.12%) | 151 (0.72%) | 0.12836449514 | 0.23867773315 | Pdlim5  Pard3  Snap23  Pip5k1c  Cdc42ep4  Nectin3  Pkp4  Vegfa  Zyx | web link |
| centrosome | Gene Ontology | GO:0005813 | 25 (3.11%) | 522 (2.49%) | 0.153391006074 | 0.280823534196 | Pcm1  Apex1  Entr1  Nde1  Pkhd1  Npm1  Mapkapk2  Bcl2l1  Csnk1a1  Hmbox1  Pard6a  Tcp1  Ndel1  Camsap2  Azin1  Cep43  Gm20695  Uxt  Zfyve19  Cep170  Spast  Macroh2a1  Snx10  Prkar1a  Eya3 | web link |
| transcription regulator complex | Gene Ontology | GO:0005667 | 13 (1.61%) | 257 (1.23%) | 0.192120199679 | 0.339935972287 | Dhrs7b  Mlxipl  Zfpm1  Hax1  Yap1  Tcf7l2  Apex1  Eya3  Crem  Mta2  Riox2  Tfdp1  Med7 | web link |
| cytoplasmic stress granule | Gene Ontology | GO:0010494 | 5 (0.62%) | 80 (0.38%) | 0.194103426593 | 0.339935972287 | Prrc2c  Hnrnpk  Ybx1  Tial1  Mcrip2 | web link |
| postsynaptic density | Gene Ontology | GO:0014069 | 16 (1.99%) | 327 (1.56%) | 0.194249127021 | 0.339935972287 | Ywhaz  Pdlim5  Sorbs2  Pip5k1c  Rps19  Mtmr2  Mpdz  Cpeb3  Lzts3  Arhgap32  Hnrnpk  Arrb2  Pkp4  Pcbp2  Epb41  Sigmar1 | web link |
| microtubule cytoskeleton | Gene Ontology | GO:0015630 | 10 (1.24%) | 190 (0.91%) | 0.197116004909 | 0.339953689626 | Nudcd2  Spast  Camsap2  Nisch  Cdc42ep4  Ss18  Septin2  Cenpv  Septin9  Rbm39 | web link |
| lysosomal membrane | Gene Ontology | GO:0005765 | 11 (1.37%) | 217 (1.04%) | 0.215026468298 | 0.365544996106 | Dram2  Spag9  Pip4p2  Gba  Atraid  Slc39a8  Tm9sf1  Ctsa  Kxd1  Abcd4  Lamtor2 | web link |
| cytoskeleton | Gene Ontology | GO:0005856 | 55 (6.83%) | 1285 (6.14%) | 0.22003974905 | 0.368799016014 | Nudcd2  Actn1  Pard3  Pcm1  Coro1b  Entr1  Pard6a  Cenpv  Nde1  Clu  Lcp1  Bin1  Lzts3  Bcl2l1  Slmap  Sorbs2  Csnk1a1  Sbds  Atat1  Arpc5  Mapk15  Tcp1  Zyx  Ndel1  Zfyve19  Bcas3  Fgd6  Camsap2  Tacc2  Tctn1  Rassf5  Cep43  Cdc42ep4  Mprip  Septin2  Pkp4  S100a9  Abl1  Septin9  Epb41  Kif21a  Ppp4r3a  Dido1  Pkhd1  Anapc5  Uxt  Cttn  Cep170  Spast  Snx10  Rabgap1  Tec  Fkbp4  Rhobtb1  Npm1 | web link |
| lysosome | Gene Ontology | GO:0005764 | 19 (2.36%) | 411 (1.96%) | 0.235991968305 | 0.389655963044 | Acp5  Dram2  Znrf1  Rragc  Spag9  Ctbs  Pip4p2  Hyal1  Gba  Ifi30  Slc39a8  Kxd1  Tm9sf1  Slc17a2  Ctsa  Gm49673  Rnaset2b  Abcd4  Lamtor2 | web link |
| growth cone | Gene Ontology | GO:0030426 | 9 (1.12%) | 177 (0.85%) | 0.242155932016 | 0.389655963044 | Copa  Arpc5  Tiam2  Cttn  Ptbp2  Abl1  Clu  Ndrg2  Sigmar1 | web link |
| ruffle | Gene Ontology | GO:0001726 | 6 (0.75%) | 109 (0.52%) | 0.242307069456 | 0.389655963044 | Actn1  Mtmr14  Myo1c  Cttn  Abl1  Lcp1 | web link |
| collagen-containing extracellular matrix | Gene Ontology | GO:0062023 | 14 (1.74%) | 296 (1.41%) | 0.25066873069 | 0.397727719361 | Plxnb2  Dcn  Ltbp3  Bmp1  Fn1  Serpinb6a  Lgals9  Angptl4  Gpc4  Hnrnpm  Clu  Fgfr2  Itih4  Cela1 | web link |
| ruffle membrane | Gene Ontology | GO:0032587 | 5 (0.62%) | 89 (0.43%) | 0.257429033099 | 0.403079670247 | Pip5k1c  Lcp1  Myo1c  Plekha1  Itgav | web link |
| catalytic step 2 spliceosome | Gene Ontology | GO:0071013 | 5 (0.62%) | 90 (0.43%) | 0.26476313803 | 0.409179395137 | Ppil3  Snrpa1  Hnrnpk  Hnrnpm  Hnrnpa2b1 | web link |
| fibrillar center | Gene Ontology | GO:0001650 | 7 (0.87%) | 139 (0.66%) | 0.287231587554 | 0.438212293833 | Ube2i  Chchd1  Eef1d  Arhgap32  Mri1  Dusp11  Nolc1 | web link |
| dendrite | Gene Ontology | GO:0030425 | 25 (3.11%) | 577 (2.76%) | 0.297243364303 | 0.44774633357 | Ube2i  Rbm3  Nectin3  Cpeb3  Opa1  Trim3  Bin1  Mtmr2  Comt  Rin3  Lzts3  Atxn10  Ybx1  Nap1l4  Abl1  Tmem185a  Sorbs2  Kif21a  Mpdz  Cttn  Clcn2  Trak1  Glrx2  Elovl5  Eif5a | web link |
| synaptic vesicle | Gene Ontology | GO:0008021 | 8 (0.99%) | 167 (0.80%) | 0.313573516281 | 0.4613180737 | Dbi  Slc40a1  Mff  Dnajc5  Septin2  Ndel1  Bin1  Mtmr2 | web link |
| basolateral plasma membrane | Gene Ontology | GO:0016323 | 11 (1.37%) | 239 (1.14%) | 0.314006419914 | 0.4613180737 | Map4k2  Slc40a1  Gm20498  Erbin  Epb41  Slc39a8  Slco1b2  Arrb2  Slc4a2  Slc2a9  Slc38a3 | web link |
| ubiquitin ligase complex | Gene Ontology | GO:0000151 | 5 (0.62%) | 98 (0.47%) | 0.324822037901 | 0.471388079393 | Ankib1  Cdk8  Fbxl12  Med7  Dcun1d1 | web link |
| endomembrane system | Gene Ontology | GO:0012505 | 7 (0.87%) | 146 (0.70%) | 0.330320136463 | 0.473591520953 | Pard3  Rnf167  Sec23b  Pip5k1c  Cdc42ep4  Cnmd  Rab4b | web link |
| dendritic spine | Gene Ontology | GO:0043197 | 9 (1.12%) | 195 (0.93%) | 0.335848017631 | 0.475784691644 | Actn1  Cttn  Zmynd8  Comt  Mtmr2  Arhgap32  Hnrnpk  Arrb2  Lzts3 | web link |
| vesicle | Gene Ontology | GO:0031982 | 7 (0.87%) | 156 (0.75%) | 0.393228325429 | 0.550519655601 | Bmp1  Myo1c  Rin3  Trf  Vegfa  Gak  Bin1 | web link |
| apical plasma membrane | Gene Ontology | GO:0016324 | 16 (1.99%) | 383 (1.83%) | 0.402830271073 | 0.557404677415 | Fn1  Pard3  Slc2a9  Slc38a3  Fxyd1  Hax1  Enpp3  Pard6a  Trf  Slc39a8  Dram2  Slc4a2  Pkhd1  Cyp4a31  Sorbs2  Mpdz | web link |
| nuclear envelope | Gene Ontology | GO:0005635 | 8 (0.99%) | 186 (0.89%) | 0.42424009446 | 0.568187954469 | Ube2i  Nup98  Atraid  Smpd4  Ndel1  Lmna  Bin1  Sigmar1 | web link |
| recycling endosome | Gene Ontology | GO:0055037 | 6 (0.75%) | 136 (0.65%) | 0.424947293679 | 0.568187954469 | Rab4b  Nisch  2610528J11Rik  Trf  Entr1  Atp11c | web link |
| early endosome membrane | Gene Ontology | GO:0031901 | 6 (0.75%) | 136 (0.65%) | 0.424947293679 | 0.568187954469 | Dop1b  Mtmr2  Atp11c  Mon2  D130043K22Rik  Hsd17b6 | web link |
| chromosome, telomeric region | Gene Ontology | GO:0000781 | 5 (0.62%) | 112 (0.53%) | 0.431977995524 | 0.571170905192 | Orc4  Dna2  Thoc7  Hnrnpa2b1  Hmbox1 | web link |
| cell junction | Gene Ontology | GO:0030054 | 35 (4.35%) | 883 (4.22%) | 0.450708621919 | 0.589388197895 | Pdlim5  Actn1  Pard3  Znrf1  Snap23  Ifi30  Nectin3  Arhgap32  Hnrnpk  Itgav  Lcp1  Atat1  Lzts3  Bcl2l1  Pard6a  Pip5k1c  Cpeb3  Sigmar1  Mapk15  Gtf2a2  Tns3  Gm20498  Epb41  Fermt3  Mpst  Pkp4  S100a9  Sorbs2  Mpdz  Zyx  Nudt9  Cttn  Mff  Erbin  Ddb2 | web link |
| receptor complex | Gene Ontology | GO:0043235 | 8 (0.99%) | 200 (0.96%) | 0.505263155521 | 0.653546907685 | Acvr2b  Tm7sf2  Ghr  Prlr  Gfra1  Lifr  Gprc5c  Nr1h3 | web link |
| microtubule | Gene Ontology | GO:0005874 | 13 (1.61%) | 342 (1.63%) | 0.55655686471 | 0.712153407532 | Atat1  Cep170  Spast  Camsap2  Kif21a  Rassf5  Fkbp4  Tcp1  Ndel1  Nde1  Septin9  Bin1  Cdk2ap2 | web link |
| postsynapse | Gene Ontology | GO:0098794 | 6 (0.75%) | 162 (0.77%) | 0.59467180586 | 0.752829201035 | Pdlim5  Snap23  Erbin  Cpeb3  Hnrnpk  Trim3 | web link |
| sarcolemma | Gene Ontology | GO:0042383 | 5 (0.62%) | 138 (0.66%) | 0.616270923727 | 0.771960420247 | Slmap  Smpd4  Slc8b1  Acp1  Fxyd1 | web link |
| ciliary basal body | Gene Ontology | GO:0036064 | 6 (0.75%) | 167 (0.80%) | 0.624234791393 | 0.773791043497 | Csnk1a1  Entr1  Camsap2  Pcm1  Mapk15  Pkhd1 | web link |
| presynapse | Gene Ontology | GO:0098793 | 6 (0.75%) | 169 (0.81%) | 0.635707184426 | 0.779888195327 | Bcl2l1  Pdlim5  Snap23  Dnajc5  4932438A13Rik  Cln8 | web link |
| neuronal cell body | Gene Ontology | GO:0043025 | 21 (2.61%) | 581 (2.77%) | 0.646062116593 | 0.780107353286 | Smurf1  Gtf2i  Pard3  Ghr  Eif5a  Ppp5c  Nap1l4  Rin3  Fkbp4  Atxn10  Pard6a  Elovl5  Gfra1  Glrx2  Ptbp2  Abl1  Nmnat3  Sorbs2  Ybx1  Pam  Hnrnpa2b1 | web link |
| perikaryon | Gene Ontology | GO:0043204 | 7 (0.87%) | 200 (0.96%) | 0.652606909912 | 0.780107353286 | Esr1  Ppp5c  Tiam2  Hnrnpa2b1  Clcn2  Mapk9  Pam | web link |
| chromosome | Gene Ontology | GO:0005694 | 20 (2.48%) | 557 (2.66%) | 0.655552397719 | 0.780107353286 | Nudcd2  Rcc1  Hmgb3  Ints7  Hmces  Pmf1  Macroh2a1  Kmt5b  Hmbox1  Cenpx  Nsd1  Septin2  Ndel1  Mbd3  Cenpv  Nde1  Pkhd1  Ppp2r5c  Shld2  Csnk1a1 | web link |
| synapse | Gene Ontology | GO:0045202 | 29 (3.60%) | 814 (3.89%) | 0.691465411453 | 0.814696870919 | Ube2i  Pdlim5  Znrf1  Snap23  Nectin3  Arhgap32  Gpc4  Nde1  Clu  Bin1  Lzts3  Bcl2l1  Rpl18  Cpeb3  Sigmar1  Uba52  Mpst  Rps19  Slc12a7  Septin2  Abl1  Sorbs2  Mpdz  Cttn  Mff  Mgll  Tiam2  Prkar1a  Eif5a | web link |
| myelin sheath | Gene Ontology | GO:0043209 | 6 (0.75%) | 184 (0.88%) | 0.714851230882 | 0.833993102696 | Tcp1  Septin2  Uba52  Ubc  Aldoa  Immt | web link |
| lamellipodium | Gene Ontology | GO:0030027 | 6 (0.75%) | 188 (0.90%) | 0.73382319195 | 0.847815144098 | Cttn  Hax1  Arpc5  Coro1b  Tiam2  Sorbs2 | web link |
| axon | Gene Ontology | GO:0030424 | 16 (1.99%) | 475 (2.27%) | 0.741056578415 | 0.847939738764 | Smurf1  Atat1  Copa  Bin1  Mgll  Comt  Rin3  Uhmk1  Nectin3  Ulk1  Gfra1  Ndel1  Nmnat3  Kif21a  Mtmr2  Nrp2 | web link |
| cell projection | Gene Ontology | GO:0042995 | 37 (4.60%) | 1062 (5.07%) | 0.757336253029 | 0.8583144201 | Pdlim5  Actn1  Rbm3  Fam149b  Pcm1  Entr1  Arhgap32  Hnrnpk  Mtmr2  Arpc5  Pkhd1  Trim3  Atat1  Lcp1  Sorbs2  Csnk1a1  Pip5k1c  Cpeb3  Mpdz  Mapk15  Ndrg2  Camsap2  Tctn1  Cep43  Fermt3  Septin2  Tmem185a  Gtf2i  Kif21a  Sigmar1  Gm20695  Cttn  Myo1c  Tiam2  Elovl5  Rhobtb1  Lzts3 | web link |
| neuron projection | Gene Ontology | GO:0043005 | 17 (2.11%) | 512 (2.45%) | 0.765959109366 | 0.859897490703 | Ralbp1  Gm20498  Esr1  Ppp5c  Snap23  Tiam2  Arpc5  Pam  Fkbp4  Mpst  Hnrnpa2b1  Sgk1  Abl1  Clu  Mapk9  Mpdz  Cpeb3 | web link |
| cell-cell junction | Gene Ontology | GO:0005911 | 6 (0.75%) | 208 (0.99%) | 0.815174761017 | 0.906596229542 | Actn1  Pard3  Mapk15  Epb41  Nectin3  Pkp4 | web link |
| extracellular region | Gene Ontology | GO:0005576 | 63 (7.83%) | 1829 (8.73%) | 0.840179351907 | 0.925753174787 | Entpd5  Fn1  Gsdmd  C4bp  Clu  Calu  Tfpi2  Thpo  Lgals9  Angptl4  Vegfa  Igfbp4  Igfbp5  Pkhd1  Gm44805  Proc  Pam  Dcn  Copa  Ltbp3  S100a9  Hp  Mup9  Albfm1  Lifr  Apoo  Ybx1  Cela1  Rspry1  Bag6  Ghr  St6gal1  Hyal1  Tctn1  Apoc3  Trf  Enox2  Rbp4  Ifi30  Manf  Mcfd2  Gm49673  Mup2  Rnaset2b  Fgf1  Lipc  Wfdc2  Serpina3m  Bmp1  Il18bp  Selenop  Enpp2  Enpp3  Cnmd  Chrd  Fetub  AI182371  Gpc4  Smoc1  Cp  Nucb1  Itih4  Hnrnpa2b1 | web link |
| glutamatergic synapse | Gene Ontology | GO:0098978 | 14 (1.74%) | 473 (2.26%) | 0.874259737112 | 0.954467052443 | Ywhaz  Actn1  Cttn  Snap23  Pip5k1c  Erbin  Hnrnpk  Gpc4  Hnrnpa2b1  Ndrg2  Bin1  Trim3  Nrp2  Prkar1a | web link |
| endosome membrane | Gene Ontology | GO:0010008 | 5 (0.62%) | 206 (0.98%) | 0.901329145701 | 0.975074257622 | Pip5k1c  Ube2d3  Arhgap32  Mtmr2  Snx10 | web link |
| cell surface | Gene Ontology | GO:0009986 | 19 (2.36%) | 655 (3.13%) | 0.921032058238 | 0.98741274712 | Smim1  Ghr  Gm20498  Prlr  Cd47  H13  Lipc  Itgav  Trf  Rer1  Atp5j  Septin2  Vegfa  Hnrnpm  Gpc4  Clu  Fam234a  Fgfr2  Pam | web link |
| extracellular space | Gene Ontology | GO:0005615 | 55 (6.83%) | 1732 (8.27%) | 0.94575099761 | 0.999999999999 | Fn1  Gsdmd  Tfpi2  Thpo  Gm49673  Angptl4  Gpc4  Igfbp4  Igfbp5  Clu  Gm44805  Proc  S100a16  Ces1f  Dcn  Copa  Hp  Tmprss6  Mup9  Ces3a  Albfm1  Il18bp  Cela1  Rbp4  Ghr  Gba  Pam  Hyal1  Tctn1  Trf  Enox2  S100a9  Manf  Aldoa  Rnaset2b  Fgf1  Sema4g  Dbi  Lipc  Wfdc2  Serpina3m  Serpina3n  Selenop  Enpp2  Serpinb6a  Slmap  Atp5j  Chrd  Fetub  Gfra1  Vegfa  Cp  Nucb1  Itih4  Bmp1 | web link |
| membrane | Gene Ontology | GO:0016020 | 301 (37.39%) | 8771 (41.89%) | 0.996391867818 | 0.999999999999 | Pdlim5  Smim1  Slc39a11  Cers2  Slc8b1  Ktn1  Apoo  Adck5  Iigp1  Gm49673  Tomm5  Mon2  Slc66a2  Opa1  Tmbim6  Srebf1  Arv1  S100a9  Retreg1  Card19  Tm7sf2  Sgk1  Esr1  Pip5k1c  Tmem256  Tmem208  Kidins220  Inpp4a  Slc39a8  Slc39a9  Rhbdf2  Pigv  Dop1b  Chmp3  Yif1b  Unc50  Cyp2r1  Ggnbp1  Aox3  Gna13  Dhrs3  Ptdss1  Bri3bp  Sec24c  Slc2a9  Timd4  Atg16l1  Unc45a  Nipsnap1  Spag9  Septin2  Wbp1l  Uba5  Gngt1  Zdhhc20  Hsd17b2  Mrpl48  Sigmar1  Hsd17b6  Pkhd1  Arhgef12  Cttn  Spast  Snx10  Dnajc5  Extl2  Enpp2  Cyp4b1  Taz  Slmap  Atp5j  Atp11c  Lpcat3  Igsf5  Kxd1  Gpc4  Trf  Nnt  Gk  Pgap2  Gm5150  Pcnx3  Actn1  Dram2  Ugt1a6b  Ralbp1  Ppp5c  Synrg  Asph  Ccdc91  Osbpl9  Enpp3  Sc5d  Slc35b3  Slco1b2  Plekha1  Cnot2  Irak1  Atat1  Immt  Gprc5c  Yap1  Bcl2l1  Gramd3  Gm20498  Mthfs  Mgat1  Cnmd  Uqcr11  Mgst1  4932438A13Rik  Cyp4a31  Ube2d3  Itgav  Golga1  Agap3  Lifr  Slc25a19  Sec24b  Fndc3b  Fam234a  Fgfr2  Slc43a1  Ugt1a9  Dhrs7b  Madd  Rdh13  Slc40a1  Ghr  Lrp3  Mettl23  Nisch  Tctn1  Hax1  Srl  AI182371  Pkp4  Tmem185a  Cdc42ep4  Mpdz  Sec61a2  Gm16867  Stk11  Rab4b  Mff  Fxyd1  Aamdc  Tmem258  Slc25a23  Ugt1a7c  Slc41a2  Icmt  Mpv17l2  Slc25a25  Atf2  Gba2  Nucb1  Sec31b  Maob  Entpd5  Ndufa5  Slc25a44  Snap23  Cd300c2  2610528J11Rik  Slc17a2  Atraid  Tmem234  Tyms  Derl2  Vegfa  Pard6a  Ugt2b36  Clu  Mtmr4  Bin1  Mtmr2  Pigp  Epb41  Sorbs2  Map4k2  Asic5  Man1a  Comt  Tmprss6  Dnajb12  Rpl15  Dio1  Dpagt1  Bag6  Exoc7  St3gal5  Gba  Pip4p2  Pomgnt2  Rgs3  Slc9a8  Tmem222  Vkorc1l1  Rnf141  Slc4a2  Ggt6  Ube2g2  Mia2  P2ry2  Phldb2  Rnaset2b  Lamtor2  Sema4g  Smurf1  Gm4951  Bet1  Ankrd13a  Ugt3a2  Clcn2  Paqr3  Naxd  Gsdmd  Rer1  Smpd4  St6gal1  Elovl5  Arrb2  Prkar1a  Tomm22  Golph3  Cox7a2  Cp  Trak1  Cln8  Pard3  Znrf1  Fmo5  Tmem131l  Mgll  Tmem176b  Ero1b  Calu  Lcp1  Slc38a10  Tmem11  Tm9sf1  Cyp2c40  Naa60  Cyb561d1  Arhgap32  Abcd4  Nrp2  Plxnb2  Slc38a4  Slc22a30  Enox2  Rnf167  Sec23b  Slc38a3  Nipa2  St3gal4  Higd1a  Pam  Acvr2b  Ndufs2  Pnkd  Arhgdib  Rhbdd3  Ndufs6  Cyp3a59  Josd2  Lman2l  Pnpla6  Lysmd3  Nagpa  Letmd1  Prlr  Cyp2d10  Ndufb2  Phkb  Derl1  Slc12a7  Copa  Insig2  H13  Susd1  Aldoa  Tmem161b  D130043K22Rik  Nectin3  Banp  Timm17b  Cyb5r3  Gpr155  Cd47  Myo1c  Tiam2  Nomo1  Pnpla2  Gcc2  Gfra1  Arap1  Erbin  Pnpla8  Eif5a | web link |
| cilium | Gene Ontology | GO:0005929 | 5 (0.62%) | 375 (1.79%) | 0.998942764788 | 0.999999999999 | Pkhd1  Septin2  Csnk1a1  Fam149b  Gm20695 | web link |
| external side of plasma membrane | Gene Ontology | GO:0009897 | 8 (0.99%) | 524 (2.50%) | 0.999473134051 | 0.999999999999 | Enox2  Ghr  Prlr  Enpp3  Itgav  Gpc4  Lifr  Gfra1 | web link |
| cellular\_component | Gene Ontology | GO:0005575 | 57 (7.08%) | 2346 (11.20%) | 0.999976172943 | 0.999999999999 | Pcnx3  Gpank1  Zfp825  Plekhg3  Ccdc163  Polr1has  Szrd1  Csad  Cyb561d1  Plekha6  Zfand4  Zfand3  Arl16  Tmem234  Fam135a  Lrp3  Fam204a  Ccdc30  Mup9  Iqcc  Slc39a9  Prxl2c  Ttc13  Cyp3a59  Atxn10  Rnf214  Elmo3  1110008P14Rik  Wsb1  L3hypdh  Mettl9  Prrc2b  Gmppa  9930021J03Rik  Tmem161b  Rnf141  Wbp1l  Manf  Susd1  Tmem222  Sbno2  Gm527  Msantd2  Brd3  Rsrp1  Josd2  Zc3h7a  Aspdh  1110065P20Rik  Haghl  Smim10l1  Pdrg1  Gm826  Wdr20  2700062C07Rik  Mrnip  Btf3l4 | web link |
| integral component of membrane | Gene Ontology | GO:0016021 | 196 (24.35%) | 6684 (31.92%) | 0.999999376614 | 0.999999999999 | Smim1  Slc39a11  Cers2  Slc8b1  Ktn1  Apoo  Adck5  Maob  Tomm5  Mon2  Opa1  Tmbim6  Arv1  Card19  Tm7sf2  Tmem258  Tmem256  Kidins220  Inpp4a  Pigp  Slc39a9  Rhbdf2  Pigv  Unc50  Cyp2r1  Dhrs3  Ptdss1  Bri3bp  Slc2a9  Timd4  Mthfs  Unc45a  Wbp1l  Rer1  Zdhhc20  Hsd17b2  Mrpl48  Sigmar1  Pkhd1  Cyp2d10  Gm5150  Spast  Ugt1a6b  Igsf5  Extl2  Enpp2  Cyp4b1  Taz  Mettl23  Smpd4  St3gal5  Lpcat3  AI182371  Nnt  Pcnx3  Dram2  Asph  Retreg1  Enpp3  Nectin3  Slc35b3  Slco1b2  Cnot2  Immt  Gprc5c  Bcl2l1  Slmap  Gm20498  Gpr155  Mgat1  Cnmd  Uqcr11  Slc41a2  4932438A13Rik  Itgav  Elovl5  Lifr  Slc25a19  Atp11c  Fndc3b  Fam234a  Fgfr2  Slc43a1  Ugt1a9  Dhrs7b  Madd  H13  Slc40a1  Ghr  Lrp3  Tomm22  Ugt1a7c  Gm49673  Tmem185a  Sec61a2  Gm16867  Pip4p2  Mff  Spag9  Aamdc  Slc25a23  Mgst1  Mpv17l2  Slc25a25  Gba2  Entpd5  Aox3  Slc25a44  Cd300c2  2610528J11Rik  Slc17a2  Comt  Derl1  Derl2  Ugt2b36  Slc39a8  Asic5  Man1a  Atraid  Tmprss6  Dnajb12  Naxd  Dpagt1  Slc66a2  St6gal1  Gramd3  Pomgnt2  Slc9a8  Epb41  Ggt6  Ube2g2  Mia2  P2ry2  Cyp4a31  Rnaset2b  Sema4g  Tmem208  Bet1  Aldoa  Sc5d  Ugt3a2  Clcn2  Paqr3  Fxyd1  Gsdmd  Srebf1  Slc4a2  Cox7a2  Cp  Cln8  Nagpa  Fmo5  Tmem131l  Tmem176b  Slc38a10  Vkorc1l1  Tmem11  Tm9sf1  Cyb561d1  Tmem234  Pam  Nrp2  Plxnb2  Slc38a4  Slc22a30  Pgap2  Rnf167  Banp  Slc38a3  Nipa2  St3gal4  Higd1a  Abcd4  Yif1b  Rhbdd3  Cyp3a59  Josd2  Lman2l  Icmt  Lysmd3  Acvr2b  Letmd1  Prlr  Slc12a7  Map4k2  Insig2  Susd1  Tmem222  Tmem161b  D130043K22Rik  Timm17b  Cd47  Nomo1  Pnpla2  Pnpla6  Pnpla8 | web link |
| integral component of plasma membrane | Gene Ontology | GO:0005887 | 16 (1.99%) | 1203 (5.75%) | 0.999999970896 | 0.999999999999 | Plxnb2  Slc38a4  Slc38a3  Ghr  Asic5  Clcn2  Cd47  Enpp2  Slc17a2  Slc39a8  Slc12a7  Slco1b2  Atp11c  Fgfr2  Pam  Sema4g | web link |
| plasma membrane | Gene Ontology | GO:0005886 | 132 (16.40%) | 5547 (26.49%) | 0.999999999999 | 0.999999999999 | Smim1  Slc39a11  Slc8b1  Cpeb3  Cnot2  Cdv3  Sgk1  Esr1  Pip5k1c  Sigmar1  Slc39a8  Rhbdf2  Rgs3  Chmp3  Ggnbp1  Gna13  Slc2a9  Tacc2  Trf  Septin2  Cttn  Rer1  Gngt1  Zdhhc20  Blvrb  Atxn3  Csnk1g2  Gm5150  Dnajc5  Enpp3  Slmap  Smpd4  Atp11c  Rhobtb1  Gpc4  Actn1  Tec  Ralbp1  Ppp5c  Asph  Ubap1  Raph1  Slco1b2  Plekha1  Irak1  Gprc5c  Cdc42ep4  Ube2d3  Itgav  Lifr  Fgfr2  S100a16  Madd  Slc40a1  Ghr  Nisch  H13  Hax1  Comt  Pkp4  Sorbs2  Mpdz  Zyx  Dbi  Rab4b  Fxyd1  Erbin  Nedd4l  Nln  Snap23  Cd300c2  2610528J11Rik  Atraid  Coro1b  Csnk1g3  Zbtb42  Dram2  Vegfa  Pard6a  Bin1  Adk  Asic5  Golph3  Dffa  Tmprss6  Slc41a2  Exoc7  Pip4p2  Epb41  S100a9  P2ry2  Phldb2  Kif21a  Sema4g  Smurf1  Ankrd13a  Clcn2  Flad1  Gsdmd  Arrb2  Prkar1a  Cp  Itih4  Nectin3  Pard3  Tmem131l  Pkhd1  Lcp1  Pam  Nrp2  Plxnb2  Slc38a4  Enox2  Arap1  Slc38a3  Sh3d19  Nipa2  Atxn10  Lysmd3  Acvr2b  Eif5  Phkb  Slc12a7  Map4k2  Aldoa  D130043K22Rik  Cd47  Myo1c  Pnpla2  Rnf114  Ppp6r3  Gfra1 | web link |
