# Supplementary material for: Regulation of Alternative Splicing of Lipid Metabolism Genes in Sepsis-Induced Liver Damage by RNA-Binding Proteins
Source: Inflammation. 2024 May 9;47(6):1952–68. doi: 10.1007/s10753-024-02017-2 (PMC11607010; doi:10.1007/s10753-024-02017-2)
Supplement: Supplementary file 1 — Supplementary file1 (ZIP 38655 KB) [file 10753_2024_2017_MOESM1_ESM.zip › Sup5_AS_GO.zip!/Sup5_AS_GO/ras/CLP_vs_Sham/Ras_GO_analysis/Ras_GO_enrichment_web_F.html]

GO enrichment analyze


# GO enrichment analyze result


| Term | Database | ID | Input number (779) | Background number (20698) | P-Value | Corrected P-Value | Input | Hyperlink |
| --- | --- | --- | --- | --- | --- | --- | --- | --- |
| protein binding | Gene Ontology | GO:0005515 | 327 (41.98%) | 6748 (32.60%) | 1.47423937502e-08 | 1.14410433127e-06 | Pdlim5  Gpank1  Cers2  Sbno2  Aars  Ppfibp1  Ube2d3  Mpdz  Chrd  Hnrnpl  Hnrnpm  Edc4  Opa1  Nde1  Mapkapk5  Srebf1  Anapc5  Kdm5a  Tfdp1  Pan3  Sgk1  Mapk15  Pip5k1c  Ctsa  Cnot10  Crem  Dcaf11  Sigmar1  Iqcc  Rhbdf2  Pnkd  Cdk2ap2  Ccar1  Smarca2  Sema4g  Rbm39  Bcas3  Mta2  Lifr  Kctd17  Ttc13  Arap1  Zbtb14  Atg16l1  Atg16l2  Zbtb18  Unc45a  Nipsnap1  Trf  Nsd1  Tiam2  Pcbp2  Relch  Abl1  Amn1  Mapk9  Atxn3  Fgf1  Arhgef12  Cttn  Pnkp  Cep170  Inca1  Arhgef19  Nolc1  Desi1  Arhgap32  Helz  Slmap  Fbxl12  Zfand4  Rhobtb1  Ddb2  Esr1  Ddb1  Ncor2  Cpsf1  Tent2  Gtf2i  C4bp  Tial1  Ralbp1  Dusp16  Ppp2r2d  Synrg  Rspry1  Neb  Prpf39  Serpinb6a  Nectin3  Lgals9  Slco1b2  Cnpy2  Clu  Ankhd1  Ubxn4  Trim3  Irak1  Immt  Trim7  Yap1  Dcn  Smg7  Rps6ka3  Gm20498  Pmf1  Bin1  Safb  Gemin7  Cpeb3  Mapkapk2  Golga1  Tnip1  Sap130  Gna13  Mbd3  Sec24b  Camta2  Fndc3b  Fam234a  Ybx1  Dhrs7b  Zfp106  Ubap1  Slc40a1  Ghr  Dnajc5  Camsap2  Akap8  Nisch  Tctn1  Sec23b  Uhmk1  Tcf7l2  Myl6  Rnf220  Per1  Pkp4  Rbp4  Cpsf6  Polrmt  Kxd1  Zbtb42  Sorbs2  Ppp4r3a  Zyx  Ywhaz  Kctd2  Lipc  Dna2  Ubc  Mff  Dcun1d1  Erbin  Selenop  Ptbp2  Dnajb6  Fkbp4  Nedd4l  Xiap  Fkbp7  Sfswap  Wsb1  Ubxn2a  Cdk8  Atf2  Smoc1  Sec31b  Eya3  Kidins220  Taf1b  Ehbp1l1  Rwdd3  Snap23  Cd300c2  Pcm1  Wac  Actn1  Dffa  Coro1b  Derl1  Derl2  Zbtb43  Vegfa  Pard6a  Hnrnpk  Ankib1  Vps13d  Mdm4  Fbxw5  Wdr37  Trim11  Lrp3  Zyg11a  Wdr53  Fn1  Septin9  Hmbox1  Mlxipl  Huwe1  Tcp1  Tmprss6  Hdgfl2  Gzf1  Ndel1  Tns3  Lmna  Elmo3  Nr1h3  Emsy  Dmxl1  Zmynd8  Exoc7  Gba  Raph1  Srrt  Pomgnt2  Btrc  9930021J03Rik  Epb41  P4ha1  Ube2g2  Mia2  Kif21a  Gstm4  Lamtor2  Ndufs2  Smurf1  Hax1  Sh3d19  Tbl1xr1  Ankrd13a  U2af1  Zc3h7a  Mtmr2  Tec  Snrpa1  Tef  Ggnbp1  Uggt1  Agap3  Arrb2  Prkar1a  Fbxo6  Tomm22  Bag6  Ube2v2  Clpb  Itih4  Cln8  Ube2i  Trappc8  Pard3  Znrf1  Srsf3  Sorbs1  Asph  Stk11  Uba5  Lcp1  Anapc10  Arhgap12  Igfbp4  Tmem176b  Spag9  Pkhd1  Kctd20  Kctd21  Npm1  Pam  Nrp2  Plxnb2  Cluh  Copa  Banp  Pnpla2  Fam120b  Nipa2  Tsc22d3  Plekha6  Mcrip2  Iigp1  Usf2  Foxk2  Uba52  N4bp2l2  Rhbdd3  Anp32a  Atxn10  Thoc7  Dedd  Gls2  Bcl2l1  Acvr2b  Cflar  Rragc  Prlr  Rassf5  Eif5  Cep43  Cdc42ep4  Fbxo34  Fermt3  Brd8  Rcbtb2  Gtpbp2  Rgs3  Brd3  Chd2  Cyb5r3  Bmp1  Rbm47  Cd47  Zfpm1  Myo1c  Septin2  Gsta2  Son  Rora  Kmt5b  Mast3  Wdr20  Ppp5c  Mapk6  Timd4  Setd4  Ppp2r5c | web link |
| oxidoreductase activity | Gene Ontology | GO:0016491 | 57 (7.32%) | 684 (3.30%) | 1.78766301762e-08 | 1.14410433127e-06 | Oxnad1  Grhpr  Aox3  Fmo5  Acox2  Idh1  Acox1  Asph  Dhrs3  Ifi30  Akr1e1  Apex1  Maob  Fxn  Cyp2c40  Cyb561d1  Bdh2  Kdm5a  Enox2  Hsd17b2  Tm7sf2  Cryz  Paox  Cbr4  Ero1b  Pam  Ndufs2  Cyp2r1  Riox2  Dio1  Dhrs7b  Uox  Jmjd7  Cyp3a59  Cmah  Vkorc1l1  P4ha1  Adhfe1  Blvrb  Cyp4a31  Cox7a2  Hsd17b6  Akr1c20  Aspdh  Cyp2d10  Nnt  Cyb5r3  Sc5d  Cyp4b1  Rtn4ip1  Haao  Rdh13  Aldh16a1  Akr1c6  Cp  Alkbh2  Alkbh3 | web link |
| transferase activity | Gene Ontology | GO:0016740 | 111 (14.25%) | 1771 (8.56%) | 5.52852731373e-08 | 2.35883832052e-06 | Cers2  Zdhhc20  Mapkapk5  Hemk1  Mapkapk2  Nme6  Vcpkmt  Sgk1  Pip5k1c  Pigp  Pigv  Clk4  Ptdss1  Ulk1  Mpst  Mapk6  Fggy  Idnk  Gne  Abl1  Mapk9  Upp2  Papss2  Tyms  Extl2  Pcyt2  Mettl23  Mgat1  Lpcat3  Rnf220  Nmnat3  Gk  Sult1b1  Flad1  Ugt1a6b  Snrk  Tec  Sugct  Ankib1  Irak1  Atat1  Trim7  Rps6ka3  Ube2d3  Naa80  Mapk15  Aasdhppt  Fgfr2  Ugt1a9  Gstm4  Uhmk1  Ugt1a7c  Polrmt  Xylb  Tent2  Rps6kb2  Nedd4l  Xiap  Mgst1  Sult2a8  Cdk8  Gba2  Csnk1g2  Csnk1g3  Ugt2b36  Adk  Uggt1  Trim11  Huwe1  Comt  Agxt2  Mettl17  Dpagt1  St3gal5  St3gal4  Pomgnt2  Ggt6  Ube2g2  Trmt5  Smurf1  Ugt3a2  Pfkfb2  Elovl5  Ube2i  Znrf1  Uckl1  Stk11  Baat  Gsta2  Naa60  Parp3  Map4k2  Rnf167  Csnk1a1  Gba  Kmt5b  St6gal1  Mocs2  Nsd1  Icmt  Ubox5  Acvr2b  Gmppa  Alas1  Pi4ka  Crot  Glrx2  Aprt  Rnf114  Mast3  Setd4 | web link |
| nucleic acid binding | Gene Ontology | GO:0003676 | 47 (6.03%) | 594 (2.87%) | 1.42239807466e-06 | 4.5516738389e-05 | Srek1  Gpank1  Rbm3  Srsf3  Enpp2  Aars  Esrp2  Hnrnpk  Hnrnpl  Hnrnpm  Tial1  Ankhd1  Ptbp3  Ptbp2  Hemk1  Npm1  Dazap1  Enox2  Rnaseh2a  Hnrnpdl  Safb  Cpeb3  Rexo5  Ybx1  Srrt  Hdlbp  Uhmk1  Upf3b  Pcbp2  Hnrnpll  Aen  Exosc10  Ddb1  Rbm47  U2af1  Rbm39  Ddx56  Enpp3  Son  Srsf11  Hnrnpa2b1  Hnrnph3  Cpsf7  Cpsf6  Snrnp70  Zcchc7  Cpsf1 | web link |
| RNA binding | Gene Ontology | GO:0003723 | 60 (7.70%) | 915 (4.42%) | 2.14814951857e-05 | 0.000549926276753 | Srek1  Rbm3  Srsf3  Ppp5c  Aars  Apex1  Rpl15  Hnrnpk  Hnrnpl  Hnrnpm  Tial1  Ankhd1  Ptbp3  Ptbp2  Son  Rpl18  Pan3  Dazap1  Cluh  Rnaseh2a  Hnrnpdl  Cryz  Npm1  Safb  Cpeb3  Rexo5  Sfswap  Unc50  Ybx1  Rpl22l1  Rps10  Zfp106  Rps19  Hdlbp  Uhmk1  Pcbp2  Gm49673  Rnaset2b  Hnrnpll  Exosc10  Mrpl24  Dna2  Esrp2  Hmgb3  Rbm39  Rbm47  Ddx56  Snrpa1  Helz  U2af1  Thoc7  Srsf11  Hnrnpa2b1  Hnrnph3  Dusp11  Cpsf7  Cpsf6  Snrnp70  Cpsf1  Eif5a | web link |
| mitogen-activated protein kinase binding | Gene Ontology | GO:0051019 | 7 (0.90%) | 36 (0.17%) | 0.000334661000544 | 0.00615207548439 | Dusp16  Tnip1  Arrb2  Abl1  Mapkapk5  Gtf2i  Mapkapk2 | web link |
| nuclear hormone receptor binding | Gene Ontology | GO:0035257 | 6 (0.77%) | 26 (0.13%) | 0.000336441628052 | 0.00615207548439 | Stat1  Tacc2  Tcf7l2  Bcas3  Ncor2  Ncor1 | web link |
| mRNA binding | Gene Ontology | GO:0003729 | 19 (2.44%) | 211 (1.02%) | 0.000411172480646 | 0.00657875969034 | Hdlbp  Tyms  Nup98  Ptbp2  Copa  Hnrnpl  Esrp2  Upf3b  Hnrnpk  Pcbp2  Hnrnpm  Rbm47  Ptbp3  Cpsf6  Snrnp70  Ybx1  Hnrnpll  Thoc2l  Luc7l2 | web link |
| identical protein binding | Gene Ontology | GO:0042802 | 92 (11.81%) | 1735 (8.38%) | 0.000490558639243 | 0.00697683398034 | Slc8b1  Tysnd1  Maob  Hnrnpk  Nde1  Esr1  Sigmar1  Chmp3  Kctd17  Atg16l1  Inca1  Mpst  Atxn3  Upp2  Polg2  Gramd3  Tnip1  Hnrnpa2b1  Nudt14  Ssbp1  Fn1  Ppp5c  Idh1  Ccdc91  Nectin3  Angptl4  Trim3  Irak1  Iigp1  Bcl2l1  Stat1  Hp  Dnajb6  Riox2  S100a16  Slc40a1  Ghr  Nisch  Sorbs2  Psmc6  Ywhaz  Lipc  Mff  Spag9  Xiap  Mgst1  Srsf11  Taf1d  Coro1b  Derl1  Hsf2  Vegfa  Bin1  Mtmr2  Kctd20  Hmbox1  Mri1  Lmna  Dbi  Emsy  Bag6  Ndel1  P4ha1  Ube2g2  Desi1  Flad1  Tef  Cbs  Arrb2  Gmeb2  Pard3  Hsf4  Fbp1  Mmaa  Lcp1  Kctd21  Npm1  Gm44805  Nrp2  Banp  Cryz  Pam  Usf2  Atxn10  Acacb  Rps19  Rassf5  Alas1  Gtpbp2  Aldoa  Bmp1  Septin2 | web link |
| hydrolase activity | Gene Ontology | GO:0016787 | 90 (11.55%) | 1696 (8.19%) | 0.000552906465844 | 0.00707720276281 | Tysnd1  Opa1  Mtmr14  Man2c1  Ctsa  Usp47  Usp40  Atg4d  Smarca2  Gne  Gm49673  Atxn3  Exosc10  Nudt9  Ddx56  Enpp3  Helz  Smpd4  Nudt14  Ptrh2  Bpnt1  Ppp5c  Haghl  Hdac10  Iigp1  Ces1f  Nudt13  Ercc5  Kynu  Hmces  Ctbs  H13  Psmc6  Lipc  Dna2  Rab4b  Gba2  Eya3  Nln  Entpd5  Mtmr4  Proc  Mtmr2  Man1a  Tmprss6  Dusp16  Dusp11  Dusp12  Acp5  Acp1  Gba  Hagh  Pip4p2  Enpp2  Rnaset2b  Aen  Bmp1  Desi1  Senp6  Pfkfb2  Ganc  Clpb  Baat  Rnpepl1  Abhd14b  Apex1  Fbp1  Mmaa  Dusp3  Rnaseh2a  Pnkd  Mindy3  Josd2  Dclre1c  Gls2  Cela1  Lyplal1  Nagpa  Jmjd7  Rragc  Hyal1  Ntpcr  Chd2  Ces3a  Mgll  Pnpla2  Pnpla6  Ggt6  Pnpla8  Gpcpd1 | web link |
| histone deacetylase activity | Gene Ontology | GO:0004407 | 5 (0.64%) | 20 (0.10%) | 0.000721363741539 | 0.00753876527364 | Mier1  Mier2  Atxn3  Mta2  Hdac10 | web link |
| catalytic activity | Gene Ontology | GO:0003824 | 29 (3.72%) | 405 (1.96%) | 0.000753493785903 | 0.00753876527364 | Pfkfb2  Rtca  Flad1  Csad  Fbp1  Pam  Man2c1  Ercc5  Agxt2  Mocs1  Echdc1  Hoga1  Kynu  Acacb  Rcl1  Hyal1  Azin1  Alas1  Papss2  Gne  Aldoa  Upp2  Dna2  Spast  Pcyt2  Enpp2  Enpp3  Ganc  Nmnat3 | web link |
| GTPase binding | Gene Ontology | GO:0051020 | 7 (0.90%) | 41 (0.20%) | 0.000765655848104 | 0.00753876527364 | Bcl2l1  Rragc  Mff  Atg16l1  Bin1  Lcp1  Ybx1 | web link |
| ferrous iron binding | Gene Ontology | GO:0008198 | 6 (0.77%) | 32 (0.15%) | 0.00109273348248 | 0.00998528425309 | Acp5  Trf  Fxn  Haao  Alkbh2  Alkbh3 | web link |
| transferase activity, transferring glycosyl groups | Gene Ontology | GO:0016757 | 19 (2.44%) | 230 (1.11%) | 0.00117015049841 | 0.00998528425309 | Upp2  Gba  Dpagt1  St3gal5  Mgat1  Ugt3a2  Pomgnt2  St6gal1  Aprt  Parp3  Ugt1a6b  St3gal4  Ugt1a7c  Ugt1a9  Pigv  Ugt2b36  Gba2  Extl2  Uggt1 | web link |
| UDP-glycosyltransferase activity | Gene Ontology | GO:0008194 | 5 (0.64%) | 23 (0.11%) | 0.00142575728246 | 0.0112787154201 | Ugt2b36  Ugt3a2  Ugt1a7c  Ugt1a9  Ugt1a6b | web link |
| transcription factor binding | Gene Ontology | GO:0008134 | 25 (3.21%) | 349 (1.69%) | 0.00168210547015 | 0.0112787154201 | Ube2i  Hnrnpk  Npm1  Tfdp1  Yap1  Bcl2l1  Esr1  Mlxipl  Gtf2a2  Ybx1  Bcas3  Atf2  Trim11  Arap1  Hyal1  Rora  Per1  Insig2  Tcf7l2  Mapk9  Ywhaz  Nolc1  Zfpm1  Mettl23  Camta2 | web link |
| ubiquitin-protein transferase activity | Gene Ontology | GO:0004842 | 19 (2.44%) | 238 (1.15%) | 0.00174100505352 | 0.0112787154201 | Smurf1  Ubox5  Btrc  Znrf1  Rnf220  Huwe1  Rspry1  Ube2d3  Ankib1  Trim11  Nedd4l  Xiap  Ube2g2  Rnf214  Rnf141  Ddb2  Trim3  Rnf167  Ube2i | web link |
| kinase activity | Gene Ontology | GO:0016301 | 39 (5.01%) | 631 (3.05%) | 0.00175319249689 | 0.0112787154201 | Tec  Uckl1  Snrk  Stk11  Csnk1g2  Csnk1g3  Mapkapk5  Irak1  Ccnh  Adk  Nme6  Rps6ka3  Csnk1a1  Pi4ka  Pip5k1c  Mapkapk2  Mapk15  Clk4  Acvr2b  Ulk1  Akap8  Uhmk1  Map4k2  Mapk6  Fggy  Idnk  Gne  Abl1  Mapk9  Papss2  Sgk1  Fgfr2  Pfkfb2  Rps6kb2  Akap8l  Mast3  Xylb  Cdk8  Gk | web link |
| growth factor binding | Gene Ontology | GO:0019838 | 7 (0.90%) | 47 (0.23%) | 0.00176229928439 | 0.0112787154201 | Acvr2b  Ltbp3  Ghr  Vegfa  Lifr  Igfbp4  Igfbp5 | web link |
| protein N-terminus binding | Gene Ontology | GO:0047485 | 12 (1.54%) | 123 (0.59%) | 0.00234537329658 | 0.0142956086649 | Dcn  Pdlim5  Zmynd8  Tbl1xr1  Acox1  Hax1  Eif3e  Epb41  Clu  Npm1  Ncor2  Eif5a | web link |
| nuclear receptor transcription coactivator activity | Gene Ontology | GO:0030374 | 7 (0.90%) | 52 (0.25%) | 0.00318826803952 | 0.018549923139 | Actn1  Ss18  Brd8  Ccar1  Eny2  Zmiz2  Atxn7l3 | web link |
| 2 iron, 2 sulfur cluster binding | Gene Ontology | GO:0051537 | 5 (0.64%) | 28 (0.14%) | 0.00356522184306 | 0.0198412346049 | Cmah  Aox3  Fxn  Glrx2  Isca2 | web link |
| NADP binding | Gene Ontology | GO:0050661 | 6 (0.77%) | 41 (0.20%) | 0.00407119794146 | 0.0217130556878 | Aspdh  Grhpr  Tm7sf2  Cryz  Fmo5  Idh1 | web link |
| transcription corepressor activity | Gene Ontology | GO:0003714 | 14 (1.80%) | 168 (0.81%) | 0.00457600405903 | 0.0234291407822 | Dhrs7b  Uxt  Zmynd8  Tbl1xr1  Mier1  Mier2  Zfpm1  Riox2  N4bp2l2  Ncor1  Ccar1  Ncor2  Mta2  Yap1 | web link |
| glucuronosyltransferase activity | Gene Ontology | GO:0015020 | 5 (0.64%) | 30 (0.14%) | 0.00485928849594 | 0.023922651057 | Ugt3a2  Ugt1a6b  Ugt1a7c  Ugt1a9  Ugt2b36 | web link |
| metal ion binding | Gene Ontology | GO:0046872 | 161 (20.67%) | 3545 (17.13%) | 0.00504917612161 | 0.0239368349469 | Pdlim5  Isca2  Zdhhc20  Kdm5a  Pan3  Nme6  Man2c1  Tent2  Esr1  Mocs1  Klf3  Mgat1  Rnf214  Cyp2r1  Mta2  Atf2  Rnf44  Arap1  Zbtb14  Cyp3a59  Zbtb18  Trf  Zfp367  Mylpf  Ablim2  Abl1  Cenpv  Cyp2d10  Extl2  Enpp2  Cyp4b1  Helz  Rai1  Atp11c  Nudt14  Rnf220  Actn1  Bpnt1  Ppp5c  Snrk  Rspry1  Asph  Haghl  Enpp3  Zfand4  Zfand3  Hdac10  Trim3  Trim7  Foxk2  Smpd4  Ercc5  Itgav  Agap3  Gna13  Zfp58  Riox2  S100a16  Fgd6  Akap8  Zmiz2  Zbtb42  Dido1  Lipc  Dna2  Zfp780b  Slc25a23  Xiap  Fkbp7  Haao  Slc25a25  Alkbh2  Alkbh3  Eya3  Nln  Taf1b  Aox3  Mt2  Zkscan3  Zbtb43  Zfp639  Ankib1  Mtmr4  Mdm4  Adk  Aasdhppt  Trim11  Man1a  Comt  Churc1  Gzf1  Zyx  Rnf141  Idh1  Nr1h3  Acp5  Aars  Zmynd8  Nucb1  Hagh  Cmah  Rora  Calu  P4ha1  S100a9  Cyp4a31  Atxn7l3  Zfyve19  Bmp1  Cutc  U2af1  Tec  Cbs  Uba5  Gmeb2  Mcfd2  Cp  Znrf1  Zfp825  Stk11  Rnpepl1  Apex1  Fxn  Fbp1  Cyp2c40  Cyb561d1  Lcp1  Pam  Nrp2  Rnf167  Sec23b  Kmt5b  Ndufs2  Pnkd  Zfp874a  Zfp874b  Mthfs  Nsd1  Dpagt1  Lman2l  Cela1  Ubox5  Dusp12  Acvr2b  Jmjd7  Acacb  Prlr  Rassf5  Zfp938  Zfp235  Adhfe1  Rnaseh2a  Gtpbp8  Zc3h7a  Zcchc7  Gne  Zfpm1  Glrx2  Zfp62  Rnf114  Nudt13 | web link |
| ribosome binding | Gene Ontology | GO:0043022 | 7 (0.90%) | 60 (0.29%) | 0.00711094887511 | 0.0325071948576 | Letmd1  Bag6  Sbds  Cpeb3  Mtif3  Sec61a2  Eif5a | web link |
| NAD binding | Gene Ontology | GO:0051287 | 6 (0.77%) | 47 (0.23%) | 0.00804131845371 | 0.0346709299256 | Grhpr  Aox3  Cyb5r3  Idh1  Ndufs2  Bdh2 | web link |
| chromatin binding | Gene Ontology | GO:0003682 | 30 (3.85%) | 499 (2.41%) | 0.00822137205616 | 0.0346709299256 | Wac  Macroh2a1  Npm1  Kdm5a  Yap1  Esr1  Kmt5b  Safb  Mapk15  Mbd3  Smarca2  Ybx1  Bcas3  Mta2  Atf2  Akap8  Tnrc18  Nap1l4  Tcf7l2  Ncapd3  Ssbp1  Brd3  Rcc1  Uxt  Srebf1  Camta2  Eny2  Mrnip  Ncor2  Eya3 | web link |
| enzyme binding | Gene Ontology | GO:0019899 | 27 (3.47%) | 437 (2.11%) | 0.00839686584136 | 0.0346709299256 | Ube2i  Fn1  Calu  Ugt1a6b  Lgals9  Fxn  Hdac10  Tmbim6  Mdm4  Esr1  Npm1  Golph3  Gckr  N4bp2l2  Atxn10  Stat1  Gstm4  Uhmk1  Ugt1a7c  Pcbp2  Luc7l2  Macroh2a1  Cbs  Arrb2  Ugt1a9  Slc4a2  Cpsf1 | web link |
| RNA polymerase II repressing transcription factor binding | Gene Ontology | GO:0001103 | 5 (0.64%) | 35 (0.17%) | 0.00948811819623 | 0.0379524727849 | Tcf7l2  Mier1  Mier2  Gtf2a2  Mta2 | web link |
| protein tyrosine phosphatase activity | Gene Ontology | GO:0004725 | 9 (1.16%) | 99 (0.48%) | 0.0123018073563 | 0.0469091630112 | Mtmr14  Acp1  Dusp16  Eya3  Mtmr4  Dusp11  Mtmr2  Dusp3  Dusp12 | web link |
| histone binding | Gene Ontology | GO:0042393 | 12 (1.54%) | 152 (0.73%) | 0.0124602464249 | 0.0469091630112 | Sbno2  Chd2  Tbl1xr1  Stat1  Rcc1  Npm1  Nap1l4  Bcas3  Anp32a  Smarca2  Gk  Kdm5a | web link |
| iron-sulfur cluster binding | Gene Ontology | GO:0051536 | 7 (0.90%) | 68 (0.33%) | 0.013811249075 | 0.050509710903 | Aox3  Isca2  Cmah  Mocs1  Glrx2  Ndufs2  Dna2 | web link |
| protein tyrosine/serine/threonine phosphatase activity | Gene Ontology | GO:0008138 | 5 (0.64%) | 40 (0.19%) | 0.0164983862539 | 0.0569988386237 | Dusp16  Dusp11  Eya3  Dusp3  Dusp12 | web link |
| enzyme inhibitor activity | Gene Ontology | GO:0004857 | 5 (0.64%) | 40 (0.19%) | 0.0164983862539 | 0.0569988386237 | Calu  Angptl4  Gckr  Ugt1a9  Fetub | web link |
| histone deacetylase binding | Gene Ontology | GO:0042826 | 10 (1.28%) | 122 (0.59%) | 0.0169215302164 | 0.0569988386237 | Mier1  Mier2  Akap8  Akap8l  Camta2  Ncor1  Hdac10  Kctd21  Ncor2  Mta2 | web link |
| unfolded protein binding | Gene Ontology | GO:0051082 | 8 (1.03%) | 88 (0.43%) | 0.0177397419152 | 0.0570271298272 | Nudcd2  Nudcd3  Dnajb6  Tcp1  Pdrg1  Clu  Npm1  Uggt1 | web link |
| protease binding | Gene Ontology | GO:0002020 | 10 (1.28%) | 123 (0.59%) | 0.017820978071 | 0.0570271298272 | Fn1  Derl1  Tysnd1  Serpinb6a  Itgav  Cflar  Xiap  Ubc  Aldoa  Bin1 | web link |
| lyase activity | Gene Ontology | GO:0016829 | 12 (1.54%) | 167 (0.81%) | 0.0243076377377 | 0.0758872592786 | Hoga1  L3hypdh  Naxd  Gstm4  Csad  Cbs  Mocs1  Aldoa  Rnaset2b  Gm49673  Pam  Echdc1 | web link |
| nucleotide binding | Gene Ontology | GO:0000166 | 83 (10.65%) | 1795 (8.67%) | 0.028838005794 | 0.0854113369376 | Ube2i  Entpd5  Flad1  Gtpbp2  Uckl1  Dcakd  Snrk  Aars  Irak1  Csnk1g2  Csnk1g3  Naxd  Uba5  Ntpcr  Opa1  Mmaa  Rtca  Mapkapk5  Helz  Abcd4  Pan3  Nme6  Map4k2  Csnk1a1  Sgk1  Mocs2  Pip5k1c  Ube2d3  Iigp1  Mapk15  Tcp1  Mast3  Gna13  Mocs1  Smarca2  Clk4  Orc4  Exosc10  Acvr2b  Stk11  Arl16  Rragc  Acacb  Rps6kb2  Mthfs  Uhmk1  Eif5  Ulk1  Chd2  Mapk6  Ube2g2  Idnk  Gne  Abl1  Septin9  Xylb  Kif21a  Gtpbp8  Psmc6  Rps6ka3  Adk  Mapkapk2  Mapk9  Papss2  Rab4b  Tent2  Spast  Fgfr2  Tec  Myo1c  Dna2  Mccc2  Ddx56  Farsa  Agap3  Atp11c  Prkar1a  Septin2  Rhobtb1  Cdk8  Nmnat3  Clpb  Gk | web link |
| ATP binding | Gene Ontology | GO:0005524 | 70 (8.99%) | 1484 (7.17%) | 0.0296069802951 | 0.0854113369376 | Ube2i  Entpd5  Naxd  Uckl1  Ppp5c  Snrk  Stk11  Rtca  Ube2d3  Csnk1g2  Csnk1g3  Uba5  Mapkapk5  Irak1  Aars  Abcd4  Chd2  Pan3  Nme6  Map4k2  Csnk1a1  Tent2  Pip5k1c  Npm1  Flad1  Myo1c  Adk  Mapk15  Tcp1  Gak  Smarca2  Clk4  Orc4  Acvr2b  Ulk1  Acacb  Mthfs  Uhmk1  Rps6kb2  Papss2  Mapk6  Ube2g2  Idnk  Gne  Abl1  Xylb  Kif21a  Psmc6  Rps6ka3  Ntpcr  Mapkapk2  Mapk9  Dna2  Sgk1  Spast  Fgfr2  Pfkfb2  Ddx56  Mccc2  Fkbp4  Farsa  Mast3  Atp11c  Tec  Cdk8  Nmnat3  Clpb  Gk  Helz  Dcakd | web link |
| structural constituent of ribosome | Gene Ontology | GO:0003735 | 12 (1.54%) | 172 (0.83%) | 0.0296706952891 | 0.0854113369376 | Mrpl36  Mrpl24  Rps10  Mrpl30  Rps19  Rpl35a  Rpl15  Uba52  Mrpl47  Kxd1  Rpl22l1  Rpl18 | web link |
| ubiquitin protein ligase activity | Gene Ontology | GO:0061630 | 18 (2.31%) | 294 (1.42%) | 0.0300274231421 | 0.0854113369376 | Smurf1  Ubox5  Btrc  Znrf1  Rnf114  Nedd4l  Huwe1  Ube2d3  Trim11  Rnf220  Xiap  Med7  Fbxo6  Ankib1  Rnf167  Trim3  Cdk8  Trim7 | web link |
| transcription coactivator activity | Gene Ontology | GO:0003713 | 14 (1.80%) | 213 (1.03%) | 0.031128008605 | 0.0866170674227 | Pmf1  Ss18  Rbm39  Nup98  Apex1  Eny2  Camta2  Atxn7l3  Ccar1  Smarca2  Npm1  Kdm5a  Mta2  Yap1 | web link |
| GTPase activator activity | Gene Ontology | GO:0005096 | 15 (1.93%) | 235 (1.14%) | 0.0329318571553 | 0.0870475022769 | Arhgef12  Arap1  Ralbp1  Arhgef19  Tiam2  Rin3  Grtp1  Arhgap32  Agap3  Arhgdib  Rgs3  Arhgap12  Sec23b  Tbc1d17  Rabgap1 | web link |
| protein serine kinase activity | Gene Ontology | GO:0106310 | 20 (2.57%) | 340 (1.64%) | 0.0333028527966 | 0.0870475022769 | Mast3  Mapk9  Rps6ka3  Csnk1a1  Sgk1  Csnk1g3  Snrk  Stk11  Uhmk1  Rps6kb2  Csnk1g2  Mapk15  Ulk1  Mapk6  Cdk8  Mapkapk5  Irak1  Clk4  Mapkapk2  Map4k2 | web link |
| exonuclease activity | Gene Ontology | GO:0004527 | 5 (0.64%) | 48 (0.23%) | 0.0336566156229 | 0.0870475022769 | Apex1  Exosc10  Rexo5  Dclre1c  Aen | web link |
| protein threonine kinase activity | Gene Ontology | GO:0106311 | 20 (2.57%) | 341 (1.65%) | 0.0341838086999 | 0.0870475022769 | Rps6ka3  Csnk1a1  Sgk1  Csnk1g3  Snrk  Stk11  Uhmk1  Irak1  Rps6kb2  Csnk1g2  Mapk15  Ulk1  Map4k2  Mapk6  Cdk8  Mapkapk5  Mapk9  Clk4  Mapkapk2  Mast3 | web link |
| carboxylic ester hydrolase activity | Gene Ontology | GO:0052689 | 6 (0.77%) | 65 (0.31%) | 0.0352176336549 | 0.0870475022769 | Lipc  Ces3a  Mgll  Baat  Pnpla6  Ces1f | web link |
| protein homodimerization activity | Gene Ontology | GO:0042803 | 35 (4.49%) | 676 (3.27%) | 0.0359669851995 | 0.0870475022769 | Actn1  Aox3  Slc8b1  Acox2  Idh1  Acox1  Tyms  Grhpr  Ugt1a6b  Smim1  Uba5  Naa60  Mmaa  Irak1  Npm1  Bcl2l1  Ghr  Usf2  Gtf2a2  Cyp2r1  Fgfr2  S100a16  Hoga1  Stat1  St6gal1  H13  Gstm4  Cep43  Ugt1a7c  Uhrf1bp1l  Mff  Nectin3  Mgll  Cbs  Ugt1a9 | web link |
| RNA polymerase II activating transcription factor binding | Gene Ontology | GO:0001102 | 5 (0.64%) | 49 (0.24%) | 0.0363639025568 | 0.0870475022769 | Tbx3  Gtf2i  Zfpm1  Ncor1  Atf2 | web link |
| dioxygenase activity | Gene Ontology | GO:0051213 | 7 (0.90%) | 83 (0.40%) | 0.0367231650231 | 0.0870475022769 | Alkbh3  Asph  Kdm5a  P4ha1  Haao  Alkbh2  Riox2 | web link |
| nucleotidyltransferase activity | Gene Ontology | GO:0016779 | 7 (0.90%) | 84 (0.41%) | 0.0388166013055 | 0.0896030579456 | Flad1  Tent2  Pcyt2  Gmppa  Papss2  Polrmt  Nmnat3 | web link |
| oxidoreductase activity, acting on the CH-OH group of donors, NAD or NADP as acceptor | Gene Ontology | GO:0016616 | 5 (0.64%) | 50 (0.24%) | 0.0392013378512 | 0.0896030579456 | Cbr4  Idh1  Bdh2  Dhrs3  Grhpr | web link |
| transcription regulatory region sequence-specific DNA binding | Gene Ontology | GO:0000976 | 15 (1.93%) | 246 (1.19%) | 0.0460896806342 | 0.103499633705 | Nr1h3  Foxk2  Arap1  Zbtb14  Mterf3  Tcf7l2  Macroh2a1  Per1  Srebf1  Hnrnpl  Zfp639  Smarca2  Yap1  Kdm5a  Tbl1xr1 | web link |
| iron ion binding | Gene Ontology | GO:0005506 | 12 (1.54%) | 185 (0.89%) | 0.0475148609972 | 0.10486038289 | Cyp2d10  Aox3  Isca2  Cyp3a59  Cyp4b1  Sc5d  P4ha1  Haao  Cyp2c40  Cyp4a31  Cyp2r1  Riox2 | web link |
| pyridoxal phosphate binding | Gene Ontology | GO:0030170 | 5 (0.64%) | 53 (0.26%) | 0.0485032394087 | 0.105227366853 | Alas1  Kynu  Cbs  Csad  Agxt2 | web link |
| endonuclease activity | Gene Ontology | GO:0004519 | 7 (0.90%) | 89 (0.43%) | 0.0504471524144 | 0.107620591817 | Dna2  Rnaseh2a  Ercc5  Apex1  Gm49673  Rnaset2b  Dclre1c | web link |
| double-stranded DNA binding | Gene Ontology | GO:0003690 | 10 (1.28%) | 149 (0.72%) | 0.0553111589293 | 0.113854711401 | Stat1  Hdgfl2  Mterf3  Polg2  Akap8  Tef  Usf2  Hnrnpk  Camta2  Cenpx | web link |
| protein-containing complex binding | Gene Ontology | GO:0044877 | 31 (3.98%) | 609 (2.94%) | 0.0555034812689 | 0.113854711401 | Ppp5c  Stk11  Coro1b  Derl1  Derl2  Opa1  Clu  Bin1  Bcl2l1  Hmbox1  Usf2  Ndel1  Nr1h3  Cflar  Inca1  Ugt1a7c  Rbp4  Ywhaz  Kctd2  Ddb1  Mff  Ugt1a6b  Esr1  Tef  Atp5j  Apex1  Srebf1  Arrb2  Ddb2  Atf2  Ncor2 | web link |
| flavin adenine dinucleotide binding | Gene Ontology | GO:0050660 | 6 (0.77%) | 73 (0.35%) | 0.0566857874295 | 0.113854711401 | Aox3  Cyb5r3  Fmo5  Acox2  Acox1  Maob | web link |
| transferase activity, transferring acyl groups | Gene Ontology | GO:0016746 | 11 (1.41%) | 170 (0.82%) | 0.0569273557006 | 0.113854711401 | Lipc  Crot  Baat  Taz  Alas1  Ggt6  Lpcat3  Naa60  Zdhhc20  Atat1  Naa80 | web link |
| hydrolase activity, acting on glycosyl bonds | Gene Ontology | GO:0016798 | 7 (0.90%) | 93 (0.45%) | 0.0611826877302 | 0.120482831223 | Man2c1  Gba  Ctbs  Hyal1  Man1a  Ganc  Gba2 | web link |
| heat shock protein binding | Gene Ontology | GO:0031072 | 6 (0.77%) | 75 (0.36%) | 0.0630539180163 | 0.122286386456 | Mettl23  Ppp5c  Dnajb6  Fkbp4  Hnrnpk  Irak1 | web link |
| monooxygenase activity | Gene Ontology | GO:0004497 | 9 (1.16%) | 136 (0.66%) | 0.0715906610604 | 0.136770218145 | Cyp2d10  Jmjd7  Fmo5  Cyp3a59  Cyp4b1  Cyp2c40  Cyp4a31  Cyp2r1  Pam | web link |
| ubiquitin protein ligase binding | Gene Ontology | GO:0031625 | 17 (2.18%) | 307 (1.48%) | 0.0734457611188 | 0.137659935869 | Ubox5  Ywhaz  Tcp1  Bag6  Tmbim6  Derl1  H13  Uba52  Pcbp2  Cbs  Ndufs2  Arrb2  Prkar1a  Ubc  Per1  Clu  Atxn3 | web link |
| nuclease activity | Gene Ontology | GO:0004518 | 9 (1.16%) | 137 (0.66%) | 0.0742073091792 | 0.137659935869 | Exosc10  Dna2  Rnaseh2a  Ercc5  Apex1  Gm49673  Rnaset2b  Dclre1c  Aen | web link |
| protein kinase C binding | Gene Ontology | GO:0005080 | 5 (0.64%) | 61 (0.29%) | 0.0791368976723 | 0.144707470029 | Abl1  Pdlim5  Itgav  Pard6a  Ugt1a9 | web link |
| ATPase binding | Gene Ontology | GO:0051117 | 7 (0.90%) | 100 (0.48%) | 0.0830920701873 | 0.149799788507 | Vcpkmt  Snx10  Fxyd1  Esr1  Derl1  Hnrnpk  Atxn3 | web link |
| zinc ion binding | Gene Ontology | GO:0008270 | 35 (4.49%) | 730 (3.53%) | 0.0855698638962 | 0.152124202482 | Mt2  Aars  Rnpepl1  Uba5  Zfand3  Hdac10  Trim3  Pam  Trim7  Trim11  Rnf167  Cryz  Esr1  Churc1  Kdm5a  Rtn4ip1  Sec24c  Sec24b  Nsd1  Mta2  Nr1h3  Zmynd8  Sec23b  Akap8  Rora  Zdhhc20  Zmiz2  Atxn7l3  Bmp1  Enpp2  Enpp3  Albfm1  Zfand4  Dusp12  Zcchc7 | web link |
| DNA binding | Gene Ontology | GO:0003677 | 78 (10.01%) | 1779 (8.60%) | 0.0868038202943 | 0.152203958872 | Taf1b  Hsf2  Cers2  Hsf4  Taf1d  Abl1  Orc4  Dnajb6  Apex1  Macroh2a1  Zbtb42  Hnrnpk  Zfand3  Crem  Tial1  Lcorl  Hmbox1  Npm1  Kdm5a  Tfdp1  Nr1h3  Hnrnpdl  Esr1  Mlxipl  Mier2  Huwe1  Safb  Ercc5  Churc1  Usf2  Srebf1  Gzf1  Rfxap  Mbd3  Srrt  Klf3  Zfp58  Ybx1  Dedd  Mta2  Atf2  Hmces  Stat1  Zbtb14  Mterf3  Zbtb18  Akap8  Rora  Polg2  Banp  Ssbp1  Chd2  Zfp367  Tcf7l2  Cenpx  Polrmt  Gtf2i  Foxk2  Rcc1  Dna2  Tbl1xr1  Tbx3  Hmgb3  Ddb2  Zfpm1  Zkscan3  Tef  Son  Pcbp2  Akap8l  Zbtb43  Camta2  Gmeb2  Zfp639  Ddb1  Nucb1  Ncor2  Ncor1 | web link |
| mRNA 3'-UTR binding | Gene Ontology | GO:0003730 | 6 (0.77%) | 83 (0.40%) | 0.0925526462158 | 0.158331181801 | Dazap1  Cryz  Cpeb3  Hnrnpk  Hnrnpl  Hnrnpa2b1 | web link |
| chromatin DNA binding | Gene Ontology | GO:0031490 | 5 (0.64%) | 64 (0.31%) | 0.0927721768367 | 0.158331181801 | Apex1  Sbno2  Per1  Kdm5a  Macroh2a1 | web link |
| methyltransferase activity | Gene Ontology | GO:0008168 | 11 (1.41%) | 187 (0.90%) | 0.0961667999704 | 0.159822018346 | Vcpkmt  Kmt5b  Comt  Mettl17  Mettl23  Tyms  Trmt5  Nsd1  Hemk1  Setd4  Icmt | web link |
| phosphatase activity | Gene Ontology | GO:0016791 | 9 (1.16%) | 145 (0.70%) | 0.0972367823187 | 0.159822018346 | Mtmr4  Acp1  Ppp5c  Fbp1  Dusp16  Dusp11  Mtmr2  Dusp3  Dusp12 | web link |
| protein phosphatase binding | Gene Ontology | GO:0019903 | 7 (0.90%) | 104 (0.50%) | 0.0973915424299 | 0.159822018346 | Pard3  Hsf4  Ghr  Ppp6r3  Ppp6r1  Mtmr4  Anapc5 | web link |
| protein serine/threonine kinase activity | Gene Ontology | GO:0004674 | 21 (2.70%) | 412 (1.99%) | 0.0995185066762 | 0.161245175374 | Acvr2b  Rps6ka3  Csnk1a1  Sgk1  Csnk1g3  Snrk  Stk11  Uhmk1  Irak1  Rps6kb2  Csnk1g2  Mapk15  Ulk1  Map4k2  Mapk6  Cdk8  Mapkapk5  Mapk9  Clk4  Mapkapk2  Mast3 | web link |
| sequence-specific DNA binding | Gene Ontology | GO:0043565 | 23 (2.95%) | 462 (2.23%) | 0.106249793079 | 0.169999668926 | Hsf2  Hsf4  Foxk2  Hmbox1  Mlxipl  Safb  Usf2  Gzf1  Ybx1  Mta2  Atf2  Zbtb14  Zbtb18  Tcf7l2  Tbx3  Rora  Polrmt  Esr1  Tef  Srebf1  Camta2  Nr1h3  Ncor2 | web link |
| protein kinase activity | Gene Ontology | GO:0004672 | 26 (3.34%) | 533 (2.58%) | 0.107693062663 | 0.170181629887 | Snrk  Stk11  Csnk1g2  Csnk1g3  Mapkapk5  Irak1  Mapkapk2  Pan3  Rps6ka3  Csnk1a1  Sgk1  Mapk15  Gak  Clk4  Acvr2b  Ulk1  Uhmk1  Map4k2  Mapk6  Abl1  Mapk9  Fgfr2  Tec  Rps6kb2  Mast3  Cdk8 | web link |
| protein domain specific binding | Gene Ontology | GO:0019904 | 17 (2.18%) | 329 (1.59%) | 0.117337202093 | 0.181375264802 | Trim11  Ywhaz  Actn1  Zmynd8  Nolc1  Hax1  Gckr  Dffa  Abl1  Hnrnpk  Arrb2  Hnrnpm  Nde1  Sorbs2  Tacc2  Tfdp1  Prkar1a | web link |
| integrin binding | Gene Ontology | GO:0005178 | 8 (1.03%) | 130 (0.63%) | 0.117677379279 | 0.181375264802 | Actn1  Fn1  Nisch  Itgav  Fermt3  Gfra1  Lcp1  Fgf1 | web link |
| magnesium ion binding | Gene Ontology | GO:0000287 | 12 (1.54%) | 217 (1.05%) | 0.119027517526 | 0.181375264802 | Rps6ka3  Bpnt1  Foxk2  Csnk1a1  Snrk  Stk11  Comt  Mast3  Atp11c  Abl1  Idh1  Aasdhppt | web link |
| guanyl-nucleotide exchange factor activity | Gene Ontology | GO:0005085 | 12 (1.54%) | 218 (1.05%) | 0.121926770298 | 0.183607371743 | Rcc1  Madd  Dock9  Plekhg3  Arhgef19  Dennd10  Arhgef12  Rin3  Tiam2  Fgd6  Nucb1  Lamtor2 | web link |
| protein kinase binding | Gene Ontology | GO:0019901 | 25 (3.21%) | 523 (2.53%) | 0.132485772451 | 0.197188126439 | Macroh2a1  Npm1  Pam  Dusp3  Bcl2l1  Rps6ka3  Esr1  Mlxipl  Tprkb  Kidins220  Fbxw5  Nisch  Atf2  Ghr  Prlr  Rps19  Tcf7l2  Slc12a7  Mapk6  Cep43  Abl1  Ywhaz  Pfkfb2  Srebf1  Rhobtb1 | web link |
| GTP binding | Gene Ontology | GO:0005525 | 19 (2.44%) | 384 (1.86%) | 0.137680066952 | 0.202563776664 | Arhgap5  Rab4b  Rragc  Gm4951  Arl16  Gtpbp2  Eif5  Srl  Mocs1  Fkbp4  Septin2  Agap3  Gna13  Opa1  Mmaa  Septin9  Gtpbp8  Iigp1  Rhobtb1 | web link |
| phosphoprotein phosphatase activity | Gene Ontology | GO:0004721 | 8 (1.03%) | 137 (0.66%) | 0.145540153468 | 0.211694768681 | Dusp16  Acp1  Ppp5c  Eya3  Mtmr4  Dusp11  Dusp3  Dusp12 | web link |
| actin filament binding | Gene Ontology | GO:0051015 | 11 (1.41%) | 205 (0.99%) | 0.151791815938 | 0.218307330788 | Actn1  Cttn  Neb  Myo1c  Arpc5  Coro1b  Mprip  Ablim2  Abl1  Lcp1  Bin1 | web link |
| GTPase activity | Gene Ontology | GO:0003924 | 15 (1.93%) | 298 (1.44%) | 0.156386737601 | 0.222416693476 | Gm4951  Rab4b  Rragc  Arhgap5  Opa1  Septin2  Agap3  Arhgdib  Gna13  Gngt1  Gtpbp2  Mmaa  Septin9  Iigp1  Rhobtb1 | web link |
| oxidoreductase activity, acting on paired donors, with incorporation or reduction of molecular oxygen | Gene Ontology | GO:0016705 | 7 (0.90%) | 119 (0.57%) | 0.161907737997 | 0.227738356743 | Cyp2d10  Cyp3a59  Cyp4b1  P4ha1  Cyp2c40  Cyp4a31  Cyp2r1 | web link |
| single-stranded DNA binding | Gene Ontology | GO:0003697 | 6 (0.77%) | 103 (0.50%) | 0.192138822101 | 0.267323578575 | Hmces  Ercc5  Hnrnpk  Pcbp2  Ssbp1  Ybx1 | web link |
| protein serine/threonine phosphatase activity | Gene Ontology | GO:0004722 | 5 (0.64%) | 87 (0.42%) | 0.229884779032 | 0.316400556087 | Mtmr4  Ppp5c  Dusp16  Dusp3  Dusp12 | web link |
| lipid binding | Gene Ontology | GO:0008289 | 14 (1.80%) | 299 (1.44%) | 0.238136001512 | 0.324270299932 | Dbi  Lipc  Zfyve19  Esr1  Golph3  Gsdmd  Osbpl9  Pard3  Insig2  Plekha1  Opa1  Snx10  Gm44805  Apoc3 | web link |
| beta-catenin binding | Gene Ontology | GO:0008013 | 5 (0.64%) | 89 (0.43%) | 0.243883001887 | 0.328600255174 | Tcf7l2  Btrc  Tbl1xr1  Rora  Esr1 | web link |
| transcription coregulator activity | Gene Ontology | GO:0003712 | 6 (0.77%) | 121 (0.58%) | 0.304568121931 | 0.406090829241 | Uxt  Hdgfl2  Camta2  Med7  Nsd1  Mta2 | web link |
| peptidase inhibitor activity | Gene Ontology | GO:0030414 | 6 (0.77%) | 122 (0.59%) | 0.311182375774 | 0.409352000234 | Serpina3m  Serpinb6a  Tfpi2  Fetub  Wfdc2  Itih4 | web link |
| phosphatidylinositol binding | Gene Ontology | GO:0035091 | 5 (0.64%) | 99 (0.48%) | 0.316608187681 | 0.409352000234 | Nisch  Pard3  Arhgap32  Sgk1  Snx10 | web link |
| thiol-dependent ubiquitin-specific protease activity | Gene Ontology | GO:0004843 | 5 (0.64%) | 99 (0.48%) | 0.316608187681 | 0.409352000234 | Usp47  Josd2  Usp40  Mindy3  Atxn3 | web link |
| cysteine-type peptidase activity | Gene Ontology | GO:0008234 | 7 (0.90%) | 151 (0.73%) | 0.341733389001 | 0.437418737922 | Senp6  Usp47  Usp40  Cflar  Mindy3  Atg4d  Atxn3 | web link |
| chaperone binding | Gene Ontology | GO:0051087 | 5 (0.64%) | 103 (0.50%) | 0.346490999353 | 0.439117306111 | Ahsa2  Cp  Bin1  Dnajb6  Fn1 | web link |
| ligase activity | Gene Ontology | GO:0016874 | 6 (0.77%) | 132 (0.64%) | 0.378256635819 | 0.474674993969 | Acacb  Mthfs  Rtca  Aars  Farsa  Mccc2 | web link |
| heme binding | Gene Ontology | GO:0020037 | 8 (1.03%) | 183 (0.88%) | 0.384079739687 | 0.477302977475 | Cyp2d10  Cyp3a59  Cyp4b1  Cbs  Cyp2c40  Cyb561d1  Cyp4a31  Cyp2r1 | web link |
| isomerase activity | Gene Ontology | GO:0016853 | 6 (0.77%) | 136 (0.66%) | 0.405261984354 | 0.498783980743 | Spast  Fkbp7  Mri1  Fkbp4  Ppil3  Gne | web link |
| serine-type endopeptidase inhibitor activity | Gene Ontology | GO:0004867 | 5 (0.64%) | 120 (0.58%) | 0.47314366761 | 0.575129006096 | Tfpi2  Wfdc2  Itih4  Serpina3m  Serpinb6a | web link |
| DNA-binding transcription activator activity, RNA polymerase II-specific | Gene Ontology | GO:0001228 | 19 (2.44%) | 491 (2.37%) | 0.483896592965 | 0.575129006096 | Nr1h3  Hsf2  Hsf4  Foxk2  Vezf1  Esr1  Zfp780b  Tcf7l2  Srebf1  Gzf1  Tef  Gmeb2  Zfp639  Rfxap  Gtf2i  Mdm4  Zfp62  Tfdp1  Atf2 | web link |
| protein C-terminus binding | Gene Ontology | GO:0008022 | 9 (1.16%) | 227 (1.10%) | 0.484683195973 | 0.575129006096 | Ube2i  Gm20498  Fn1  Prrc2c  Myo1c  Epb41  Abl1  Mpdz  Yap1 | web link |
| heparin binding | Gene Ontology | GO:0008201 | 6 (0.77%) | 148 (0.72%) | 0.485265098893 | 0.575129006096 | Lipc  Fn1  Chrd  Vegfa  Nrp2  Fgf1 | web link |
| RNA polymerase II transcription regulatory region sequence-specific DNA binding | Gene Ontology | GO:0000977 | 15 (1.93%) | 397 (1.92%) | 0.531142225053 | 0.623726649604 | Crem  Hsf2  Hsf4  Tfdp1  Zbtb43  Rora  Macroh2a1  Srebf1  Klf3  Vezf1  Zfp938  Atf2  Atxn3  Rfxap  Zfp825 | web link |
| carbohydrate binding | Gene Ontology | GO:0030246 | 8 (1.03%) | 215 (1.04%) | 0.563455040715 | 0.65565677465 | Man2c1  Gckr  Nomo1  Ganc  Lgals9  Fbxo6  Lman2l  Gpcpd1 | web link |
| small GTPase binding | Gene Ontology | GO:0031267 | 10 (1.28%) | 271 (1.31%) | 0.570548224011 | 0.657929483544 | Rcc1  Ralbp1  Rabgap1  Dennd10  Myo1c  Rin3  Arhgdib  Pard6a  Ppp6r1  Rassf5 | web link |
| transmembrane transporter activity | Gene Ontology | GO:0022857 | 8 (1.03%) | 219 (1.06%) | 0.58443983692 | 0.667931242194 | Slc2a9  Ralbp1  Slc22a30  Slc35b3  Slc17a2  Slc12a7  Slco1b2  Slc43a1 | web link |
| helicase activity | Gene Ontology | GO:0004386 | 5 (0.64%) | 137 (0.66%) | 0.590888680899 | 0.669325231461 | Smarca2  Dna2  Ddx56  Chd2  Helz | web link |
| protein heterodimerization activity | Gene Ontology | GO:0046982 | 13 (1.67%) | 365 (1.76%) | 0.620199180412 | 0.696363992042 | Bcl2l1  Rcc1  Rragc  Macroh2a1  Mlxipl  Nolc1  Usf2  Ugt1a6b  Ugt1a7c  Mapk6  Ugt1a9  Gtf2a2  Irak1 | web link |
| DNA-binding transcription factor activity | Gene Ontology | GO:0003700 | 22 (2.82%) | 616 (2.98%) | 0.630956142621 | 0.702281619613 | Hsf2  Hsf4  Zfp639  Crem  Tfdp1  Stat1  Esr1  Mlxipl  Usf2  Nr1h3  Arap1  Zbtb14  Tcf7l2  Zfp362  Zfp367  Rora  Gtf2i  Foxk2  Tbx3  Tef  Srebf1  Atf2 | web link |
| serine-type endopeptidase activity | Gene Ontology | GO:0004252 | 7 (0.90%) | 202 (0.98%) | 0.6408938819 | 0.705944675808 | Tysnd1  Hp  Tmprss6  Rhbdf2  Rhbdd3  Proc  Cela1 | web link |
| peptidase activity | Gene Ontology | GO:0008233 | 19 (2.44%) | 538 (2.60%) | 0.64527755523 | 0.705944675808 | Hmces  Josd2  Ctsa  Bmp1  Desi1  Tysnd1  Usp47  H13  Usp40  Tmprss6  Rnpepl1  Senp6  Jmjd7  Mindy3  Atg4d  Cela1  Proc  Atxn3  Nln | web link |
| calmodulin binding | Gene Ontology | GO:0005516 | 6 (0.77%) | 182 (0.88%) | 0.685517433583 | 0.743612131344 | Camsap2  Myo1c  Phkb  Mapkapk5  Epb41  Mapkapk2 | web link |
| signaling receptor binding | Gene Ontology | GO:0005102 | 12 (1.54%) | 364 (1.76%) | 0.720619159173 | 0.772478204397 | Bag6  Fn1  Gba  Erbin  Trak1  Myo1c  Itgav  Thpo  Lgals9  Angptl4  Arrb2  Clu | web link |
| microtubule binding | Gene Ontology | GO:0008017 | 8 (1.03%) | 249 (1.20%) | 0.724198316623 | 0.772478204397 | Spast  Camsap2  Ppp5c  Opa1  Sbds  Ndel1  Nde1  Kif21a | web link |
| ATPase activity | Gene Ontology | GO:0016887 | 11 (1.41%) | 343 (1.66%) | 0.747472601078 | 0.786810286298 | Dna2  Spast  Ddx56  Atp5j  Tcp1  Chd2  Atp11c  Smarca2  Clpb  Abcd4  Psmc6 | web link |
| sequence-specific double-stranded DNA binding | Gene Ontology | GO:1990837 | 17 (2.18%) | 517 (2.50%) | 0.749928554128 | 0.786810286298 | Hmbox1  Zbtb18  Hsf4  Zbtb14  Esr1  Hsf2  Tef  Usf2  Abl1  Zbtb43  Klf3  Crem  Gmeb2  Tbx3  Srebf1  Ybx1  Atf2 | web link |
| calcium ion binding | Gene Ontology | GO:0005509 | 23 (2.95%) | 709 (3.43%) | 0.797329945126 | 0.829741731513 | Actn1  Asph  Calu  Lcp1  Proc  Pam  Ltbp3  Man1a  S100a16  Ppp2r3d  Myl6  S100a9  Mylpf  Susd1  Bmp1  Enpp2  Enpp3  Slc25a23  Fkbp7  Slc25a25  Mcfd2  Smoc1  Nucb1 | web link |
| RNA polymerase II cis-regulatory region sequence-specific DNA binding | Gene Ontology | GO:0000978 | 36 (4.62%) | 1101 (5.32%) | 0.832844770637 | 0.859710730981 | Hsf2  Hsf4  Zbtb42  Zfp639  Crem  Yap1  Foxk2  Esr1  Mlxipl  Safb  Usf2  Klf3  Zfp874a  Zfp874b  Nsd1  Gzf1  Zfp58  Atf2  Stat1  Zbtb14  Zbtb18  Rora  Tbx3  Per1  Zfp367  Tcf7l2  Chd2  Zfp362  Zfp780b  Tef  Srebf1  Gmeb2  Ccar1  Nr1h3  Zfp235  Zfp62 | web link |
| DNA-binding transcription repressor activity, RNA polymerase II-specific | Gene Ontology | GO:0001227 | 9 (1.16%) | 336 (1.62%) | 0.889473713767 | 0.910821082897 | Foxk2  Zbtb14  Mlxipl  Zbtb18  Tbx3  Gzf1  Crem  Klf3  Hsf4 | web link |
| actin binding | Gene Ontology | GO:0003779 | 10 (1.28%) | 384 (1.86%) | 0.916485273528 | 0.931032658822 | Pdlim5  Actn1  Myo1c  Neb  Arpc5  Epb41  Coro1b  Mprip  Ablim2  Lcp1 | web link |
| molecular\_function | Gene Ontology | GO:0003674 | 106 (13.61%) | 3542 (17.11%) | 0.997158912988 | 0.997949041791 | Fam149b  Dop1b  Arl16  1110032A03Rik  Retreg1  Card19  Cdv3  Tmem258  Fam204a  Tmem256  Ccdc30  Cnot10  Wdr37  Iqcc  Slc39a9  2700062C07Rik  Bri3bp  Wbp1l  Szrd1  Manf  Cenpv  Arv1  Shld2  Mettl9  Gm5150  Thada  Smim10l1  Mrnip  C87436  Dram2  Ccdc163  Synrg  Ccdc97  Haghl  Plekha6  Zfand4  1110065P20Rik  Cdc123  Gpr155  4932438A13Rik  Ttc13  Fam234a  1110008P14Rik  Washc3  Prrc2b  Txnl4a  Tmem185a  Aamdc  Wsb1  Mpv17l2  Arhgap12  Pcnx3  2610528J11Rik  Vps13d  Wdr53  Chchd7  Atraid  Mup9  Tns3  Dmxl1  Slc66a2  9930021J03Rik  Tmem161b  Ggt6  Tmem208  Eapp  Gm527  Msantd2  Fam160a2  Mcfd2  Cox7a2  Trappc8  Zfp825  Cuedc2  Tmem131l  Polr1has  Entr1  Tmem11  Tm9sf1  Inip  Tmem234  Mtfr1l  Ccdc58  Higd1a  Cox19  Dcaf11  Yif1b  Ndufs6  Atxn10  Lysmd3  Letmd1  Gmppa  Ndufb2  Fbxo34  Pdrg1  Rcbtb2  Susd1  Tmem222  D130043K22Rik  Ociad2  Mrpl30  Anapc15  Nomo1  Fance  Gm826  Wdr20 | web link |
| DNA-binding transcription factor activity, RNA polymerase II-specific | Gene Ontology | GO:0000981 | 24 (3.08%) | 1055 (5.10%) | 0.997949041791 | 0.997949041791 | Hsf4  Tcf7l2  Zbtb42  Crem  Zfp938  Tfdp1  Zfp825  Foxk2  Mlxipl  Usf2  Klf3  Zfp874a  Zfp874b  Zfp58  Atf2  Stat1  Zbtb18  Rora  Tbx3  Zfp367  Vezf1  Tef  Srebf1  Zfp235 | web link |
