# Supplementary material for: Regulation of Alternative Splicing of Lipid Metabolism Genes in Sepsis-Induced Liver Damage by RNA-Binding Proteins
Source: Inflammation. 2024 May 9;47(6):1952–68. doi: 10.1007/s10753-024-02017-2 (PMC11607010; doi:10.1007/s10753-024-02017-2)
Supplement: Supplementary file 1 — Supplementary file1 (ZIP 38655 KB) [file 10753_2024_2017_MOESM1_ESM.zip › Sup5_AS_GO.zip!/Sup5_AS_GO/ras/CLP_vs_Sham/Ras_GO_analysis/Ras_GO_enrichment_P_top.pdf]

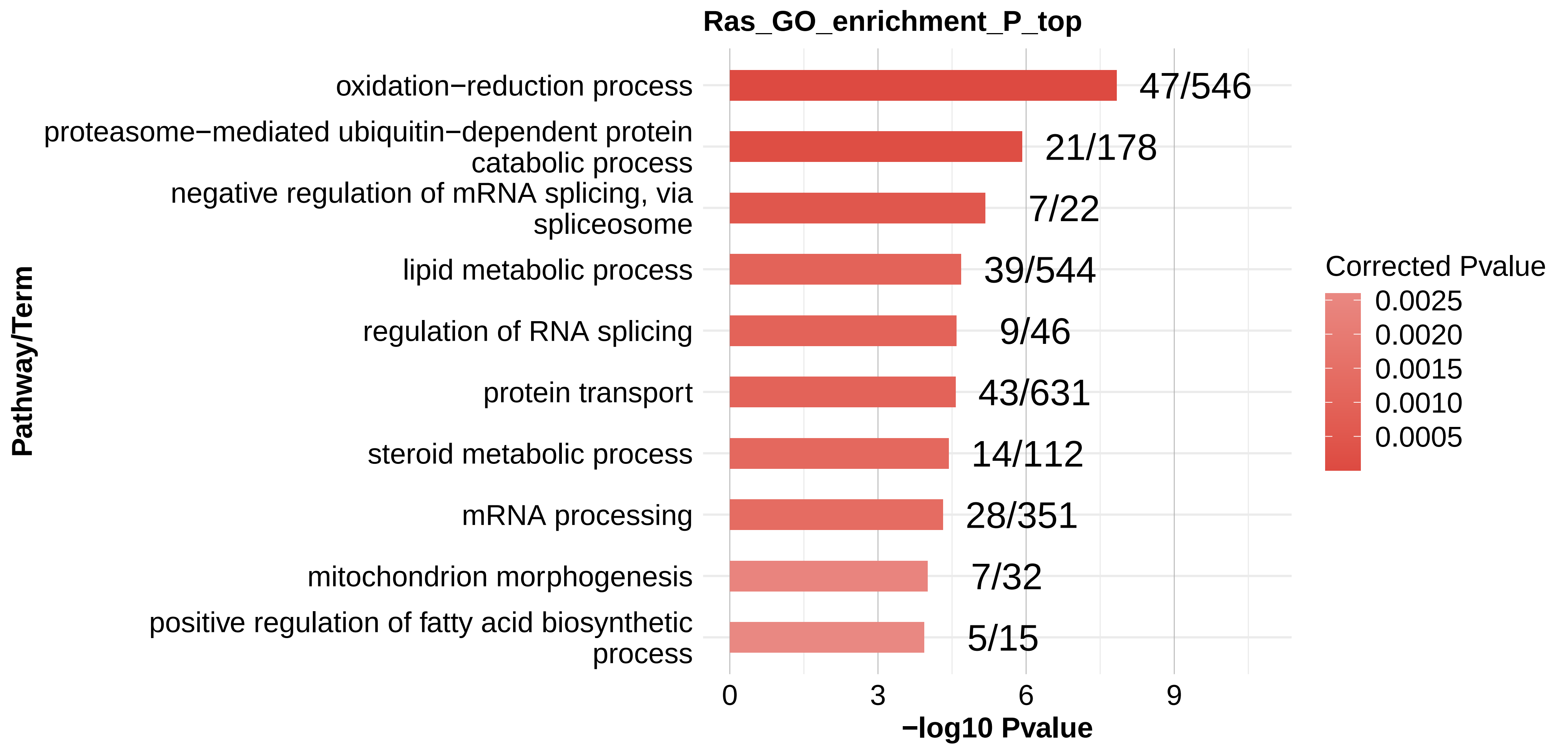
Ras_GO_enrichment_P_top
Pathway/Term
oxidation−reduction process
proteasome−mediated ubiquitin−dependent protein catabolic process
negative regulation of mRNA splicing, via spliceosome
lipid metabolic process
regulation of RNA splicing
protein transport
steroid metabolic process
mRNA processing
mitochondrion morphogenesis
positive regulation of fatty acid biosynthetic process
47/546
21/178
7/22
39/544
9/46
43/631
14/112
28/351
7/32
5/15
0
3
6
9
−log10 Pvalue
Corrected Pvalue
0.0025
0.0020
0.0015
0.0010
0.0005
