# Supplementary material for: Regulation of Alternative Splicing of Lipid Metabolism Genes in Sepsis-Induced Liver Damage by RNA-Binding Proteins
Source: Inflammation. 2024 May 9;47(6):1952–68. doi: 10.1007/s10753-024-02017-2 (PMC11607010; doi:10.1007/s10753-024-02017-2)
Supplement: Supplementary file 1 — Supplementary file1 (ZIP 38655 KB) [file 10753_2024_2017_MOESM1_ESM.zip › Sup5_AS_GO.zip!/Sup5_AS_GO/ras/CLP_vs_Sham/Ras_GO_analysis/Ras_GO_enrichment_F_top.pdf]

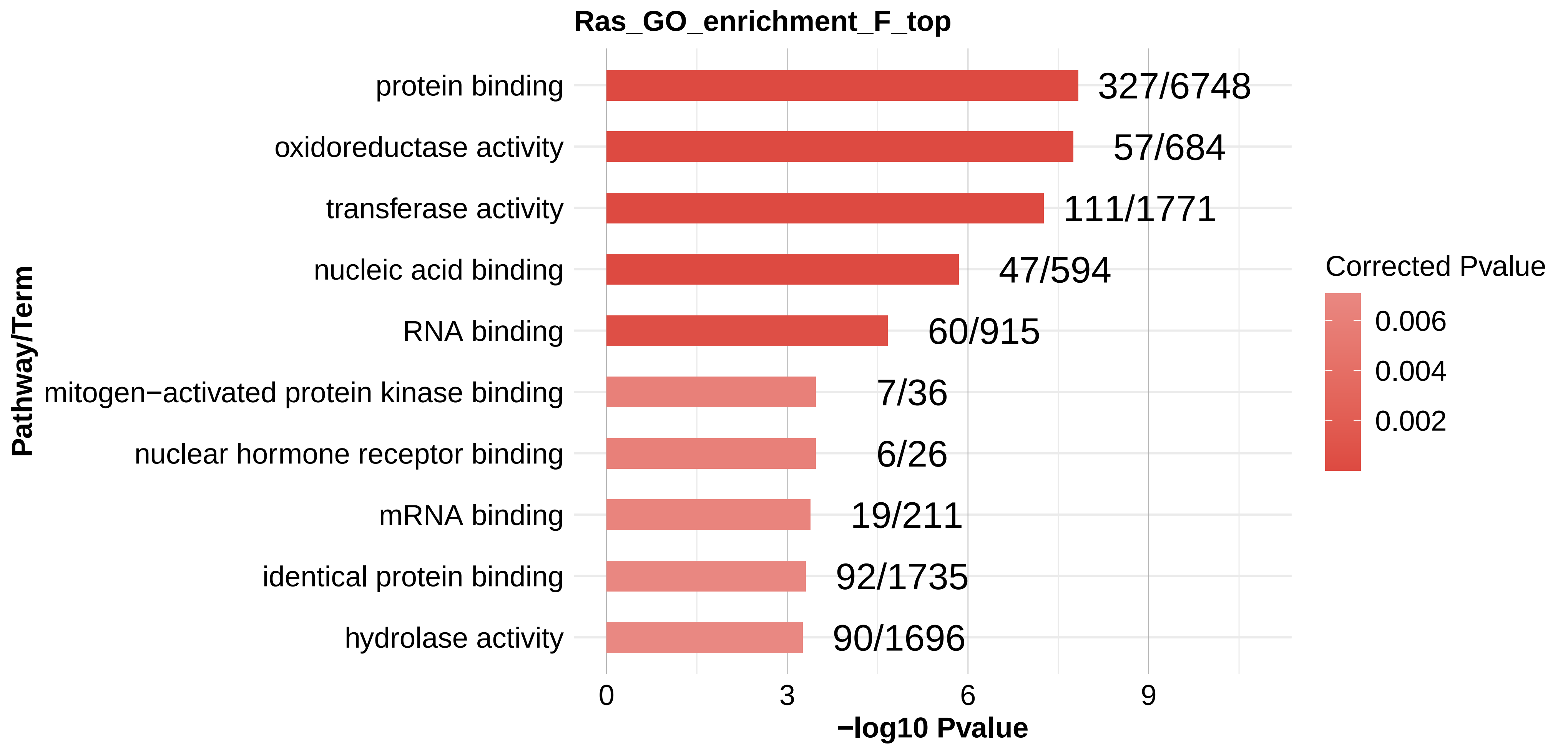

Ras_GO_enrichment_F_top
protein binding
oxidoreductase activity
transferase activity
nucleic acid binding
RNA binding
mitogen−activated protein kinase binding
nuclear hormone receptor binding
mRNA binding
identical protein binding
hydrolase activity
327/6748
57/684
111/1771
47/594
60/915
7/36
6/26
19/211
92/1735
90/1696
Pathway/Term
0
3
6
9
−log10 Pvalue
Corrected Pvalue
0.006
0.004
0.002
