# Supplementary material for: Regulation of Alternative Splicing of Lipid Metabolism Genes in Sepsis-Induced Liver Damage by RNA-Binding Proteins
Source: Inflammation. 2024 May 9;47(6):1952–68. doi: 10.1007/s10753-024-02017-2 (PMC11607010; doi:10.1007/s10753-024-02017-2)
Supplement: Supplementary file 1 — Supplementary file1 (ZIP 38655 KB) [file 10753_2024_2017_MOESM1_ESM.zip › Sup5_AS_GO.zip!/Sup5_AS_GO/ras/CLP_vs_Sham/Ras_GO_analysis/Ras_GO_enrichment_C_top.pdf]

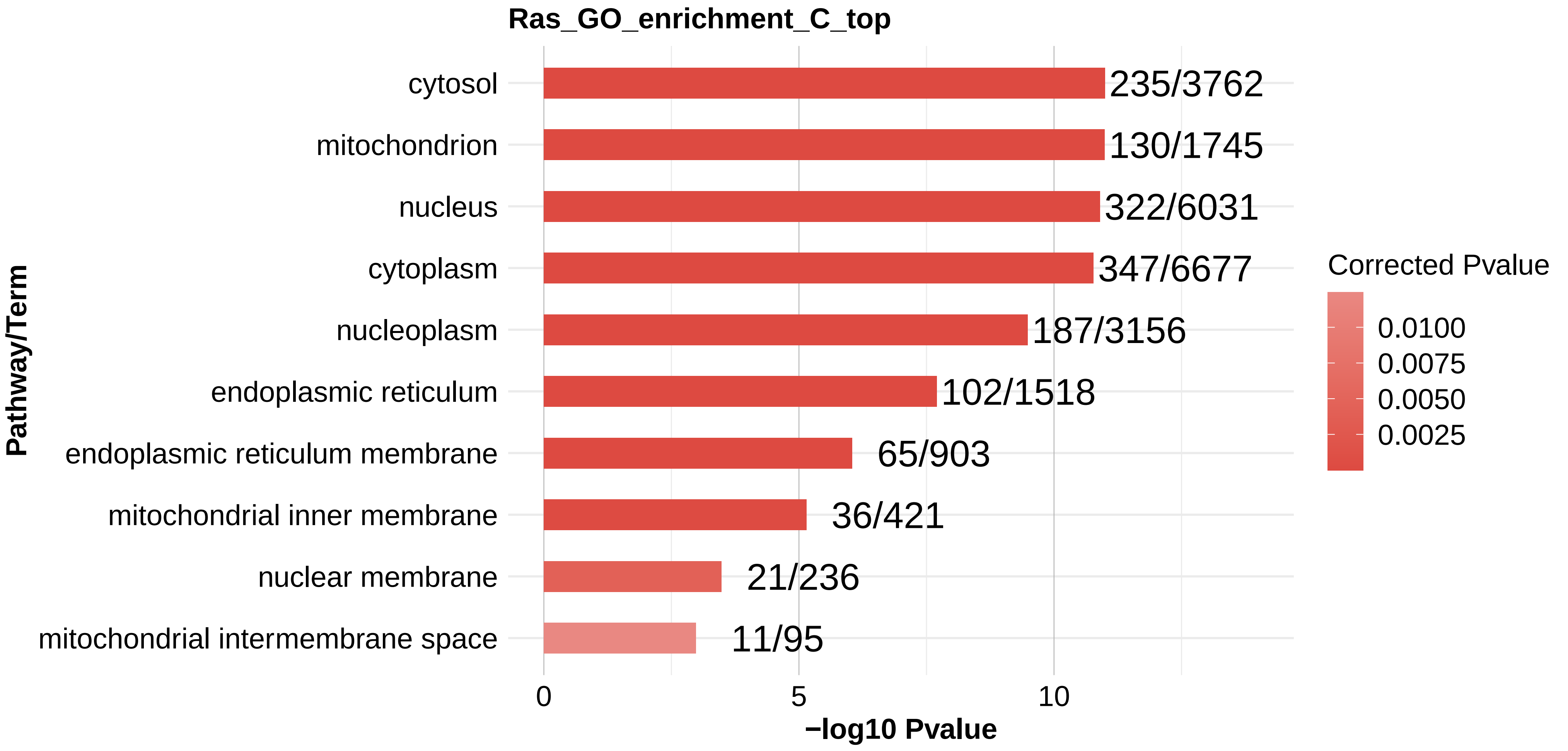

Ras_GO_enrichment_C_top
Pathway/Term
cytosol
mitochondrion
nucleus
cytoplasm
nucleoplasm
endoplasmic reticulum
endoplasmic reticulum membrane
mitochondrial inner membrane
nuclear membrane
mitochondrial intermembrane space
235/3762
130/1745
322/6031
347/6677
187/3156
102/1518
65/903
36/421
21/236
11/95
0
5
10
−log10 Pvalue
Corrected Pvalue
0.0100
0.0075
0.0050
0.0025
