# Supplementary material for: Regulation of Alternative Splicing of Lipid Metabolism Genes in Sepsis-Induced Liver Damage by RNA-Binding Proteins
Source: Inflammation. 2024 May 9;47(6):1952–68. doi: 10.1007/s10753-024-02017-2 (PMC11607010; doi:10.1007/s10753-024-02017-2)
Supplement: Supplementary file 1 — Supplementary file1 (ZIP 38655 KB) [file 10753_2024_2017_MOESM1_ESM.zip › Sup5_AS_GO.zip!/Sup5_AS_GO/ras/CLPDCA_vs_Sham/Ras_GO_analysis/Ras_GO_enrichment_web_P.html]

GO enrichment analyze


# GO enrichment analyze result


| Term | Database | ID | Input number (501) | Background number (20961) | P-Value | Corrected P-Value | Input | Hyperlink |
| --- | --- | --- | --- | --- | --- | --- | --- | --- |
| mRNA processing | Gene Ontology | GO:0006397 | 23 (4.59%) | 351 (1.67%) | 1.4004611121e-05 | 0.00215671011263 | Srsf5  Tsen34  Srsf2  Hnrnpu  Ptbp3  Ptbp1  Cdk12  Hnrnpa1  Gemin6  Zc3h13  Hsf1  Scaf1  Nudt21  Thoc7  Tardbp  Srrm1  Hnrnpll  Thoc2l  Tdrd3  Snrpa1  Rbm25  Sf3a2  Cpsf7 | web link |
| phospholipid biosynthetic process | Gene Ontology | GO:0008654 | 8 (1.60%) | 59 (0.28%) | 7.65856925529e-05 | 0.0033477761656 | Tmem38b  Pcyt2  Cept1  Agpat3  Gnpat  Crls1  Abhd5  Cdipt | web link |
| regulation of apoptotic process | Gene Ontology | GO:0042981 | 15 (2.99%) | 196 (0.94%) | 7.86432922277e-05 | 0.0033477761656 | Acer2  Dnaja3  Card19  Esr1  Fas  Bcl2l1  Madd  Apex1  Cflar  Rbm25  Nf2  Anp32a  Clu  Gm45808  Tardbp | web link |
| negative regulation of transcription by RNA polymerase II | Gene Ontology | GO:0000122 | 41 (8.18%) | 913 (4.36%) | 8.69552250806e-05 | 0.0033477761656 | Ube2i  Tsg101  Fnip1  Deaf1  Hnrnpu  Thra  Ezh2  Vegfa  Fbp1  Ctbp1  Rsl1  Kdm5a  Smarcc2  Foxp1  Foxk2  Esr1  Spop  Cpeb3  Tmprss6  Hsf1  Map2k5  Smarca4  Nfib  Zfp932  Zbtb14  Zfp973  Ifi27  Timeless  Hnf1b  Eed  Dnaja3  Uxt  Cops2  Mapk15  Cggbp1  Zfpm1  Zfp131  2610044O15Rik8  Ppp1r13l  Ncor2  Ncor1 | web link |
| protein ubiquitination | Gene Ontology | GO:0016567 | 28 (5.59%) | 545 (2.60%) | 0.000135760643546 | 0.00418142782123 | Ube2i  Ube2d3  Ubr3  Ankib1  Rnf149  Klhl13  Ube2j2  Spop  Rnf181  Dcaf11  Marchf8  Uba52  Mycbp2  Ubc  Rnf215  Rnf216  Zswim8  Rnf146  Btrc  Smurf1  Rnf123  Trim26  Xiap  Fbxo6  Birc6  Pja1  Pja2  Cdc42 | web link |
| regulation of cell cycle | Gene Ontology | GO:0051726 | 12 (2.40%) | 145 (0.69%) | 0.000194297790299 | 0.00498697661766 | Srsf5  Madd  Gas2l1  Cdk12  Btrc  Cdk16  Xiap  Nf2  Ctbp1  Tardbp  Tfdp1  Tsc1 | web link |
| iron ion homeostasis | Gene Ontology | GO:0055072 | 6 (1.20%) | 39 (0.19%) | 0.000302101352173 | 0.00541864133155 | Tfr2  Slc11a2  Slc40a1  Tmprss6  Neo1  Steap3 | web link |
| lipid metabolic process | Gene Ontology | GO:0006629 | 27 (5.39%) | 544 (2.60%) | 0.000308152274333 | 0.00541864133155 | Acer2  Cept1  Sc5d  Angptl4  Agpat3  Sgms2  Sptssa  Echdc2  Adipor2  St3gal4  Aldh3a2  Gstm4  Crls1  Arv1  Mvk  Gba2  Hsd17b6  Cdipt  Aasdh  Pcyt2  Enpp2  Elovl5  Pnpla6  Fdxr  Akr1c6  Mid1ip1  Abhd5 | web link |
| RNA splicing | Gene Ontology | GO:0008380 | 17 (3.39%) | 272 (1.30%) | 0.000316673844051 | 0.00541864133155 | Srsf5  Srsf2  Cdk12  Hnrnpa1  Gemin6  Hnrnpu  Snrpa1  Zc3h13  Rbm25  Srsf11  Scaf1  Sf3a2  Ptbp3  Ptbp1  Thoc7  Zfp638  Tardbp | web link |
| endosome to lysosome transport | Gene Ontology | GO:0008333 | 6 (1.20%) | 43 (0.21%) | 0.000520772233687 | 0.00801989239878 | Tsg101  Fam160a2  Trak1  Kif13a  Bin1  Gprasp1 | web link |
| oxidation-reduction process | Gene Ontology | GO:0055114 | 26 (5.19%) | 546 (2.60%) | 0.000732629591225 | 0.00979153327142 | Cyp4f13  Idh1  Ero1b  Dhrs3  Akr1e1  Dhtkd1  Sc5d  Hsd3b2  Ctbp1  Paox  D2hgdh  Ndufs2  Cyp2r1  Idh3g  Aldh3a2  Cyp3a59  Fdxr  P4ha1  Adhfe1  Cyp4a31  Hsd17b6  Cyp4b1  Pcyox1  Rdh11  Aldh16a1  Akr1c6 | web link |
| negative regulation of translation | Gene Ontology | GO:0017148 | 8 (1.60%) | 82 (0.39%) | 0.000762976618552 | 0.00979153327142 | Gigyf2  Cnot10  Cpeb3  Gm45713  Xrn1  Pcif1  Mvk  Tsc1 | web link |
| methylation | Gene Ontology | GO:0032259 | 12 (2.40%) | 184 (0.88%) | 0.00162862663901 | 0.0192929617236 | Kmt2c  Setdb1  Pcmtd1  Mettl17  Mtr  Comt  Setd4  Ezh2  Pcif1  Nsd1  Etfbkmt  Eef1akmt2 | web link |
| mRNA transport | Gene Ontology | GO:0051028 | 8 (1.60%) | 94 (0.45%) | 0.00185914152591 | 0.020450556785 | Hsf1  Zc3h11a  Poldip3  Hnrnpa1  Nup37  G3bp2  Thoc7  Eif5a | web link |
| liver development | Gene Ontology | GO:0001889 | 8 (1.60%) | 97 (0.46%) | 0.00226884042165 | 0.0220645900812 | Srsf5  Smarca4  Egfr  Hnf1b  Nf1  Kras  Proc  Cadm1 | web link |
| negative regulation of cysteine-type endopeptidase activity involved in apoptotic process | Gene Ontology | GO:0043154 | 7 (1.40%) | 77 (0.37%) | 0.00243034426055 | 0.0220645900812 | Bcl2l1  Dnaja3  Fnip1  Cflar  Xiap  Vegfa  Map2k5 | web link |
| mitochondrial respiratory chain complex I assembly | Gene Ontology | GO:0032981 | 6 (1.20%) | 58 (0.28%) | 0.00255885644965 | 0.0220645900812 | Taz  Ndufaf4  Ndufs2  Ndufaf1  Ndufaf2  Dmac2 | web link |
| response to cytokine | Gene Ontology | GO:0034097 | 7 (1.40%) | 78 (0.37%) | 0.00261619007973 | 0.0220645900812 | Bcl2l1  Cfh  Ghr  Itih4  Serpina1a  Lifr  Mapkapk2 | web link |
| translation | Gene Ontology | GO:0006412 | 16 (3.19%) | 303 (1.45%) | 0.00272225462041 | 0.0220645900812 | Mrpl36  Rpl38  Eif4g3  Eef1d  Egfr  Rpl35a  Cpeb3  Mrps2  Mrps17  Rpl15  Uba52  Gm45713  Mtif2  Mettl17  Mars1  Eif5a | web link |
| skeletal muscle tissue development | Gene Ontology | GO:0007519 | 6 (1.20%) | 60 (0.29%) | 0.00304105041468 | 0.023416088193 | Foxp1  Taz  Myl6  Cflar  Nf1  Flnb | web link |
| negative regulation of transcription, DNA-templated | Gene Ontology | GO:0045892 | 26 (5.19%) | 622 (2.97%) | 0.00439651398301 | 0.0322411025421 | Ube2i  Sbno2  Tle2  Deaf1  Thra  Ezh2  Ctbp1  Rsl1  Smarcc2  Mphosph8  Foxp1  Foxk2  Tmprss6  Hmga1  Smarca4  Zbtb14  Mterf3  Btrc  Timeless  Sfmbt1  Set  Cops2  Bahd1  Phc2  Ncor2  Ncor1 | web link |
| mitochondrion organization | Gene Ontology | GO:0007005 | 7 (1.40%) | 87 (0.42%) | 0.00482021222715 | 0.0337414855901 | Dnaja3  Gabpb1  Cfh  Taz  Mtx3  Sharpin  Nrf1 | web link |
| steroid metabolic process | Gene Ontology | GO:0008202 | 8 (1.60%) | 112 (0.53%) | 0.00548296578886 | 0.03618819073 | Arv1  Cyp3a59  Sc5d  Fdxr  Akr1c6  Mvk  Gba2  Hsd17b6 | web link |
| chromatin organization | Gene Ontology | GO:0006325 | 15 (2.99%) | 298 (1.42%) | 0.00563971803585 | 0.03618819073 | Smarcc2  Kmt2c  Emsy  Tdrd3  Wac  Hnrnpu  Kdm5a  Sfmbt1  Ezh2  Naa60  Nsd1  Eed  0610010K14Rik  Smarca4  Setdb1 | web link |
| positive regulation of cell differentiation | Gene Ontology | GO:0045597 | 5 (1.00%) | 49 (0.23%) | 0.00613763884636 | 0.0378078552936 | Clu  Ppp1r13l  Smarca4  Nf2  Ghr | web link |
| neuron projection morphogenesis | Gene Ontology | GO:0048812 | 6 (1.20%) | 72 (0.34%) | 0.00747356051888 | 0.0442664738426 | Mink1  Trio  Egfr  Lifr  Dtnbp1  Clu | web link |
| autophagy | Gene Ontology | GO:0006914 | 10 (2.00%) | 172 (0.82%) | 0.0085511985216 | 0.0487735026788 | Atg4c  Dram2  Stk11  Retreg1  Rubcn  Tbc1d5  Atg13  Tm9sf1  Tmem208  Sec16b | web link |
| protein autophosphorylation | Gene Ontology | GO:0046777 | 10 (2.00%) | 174 (0.83%) | 0.00923608547729 | 0.0490467987415 | Mink1  Cdk12  Stk16  Stk11  Egfr  Uhmk1  Fgfr3  Mapk15  Clk4  Mapkapk2 | web link |
| actin cytoskeleton organization | Gene Ontology | GO:0030036 | 10 (2.00%) | 174 (0.83%) | 0.00923608547729 | 0.0490467987415 | Pdlim5  Coro1b  Mtss1  Nf2  Nf1  Ablim2  Flnb  Cdc42  Inf2  Kras | web link |
| regulation of growth | Gene Ontology | GO:0040008 | 6 (1.20%) | 77 (0.37%) | 0.0102711605994 | 0.0524999225201 | Bcl2l1  Tsg101  Selenop  Safb  Vegfa  Igfbp4 | web link |
| proteasome-mediated ubiquitin-dependent protein catabolic process | Gene Ontology | GO:0043161 | 10 (2.00%) | 178 (0.85%) | 0.0107309650879 | 0.0524999225201 | Smurf1  Ube2i  Spop  Btrc  Ube2d3  Dcaf11  Pcbp2  Rnf216  Zswim8  Sharpin | web link |
| negative regulation of protein kinase activity | Gene Ontology | GO:0006469 | 6 (1.20%) | 78 (0.37%) | 0.0109090748094 | 0.0524999225201 | Dnaja3  Cep43  Prkar1a  Nf1  Dtnbp1  Nf2 | web link |
| positive regulation of stress fiber assembly | Gene Ontology | GO:0051496 | 5 (1.00%) | 58 (0.28%) | 0.012391437134 | 0.0561259211362 | Carmil1  Cdc42  Nf2  Tpm1  Tsc1 | web link |
| sphingolipid metabolic process | Gene Ontology | GO:0006665 | 5 (1.00%) | 58 (0.28%) | 0.012391437134 | 0.0561259211362 | Acer2  Gba2  Arv1  Sptssa  Sgms2 | web link |
| cellular response to amino acid stimulus | Gene Ontology | GO:0071230 | 5 (1.00%) | 59 (0.28%) | 0.0132802581344 | 0.0584331357915 | Bcl2l1  Nsmf  Hsf1  Egfr  Cpeb3 | web link |
| wound healing | Gene Ontology | GO:0042060 | 7 (1.40%) | 106 (0.51%) | 0.0137011239519 | 0.0586103635722 | Tsku  Sdc1  Egfr  Tpm1  Coro1b  Cflar  Nf1 | web link |
| circadian regulation of gene expression | Gene Ontology | GO:0032922 | 5 (1.00%) | 60 (0.29%) | 0.0142110951999 | 0.0591488827238 | Hnf1b  Ppp1ca  Hnrnpu  Mycbp2  Kdm5a | web link |
| regulation of circadian rhythm | Gene Ontology | GO:0042752 | 5 (1.00%) | 61 (0.29%) | 0.0151847531752 | 0.0599603074099 | Ezh2  Btrc  Ppp1ca  Timeless  Tardbp | web link |
| glutathione metabolic process | Gene Ontology | GO:0006749 | 5 (1.00%) | 61 (0.29%) | 0.0151847531752 | 0.0599603074099 | Gstp3  Idh1  Mgst1  Gstm4  Gsta3 | web link |
| regulation of translation | Gene Ontology | GO:0006417 | 8 (1.60%) | 135 (0.64%) | 0.0159652955835 | 0.0614663879964 | Tcof1  Rpl38  Ppp1ca  Poldip3  Cnot10  Cpeb3  Gm45713  Tsc1 | web link |
| transcription by RNA polymerase II | Gene Ontology | GO:0006366 | 7 (1.40%) | 110 (0.52%) | 0.0165102264911 | 0.0620140214545 | Thra  Taf13  Scaf1  Gtf2a2  Gtf2i  Polr2h  Tfdp1 | web link |
| regulation of protein stability | Gene Ontology | GO:0031647 | 6 (1.20%) | 87 (0.42%) | 0.0179578163664 | 0.0658453266767 | Asph  Rnf149  Asgr2  Nf2  Tardbp  Kras | web link |
| apoptotic process | Gene Ontology | GO:0006915 | 22 (4.39%) | 567 (2.71%) | 0.0184392495562 | 0.0660382425965 | Dram2  Coa8  Stk11  Tmem219  Tmbim4  Cadm1  Bcl2l1  Fas  Ube2d3  Rnf216  Madd  Dido1  Dnaja3  Uxt  Ahcyl1  Birc6  Cflar  Xiap  Rbm25  Ppp1r13l  Bnip2  Eif5a | web link |
| chromosome segregation | Gene Ontology | GO:0007059 | 6 (1.20%) | 88 (0.42%) | 0.0188963087659 | 0.0661370806807 | Ube2i  Pmf1  Nup37  Naa60  Nde1  Mms19 | web link |
| rhythmic process | Gene Ontology | GO:0048511 | 8 (1.60%) | 141 (0.67%) | 0.0201978479769 | 0.0691215241875 | Ppp1ca  Btrc  Timeless  Hnrnpu  Ezh2  Mycbp2  Kdm5a  Tardbp | web link |
| phosphorylation | Gene Ontology | GO:0016310 | 24 (4.79%) | 641 (3.06%) | 0.0209086489247 | 0.0699985203132 | Stk11  Stk16  Pkdcc  Mapkapk2  Nme4  Mvk  Csnk1a1  Pi4ka  Cdk12  Cdk16  Ppip5k2  Mapk15  Sgms2  Map2k5  Clk4  Fgfr3  Mink1  Uhmk1  Cask  Xylb  Trio  Egfr  Rps6kb2  Mast3 | web link |
| protein phosphorylation | Gene Ontology | GO:0006468 | 23 (4.59%) | 616 (2.94%) | 0.0240841327361 | 0.0789139668375 | Hsf1  Stk11  Stk16  Pkdcc  Mapkapk2  Csnk1a1  Cdk12  Cdk16  Mapk15  Gak  Map2k5  Clk4  Fgfr3  Mink1  Uhmk1  Cask  Phkb  Trio  Cops2  Egfr  Rps6kb2  Mast3  Prkar1a | web link |
| response to estradiol | Gene Ontology | GO:0032355 | 7 (1.40%) | 120 (0.57%) | 0.0252677815288 | 0.0810674657381 | Hsf1  Ghr  Esr1  Egfr  Mapk15  Ezh2  Ncor2 | web link |
| Golgi organization | Gene Ontology | GO:0007030 | 6 (1.20%) | 95 (0.45%) | 0.0264136508711 | 0.0830143313091 | Lysmd3  Trappc8  Csnk1a1  Gak  Sec16b  Cdc42 | web link |
| ubiquitin-dependent ERAD pathway | Gene Ontology | GO:0030433 | 5 (1.00%) | 71 (0.34%) | 0.0274331235615 | 0.0837714162281 | Psmc6  Dnajb12  Derl3  Fbxo6  Ube2j2 | web link |
| negative regulation of cell growth | Gene Ontology | GO:0030308 | 7 (1.40%) | 123 (0.59%) | 0.028416674571 | 0.0837714162281 | Adipor2  Stk11  Dcun1d3  Fbp1  Gas2l1  Smarca4  Nf2 | web link |
| positive regulation of MAPK cascade | Gene Ontology | GO:0043410 | 7 (1.40%) | 123 (0.59%) | 0.028416674571 | 0.0837714162281 | Adra1a  Vegfa  Bcar3  Igfbp4  Fgfr3  Bnip2  Cdc42 | web link |
| positive regulation of protein ubiquitination | Gene Ontology | GO:0031398 | 5 (1.00%) | 72 (0.34%) | 0.0289226431916 | 0.0837714162281 | Mapk15  Xiap  Septin4  Mycbp2  Dnaja3 | web link |
| protein stabilization | Gene Ontology | GO:0050821 | 9 (1.80%) | 180 (0.86%) | 0.0294353371286 | 0.0837714162281 | Cdc37l1  Dnaja3  Coa8  Ifi30  Ncln  Tcp1  Clu  Irgm2  Tsc1 | web link |
| cholesterol metabolic process | Gene Ontology | GO:0008203 | 6 (1.20%) | 98 (0.47%) | 0.0301665307626 | 0.0837714162281 | Tsku  Fdxr  Arv1  Mvk  Gba2  Cln8 | web link |
| positive regulation of epithelial cell proliferation | Gene Ontology | GO:0050679 | 5 (1.00%) | 73 (0.35%) | 0.0304623331739 | 0.0837714162281 | Vegfa  Foxp1  Egfr  Map2k5  Esr1 | web link |
| negative regulation of DNA-binding transcription factor activity | Gene Ontology | GO:0043433 | 5 (1.00%) | 74 (0.35%) | 0.032052600065 | 0.0851051794829 | Spop  Thra  Ezh2  Zfp932  Esr1 | web link |
| roof of mouth development | Gene Ontology | GO:0060021 | 5 (1.00%) | 74 (0.35%) | 0.032052600065 | 0.0851051794829 | Asph  Pkdcc  Zfp950  Plekha1  Dhrs3 | web link |
| negative regulation of neuron death | Gene Ontology | GO:1901215 | 5 (1.00%) | 75 (0.36%) | 0.0336938176241 | 0.0861172612906 | Hsf1  Dtnbp1  Vegfa  Ghr  Esr1 | web link |
| blood coagulation | Gene Ontology | GO:0007596 | 5 (1.00%) | 75 (0.36%) | 0.0336938176241 | 0.0861172612906 | F10  Proc  Enpp4  Habp2  Dtnbp1 | web link |
| negative regulation of apoptotic process | Gene Ontology | GO:0043066 | 21 (4.19%) | 570 (2.72%) | 0.034111382719 | 0.0861172612906 | Bcl2l1  Dnaja3  Cln8  Slc40a1  Proc  Ndufaf4  Prlr  Birc6  Higd1a  Egfr  Lims2  Hnf1b  Cflar  Xiap  Angptl4  Vegfa  Fas  Clu  Tmbim4  Smarca4  Eif5a | web link |
| endoplasmic reticulum to Golgi vesicle-mediated transport | Gene Ontology | GO:0006888 | 6 (1.20%) | 102 (0.49%) | 0.035690132634 | 0.0886496842843 | Sec22c  Trappc8  Cope  Yif1b  Sec16b  Sec31a | web link |
| response to nutrient | Gene Ontology | GO:0007584 | 5 (1.00%) | 77 (0.37%) | 0.0371304355209 | 0.0903993869339 | Abcg8  Hsf1  Bckdhb  Adipor2  Gnpat | web link |
| kidney development | Gene Ontology | GO:0001822 | 8 (1.60%) | 159 (0.76%) | 0.0375685763881 | 0.0903993869339 | Cfh  Zbtb14  Timeless  Hnf1b  Vegfa  Zfp950  Cyp4a31  Tsc1 | web link |
| ossification | Gene Ontology | GO:0001503 | 6 (1.20%) | 104 (0.50%) | 0.0386801440207 | 0.0916421873721 | Sbno2  Tmem38b  Rpl38  Sun1  Thra  Pkdcc | web link |
| cellular response to drug | Gene Ontology | GO:0035690 | 5 (1.00%) | 79 (0.38%) | 0.0407745200652 | 0.0951405468189 | Hsf1  Acer2  Egfr  Fbp1  Rnf149 | web link |
| immune system process | Gene Ontology | GO:0002376 | 18 (3.59%) | 481 (2.29%) | 0.0417299869208 | 0.0959166863552 | Ido2  Erap1  Masp1  Cfh  Cr1l  Trim26  C4bp  Rubcn  Ifi30  1810058I24Rik  Marchf8  Sema4a  Pcbp2  G3bp2  AI182371  Nlrc5  Irf9  Cadm1 | web link |
| positive regulation of DNA-binding transcription factor activity | Gene Ontology | GO:0051091 | 6 (1.20%) | 107 (0.51%) | 0.0434561081452 | 0.0981813377272 | Ube2i  Hsf1  Esr1  Trim26  Smarca4  Tfdp1 | web link |
| ubiquitin-dependent protein catabolic process | Gene Ontology | GO:0006511 | 11 (2.20%) | 256 (1.22%) | 0.044601765218 | 0.0981813377272 | Smurf1  Spop  Birc6  Btrc  Ube2d3  Ankib1  Rnf149  Rnf215  Eloc  Ubr3  Rnf146 | web link |
| forebrain development | Gene Ontology | GO:0030900 | 5 (1.00%) | 81 (0.39%) | 0.0446278807851 | 0.0981813377272 | Nde1  Nfib  Foxp1  Smarca4  Ncor2 | web link |
| positive regulation of transcription by RNA polymerase II | Gene Ontology | GO:0045944 | 38 (7.58%) | 1201 (5.73%) | 0.0478580103696 | 0.103804698548 | Hsf1  Sbno2  Tfr2  Gabpb1  Zbtb7c  Deaf1  Hnrnpu  Thra  Apex1  Vegfa  Tfdp1  Slc40a1  Foxp1  Foxk2  Esr1  Safb  Tmprss6  Hmga1  Zfp62  Gtf2a2  Nlrc5  Smarca4  Nrf1  Nfib  Nfia  Map2k5  Stk16  Cask  Hnf1b  Pcbp2  Ablim2  Gtf2i  Zfp467  Fubp3  Zfpm1  Egfr  Zfp131  Ssbp4 | web link |
| cell differentiation | Gene Ontology | GO:0030154 | 30 (5.99%) | 918 (4.38%) | 0.0523784351248 | 0.111738768397 | Tsg101  Sbno2  Stk11  Hnrnpu  Thra  Nde1  Pkdcc  Vegfa  Tmem176b  Ptbp3  Ctbp1  Flnb  Bin1  Cadm1  Strbp  Sun1  Cdk16  Nudt21  Zfp932  Sfmbt1  Septin4  Sema4a  Ndrg2  Sema4g  Smurf1  Ngef  Zfpm1  Egfr  Abhd5  Cdc42 | web link |
| positive regulation of cold-induced thermogenesis | Gene Ontology | GO:0120162 | 5 (1.00%) | 85 (0.41%) | 0.0529670785258 | 0.111738768397 | Prlr  Hsf1  Adipor2  Vegfa  Thra | web link |
| lung development | Gene Ontology | GO:0030324 | 6 (1.20%) | 113 (0.54%) | 0.0540740661158 | 0.112532515971 | Nfib  Foxp1  Ppp1ca  Egfr  Timeless  Vegfa | web link |
| hippocampus development | Gene Ontology | GO:0021766 | 5 (1.00%) | 86 (0.41%) | 0.055184072589 | 0.113311295716 | Ezh2  Tsku  Nf2  Hsd3b2  Tsc1 | web link |
| cellular response to DNA damage stimulus | Gene Ontology | GO:0006974 | 17 (3.39%) | 470 (2.24%) | 0.0604658790448 | 0.122522965433 | Acer2  Hsf1  Cyren  Hmces  Timeless  Stk11  Wac  Ube2d3  Apex1  Mapk15  Xiap  Emsy  Fbxo6  Swi5  Nabp2  Mapkapk2  Mms19 | web link |
| MAPK cascade | Gene Ontology | GO:0000165 | 5 (1.00%) | 89 (0.42%) | 0.0621522510459 | 0.124304502092 | Hsf1  Mapk15  Nf1  Map2k5  Mink1 | web link |
| nervous system development | Gene Ontology | GO:0007399 | 15 (2.99%) | 410 (1.96%) | 0.0690473930964 | 0.136324340216 | Smarcc2  Sema4g  Tsku  Ngef  Deaf1  Neo1  Vegfa  Gfra1  Cdc42  Nde1  Sema4a  Ndrg2  Cln8  Smarca4  Tsc1 | web link |
| positive regulation of transcription, DNA-templated | Gene Ontology | GO:0045893 | 23 (4.59%) | 691 (3.30%) | 0.0699872084826 | 0.13643076084 | Tsg101  Asph  Wac  Kdm5a  Tfdp1  Smarcc2  Foxp1  Foxk2  Pmf1  Esr1  Hmga1  Nsd1  Smarca4  Nfib  Nfia  Deaf1  Btrc  Hnf1b  Ing1  Mms19  Tdrd3  Fubp3  Egfr | web link |
| post-embryonic development | Gene Ontology | GO:0009791 | 5 (1.00%) | 94 (0.45%) | 0.0748170803173 | 0.141445358089 | Ppp1r13l  Selenop  Zfp950  Plekha1  Gigyf2 | web link |
| cellular response to hypoxia | Gene Ontology | GO:0071456 | 6 (1.20%) | 123 (0.59%) | 0.0749783867741 | 0.141445358089 | Hp1bp3  Cr1l  Higd1a  Cflar  Vegfa  Slc29a1 | web link |
| positive regulation of cell population proliferation | Gene Ontology | GO:0008284 | 20 (3.99%) | 590 (2.81%) | 0.0753150608006 | 0.141445358089 | Acer2  Bcl2l1  Ndufaf4  Prlr  Birc6  Chtop  Enpp2  Cep43  Egfr  Mapk15  Hsf1  Ezh2  Vegfa  Lifr  Clu  Eapp  Kras  Smarca4  Fgfr3  Eif5a | web link |
| positive regulation of peptidyl-serine phosphorylation | Gene Ontology | GO:0033138 | 5 (1.00%) | 95 (0.45%) | 0.077506159731 | 0.143806609621 | Irgm2  Fnip1  Vegfa  Egfr  Bcar3 | web link |
| regulation of cell population proliferation | Gene Ontology | GO:0042127 | 8 (1.60%) | 190 (0.91%) | 0.0865746029561 | 0.158720105419 | Hp1bp3  Birc6  Egfr  Ezh2  Nf2  Nf1  Fas  Clu | web link |
| nucleic acid phosphodiester bond hydrolysis | Gene Ontology | GO:0090305 | 6 (1.20%) | 129 (0.62%) | 0.0894421185716 | 0.162048073647 | Meiob  Tsen34  Enpp2  Apex1  Xrn1  Rexo4 | web link |
| response to bacterium | Gene Ontology | GO:0009617 | 8 (1.60%) | 192 (0.92%) | 0.0906214497825 | 0.162275619378 | Ociad2  Nfib  Adipor2  Prlr  Cflar  Nlrc5  Baiap2  Irgm2 | web link |
| transcription, DNA-templated | Gene Ontology | GO:0006351 | 5 (1.00%) | 100 (0.48%) | 0.0917173030692 | 0.162350168651 | Fubp3  Ppp1r13l  Polr2h  Dido1  Ablim2 | web link |
| in utero embryonic development | Gene Ontology | GO:0001701 | 11 (2.20%) | 293 (1.40%) | 0.0946670186521 | 0.165667282641 | Hsf1  Bcl2l1  Ly6e  Foxp1  Cr1l  Chtop  Tpm1  Vegfa  Ubr3  Smarca4  Ncor2 | web link |
| small GTPase mediated signal transduction | Gene Ontology | GO:0007264 | 5 (1.00%) | 102 (0.49%) | 0.0977525309526 | 0.166988468948 | Dnaja3  Dock9  Cdc42  Bcar3  G3bp2 | web link |
| protein polyubiquitination | Gene Ontology | GO:0000209 | 7 (1.40%) | 164 (0.78%) | 0.0991468700099 | 0.166988468948 | Smurf1  Ube2j2  Spop  Btrc  Ube2d3  Marchf8  Ankib1 | web link |
| cell division | Gene Ontology | GO:0051301 | 13 (2.59%) | 365 (1.74%) | 0.100562902565 | 0.166988468948 | Ube2i  Tsg101  Klhl13  Csnk1a1  Pmf1  Ppp1ca  Birc6  Timeless  Hnrnpu  Pard6a  Septin4  Nup37  Nde1 | web link |
| positive regulation of canonical Wnt signaling pathway | Gene Ontology | GO:0090263 | 5 (1.00%) | 103 (0.49%) | 0.100843685793 | 0.166988468948 | Xiap  Thra  Egfr  Ppp2r3a  Rnf146 | web link |
| multicellular organism growth | Gene Ontology | GO:0035264 | 5 (1.00%) | 103 (0.49%) | 0.100843685793 | 0.166988468948 | Pkdcc  Ppp1r13l  Zfp950  Plekha1  Gigyf2 | web link |
| protein folding | Gene Ontology | GO:0006457 | 5 (1.00%) | 106 (0.51%) | 0.110405702839 | 0.180167582077 | Cdc37l1  Dnaja3  Tcp1  Ahsa2  Ppig | web link |
| positive regulation of protein phosphorylation | Gene Ontology | GO:0001934 | 9 (1.80%) | 235 (1.12%) | 0.112032806649 | 0.180167582077 | Fnip1  Adipor2  Fas  Egfr  Mapk15  Vegfa  Dtnbp1  Irgm2  Kras | web link |
| cellular response to insulin stimulus | Gene Ontology | GO:0032869 | 5 (1.00%) | 107 (0.51%) | 0.113687545716 | 0.180167582077 | Srsf5  Cflar  Slc39a14  Mars1  Ghr | web link |
| regulation of transcription by RNA polymerase II | Gene Ontology | GO:0006357 | 43 (8.58%) | 1489 (7.10%) | 0.11374405971 | 0.180167582077 | Hsf1  Zbtb7c  Gm28557  Deaf1  Thra  Zkscan1  Ezh2  Vegfa  Zfp141  Rsl1  Tfdp1  Smarcc2  Foxp1  Foxk2  Esr1  Irf9  Safb  Zfp950  Zfp874a  Zfp995  Nrf1  Nfib  Nfia  Zfp932  2010315B03Rik  Zbtb14  Zfp973  Cask  Hnf1b  Zfp235  Ctdsp1  Phf20l1  Repin1  Gm14403  Uxt  Zfp467  Cggbp1  Zfpm1  Zfp131  Zfp946  2610044O15Rik8  Ppp1r13l  Zfp62 | web link |
| regulation of catalytic activity | Gene Ontology | GO:0050790 | 10 (2.00%) | 270 (1.29%) | 0.114652097685 | 0.180167582077 | Plekhg3  Madd  Dock9  Ngef  Sil1  Trio  Ppp4r1  Mycbp2  Arhgef12  Bcar3 | web link |
| exocytosis | Gene Ontology | GO:0006887 | 5 (1.00%) | 108 (0.52%) | 0.117015750791 | 0.180204256218 | Scamp5  Cdk16  Exoc7  Washc1  Snap23 | web link |
| female pregnancy | Gene Ontology | GO:0007565 | 5 (1.00%) | 108 (0.52%) | 0.117015750791 | 0.180204256218 | Cr1l  Adipor2  Comt  Slc38a3  Kras | web link |
| peptidyl-tyrosine phosphorylation | Gene Ontology | GO:0018108 | 6 (1.20%) | 142 (0.68%) | 0.125543208262 | 0.191422317547 | Egfr  Stk16  Pkdcc  Map2k5  Clk4  Fgfr3 | web link |
| negative regulation of cell population proliferation | Gene Ontology | GO:0008285 | 13 (2.59%) | 392 (1.87%) | 0.148083190699 | 0.223576582035 | Hsf1  Nfib  Zbtb7c  Dnaja3  Stk11  Asph  Nudt6  Hmga1  Nf2  Nf1  Ing1  Tsg101  Tsc1 | web link |
| cell migration | Gene Ontology | GO:0016477 | 9 (1.80%) | 255 (1.22%) | 0.158984573517 | 0.237439993507 | Carmil1  Sdc1  Coro1b  Vegfa  Nde1  Gfra1  Lcp1  Cdc42  Asap3 | web link |
| sodium ion transport | Gene Ontology | GO:0006814 | 5 (1.00%) | 120 (0.57%) | 0.160349086524 | 0.237439993507 | Slc38a10  Slc38a7  Slc38a3  Slc17a2  Fxyd1 | web link |
| cell-cell adhesion | Gene Ontology | GO:0098609 | 6 (1.20%) | 155 (0.74%) | 0.167624793828 | 0.245849697615 | Mpzl2  Ppp1ca  Egfr  Lims2  Fndc3a  Cdc42 | web link |
| proteolysis | Gene Ontology | GO:0006508 | 20 (3.99%) | 664 (3.17%) | 0.172740618191 | 0.25052879648 | Cflar  Tfr2  Sppl2b  Hmces  Desi1  Rnpepl1  Metap1d  Pgpep1l  Masp1  Tmprss6  Ncln  Atg4c  Adamts10  Habp2  Tysnd1  F10  Josd2  Proc  Erap1  Abhd5 | web link |
| endocytosis | Gene Ontology | GO:0006897 | 8 (1.60%) | 226 (1.08%) | 0.175152231765 | 0.25052879648 | Ghr  Snx10  Enpp2  Rubcn  Rin2  Asgr2  Bin1  Cdc42 | web link |
| negative regulation of cell migration | Gene Ontology | GO:0030336 | 5 (1.00%) | 124 (0.59%) | 0.176063965567 | 0.25052879648 | Mapk15  Afdn  Nf1  Tpm1  Miip | web link |
| heart development | Gene Ontology | GO:0007507 | 9 (1.80%) | 263 (1.25%) | 0.180072605253 | 0.25052879648 | Pdlim5  Foxp1  Adipor2  Map2k5  Zfpm1  Taz  Prkar1a  Nf1  Smarca4 | web link |
| protein transport | Gene Ontology | GO:0015031 | 19 (3.79%) | 631 (3.01%) | 0.180558013029 | 0.25052879648 | Tsg101  Sil1  Fam160a2  Scamp5  Exoc7  Snap23  Cope  Sys1  Tbc1d5  Nup37  Atg4c  Pkdcc  Yif1b  Sec22c  Snx10  Mtx3  Sec16b  Sec31a  Eif5a | web link |
| fatty acid metabolic process | Gene Ontology | GO:0006631 | 7 (1.40%) | 193 (0.92%) | 0.18057595071 | 0.25052879648 | Adipor2  Aldh3a2  Elovl5  Gnpat  Aasdh  Echdc2  Abhd5 | web link |
| lipid transport | Gene Ontology | GO:0006869 | 5 (1.00%) | 130 (0.62%) | 0.200664060141 | 0.275913082694 | Nme4  Abcg8  Arv1  Apoc3  Apol9b | web link |
| spermatogenesis | Gene Ontology | GO:0007283 | 14 (2.79%) | 458 (2.19%) | 0.209398205311 | 0.283396744544 | Bcl2l1  Hsf1  Strbp  Sun1  Stk11  Cdk16  Snrpa1  Hmga1  Sfmbt1  Septin4  Plekha1  Phc2  Kdm5a  Cadm1 | web link |
| positive regulation of GTPase activity | Gene Ontology | GO:0043547 | 10 (2.00%) | 310 (1.48%) | 0.209787200506 | 0.283396744544 | Arhgef12  Arap2  Afdn  Rin2  Ezh2  Nf1  Rgs3  Bin1  Bcar3  Asap3 | web link |
| protein dephosphorylation | Gene Ontology | GO:0006470 | 6 (1.20%) | 169 (0.81%) | 0.218563459699 | 0.292684980815 | Btrc  Ppp1ca  Ppp2r3a  Stk11  Mtmr4  Ctdsp1 | web link |
| Wnt signaling pathway | Gene Ontology | GO:0016055 | 7 (1.40%) | 205 (0.98%) | 0.220596975857 | 0.292861502431 | Porcn  Csnk1a1  Tle2  Btrc  Xiap  Ndrg2  Rnf146 | web link |
| potassium ion transport | Gene Ontology | GO:0006813 | 5 (1.00%) | 138 (0.66%) | 0.235113254644 | 0.306842722163 | Fxyd1  Slc12a7  Tmem175  Tmem38b  Tsc1 | web link |
| glucose homeostasis | Gene Ontology | GO:0042593 | 5 (1.00%) | 138 (0.66%) | 0.235113254644 | 0.306842722163 | Spop  Ero1b  Stk11  Adipor2  Ncor2 | web link |
| dephosphorylation | Gene Ontology | GO:0016311 | 6 (1.20%) | 178 (0.85%) | 0.253745180064 | 0.328376115377 | Mtmr14  Ppp1ca  Fbp1  Mtmr4  Ctdsp1  Nt5dc1 | web link |
| brain development | Gene Ontology | GO:0007420 | 8 (1.60%) | 258 (1.23%) | 0.277057688384 | 0.355557366759 | Nfib  Slc38a3  Selenop  Septin4  Nf2  Nf1  Baiap2  Sharpin | web link |
| regulation of transcription, DNA-templated | Gene Ontology | GO:0006355 | 37 (7.39%) | 1403 (6.69%) | 0.289671687251 | 0.368218212349 | Hsf1  Sbno2  Tle2  Nfia  Thra  Zkscan1  Zfp141  Rsl1  Tfdp1  Gm28557  Foxp1  Foxk2  Esr1  Irf9  Safb  Hmga1  Zfp950  Gm14403  Mterf3  Smarca4  Zfp995  Zfp874a  Nfib  Emsy  Zfp932  2010315B03Rik  Zfp973  Sfmbt1  Zfp235  Phf20l1  Gtf2i  Kmt2c  Hp1bp3  Zfp467  Fubp3  Zfp946  2610044O15Rik8 | web link |
| response to organic cyclic compound | Gene Ontology | GO:0014070 | 5 (1.00%) | 151 (0.72%) | 0.294096364408 | 0.368218212349 | Hsf1  Hnf1b  Idh1  Egfr  Comt | web link |
| protein localization to plasma membrane | Gene Ontology | GO:0072659 | 5 (1.00%) | 151 (0.72%) | 0.294096364408 | 0.368218212349 | Slmap  Pgap2  Gak  Cask  Mrap | web link |
| response to lipopolysaccharide | Gene Ontology | GO:0032496 | 6 (1.20%) | 191 (0.91%) | 0.306958613619 | 0.381222794333 | Hsf1  Foxp1  Adipor2  Comt  Mgst1  Mapkapk2 | web link |
| cell population proliferation | Gene Ontology | GO:0008283 | 5 (1.00%) | 159 (0.76%) | 0.33157233874 | 0.405241483604 | Cops2  Bcl2l1  Vegfa  Prkar1a  Naa60 | web link |
| response to hypoxia | Gene Ontology | GO:0001666 | 6 (1.20%) | 197 (0.94%) | 0.332167630414 | 0.405241483604 | Slc11a2  Cflar  Alas2  Angptl4  Vegfa  Nf1 | web link |
| DNA repair | Gene Ontology | GO:0006281 | 10 (2.00%) | 352 (1.68%) | 0.334192652063 | 0.405241483604 | Hsf1  Emsy  Timeless  Cyren  Ube2d3  Apex1  Fbxo6  Swi5  Nabp2  Mms19 | web link |
| peptidyl-serine phosphorylation | Gene Ontology | GO:0018105 | 5 (1.00%) | 163 (0.78%) | 0.350487780056 | 0.418498745964 | Uhmk1  Mast3  Mapkapk2  Csnk1a1  Rps6kb2 | web link |
| cell cycle | Gene Ontology | GO:0007049 | 16 (3.19%) | 595 (2.84%) | 0.350560637853 | 0.418498745964 | Ube2i  Tsg101  Klhl13  Csnk1a1  Pmf1  Ppp1ca  Birc6  Stk11  Timeless  Hnrnpu  Pard6a  Septin4  Nup37  Nde1  Ing1  Tfdp1 | web link |
| positive regulation of neuron projection development | Gene Ontology | GO:0010976 | 5 (1.00%) | 164 (0.78%) | 0.355226886722 | 0.42080723504 | Cflar  Ddx56  Sf3a2  Nf1  Baiap2 | web link |
| mRNA splicing, via spliceosome | Gene Ontology | GO:0000398 | 5 (1.00%) | 167 (0.80%) | 0.369457080736 | 0.431794023772 | Srsf5  Cwf19l1  Snrpa1  Sf3a2  Srsf2 | web link |
| positive regulation of cell migration | Gene Ontology | GO:0030335 | 7 (1.40%) | 245 (1.17%) | 0.370109163233 | 0.431794023772 | Carmil1  Washc1  Egfr  Cep43  Vegfa  Sema4a  Sema4g | web link |
| negative regulation of neuron apoptotic process | Gene Ontology | GO:0043524 | 5 (1.00%) | 169 (0.81%) | 0.378947476318 | 0.438781288369 | Bcl2l1  Xiap  Set  Retreg1  Kras | web link |
| response to drug | Gene Ontology | GO:0042493 | 9 (1.80%) | 329 (1.57%) | 0.388000235722 | 0.445910718665 | Adra1a  Hnf1b  Adipor2  Abcg8  Comt  Apex1  Mgst1  Gnpat  Akr1c6 | web link |
| carbohydrate metabolic process | Gene Ontology | GO:0005975 | 6 (1.20%) | 215 (1.03%) | 0.408775339664 | 0.463120388693 | Ppp1ca  Mgam  Phkb  Fbp1  Gba2  Xylb | web link |
| negative regulation of gene expression | Gene Ontology | GO:0010629 | 8 (1.60%) | 295 (1.41%) | 0.408989434171 | 0.463120388693 | Hsf1  Adipor2  Esr1  Ezh2  Vegfa  Cnpy2  Gas2l1  Tardbp | web link |
| negative regulation of endopeptidase activity | Gene Ontology | GO:0010951 | 5 (1.00%) | 176 (0.84%) | 0.412100135178 | 0.463236648302 | AI182371  Birc6  Serpina1a  Fetub  Itih4 | web link |
| positive regulation of ERK1 and ERK2 cascade | Gene Ontology | GO:0070374 | 6 (1.20%) | 223 (1.06%) | 0.442724357644 | 0.494054717951 | Adra1a  Esr1  Egfr  Cflar  Vegfa  Fgfr3 | web link |
| positive regulation of apoptotic process | Gene Ontology | GO:0043065 | 9 (1.80%) | 360 (1.72%) | 0.492918158157 | 0.546110765152 | Bcl2l1  Dnaja3  Fas  Dcun1d3  Septin4  Nf1  Clu  Bin1  Eif5a | web link |
| protein phosphopantetheinylation | Gene Ontology | GO:0018215 | 5 (1.00%) | 201 (0.96%) | 0.52697033732 | 0.579667371052 | Zdhhc20  Ero1b  Asph  Hccs  Zdhhc6 | web link |
| regulation of gene expression | Gene Ontology | GO:0010468 | 9 (1.80%) | 376 (1.79%) | 0.545373312492 | 0.595655958325 | Tsku  Fas  Cggbp1  Ezh2  Pcbp2  Nf1  Fubp3  Trim26  Tardbp | web link |
| innate immune response | Gene Ontology | GO:0045087 | 13 (2.59%) | 560 (2.67%) | 0.582282761859 | 0.631489755819 | Cfh  Cr1l  Trim26  C4bp  Ankhd1  Masp1  1810058I24Rik  Pcbp2  G3bp2  AI182371  Nlrc5  Pja2  Irgm2 | web link |
| ion transport | Gene Ontology | GO:0006811 | 14 (2.79%) | 622 (2.97%) | 0.62887080588 | 0.677245483255 | Slc38a7  Tmem38b  Slc11a2  Slc38a3  Slc39a14  Fxyd1  Slc40a1  Slc17a2  Slc38a10  Slc12a7  Slc39a11  Atp5j  Tmem175  Slc39a7 | web link |
| multicellular organism development | Gene Ontology | GO:0007275 | 23 (4.59%) | 1040 (4.96%) | 0.680081921191 | 0.727309832385 | Ube2i  Deaf1  Hnrnpu  Pkdcc  Vegfa  Nde1  Bin1  Kdm5a  Cadm1  Strbp  Poglut1  Zc3h13  Ndrg2  Mink1  Timeless  Plekhb1  Sema4a  Sema4g  Tsku  Ngef  Cr1l  Egfr  Phc2 | web link |
| transmembrane transport | Gene Ontology | GO:0055085 | 12 (2.40%) | 568 (2.71%) | 0.708710931572 | 0.752266166051 | Slc39a11  Slc25a44  Slc39a14  Slc22a18  Slc22a30  Mpv17  Slc17a2  Abcg8  Slc12a7  Slc25a19  Abcc6  Slc39a7 | web link |
| vesicle-mediated transport | Gene Ontology | GO:0016192 | 5 (1.00%) | 249 (1.19%) | 0.713187404178 | 0.752266166051 | Sec22c  Sec16b  Snap23  Sec31a  Cope | web link |
| angiogenesis | Gene Ontology | GO:0001525 | 5 (1.00%) | 254 (1.21%) | 0.729249345514 | 0.763975504824 | Sema4a  Angptl4  Vegfa  Pnpla6  Cfh | web link |
| positive regulation of gene expression | Gene Ontology | GO:0010628 | 10 (2.00%) | 525 (2.50%) | 0.808854775117 | 0.841646184919 | Hsf1  Fubp3  Afdn  Hnrnpu  Hnf1b  Vegfa  Dtnbp1  Clu  Sec16b  Kras | web link |
| inflammatory response | Gene Ontology | GO:0006954 | 6 (1.20%) | 344 (1.64%) | 0.833767672004 | 0.861746452944 | Cfh  Sdc1  Igfbp4  AI182371  Pja2  Mapkapk2 | web link |
| intracellular signal transduction | Gene Ontology | GO:0035556 | 7 (1.40%) | 421 (2.01%) | 0.879259228259 | 0.902706141012 | Arhgef12  Egfr  Mapk15  Mast3  Sh2b1  Nlrc5  Mapkapk2 | web link |
| biological\_process | Gene Ontology | GO:0008150 | 57 (11.38%) | 2834 (13.52%) | 0.934044660865 | 0.952601839558 | Gpank1  9430038I01Rik  Ccdc97  Cmbl  Fbrsl1  Atp5mpl  Plekha6  Zfand4  1700066M21Rik  Mmaa  Tmem219  Fam169b  Apol9b  Snx29  Card19  Ccdc58  Thap2  0610010K14Rik  Golga1  Gucd1  2700062C07Rik  Phf7  Proser2  Ccdc159  Miip  Mbd6  Fpgt  Szrd1  Bri3bp  Wdr20  Arl14ep  Vwa5a  Kctd18  Fam47e  Haghl  Wbp1l  Septin4  Rcbtb2  Spata2l  Susd1  Tmem222  D11Wsu47e  Ntpcr  Mettl9  Qser1  Smco4  Fam214a  Fam214b  Commd4  Smim10l1  Wdr27  Fance  Anks3  Srsf11  Sap18  Vwa3b  Btf3l4 | web link |
| immune response | Gene Ontology | GO:0006955 | 5 (1.00%) | 376 (1.79%) | 0.9481102305 | 0.960585365112 | Marchf8  Fas  Enpp2  Cfh  Ccl27a | web link |
| signal transduction | Gene Ontology | GO:0007165 | 25 (4.99%) | 2811 (13.41%) | 0.999999999903 | 1.0 | Magi1  Adgre5  Afdn  Raph1  Vegfa  Nf1  Sh2b1  Gm45844  Adra1a  Fas  Ppp4r1  Rin2  Rgs3  Arhgap5  Ndrg2  Arap2  Nras  Gm45713  Ccl27a  Zyx  Erap1  Egfr  Rps6kb2  Bcar3  Kras | web link |
| G protein-coupled receptor signaling pathway | Gene Ontology | GO:0007186 | 5 (1.00%) | 2101 (10.02%) | 1.0 | 1.0 | Adgre5  Adra1a  Arhgef12  Gm45844  Rgs3 | web link |
