# Supplementary material for: Regulation of Alternative Splicing of Lipid Metabolism Genes in Sepsis-Induced Liver Damage by RNA-Binding Proteins
Source: Inflammation. 2024 May 9;47(6):1952–68. doi: 10.1007/s10753-024-02017-2 (PMC11607010; doi:10.1007/s10753-024-02017-2)
Supplement: Supplementary file 1 — Supplementary file1 (ZIP 38655 KB) [file 10753_2024_2017_MOESM1_ESM.zip › Sup5_AS_GO.zip!/Sup5_AS_GO/ras/CLPDCA_vs_Sham/Ras_GO_analysis/Ras_GO_enrichment_web_C.html]

GO enrichment analyze


# GO enrichment analyze result


| Term | Database | ID | Input number (574) | Background number (20939) | P-Value | Corrected P-Value | Input | Hyperlink |
| --- | --- | --- | --- | --- | --- | --- | --- | --- |
| mitochondrion | Gene Ontology | GO:0005739 | 91 (15.85%) | 1745 (8.33%) | 1.63708180345e-09 | 1.68619425756e-07 | Coa8  Apoo  Mpv17  Hsd3b2  Nme4  Card19  Esr1  1810058I24Rik  Mtx3  Hccs  Slc11a2  Aldh3a2  Mthfs  Osgepl1  Mars1  Septin4  Mterf3  Crls1  Nipsnap1  Cfh  Rpl35a  Taz  Atp5j  Mtif2  Ssbp1  Abhd5  Idh1  Sugct  Immt  Bcl2l1  Idh3g  Slc25a19  Echdc2  Ghr  Ifi27  Gnpat  Mff  Mgst1  Mpv17l2  Fdxr  Kras  Slc25a44  Snap23  Atp5mpl  Clu  D2hgdh  Comt  Agxt2  Bphl  Mettl17  Ociad2  Metap1d  P4ha1  Thra  Irgm2  Smurf1  Apex1  Cox7a2  Cln8  Fastkd3  Stk11  Dhtkd1  Mmaa  Atg13  Dmac2  Bri3bp  Mtfr1l  Ccdc58  Prodh2  Higd1a  Mrps2  Ndufaf4  Ndufs2  Ndufaf1  Ndufaf2  Hoga1  Etfdh  Alas2  Mtarc2  Adhfe1  Etfbkmt  Mcee  Foxk2  Dglucy  Dnaja3  Trak1  Mrps17  Tmem143  Bckdhb  Mrpl36  Etfrf1 | web link |
| cytosol | Gene Ontology | GO:0005829 | 147 (25.61%) | 3762 (17.97%) | 2.34580286218e-06 | 0.000120808847402 | Pdlim5  Apoo  Afdn  Rubcn  Nf2  Tfdp1  Asap3  Rpl38  Fas  Irf9  Cdk16  Tcp1  Gak  Rnf216  Tut4  Midn  Hccs  Zbtb14  Aldh3a2  Mterf3  Ubc  Mars1  Pcbp2  Phf7  Ube2i  Upp2  Rnf123  Desi1  Rpl35a  Cdc37l1  Sap18  G3bp2  Ppp1r13l  Mid1ip1  Abhd5  Fnip1  Eef1d  Tbc1d5  Thra  Txndc9  Raph1  Plekha1  Sh2b1  Flnb  Tmem87a  Adra1a  Gemin6  Cpeb3  Baiap2  Zswim8  Madd  Washc1  Slc40a1  Ghr  Gstm4  Mvk  Zyx  Wdyhv1  Ngef  Ppp1ca  Tdrd3  Zc3h4  Commd4  Xiap  Ncor1  Gss  Gba2  Sec31a  Tsg101  Hsf1  Cmbl  Gigyf2  Ifi30  Coro1b  Clu  Bin1  Mphosph8  Xylb  Comt  Bnip2  Nlrc5  Idh1  Nrf1  Exoc7  Slc66a2  Mtr  Fndc3a  Btrc  Sh3glb2  Masp1  Gstp3  Anks1  Ahsa2  Eed  Phldb2  Irgm2  Smurf1  Fam160a2  Set  Ahcyl1  Rpl15  Ampd2  Pgpep1l  Fbxo3  Akr1c6  Rnf146  Srsf5  Srsf2  Stk11  Stk16  Akr1e1  Atg13  Fbp1  Ppig  Lcp1  Tsc1  Tcof1  Csnk1a1  Tpm1  0610010K14Rik  Ppip5k2  Arl14ep  Uba52  Ndufaf1  Xrn1  Map2k5  Mthfs  Arhgap5  Josd2  Thoc7  Bcl2l1  Carmil1  Cask  Nabp2  Eef1akmt2  Mms19  Repin1  Dnaja3  Sys1  Mat1a  Gsta3  Pnpla6  Gprasp1  Vwa3b  Setd4  Sharpin  Cdc42 | web link |
| nuclear speck | Gene Ontology | GO:0016607 | 27 (4.70%) | 403 (1.92%) | 2.03011262735e-05 | 0.000509613832418 | Srsf5  Srsf2  Poldip3  Afdn  Wac  Hnrnpu  Apex1  Zfp638  Ppig  Csnk1a1  Cdk12  Zc3h13  Zc3h14  Phf7  Thoc7  Rexo4  Carmil1  Sap18  Srrm1  Chtop  Luc7l2  Hp1bp3  Snrpa1  Spop  Rbm25  Srsf11  Sf3a2 | web link |
| endoplasmic reticulum membrane | Gene Ontology | GO:0005789 | 47 (8.19%) | 903 (4.31%) | 2.08112673663e-05 | 0.000509613832418 | Sec22c  Apoo  Cept1  Calu  Ncln  Sc5d  Derl3  Hsd3b2  Dtnbp1  Pex16  Porcn  Card19  Tmem38b  Ube2j2  Ero1b  Dnajb12  Elovl5  Yif1b  Cyp3a59  Pigx  Agpat3  Cyp2r1  Sptssa  Pnpla6  Cln8  Aldh3a2  Asph  Retreg1  Ggcx  Tmem208  Arv1  Irgm2  Gba2  Cdipt  Erap1  Pja2  Zdhhc6  Egfr  Cyp4b1  Mrap  Mgst1  Rdh11  Sec16b  Slc39a7  Pgap2  Sec31a  Eif5a | web link |
| cytoplasm | Gene Ontology | GO:0005737 | 229 (39.90%) | 6677 (31.89%) | 2.47385355543e-05 | 0.000509613832418 | Sec22c  Pdlim5  Slc39a11  Gas2l1  Slc22a18  Mpv17  Afdn  Hnrnpu  Pcmtd1  Nf2  Hsd3b2  Nde1  Ctbp1  Eapp  Tfdp1  Asap3  Ppip5k2  Cyren  Mtmr14  Esr1  Spop  Fas  Hnrnpa1  Rnf181  Rin2  Atg4c  Tcp1  Mtx3  Zc3h14  Rgs3  Steap3  Cyp2r1  Tardbp  Midn  Pja2  Slc11a2  Arap2  Arl14ep  Mthfs  Ubc  Uxt  Fam47e  Pcbp2  Spata2l  Cdc42  Zdhhc20  Ube2i  Upp2  Arhgef12  Rnf123  Cfh  Snx10  Cops2  Fubp3  Desi1  Enpp2  Nudt6  Fgfr3  Slmap  Cdc37l1  Rbm25  G3bp2  Cpsf7  Ppp1r13l  Pgpep1l  Mid1ip1  Abhd5  Zyx  Fnip1  Tfr2  Dram2  Nf1  Poldip3  Egfr  Idh1  Asph  Thra  Txndc9  Ubr3  Pxmp2  Plekha1  Ablim2  Sh2b1  Flnb  Adra1a  Foxp1  Ssb  Foxk2  Setd4  Gemin6  Cpeb3  Mapkapk2  Mapk15  Fpgt  Mycbp2  Baiap2  Ndrg2  Zswim8  Setdb1  Madd  Washc1  Ghr  Krt10  Gstm4  Uhmk1  Plekhb1  Pja1  Cep43  Mvk  Psmc6  Tagln  Wdyhv1  Ngef  Ppp1ca  Tdrd3  Commd4  Rps6kb2  Xiap  Sult2a8  Bnip2  Kras  Tsg101  Meiob  Cmbl  Sun1  Gigyf2  Snap23  Cdk16  Csad  Comt  Coro1b  Ezh2  Vegfa  Ankib1  Clu  Mtmr4  Bin1  Mphosph8  Strbp  Gtf2i  Ubap2l  Nadsyn1  Pard6a  Rnf149  Bbip1  Dtnb  Dido1  Bphl  Nlrc5  Magi1  Rnf146  Mink1  Stk11  Exoc7  Mtr  Deaf1  Btrc  Sh3glb2  Nsmf  Anks1  Anks3  Ints7  Cyp4a31  Phldb2  Nudt21  Smurf1  Erap1  Set  Ahcyl1  Ankrd13a  Trim26  Prkar1a  Fbxo6  Akr1c6  Nrf1  Itih4  Dctn4  Hsf1  Rnf216  Srsf2  Ankhd1  Stk16  Akr1e1  Apex1  Ppig  Atg13  Fbp1  Dtnbp1  Mmaa  Lcp1  Tsc1  Csnk1a1  Cope  Paox  Tpm1  Marchf8  Uba52  Xrn1  Map2k5  Anp32a  Josd2  Thoc7  Ido2  Bcl2l1  Carmil1  Mpv17l2  Jmjd4  Bcar3  Gmppa  Cask  Mars1  Cnot10  Dcun1d3  Etfbkmt  Eef1akmt2  Mms19  Dnaja3  Trio  Lims2  Birc6  Trak1  Gsta3  Cflar  Sec31a  Septin4  Eif5a  Tut4  Sharpin  Gprasp1 | web link |
| mitochondrial inner membrane | Gene Ontology | GO:0005743 | 27 (4.70%) | 421 (2.01%) | 4.33893511061e-05 | 0.000744850527322 | Slc25a44  Coa8  Apoo  Mpv17  Hsd3b2  Clu  Immt  Bcl2l1  Prodh2  Higd1a  Ndufs2  Slc25a19  Hccs  Aldh3a2  Etfdh  Alas2  Mtarc2  Bckdhb  Crls1  Ociad2  Nipsnap1  Taz  Atp5j  Mgst1  Mpv17l2  Fdxr  Cox7a2 | web link |
| nucleus | Gene Ontology | GO:0005634 | 208 (36.24%) | 6031 (28.80%) | 5.59748216122e-05 | 0.000823629518009 | Slc39a11  Meiob  Safb  Hnrnpu  Nf2  Nf1  Eed  Ctbp1  Hmga1  Rsl1  Mapkapk2  Tfdp1  Clk4  Gm28557  Cyren  Cggbp1  Ube2j2  Esr1  Cdk12  Irf9  Cnot10  Cdk16  Nup37  Ing1  Zc3h13  Zc3h14  Gtf2a2  Rgs3  Rnf216  Smarca4  Tardbp  Midn  Nfib  Nfia  Slc11a2  Zbtb14  Aldh3a2  Anp32a  Mars1  Sfmbt1  Pcbp2  Ube2i  Polr2h  Thoc2l  Uxt  Cfh  Snx10  Cops2  Fubp3  Ddx56  Nudt5  Zfp995  Rbm25  Sf3a2  Cpsf7  Ppp1r13l  Ssbp4  Ncor1  Tsen34  Fgfr3  Gabpb1  Magi1  Zbtb7c  Poldip3  Egfr  Deaf1  Thra  Txndc9  Rpl15  Plekha1  Knop1  Sh2b1  Flnb  Adra1a  Foxp1  Ssb  Foxk2  Pmf1  Setd4  Gemin6  Ube2d3  Cpeb3  Kdm5a  Mapk15  Mbd6  Mycbp2  Pcif1  Phf7  Ndrg2  Setdb1  Ghr  Timeless  Uhmk1  Hnf1b  Cep43  Gtf2i  Hnrnpll  Psmc6  Luc7l2  Wdyhv1  Ints7  Ppp1ca  Tdrd3  Rexo4  Commd4  Ncor2  Spop  Xiap  Mgst1  Dtnbp1  Eloc  Zfp62  Tsg101  Sbno2  Sun1  Wac  Zkscan1  Ezh2  Vegfa  Zfp638  Ptbp3  Clu  Ptbp1  Bin1  Mphosph8  Strbp  Ubap2l  Dtnb  Pard6a  Dnajb12  Agpat3  Zyx  Nlrc5  Nudt21  Ctdsp1  Nrf1  Emsy  Dnaja3  Cept1  Btrc  Nsmf  Hsf1  Smarcc2  Zfp874a  Eapp  Kmt2c  Set  Desi1  Xrn1  Trim26  Snrpa1  Fbxo3  Akr1c6  Rnf146  Trak1  Srsf5  Thap2  Srsf2  Tle2  Rtca  Stk11  Apex1  Hnrnpa1  Fbp1  Tmem176b  Ppig  Zfp141  Rps6kb2  Phf20l1  Tsc1  Tcof1  Csnk1a1  Higd1a  Mid1ip1  0610010K14Rik  Mtus1  Uba52  Zfp950  Scaf1  Map2k5  Ubc  Nsd1  Thoc7  Bcl2l1  Carmil1  Zfp932  Chtop  Cask  Dcun1d3  Zfp235  Nabp2  Eef1akmt2  Mms19  Repin1  Taf13  Dido1  Hp1bp3  Zfp467  Birc6  Fam214b  Zfpm1  Zfp131  Zfp946  Phc2  Swi5  Gprasp1  Tut4  Eif5a | web link |
| endoplasmic reticulum | Gene Ontology | GO:0005783 | 66 (11.50%) | 1518 (7.25%) | 0.000132389654928 | 0.0017045168072 | Sec22c  Ggcx  Asph  Gigyf2  Sc5d  Apoo  Cept1  Calu  Ncln  Apex1  Zdhhc20  Serpina1a  Cnpy2  Hsd3b2  Dtnbp1  Clu  Yif1b  Proc  F10  Pex16  P4ha1  Porcn  Card19  Tmem38b  Ube2j2  Tmem258  Slc39a7  Poglut1  Dnajb12  Elovl5  Agpat3  Anp32a  Pigx  Arhgap5  Cyp2r1  Sptssa  Fgfr3  Rdh11  Bcl2l1  Cln8  Mgst1  Aldh3a2  Ero1b  Retreg1  Pgap2  Tmem208  Arv1  Sec16b  Hsd17b6  Cdipt  Sil1  Erap1  Set  Ahcyl1  Ces3a  Snx10  Zdhhc6  Egfr  Cyp4b1  Mrap  Derl3  Pnpla6  Gba2  Pja2  Sec31a  Eif5a | web link |
| mitochondrial membrane | Gene Ontology | GO:0031966 | 14 (2.44%) | 174 (0.83%) | 0.000325891038 | 0.00372964187933 | Bcl2l1  Card19  Slc25a44  Mff  Trak1  Higd1a  Taz  Atp5mpl  Ndufaf4  Etfdh  Hsd3b2  Gnpat  Crls1  Clu | web link |
| Golgi apparatus | Gene Ontology | GO:0005794 | 57 (9.93%) | 1309 (6.25%) | 0.000373217953443 | 0.00384414492046 | Acer2  Enpp2  Slc39a11  Gigyf2  Sys1  Stk16  Tbc1d5  Slc38a10  Pkdcc  Serpina1a  Gfra1  Naa60  Yif1b  Proc  Phf7  Pgap2  Pmf1  Esr1  Neo1  Golga1  Tcp1  Calu  Gak  Sgms2  Apoo  Fndc3a  Ndrg2  Sppl2b  Fgfr3  Slc39a7  Lysmd3  Mink1  Scamp5  St3gal5  St3gal4  Cept1  Nras  Uhmk1  Retreg1  Baiap2  Zdhhc20  Arv1  Irgm2  Sec16b  Mapk15  Cdipt  Tmbim4  Zdhhc6  Tdrd3  Birc6  Paqr3  Cope  Egfr  Golgb1  Dram2  Gba2  Pja2 | web link |
| apical dendrite | Gene Ontology | GO:0097440 | 5 (0.87%) | 27 (0.13%) | 0.000744780612973 | 0.00697385483056 | Nsmf  Clu  Cask  Cpeb3  Fas | web link |
| intracellular membrane-bounded organelle | Gene Ontology | GO:0043231 | 47 (8.19%) | 1061 (5.07%) | 0.000855198339679 | 0.00734045241558 | Dram2  Rubcn  Ifi30  Tmem208  Serpina1a  Zfp638  Mrap  Hsd3b2  Ppig  Clu  F10  Tsc1  Adra1a  Ggcx  Foxk2  Pmf1  Sun1  Comt  Tbc1d5  Aldh3a2  Gak  Mycbp2  Asap3  Setdb1  Washc1  Arl14ep  Cyp3a59  Calu  Hnf1b  P4ha1  Zdhhc20  Cyp4a31  Mvk  Eef1akmt2  Hsd17b6  Dnaja3  Erap1  Cyp2r1  Ahcyl1  Sc5d  Cyp4b1  Commd4  Mgst1  Bnip2  Sec16b  Sec31a  Abhd5 | web link |
| nucleoplasm | Gene Ontology | GO:0005654 | 114 (19.86%) | 3156 (15.07%) | 0.0010086894863 | 0.00799192439142 | Gemin6  Afdn  Hnrnpu  Magi1  Ctbp1  Mapkapk2  Tfdp1  Asap3  Cdk12  Hnrnpa1  Ndufs2  Nup37  Zc3h13  Neo1  Rexo4  Gtf2a2  Rnf216  Slc39a7  Nfib  Nfia  Zbtb14  Arl14ep  Rubcn  Sfmbt1  Pcbp2  Phf7  Polr2h  Mtif2  Sap18  Nrf1  Ncor2  Ncor1  Abhd5  Tsen34  Gabpb1  Eef1d  Deaf1  Masp1  Snrpa1  Plekha1  Smarcc2  Foxk2  Pmf1  Safb  Cpeb3  Mbd6  Mycbp2  Pcif1  Baiap2  Setdb1  Slc40a1  Zfp973  Timeless  Uhmk1  Dcaf11  Hnf1b  Gtf2i  Dido1  Luc7l2  Ppp1ca  Tdrd3  Zc3h4  Ube2i  Srsf11  Srsf5  Snap23  Wac  Ezh2  Zfp638  Mphosph8  Sppl2b  Mettl17  Rnf146  Emsy  Vwa5a  Sh3glb2  Nsmf  Anks1  Eed  Abcc6  Set  Cggbp1  Bahd1  Ctdsp1  Fbxo3  Tardbp  Hsf1  Fastkd3  Srsf2  Tle2  Rtca  Stk11  Stk16  Apex1  Tcof1  Cope  Higd1a  0610010K14Rik  Uba52  Ubc  Adra1a  Carmil1  Chtop  Nabp2  Eef1akmt2  Mms19  Repin1  Taf13  Zfp131  Wdr27  Phc2  Septin4  2610044O15Rik8  Vwa3b | web link |
| stress fiber | Gene Ontology | GO:0001725 | 8 (1.39%) | 82 (0.39%) | 0.00182639937282 | 0.0134370811001 | Pdlim5  Gas2l1  Tpm1  Coro1b  Zyx  Lcp1  Flnb  Dctn4 | web link |
| peroxisome | Gene Ontology | GO:0005777 | 10 (1.74%) | 136 (0.65%) | 0.0043132897874 | 0.0296179232068 | Mff  Aldh3a2  Tysnd1  Idh1  Paox  Mtarc2  Gnpat  Mvk  Mpv17  Pex16 | web link |
| cytoplasmic ribonucleoprotein granule | Gene Ontology | GO:0036464 | 7 (1.22%) | 77 (0.37%) | 0.00515008562437 | 0.0331536762069 | Gabpb1  Ghr  Poldip3  Chtop  Hnrnpu  Tut4  Cdc42 | web link |
| endosome membrane | Gene Ontology | GO:0010008 | 12 (2.09%) | 206 (0.98%) | 0.0116468040087 | 0.0705659301702 | Tsg101  Washc1  Slc11a2  Snx10  Egfr  Ube2d3  Tbc1d5  Kif13a  Dtnbp1  Sppl2b  Steap3  Tmem175 | web link |
| endosome | Gene Ontology | GO:0005768 | 28 (4.88%) | 662 (3.16%) | 0.0160274479345 | 0.0863125456529 | Tsg101  Gigyf2  Tbc1d5  Washc1  Gfra1  Dtnbp1  Mtmr4  Bin1  Slc39a14  Ube2d3  Marchf8  Rnf215  Steap3  Tmem175  Scamp5  Slc11a2  Slc66a2  Rubcn  Hsd17b6  Ociad2  Trio  Sppl2b  Snx10  Birc6  Trak1  Ankrd13a  Egfr  Sec31a | web link |
| midbody | Gene Ontology | GO:0030496 | 10 (1.74%) | 166 (0.79%) | 0.0164111842404 | 0.0863125456529 | Klhl13  Birc6  Safb  Hnrnpu  Cpeb3  Uhmk1  Txndc9  Kif13a  Dtnbp1  Cdc42 | web link |
| heterochromatin | Gene Ontology | GO:0000792 | 5 (0.87%) | 55 (0.26%) | 0.0171647426544 | 0.0863125456529 | Hsf1  Mphosph8  Tcp1  Smarca4  Phc2 | web link |
| microtubule organizing center | Gene Ontology | GO:0005815 | 17 (2.96%) | 353 (1.69%) | 0.0182606413871 | 0.0863125456529 | Hsf1  Tsg101  Uxt  Csnk1a1  Snx10  Birc6  Bcl2l1  Hnrnpu  Cep43  Txndc9  Tcp1  Pard6a  Exoc7  Nde1  Slmap  Dctn4  Cdc42 | web link |
| integral component of Golgi membrane | Gene Ontology | GO:0030173 | 5 (0.87%) | 56 (0.27%) | 0.0184356893628 | 0.0863125456529 | Zdhhc20  Acer2  Yif1b  Sys1  Sgms2 | web link |
| nuclear matrix | Gene Ontology | GO:0016363 | 7 (1.22%) | 105 (0.50%) | 0.0255056999685 | 0.11422117812 | Mat1a  Cask  Hnrnpu  Nsmf  Anp32a  Ppig  Ncor2 | web link |
| Golgi membrane | Gene Ontology | GO:0000139 | 21 (3.66%) | 485 (2.32%) | 0.0272229386533 | 0.116831778387 | Acer2  Pgap2  St3gal5  Cope  Apoo  Paqr3  Sppl2b  Sys1  Egfr  Scamp5  Yif1b  Sgms2  Naa60  Zdhhc20  Fndc3a  Gba2  Tmbim4  Irgm2  Sec16b  Pja2  Cdc42 | web link |
| focal adhesion | Gene Ontology | GO:0005925 | 10 (1.74%) | 184 (0.88%) | 0.0307783674745 | 0.11771387256 | Zyx  Flnb  Arl14ep  Tle2  Lims2  Dctn4  Phldb2  Lcp1  Bcar3  Asap3 | web link |
| actin filament | Gene Ontology | GO:0005884 | 6 (1.05%) | 86 (0.41%) | 0.0307785941459 | 0.11771387256 | Dnaja3  Gas2l1  Tpm1  Coro1b  Lcp1  Tsc1 | web link |
| cytosolic large ribosomal subunit | Gene Ontology | GO:0022625 | 5 (0.87%) | 64 (0.31%) | 0.0308570345546 | 0.11771387256 | Rpl15  Uba52  Gm45713  Rpl35a  Rpl38 | web link |
| integral component of endoplasmic reticulum membrane | Gene Ontology | GO:0030176 | 7 (1.22%) | 113 (0.54%) | 0.0360330668978 | 0.131057526174 | Porcn  Elovl5  Asph  Retreg1  Dnajb12  Derl3  Sgms2 | web link |
| postsynaptic density | Gene Ontology | GO:0014069 | 15 (2.61%) | 327 (1.56%) | 0.0368996918355 | 0.131057526174 | Pdlim5  Tsc1  Rpl38  Mink1  Cask  Nsmf  Dtnb  Cpeb3  Nf1  Dtnbp1  Baiap2  Pja2  Pcbp2  Sharpin  Cadm1 | web link |
| nucleolus | Gene Ontology | GO:0005730 | 31 (5.40%) | 813 (3.88%) | 0.040950858309 | 0.136299171933 | Tsg101  Srsf5  Thap2  Knop1  Tsen34  Retreg1  Apex1  Nf2  Magi1  Hsd3b2  Kdm5a  Mphosph8  Tcof1  Mtus1  Rexo4  Smarca4  Zc3h14  Midn  Idh3g  Zbtb14  Arl14ep  Chtop  Mars1  Cask  Taf13  Bckdhb  Nf1  Polr2h  Ppp1ca  Ddx56  Tut4 | web link |
| condensed chromosome kinetochore | Gene Ontology | GO:0000777 | 6 (1.05%) | 93 (0.44%) | 0.0426502426039 | 0.136299171933 | Hsf1  Csnk1a1  Pmf1  Hnrnpu  Nup37  Nde1 | web link |
| perinuclear region of cytoplasm | Gene Ontology | GO:0048471 | 29 (5.05%) | 755 (3.61%) | 0.0434116225416 | 0.136299171933 | Hsf1  Stk16  Apex1  Nf2  Clu  Lcp1  Inf2  Tsc1  Mtmr14  Esr1  Fas  Golga1  Asgr2  Anp32a  Ndrg2  Atp9b  Slc11a2  Cep43  Dcun1d3  Cask  Zdhhc20  Coro1b  Set  Ankrd13a  Trak1  Egfr  Xiap  Bnip2  Sec31a | web link |
| presynapse | Gene Ontology | GO:0098793 | 9 (1.57%) | 169 (0.81%) | 0.0436686667359 | 0.136299171933 | Bcl2l1  Pdlim5  Nf1  Snap23  Ppp1ca  Slc29a1  Baiap2  Ctbp1  Cln8 | web link |
| centrosome | Gene Ontology | GO:0005813 | 21 (3.66%) | 522 (2.49%) | 0.0525322619955 | 0.157524345335 | Bcl2l1  Hsf1  Uxt  Washc1  Csnk1a1  Prkar1a  Snx10  Hnrnpu  Cep43  Txndc9  Apex1  Kif13a  Tcp1  Pard6a  Nde1  Nlrc5  Nudt21  Sppl2b  Mapkapk2  Dctn4  Cdc42 | web link |
| kinetochore | Gene Ontology | GO:0000776 | 7 (1.22%) | 124 (0.59%) | 0.0546711618258 | 0.157524345335 | Hsf1  Csnk1a1  Pmf1  Hnrnpu  Nup37  Nde1  Dctn4 | web link |
| mitochondrial outer membrane | Gene Ontology | GO:0005741 | 9 (1.57%) | 177 (0.85%) | 0.0554884622271 | 0.157524345335 | Bcl2l1  Slc11a2  Mff  Mgst1  1810058I24Rik  Taz  Mtarc2  Mtx3  Bri3bp | web link |
| adherens junction | Gene Ontology | GO:0005912 | 8 (1.39%) | 151 (0.72%) | 0.0565864153146 | 0.157524345335 | Pdlim5  Snap23  Afdn  Ppp1ca  Vegfa  Nf2  Magi1  Zyx | web link |
| Schaffer collateral - CA1 synapse | Gene Ontology | GO:0098685 | 6 (1.05%) | 104 (0.50%) | 0.0664488507657 | 0.180111358654 | Cadm1  Cask  Dtnbp1  Baiap2  Rnf216  Cdc42 | web link |
| ruffle | Gene Ontology | GO:0001726 | 6 (1.05%) | 109 (0.52%) | 0.0793851878152 | 0.209658316538 | Mtmr14  Nf2  Sh2b1  Baiap2  Lcp1  Asap3 | web link |
| Z disc | Gene Ontology | GO:0030018 | 7 (1.22%) | 138 (0.66%) | 0.0857688359241 | 0.220854752504 | Adra1a  Pdlim5  Stk11  Neb  Slmap  Flnb  Bin1 | web link |
| synapse | Gene Ontology | GO:0045202 | 29 (5.05%) | 814 (3.89%) | 0.0913550061231 | 0.229501600748 | Ube2i  Pdlim5  Snap23  Nf2  Dtnbp1  Nde1  Clu  Bin1  Cadm1  Bcl2l1  Rpl38  Cdk16  Cpeb3  Uba52  Dtnb  Xrn1  Mink1  Ly6e  Scamp5  Cask  Nsmf  Slc12a7  Dnaja3  Mff  Egfr  Prkar1a  Pja2  Sharpin  Eif5a | web link |
| endoplasmic reticulum lumen | Gene Ontology | GO:0005788 | 6 (1.05%) | 115 (0.55%) | 0.096637114041 | 0.236991017767 | Sil1  Erap1  Ces3a  Calu  Poglut1  P4ha1 | web link |
| nuclear body | Gene Ontology | GO:0016604 | 13 (2.26%) | 318 (1.52%) | 0.0999761499282 | 0.239477754479 | Ube2i  Mrpl36  Ints7  Tle2  Retreg1  Gemin6  Snrpa1  Ncor2  Raph1  Sap18  Fas  Nudt21  Eef1akmt2 | web link |
| lipid droplet | Gene Ontology | GO:0005811 | 5 (0.87%) | 93 (0.44%) | 0.112276435603 | 0.262828928798 | Abhd5  Set  Dhrs3  Repin1  Tsc1 | web link |
| nuclear membrane | Gene Ontology | GO:0031965 | 10 (1.74%) | 236 (1.13%) | 0.115794119488 | 0.265039873495 | Adra1a  Bcl2l1  Cept1  Sun1  Egfr  Nsmf  Dnajb12  Tmem176b  Rnf123  Repin1 | web link |
| ubiquitin ligase complex | Gene Ontology | GO:0000151 | 5 (0.87%) | 98 (0.47%) | 0.131897887671 | 0.295336574567 | Ubr3  Ankib1  Dcun1d3  Sharpin  Ube2j2 | web link |
| lysosomal membrane | Gene Ontology | GO:0005765 | 9 (1.57%) | 217 (1.04%) | 0.143492370416 | 0.314462003253 | Fnip1  Tmem175  Slc11a2  Slc39a14  Wdr59  Marchf8  Dram2  Tm9sf1  Sppl2b | web link |
| microtubule cytoskeleton | Gene Ontology | GO:0015630 | 8 (1.39%) | 190 (0.91%) | 0.152264874575 | 0.325345393624 | Strbp  Cdk16  Mtus1  Septin4  Mycbp2  Pcif1  Dtnbp1  Mid1ip1 | web link |
| postsynapse | Gene Ontology | GO:0098794 | 7 (1.22%) | 162 (0.77%) | 0.157934657099 | 0.325345393624 | Pdlim5  Ppp1ca  Snap23  Cpeb3  Nsmf  Slc29a1  Baiap2 | web link |
| ribonucleoprotein complex | Gene Ontology | GO:1990904 | 7 (1.22%) | 162 (0.77%) | 0.157934657099 | 0.325345393624 | Hsf1  Csnk1a1  Hnrnpa1  Hnrnpu  Zc3h14  G3bp2  Ssb | web link |
| mitochondrial matrix | Gene Ontology | GO:0005759 | 9 (1.57%) | 224 (1.07%) | 0.163694821771 | 0.327052178898 | Dglucy  Bcl2l1  Dnaja3  Mthfs  Alas2  Bckdhb  Etfbkmt  Nme4  Mettl17 | web link |
| apical plasma membrane | Gene Ontology | GO:0016324 | 14 (2.44%) | 383 (1.83%) | 0.169711013604 | 0.327052178898 | Slc39a14  Dram2  Ahcyl1  Slc38a3  Slc22a18  Fxyd1  Abcg8  Fas  Egfr  Pard6a  Slc29a1  Slc11a2  Cyp4a31  Abcc6 | web link |
| early endosome membrane | Gene Ontology | GO:0031901 | 6 (1.05%) | 136 (0.65%) | 0.170766745439 | 0.327052178898 | Tsg101  Washc1  Slc39a14  Egfr  Marchf8  Hsd17b6 | web link |
| ribosome | Gene Ontology | GO:0005840 | 8 (1.39%) | 196 (0.94%) | 0.171464249131 | 0.327052178898 | Mrpl36  Rpl38  Mrps2  Rpl35a  Mrps17  Rpl15  Uba52  Gm45713 | web link |
| synaptic vesicle | Gene Ontology | GO:0008021 | 7 (1.22%) | 167 (0.80%) | 0.175680026642 | 0.329000777165 | Scamp5  Slc40a1  Mff  Cdk16  Septin4  Bin1  Cadm1 | web link |
| fibrillar center | Gene Ontology | GO:0001650 | 6 (1.05%) | 139 (0.66%) | 0.182903081061 | 0.336411024094 | Ube2i  Nfib  Tcof1  Eef1d  Deaf1  Mbd6 | web link |
| chromosome | Gene Ontology | GO:0005694 | 19 (3.31%) | 557 (2.66%) | 0.194398577042 | 0.351281639216 | Hsf1  Mphosph8  Ints7  Hmces  Pmf1  Meiob  Hp1bp3  Timeless  Hnrnpu  Nup37  Hmga1  Nsd1  Ezh2  Mbd6  Nde1  Eed  Repin1  Setdb1  Csnk1a1 | web link |
| chromosome, centromeric region | Gene Ontology | GO:0000775 | 6 (1.05%) | 143 (0.68%) | 0.199594808844 | 0.354452850188 | Hsf1  Csnk1a1  Pmf1  Hnrnpu  Nup37  Nde1 | web link |
| basolateral plasma membrane | Gene Ontology | GO:0016323 | 9 (1.57%) | 239 (1.14%) | 0.211135684259 | 0.366251135096 | Slc38a3  Slc39a14  Cr1l  Egfr  Cask  Slc29a1  Abcc6  Cadm1  Slc40a1 | web link |
| apical part of cell | Gene Ontology | GO:0045177 | 5 (0.87%) | 116 (0.55%) | 0.213350175784 | 0.366251135096 | Afdn  Mgst1  Slc11a2  Nf2  Cdc42 | web link |
| lysosome | Gene Ontology | GO:0005764 | 14 (2.44%) | 411 (1.96%) | 0.239917025985 | 0.405105797975 | Fnip1  Sppl2b  Slc11a2  Slc39a14  Gns  Pcyox1  Rubcn  Slc17a2  Marchf8  Ifi30  Dram2  Tm9sf1  Wdr59  Tmem175 | web link |
| nuclear envelope | Gene Ontology | GO:0005635 | 7 (1.22%) | 186 (0.89%) | 0.249889065782 | 0.415138286702 | Ube2i  Slc22a18  Sun1  Nsmf  Agpat3  Bin1  Bnip2 | web link |
| actin cytoskeleton | Gene Ontology | GO:0015629 | 8 (1.39%) | 223 (1.06%) | 0.269286821602 | 0.44026258135 | Pdlim5  Tpm1  Mtss1  Ablim2  Baiap2  Lcp1  Sppl2b  Zyx | web link |
| protein-containing complex | Gene Ontology | GO:0032991 | 27 (4.70%) | 869 (4.15%) | 0.277925129248 | 0.447285754883 | Hsf1  Gigyf2  Stk11  Neb  Hnrnpu  Ncln  Nf2  Nf1  Clu  Tsc1  Esr1  Sdc1  Higd1a  Pard6a  Tpm1  Pxmp2  Cask  Slc12a7  Etfbkmt  Set  Mff  Desi1  Tmem258  Egfr  Xiap  Prkar1a  Cdc42 | web link |
| spindle pole | Gene Ontology | GO:0000922 | 5 (0.87%) | 133 (0.64%) | 0.301161638096 | 0.469054636023 | Hsf1  Birc6  Uxt  Hnrnpu  Dctn4 | web link |
| cell junction | Gene Ontology | GO:0030054 | 27 (4.70%) | 883 (4.22%) | 0.306313608578 | 0.469054636023 | Pdlim5  Snap23  Afdn  Ifi30  Pard6a  Magi1  Dtnbp1  Lcp1  Cadm1  Bcl2l1  Cdk16  Cpeb3  Lims2  Mapk15  Gtf2a2  Mink1  Nfia  Scamp5  Nsmf  Zyx  Dnaja3  Ppp1r13l  Mff  Egfr  Sharpin  Pja2  Bcar3 | web link |
| receptor complex | Gene Ontology | GO:0043235 | 7 (1.22%) | 200 (0.96%) | 0.309667138345 | 0.469054636023 | Ghr  Prlr  Egfr  Abcg8  Cr1l  Gfra1  Lifr | web link |
| trans-Golgi network | Gene Ontology | GO:0005802 | 7 (1.22%) | 200 (0.96%) | 0.309667138345 | 0.469054636023 | Slc11a2  Slc66a2  Birc6  Sys1  Golga1  Atp9b  Rnf215 | web link |
| spindle | Gene Ontology | GO:0005819 | 7 (1.22%) | 203 (0.97%) | 0.322843207663 | 0.48192536796 | Hnrnpu  Mapk15  Mms19  Map2k5  Nde1  Dido1  Cdc42 | web link |
| neuron projection | Gene Ontology | GO:0043005 | 16 (2.79%) | 512 (2.45%) | 0.331560059115 | 0.487866944126 | Nf1  Esr1  Fas  Ppp1ca  Cdk16  Cpeb3  Nsmf  Anks1  Nf2  Snap23  Dtnbp1  Baiap2  Clu  Flnb  Cadm1  Cdc42 | web link |
| cell-cell junction | Gene Ontology | GO:0005911 | 7 (1.22%) | 208 (0.99%) | 0.3449955985 | 0.500486572471 | Afdn  Cask  Lims2  Mapk15  Magi1  Cdc42  Cadm1 | web link |
| cell cortex | Gene Ontology | GO:0005938 | 6 (1.05%) | 177 (0.85%) | 0.357598511597 | 0.504542302226 | Trak1  Pard6a  Nsmf  Flnb  Dctn4  Tsc1 | web link |
| secretory granule | Gene Ontology | GO:0030141 | 5 (0.87%) | 144 (0.69%) | 0.360689147187 | 0.504542302226 | Baiap2  Fas  Cdc42  Vegfa  Snx10 | web link |
| spliceosomal complex | Gene Ontology | GO:0005681 | 6 (1.05%) | 178 (0.85%) | 0.36248670257 | 0.504542302226 | Srsf2  Hnrnpa1  Wac  Hnrnpu  Snrpa1  Sf3a2 | web link |
| late endosome | Gene Ontology | GO:0005770 | 5 (0.87%) | 149 (0.71%) | 0.387931071448 | 0.531080896881 | Tsg101  Washc1  Slc11a2  Rubcn  Ankrd13a | web link |
| myelin sheath | Gene Ontology | GO:0043209 | 6 (1.05%) | 184 (0.88%) | 0.391865516145 | 0.531080896881 | Tcp1  Uba52  Septin4  Ubc  Immt  Cdc42 | web link |
| cytoskeleton | Gene Ontology | GO:0005856 | 37 (6.45%) | 1285 (6.14%) | 0.402148541318 | 0.537938957867 | Hsf1  Tsg101  Gas2l1  Mms19  Zyx  Txndc9  Nf2  Nde1  Clu  Flnb  Bin1  Lcp1  Tsc1  Bcl2l1  Strbp  Pard6a  Csnk1a1  Tpm1  Mapk15  Tcp1  Mycbp2  Baiap2  Carmil1  Washc1  Cep43  Hnrnpu  Nsmf  Septin4  Dido1  Coro1b  Uxt  Snx10  Birc6  Slmap  Mid1ip1  Dctn4  Cdc42 | web link |
| cytoplasmic vesicle | Gene Ontology | GO:0031410 | 23 (4.01%) | 789 (3.77%) | 0.411091033215 | 0.542378737347 | Trappc8  Tfr2  Snap23  Tbc1d5  Scamp5  Tm9sf1  Dtnbp1  Clu  Bcl2l1  Cope  Sun1  Cdk16  Marchf8  Golga1  Fndc3a  Fgfr3  Washc1  Slc11a2  Rcbtb2  Mff  Mapk15  Trak1  Sec31a | web link |
| glutamatergic synapse | Gene Ontology | GO:0098978 | 14 (2.44%) | 473 (2.26%) | 0.423112180275 | 0.542378737347 | Adra1a  Porcn  Ngef  Ppp1ca  Snap23  Nsmf  Dtnbp1  Prkar1a  Baiap2  Ctbp1  Ndrg2  Rnf216  Bin1  Cadm1 | web link |
| vesicle | Gene Ontology | GO:0031982 | 5 (0.87%) | 156 (0.75%) | 0.425920430379 | 0.542378737347 | Cask  Gak  Bin1  Gigyf2  Vegfa | web link |
| neuronal cell body | Gene Ontology | GO:0043025 | 17 (2.96%) | 581 (2.77%) | 0.4265308517 | 0.542378737347 | Smurf1  Slc38a7  Pard6a  Cfh  Ghr  Ppp1ca  Fas  Cask  Elovl5  Gfra1  Xrn1  Dtnbp1  Baiap2  Flnb  Eif5a  Gtf2i  Cdc42 | web link |
| dendritic spine | Gene Ontology | GO:0043197 | 6 (1.05%) | 195 (0.93%) | 0.445525219449 | 0.559623141503 | Ppp1ca  Comt  Nsmf  Dtnbp1  Baiap2  Cdc42 | web link |
| growth cone | Gene Ontology | GO:0030426 | 5 (0.87%) | 177 (0.85%) | 0.535961641608 | 0.664658217083 | Clu  Ndrg2  Tsc1  Ngef  Dtnbp1 | web link |
| axon | Gene Ontology | GO:0030424 | 13 (2.26%) | 475 (2.27%) | 0.542051361505 | 0.664658217083 | Smurf1  Mink1  Slc38a7  Cfh  Afdn  Comt  Uhmk1  Gfra1  Mycbp2  Nf1  Dtnbp1  Bin1  Cadm1 | web link |
| chromatin | Gene Ontology | GO:0000785 | 9 (1.57%) | 333 (1.59%) | 0.564357805971 | 0.683868870765 | Uxt  Set  Esr1  Timeless  Hmga1  Ezh2  Smarca4  Ncor2  Ncor1 | web link |
| lamellipodium | Gene Ontology | GO:0030027 | 5 (0.87%) | 188 (0.90%) | 0.589640622429 | 0.706197489654 | Coro1b  Baiap2  Carmil1  Nf2  Tsc1 | web link |
| dendrite | Gene Ontology | GO:0030425 | 15 (2.61%) | 577 (2.76%) | 0.620109898369 | 0.734153098069 | Ube2i  Mink1  Eif5a  Xrn1  Trak1  Comt  Cask  Nsmf  Elovl5  Cpeb3  Nf1  Cdc42  Bin1  Sharpin  Cadm1 | web link |
| early endosome | Gene Ontology | GO:0005769 | 7 (1.22%) | 274 (1.31%) | 0.627589625563 | 0.734565129921 | Tsg101  Trio  Washc1  Slc11a2  Trak1  Rubcn  Nf2 | web link |
| transcription regulator complex | Gene Ontology | GO:0005667 | 6 (1.05%) | 257 (1.23%) | 0.710189883844 | 0.821905146471 | Apex1  Tle2  Zfpm1  Hnf1b  Tfdp1  Nrf1 | web link |
| membrane raft | Gene Ontology | GO:0045121 | 6 (1.05%) | 287 (1.37%) | 0.80204155815 | 0.917892005439 | Fas  Egfr  Cask  Cflar  Nf2  Kras | web link |
| cell projection | Gene Ontology | GO:0042995 | 25 (4.36%) | 1062 (5.07%) | 0.811799367118 | 0.918849833111 | Pdlim5  Fam149b  Nf2  Magi1  Lcp1  Tsc1  Csnk1a1  Cpeb3  Mapk15  Mycbp2  Baiap2  Ndrg2  Mink1  Carmil1  Cep43  Nsmf  Anks1  Anks3  Septin4  Gtf2i  Trio  Ngef  Elovl5  Cdc42  Ttc23 | web link |
| collagen-containing extracellular matrix | Gene Ontology | GO:0062023 | 6 (1.05%) | 296 (1.41%) | 0.824719206018 | 0.920619251483 | Angptl4  Vwa5a  Adamts10  Serpina1a  Clu  Itih4 | web link |
| microtubule | Gene Ontology | GO:0005874 | 7 (1.22%) | 342 (1.63%) | 0.831238741631 | 0.920619251483 | Gas2l1  Bin1  Tcp1  Nde1  Baiap2  Kifc2  Mid1ip1 | web link |
| membrane | Gene Ontology | GO:0016020 | 226 (39.37%) | 8771 (41.89%) | 0.900264975713 | 0.986460558494 | Sec22c  Pdlim5  Asph  Slc39a11  Coa8  Slc22a18  Apoo  Mpv17  Zdhhc20  Pcmtd1  Nf2  Magi1  Ubr3  Tmbim4  Phldb2  Nme4  Marchf8  Card19  Ggcx  Slc39a14  Ube2j2  Esr1  Fas  Zdhhc6  Asgr2  Neo1  Mtx3  Mpdu1  Rgs3  Pigx  Pxmp2  Cyp2r1  Slc39a7  Hccs  Scamp5  Bri3bp  Aldh3a2  Cyp3a59  Mars1  Wbp1l  Acer2  Rnf215  Crls1  Ahcyl1  Arv1  Ncln  Hsd17b6  Cdipt  Nipsnap1  Smco4  Enpp4  Snx10  Enpp2  Cyp4b1  Taz  Fgfr3  Slmap  Atp5j  Golgb1  AI182371  Gfra1  Pja2  Fnip1  Mtarc2  Dram2  Hsd3b2  Rdh11  Adgre5  Egfr  Cept1  Tbc1d5  Slc11a2  Sc5d  Stra6l  Sh2b1  Adipor2  Immt  Tmem87a  Bcl2l1  Mthfs  Sdc1  Xkr9  Ube2d3  Golga1  Lifr  Slc25a19  Baiap2  Fndc3a  Tmem175  Sptssa  Ifi27  Prkar1a  Ly6e  Washc1  Slc40a1  Ghr  Mgam  Plekhb1  Etfdh  Gnpat  F10  Ngef  Mff  Fxyd1  Tmem258  D11Wsu47e  Dcun1d3  Cyp4f13  Mpv17l2  Fdxr  Gba2  Sec31a  Kras  Tsg101  Sun1  Slc25a44  Nf1  Snap23  Tfr2  Slc17a2  Entpd8  Atp5mpl  Derl3  Vegfa  Pard6a  Clu  Tmem219  Mtmr4  Bin1  Cyp4a31  Pex16  Porcn  Abcg8  Comt  Wdr59  Rnf149  Dnajb12  Agpat3  Sgms2  Rpl15  Sppl2b  Stk11  Exoc7  St3gal5  St3gal4  Mtr  Ero1b  Tm4sf1  Gm45713  Dhrs3  Nsmf  Anks1  Arhgef12  Mia2  Tmem208  Sema4a  Abcc6  Irgm2  Steap3  Sema4g  Smurf1  Erap1  Tsc1  Ankrd13a  Cr1l  Paqr3  Plekha1  Mrap  Elovl5  Atp9b  Cox7a2  1810058I24Rik  Cln8  Eri2  Slc22a30  Dtnbp1  Stk16  Calu  Slc38a10  Tm9sf1  Naa60  Cyb561d1  Lcp1  Cadm1  Madd  Slc38a7  Pgap2  Slc66a2  Slc38a3  Cope  Pcyox1  Higd1a  Lims2  Ndufaf4  Ndufs2  Mgst1  Yif1b  Ndufaf2  Josd2  Retreg1  Lysmd3  Adra1a  Carmil1  Tmem176b  Prlr  Gm45808  Nras  Cask  Phkb  Slc12a7  Tmem38b  Susd1  Tmem222  Dnaja3  Tmprss6  Birc6  Trak1  Sys1  Mpzl2  Tmem143  Pnpla6  Slc29a1  Sec16b  Eif5a  Bcar3  Cdc42 | web link |
| cell surface | Gene Ontology | GO:0009986 | 12 (2.09%) | 655 (3.13%) | 0.949046544766 | 0.999999993224 | Prlr  Slc11a2  Ghr  Cr1l  Fas  Egfr  Hnrnpu  Atp5j  Vegfa  Sdc1  Clu  Fgfr3 | web link |
| external side of plasma membrane | Gene Ontology | GO:0009897 | 9 (1.57%) | 524 (2.50%) | 0.952486213448 | 0.999999993224 | Tfr2  Ghr  Cfh  Cr1l  Sdc1  Prlr  Gfra1  Lifr  Fas | web link |
| cilium | Gene Ontology | GO:0005929 | 5 (0.87%) | 375 (1.79%) | 0.977844244366 | 0.999999993224 | Ttc23  Anks3  Septin4  Fam149b  Csnk1a1 | web link |
| cellular\_component | Gene Ontology | GO:0005575 | 47 (8.19%) | 2346 (11.20%) | 0.993421166923 | 0.999999993224 | Plekhg3  Gpank1  Fam135a  Sbno2  9430038I01Rik  Cyp4f13  Szrd1  Csad  Fbrsl1  Plekha6  Zfand4  1700066M21Rik  Cyb561d1  Tmem219  Fam169b  Apol9b  Rin2  Gucd1  Wdcp  Cyp3a59  Pigx  Proser2  Ccdc159  Aasdh  Miip  L3hypdh  Fpgt  Zfp973  Kctd18  Gmppa  Haghl  2700062C07Rik  Wbp1l  Septin4  Susd1  Tmem222  Gpatch4  Mettl9  Qser1  Smco4  Fam214a  Josd2  Smim10l1  Wdr20  Lrrc28  Btf3l4  Nt5dc1 | web link |
| integral component of membrane | Gene Ontology | GO:0016021 | 152 (26.48%) | 6684 (31.92%) | 0.998267699654 | 0.999999993224 | Sec22c  Acer2  Slc39a11  Slc22a18  Xkr9  Mpv17  Zdhhc20  Hsd3b2  Ubr3  Tmbim4  Gm45844  Cyp4a31  Card19  Ggcx  Slc39a14  Ube2j2  Tmem258  Fas  Sun1  1810058I24Rik  Neo1  Mpdu1  Cyp4f13  Rnf215  Pxmp2  Cyp2r1  Slc39a7  Scamp5  Slc11a2  Abcg8  Cyp3a59  Mars1  Wbp1l  Crls1  Arv1  Ncln  Cdipt  Zdhhc6  Enpp4  Smco4  Enpp2  Cyp4b1  Stra6l  Fgfr3  Slmap  Golgb1  AI182371  Pgap2  Tfr2  Dram2  Adgre5  Egfr  Asph  Retreg1  Sc5d  Taz  Immt  Tmem87a  Bcl2l1  Sdc1  Eri2  Lifr  Slc25a19  Fndc3a  Tmem175  Sptssa  Mgam  Atp9b  Madd  Slc40a1  Ghr  Ifi27  Plekhb1  Mff  Fxyd1  D11Wsu47e  Mgst1  Mpv17l2  Gba2  Bri3bp  Slc25a44  Gigyf2  Slc17a2  Entpd8  Atp5mpl  Derl3  Tmem219  Pex16  Porcn  Comt  Tmprss6  Rnf149  Dnajb12  Agpat3  Sgms2  Sppl2b  Slc66a2  St3gal4  Mtr  Cept1  Tm4sf1  Dhrs3  Pigx  Mia2  Tmem208  Sema4a  Abcc6  Steap3  Sema4g  Erap1  Apoo  Cr1l  Paqr3  Mrap  Elovl5  Rdh11  Cox7a2  Cln8  Slc22a30  Slc38a10  Tm9sf1  Tmem176b  Cyb561d1  Cadm1  Slc38a7  Tmem38b  St3gal5  Slc38a3  Pcyox1  Higd1a  Marchf8  Aldh3a2  Asgr2  Yif1b  Mthfs  Josd2  Lysmd3  Adra1a  Carmil1  Adipor2  Prlr  Cask  Slc12a7  Gm45713  Susd1  Tmem222  Anks1  Sys1  Mpzl2  Tmem143  Pnpla6  Slc29a1 | web link |
| extracellular region | Gene Ontology | GO:0005576 | 32 (5.57%) | 1829 (8.73%) | 0.998439995914 | 0.999999993224 | Apoo  C4bp  Calu  Ifi30  Pkdcc  Angptl4  Vegfa  1700066M21Rik  Igfbp4  Clu  Proc  Apol9b  Fas  Lifr  Apoc3  Rnase4  Ghr  Masp1  Ccl27a  F10  Erap1  Tsku  Cfh  Selenop  Enpp2  Adamts10  Serpina1a  Fetub  Habp2  Sdc1  AI182371  Itih4 | web link |
| integral component of plasma membrane | Gene Ontology | GO:0005887 | 18 (3.14%) | 1203 (5.75%) | 0.998828016172 | 0.999999993224 | Adra1a  Tfr2  Slc11a2  Ghr  Slc38a3  Abcg8  Enpp2  Tm4sf1  Egfr  Slc17a2  Slc12a7  Neo1  Sgms2  Slc29a1  Sema4a  Slc39a14  Fgfr3  Sema4g | web link |
| extracellular space | Gene Ontology | GO:0005615 | 28 (4.88%) | 1732 (8.27%) | 0.999487086473 | 0.999999993224 | Ces3a  Masp1  Serpina1a  Gfra1  Igfbp4  Clu  Proc  Fas  Tmprss6  Serpina11  Rnase4  Ghr  Ccl27a  Sema4a  F10  Sema4g  Tsku  Cfh  Serpina3n  Serpina7  Selenop  Enpp2  Slmap  Atp5j  Angptl4  Fetub  Vegfa  Itih4 | web link |
| plasma membrane | Gene Ontology | GO:0005886 | 96 (16.72%) | 5547 (26.49%) | 0.999999993224 | 0.999999993224 | Slc39a11  Slc39a14  Afdn  Nf2  Magi1  Gm45844  Abcc6  Esr1  Fas  Cdk16  Neo1  Rgs3  Scamp5  Slc11a2  Arl14ep  Baiap2  Zdhhc20  Cdipt  Cfh  Enpp4  Egfr  Stra6l  Slmap  Gfra1  Pja2  Tfr2  Dram2  Adgre5  Asph  Ly6e  Raph1  Plekha1  Sh2b1  Flnb  Adra1a  Xkr9  Ube2d3  Cpeb3  Lifr  Phf7  Fgfr3  Madd  Slc40a1  Ghr  Zyx  Fxyd1  Commd4  Kras  Tsg101  Snap23  Entpd8  Coro1b  Vegfa  Pard6a  Tmem219  Bin1  Mphosph8  Comt  Tmprss6  Dtnb  Sgms2  Sppl2b  Rnf146  Exoc7  Nsmf  Sema4a  Phldb2  Sema4g  Smurf1  Erap1  Ahcyl1  Ankrd13a  Ppp1ca  Mrap  Prkar1a  Itih4  Stk16  Dtnbp1  Lcp1  Cadm1  Slc38a3  Pcyox1  Lims2  Abcg8  Xrn1  Lysmd3  Carmil1  Adipor2  Cask  Phkb  Slc12a7  Dcun1d3  Dnaja3  Tsku  Slc29a1  Cdc42 | web link |
