# Supplementary material for: Regulation of Alternative Splicing of Lipid Metabolism Genes in Sepsis-Induced Liver Damage by RNA-Binding Proteins
Source: Inflammation. 2024 May 9;47(6):1952–68. doi: 10.1007/s10753-024-02017-2 (PMC11607010; doi:10.1007/s10753-024-02017-2)
Supplement: Supplementary file 1 — Supplementary file1 (ZIP 38655 KB) [file 10753_2024_2017_MOESM1_ESM.zip › Sup5_AS_GO.zip!/Sup5_AS_GO/ras/CLPDCA_vs_Sham/Ras_GO_analysis/Ras_GO_enrichment_web_F.html]

GO enrichment analyze


# GO enrichment analyze result


| Term | Database | ID | Input number (547) | Background number (20698) | P-Value | Corrected P-Value | Input | Hyperlink |
| --- | --- | --- | --- | --- | --- | --- | --- | --- |
| transferase activity | Gene Ontology | GO:0016740 | 85 (15.54%) | 1771 (8.56%) | 4.77840369495e-08 | 4.15721121461e-06 | Ube2i  Pcmtd1  Gstm4  Mettl17  Rnf181  Map2k5  Stk11  Cept1  Sugct  Zdhhc20  St3gal4  Ezh2  Naa60  Ankib1  Rps6kb2  Mapkapk2  Ubr3  Rnf149  Ube2d3  Porcn  Mvk  Csnk1a1  Ube2j2  Poglut1  Cdk12  Pcyt2  Smurf1  Cdk16  Comt  Gsta3  Pkdcc  Marchf8  Mapk15  Mast3  Agpat3  Mycbp2  Pcif1  Nsd1  Trim26  Mat1a  Rnf216  Rnf123  Setdb1  Tut4  Sgms2  Mink1  Agxt2  Fpgt  St3gal5  Upp2  Mtr  Stk16  Gmppa  Uhmk1  Cask  Alas2  Ppip5k2  Clk4  Etfbkmt  Gnpat  Crls1  Xylb  Eef1akmt2  Cdipt  Kmt2c  Rnf146  Trio  Nme4  Pi4ka  Zdhhc6  Birc6  Parp6  Elovl5  Egfr  Nudt5  Fgfr3  Osgepl1  Xiap  Mgst1  Sult2a8  Pja1  Gba2  Pja2  Setd4  Abhd5 | web link |
| nucleic acid binding | Gene Ontology | GO:0003676 | 35 (6.40%) | 594 (2.87%) | 9.3489303532e-06 | 0.00031523813871 | Srsf5  Tsen34  Gpank1  Srsf2  Poldip3  Ddx56  Zfp638  Ankhd1  Ptbp3  Ptbp1  Hnrnpa1  Eri2  Cpeb3  Xrn1  Ssb  Gpatch4  Rexo4  Rnase4  Uhmk1  Snhg4  Pcbp2  Gm27029  Nabp2  Hnrnpll  Safb  Fubp3  Enpp2  Rbm25  Srsf11  Sf3a2  G3bp2  Cpsf7  Tardbp  Tut4  Tut7 | web link |
| chromatin binding | Gene Ontology | GO:0003682 | 31 (5.67%) | 499 (2.41%) | 1.08702806452e-05 | 0.00031523813871 | Hsf1  Meiob  Wac  Hnrnpu  Ezh2  Ctbp1  Kdm5a  Smarcc2  Mphosph8  Foxp1  Esr1  Safb  Hmga1  Mapk15  Mbd6  Nudt21  Smarca4  Setdb1  Nfia  Zfp932  Sfmbt1  Eed  Uxt  Set  Hp1bp3  Tdrd3  Egfr  Bahd1  Phc2  Ssbp1  Ncor2 | web link |
| oxidoreductase activity | Gene Ontology | GO:0016491 | 38 (6.95%) | 684 (3.30%) | 1.47330511135e-05 | 0.00032044386172 | Tdo2  Cyp4f13  Idh1  Asph  Dhrs3  Ifi30  Akr1e1  Dhtkd1  Sc5d  Hsd3b2  Cyb561d1  Ctbp1  Kdm5a  Paox  Ero1b  D2hgdh  Ndufs2  Cyp2r1  Ido2  Jmjd4  Aldh3a2  Cyp3a59  Etfdh  Aldh16a1  P4ha1  Bckdhb  Adhfe1  Cyp4a31  Cox7a2  Hsd17b6  Apex1  Cyp4b1  Pcyox1  Mtarc2  Rdh11  Fdxr  Akr1c6  Prodh2 | web link |
| protein binding | Gene Ontology | GO:0005515 | 224 (40.95%) | 6748 (32.60%) | 2.02115730905e-05 | 0.000351681371775 | Pdlim5  Gpank1  Gas2l1  Sbno2  Gemin6  Afdn  Plekhb1  Hnrnpu  Nf2  Magi1  Eed  Ubr3  Ctbp1  Kdm5a  Tfdp1  Asap3  Mapk15  Spop  Fas  Irf9  Sun1  Cdk16  Dcaf11  Nup37  Tcp1  Neo1  Rgs3  Sema4g  Smarca4  Slc39a7  Midn  Hccs  Arap2  Zbtb14  Abcg8  Ubc  Arhgef12  Rubcn  Sfmbt1  Pcbp2  Baiap2  Nipsnap1  Cfh  Zdhhc6  Cops2  Aasdh  Desi1  Egfr  Stra6l  Slmap  Ezh2  Pja1  Esr1  P4ha1  Ppp1r13l  Ssbp4  Mid1ip1  Abhd5  Tsen34  Tfr2  Gabpb1  Nf1  Zbtb7c  C4bp  Asph  Thra  Raph1  Plekha6  Zfand4  Cnpy2  Nde1  Sh2b1  Zfp932  Flnb  Deaf1  Immt  Bcl2l1  Pard6a  Trafd1  Foxk2  Pmf1  Sdc1  Setd4  Safb  Ube2d3  Cpeb3  Mapkapk2  Golga1  Lifr  Pcif1  Fndc3a  Taf13  Rnf123  Setdb1  Slc40a1  Ghr  Mphosph8  Cdc42  Timeless  Uhmk1  Myl6  Kmt2c  Mycbp2  Cep43  Gnpat  Gtf2i  Zyx  Tagln  Hnf1b  Pja2  Ngef  Mff  Ppp1ca  Tdrd3  Selenop  Ncor2  Xiap  Dtnbp1  Eloc  Cln8  Sec31a  Kras  Tsg101  Meiob  Cnot10  Gstm4  Gigyf2  Fnip1  Wac  Ppp4r1  Coro1b  Kif13a  Serpina1a  Vegfa  Tmprss6  Ankib1  Clu  Bin1  Porcn  Ubap2l  Wdr59  Rnf149  Poglut1  Dtnb  Nlrc5  Nudt21  Ctdsp1  Rnf146  Emsy  Stk11  Exoc7  Dnaja3  Eif4g3  Neb  Btrc  Sh3glb2  Nsmf  Anks1  Anks3  Mia2  Sema4a  Ndufs2  Smurf1  Ahcyl1  Ankrd13a  Trim26  Snrpa1  Fbxo3  Prkar1a  Fbxo6  Tardbp  Itih4  Ttc23  Ube2i  Trappc8  Srsf5  Srsf2  Tle2  Ankhd1  Snap23  Atg13  Igfbp4  Tmem176b  Lcp1  Gsta3  Tsc1  Klhl13  Tcof1  Cope  Tpm1  Uba52  Map2k5  Anp32a  Nsd1  Cadm1  Thoc7  Smarcc2  Carmil1  Cflar  Adipor2  Prlr  Chtop  Cask  Foxp1  Rcbtb2  Nrf1  Gstp3  Trio  Tsku  Zfp467  Birc6  Zfpm1  Ppfibp1  Zfp131  Wdr27  Mast3  Wdr20  Phc2  Swi5  Septin4  Gprasp1  Tut4  Sharpin  Lrrc28 | web link |
| protein-containing complex binding | Gene Ontology | GO:0044877 | 34 (6.22%) | 609 (2.94%) | 3.76736354606e-05 | 0.000546267714179 | Tsg101  Poldip3  Stk11  Tbc1d5  Thra  Coro1b  Derl3  Clu  Cflar  Bin1  Tsc1  Bcl2l1  Esr1  Fas  Ndufaf2  Smarca4  Nme4  Carmil1  Scamp5  Nras  Cask  Hnrnpu  Hnf1b  Bckdhb  Dnaja3  Mff  Ppp1ca  Egfr  Atp5j  Apex1  Eloc  Ncor2  Sharpin  Kras | web link |
| single-stranded DNA binding | Gene Ontology | GO:0003697 | 11 (2.01%) | 103 (0.50%) | 8.82406983169e-05 | 0.00109670582194 | Meiob  Hmces  Fubp3  Hnrnpa1  Hnrnpu  Ssbp4  Pcbp2  Rexo4  Ssbp1  Nabp2  Polr2h | web link |
| metal ion binding | Gene Ontology | GO:0046872 | 127 (23.22%) | 3545 (17.13%) | 0.000137170992153 | 0.00149173453967 | Acer2  Pdlim5  Asph  Cyp4f13  Rnf44  Haghl  Ubr3  Rsl1  Kdm5a  Asap3  Gm28557  Marchf8  Esr1  Rnf181  Zc3h13  Zc3h14  Rnf215  Cyp2r1  Hccs  Zc3h11a  Zbtb14  Abcg8  Cyp3a59  Osgepl1  Ablim2  Zdhhc20  Ing1  Rnf123  Enpp4  Enpp2  Nudt5  Pja1  Pja2  Zyx  Bpnt1  Zbtb7c  Idh1  Cept1  Pter  Cyp4b1  Cpsf4l  Zfand4  Zfp932  Foxp1  Trafd1  Idh3g  Eri2  Mycbp2  Phf7  Zswim8  Zfp995  Nme4  Gss  2010315B03Rik  Zfp973  Mvk  Dido1  Ppp1ca  Zc3h4  Xiap  Zfp62  Masp1  Entpd8  Zkscan1  Zfp638  Ankib1  Mtmr4  Comt  Rnf149  Dtnb  Gm14403  Repin1  Ctdsp1  Rnf146  Metap1d  Hagh  Deaf1  Calu  P4ha1  Cyp4a31  Thra  Kmt2c  Erap1  Rnf216  Ampd2  Trim26  Atp9b  Thap2  Tdo2  Gns  Stk11  Rnpepl1  Apex1  Fbp1  Cyb561d1  Lcp1  Phf20l1  Zfp141  Lims2  Mtr  Ndufs2  Zfp950  Zfp874a  Map2k5  Mthfs  Nsd1  Ido2  Adipor2  Jmjd4  Prlr  Etfdh  Zfp235  Adhfe1  Mcee  Foxk2  Dnaja3  Zfp467  Zfpm1  Mat1a  Zfp131  Adamts10  Zfp946  Phc2  2610044O15Rik8  Tut4  Sharpin  Nt5dc1 | web link |
| RNA binding | Gene Ontology | GO:0003723 | 42 (7.68%) | 915 (4.42%) | 0.000385764003302 | 0.00372905203192 | Srsf5  Srsf2  Poldip3  Hnrnpu  Nudt5  Apex1  Rpl15  Ezh2  Zfp638  Ankhd1  Ptbp3  Ptbp1  Strbp  Hnrnpa1  Safb  Cpeb3  Xrn1  Nudt21  Thoc7  Scaf1  Eif4g3  Tardbp  Chtop  Uhmk1  Mars1  Snhg4  Pcbp2  Fubp3  Hnrnpll  Ahcyl1  Tdrd3  Smarca4  Ddx56  Snrpa1  Mrps17  Rbm25  Srsf11  Zc3h14  G3bp2  Cpsf7  Ssb  Eif5a | web link |
| chromatin DNA binding | Gene Ontology | GO:0031490 | 7 (1.28%) | 64 (0.31%) | 0.00146321464315 | 0.0126477861519 | Hsf1  Sbno2  Hnrnpu  Thra  Apex1  Ezh2  Kdm5a | web link |
| transcription corepressor activity | Gene Ontology | GO:0003714 | 12 (2.19%) | 168 (0.81%) | 0.00174400300767 | 0.0126477861519 | Tsg101  Uxt  Cops2  Tle2  Zfpm1  Hnrnpu  Sfmbt1  Ezh2  Ctbp1  Smarca4  Ncor2  Ncor1 | web link |
| ubiquitin-protein transferase activity | Gene Ontology | GO:0004842 | 15 (2.74%) | 238 (1.15%) | 0.00174452222785 | 0.0126477861519 | Smurf1  Ube2i  Klhl13  Birc6  Btrc  Rnf181  Ube2d3  Marchf8  Ubr3  Xiap  Ankib1  Pja2  Rnf123  Sharpin  Rnf146 | web link |
| magnesium ion binding | Gene Ontology | GO:0000287 | 14 (2.56%) | 217 (1.05%) | 0.0019749072077 | 0.0132166866977 | Gss  Bpnt1  Csnk1a1  Idh3g  Idh1  Stk11  Comt  Nudt5  Mast3  Septin4  Atp9b  Mat1a  Mvk  Foxk2 | web link |
| ubiquitin protein ligase activity | Gene Ontology | GO:0061630 | 17 (3.11%) | 294 (1.42%) | 0.00223654786812 | 0.0138985474662 | Smurf1  Rnf149  Rnf215  Trim26  Btrc  Rnf181  Ube2d3  Ankib1  Marchf8  Pja1  Xiap  Mycbp2  Fbxo6  Cdc42  Ubr3  Rnf216  Rnf146 | web link |
| mRNA binding | Gene Ontology | GO:0003729 | 13 (2.38%) | 211 (1.02%) | 0.00417171758242 | 0.022138147949 | Srsf5  Zc3h11a  Fubp3  Rbm25  Gm45713  Pcbp2  G3bp2  Ptbp3  Nudt21  Mvk  Hnrnpll  Thoc2l  Luc7l2 | web link |
| methyltransferase activity | Gene Ontology | GO:0008168 | 12 (2.19%) | 187 (0.90%) | 0.00420369537906 | 0.022138147949 | Kmt2c  Pcmtd1  Etfbkmt  Mettl17  Mtr  Comt  Setd4  Ezh2  Pcif1  Nsd1  Eef1akmt2  Setdb1 | web link |
| GTPase binding | Gene Ontology | GO:0051020 | 5 (0.91%) | 41 (0.20%) | 0.00432584500152 | 0.022138147949 | Bcl2l1  Nme4  Lcp1  Bin1  Mff | web link |
| ribonucleoprotein complex binding | Gene Ontology | GO:0043021 | 5 (0.91%) | 43 (0.21%) | 0.00532225026839 | 0.0247707803697 | Uhmk1  Ezh2  Rpl35a  Hnrnpu  Ppp1ca | web link |
| protein N-terminus binding | Gene Ontology | GO:0047485 | 9 (1.65%) | 123 (0.59%) | 0.00540971065545 | 0.0247707803697 | Pdlim5  Klhl13  Tpm1  Clu  Tsc1  Smarca4  Ncor2  Dctn4  Eif5a | web link |
| catalytic activity | Gene Ontology | GO:0003824 | 20 (3.66%) | 405 (1.96%) | 0.00583692811117 | 0.0253906372836 | Upp2  Fahd2a  Pcyt2  Hoga1  Auh  Mgam  D2hgdh  Enpp2  Gns  Rtca  Agxt2  Echdc2  Alas2  Mtarc2  Bckdhb  Fbp1  Hsd3b2  Gnpat  Enpp4  Csad | web link |
| identical protein binding | Gene Ontology | GO:0042802 | 63 (11.52%) | 1735 (8.38%) | 0.00618480656646 | 0.025622770061 | Hsf1  Tdo2  Tysnd1  Idh1  Hnrnpu  Mat1a  Coro1b  Mmaa  Angptl4  Vegfa  Fbp1  Nde1  Ctbp1  Lcp1  Bin1  Flnb  Bcl2l1  Foxp1  Esr1  Sdc1  Tmem38b  Tpm1  0610010K14Rik  Mgst1  Mycbp2  Baiap2  Nudt21  Emsy  Fgfr3  Mtss1  Gss  Adipor2  Slc40a1  Ghr  Tardbp  Sh3glb2  Hnf1b  P4ha1  Septin4  Cdc42  Eed  Mvk  Psmc6  Upp2  Fas  Ahcyl1  Cfh  Mff  Desi1  Ampd2  Cggbp1  Egfr  Nudt5  Mrap  Xiap  Serpina1a  Srsf11  Sharpin  Phc2  Ssbp1  Ppp1r13l  Mid1ip1  Kras | web link |
| exonuclease activity | Gene Ontology | GO:0004527 | 5 (0.91%) | 48 (0.23%) | 0.00850320193302 | 0.0336262985533 | Apex1  Meiob  Eri2  Xrn1  Rexo4 | web link |
| oxidoreductase activity, acting on the CH-OH group of donors, NAD or NADP as acceptor | Gene Ontology | GO:0016616 | 5 (0.91%) | 50 (0.24%) | 0.01008029375 | 0.0381298067933 | Ctbp1  Idh1  Dhrs3  Idh3g  Hsd3b2 | web link |
| transferase activity, transferring acyl groups | Gene Ontology | GO:0016746 | 10 (1.83%) | 170 (0.82%) | 0.0151621020255 | 0.0549626198423 | Porcn  Zdhhc6  Gnpat  Osgepl1  Taz  Alas2  Agpat3  Naa60  Zdhhc20  Abhd5 | web link |
| dioxygenase activity | Gene Ontology | GO:0051213 | 6 (1.10%) | 83 (0.40%) | 0.022593551471 | 0.0786255591192 | Ido2  Tdo2  Jmjd4  Asph  P4ha1  Kdm5a | web link |
| promoter-specific chromatin binding | Gene Ontology | GO:1990841 | 5 (0.91%) | 66 (0.32%) | 0.0302516272091 | 0.101226598738 | Hsf1  Ezh2  Hnrnpu  Setdb1  Esr1 | web link |
| lyase activity | Gene Ontology | GO:0016829 | 9 (1.65%) | 167 (0.81%) | 0.0337138987557 | 0.108633673768 | Dglucy  Hoga1  Ggcx  L3hypdh  Tsen34  Gstm4  Csad  Hccs  Echdc2 | web link |
| protein serine kinase activity | Gene Ontology | GO:0106310 | 15 (2.74%) | 340 (1.64%) | 0.0375693244636 | 0.115160335647 | Mink1  Trio  Csnk1a1  Cdk12  Stk16  Stk11  Cdk16  Uhmk1  Cask  Rps6kb2  Mapk15  Mast3  Map2k5  Clk4  Mapkapk2 | web link |
| protein threonine kinase activity | Gene Ontology | GO:0106311 | 15 (2.74%) | 341 (1.65%) | 0.0383867785492 | 0.115160335647 | Mink1  Trio  Csnk1a1  Cdk12  Cdk16  Stk11  Stk16  Uhmk1  Cask  Rps6kb2  Mapk15  Mast3  Map2k5  Clk4  Mapkapk2 | web link |
| protease binding | Gene Ontology | GO:0002020 | 7 (1.28%) | 123 (0.59%) | 0.0449264872935 | 0.125550403643 | Fas  Tysnd1  Cflar  Xiap  Serpina1a  Ubc  Bin1 | web link |
| ATP binding | Gene Ontology | GO:0005524 | 50 (9.14%) | 1484 (7.17%) | 0.0457513619329 | 0.125550403643 | Ube2i  Egfr  Rtca  Stk11  Stk16  Hnrnpu  Entpd8  Kif13a  Pkdcc  Nadsyn1  Magi1  Aasdh  Mapkapk2  Nme4  Xylb  Csnk1a1  Ube2j2  Cdk12  Cdk16  Ube2d3  Ppip5k2  Mapk15  Tcp1  Gak  Map2k5  Nlrc5  Clk4  Fgfr3  Mink1  Gss  Idh3g  Abcg8  Mthfs  Uhmk1  Cask  Mars1  Gm27029  Abcc6  Mvk  Psmc6  Ntpcr  Dnaja3  Trio  Smarca4  Ddx56  Mat1a  Rps6kb2  Mast3  Atp9b  Kifc2 | web link |
| actin filament binding | Gene Ontology | GO:0051015 | 10 (1.83%) | 205 (0.99%) | 0.0464171610891 | 0.125550403643 | Gas2l1  Neb  Afdn  Egfr  Tpm1  Coro1b  Ablim2  Lcp1  Bin1  Tagln | web link |
| kinase activity | Gene Ontology | GO:0016301 | 24 (4.39%) | 631 (3.05%) | 0.0484309521548 | 0.125550403643 | Stk11  Stk16  Pkdcc  Mapkapk2  Nme4  Mvk  Csnk1a1  Pi4ka  Cdk12  Cdk16  Ppip5k2  Mapk15  Sgms2  Map2k5  Clk4  Fgfr3  Mink1  Uhmk1  Cask  Xylb  Trio  Egfr  Rps6kb2  Mast3 | web link |
| histone binding | Gene Ontology | GO:0042393 | 8 (1.46%) | 152 (0.73%) | 0.0491035169145 | 0.125550403643 | Smarcc2  Sbno2  Set  Phc2  Sfmbt1  Anp32a  Smarca4  Kdm5a | web link |
| zinc ion binding | Gene Ontology | GO:0008270 | 27 (4.94%) | 730 (3.53%) | 0.0505087830749 | 0.125550403643 | Pter  Thra  Zfand4  Zfp638  Ubr3  Kdm5a  Trafd1  Esr1  Eri2  Marchf8  Dtnb  Mycbp2  Nsd1  Zswim8  Setdb1  Rnf146  Slc11a2  Mtr  Rnpepl1  Zdhhc20  Erap1  Trim26  Enpp2  Sf3a2  Phc2  Tut4  Tut7 | web link |
| heme binding | Gene Ontology | GO:0020037 | 9 (1.65%) | 183 (0.88%) | 0.0546271664749 | 0.132015652314 | Ido2  Hccs  Tdo2  Cyp4f13  Cyp4b1  Cyp3a59  Cyb561d1  Cyp4a31  Cyp2r1 | web link |
| RNA polymerase II cis-regulatory region sequence-specific DNA binding | Gene Ontology | GO:0000978 | 38 (6.95%) | 1101 (5.32%) | 0.0565489897115 | 0.132966543376 | Hsf1  Zbtb7c  Stk16  Hnrnpu  Thra  Zkscan1  Ezh2  Zfp141  Rsl1  Gm28557  Foxp1  Foxk2  Esr1  Irf9  Safb  Zfp950  Zfp874a  Nsd1  Nlrc5  Smarca4  Zfp995  Gm14403  Nfib  Nfia  Zfp932  2010315B03Rik  Zbtb14  Zfp973  Hnf1b  Zfp235  Eed  Zfp467  Zfp131  Nrf1  Zfp946  2610044O15Rik8  Tardbp  Zfp62 | web link |
| protein homodimerization activity | Gene Ontology | GO:0042803 | 25 (4.57%) | 676 (3.27%) | 0.0582428141327 | 0.133345390251 | Tsg101  Idh1  Masp1  Thra  Naa60  Mmaa  Ctbp1  Cadm1  Bcl2l1  Tpm1  Gtf2a2  Nudt21  Cyp2r1  Nrf1  Hoga1  Gss  Ghr  Aldh3a2  Gstm4  Cep43  Hnf1b  Plekhb1  Sppl2b  Mff  Nudt5 | web link |
| transcription regulatory region sequence-specific DNA binding | Gene Ontology | GO:0000976 | 11 (2.01%) | 246 (1.19%) | 0.0631471325688 | 0.140531204405 | Hsf1  Gabpb1  Foxk2  Zbtb14  Ezh2  Mterf3  Irf9  Thra  Hnf1b  Foxp1  Kdm5a | web link |
| ubiquitin protein ligase binding | Gene Ontology | GO:0031625 | 13 (2.38%) | 307 (1.48%) | 0.0653084514306 | 0.140531204405 | Tsg101  Washc1  Ube2j2  Slc22a18  Spop  Egfr  Uba52  Tcp1  Ndufs2  Pcbp2  Prkar1a  Ubc  Clu | web link |
| hydrolase activity | Gene Ontology | GO:0016787 | 55 (10.05%) | 1696 (8.19%) | 0.0662273492023 | 0.140531204405 | Acer2  Meiob  Cmbl  Bpnt1  Tysnd1  Masp1  Entpd8  Apex1  Fbp1  Mmaa  Mtmr4  Proc  Haghl  Hmces  Mtmr14  Kras  Eri2  Tmprss6  Atg4c  Xrn1  Bphl  Nudt21  Josd2  Smarca4  Ctdsp1  Rexo4  Gns  Rnase4  Hagh  Metap1d  Mgam  Rnpepl1  Pter  Enpp2  Nt5dc1  F10  Psmc6  Ntpcr  Erap1  Sppl2b  Ces3a  Desi1  Ampd2  Pgpep1l  Ppp1ca  Ddx56  Nudt5  Nudt6  Adamts10  Pnpla6  Habp2  Gba2  Wdyhv1  Enpp4  Cdc42 | web link |
| nucleotidyltransferase activity | Gene Ontology | GO:0016779 | 5 (0.91%) | 84 (0.41%) | 0.0716429587571 | 0.144983006104 | Pcyt2  Fpgt  Gmppa  Nudt5  Tut4 | web link |
| nuclease activity | Gene Ontology | GO:0004518 | 7 (1.28%) | 137 (0.66%) | 0.0716582673849 | 0.144983006104 | Tsen34  Rnase4  Meiob  Eri2  Apex1  Rexo4  Xrn1 | web link |
| transcription factor binding | Gene Ontology | GO:0008134 | 14 (2.56%) | 349 (1.69%) | 0.0810930554161 | 0.160343086846 | Bcl2l1  Dnaja3  Nfia  Foxp1  Esr1  Zfpm1  Hmga1  Ube2i  Gtf2a2  Thra  Ctbp1  Ppp1r13l  Smarca4  Tfdp1 | web link |
| transmembrane receptor protein tyrosine kinase activity | Gene Ontology | GO:0004714 | 6 (1.10%) | 115 (0.56%) | 0.0846287977946 | 0.163615675736 | Egfr  Stk16  Pkdcc  Map2k5  Clk4  Fgfr3 | web link |
| structural constituent of ribosome | Gene Ontology | GO:0003735 | 8 (1.46%) | 172 (0.83%) | 0.0867421312686 | 0.164055770008 | Mrpl36  Rpl38  Mrps2  Rpl35a  Mrps17  Rpl15  Uba52  Gm45713 | web link |
| oxidoreductase activity, acting on paired donors, with incorporation or reduction of molecular oxygen | Gene Ontology | GO:0016705 | 6 (1.10%) | 119 (0.57%) | 0.0958760464366 | 0.177472681702 | Cyp4f13  Cyp4b1  P4ha1  Cyp3a59  Cyp4a31  Cyp2r1 | web link |
| nucleotide binding | Gene Ontology | GO:0000166 | 56 (10.24%) | 1795 (8.67%) | 0.108977969406 | 0.197522569548 | Ube2i  Egfr  Rtca  Stk11  Stk16  Hnrnpu  Entpd8  Kif13a  Pkdcc  Nadsyn1  Magi1  Mmaa  Aasdh  Mapkapk2  Mtif2  Nme4  Mvk  Csnk1a1  Ube2j2  Cdk12  Cdk16  Ube2d3  Ppip5k2  Mapk15  Tcp1  Fpgt  Map2k5  Nlrc5  Clk4  Fgfr3  Prkar1a  Mink1  Gss  Idh3g  Abcg8  Mthfs  Nras  Uhmk1  Cask  Mars1  Septin4  Gm27029  Abcc6  Xylb  Psmc6  Ntpcr  Trio  Smarca4  Ddx56  Mat1a  Rps6kb2  Mast3  Atp9b  Cdc42  Kifc2  Kras | web link |
| protein kinase activity | Gene Ontology | GO:0004672 | 19 (3.47%) | 533 (2.58%) | 0.116292079839 | 0.2057805268 | Mink1  Trio  Csnk1a1  Cdk12  Egfr  Stk11  Stk16  Uhmk1  Cask  Mapkapk2  Mapk15  Pkdcc  Mast3  Gak  Map2k5  Rps6kb2  Clk4  Fgfr3  Cdk16 | web link |
| iron ion binding | Gene Ontology | GO:0005506 | 8 (1.46%) | 185 (0.89%) | 0.118264670575 | 0.2057805268 | Slc11a2  Cyp3a59  Cyp4b1  Sc5d  P4ha1  Cyp4f13  Cyp4a31  Cyp2r1 | web link |
| guanyl-nucleotide exchange factor activity | Gene Ontology | GO:0005085 | 9 (1.65%) | 218 (1.05%) | 0.125126908765 | 0.213451785541 | Fnip1  Trio  Dock9  Ngef  Plekhg3  Madd  Mycbp2  Arhgef12  Bcar3 | web link |
| protein serine/threonine kinase activity | Gene Ontology | GO:0004674 | 15 (2.74%) | 412 (1.99%) | 0.132775676227 | 0.221584122019 | Mink1  Trio  Csnk1a1  Cdk12  Stk16  Stk11  Cdk16  Uhmk1  Cask  Rps6kb2  Mapk15  Mast3  Map2k5  Clk4  Mapkapk2 | web link |
| chaperone binding | Gene Ontology | GO:0051087 | 5 (0.91%) | 103 (0.50%) | 0.13730511863 | 0.221584122019 | Cdc37l1  Fnip1  Ahsa2  Bin1  Tsc1 | web link |
| ligase activity | Gene Ontology | GO:0016874 | 6 (1.10%) | 132 (0.64%) | 0.137534972288 | 0.221584122019 | Gss  Mthfs  Rtca  Nadsyn1  Mars1  Aasdh | web link |
| PDZ domain binding | Gene Ontology | GO:0030165 | 5 (0.91%) | 111 (0.54%) | 0.170829856022 | 0.270221772254 | Baiap2  Ctbp1  Plekha1  Cask  Cadm1 | web link |
| kinase binding | Gene Ontology | GO:0019900 | 5 (0.91%) | 117 (0.57%) | 0.197842159258 | 0.30372228596 | Midn  Fas  Egfr  Bcar3  Wdcp | web link |
| double-stranded DNA binding | Gene Ontology | GO:0003690 | 6 (1.10%) | 149 (0.72%) | 0.202392413253 | 0.30372228596 | Nfib  Mterf3  Cggbp1  Hnrnpu  Rexo4  Tardbp | web link |
| protein tyrosine kinase activity | Gene Ontology | GO:0004713 | 5 (0.91%) | 118 (0.57%) | 0.202481523973 | 0.30372228596 | Clk4  Pkdcc  Egfr  Fgfr3  Map2k5 | web link |
| peptidase activity | Gene Ontology | GO:0008233 | 17 (3.11%) | 538 (2.60%) | 0.258942249755 | 0.381830097097 | Erap1  Josd2  Hmces  Desi1  Tysnd1  Pgpep1l  Masp1  Tmprss6  Rnpepl1  Atg4c  Adamts10  Habp2  Metap1d  F10  Sppl2b  Proc  Abhd5 | web link |
| monooxygenase activity | Gene Ontology | GO:0004497 | 5 (0.91%) | 136 (0.66%) | 0.291111121233 | 0.422111125789 | Cyp3a59  Cyp4a31  Cyp2r1  Cyp4b1  Cyp4f13 | web link |
| actin binding | Gene Ontology | GO:0003779 | 12 (2.19%) | 384 (1.86%) | 0.317903742288 | 0.453403698017 | Pdlim5  Washc1  Neb  Hnrnpu  Tpm1  Inf2  Coro1b  Mtss1  Nf2  Ablim2  Lcp1  Flnb | web link |
| transcription coactivator activity | Gene Ontology | GO:0003713 | 7 (1.28%) | 213 (1.03%) | 0.333219181538 | 0.464008063762 | Pmf1  Tdrd3  Hmga1  Apex1  Smarca4  Kdm5a  Mms19 | web link |
| serine-type peptidase activity | Gene Ontology | GO:0008236 | 6 (1.10%) | 179 (0.86%) | 0.336005839276 | 0.464008063762 | Tysnd1  Masp1  Tmprss6  Habp2  F10  Proc | web link |
| calmodulin binding | Gene Ontology | GO:0005516 | 6 (1.10%) | 182 (0.88%) | 0.350082924344 | 0.47589397528 | Fas  Egfr  Cask  Phkb  Ndufaf4  Mapkapk2 | web link |
| protein C-terminus binding | Gene Ontology | GO:0008022 | 7 (1.28%) | 227 (1.10%) | 0.393512004926 | 0.526700683516 | Ube2i  Taf13  Sdc1  Cask  Plekhb1  Mid1ip1  Pex16 | web link |
| GTPase activator activity | Gene Ontology | GO:0005096 | 7 (1.28%) | 235 (1.14%) | 0.428082373156 | 0.564290400978 | Arhgef12  Arap2  Tbc1d5  Rin2  Nf1  Rgs3  Asap3 | web link |
| serine-type endopeptidase activity | Gene Ontology | GO:0004252 | 6 (1.10%) | 202 (0.98%) | 0.444247676578 | 0.573869852415 | Tysnd1  Masp1  Tmprss6  Habp2  F10  Proc | web link |
| sequence-specific DNA binding | Gene Ontology | GO:0043565 | 13 (2.38%) | 462 (2.23%) | 0.448541953612 | 0.573869852415 | Hsf1  Foxp1  Foxk2  Esr1  Zbtb14  Safb  Hnrnpu  Zfp131  Hnf1b  Ezh2  Thra  Rsl1  Ncor2 | web link |
| DNA binding | Gene Ontology | GO:0003677 | 48 (8.78%) | 1779 (8.60%) | 0.462111630831 | 0.582662491048 | Hsf1  Meiob  Thap2  Deaf1  Hnrnpu  Thra  Apex1  Ezh2  Zfp638  Hmga1  Kdm5a  Zkscan1  Foxp1  Foxk2  Esr1  Irf9  Safb  0610010K14Rik  Mbd6  Dtnb  Tfdp1  Smarca4  Setdb1  Tardbp  Nfib  Nfia  Hmces  Zbtb14  Mterf3  Timeless  Hnf1b  Pcbp2  Gtf2i  Nabp2  Polr2h  Repin1  Taf13  Set  Hp1bp3  Zfp467  Cggbp1  Zfpm1  Zfp131  Phc2  Ssbp1  Nrf1  Ncor2  Ncor1 | web link |
| enzyme binding | Gene Ontology | GO:0019899 | 12 (2.19%) | 437 (2.11%) | 0.487498273531 | 0.605890711389 | Ube2i  Fnip1  Trio  Esr1  Egfr  Gstm4  Calu  Uhmk1  Pcbp2  Hmga1  Mms19  Luc7l2 | web link |
| GTPase activity | Gene Ontology | GO:0003924 | 8 (1.46%) | 298 (1.44%) | 0.532069727312 | 0.651972764452 | Nras  Septin4  Mtif2  Mmaa  Arhgap5  Kras  Irgm2  Cdc42 | web link |
| transferase activity, transferring glycosyl groups | Gene Ontology | GO:0016757 | 6 (1.10%) | 230 (1.11%) | 0.570060670596 | 0.688823310303 | Upp2  St3gal5  St3gal4  Parp6  Poglut1  Gba2 | web link |
| protein kinase binding | Gene Ontology | GO:0019901 | 13 (2.38%) | 523 (2.53%) | 0.628589335649 | 0.741111914058 | Bcl2l1  Dnaja3  Ghr  Esr1  Prlr  Egfr  Cep43  Cdk12  Slc12a7  Atg13  Irgm2  Hsf1  Cdc42 | web link |
| protein heterodimerization activity | Gene Ontology | GO:0046982 | 9 (1.65%) | 365 (1.76%) | 0.630371053337 | 0.741111914058 | Hsf1  Bcl2l1  Tcof1  Abcg8  Tpm1  Taf13  Gtf2a2  Krt10  Adra1a | web link |
| microtubule binding | Gene Ontology | GO:0008017 | 6 (1.10%) | 249 (1.20%) | 0.646932063541 | 0.750441193707 | Strbp  Gas2l1  Kif13a  Nf1  Nde1  Kifc2 | web link |
| DNA-binding transcription repressor activity, RNA polymerase II-specific | Gene Ontology | GO:0001227 | 8 (1.46%) | 336 (1.62%) | 0.666702937413 | 0.758730045737 | Hsf1  Foxp1  Foxk2  Zbtb14  Zfp973  Deaf1  Zfp131  2610044O15Rik8 | web link |
| lipid binding | Gene Ontology | GO:0008289 | 7 (1.28%) | 299 (1.44%) | 0.679411591081 | 0.758730045737 | Nme4  Esr1  Snx10  Plekha1  Nf1  Apol9b  Apoc3 | web link |
| ATPase activity | Gene Ontology | GO:0016887 | 8 (1.46%) | 343 (1.66%) | 0.688808028649 | 0.758730045737 | Abcg8  Ddx56  Atp5j  Tcp1  Atp9b  Abcc6  Smarca4  Psmc6 | web link |
| GTP binding | Gene Ontology | GO:0005525 | 9 (1.65%) | 384 (1.86%) | 0.68896176567 | 0.758730045737 | Nras  Fpgt  Septin4  Mtif2  Mmaa  Arhgap5  Cdc42  Irgm2  Kras | web link |
| DNA-binding transcription factor activity | Gene Ontology | GO:0003700 | 14 (2.56%) | 616 (2.98%) | 0.754839198744 | 0.820887628634 | Hsf1  Nfib  Nfia  Foxp1  Foxk2  2010315B03Rik  Esr1  Irf9  Zbtb14  Thra  Hnf1b  Gtf2i  Tfdp1  Nrf1 | web link |
| protein domain specific binding | Gene Ontology | GO:0019904 | 7 (1.28%) | 329 (1.59%) | 0.769764977797 | 0.826784605782 | Scaf1  Thra  Prkar1a  Nde1  Ctbp1  Tfdp1  Nf2 | web link |
| molecular\_function | Gene Ontology | GO:0003674 | 87 (15.90%) | 3542 (17.11%) | 0.792109131399 | 0.840408468679 | Coa8  9430038I01Rik  Tmbim4  D11Wsu47e  Fam149b  Cyren  Tmem258  Cnot10  Nup37  Rin2  Gucd1  Mtx3  2700062C07Rik  Bri3bp  Arl14ep  Bbip1  Kctd18  Fam47e  Wbp1l  Septin4  Spata2l  Arv1  Mettl9  Smco4  Smim10l1  Dram2  Ccdc97  Retreg1  Plekha6  Zfand4  1700066M21Rik  Xkr9  Fpgt  Proser2  Zswim8  Card19  Commd4  Mpv17l2  Cmbl  Ncln  Atp5mpl  Tmem219  Fam169b  Ubap2l  Wdr59  Snx29  Trappc13  Miip  Szrd1  Slc66a2  Haghl  Pigx  Tmem208  Eapp  Fam160a2  Cox7a2  Trappc8  Thap2  Tm9sf1  Phf20l1  Mtfr1l  Ccdc58  Higd1a  Lims2  Ndufaf4  Dcaf11  Yif1b  Ccdc159  Aasdh  Lysmd3  Gmppa  Dcun1d3  Rcbtb2  Susd1  Tmem222  Ociad2  Qser1  Fam214a  Fam214b  Sys1  Wdr27  Fance  Wdr20  Sec16b  Vwa3b  Etfrf1  Lrrc28 | web link |
| DNA-binding transcription factor activity, RNA polymerase II-specific | Gene Ontology | GO:0000981 | 24 (4.39%) | 1055 (5.10%) | 0.804148703215 | 0.842902857587 | Hsf1  Zbtb7c  Deaf1  Thra  Zkscan1  Zfp141  Rsl1  Tfdp1  Gm28557  Foxp1  Foxk2  Irf9  Zfp950  Zfp874a  Zfp995  Nrf1  Nfib  Nfia  Zfp932  Hnf1b  Zfp235  Gm14403  Zfp131  Zfp946 | web link |
| DNA-binding transcription activator activity, RNA polymerase II-specific | Gene Ontology | GO:0001228 | 10 (1.83%) | 491 (2.37%) | 0.839278190848 | 0.86925241195 | Nfib  Nfia  Foxk2  Zfp467  Esr1  Zfp131  Gtf2i  Zfp62  Tfdp1  Nrf1 | web link |
| signaling receptor binding | Gene Ontology | GO:0005102 | 6 (1.10%) | 364 (1.76%) | 0.921705847874 | 0.943393044294 | Trak1  Egfr  Angptl4  Clu  Cadm1  Mtss1 | web link |
| calcium ion binding | Gene Ontology | GO:0005509 | 13 (2.38%) | 709 (3.43%) | 0.938077010387 | 0.948984882601 | Adgre5  Enpp2  Ppp2r3a  Asph  Calu  Ppp2r3d  Masp1  Myl6  Habp2  Susd1  F10  Proc  Lcp1 | web link |
| sequence-specific double-stranded DNA binding | Gene Ontology | GO:1990837 | 7 (1.28%) | 517 (2.50%) | 0.98455542999 | 0.98455542999 | Hsf1  Nfib  Esr1  Zfp973  Irf9  Zbtb14  Hnrnpu | web link |
