# Supplementary material for: Regulation of Alternative Splicing of Lipid Metabolism Genes in Sepsis-Induced Liver Damage by RNA-Binding Proteins
Source: Inflammation. 2024 May 9;47(6):1952–68. doi: 10.1007/s10753-024-02017-2 (PMC11607010; doi:10.1007/s10753-024-02017-2)
Supplement: Supplementary file 1 — Supplementary file1 (ZIP 38655 KB) [file 10753_2024_2017_MOESM1_ESM.zip › Sup5_AS_GO.zip!/Sup5_AS_GO/ras/CLPDCA_vs_Sham/Ras_GO_analysis/Ras_GO_enrichment_P_top.pdf]

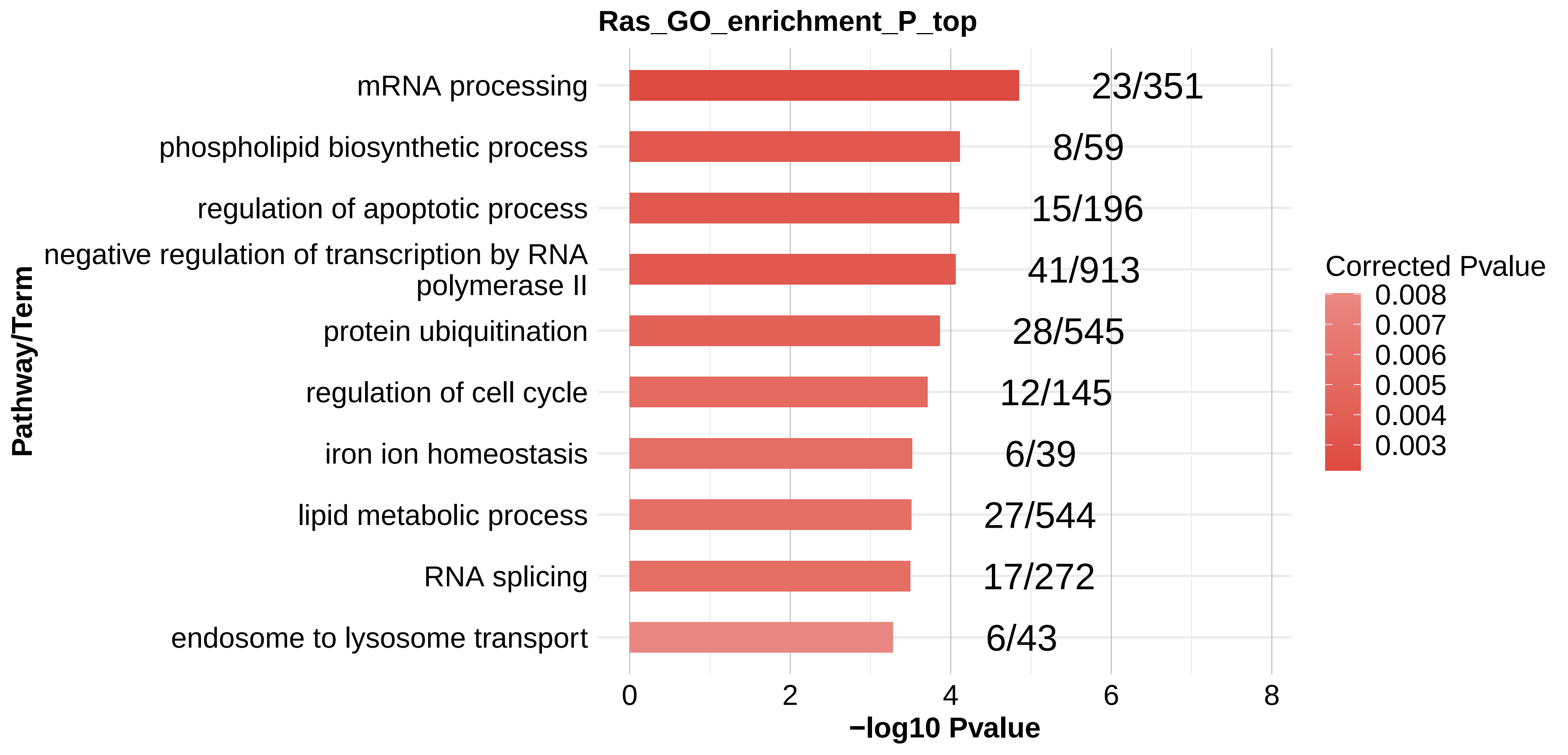

Ras_GO_enrichment_P_top
mRNA processing
phospholipid biosynthetic process
regulation of apoptotic process
negative regulation of transcription by RNA polymerase II
protein ubiquitination
regulation of cell cycle
iron ion homeostasis
lipid metabolic process
RNA splicing
endosome to lysosome transport
23/351
8/59
15/196
41/913
28/545
12/145
6/39
27/544
17/272
6/43
Pathway/Term
0
2
4
6
8
−log10 Pvalue
Corrected Pvalue
0.008
0.007
0.006
0.005
0.004
0.003
