# Supplementary material for: Regulation of Alternative Splicing of Lipid Metabolism Genes in Sepsis-Induced Liver Damage by RNA-Binding Proteins
Source: Inflammation. 2024 May 9;47(6):1952–68. doi: 10.1007/s10753-024-02017-2 (PMC11607010; doi:10.1007/s10753-024-02017-2)
Supplement: Supplementary file 1 — Supplementary file1 (ZIP 38655 KB) [file 10753_2024_2017_MOESM1_ESM.zip › Sup5_AS_GO.zip!/Sup5_AS_GO/ras/CLPDCA_vs_Sham/Ras_GO_analysis/Ras_GO_enrichment_F_top.pdf]

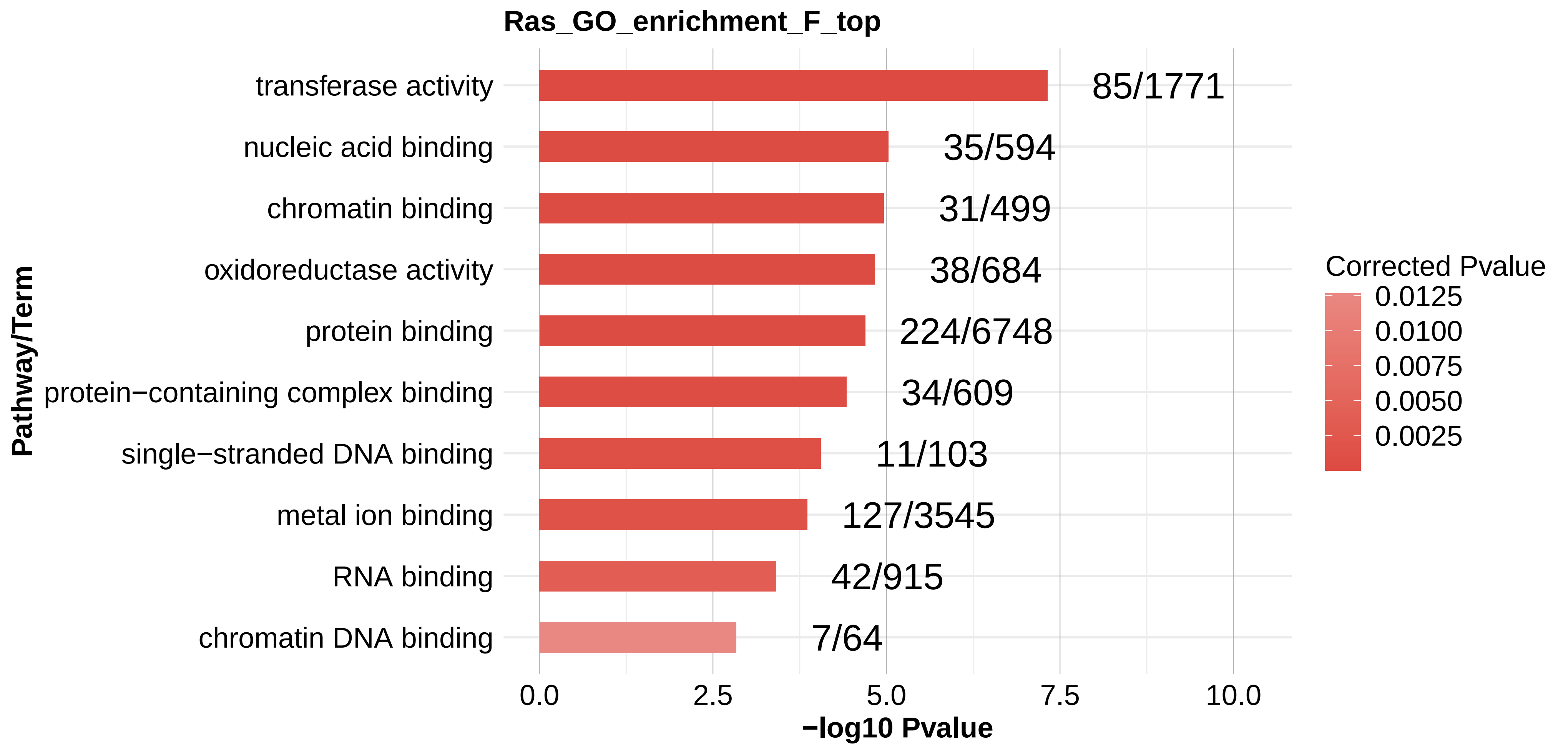

Ras_GO_enrichment_F_top
Pathway/Term
transferase activity
nucleic acid binding
chromatin binding
oxidoreductase activity
protein binding
protein−containing complex binding
single−stranded DNA binding
metal ion binding
RNA binding
chromatin DNA binding
85/1771
35/594
31/499
38/684
224/6748
34/609
11/103
127/3545
42/915
7/64
0.0
2.5
5.0
7.5
10.0
−log10 Pvalue
Corrected Pvalue
0.0125
0.0100
0.0075
0.0050
0.0025
