# Supplementary material for: Regulation of Alternative Splicing of Lipid Metabolism Genes in Sepsis-Induced Liver Damage by RNA-Binding Proteins
Source: Inflammation. 2024 May 9;47(6):1952–68. doi: 10.1007/s10753-024-02017-2 (PMC11607010; doi:10.1007/s10753-024-02017-2)
Supplement: Supplementary file 1 — Supplementary file1 (ZIP 38655 KB) [file 10753_2024_2017_MOESM1_ESM.zip › Sup5_AS_GO.zip!/Sup5_AS_GO/ras/CLPDCA_vs_Sham/Ras_GO_analysis/Ras_GO_enrichment_C_top.pdf]

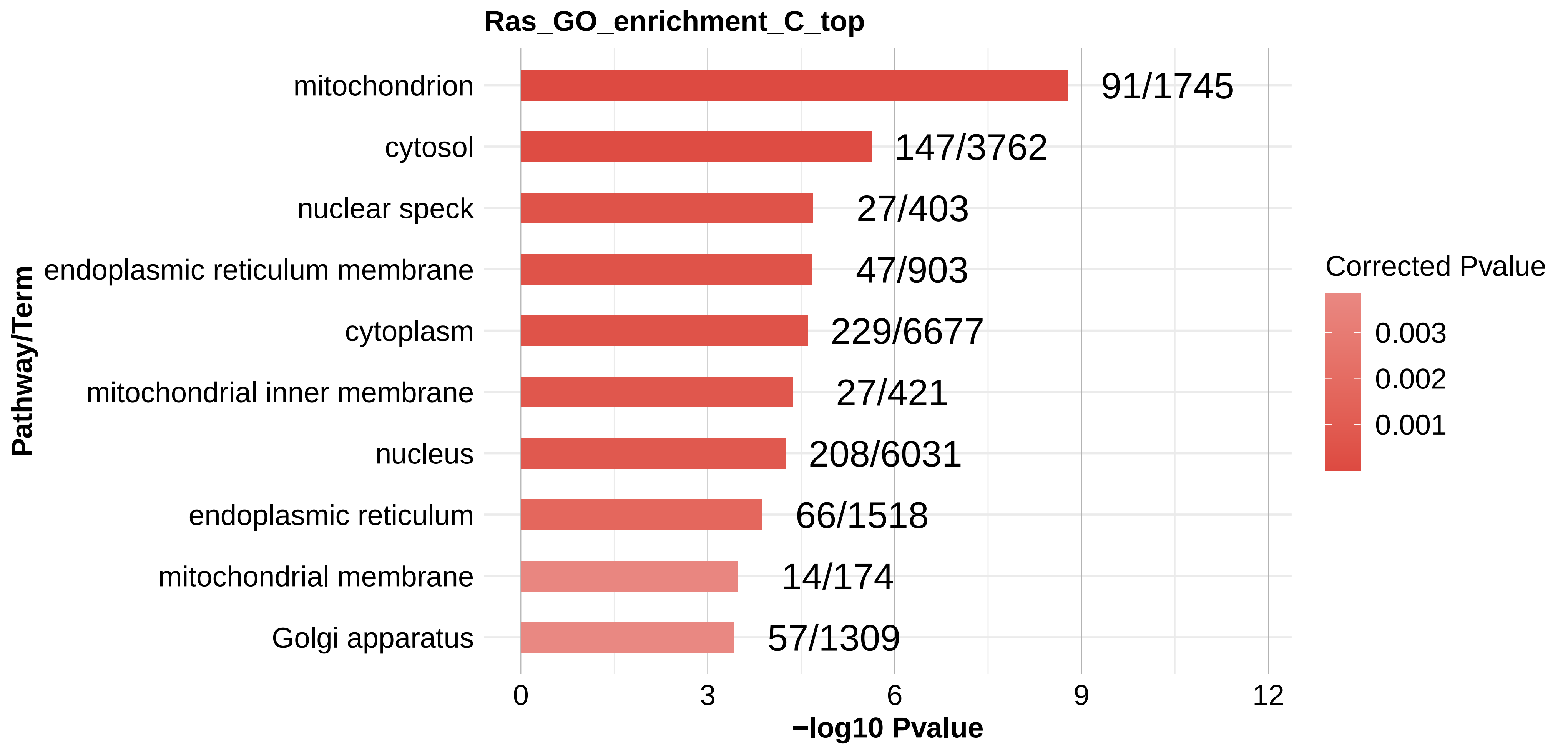
Ras_GO_enrichment_C_top
Pathway/Term
mitochondrion
cytosol
nuclear speck
endoplasmic reticulum membrane
cytoplasm
mitochondrial inner membrane
nucleus
endoplasmic reticulum
mitochondrial membrane
Golgi apparatus
91/1745
147/3762
27/403
47/903
229/6677
27/421
208/6031
66/1518
14/174
57/1309
0
3
6
9
12
−log10 Pvalue
Corrected Pvalue
0.003
0.002
0.001
