# Supplementary material for: Regulation of Alternative Splicing of Lipid Metabolism Genes in Sepsis-Induced Liver Damage by RNA-Binding Proteins
Source: Inflammation. 2024 May 9;47(6):1952–68. doi: 10.1007/s10753-024-02017-2 (PMC11607010; doi:10.1007/s10753-024-02017-2)
Supplement: Supplementary file 1 — Supplementary file1 (ZIP 38655 KB) [file 10753_2024_2017_MOESM1_ESM.zip › Sup5_AS_GO.zip!/Sup5_AS_GO/ras/di_RAS/GO_KEGG/CLP_vs_Sham_and_CLPDCA_vs_Sham_di_RAS/CLP_vs_Sham_diUp_nir_GO_analysis/CLP_vs_Sham_diUp_nir_GO_enrichment_web_C.html]

GO enrichment analyze


# GO enrichment analyze result


| Term | Database | ID | Input number (373) | Background number (20939) | P-Value | Corrected P-Value | Input | Hyperlink |
| --- | --- | --- | --- | --- | --- | --- | --- | --- |
| cytosol | Gene Ontology | GO:0005829 | 112 (30.03%) | 3762 (17.97%) | 7.10960712524e-09 | 5.19001320143e-07 | Apoo  Uox  Hnrnpk  Mon2  Cpeb3  Cnot2  Mapkapk5  Naa80  Vcpkmt  Cdv3  Sgk1  Usp40  Chmp3  Atg16l1  Atg16l2  Unc45a  Mpst  Mapk6  Abl1  Mapk9  Akr1c20  Upp2  Cep170  Dnajc5  Rpl35a  Helz  Farsa  Tnip1  Hnrnpl  Huwe1  Eef1d  Raph1  Uba5  Plekha1  Gm44805  Atat1  Yap1  Smg7  Dnajb6  Itgav  Mtmr2  Sec24c  Sec24b  Thada  Echdc1  Ubap1  Kynu  Ghr  Camsap2  Tnrc18  Tcf7l2  Txnl4a  Ppp4r3a  Zyx  Ywhaz  Sbds  Osbpl9  Ubxn2a  Alkbh3  Aox3  Pcm1  Acox1  Ces1f  Coro1b  Bdh2  Adk  Chchd1  Mlxipl  Golph3  Mri1  Tns3  Nr1h3  Rpl22l1  Bag6  Slc66a2  Btrc  Eif3e  Epb41  Ube2g2  Phldb2  Tbc1d17  Rabgap1  Flad1  Arrb2  Gmeb2  Nudcd2  Grhpr  Znrf1  Cuedc2  Fmo5  Abhd14b  Lcp1  Dusp3  Sh3d19  Rpl18  Mocs2  Mocs1  Atxn10  Josd2  Thoc7  Lyplal1  Rps10  Rps19  Eif5  Akt1s1  Uhrf1bp1l  Myo1c  Pnpla2  Pnpla6  Ppp6r1  Setd4  Ppp2r5c | web link |
| mitochondrion | Gene Ontology | GO:0005739 | 58 (15.55%) | 1745 (8.33%) | 2.76673118749e-06 | 0.000100985688343 | Tomm5  Mettl17  Apoo  Uox  Ghr  Abl1  Slc35b3  Ndufa5  Tomm22  Mrpl48  Foxk2  Sgk1  Pnkp  Ctsa  Golph3  Higd1a  Pnkd  Ndufs6  Atg4d  Mccc2  Ywhaz  Tmem11  Gm20498  Acacb  P4ha1  Tnrc18  Rdh13  Hax1  Polrmt  Mpst  Chchd1  Mrpl47  Gm49673  Mapk9  Etfbl  Gtpbp8  Dbi  Mrpl24  Nipsnap1  Dna2  Rab4b  Mrpl30  Mff  Maob  Acox1  Naxd  Dcakd  Rpl35a  Taz  Ggnbp1  Nudt13  Hnrnpk  Mtif3  Slc25a25  Rnaset2b  Cox7a2  Pnpla8  Alkbh3 | web link |
| endoplasmic reticulum | Gene Ontology | GO:0005783 | 51 (13.67%) | 1518 (7.25%) | 8.94183066591e-06 | 0.000217584546204 | Entpd5  Cers2  Ktn1  Fmo5  Tmem131l  Apoo  Ero1b  Calu  Ugt1a6b  Uba5  Cnpy2  Tmbim6  Rpl18  Ces1f  Pgap2  Tm7sf2  Sgk1  Pigp  BC031181  Srebf1  Yif1b  Cyp2r1  Thada  Pnpla6  Dhrs7b  Kctd17  Gba  H13  Hax1  Derl1  Abl1  Ugt1a7c  Insig2  Ube2g2  Manf  Gm49673  Rnaset2b  Hsd17b6  Dbi  Cyp2d10  Snx10  Rer1  Smpd4  Fkbp7  Atp11c  Lpcat3  Ubxn2a  Mcfd2  P4ha1  Pnpla8  Uggt1 | web link |
| nucleus | Gene Ontology | GO:0005634 | 143 (38.34%) | 6031 (28.80%) | 3.98617785238e-05 | 0.000727477458059 | Cers2  Hnrnpk  Hnrnpl  Edc4  Rbm3  Mapkapk5  Anapc5  Rfxap  Sgk1  Mier1  Mier2  Usp40  Inpp4a  Epc1  N4bp2l2  Rexo5  Gtf2a2  Smarca2  Clk4  Bcas3  Mindy3  Inca1  Unc45a  Ncapd3  Septin2  Zfp367  Med7  Cenpx  Abl1  Mapk9  Shld2  Zfp58  Snx10  Helz  Mettl23  Smpd4  Tnip1  Ccar1  C87436  Tial1  Nup98  Snrk  Uba5  Crem  Cnot2  Hdac10  Yap1  Smg7  Foxk2  Pcnp  4932438A13Rik  Safb  Dnajb6  Cpeb3  Mcrip2  Mapk15  Ndrg2  Dhrs7b  Ghr  Hax1  Tcf7l2  Tbx3  Pkp4  Txnl4a  Ppp4r3a  Psmc6  Luc7l2  Ywhaz  Dna2  Adk  Erbin  Parp3  Sbds  Ubxn2a  Cdk8  Nucb1  Alkbh3  Eya3  Rwdd3  Csnk1g3  Zbtb42  Zbtb43  Vegfa  Ptbp2  Mtmr2  Chchd1  Mlxipl  Huwe1  Arpc5  Ss18  Gzf1  Zyx  Mri1  Nr1h3  Dbi  Bag6  Btrc  Eif3e  Epb41  Sap130  Aen  Tbl1xr1  U2af1  Rbm39  Srebf1  Arrb2  Gmeb2  Lcorl  Plekha1  Hsf2  Cuedc2  Uckl1  Abhd14b  Esrp2  Tmem176b  Zmynd8  Dusp3  Banp  Pnkp  Kmt5b  Higd1a  Pnkd  Zfp874b  Nsd1  Thoc7  Dclre1c  Rps10  Rragc  Acacb  Nap1l4  Rassf5  Zfp938  Akt1s1  Zfp235  Chd2  Myo1c  Akap8l  Hnrnph3  Ppp6r1  Mapk6  Snrnp70  Setd4  Ppp2r5c | web link |
| cytoplasm | Gene Ontology | GO:0005737 | 153 (41.02%) | 6677 (31.89%) | 0.000111834995541 | 0.0016327909349 | Pcmtd2  Rbm3  Hnrnpk  Hnrnpl  Tial1  Nde1  Cnot2  Mapkapk5  Mcrip2  Anapc5  Naa80  Vcpkmt  Cdv3  Sgk1  Mier2  Inpp4a  Atg4d  Chmp3  Cyp2r1  Bcas3  Kctd17  Fgd6  Atg16l1  Atg16l2  Inca1  Unc45a  Mpst  Mapk6  Abl1  Mapk9  Upp2  Cyp2d10  Cep170  Snx10  Dnajc5  Mettl23  Mgat1  Farsa  Tnip1  Nudt14  Ccar1  Pfkfb2  Snrk  Uba5  Crem  Tmbim6  Hdac10  Cdc123  Rpl18  Yap1  Slmap  Smg7  Ghr  Dnajb6  Cpeb3  Adk  Mapk15  Khk  Ndrg2  Echdc1  Ubap1  Kynu  Gm20498  Camsap2  Rassf5  Tctn1  Hax1  Pkp4  Txnl4a  Ppp4r3a  Psmc6  Dbi  Dna2  Erbin  Parp3  Sbds  Nucb1  Alkbh3  Eya3  Aox3  Rwdd3  Pcm1  Acox1  Ces1f  Coro1b  Csnk1g3  Zbtb42  Hsf2  Vegfa  Bdh2  Mtmr2  Mlxipl  Huwe1  Arpc5  Mpdz  Gzf1  Zyx  Mri1  Nr1h3  Ywhaz  Zmynd8  Btrc  Eif3e  Epb41  Phldb2  Zfyve19  Rabgap1  Flad1  Serpinb6a  Ggnbp1  Srebf1  Arrb2  Fbxo6  Gmeb2  Plekha1  Nudcd2  Grhpr  Pard3  Cuedc2  Uckl1  Tmem131l  Arhgap12  Bag6  Kctd20  Nedd4l  Lcp1  Map4k2  Pnpla2  Sh3d19  Atat1  Mocs2  Pnkd  Atxn10  Josd2  Thoc7  Rps10  Rragc  Nap1l4  Rps19  Gmppa  Eif5  Cdc42ep4  Akt1s1  Uhrf1bp1l  Foxk2  Myo1c  Anapc10  Akap8l  Albfm1  Septin2  Ppp6r1  Snrnp70  Setd4 | web link |
| nucleoplasm | Gene Ontology | GO:0005654 | 81 (21.72%) | 3156 (15.07%) | 0.000353233703336 | 0.00429767672392 | C87436  Hsf2  Rbm3  Cuedc2  Nup98  Higd1a  Eef1d  Inpp4a  Abhd14b  Esrp2  Zbtb42  Hnrnpk  Hnrnpl  Plekha1  Edc4  Hdac10  Cnot2  Mapkapk5  Parp3  Adk  Dusp3  Chchd1  Banp  Pnpla2  Mlxipl  Mier1  Mier2  Huwe1  Safb  Kmt5b  Cpeb3  Epc1  Mocs2  Dcaf11  Tnip1  Gzf1  Mindy3  Atg4d  Tnrc18  Smarca2  Luc7l2  Rbm39  Dclre1c  Nr1h3  Rps19  Bag6  Gtf2a2  Rragc  Zmynd8  Mri1  Atg16l2  Inca1  Tcf7l2  Ncapd3  Gmeb2  Septin2  Dna2  Zfp367  Med7  Abl1  Txnl4a  Pfkfb2  Ppp4r3a  Aen  Foxk2  Sh3d19  Tbl1xr1  Pcnp  Pnkp  Myo1c  Dnajb6  Mettl17  Sbds  Srebf1  Arrb2  Hnrnph3  Cdk8  Snrnp70  Alkbh3  Eya3  Ppp2r5c | web link |
| endoplasmic reticulum lumen | Gene Ontology | GO:0005788 | 8 (2.14%) | 115 (0.55%) | 0.0010629895452 | 0.0103491283779 | Entpd5  Calu  Fkbp7  Manf  Gm49673  P4ha1  Rnaset2b  Uggt1 | web link |
| nuclear membrane | Gene Ontology | GO:0031965 | 12 (3.22%) | 236 (1.13%) | 0.00113415105512 | 0.0103491283779 | Cuedc2  Nup98  Pcm1  Tnrc18  Tmem176b  Hax1  Inpp4a  Epc1  Mindy3  Erbin  Txnl4a  Aen | web link |
| cell leading edge | Gene Ontology | GO:0031252 | 6 (1.61%) | 69 (0.33%) | 0.00143077106371 | 0.0116051430724 | Ywhaz  Coro1b  Raph1  Abl1  Phldb2  Bcas3 | web link |
| ribosome | Gene Ontology | GO:0005840 | 10 (2.68%) | 196 (0.94%) | 0.00279697207739 | 0.0201521787866 | Rpl22l1  Mrpl24  Chchd1  Mrpl30  Rps19  Rpl35a  Rps10  Mrpl47  Mrpl48  Rpl18 | web link |
| endoplasmic reticulum-Golgi intermediate compartment | Gene Ontology | GO:0005793 | 6 (1.61%) | 81 (0.39%) | 0.00324252397588 | 0.0201521787866 | Fn1  Yif1b  Mcfd2  Rer1  Nucb1  Uggt1 | web link |
| endoplasmic reticulum membrane | Gene Ontology | GO:0005789 | 28 (7.51%) | 903 (4.31%) | 0.00331268692382 | 0.0201521787866 | Cers2  Fmo5  Apoo  Ero1b  Calu  Derl1  Uba5  Tmbim6  Pgap2  Tm7sf2  Sgk1  Yif1b  Sec24c  Sec24b  Cyp2r1  Lpcat3  Dhrs7b  Cyp3a59  H13  Ugt1a7c  Insig2  Gm4951  Cyp2d10  Smpd4  Srebf1  Atp11c  Pnpla6  Pnpla8 | web link |
| mitochondrial inner membrane | Gene Ontology | GO:0005743 | 16 (4.29%) | 421 (2.01%) | 0.00384491172661 | 0.0215906581571 | Nipsnap1  Tmem11  Tmem256  Gm20498  Apoo  Rdh13  Higd1a  Taz  Maob  Mpst  Ugt1a7c  Ndufs6  Tomm22  Ndufa5  Cox7a2  Slc25a25 | web link |
| endosome | Gene Ontology | GO:0005768 | 21 (5.63%) | 662 (3.16%) | 0.00809716780947 | 0.0422209464351 | Pip4p2  Ubap1  Dennd10  Rab4b  Slc66a2  Snx10  Arrb2  Znrf1  Golph3  Uhrf1bp1l  2610528J11Rik  Arpc5  Nedd4l  Osbpl9  Atp11c  Lamtor2  Nipa2  Chmp3  Mon2  Mtmr2  Hsd17b6 | web link |
| intracellular membrane-bounded organelle | Gene Ontology | GO:0043231 | 30 (8.04%) | 1061 (5.07%) | 0.00872066998563 | 0.0424405939301 | Fmo5  Calu  Ugt1a6b  Uba5  Ugt2b36  Mtmr2  Ces1f  Tm7sf2  Ctsa  Golph3  Nup98  Epc1  Srebf1  Smarca2  Cyp2r1  Ubap1  Bag6  Rragc  Cyp3a59  Hax1  Osbpl9  Ugt1a7c  Hsd17b2  Hsd17b6  Foxk2  Cyp2d10  Dnajc5  P4ha1  Rhobtb1  Pnpla8 | web link |
| spindle | Gene Ontology | GO:0005819 | 9 (2.41%) | 203 (0.97%) | 0.0108340854478 | 0.0468563651105 | Pard3  Cep170  Sbds  Atat1  Mapk15  Septin2  Pkp4  Nde1  Anapc5 | web link |
| basolateral plasma membrane | Gene Ontology | GO:0016323 | 10 (2.68%) | 239 (1.14%) | 0.0109117562586 | 0.0468563651105 | Map4k2  Slc2a9  Gm20498  Erbin  Epb41  Slc39a8  Slco1b2  Arrb2  Slc4a2  Slc38a3 | web link |
| mitotic spindle | Gene Ontology | GO:0072686 | 6 (1.61%) | 109 (0.52%) | 0.0134032680655 | 0.0543576982656 | Nudcd2  Tbl1xr1  Cep170  Epb41  Pkp4  Atat1 | web link |
| actin cytoskeleton | Gene Ontology | GO:0015629 | 9 (2.41%) | 223 (1.06%) | 0.018917879484 | 0.0726844843333 | Myo1c  Hax1  Arpc5  Epb41  Septin2  Abl1  Lcp1  Cdc42ep4  Zyx | web link |
| trans-Golgi network | Gene Ontology | GO:0005802 | 8 (2.14%) | 200 (0.96%) | 0.0273195771669 | 0.0956205711178 | Gba  Slc66a2  Smpd4  Golph3  Golga1  Atp11c  Relch  Nucb1 | web link |
| mitochondrial intermembrane space | Gene Ontology | GO:0005758 | 5 (1.34%) | 95 (0.45%) | 0.0275072875818 | 0.0956205711178 | Gm49673  Golph3  Rnaset2b  Hax1  Ggnbp1 | web link |
| nuclear speck | Gene Ontology | GO:0016607 | 13 (3.49%) | 403 (1.92%) | 0.0293867004487 | 0.0975104151252 | Sgk1  Akap8l  U2af1  Thoc7  Unc45a  Mocs2  Sap130  Erbin  Snrnp70  Rbm39  Ppp4r3a  Rfxap  Luc7l2 | web link |
| protein-containing complex | Gene Ontology | GO:0032991 | 23 (6.17%) | 869 (4.15%) | 0.0388258921043 | 0.118741166882 | Ndufa5  Abl1  Pcm1  Neb  Ugt1a6b  Pard3  Mapkapk5  Akt1s1  Uggt1  Vcpkmt  Mier1  Mier2  Higd1a  Rbm39  Epb41  Ugt1a7c  Mapk6  Polrmt  Ywhaz  Mff  Mettl23  Srebf1  Cdk8 | web link |
| mitochondrial outer membrane | Gene Ontology | GO:0005741 | 7 (1.88%) | 177 (0.85%) | 0.0396438023936 | 0.118741166882 | Tomm5  Mff  Gm20498  Hax1  Taz  Maob  Tomm22 | web link |
| spliceosomal complex | Gene Ontology | GO:0005681 | 7 (1.88%) | 178 (0.85%) | 0.0406647831788 | 0.118741166882 | Rbm3  U2af1  Hnrnpk  Ppil3  Txnl4a  Ptbp2  Snrnp70 | web link |
| perinuclear region of cytoplasm | Gene Ontology | GO:0048471 | 20 (5.36%) | 755 (3.61%) | 0.051242564311 | 0.143873353642 | Ywhaz  Rab4b  Sgk1  Gm20498  Golga1  Abl1  Unc45a  Enpp3  Dnajb6  Coro1b  Mgat1  Septin2  Pkp4  Hnrnpl  Manf  Ndrg2  Atxn10  Ccar1  Lcp1  Mtmr2 | web link |
| nuclear body | Gene Ontology | GO:0016604 | 10 (2.68%) | 318 (1.52%) | 0.0596094021877 | 0.161166161471 | Inca1  Banp  Pcnp  Nup98  Myo1c  Epb41  Raph1  Med7  Abl1  Parp3 | web link |
| Golgi apparatus | Gene Ontology | GO:0005794 | 31 (8.31%) | 1309 (6.25%) | 0.0650050466366 | 0.169477443017 | Apoo  Calu  Osbpl9  Slc35b3  Uba5  Naa60  Nedd4l  Atat1  Pgap2  Smpd4  Golph3  Golga1  Yif1b  Ndrg2  Ggnbp1  Nagpa  Zmynd8  Gba  Camsap2  Unc45a  Map4k2  Rer1  Dbi  Mapk15  Dclre1c  Mgat1  Srebf1  Dnajc5  Mcfd2  Nucb1  Ppp2r5c | web link |
| ribonucleoprotein complex | Gene Ontology | GO:1990904 | 6 (1.61%) | 162 (0.77%) | 0.0703139709459 | 0.173071921311 | Tial1  Nup98  Esrp2  Akap8l  Hnrnpl  Hnrnph3 | web link |
| kinetochore | Gene Ontology | GO:0000776 | 5 (1.34%) | 124 (0.59%) | 0.0711254471143 | 0.173071921311 | Nde1  Nudcd2  Septin2  Nup98  Cenpx | web link |
| midbody | Gene Ontology | GO:0030496 | 6 (1.61%) | 166 (0.79%) | 0.0770552890191 | 0.181452777368 | Zfyve19  Safb  Cpeb3  Septin2  Pkp4  Chmp3 | web link |
| transcription regulator complex | Gene Ontology | GO:0005667 | 8 (2.14%) | 257 (1.23%) | 0.0900004099498 | 0.202615695836 | Dhrs7b  Mlxipl  Hax1  Tcf7l2  Crem  Yap1  Eya3  Med7 | web link |
| mitochondrial membrane | Gene Ontology | GO:0031966 | 6 (1.61%) | 174 (0.83%) | 0.0915933967477 | 0.202615695836 | Mff  Gm20498  Higd1a  Taz  Tmbim6  Pnpla8 | web link |
| peroxisome | Gene Ontology | GO:0005777 | 5 (1.34%) | 136 (0.65%) | 0.0962613794676 | 0.203154700928 | Uox  Dhrs7b  Acox1  Mff  Pnpla8 | web link |
| cell cortex | Gene Ontology | GO:0005938 | 6 (1.61%) | 177 (0.85%) | 0.0974029388011 | 0.203154700928 | Pard3  Hax1  Epb41  Septin2  Hnrnpk  Rhobtb1 | web link |
| focal adhesion | Gene Ontology | GO:0005925 | 6 (1.61%) | 184 (0.88%) | 0.111699217183 | 0.217708938665 | Itgav  Tns3  Phldb2  Atat1  Lcp1  Zyx | web link |
| chromosome, centromeric region | Gene Ontology | GO:0000775 | 5 (1.34%) | 143 (0.68%) | 0.112744985408 | 0.217708938665 | Nudcd2  Nde1  Septin2  Ppp2r5c  Cenpx | web link |
| cytoskeleton | Gene Ontology | GO:0005856 | 29 (7.77%) | 1285 (6.14%) | 0.113327940675 | 0.217708938665 | Nudcd2  Pard3  Pcm1  Coro1b  Nde1  Lcp1  Anapc5  Sbds  Atat1  Arpc5  Mapk15  Bcas3  Fgd6  Camsap2  Tctn1  Rassf5  Cdc42ep4  Septin2  Pkp4  Abl1  Epb41  Ppp4r3a  Zyx  Zfyve19  Cep170  Snx10  Rabgap1  Slmap  Rhobtb1 | web link |
| lysosome | Gene Ontology | GO:0005764 | 11 (2.95%) | 411 (1.96%) | 0.118796953764 | 0.222363528839 | Acp5  Znrf1  Rragc  Gba  Ctbs  Pip4p2  Slc39a8  Ctsa  Gm49673  Rnaset2b  Lamtor2 | web link |
| microtubule cytoskeleton | Gene Ontology | GO:0015630 | 6 (1.61%) | 190 (0.91%) | 0.124755149284 | 0.225996223567 | Nudcd2  Camsap2  Cdc42ep4  Ss18  Septin2  Rbm39 | web link |
| late endosome | Gene Ontology | GO:0005770 | 5 (1.34%) | 149 (0.71%) | 0.12787934123 | 0.225996223567 | Chmp3  Derl1  Lipc  Dennd10  Lamtor2 | web link |
| postsynaptic density | Gene Ontology | GO:0014069 | 9 (2.41%) | 327 (1.56%) | 0.131655181729 | 0.225996223567 | Ywhaz  Rps19  Mtmr2  Cpeb3  Epb41  Hnrnpk  Arrb2  Pkp4  Mpdz | web link |
| adherens junction | Gene Ontology | GO:0005912 | 5 (1.34%) | 151 (0.72%) | 0.133121063197 | 0.225996223567 | Pkp4  Pard3  Vegfa  Zyx  Cdc42ep4 | web link |
| receptor complex | Gene Ontology | GO:0043235 | 6 (1.61%) | 200 (0.96%) | 0.148075868276 | 0.245671326913 | Tm7sf2  Ghr  Prlr  Lifr  Gprc5c  Nr1h3 | web link |
| synaptic vesicle | Gene Ontology | GO:0008021 | 5 (1.34%) | 167 (0.80%) | 0.17828760757 | 0.2864365592 | Dbi  Dnajc5  Septin2  Mtmr2  Mff | web link |
| microtubule organizing center | Gene Ontology | GO:0005815 | 9 (2.41%) | 353 (1.69%) | 0.180494270181 | 0.2864365592 | Nudcd2  Zfyve19  Cep170  Snx10  Pcm1  Slmap  Rabgap1  Nde1  Ppp4r3a | web link |
| early endosome | Gene Ontology | GO:0005769 | 7 (1.88%) | 274 (1.31%) | 0.218101782277 | 0.338753832048 | Lipc  Nipa2  2610528J11Rik  Derl1  Uhrf1bp1l  Chmp3  Nucb1 | web link |
| apical plasma membrane | Gene Ontology | GO:0016324 | 9 (2.41%) | 383 (1.83%) | 0.245326849118 | 0.370951846278 | Pard3  Slc2a9  Slc38a3  Hax1  Enpp3  Fn1  Slc39a8  Slc4a2  Mpdz | web link |
| Golgi membrane | Gene Ontology | GO:0000139 | 11 (2.95%) | 485 (2.32%) | 0.248995074899 | 0.370951846278 | Map4k2  Smpd4  Apoo  Huwe1  Mgat1  Slc35b3  Pgap2  Rer1  Naa60  Yif1b  Srebf1 | web link |
| cell junction | Gene Ontology | GO:0030054 | 18 (4.83%) | 883 (4.22%) | 0.311971172344 | 0.455477911622 | Pkp4  Pard3  Znrf1  Gm20498  Mff  Erbin  Itgav  Epb41  Mapk15  Mpst  Hnrnpk  Cpeb3  Gtf2a2  Tns3  Lcp1  Atat1  Mpdz  Zyx | web link |
| cytoplasmic vesicle | Gene Ontology | GO:0031410 | 16 (4.29%) | 789 (3.77%) | 0.333358171584 | 0.477159735797 | Znrf1  Mff  Dnajc5  Hax1  Rhobtb1  Myo1c  Pip4p2  Tbc1d17  Mapk15  Srebf1  Arrb2  Sec24c  Sec24b  Chmp3  Mpdz  Golga1 | web link |
| lysosomal membrane | Gene Ontology | GO:0005765 | 5 (1.34%) | 217 (1.04%) | 0.344518324166 | 0.483650724311 | Slc39a8  Pip4p2  Ctsa  Lamtor2  Gba | web link |
| mitochondrial matrix | Gene Ontology | GO:0005759 | 5 (1.34%) | 224 (1.07%) | 0.3692061123 | 0.508529173545 | Polrmt  Atg4d  Etfbl  Mccc2  Mettl17 | web link |
| chromatin | Gene Ontology | GO:0000785 | 6 (1.61%) | 333 (1.59%) | 0.545891405482 | 0.737964307411 | Nap1l4  Tcf7l2  Akap8l  Hnrnpk  Smarca2  Nr1h3 | web link |
| neuron projection | Gene Ontology | GO:0043005 | 9 (2.41%) | 512 (2.45%) | 0.563228464857 | 0.747557780628 | Sgk1  Ralbp1  Arpc5  Gm20498  Mpst  Cpeb3  Abl1  Mapk9  Mpdz | web link |
| cell surface | Gene Ontology | GO:0009986 | 11 (2.95%) | 655 (3.13%) | 0.621607679703 | 0.810310011041 | Smim1  Ghr  Gm20498  Prlr  Cd47  H13  Lipc  Itgav  Septin2  Vegfa  Rer1 | web link |
| nucleolus | Gene Ontology | GO:0005730 | 13 (3.49%) | 813 (3.88%) | 0.693856432636 | 0.878003732537 | Rps10  Pnkp  Sbds  Rps19  Myo1c  Abhd14b  Cdk8  Rexo5  Abl1  Gzf1  Parp3  Rpl18  Aen | web link |
| dendrite | Gene Ontology | GO:0030425 | 9 (2.41%) | 577 (2.76%) | 0.703204307646 | 0.878003732537 | Rbm3  Clcn2  Nap1l4  Cpeb3  Mtmr2  Abl1  Atxn10  Tmem185a  Mpdz | web link |
| cell projection | Gene Ontology | GO:0042995 | 17 (4.56%) | 1062 (5.07%) | 0.709619455064 | 0.878003732537 | Rbm3  Camsap2  Pcm1  Tctn1  Myo1c  Lcp1  Mpdz  Mapk15  Septin2  Hnrnpk  Mtmr2  Arpc5  Rhobtb1  Ndrg2  Atat1  Tmem185a  Cpeb3 | web link |
| microtubule | Gene Ontology | GO:0005874 | 5 (1.34%) | 342 (1.63%) | 0.731771062051 | 0.890321458829 | Nde1  Camsap2  Atat1  Rassf5  Cep170 | web link |
| chromosome | Gene Ontology | GO:0005694 | 8 (2.14%) | 557 (2.66%) | 0.778729461061 | 0.931922141926 | Nudcd2  Kmt5b  Nsd1  Septin2  Cenpx  Nde1  Shld2  Ppp2r5c | web link |
| centrosome | Gene Ontology | GO:0005813 | 7 (1.88%) | 522 (2.49%) | 0.824923926905 | 0.956968102431 | Zfyve19  Cep170  Snx10  Camsap2  Pcm1  Nde1  Eya3 | web link |
| extracellular region | Gene Ontology | GO:0005576 | 28 (7.51%) | 1829 (8.73%) | 0.82587658155 | 0.956968102431 | Entpd5  Fn1  Apoo  Calu  Tfpi2  Vegfa  Igfbp4  Igfbp5  Gm44805  Mup2  Cnmd  Lifr  Il18bp  Bag6  Ghr  Tctn1  Manf  Gm49673  Rnaset2b  Lipc  Cp  Serpina3m  Enpp3  Albfm1  Mcfd2  Smoc1  Wfdc2  Nucb1 | web link |
| membrane | Gene Ontology | GO:0016020 | 146 (39.14%) | 8771 (41.89%) | 0.872559878417 | 0.99526361132 | Smim1  Cers2  Ktn1  Apoo  Adck5  Ugt1a6b  Ndufa5  Mon2  Slc66a2  Tomm5  Tmbim6  Tm7sf2  Sgk1  Tmem256  Inpp4a  Pigp  Slc39a9  Chmp3  Cyp2r1  Ggnbp1  Slc2a9  Atg16l1  Unc45a  Cyp2d10  Septin2  Wbp1l  Uba5  Gm49673  Hsd17b2  Mrpl48  Hsd17b6  Nipsnap1  Snx10  Dnajc5  Enpp3  Taz  Slmap  Mgat1  Atp11c  Lpcat3  Igsf5  Ralbp1  Osbpl9  Slc35b3  Slco1b2  Plekha1  Cnot2  Atat1  Gprc5c  Yap1  Gramd3  Gm20498  Smpd4  Cnmd  4932438A13Rik  Itgav  Golga1  Agap3  Lifr  Sec24c  Sec24b  Fndc3b  Slc43a1  Dhrs7b  H13  Ghr  Mettl23  Tomm22  Hax1  Ugt1a7c  Tmem185a  Mpdz  Rab4b  Mff  Erbin  Pkp4  Slc25a25  Nucb1  Maob  Entpd5  Aox3  Tctn1  2610528J11Rik  Derl1  Vegfa  Ugt2b36  Mtmr2  Slc39a8  Golph3  Slc41a2  Bag6  Rdh13  Gba  Pip4p2  Epb41  Ggt6  Ube2g2  Mia2  P2ry2  Phldb2  Rnaset2b  Lamtor2  Sema4g  Gm4951  Clcn2  Naxd  Srebf1  Arrb2  Slc4a2  Cox7a2  Cp  Pard3  Znrf1  Fmo5  Tmem131l  Tmem176b  Ero1b  Calu  Tmem11  Naa60  Tmem234  Lcp1  Nrp2  Plxnb2  Slc38a4  Map4k2  Banp  Slc38a3  Nipa2  Higd1a  Pnkd  Yif1b  Ndufs6  Cyp3a59  Josd2  Nagpa  Prlr  Cdc42ep4  Pgap2  Insig2  Cd47  Myo1c  Pnpla2  Pnpla6  Rer1  Pnpla8 | web link |
| synapse | Gene Ontology | GO:0045202 | 10 (2.68%) | 814 (3.89%) | 0.918384650535 | 0.999939901937 | Znrf1  Mff  Rps19  Mpst  Cpeb3  Abl1  Septin2  Nde1  Rpl18  Mpdz | web link |
| extracellular space | Gene Ontology | GO:0005615 | 24 (6.43%) | 1732 (8.27%) | 0.922640579849 | 0.999939901937 | Fn1  Tfpi2  Vegfa  Igfbp4  Igfbp5  Gm44805  Ces1f  Il18bp  Ghr  Gba  Tctn1  Manf  Gm49673  Rnaset2b  Sema4g  Dbi  Lipc  Wfdc2  Serpina3m  Serpinb6a  Slmap  Albfm1  Cp  Nucb1 | web link |
| glutamatergic synapse | Gene Ontology | GO:0098978 | 5 (1.34%) | 473 (2.26%) | 0.926386923494 | 0.999939901937 | Ywhaz  Erbin  Ndrg2  Hnrnpk  Nrp2 | web link |
| neuronal cell body | Gene Ontology | GO:0043025 | 6 (1.61%) | 581 (2.77%) | 0.94886600989 | 0.999939901937 | Pard3  Ghr  Nap1l4  Atxn10  Abl1  Ptbp2 | web link |
| external side of plasma membrane | Gene Ontology | GO:0009897 | 5 (1.34%) | 524 (2.50%) | 0.958477851354 | 0.999939901937 | Prlr  Enpp3  Lifr  Itgav  Ghr | web link |
| integral component of membrane | Gene Ontology | GO:0016021 | 99 (26.54%) | 6684 (31.92%) | 0.990273234087 | 0.999939901937 | Smim1  Cers2  Ktn1  Apoo  Adck5  Maob  Tomm5  Mon2  Tmbim6  Tm7sf2  Tmem256  Inpp4a  Pigp  Slc39a9  Cyp2r1  Slc2a9  Cyp3a59  Unc45a  Wbp1l  Rer1  Gm49673  Hsd17b2  Mrpl48  Cyp2d10  Ugt1a6b  Igsf5  Enpp3  Taz  Slmap  Smpd4  Atp11c  Lpcat3  Slc35b3  Slco1b2  Cnot2  Gprc5c  Mettl23  Gm20498  Mgat1  Cnmd  4932438A13Rik  Itgav  Lifr  Fndc3b  Slc43a1  Dhrs7b  H13  Ghr  Tomm22  Ugt1a7c  Tmem185a  Mff  Slc25a25  Entpd5  Aox3  2610528J11Rik  Derl1  Ugt2b36  Slc39a8  Slc41a2  Slc66a2  Gramd3  Epb41  Ggt6  Ube2g2  Mia2  P2ry2  Rnaset2b  Sema4g  Clcn2  Naxd  Srebf1  Slc4a2  Cox7a2  Cp  Fmo5  Tmem131l  Tmem11  Tmem176b  Tmem234  Nrp2  Plxnb2  Slc38a4  Map4k2  Banp  Slc38a3  Nipa2  Higd1a  Yif1b  Pip4p2  Josd2  Nagpa  Prlr  Pgap2  Insig2  Cd47  Pnpla2  Pnpla6  Pnpla8 | web link |
| cellular\_component | Gene Ontology | GO:0005575 | 26 (6.97%) | 2346 (11.20%) | 0.997875054386 | 0.999939901937 | Gpank1  Ccdc163  Polr1has  Haghl  Tmem234  1110065P20Rik  Fam204a  Ccdc30  Iqcc  Slc39a9  Prxl2c  Prrc2b  Atxn10  Rnf214  Josd2  Gm826  Aspdh  Cyp3a59  Gmppa  Wbp1l  Manf  Msantd2  Rsrp1  Zc3h7a  Wsb1  Wdr20 | web link |
| integral component of plasma membrane | Gene Ontology | GO:0005887 | 10 (2.68%) | 1203 (5.75%) | 0.998417867245 | 0.999939901937 | Plxnb2  Slc38a4  Slc38a3  Ghr  Clcn2  Cd47  Slc39a8  Slco1b2  Atp11c  Sema4g | web link |
| plasma membrane | Gene Ontology | GO:0005886 | 68 (18.23%) | 5547 (26.49%) | 0.999939901937 | 0.999939901937 | Smim1  Pard3  Ralbp1  Tmem131l  2610528J11Rik  Itgav  Coro1b  Raph1  Zbtb42  Slco1b2  Vegfa  Plekha1  Cnot2  Lcp1  Nedd4l  Adk  Gprc5c  Plxnb2  Slc38a4  Map4k2  Cdv3  Slc2a9  Slc38a3  Smpd4  Sh3d19  Nipa2  Golph3  Cpeb3  Slc39a8  Lifr  Atxn10  Arrb2  Ggnbp1  Ubap1  Slc41a2  Ghr  Nrp2  Pip4p2  H13  Hax1  Eif5  Epb41  Chmp3  Septin2  Pkp4  Rer1  P2ry2  Phldb2  Cdc42ep4  Mpdz  Zyx  Sema4g  Dbi  Rab4b  Sgk1  Csnk1g3  Dnajc5  Cd47  Flad1  Myo1c  Enpp3  Clcn2  Slmap  Pnpla2  Atp11c  Rhobtb1  Erbin  Cp | web link |
