# Supplementary material for: Regulation of Alternative Splicing of Lipid Metabolism Genes in Sepsis-Induced Liver Damage by RNA-Binding Proteins
Source: Inflammation. 2024 May 9;47(6):1952–68. doi: 10.1007/s10753-024-02017-2 (PMC11607010; doi:10.1007/s10753-024-02017-2)
Supplement: Supplementary file 1 — Supplementary file1 (ZIP 38655 KB) [file 10753_2024_2017_MOESM1_ESM.zip › Sup5_AS_GO.zip!/Sup5_AS_GO/ras/di_RAS/GO_KEGG/CLP_vs_Sham_and_CLPDCA_vs_Sham_di_RAS/CLP_vs_Sham_diUp_nir_GO_analysis/CLP_vs_Sham_diUp_nir_GO_enrichment_web_P.html]

GO enrichment analyze


# GO enrichment analyze result


| Term | Database | ID | Input number (304) | Background number (20961) | P-Value | Corrected P-Value | Input | Hyperlink |
| --- | --- | --- | --- | --- | --- | --- | --- | --- |
| proteasome-mediated ubiquitin-dependent protein catabolic process | Gene Ontology | GO:0043161 | 13 (4.28%) | 178 (0.85%) | 2.11723568089e-06 | 0.000184199504237 | Btrc  Kctd17  Tbl1xr1  Nedd4l  Huwe1  Pcnp  Dcaf11  Arrb2  Ubxn2a  Bag6  Znrf1  Derl1  Ppp2r5c | web link |
| oxidation-reduction process | Gene Ontology | GO:0055114 | 22 (7.24%) | 546 (2.60%) | 1.72919846612e-05 | 0.00075220133276 | Grhpr  Aox3  Fmo5  Acox1  Ero1b  Maob  Bdh2  Tm7sf2  Prxl2c  Cyp2r1  Dhrs7b  Uox  Cyp3a59  P4ha1  Hsd17b2  Hsd17b6  Akr1c20  Aspdh  Cyp2d10  Rdh13  Cp  Alkbh3 | web link |
| translation | Gene Ontology | GO:0006412 | 15 (4.93%) | 303 (1.45%) | 3.97186619352e-05 | 0.00110778349835 | Rpl22l1  Mrpl24  Rbm3  Mrpl30  Eef1d  Eif5  Cpeb3  Rpl35a  Farsa  Mtif3  Eif3e  Mrpl47  Rps19  Rpl18  Mettl17 | web link |
| regulation of RNA splicing | Gene Ontology | GO:0043484 | 6 (1.97%) | 46 (0.22%) | 5.09325746367e-05 | 0.00110778349835 | Esrp2  Hnrnpl  Hnrnph3  Ptbp2  Snrnp70  Clk4 | web link |
| positive regulation of dendritic spine development | Gene Ontology | GO:0060999 | 5 (1.64%) | 32 (0.15%) | 9.06731110428e-05 | 0.00157771213215 | Mapk6  Mapkapk5  Hnrnpk  Zmynd8  Cpeb3 | web link |
| protein transport | Gene Ontology | GO:0015031 | 22 (7.24%) | 631 (3.01%) | 0.00014596971382 | 0.00211656085038 | Tomm5  Ktn1  Nup98  Derl1  Mon2  Golph3  Yif1b  Sec24c  Atg4d  Chmp3  Ubap1  Atg16l1  Atg16l2  Sec24b  Tbc1d17  Rab4b  Snx10  Dennd10  Myo1c  Arrb2  Tomm22  Mcfd2 | web link |
| phosphorylation | Gene Ontology | GO:0016310 | 21 (6.91%) | 641 (3.06%) | 0.000470838414495 | 0.00585184886587 | Papss2  Csnk1g3  Pfkfb2  Sgk1  Mapk15  Snrk  Pnkp  Dcakd  Uckl1  Cdk8  Ulk1  Mast3  Mapk6  Fggy  Akap8l  Abl1  Mapkapk5  Mapk9  Clk4  Adk  Map4k2 | web link |
| nucleic acid phosphodiester bond hydrolysis | Gene Ontology | GO:0090305 | 8 (2.63%) | 129 (0.62%) | 0.000597303958653 | 0.00649568055035 | Dna2  Enpp3  Alkbh3  Rexo5  Gm49673  Rnaset2b  Dclre1c  Aen | web link |
| lipid homeostasis | Gene Ontology | GO:0055088 | 5 (1.64%) | 49 (0.23%) | 0.000702225727494 | 0.00670861712648 | Acox1  Pnpla2  Pnpla8  Thada  Nr1h3 | web link |
| regulation of cell growth | Gene Ontology | GO:0001558 | 5 (1.64%) | 50 (0.24%) | 0.000771105416837 | 0.00670861712648 | Epb41  Lamtor2  4932438A13Rik  Igfbp4  Igfbp5 | web link |
| lipid metabolic process | Gene Ontology | GO:0006629 | 18 (5.92%) | 544 (2.60%) | 0.00105812148802 | 0.00776558939558 | Lipc  4932438A13Rik  Cers2  Tm7sf2  Acacb  Gba  Acox1  Pnpla8  Pnpla2  Srebf1  Insig2  Lpcat3  Hsd17b2  Gm44805  Pnpla6  Mtmr2  Hsd17b6  Nr1h3 | web link |
| cell cycle | Gene Ontology | GO:0007049 | 19 (6.25%) | 595 (2.84%) | 0.00117331703019 | 0.00776558939558 | Zfyve19  Ccar1  Banp  Anapc15  Rabgap1  Inca1  Anapc10  Epb41  Pcnp  Septin2  Pard3  Mapk6  Chmp3  Cenpx  Nde1  Txnl4a  Cdc123  Ncapd3  Anapc5 | web link |
| steroid metabolic process | Gene Ontology | GO:0008202 | 7 (2.30%) | 112 (0.53%) | 0.00124621414235 | 0.00776558939558 | Tm7sf2  Gba  Cyp3a59  Srebf1  Insig2  Hsd17b6  Akr1c20 | web link |
| in utero embryonic development | Gene Ontology | GO:0001701 | 12 (3.95%) | 293 (1.40%) | 0.00124963507515 | 0.00776558939558 | Tctn1  Tbx3  Prrc2b  H13  Tcf7l2  Mgat1  N4bp2l2  Vegfa  Sec24c  Smpd4  Hsd17b2  Dusp3 | web link |
| cellular response to DNA damage stimulus | Gene Ontology | GO:0006974 | 16 (5.26%) | 470 (2.24%) | 0.00148492954478 | 0.00861259135974 | Dna2  Alkbh3  Sgk1  Huwe1  Parp3  Mapk15  Chd2  Fbxo6  Cenpx  Abl1  Hdac10  Yap1  Shld2  Dclre1c  Eya3  Aen | web link |
| DNA repair | Gene Ontology | GO:0006281 | 13 (4.28%) | 352 (1.68%) | 0.0020040021394 | 0.010896761633 | Dna2  Dclre1c  Pnkp  Kmt5b  Huwe1  Parp3  Fbxo6  Cenpx  Abl1  Hdac10  Shld2  Alkbh3  Eya3 | web link |
| lipid catabolic process | Gene Ontology | GO:0016042 | 7 (2.30%) | 127 (0.61%) | 0.00255904405934 | 0.0130962843037 | Lipc  Tbl1xr1  Pnpla2  Pnpla6  Gm44805  Pnpla8  Ces1f | web link |
| cell division | Gene Ontology | GO:0051301 | 13 (4.28%) | 365 (1.74%) | 0.00274208331849 | 0.013253402706 | Zfyve19  Anapc15  Anapc10  Epb41  Nde1  Septin2  Pard3  Cenpx  Chmp3  Txnl4a  Cdc123  Ncapd3  Anapc5 | web link |
| ubiquitin-dependent ERAD pathway | Gene Ontology | GO:0030433 | 5 (1.64%) | 71 (0.34%) | 0.00370282743224 | 0.016124095295 | Derl1  Bag6  Psmc6  Fbxo6  Ube2g2 | web link |
| autophagy | Gene Ontology | GO:0006914 | 8 (2.63%) | 172 (0.82%) | 0.00370668857356 | 0.016124095295 | Ulk1  Tmbim6  Gba  Atg16l1  Atg4d  Abl1  Hdac10  Tbc1d17 | web link |
| positive regulation of protein ubiquitination | Gene Ontology | GO:0031398 | 5 (1.64%) | 72 (0.34%) | 0.00393288695869 | 0.0162933888289 | Derl1  Mapk9  Rassf5  Huwe1  Mapk15 | web link |
| regulation of growth | Gene Ontology | GO:0040008 | 5 (1.64%) | 77 (0.37%) | 0.00523859755063 | 0.020716272132 | Safb  Igfbp5  Vegfa  Igfbp4  Epc1 | web link |
| response to cytokine | Gene Ontology | GO:0034097 | 5 (1.64%) | 78 (0.37%) | 0.00553246452448 | 0.0209271484187 | Acp5  Slco1b2  Lifr  Ghr  Serpina3m | web link |
| response to glucose | Gene Ontology | GO:0009749 | 5 (1.64%) | 81 (0.39%) | 0.00648367794913 | 0.0235033325656 | Mlxipl  Pfkfb2  Srebf1  Khk  Tcf7l2 | web link |
| positive regulation of translation | Gene Ontology | GO:0045727 | 5 (1.64%) | 82 (0.39%) | 0.00682471055708 | 0.0237499927386 | Plxnb2  Hnrnpl  Rbm3  Eif3e  Cpeb3 | web link |
| negative regulation of ERK1 and ERK2 cascade | Gene Ontology | GO:0070373 | 5 (1.64%) | 83 (0.40%) | 0.00717809777099 | 0.0240190194645 | Abl1  Ndrg2  Tnip1  Gm20498  Dusp3 | web link |
| regulation of GTPase activity | Gene Ontology | GO:0043087 | 5 (1.64%) | 85 (0.41%) | 0.00792281858796 | 0.0255290821168 | Plxnb2  Rabgap1  Fgd6  Arhgap12  Ralbp1 | web link |
| intracellular signal transduction | Gene Ontology | GO:0035556 | 13 (4.28%) | 421 (2.01%) | 0.00885114328757 | 0.0275017666435 | Map4k2  Sgk1  Mapk15  Snrk  Rassf5  Ss18  Mast3  Mapk6  Wsb1  Igfbp5  Tns3  Mapkapk5  Mapk9 | web link |
| activation of GTPase activity | Gene Ontology | GO:0090630 | 5 (1.64%) | 93 (0.44%) | 0.0114389415194 | 0.0343168245583 | Rabgap1  Arhgef19  Eif5  Tbc1d17  Bcas3 | web link |
| cell migration | Gene Ontology | GO:0016477 | 9 (2.96%) | 255 (1.22%) | 0.0124581467267 | 0.0350231857234 | Cd47  Golph3  Arpc5  Coro1b  Tns3  Vegfa  Itgav  Nde1  Lcp1 | web link |
| positive regulation of transcription, DNA-templated | Gene Ontology | GO:0045893 | 18 (5.92%) | 691 (3.30%) | 0.0124795259474 | 0.0350231857234 | Mapk9  Btrc  Foxk2  Tbx3  Mlxipl  Nup98  Tcf7l2  Banp  Ss18  Srebf1  Tbl1xr1  Nsd1  Ccar1  Smarca2  Nr1h3  Rbm39  Rfxap  Yap1 | web link |
| protein ubiquitination | Gene Ontology | GO:0016567 | 15 (4.93%) | 545 (2.60%) | 0.013802855634 | 0.0362621337413 | Btrc  Znrf1  Nedd4l  Huwe1  Anapc10  Pcnp  Dcaf11  Wsb1  Arrb2  Med7  Fbxo6  Cdk8  Rnf214  Anapc5  Ube2g2 | web link |
| cholesterol metabolic process | Gene Ontology | GO:0008203 | 5 (1.64%) | 98 (0.47%) | 0.0141058824192 | 0.0362621337413 | Lipc  Srebf1  Insig2  Tm7sf2  Gba | web link |
| negative regulation of transcription by RNA polymerase II | Gene Ontology | GO:0000122 | 22 (7.24%) | 913 (4.36%) | 0.0141714085886 | 0.0362621337413 | Zbtb42  Hnrnpk  Vegfa  Crem  Hdac10  Cnot2  Yap1  Foxk2  Mlxipl  Mier1  Mier2  Cpeb3  Epc1  Mapk15  N4bp2l2  Gzf1  Nr1h3  Tcf7l2  Tbx3  Sap130  Tbl1xr1  Srebf1 | web link |
| multicellular organism growth | Gene Ontology | GO:0035264 | 5 (1.64%) | 103 (0.49%) | 0.01716183427 | 0.042659416614 | Tcf7l2  Tbl1xr1  Plekha1  Ndufs6  Slc25a25 | web link |
| negative regulation of transcription, DNA-templated | Gene Ontology | GO:0045892 | 16 (5.26%) | 622 (2.97%) | 0.0198962330927 | 0.04556216443 | Dhrs7b  Pphln1  Zmynd8  Tbl1xr1  Mlxipl  Btrc  Dnajb6  Tcf7l2  Epc1  Tbx3  Gzf1  Hdac10  Ccar1  Smarca2  Nr1h3  Foxk2 | web link |
| positive regulation of peptidyl-tyrosine phosphorylation | Gene Ontology | GO:0050731 | 5 (1.64%) | 107 (0.51%) | 0.0199007154982 | 0.04556216443 | Abl1  Arrb2  Hax1  Ghr  Vegfa | web link |
| cellular response to insulin stimulus | Gene Ontology | GO:0032869 | 5 (1.64%) | 107 (0.51%) | 0.0199007154982 | 0.04556216443 | Hnrnpk  Srebf1  Insig2  Ghr  Sgk1 | web link |
| metabolic process | Gene Ontology | GO:0008152 | 7 (2.30%) | 190 (0.91%) | 0.0211853137395 | 0.0472421646965 | Dna2  Acacb  Gba  Ctbs  Pfkfb2  Enpp3  Papss2 | web link |
| regulation of cell shape | Gene Ontology | GO:0008360 | 6 (1.97%) | 149 (0.71%) | 0.0217205354926 | 0.0472421646965 | Plxnb2  Fn1  Cdc42ep4  Vegfa  Rhobtb1  Epb41 | web link |
| positive regulation of transcription by RNA polymerase II | Gene Ontology | GO:0045944 | 26 (8.55%) | 1201 (5.73%) | 0.0273634512721 | 0.0580639087969 | Hsf2  Abhd14b  Hnrnpk  Vegfa  Crem  Rfxap  Yap1  Foxk2  Mlxipl  Safb  Epc1  Ss18  Srebf1  Gzf1  Gtf2a2  Smarca2  Bcas3  Nr1h3  Hax1  Tcf7l2  Tbx3  Tbl1xr1  Mettl23  Akap8l  Tnip1  Gmeb2 | web link |
| chromatin organization | Gene Ontology | GO:0006325 | 9 (2.96%) | 298 (1.42%) | 0.030468017511 | 0.0631123219871 | Chd2  Bag6  Banp  Kmt5b  Epc1  Naa60  Nsd1  Eya3  Tbl1xr1 | web link |
| mRNA processing | Gene Ontology | GO:0006397 | 10 (3.29%) | 351 (1.67%) | 0.0328725069342 | 0.0665094907739 | Esrp2  U2af1  Rbm39  Hnrnpk  Akap8l  Ppil3  Hnrnpl  Txnl4a  Ptbp2  Thoc7 | web link |
| positive regulation of protein kinase B signaling | Gene Ontology | GO:0051897 | 5 (1.64%) | 126 (0.60%) | 0.0367546652596 | 0.0726739972179 | Arrb2  Hax1  Vegfa  Tcf7l2  Igfbp5 | web link |
| protein autophosphorylation | Gene Ontology | GO:0046777 | 6 (1.97%) | 174 (0.83%) | 0.0416817677762 | 0.080584751034 | Ulk1  Mapk15  Csnk1g3  Abl1  Mapkapk5  Clk4 | web link |
| RNA splicing | Gene Ontology | GO:0008380 | 8 (2.63%) | 272 (1.30%) | 0.0455236098304 | 0.0845573274846 | Ppil3  U2af1  Rbm39  Esrp2  Hnrnpk  Ptbp2  Txnl4a  Thoc7 | web link |
| dephosphorylation | Gene Ontology | GO:0016311 | 6 (1.97%) | 178 (0.85%) | 0.0456803953078 | 0.0845573274846 | Acp5  Pnkp  Pfkfb2  Inpp4a  Mtmr2  Dusp3 | web link |
| endocytosis | Gene Ontology | GO:0006897 | 7 (2.30%) | 226 (1.08%) | 0.0472681465494 | 0.0856735156207 | Gm20498  Ralbp1  Snx10  Ghr  Csnk1g3  Arrb2  Abl1 | web link |
| cytoskeleton organization | Gene Ontology | GO:0007010 | 5 (1.64%) | 136 (0.65%) | 0.0483265550471 | 0.0858042916143 | Sh3d19  Ss18  Mast3  Znrf1  Epb41 | web link |
| methylation | Gene Ontology | GO:0032259 | 6 (1.97%) | 184 (0.88%) | 0.0521123608233 | 0.0906755078325 | Vcpkmt  Kmt5b  Mettl17  Mettl23  Nsd1  Setd4 | web link |
| fatty acid metabolic process | Gene Ontology | GO:0006631 | 6 (1.97%) | 193 (0.92%) | 0.0627477487288 | 0.107040277243 | Lipc  Acacb  Acox1  Crem  Ndufs6  Pnpla8 | web link |
| protein phosphorylation | Gene Ontology | GO:0006468 | 14 (4.61%) | 616 (2.94%) | 0.0661855479903 | 0.110733512984 | Ywhaz  Map4k2  Sgk1  Csnk1g3  Mapk15  Snrk  Cdk8  Ulk1  Mast3  Mapk6  Abl1  Mapkapk5  Mapk9  Clk4 | web link |
| cell-cell adhesion | Gene Ontology | GO:0098609 | 5 (1.64%) | 155 (0.74%) | 0.0756152056388 | 0.124123073407 | Pkp4  Igsf5  Cd47  Pard3  Gm20498 | web link |
| angiogenesis | Gene Ontology | GO:0001525 | 7 (2.30%) | 254 (1.21%) | 0.0776821681392 | 0.125154604224 | Ywhaz  Fn1  Itgav  Vegfa  Pnpla6  Bcas3  Nrp2 | web link |
| neuron projection development | Gene Ontology | GO:0031175 | 5 (1.64%) | 159 (0.76%) | 0.0822377037221 | 0.130085094979 | Atxn10  Camsap2  Mapk9  Ulk1  Septin2 | web link |
| peptidyl-serine phosphorylation | Gene Ontology | GO:0018105 | 5 (1.64%) | 163 (0.78%) | 0.0891581391313 | 0.137067967265 | Csnk1g3  Mapkapk5  Mapk9  Sgk1  Mast3 | web link |
| positive regulation of cell population proliferation | Gene Ontology | GO:0008284 | 13 (4.28%) | 590 (2.81%) | 0.0898031509668 | 0.137067967265 | Fn1  Tbx3  Mlxipl  Mapk15  Cd47  Itgav  Prlr  Vegfa  Lifr  Tial1  Tns3  Ccar1  Yap1 | web link |
| protein dephosphorylation | Gene Ontology | GO:0006470 | 5 (1.64%) | 169 (0.81%) | 0.100086338862 | 0.150129508292 | Eya3  Btrc  Ppp4r3a  Dusp3  Ppp2r5c | web link |
| cell differentiation | Gene Ontology | GO:0030154 | 18 (5.92%) | 918 (4.38%) | 0.120999975999 | 0.178423693423 | Pard3  Bag6  Cnmd  Prrc2b  Huwe1  Unc45a  Itgav  Nedd4l  Septin2  Vegfa  Eya3  Tmem176b  Nde1  Ndrg2  Sema4g  Ggnbp1  Nrp2  Nr1h3 | web link |
| spermatogenesis | Gene Ontology | GO:0007283 | 10 (3.29%) | 458 (2.19%) | 0.131234057914 | 0.190289383976 | Hsf2  Bag6  4932438A13Rik  Acox1  Septin2  Crem  Slc4a2  Atat1  Ggnbp1  Plekha1 | web link |
| positive regulation of cell migration | Gene Ontology | GO:0030335 | 6 (1.97%) | 245 (1.17%) | 0.146906967596 | 0.209523052146 | Fn1  Myo1c  Itgav  Vegfa  Ccar1  Sema4g | web link |
| cilium assembly | Gene Ontology | GO:0060271 | 5 (1.64%) | 196 (0.94%) | 0.156796436029 | 0.220020805395 | Atxn10  Pcm1  Septin2  Tctn1  Snx10 | web link |
| positive regulation of GTPase activity | Gene Ontology | GO:0043547 | 7 (2.30%) | 310 (1.48%) | 0.16556612175 | 0.228638930036 | Ralbp1  Arhgef19  Agap3  Arhgap12  Pkp4  Rabgap1  Bcas3 | web link |
| protein phosphopantetheinylation | Gene Ontology | GO:0018215 | 5 (1.64%) | 201 (0.96%) | 0.168515057178 | 0.229075155852 | Mgat1  Ero1b  Uba5  Ggt6  Atat1 | web link |
| Wnt signaling pathway | Gene Ontology | GO:0016055 | 5 (1.64%) | 205 (0.98%) | 0.178130319432 | 0.238420581394 | Tmem131l  Csnk1g3  Ndrg2  Btrc  Tcf7l2 | web link |
| regulation of catalytic activity | Gene Ontology | GO:0050790 | 6 (1.97%) | 270 (1.29%) | 0.199543726791 | 0.263034912588 | Dock9  Dennd10  Septin2  Nucb1  Lamtor2  Ppp2r5c | web link |
| negative regulation of apoptotic process | Gene Ontology | GO:0043066 | 11 (3.62%) | 570 (2.72%) | 0.207535518043 | 0.269486418952 | Bag6  Sgk1  Fn1  Prlr  Higd1a  Golph3  Hax1  Tbx3  Hnrnpk  Vegfa  Tmbim6 | web link |
| intracellular protein transport | Gene Ontology | GO:0006886 | 6 (1.97%) | 291 (1.39%) | 0.248336072444 | 0.316505166646 | Snx10  Rabgap1  Sec24b  Sec24c  Tomm22  Tbc1d17 | web link |
| regulation of transcription by RNA polymerase II | Gene Ontology | GO:0006357 | 25 (8.22%) | 1489 (7.10%) | 0.251021339064 | 0.316505166646 | Hsf2  Zbtb42  Hnrnpk  Vegfa  Crem  Rfxap  Foxk2  Mlxipl  Safb  Epc1  Gzf1  Zfp874b  Zfp58  Rbm39  Zbtb43  Zfp938  Tbx3  Zfp235  Tcf7l2  Tbl1xr1  Srebf1  Gmeb2  Zfp367  Lcorl  Med7 | web link |
| positive regulation of protein phosphorylation | Gene Ontology | GO:0001934 | 5 (1.64%) | 235 (1.12%) | 0.255895160555 | 0.318041128118 | Arrb2  Mapk15  Abl1  Mapk9  Vegfa | web link |
| apoptotic process | Gene Ontology | GO:0006915 | 10 (3.29%) | 567 (2.71%) | 0.309304384371 | 0.374789109072 | Bag6  Tial1  Sgk1  Rassf5  Ccar1  Mindy3  Atg4d  Abl1  Tmbim6  Aen | web link |
| transmembrane transport | Gene Ontology | GO:0055085 | 10 (3.29%) | 568 (2.71%) | 0.311183439736 | 0.374789109072 | Ralbp1  Slc2a9  Clcn2  Slc25a25  Slc39a8  Slc39a9  Slc35b3  Slco1b2  Slc4a2  Slc43a1 | web link |
| ubiquitin-dependent protein catabolic process | Gene Ontology | GO:0006511 | 5 (1.64%) | 256 (1.22%) | 0.314478217957 | 0.374789109072 | Nedd4l  Btrc  Bag6  Usp40  Ube2g2 | web link |
| response to drug | Gene Ontology | GO:0042493 | 6 (1.97%) | 329 (1.57%) | 0.343382852097 | 0.403706866655 | Ywhaz  Acacb  Ss18  Srebf1  Slco1b2  Mapk9 | web link |
| nervous system development | Gene Ontology | GO:0007399 | 7 (2.30%) | 410 (1.96%) | 0.384888617203 | 0.446470795956 | Fn1  Nde1  Vegfa  Atxn10  Ndrg2  Nrp2  Sema4g | web link |
| ion transport | Gene Ontology | GO:0006811 | 10 (3.29%) | 622 (2.97%) | 0.41537232023 | 0.475491998158 | Slc38a4  Slc41a2  Slc38a3  Clcn2  Slc4a2  Slc39a8  Slc39a9  Slco1b2  Nipa2  Cp | web link |
| negative regulation of gene expression | Gene Ontology | GO:0010629 | 5 (1.64%) | 295 (1.41%) | 0.426143032346 | 0.481486283299 | Hnrnpk  Acacb  Vegfa  Cnpy2  Yap1 | web link |
| regulation of gene expression | Gene Ontology | GO:0010468 | 6 (1.97%) | 376 (1.79%) | 0.464294906258 | 0.517867395441 | Dhrs7b  Tbl1xr1  Fn1  Hnrnpk  Ubxn2a  Yap1 | web link |
| regulation of transcription, DNA-templated | Gene Ontology | GO:0006355 | 21 (6.91%) | 1403 (6.69%) | 0.473019477996 | 0.520920184629 | Zfp874b  Dhrs7b  Hsf2  Zmynd8  Foxk2  Mier1  Safb  Tcf7l2  Slc39a8  Tbx3  Srebf1  Zfp235  Crem  Cnot2  Hdac10  Zfp938  Ccar1  Smarca2  Zfp58  Sap130  Nr1h3 | web link |
| multicellular organism development | Gene Ontology | GO:0007275 | 15 (4.93%) | 1040 (4.96%) | 0.546458655392 | 0.594273787739 | Plxnb2  Banp  Cnmd  Tctn1  Unc45a  Itgav  Tbx3  Vegfa  Nrp2  Nde1  Cnot2  Ndrg2  Ggnbp1  Eya3  Sema4g | web link |
| cell adhesion | Gene Ontology | GO:0007155 | 8 (2.63%) | 571 (2.72%) | 0.58919295418 | 0.632836876712 | Pard3  Fn1  Cd47  Itgav  Pkp4  Abl1  Mpdz  Zyx | web link |
| positive regulation of apoptotic process | Gene Ontology | GO:0043065 | 5 (1.64%) | 360 (1.72%) | 0.600668395117 | 0.637294516771 | Abl1  Ccar1  Mapk9  Tcf7l2  Tomm22 | web link |
| immune system process | Gene Ontology | GO:0002376 | 5 (1.64%) | 481 (2.29%) | 0.829969249866 | 0.869967767932 | Gm49673  Rnaset2b  Bag6  Dclre1c  Map4k2 | web link |
| proteolysis | Gene Ontology | GO:0006508 | 7 (2.30%) | 664 (3.17%) | 0.850896734398 | 0.881285903484 | Ctsa  H13  Usp40  Ggt6  Mindy3  Atg4d  Josd2 | web link |
| positive regulation of gene expression | Gene Ontology | GO:0010628 | 5 (1.64%) | 525 (2.50%) | 0.880989051054 | 0.901718205196 | Arrb2  Mapk9  Vegfa  Fn1  Yap1 | web link |
| biological\_process | Gene Ontology | GO:0008150 | 32 (10.53%) | 2834 (13.52%) | 0.951528721462 | 0.962593008921 | C87436  Gpank1  Ccdc163  Polr1has  Ccdc97  Smim1  Tmem234  1110065P20Rik  Chchd1  Cdv3  Mrpl30  Slc39a9  Fam204a  Tmem256  Ccdc30  Mcrip2  Iqcc  Golga1  Gm826  Fgd6  Haghl  Wbp1l  Ggt6  Tmem185a  Gtpbp8  Msantd2  Rsrp1  Cep170  Gramd3  Wsb1  Wdr20  Uhrf1bp1l | web link |
| signal transduction | Gene Ontology | GO:0007165 | 16 (5.26%) | 2811 (13.41%) | 0.999999164186 | 0.999999164186 | Plxnb2  Ywhaz  Ulk1  Ralbp1  Erbin  Rassf5  Raph1  Agap3  Arrb2  Manf  P2ry2  Vegfa  Ndrg2  Arhgap12  Zyx  Ppp2r5c | web link |
