# Supplementary material for: Regulation of Alternative Splicing of Lipid Metabolism Genes in Sepsis-Induced Liver Damage by RNA-Binding Proteins
Source: Inflammation. 2024 May 9;47(6):1952–68. doi: 10.1007/s10753-024-02017-2 (PMC11607010; doi:10.1007/s10753-024-02017-2)
Supplement: Supplementary file 1 — Supplementary file1 (ZIP 38655 KB) [file 10753_2024_2017_MOESM1_ESM.zip › Sup5_AS_GO.zip!/Sup5_AS_GO/ras/di_RAS/GO_KEGG/CLP_vs_Sham_and_CLPDCA_vs_Sham_di_RAS/CLP_vs_Sham_diUp_nir_GO_analysis/CLP_vs_Sham_diUp_nir_GO_enrichment_web_F.html]

GO enrichment analyze


# GO enrichment analyze result


| Term | Database | ID | Input number (345) | Background number (20698) | P-Value | Corrected P-Value | Input | Hyperlink |
| --- | --- | --- | --- | --- | --- | --- | --- | --- |
| protein binding | Gene Ontology | GO:0005515 | 154 (44.64%) | 6748 (32.60%) | 1.72677426358e-06 | 0.00011569387566 | Gpank1  Cers2  Hnrnpk  Hnrnpl  Edc4  Nde1  Mapkapk5  Anapc5  Sgk1  Mapk15  Ctsa  Iqcc  Pnkd  Lamtor2  Smarca2  Rbm39  Bcas3  Kctd17  Atg16l1  Atg16l2  Inca1  Unc45a  Nsd1  Septin2  Mapk6  Abl1  Mapk9  Nipsnap1  Cep170  Dnajc5  Helz  Slmap  Tnip1  Arhgef19  Ccar1  Tial1  Ralbp1  Neb  Raph1  Slco1b2  Cnpy2  Ptbp2  Yap1  Smg7  Ghr  Safb  Dnajb6  Cpeb3  Mcrip2  Golga1  Srebf1  Lifr  Sec24b  Relch  Fndc3b  Dhrs7b  Ubap1  Tctn1  Gm20498  Camsap2  Rhobtb1  Hax1  Tcf7l2  Myl6  Pkp4  Polrmt  Ppp4r3a  Zyx  Ywhaz  Kctd2  Lipc  Dna2  Mff  Erbin  Nedd4l  Fkbp7  Wsb1  Ubxn2a  Cdk8  Smoc1  Eya3  Ehbp1l1  Rwdd3  Pcm1  Coro1b  Derl1  Zbtb42  Zbtb43  Vegfa  Vps13d  Mtmr2  Uggt1  Fn1  Mlxipl  Huwe1  Mpdz  Gzf1  Tns3  Nr1h3  Dmxl1  Zmynd8  Gba  Btrc  Epb41  P4ha1  Ube2g2  Mia2  Sap130  Sema4g  Sh3d19  Tbl1xr1  U2af1  Serpinb6a  Ggnbp1  Agap3  Arrb2  Fbxo6  Tomm22  Crem  Pard3  Znrf1  Uba5  Arhgap12  Igfbp4  Tmem176b  Bag6  Lcp1  Kctd20  Nrp2  Plxnb2  Banp  Pnkp  Kmt5b  Nipa2  Dcaf11  N4bp2l2  Atxn10  Thoc7  Rragc  Prlr  Rassf5  Eif5  Cdc42ep4  Foxk2  Chd2  Zc3h7a  Cd47  Myo1c  Anapc10  Pnpla2  Mast3  Wdr20  Setd4  Ppp2r5c | web link |
| transferase activity | Gene Ontology | GO:0016740 | 48 (13.91%) | 1771 (8.56%) | 0.000556805574782 | 0.0186529867552 | Vcpkmt  Pfkfb2  Cers2  Uckl1  Snrk  Csnk1g3  Naa60  Ugt2b36  Znrf1  Mapkapk5  Atat1  Adk  Uggt1  Map4k2  Sgk1  Mapk15  Kmt5b  Huwe1  Naa80  Pigp  Mocs2  Mgat1  Nsd1  Clk4  Mettl17  Ulk1  Gba  Gmppa  Mpst  Ugt1a7c  Ggt6  Fggy  Abl1  Mapk9  Upp2  Papss2  Ugt1a6b  Flad1  Polrmt  Parp3  Mettl23  Nedd4l  Mast3  Lpcat3  Cdk8  Mapk6  Setd4  Ube2g2 | web link |
| growth factor binding | Gene Ontology | GO:0019838 | 5 (1.45%) | 47 (0.23%) | 0.00107964081599 | 0.0241119782238 | Igfbp4  Vegfa  Lifr  Ghr  Igfbp5 | web link |
| oxidoreductase activity | Gene Ontology | GO:0016491 | 22 (6.38%) | 684 (3.30%) | 0.00265784636971 | 0.0445189266927 | Grhpr  Aox3  Fmo5  Acox1  Ero1b  Maob  Bdh2  Tm7sf2  Cyp2r1  Dhrs7b  Uox  Cyp3a59  P4ha1  Hsd17b2  Hsd17b6  Akr1c20  Aspdh  Cyp2d10  Rdh13  Cox7a2  Cp  Alkbh3 | web link |
| hydrolase activity | Gene Ontology | GO:0016787 | 43 (12.46%) | 1696 (8.19%) | 0.0038396671693 | 0.0514515400686 | Entpd5  Haghl  Abhd14b  Hdac10  Mtmr2  Dusp3  Ces1f  Pnpla2  Ctsa  Usp40  Pnkd  Mindy3  Atg4d  Smarca2  Josd2  Dclre1c  Lyplal1  Acp5  Nagpa  Kynu  Rragc  Gba  Ctbs  Pip4p2  H13  Chd2  Ggt6  Gm49673  Rnaset2b  Psmc6  Aen  Lipc  Dna2  Rab4b  Pfkfb2  Enpp3  Helz  Nudt13  Pnpla6  Nudt14  Smpd4  Pnpla8  Eya3 | web link |
| identical protein binding | Gene Ontology | GO:0042802 | 43 (12.46%) | 1735 (8.38%) | 0.00572189653218 | 0.0638945112761 | Pard3  Fn1  Coro1b  Maob  Hsf2  Vegfa  Nde1  Bag6  Gm44805  Lcp1  Nrp2  Kctd20  Banp  Dnajb6  Mtmr2  Tnip1  Mri1  Atxn10  Dbi  Kctd17  Ghr  Acacb  Atg16l1  Rps19  Inca1  Rassf5  Chmp3  Septin2  P4ha1  Ube2g2  Psmc6  Ywhaz  Upp2  Lipc  Mff  Derl1  Flad1  Mpst  Gramd3  Hnrnpk  Arrb2  Nudt14  Gmeb2 | web link |
| transcription corepressor activity | Gene Ontology | GO:0003714 | 8 (2.32%) | 168 (0.81%) | 0.00730945713343 | 0.0699619468485 | Dhrs7b  Zmynd8  Tbl1xr1  Mier1  Mier2  N4bp2l2  Ccar1  Yap1 | web link |
| structural constituent of ribosome | Gene Ontology | GO:0003735 | 8 (2.32%) | 172 (0.83%) | 0.00837135121233 | 0.0701100664033 | Rpl22l1  Mrpl24  Rps10  Mrpl30  Rps19  Rpl35a  Mrpl47  Rpl18 | web link |
| kinase activity | Gene Ontology | GO:0016301 | 19 (5.51%) | 631 (3.05%) | 0.00991794215786 | 0.0738335693974 | Papss2  Csnk1g3  Sgk1  Uckl1  Mapk15  Snrk  Pfkfb2  Cdk8  Ulk1  Map4k2  Mapk6  Fggy  Akap8l  Abl1  Mapkapk5  Mapk9  Clk4  Adk  Mast3 | web link |
| protein serine kinase activity | Gene Ontology | GO:0106310 | 12 (3.48%) | 340 (1.64%) | 0.0121370524951 | 0.0755153424323 | Ulk1  Sgk1  Csnk1g3  Snrk  Mapk15  Map4k2  Mapk6  Cdk8  Mapkapk5  Mapk9  Clk4  Mast3 | web link |
| protein threonine kinase activity | Gene Ontology | GO:0106311 | 12 (3.48%) | 341 (1.65%) | 0.0123980412949 | 0.0755153424323 | Map4k2  Sgk1  Csnk1g3  Snrk  Mapk15  Ulk1  Mast3  Mapk6  Cdk8  Mapkapk5  Mapk9  Clk4 | web link |
| protein N-terminus binding | Gene Ontology | GO:0047485 | 6 (1.74%) | 123 (0.59%) | 0.0171613238441 | 0.0958173914629 | Zmynd8  Tbl1xr1  Acox1  Hax1  Eif3e  Epb41 | web link |
| transferase activity, transferring acyl groups | Gene Ontology | GO:0016746 | 7 (2.03%) | 170 (0.82%) | 0.0241523529681 | 0.116338362138 | Lipc  Taz  Ggt6  Lpcat3  Naa60  Atat1  Naa80 | web link |
| nucleotide binding | Gene Ontology | GO:0000166 | 41 (11.88%) | 1795 (8.67%) | 0.0243095085065 | 0.116338362138 | Entpd5  Flad1  Uckl1  Dcakd  Snrk  Csnk1g3  Uba5  Mapkapk5  Adk  Map4k2  Sgk1  Mapk15  Mocs2  Mocs1  Mast3  Smarca2  Clk4  Mccc2  Ulk1  Rragc  Acacb  Eif5  Septin2  Chd2  Mapk6  Ube2g2  Abl1  Mapk9  Gtpbp8  Psmc6  Papss2  Rab4b  Naxd  Myo1c  Dna2  Helz  Farsa  Agap3  Atp11c  Rhobtb1  Cdk8 | web link |
| lipid binding | Gene Ontology | GO:0008289 | 10 (2.90%) | 299 (1.44%) | 0.0290284618586 | 0.129660462969 | Dbi  Lipc  Zfyve19  Snx10  Golph3  Osbpl9  Pard3  Insig2  Plekha1  Gm44805 | web link |
| RNA binding | Gene Ontology | GO:0003723 | 23 (6.67%) | 915 (4.42%) | 0.0335363235644 | 0.140433354926 | Rbm3  Esrp2  Hnrnpk  Hnrnpl  Tial1  Ptbp2  Rpl18  Safb  Cpeb3  Rexo5  Rbm39  Rpl22l1  Rps10  Rps19  Gm49673  Rnaset2b  Mrpl24  Dna2  U2af1  Thoc7  Helz  Hnrnph3  Snrnp70 | web link |
| transferase activity, transferring glycosyl groups | Gene Ontology | GO:0016757 | 8 (2.32%) | 230 (1.11%) | 0.0395521638858 | 0.154801368308 | Upp2  Mgat1  Gba  Parp3  Ugt1a6b  Ugt1a7c  Ugt2b36  Uggt1 | web link |
| nucleic acid binding | Gene Ontology | GO:0003676 | 16 (4.64%) | 594 (2.87%) | 0.0415884273066 | 0.154801368308 | Gpank1  Rbm3  Hnrnph3  U2af1  Safb  Enpp3  Cpeb3  Esrp2  Hnrnpk  Hnrnpl  Tial1  Ptbp2  Rexo5  Snrnp70  Rbm39  Aen | web link |
| protein serine/threonine kinase activity | Gene Ontology | GO:0004674 | 12 (3.48%) | 412 (1.99%) | 0.0444343613835 | 0.156689590142 | Map4k2  Sgk1  Csnk1g3  Snrk  Mapk15  Ulk1  Mast3  Mapk6  Cdk8  Mapkapk5  Mapk9  Clk4 | web link |
| transcription regulatory region sequence-specific DNA binding | Gene Ontology | GO:0000976 | 8 (2.32%) | 246 (1.19%) | 0.0546809204387 | 0.177381496455 | Foxk2  Tcf7l2  Srebf1  Hnrnpl  Tbl1xr1  Smarca2  Nr1h3  Yap1 | web link |
| ATP binding | Gene Ontology | GO:0005524 | 33 (9.57%) | 1484 (7.17%) | 0.0555971854561 | 0.177381496455 | Entpd5  Flad1  Uckl1  Dcakd  Snrk  Chd2  Csnk1g3  Uba5  Mapkapk5  Adk  Map4k2  Sgk1  Mapk15  Smarca2  Clk4  Mccc2  Ulk1  Acacb  Papss2  Mapk6  Ube2g2  Abl1  Naxd  Psmc6  Mapk9  Dna2  Pfkfb2  Myo1c  Helz  Farsa  Mast3  Atp11c  Cdk8 | web link |
| enzyme binding | Gene Ontology | GO:0019899 | 12 (3.48%) | 437 (2.11%) | 0.0635292089761 | 0.186413762278 | Fn1  Golph3  Calu  Ugt1a6b  Slc4a2  Arrb2  Ugt1a7c  Tmbim6  Atxn10  Hdac10  N4bp2l2  Luc7l2 | web link |
| mRNA binding | Gene Ontology | GO:0003729 | 7 (2.03%) | 211 (1.02%) | 0.0639927840655 | 0.186413762278 | Nup98  Esrp2  Hnrnpk  Hnrnpl  Ptbp2  Snrnp70  Luc7l2 | web link |
| transcription factor binding | Gene Ontology | GO:0008134 | 10 (2.90%) | 349 (1.69%) | 0.0685641499199 | 0.19140825186 | Ywhaz  Mlxipl  Tcf7l2  Mettl23  Hnrnpk  Insig2  Gtf2a2  Mapk9  Bcas3  Yap1 | web link |
| nuclease activity | Gene Ontology | GO:0004518 | 5 (1.45%) | 137 (0.66%) | 0.0795103906626 | 0.213087846976 | Gm49673  Rnaset2b  Dna2  Dclre1c  Aen | web link |
| protein C-terminus binding | Gene Ontology | GO:0008022 | 7 (2.03%) | 227 (1.10%) | 0.0865126750246 | 0.222936508717 | Gm20498  Fn1  Myo1c  Epb41  Abl1  Mpdz  Yap1 | web link |
| methyltransferase activity | Gene Ontology | GO:0008168 | 6 (1.74%) | 187 (0.90%) | 0.0935126026849 | 0.232049791848 | Vcpkmt  Kmt5b  Mettl17  Mettl23  Nsd1  Setd4 | web link |
| histone binding | Gene Ontology | GO:0042393 | 5 (1.45%) | 152 (0.73%) | 0.110922723863 | 0.257089958257 | Smarca2  Chd2  Nap1l4  Tbl1xr1  Bcas3 | web link |
| protein kinase activity | Gene Ontology | GO:0004672 | 13 (3.77%) | 533 (2.58%) | 0.111277743126 | 0.257089958257 | Ulk1  Sgk1  Csnk1g3  Mapk15  Snrk  Cdk8  Mast3  Mapk6  Abl1  Mapkapk5  Mapk9  Clk4  Map4k2 | web link |
| actin filament binding | Gene Ontology | GO:0051015 | 6 (1.74%) | 205 (0.99%) | 0.128897393999 | 0.284662763799 | Neb  Myo1c  Arpc5  Coro1b  Abl1  Lcp1 | web link |
| chromatin binding | Gene Ontology | GO:0003682 | 12 (3.48%) | 499 (2.41%) | 0.131709636981 | 0.284662763799 | Kmt5b  Safb  Tnrc18  Nap1l4  Tcf7l2  Ncapd3  Mapk15  Srebf1  Smarca2  Bcas3  Eya3  Yap1 | web link |
| catalytic activity | Gene Ontology | GO:0003824 | 10 (2.90%) | 405 (1.96%) | 0.141257112112 | 0.290493214793 | Upp2  Papss2  Acacb  Pfkfb2  Enpp3  Mocs1  Dna2  Kynu  Flad1  Echdc1 | web link |
| transcription coactivator activity | Gene Ontology | GO:0003713 | 6 (1.74%) | 213 (1.03%) | 0.146432009855 | 0.290493214793 | Nup98  Ss18  Ccar1  Smarca2  Rbm39  Yap1 | web link |
| lyase activity | Gene Ontology | GO:0016829 | 5 (1.45%) | 167 (0.81%) | 0.147414467209 | 0.290493214793 | Gm49673  Mocs1  Rnaset2b  Naxd  Echdc1 | web link |
| guanyl-nucleotide exchange factor activity | Gene Ontology | GO:0005085 | 6 (1.74%) | 218 (1.05%) | 0.157913920199 | 0.302292361524 | Dock9  Arhgef19  Dennd10  Fgd6  Nucb1  Lamtor2 | web link |
| protein kinase binding | Gene Ontology | GO:0019901 | 12 (3.48%) | 523 (2.53%) | 0.166047454188 | 0.30903276196 | Ywhaz  Ghr  Mlxipl  Rps19  Pfkfb2  Tcf7l2  Prlr  Srebf1  Mapk6  Rhobtb1  Abl1  Dusp3 | web link |
| GTP binding | Gene Ontology | GO:0005525 | 9 (2.61%) | 384 (1.86%) | 0.193419550733 | 0.343029378216 | Gm4951  Rab4b  Rragc  Eif5  Septin2  Agap3  Rhobtb1  Gtpbp8  Mocs1 | web link |
| iron ion binding | Gene Ontology | GO:0005506 | 5 (1.45%) | 185 (0.89%) | 0.196913329023 | 0.343029378216 | Cyp3a59  P4ha1  Aox3  Cyp2d10  Cyp2r1 | web link |
| GTPase activator activity | Gene Ontology | GO:0005096 | 6 (1.74%) | 235 (1.14%) | 0.199673817171 | 0.343029378216 | Ralbp1  Arhgef19  Agap3  Arhgap12  Rabgap1  Tbc1d17 | web link |
| ubiquitin-protein transferase activity | Gene Ontology | GO:0004842 | 6 (1.74%) | 238 (1.15%) | 0.207437817207 | 0.347458343823 | Huwe1  Znrf1  Btrc  Nedd4l  Ube2g2  Rnf214 | web link |
| ubiquitin protein ligase activity | Gene Ontology | GO:0061630 | 7 (2.03%) | 294 (1.42%) | 0.220927016824 | 0.361027076273 | Huwe1  Znrf1  Btrc  Nedd4l  Med7  Fbxo6  Cdk8 | web link |
| protein homodimerization activity | Gene Ontology | GO:0042803 | 14 (4.06%) | 676 (3.27%) | 0.239811293631 | 0.380056118524 | Uba5  Smim1  Aox3  Ghr  Mff  Acox1  Grhpr  H13  Ugt1a6b  Ugt1a7c  Uhrf1bp1l  Gtf2a2  Naa60  Cyp2r1 | web link |
| sequence-specific DNA binding | Gene Ontology | GO:0043565 | 10 (2.90%) | 462 (2.23%) | 0.243916613381 | 0.380056118524 | Hsf2  Foxk2  Mlxipl  Safb  Tcf7l2  Tbx3  Srebf1  Gzf1  Polrmt  Nr1h3 | web link |
| magnesium ion binding | Gene Ontology | GO:0000287 | 5 (1.45%) | 217 (1.05%) | 0.295784905041 | 0.440253331197 | Abl1  Snrk  Mast3  Atp11c  Foxk2 | web link |
| small GTPase binding | Gene Ontology | GO:0031267 | 6 (1.74%) | 271 (1.31%) | 0.298720150941 | 0.440253331197 | Ralbp1  Rabgap1  Dennd10  Rassf5  Myo1c  Ppp6r1 | web link |
| transmembrane transporter activity | Gene Ontology | GO:0022857 | 5 (1.45%) | 219 (1.06%) | 0.30226348112 | 0.440253331197 | Slc35b3  Slco1b2  Slc2a9  Ralbp1  Slc43a1 | web link |
| protein domain specific binding | Gene Ontology | GO:0019904 | 7 (2.03%) | 329 (1.59%) | 0.310062426052 | 0.442003883947 | Ywhaz  Zmynd8  Hax1  Abl1  Hnrnpk  Arrb2  Nde1 | web link |
| DNA binding | Gene Ontology | GO:0003677 | 32 (9.28%) | 1779 (8.60%) | 0.351902646225 | 0.491197443689 | Hsf2  Cers2  Abl1  Zbtb42  Hnrnpk  Crem  Tial1  Rfxap  Foxk2  Mlxipl  Mier2  Huwe1  Safb  Dnajb6  Gzf1  Zfp58  Zbtb43  Nr1h3  Tcf7l2  Tbx3  Chd2  Zfp367  Cenpx  Polrmt  Banp  Dna2  Tbl1xr1  Akap8l  Srebf1  Gmeb2  Nucb1  Lcorl | web link |
| GTPase activity | Gene Ontology | GO:0003924 | 6 (1.74%) | 298 (1.44%) | 0.378155268071 | 0.506995051237 | Gm4951  Rab4b  Rragc  Septin2  Agap3  Rhobtb1 | web link |
| RNA polymerase II cis-regulatory region sequence-specific DNA binding | Gene Ontology | GO:0000978 | 20 (5.80%) | 1101 (5.32%) | 0.378354515848 | 0.506995051237 | Hsf2  Chd2  Foxk2  Nsd1  Mlxipl  Safb  Tcf7l2  Tbx3  Zbtb42  Srebf1  Zfp367  Crem  Zfp874b  Ccar1  Gmeb2  Gzf1  Zfp58  Nr1h3  Zfp235  Yap1 | web link |
| signaling receptor binding | Gene Ontology | GO:0005102 | 7 (2.03%) | 364 (1.76%) | 0.404654559765 | 0.521681738288 | Bag6  Fn1  Gba  Erbin  Myo1c  Itgav  Arrb2 | web link |
| ubiquitin protein ligase binding | Gene Ontology | GO:0031625 | 6 (1.74%) | 307 (1.48%) | 0.404887319268 | 0.521681738288 | Ywhaz  Bag6  H13  Derl1  Arrb2  Tmbim6 | web link |
| metal ion binding | Gene Ontology | GO:0046872 | 61 (17.68%) | 3545 (17.13%) | 0.413749538712 | 0.523041869692 | Pnkd  Aox3  Znrf1  Dna2  Snrk  Rnf44  Uba5  Haghl  Zbtb42  Zbtb43  Hdac10  Lcp1  Adk  Nrp2  Foxk2  Mgat1  Kmt5b  Itgav  Mocs1  Agap3  Gzf1  Zfp874b  Nsd1  Rnf214  Zfp58  Cyp2r1  Nr1h3  Acp5  Fgd6  Zmynd8  Nucb1  Acacb  Prlr  Cyp3a59  Rassf5  Lipc  Zfp938  Calu  P4ha1  Zfp367  Abl1  Gtpbp8  Zyx  Cyp2d10  Zfyve19  Zc3h7a  U2af1  Nudt13  Enpp3  Helz  Smpd4  Fkbp7  Atp11c  Nudt14  Slc25a25  Gmeb2  Mcfd2  Cp  Zfp235  Alkbh3  Eya3 | web link |
| protein-containing complex binding | Gene Ontology | GO:0044877 | 11 (3.19%) | 609 (2.94%) | 0.436244418059 | 0.541266222407 | Ywhaz  Kctd2  Mff  Ugt1a6b  Inca1  Coro1b  Derl1  Ugt1a7c  Arrb2  Srebf1  Nr1h3 | web link |
| protein heterodimerization activity | Gene Ontology | GO:0046982 | 6 (1.74%) | 365 (1.76%) | 0.570665003116 | 0.688802721534 | Rragc  Mlxipl  Ugt1a6b  Ugt1a7c  Mapk6  Gtf2a2 | web link |
| DNA-binding transcription activator activity, RNA polymerase II-specific | Gene Ontology | GO:0001228 | 8 (2.32%) | 491 (2.37%) | 0.575715707551 | 0.688802721534 | Hsf2  Foxk2  Tcf7l2  Srebf1  Gzf1  Gmeb2  Rfxap  Nr1h3 | web link |
| actin binding | Gene Ontology | GO:0003779 | 6 (1.74%) | 384 (1.86%) | 0.620043424973 | 0.728822973213 | Neb  Myo1c  Arpc5  Epb41  Coro1b  Lcp1 | web link |
| RNA polymerase II transcription regulatory region sequence-specific DNA binding | Gene Ontology | GO:0000977 | 6 (1.74%) | 397 (1.92%) | 0.651911265484 | 0.751705778034 | Hsf2  Zfp938  Zbtb43  Crem  Srebf1  Rfxap | web link |
| DNA-binding transcription repressor activity, RNA polymerase II-specific | Gene Ontology | GO:0001227 | 5 (1.45%) | 336 (1.62%) | 0.661949864239 | 0.751705778034 | Mlxipl  Tbx3  Gzf1  Foxk2  Crem | web link |
| ATPase activity | Gene Ontology | GO:0016887 | 5 (1.45%) | 343 (1.66%) | 0.679521789222 | 0.758799331298 | Smarca2  Chd2  Atp11c  Psmc6  Dna2 | web link |
| DNA-binding transcription factor activity | Gene Ontology | GO:0003700 | 9 (2.61%) | 616 (2.98%) | 0.702175198174 | 0.771241611109 | Hsf2  Foxk2  Mlxipl  Tcf7l2  Tbx3  Srebf1  Zfp367  Crem  Nr1h3 | web link |
| sequence-specific double-stranded DNA binding | Gene Ontology | GO:1990837 | 7 (2.03%) | 517 (2.50%) | 0.761789646605 | 0.823224295525 | Hsf2  Tbx3  Abl1  Srebf1  Crem  Gmeb2  Zbtb43 | web link |
| calcium ion binding | Gene Ontology | GO:0005509 | 9 (2.61%) | 709 (3.43%) | 0.839488109394 | 0.892788941737 | Calu  Enpp3  Myl6  Fkbp7  Slc25a25  Mcfd2  Lcp1  Nucb1  Smoc1 | web link |
| zinc ion binding | Gene Ontology | GO:0008270 | 9 (2.61%) | 730 (3.53%) | 0.862410479084 | 0.902835970291 | Zmynd8  Enpp3  Albfm1  Uba5  Sec24c  Sec24b  Nsd1  Hdac10  Nr1h3 | web link |
| peptidase activity | Gene Ontology | GO:0008233 | 6 (1.74%) | 538 (2.60%) | 0.887544433403 | 0.914853492892 | Ctsa  H13  Usp40  Mindy3  Atg4d  Josd2 | web link |
| DNA-binding transcription factor activity, RNA polymerase II-specific | Gene Ontology | GO:0000981 | 12 (3.48%) | 1055 (5.10%) | 0.940442934684 | 0.954692070058 | Foxk2  Mlxipl  Tcf7l2  Tbx3  Zbtb42  Srebf1  Zfp235  Zfp938  Zfp874b  Zfp367  Zfp58  Crem | web link |
| molecular\_function | Gene Ontology | GO:0003674 | 46 (13.33%) | 3542 (17.11%) | 0.977285358002 | 0.977285358002 | C87436  Cuedc2  Ccdc163  Polr1has  Ccdc97  Haghl  Tmem234  Tmem11  Arhgap12  Vps13d  1110065P20Rik  Cdc123  Cdv3  Mrpl30  Fam204a  Tmem256  Ccdc30  4932438A13Rik  Higd1a  Iqcc  Slc39a9  Dcaf11  Yif1b  Ndufs6  Atxn10  Thada  Wsb1  Dmxl1  Slc66a2  Prrc2b  Gmppa  2610528J11Rik  Tns3  Wbp1l  Ggt6  Manf  Txnl4a  Tmem185a  Shld2  Msantd2  Anapc15  Tmem131l  Gm826  Wdr20  Mcfd2  Cox7a2 | web link |
