# Supplementary material for: Regulation of Alternative Splicing of Lipid Metabolism Genes in Sepsis-Induced Liver Damage by RNA-Binding Proteins
Source: Inflammation. 2024 May 9;47(6):1952–68. doi: 10.1007/s10753-024-02017-2 (PMC11607010; doi:10.1007/s10753-024-02017-2)
Supplement: Supplementary file 1 — Supplementary file1 (ZIP 38655 KB) [file 10753_2024_2017_MOESM1_ESM.zip › Sup5_AS_GO.zip!/Sup5_AS_GO/ras/di_RAS/GO_KEGG/CLP_vs_Sham_and_CLPDCA_vs_Sham_di_RAS/CLP_vs_Sham_diUp_nir_GO_analysis/CLP_vs_Sham_diUp_nir_GO_enrichment_P_top.pdf]

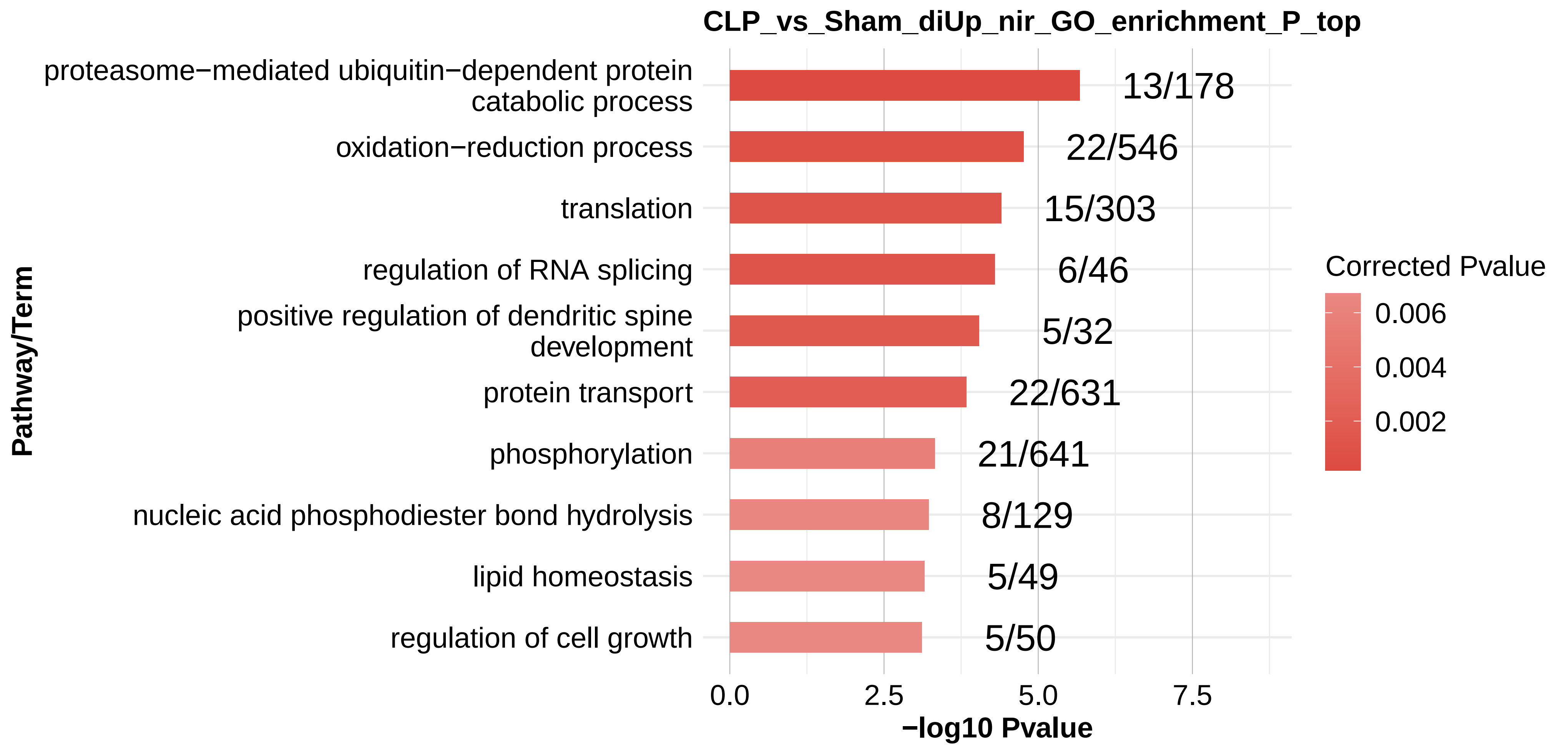
CLP_vs_Sham_diUp_nir_GO_enrichment_P_top
Pathway/Term
proteasome−mediated ubiquitin−dependent protein catabolic process
oxidation−reduction process
translation
regulation of RNA splicing
positive regulation of dendritic spine development
protein transport
phosphorylation
nucleic acid phosphodiester bond hydrolysis
lipid homeostasis
regulation of cell growth
13/178
22/546
15/303
6/46
5/32
22/631
21/641
8/129
5/49
5/50
0.0
2.5
5.0
7.5
−log10 Pvalue
Corrected Pvalue
0.006
0.004
0.002
