# Supplementary material for: Regulation of Alternative Splicing of Lipid Metabolism Genes in Sepsis-Induced Liver Damage by RNA-Binding Proteins
Source: Inflammation. 2024 May 9;47(6):1952–68. doi: 10.1007/s10753-024-02017-2 (PMC11607010; doi:10.1007/s10753-024-02017-2)
Supplement: Supplementary file 1 — Supplementary file1 (ZIP 38655 KB) [file 10753_2024_2017_MOESM1_ESM.zip › Sup5_AS_GO.zip!/Sup5_AS_GO/ras/di_RAS/GO_KEGG/CLP_vs_Sham_and_CLPDCA_vs_Sham_di_RAS/CLP_vs_Sham_diUp_nir_GO_analysis/CLP_vs_Sham_diUp_nir_GO_enrichment_F_top.pdf]

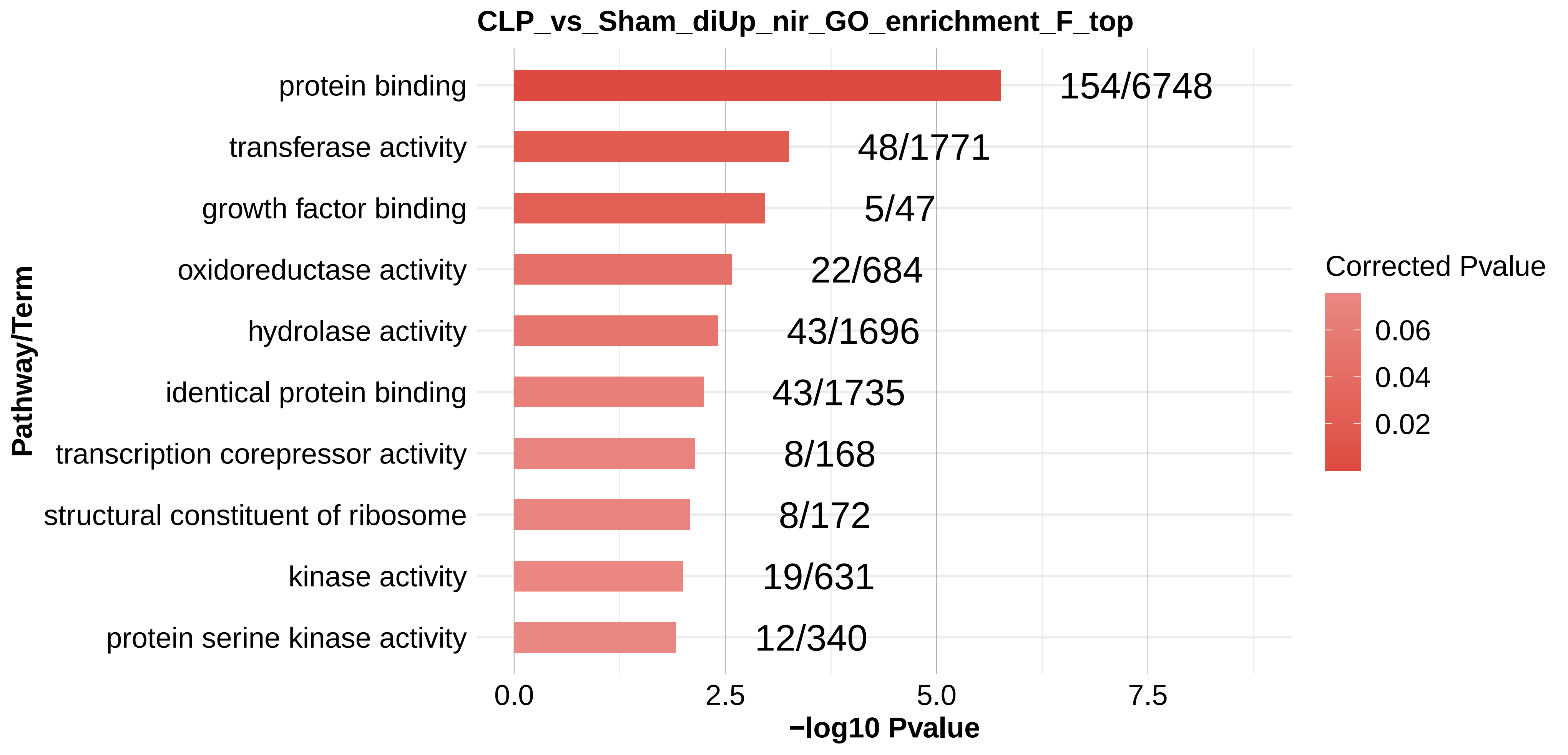

CLP_vs_Sham_diUp_nir_GO_enrichment_F_top
protein binding
transferase activity
growth factor binding
oxidoreductase activity
hydrolase activity
identical protein binding
transcription corepressor activity
structural constituent of ribosome
kinase activity
protein serine kinase activity
154/6748
48/1771
5/47
22/684
43/1696
43/1735
8/168
8/172
19/631
12/340
Pathway/Term
0.0
2.5
5.0
7.5
−log10 Pvalue
Corrected Pvalue
0.06
0.04
0.02
