# Supplementary material for: Regulation of Alternative Splicing of Lipid Metabolism Genes in Sepsis-Induced Liver Damage by RNA-Binding Proteins
Source: Inflammation. 2024 May 9;47(6):1952–68. doi: 10.1007/s10753-024-02017-2 (PMC11607010; doi:10.1007/s10753-024-02017-2)
Supplement: Supplementary file 1 — Supplementary file1 (ZIP 38655 KB) [file 10753_2024_2017_MOESM1_ESM.zip › Sup5_AS_GO.zip!/Sup5_AS_GO/ras/di_RAS/GO_KEGG/CLP_vs_Sham_and_CLPDCA_vs_Sham_di_RAS/CLP_vs_Sham_diDown_nir_GO_analysis/CLP_vs_Sham_diDown_nir_GO_enrichment_C_top.pdf]

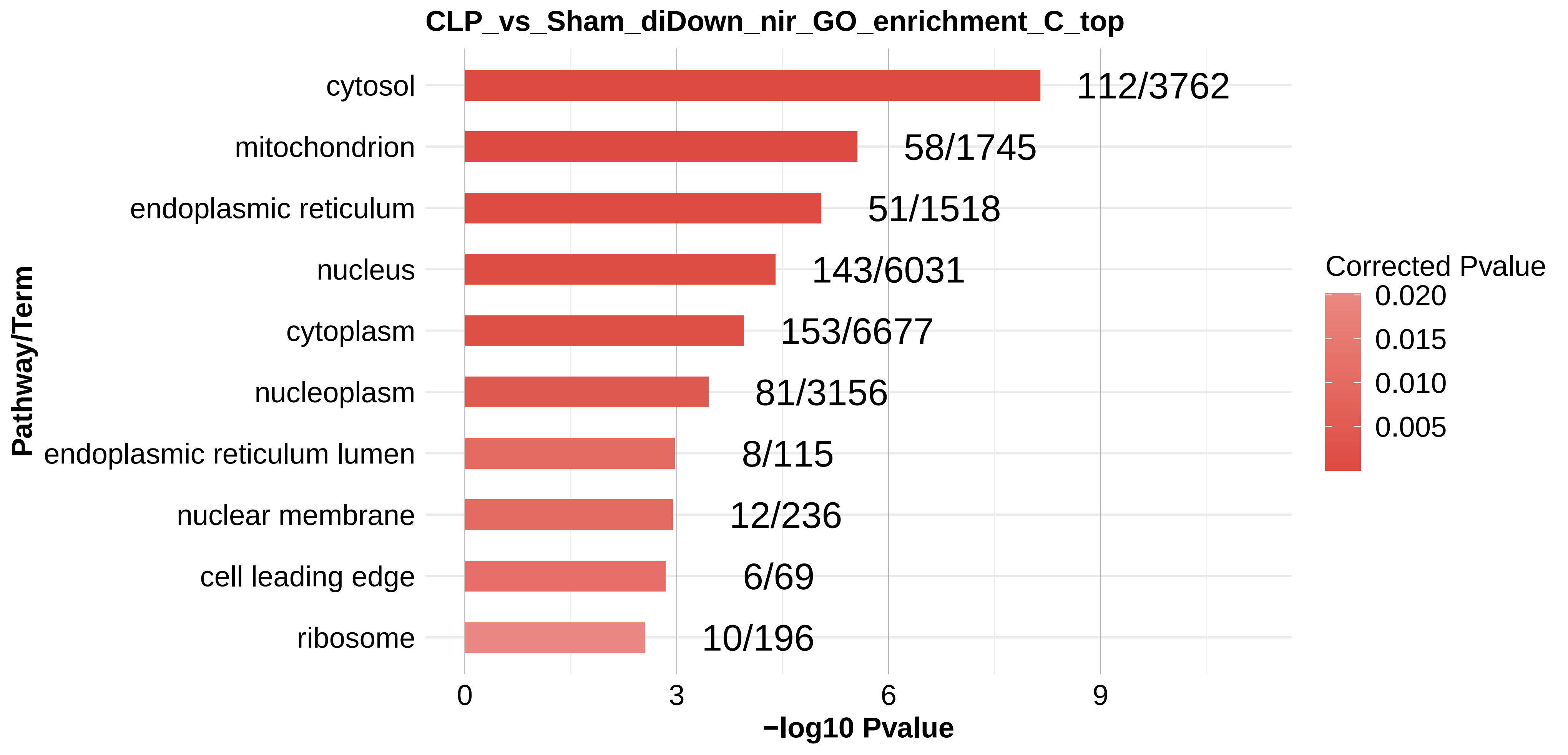
CLP_vs_Sham_diDown_nir_GO_enrichment_C_top
Pathway/Term
cytosol
mitochondrion
endoplasmic reticulum
nucleus
cytoplasm
nucleoplasm
endoplasmic reticulum lumen
nuclear membrane
cell leading edge
ribosome
112/3762
58/1745
51/1518
143/6031
153/6677
81/3156
8/115
12/236
6/69
10/196
0
3
6
9
−log10 Pvalue
Corrected Pvalue
0.020
0.015
0.010
0.005
