# Supplementary material for: Regulation of Alternative Splicing of Lipid Metabolism Genes in Sepsis-Induced Liver Damage by RNA-Binding Proteins
Source: Inflammation. 2024 May 9;47(6):1952–68. doi: 10.1007/s10753-024-02017-2 (PMC11607010; doi:10.1007/s10753-024-02017-2)
Supplement: Supplementary file 1 — Supplementary file1 (ZIP 38655 KB) [file 10753_2024_2017_MOESM1_ESM.zip › Sup5_AS_GO.zip!/Sup5_AS_GO/ras/di_RAS/GO_KEGG/CLP_vs_Sham_and_CLPDCA_vs_Sham_di_RAS/CLPDCA_vs_Sham_diDown_nir_GO_analysis/CLPDCA_vs_Sham_diDown_nir_GO_enrichment_web_C.html]

GO enrichment analyze


# GO enrichment analyze result


| Term | Database | ID | Input number (242) | Background number (20939) | P-Value | Corrected P-Value | Input | Hyperlink |
| --- | --- | --- | --- | --- | --- | --- | --- | --- |
| nucleus | Gene Ontology | GO:0005634 | 102 (42.15%) | 6031 (28.80%) | 5.66449561834e-06 | 0.000283224780917 | Meiob  Hnrnpu  Nf2  Nf1  Ctbp1  Rsl1  Cyren  Ube2j2  Esr1  Irf9  Hnrnpa1  Cdk16  Nup37  Zc3h13  Rnf216  Smarca4  Tardbp  Nfia  Slc11a2  Mars1  Sfmbt1  Pcbp2  Cfh  Cops2  Fubp3  Zfp995  Wdyhv1  Gabpb1  Magi1  Zbtb7c  Deaf1  Plekha1  Knop1  Flnb  Adra1a  Foxk2  Setd4  Ube2d3  Mbd6  Mycbp2  Phf7  Ndrg2  Setdb1  Ghr  Timeless  Psmc6  Luc7l2  Ppp1ca  Mgst1  Eloc  Zfp62  Tsg101  Sbno2  Sun1  Wac  Zkscan1  Mphosph8  Ubap2l  Dtnb  Nlrc5  Nudt21  Ctdsp1  Rnf146  Emsy  Cept1  Nsmf  Eed  Set  Desi1  Clk4  Cggbp1  Fbxo3  Ssb  Srsf2  Tle2  Stk11  Dtnbp1  Tsc1  Csnk1a1  Higd1a  0610010K14Rik  Fgfr3  Uba52  Scaf1  Anp32a  Nsd1  Smarcc2  Carmil1  Zfp932  Dcun1d3  Zfp235  Nabp2  Mms19  Repin1  Dnaja3  Hp1bp3  Zfp467  Birc6  Fam214b  Zfp131  Swi5  Tut4 | web link |
| cytoplasm | Gene Ontology | GO:0005737 | 103 (42.56%) | 6677 (31.89%) | 0.000291440347932 | 0.00728600869831 | Sec22c  Pcmtd1  Gas2l1  Slc22a18  Hnrnpu  Nf2  Hsd3b2  Ctbp1  Asap3  Cyren  Esr1  Fas  Sun1  Rnf181  Rin2  Mtx3  Cyp2r1  Tardbp  Slc11a2  Arl14ep  Bbip1  Mars1  Fam47e  Set  Pcbp2  Cfh  Cops2  Fubp3  Enpp2  Nudt6  Fgfr3  Slmap  G3bp2  Pja1  Pja2  Fnip1  Nf1  Deaf1  Plekha1  Flnb  Adra1a  Foxk2  Setd4  Fpgt  Mycbp2  Baiap2  Ndrg2  Rnf123  Setdb1  Washc1  Ghr  Krt10  Mvk  Psmc6  Wdyhv1  Ngef  Ppp1ca  Kras  Tsg101  Meiob  Cmbl  Hnrnpa1  Gigyf2  Cdk16  Mphosph8  Ubap2l  Rnf149  Dtnb  Bphl  Nlrc5  Magi1  Rnf146  Exoc7  Nsmf  Phldb2  Nudt21  Rnf216  Ahcyl1  Desi1  Pgpep1l  Fbxo6  Ssb  Dctn4  Srsf2  Stk11  Dtnbp1  Tsc1  Csnk1a1  Uba52  Anp32a  Josd2  Ido2  Carmil1  Jmjd4  Dcun1d3  Etfbkmt  Mms19  Dnaja3  Birc6  Gsta3  Cflar  Tut4  Cdc42 | web link |
| nucleoplasm | Gene Ontology | GO:0005654 | 56 (23.14%) | 3156 (15.07%) | 0.000560247954378 | 0.00933746590629 | Gabpb1  Srsf2  Tle2  Stk11  Deaf1  Emsy  Plekha1  Magi1  Ctbp1  Zfp131  Asap3  Adra1a  Mphosph8  Foxk2  Hnrnpa1  Higd1a  Dcaf11  Nup37  0610010K14Rik  Rnf216  Uba52  Mbd6  Zc3h13  Mycbp2  Baiap2  Repin1  Setdb1  Tardbp  Smarcc2  Carmil1  Rubcn  Nfia  Arl14ep  Wac  Timeless  Hnrnpu  Nsmf  Sfmbt1  Pcbp2  Phf7  Eed  Nabp2  Mms19  Luc7l2  Set  Ppp1ca  Cggbp1  Bahd1  Ctdsp1  Wdr27  Fbxo3  Srsf11  Mtif2  Sap18  2610044O15Rik8  Rnf146 | web link |
| mitochondrion | Gene Ontology | GO:0005739 | 34 (14.05%) | 1745 (8.33%) | 0.00184517146792 | 0.0198778094135 | Coa8  Stk11  Atp5mpl  Hsd3b2  Dmac2  Immt  Foxk2  Esr1  Prodh2  Higd1a  Mrps2  Ndufaf4  Mtx3  Bphl  Hccs  Slc11a2  Ghr  Mterf3  Ifi27  Osgepl1  Mars1  Alas2  P4ha1  Etfbkmt  Crls1  Dnaja3  Cfh  Taz  Mrps17  Atp5j  Mgst1  Mtif2  Etfrf1  Kras | web link |
| cytoplasmic ribonucleoprotein granule | Gene Ontology | GO:0036464 | 5 (2.07%) | 77 (0.37%) | 0.00198778094135 | 0.0198778094135 | Cdc42  Gabpb1  Tut4  Ghr  Hnrnpu | web link |
| cytosol | Gene Ontology | GO:0005829 | 61 (25.21%) | 3762 (17.97%) | 0.00288914642706 | 0.0240762202255 | Tsg101  Fnip1  Cmbl  Gigyf2  Srsf2  Baiap2  Stk11  Rubcn  Ghr  Plekha1  Nf2  Eed  Flnb  Tmem87a  Tsc1  Adra1a  Mphosph8  Csnk1a1  Fas  Irf9  Setd4  Cdk16  0610010K14Rik  Rnf216  Uba52  Gak  Phf7  Josd2  Rnf123  Rnf146  Hccs  Carmil1  Washc1  Exoc7  Slc66a2  Arl14ep  Mterf3  Mars1  Pcbp2  Nlrc5  Ngef  Phldb2  Mvk  Nabp2  Mms19  Repin1  Dnaja3  Set  Ahcyl1  Ppp1ca  Pgpep1l  Desi1  Gsta3  Fbxo3  Pnpla6  Sap18  G3bp2  Asap3  Wdyhv1  Tut4  Cdc42 | web link |
| endoplasmic reticulum membrane | Gene Ontology | GO:0005789 | 20 (8.26%) | 903 (4.31%) | 0.00424518122917 | 0.0303227230655 | Sec22c  Porcn  Pja2  Ube2j2  Cyp3a59  Cept1  Mrap  Ncln  Pex16  Pigx  Mgst1  Yif1b  Rdh11  Hsd3b2  Dtnbp1  Derl3  Cdipt  Cyp2r1  Pgap2  Pnpla6 | web link |
| Schaffer collateral - CA1 synapse | Gene Ontology | GO:0098685 | 5 (2.07%) | 104 (0.50%) | 0.0072031852024 | 0.0415333860083 | Baiap2  Dtnbp1  Rnf216  Cdc42  Cadm1 | web link |
| nuclear speck | Gene Ontology | GO:0016607 | 11 (4.55%) | 403 (1.92%) | 0.0074760094815 | 0.0415333860083 | Carmil1  Hp1bp3  Csnk1a1  Srsf2  Wac  Hnrnpu  Zc3h13  Srsf11  Sap18  Phf7  Luc7l2 | web link |
| mitochondrial inner membrane | Gene Ontology | GO:0005743 | 11 (4.55%) | 421 (2.01%) | 0.0101689188545 | 0.0508445942724 | Hccs  Coa8  Prodh2  Higd1a  Taz  Atp5j  Alas2  Mgst1  Hsd3b2  Crls1  Immt | web link |
| postsynaptic density | Gene Ontology | GO:0014069 | 9 (3.72%) | 327 (1.56%) | 0.0141629431206 | 0.0604860496568 | Tsc1  Nsmf  Dtnb  Nf1  Dtnbp1  Baiap2  Pja2  Pcbp2  Cadm1 | web link |
| mitochondrial membrane | Gene Ontology | GO:0031966 | 6 (2.48%) | 174 (0.83%) | 0.0158866420796 | 0.0604860496568 | Higd1a  Taz  Atp5mpl  Ndufaf4  Hsd3b2  Crls1 | web link |
| neuron projection | Gene Ontology | GO:0043005 | 12 (4.96%) | 512 (2.45%) | 0.0164097058219 | 0.0604860496568 | Ppp1ca  Fas  Esr1  Cdk16  Nsmf  Nf2  Nf1  Dtnbp1  Baiap2  Flnb  Cdc42  Cadm1 | web link |
| endoplasmic reticulum | Gene Ontology | GO:0005783 | 27 (11.16%) | 1518 (7.25%) | 0.0169360939039 | 0.0604860496568 | Sec22c  Gigyf2  Cept1  Ncln  Derl3  Hsd3b2  Dtnbp1  Pex16  Porcn  Pgap2  Ube2j2  Poglut1  Yif1b  Anp32a  Pigx  Cyp2r1  Fgfr3  Pnpla6  P4ha1  Cdipt  Set  Ahcyl1  Ces3a  Mrap  Mgst1  Rdh11  Pja2 | web link |
| focal adhesion | Gene Ontology | GO:0005925 | 6 (2.48%) | 184 (0.88%) | 0.0203242724888 | 0.0617114079277 | Arl14ep  Tle2  Phldb2  Flnb  Dctn4  Asap3 | web link |
| intracellular membrane-bounded organelle | Gene Ontology | GO:0043231 | 20 (8.26%) | 1061 (5.07%) | 0.0219998353557 | 0.0617114079277 | Adra1a  Dnaja3  Washc1  Tsc1  Foxk2  Mgst1  Arl14ep  Cyp3a59  Sun1  Rubcn  Mrap  Cyp2r1  P4ha1  Gak  Mycbp2  Hsd3b2  Ahcyl1  Mvk  Setdb1  Asap3 | web link |
| glutamatergic synapse | Gene Ontology | GO:0098978 | 11 (4.55%) | 473 (2.26%) | 0.0221937741419 | 0.0617114079277 | Adra1a  Porcn  Ngef  Ppp1ca  Dtnbp1  Nsmf  Baiap2  Ctbp1  Ndrg2  Rnf216  Cadm1 | web link |
| Z disc | Gene Ontology | GO:0030018 | 5 (2.07%) | 138 (0.66%) | 0.022216106854 | 0.0617114079277 | Slmap  Adra1a  Flnb  Stk11  Neb | web link |
| chromosome | Gene Ontology | GO:0005694 | 12 (4.96%) | 557 (2.66%) | 0.0291422258323 | 0.0766900679797 | Mphosph8  Hp1bp3  Csnk1a1  Meiob  Timeless  Hnrnpu  Nup37  Mbd6  Nsd1  Eed  Setdb1  Repin1 | web link |
| endosome membrane | Gene Ontology | GO:0010008 | 6 (2.48%) | 206 (0.98%) | 0.0328838935277 | 0.0822097338193 | Tsg101  Washc1  Slc11a2  Ube2d3  Kif13a  Dtnbp1 | web link |
| ribonucleoprotein complex | Gene Ontology | GO:1990904 | 5 (2.07%) | 162 (0.77%) | 0.0402494583798 | 0.0958320437615 | G3bp2  Hnrnpa1  Hnrnpu  Csnk1a1  Ssb | web link |
| midbody | Gene Ontology | GO:0030496 | 5 (2.07%) | 166 (0.79%) | 0.0439189216498 | 0.0998157310222 | Kif13a  Birc6  Dtnbp1  Hnrnpu  Cdc42 | web link |
| microtubule organizing center | Gene Ontology | GO:0005815 | 8 (3.31%) | 353 (1.69%) | 0.0534018737401 | 0.11609102987 | Tsg101  Csnk1a1  Exoc7  Birc6  Hnrnpu  Slmap  Dctn4  Cdc42 | web link |
| nuclear membrane | Gene Ontology | GO:0031965 | 6 (2.48%) | 236 (1.13%) | 0.056831474002 | 0.118398904171 | Adra1a  Sun1  Cept1  Nsmf  Rnf123  Repin1 | web link |
| dendritic spine | Gene Ontology | GO:0043197 | 5 (2.07%) | 195 (0.93%) | 0.0763591467426 | 0.152718293485 | Nsmf  Baiap2  Ppp1ca  Cdc42  Dtnbp1 | web link |
| trans-Golgi network | Gene Ontology | GO:0005802 | 5 (2.07%) | 200 (0.96%) | 0.0829809116746 | 0.159578676297 | Rnf215  Golga1  Slc11a2  Slc66a2  Birc6 | web link |
| Golgi apparatus | Gene Ontology | GO:0005794 | 20 (8.26%) | 1309 (6.25%) | 0.123530155782 | 0.228759547745 | Tmbim4  Enpp2  Pgap2  Gigyf2  Esr1  Birc6  Cept1  Golga1  Pkdcc  Gak  Sgms2  Acer2  Naa60  Baiap2  Yif1b  Ndrg2  Pja2  Phf7  Fgfr3  Cdipt | web link |
| protein-containing complex | Gene Ontology | GO:0032991 | 14 (5.79%) | 869 (4.15%) | 0.132781943731 | 0.237110613805 | Set  Nf1  Gigyf2  Esr1  Neb  Stk11  Higd1a  Hnrnpu  Ncln  Desi1  Nf2  Etfbkmt  Cdc42  Tsc1 | web link |
| perinuclear region of cytoplasm | Gene Ontology | GO:0048471 | 12 (4.96%) | 755 (3.61%) | 0.166182038887 | 0.286520756701 | Set  Slc11a2  Esr1  Fas  Golga1  Asgr2  Dcun1d3  Nf2  Anp32a  Ndrg2  Inf2  Tsc1 | web link |
| Golgi membrane | Gene Ontology | GO:0000139 | 8 (3.31%) | 485 (2.32%) | 0.200556393246 | 0.33426065541 | Acer2  Pgap2  Yif1b  Sgms2  Naa60  Tmbim4  Pja2  Cdc42 | web link |
| early endosome | Gene Ontology | GO:0005769 | 5 (2.07%) | 274 (1.31%) | 0.211634728264 | 0.34134633591 | Tsg101  Washc1  Slc11a2  Rubcn  Nf2 | web link |
| nucleolus | Gene Ontology | GO:0005730 | 12 (4.96%) | 813 (3.88%) | 0.232639107637 | 0.359980144607 | Tsg101  Mphosph8  Knop1  Ppp1ca  Arl14ep  Mars1  Nf2  Nf1  Magi1  Smarca4  Tut4  Hsd3b2 | web link |
| endosome | Gene Ontology | GO:0005768 | 10 (4.13%) | 662 (3.16%) | 0.237586895441 | 0.359980144607 | Tsg101  Washc1  Slc11a2  Slc66a2  Gigyf2  Birc6  Ube2d3  Rubcn  Dtnbp1  Rnf215 | web link |
| centrosome | Gene Ontology | GO:0005813 | 8 (3.31%) | 522 (2.49%) | 0.258052832811 | 0.379489460016 | Washc1  Csnk1a1  Hnrnpu  Kif13a  Nlrc5  Nudt21  Dctn4  Cdc42 | web link |
| apical plasma membrane | Gene Ontology | GO:0016324 | 6 (2.48%) | 383 (1.83%) | 0.283067549297 | 0.404382213282 | Slc11a2  Slc22a18  Slc38a3  Abcg8  Fas  Ahcyl1 | web link |
| cytoskeleton | Gene Ontology | GO:0005856 | 17 (7.02%) | 1285 (6.14%) | 0.317433630953 | 0.44088004299 | Tsg101  Slmap  Carmil1  Washc1  Gas2l1  Csnk1a1  Birc6  Tsc1  Dctn4  Hnrnpu  Nsmf  Nf2  Baiap2  Flnb  Cdc42  Mms19  Mycbp2 | web link |
| neuronal cell body | Gene Ontology | GO:0043025 | 8 (3.31%) | 581 (2.77%) | 0.357996013931 | 0.483778397204 | Ghr  Cfh  Ppp1ca  Fas  Dtnbp1  Baiap2  Flnb  Cdc42 | web link |
| synapse | Gene Ontology | GO:0045202 | 10 (4.13%) | 814 (3.89%) | 0.467482792711 | 0.615108937778 | Dnaja3  Ly6e  Cdk16  Nsmf  Uba52  Dtnb  Nf2  Dtnbp1  Pja2  Cadm1 | web link |
| cell projection | Gene Ontology | GO:0042995 | 12 (4.96%) | 1062 (5.07%) | 0.573875773766 | 0.735738171495 | Carmil1  Ngef  Csnk1a1  Nf2  Nsmf  Mycbp2  Magi1  Baiap2  Ndrg2  Cdc42  Fam149b  Tsc1 | web link |
| axon | Gene Ontology | GO:0030424 | 5 (2.07%) | 475 (2.27%) | 0.644903426126 | 0.806129282658 | Nf1  Dtnbp1  Mycbp2  Cfh  Cadm1 | web link |
| cell surface | Gene Ontology | GO:0009986 | 6 (2.48%) | 655 (3.13%) | 0.772083929551 | 0.921162935864 | Slc11a2  Ghr  Fas  Hnrnpu  Atp5j  Fgfr3 | web link |
| membrane | Gene Ontology | GO:0016020 | 96 (39.67%) | 8771 (41.89%) | 0.778671864973 | 0.921162935864 | Sec22c  Acer2  Pcmtd1  Coa8  Slc22a18  Cyp4f13  Nf2  Hsd3b2  Tmbim4  Phldb2  Ube2j2  Esr1  Fas  Sun1  Mtx3  Pigx  Cyp2r1  Hccs  Slc11a2  Abcg8  Cyp3a59  Mars1  Crls1  Ncln  Cdipt  Josd2  Enpp4  Enpp2  Taz  Fgfr3  Slmap  Atp5j  Pja2  Fnip1  Magi1  Adgre5  Cept1  Stra6l  Immt  Tmem87a  Adra1a  Ube2d3  Golga1  Lifr  Baiap2  Mgam  Ly6e  Washc1  Ghr  Ifi27  Nf1  Ngef  Dcun1d3  Kras  Tsg101  Entpd8  Atp5mpl  Derl3  Tmem219  Gm45808  Pex16  Porcn  Wdr59  Rnf149  Sgms2  Stk11  Exoc7  Slc66a2  Nsmf  Rnf215  Sema4a  D11Wsu47e  Sema4g  Ahcyl1  Mrap  Rdh11  Plekha1  Slc22a30  Dtnbp1  Naa60  Cadm1  Pgap2  Slc38a3  Higd1a  Ndufaf4  Asgr2  Mgst1  Yif1b  Tsc1  Carmil1  Adipor2  Gm45713  Dnaja3  Birc6  Pnpla6  Cdc42 | web link |
| cell junction | Gene Ontology | GO:0030054 | 8 (3.31%) | 883 (4.22%) | 0.805081823718 | 0.921162935864 | Dnaja3  Nfia  Cdk16  Nsmf  Magi1  Dtnbp1  Pja2  Cadm1 | web link |
| cytoplasmic vesicle | Gene Ontology | GO:0031410 | 7 (2.89%) | 789 (3.77%) | 0.81062338356 | 0.921162935864 | Washc1  Slc11a2  Sun1  Cdk16  Golga1  Dtnbp1  Fgfr3 | web link |
| integral component of plasma membrane | Gene Ontology | GO:0005887 | 10 (4.13%) | 1203 (5.75%) | 0.894320797832 | 0.981782827823 | Adra1a  Slc11a2  Slc38a3  Ghr  Abcg8  Enpp2  Sgms2  Sema4a  Fgfr3  Sema4g | web link |
| cellular\_component | Gene Ontology | GO:0005575 | 21 (8.68%) | 2346 (11.20%) | 0.916449231703 | 0.981782827823 | Mettl9  Gpank1  Qser1  Fpgt  Plekhg3  9430038I01Rik  Cyp4f13  Tmem219  Kctd18  Fam214a  Rin2  Gucd1  1700066M21Rik  Cyp3a59  Pigx  Proser2  Fam169b  Sbno2  Apol9b  Josd2  Nt5dc1 | web link |
| extracellular space | Gene Ontology | GO:0005615 | 13 (5.37%) | 1732 (8.27%) | 0.967778111257 | 0.981782827823 | Tsku  Cfh  Ghr  Fas  Rnase4  Enpp2  Slmap  Atp5j  Ces3a  Ccl27a  Igfbp4  Sema4a  Sema4g | web link |
| plasma membrane | Gene Ontology | GO:0005886 | 51 (21.07%) | 5547 (26.49%) | 0.978930391423 | 0.981782827823 | Tsg101  Adgre5  Carmil1  Entpd8  Plekha1  Magi1  Dtnbp1  Adipor2  Flnb  Gm45844  Tmem219  Cadm1  Adra1a  Mphosph8  Sgms2  Slc38a3  Esr1  Fas  Cdk16  Ube2d3  Arl14ep  Dtnb  Lifr  Baiap2  Sema4g  Nf2  Fgfr3  Rnf146  Ly6e  Slc11a2  Exoc7  Ghr  Abcg8  Tsku  Nsmf  Dcun1d3  Phf7  Sema4a  Phldb2  Cdipt  Dnaja3  Pja2  Ahcyl1  Cfh  Ppp1ca  Enpp4  Stra6l  Slmap  Mrap  Cdc42  Kras | web link |
| integral component of membrane | Gene Ontology | GO:0016021 | 63 (26.03%) | 6684 (31.92%) | 0.981034664575 | 0.981782827823 | Sec22c  Acer2  Slc22a18  Adgre5  Cyp4f13  Cept1  Entpd8  Atp5mpl  Derl3  Taz  Hsd3b2  Sema4g  Adipor2  Tmem219  Tmbim4  Gm45844  Immt  Tmem87a  Cadm1  Adra1a  Porcn  Gigyf2  Pgap2  Slc66a2  Slc38a3  Fas  Sun1  Higd1a  Pex16  Rnf149  Slc22a30  Asgr2  Yif1b  Sgms2  Rnf215  Josd2  Mgam  Pnpla6  Carmil1  Slc11a2  Ghr  Abcg8  Cyp3a59  Ifi27  Mars1  Pigx  Gm45713  Crls1  Sema4a  D11Wsu47e  Ncln  Cdipt  Cyp2r1  Ube2j2  Stra6l  Enpp4  Enpp2  Mrap  Fgfr3  Slmap  Lifr  Mgst1  Rdh11 | web link |
| extracellular region | Gene Ontology | GO:0005576 | 13 (5.37%) | 1829 (8.73%) | 0.981782827823 | 0.981782827823 | Igfbp4  Tsku  Rnase4  Ghr  Fas  Enpp2  Cfh  Pkdcc  Ccl27a  Lifr  Habp2  Apol9b  1700066M21Rik | web link |
