# Supplementary material for: Regulation of Alternative Splicing of Lipid Metabolism Genes in Sepsis-Induced Liver Damage by RNA-Binding Proteins
Source: Inflammation. 2024 May 9;47(6):1952–68. doi: 10.1007/s10753-024-02017-2 (PMC11607010; doi:10.1007/s10753-024-02017-2)
Supplement: Supplementary file 1 — Supplementary file1 (ZIP 38655 KB) [file 10753_2024_2017_MOESM1_ESM.zip › Sup5_AS_GO.zip!/Sup5_AS_GO/ras/di_RAS/GO_KEGG/CLP_vs_Sham_and_CLPDCA_vs_Sham_di_RAS/CLPDCA_vs_Sham_diDown_nir_GO_analysis/CLPDCA_vs_Sham_diDown_nir_GO_enrichment_web_P.html]

GO enrichment analyze


# GO enrichment analyze result


| Term | Database | ID | Input number (189) | Background number (20961) | P-Value | Corrected P-Value | Input | Hyperlink |
| --- | --- | --- | --- | --- | --- | --- | --- | --- |
| protein ubiquitination | Gene Ontology | GO:0016567 | 16 (8.47%) | 545 (2.60%) | 3.67984992713e-05 | 0.00136430862308 | Pja2  Ube2j2  Birc6  Rnf181  Dcaf11  Ube2d3  Rnf149  Pja1  Uba52  Mycbp2  Fbxo6  Cdc42  Rnf215  Rnf216  Rnf123  Rnf146 | web link |
| regulation of apoptotic process | Gene Ontology | GO:0042981 | 9 (4.76%) | 196 (0.94%) | 7.48023476518e-05 | 0.00136430862308 | Acer2  Dnaja3  Esr1  Fas  Cflar  Nf2  Anp32a  Gm45808  Tardbp | web link |
| chromatin organization | Gene Ontology | GO:0006325 | 11 (5.82%) | 298 (1.42%) | 8.70835291324e-05 | 0.00136430862308 | Smarcc2  Emsy  Wac  Hnrnpu  0610010K14Rik  Sfmbt1  Naa60  Nsd1  Eed  Smarca4  Setdb1 | web link |
| negative regulation of transcription, DNA-templated | Gene Ontology | GO:0045892 | 15 (7.94%) | 622 (2.97%) | 0.000538707370489 | 0.00582319479446 | Smarcc2  Sbno2  Set  Foxk2  Mphosph8  Cops2  Tle2  Timeless  Bahd1  Sfmbt1  Deaf1  Mterf3  Ctbp1  Rsl1  Smarca4 | web link |
| negative regulation of transcription by RNA polymerase II | Gene Ontology | GO:0000122 | 19 (10.05%) | 913 (4.36%) | 0.000619488807921 | 0.00582319479446 | Tsg101  Dnaja3  Zfp932  Foxk2  Fnip1  Esr1  Cops2  Timeless  Ifi27  Deaf1  Hnrnpu  Zfp131  Ctbp1  Eed  2610044O15Rik8  Smarcc2  Rsl1  Cggbp1  Smarca4 | web link |
| negative regulation of cell growth | Gene Ontology | GO:0030308 | 6 (3.17%) | 123 (0.59%) | 0.000881323770489 | 0.00599983453818 | Adipor2  Stk11  Dcun1d3  Nf2  Gas2l1  Smarca4 | web link |
| actin cytoskeleton organization | Gene Ontology | GO:0030036 | 7 (3.70%) | 174 (0.83%) | 0.00103785686213 | 0.00599983453818 | Mtss1  Nf2  Nf1  Flnb  Kras  Inf2  Cdc42 | web link |
| regulation of protein stability | Gene Ontology | GO:0031647 | 5 (2.65%) | 87 (0.42%) | 0.00114890448603 | 0.00599983453818 | Rnf149  Asgr2  Kras  Nf2  Tardbp | web link |
| mitochondrion organization | Gene Ontology | GO:0007005 | 5 (2.65%) | 87 (0.42%) | 0.00114890448603 | 0.00599983453818 | Dnaja3  Gabpb1  Mtx3  Cfh  Taz | web link |
| cell differentiation | Gene Ontology | GO:0030154 | 18 (9.52%) | 918 (4.38%) | 0.00167876667395 | 0.00789020336756 | Tsg101  Sbno2  Sema4g  Stk11  Zfp932  Sun1  Cdk16  Hnrnpu  Sfmbt1  Pkdcc  Ndrg2  Ngef  Sema4a  Ctbp1  Nudt21  Cdc42  Flnb  Cadm1 | web link |
| regulation of cell cycle | Gene Ontology | GO:0051726 | 6 (3.17%) | 145 (0.69%) | 0.00204929638017 | 0.00875608453347 | Gas2l1  Cdk16  Nf2  Ctbp1  Tardbp  Tsc1 | web link |
| cellular response to DNA damage stimulus | Gene Ontology | GO:0006974 | 11 (5.82%) | 470 (2.24%) | 0.003699685616 | 0.0144904353293 | Acer2  Cyren  Timeless  Stk11  Wac  Ube2d3  Emsy  Fbxo6  Swi5  Nabp2  Mms19 | web link |
| nervous system development | Gene Ontology | GO:0007399 | 10 (5.29%) | 410 (1.96%) | 0.00417895435609 | 0.0151085272874 | Smarcc2  Tsku  Ngef  Deaf1  Cdc42  Sema4a  Ndrg2  Sema4g  Smarca4  Tsc1 | web link |
| apoptotic process | Gene Ontology | GO:0006915 | 12 (6.35%) | 567 (2.71%) | 0.00548645009938 | 0.0184187967622 | Dnaja3  Rnf216  Ahcyl1  Coa8  Birc6  Stk11  Ube2d3  Cflar  Fas  Tmem219  Tmbim4  Cadm1 | web link |
| rhythmic process | Gene Ontology | GO:0048511 | 5 (2.65%) | 141 (0.67%) | 0.00911569599893 | 0.02856251413 | Mycbp2  Ppp1ca  Timeless  Hnrnpu  Tardbp | web link |
| RNA splicing | Gene Ontology | GO:0008380 | 7 (3.70%) | 272 (1.30%) | 0.0119124703283 | 0.0349928815895 | Srsf2  Hnrnpa1  Hnrnpu  Zc3h13  Srsf11  Scaf1  Tardbp | web link |
| DNA repair | Gene Ontology | GO:0006281 | 8 (4.23%) | 352 (1.68%) | 0.0148068151162 | 0.0404008048986 | Emsy  Timeless  Cyren  Ube2d3  Fbxo6  Swi5  Nabp2  Mms19 | web link |
| regulation of transcription, DNA-templated | Gene Ontology | GO:0006355 | 21 (11.11%) | 1403 (6.69%) | 0.0154726486846 | 0.0404008048986 | Sbno2  Emsy  Zfp932  Foxk2  2010315B03Rik  Esr1  Fubp3  Irf9  Zfp467  Nfia  Zkscan1  Sfmbt1  Zfp235  Gm14403  Mterf3  Tle2  Hp1bp3  2610044O15Rik8  Smarca4  Zfp995  Rsl1 | web link |
| endocytosis | Gene Ontology | GO:0006897 | 6 (3.17%) | 226 (1.08%) | 0.0168594727225 | 0.0417050114714 | Ghr  Enpp2  Rubcn  Rin2  Asgr2  Cdc42 | web link |
| translation | Gene Ontology | GO:0006412 | 7 (3.70%) | 303 (1.45%) | 0.0203251879446 | 0.0477641916698 | Eif4g3  Mrps2  Mars1  Mrps17  Uba52  Gm45713  Mtif2 | web link |
| methylation | Gene Ontology | GO:0032259 | 5 (2.65%) | 184 (0.88%) | 0.025839589248 | 0.0547589825502 | Nsd1  Pcmtd1  Setd4  Etfbkmt  Setdb1 | web link |
| negative regulation of cell population proliferation | Gene Ontology | GO:0008285 | 8 (4.23%) | 392 (1.87%) | 0.0261934096427 | 0.0547589825502 | Tsg101  Dnaja3  Zbtb7c  Stk11  Nudt6  Nf2  Nf1  Tsc1 | web link |
| regulation of transcription by RNA polymerase II | Gene Ontology | GO:0006357 | 21 (11.11%) | 1489 (7.10%) | 0.027711567103 | 0.0547589825502 | Smarcc2  Gm14403  Nfia  Cggbp1  Zfp932  Foxk2  2010315B03Rik  Esr1  Irf9  Zfp467  Deaf1  Zfp131  Zkscan1  Zfp62  Zfp235  Ctdsp1  2610044O15Rik8  Rsl1  Zfp995  Zbtb7c  Repin1 | web link |
| ubiquitin-dependent protein catabolic process | Gene Ontology | GO:0006511 | 6 (3.17%) | 256 (1.22%) | 0.0288794693249 | 0.0547589825502 | Birc6  Ube2d3  Rnf149  Eloc  Rnf215  Rnf146 | web link |
| regulation of cell population proliferation | Gene Ontology | GO:0042127 | 5 (2.65%) | 190 (0.91%) | 0.0291271183778 | 0.0547589825502 | Birc6  Hp1bp3  Nf2  Nf1  Fas | web link |
| immune system process | Gene Ontology | GO:0002376 | 9 (4.76%) | 481 (2.29%) | 0.0308162894895 | 0.0557063694618 | Ido2  Cfh  Irf9  Rubcn  Sema4a  Pcbp2  G3bp2  Nlrc5  Cadm1 | web link |
| Wnt signaling pathway | Gene Ontology | GO:0016055 | 5 (2.65%) | 205 (0.98%) | 0.0384643996341 | 0.0669565475113 | Tle2  Ndrg2  Rnf146  Csnk1a1  Porcn | web link |
| mRNA processing | Gene Ontology | GO:0006397 | 7 (3.70%) | 351 (1.67%) | 0.0403672356113 | 0.0677592883475 | Srsf2  Hnrnpa1  Hnrnpu  Zc3h13  Scaf1  Nudt21  Tardbp | web link |
| cell division | Gene Ontology | GO:0051301 | 7 (3.70%) | 365 (1.74%) | 0.0480257396844 | 0.0769137822329 | Tsg101  Csnk1a1  Ppp1ca  Birc6  Timeless  Hnrnpu  Nup37 | web link |
| positive regulation of transcription, DNA-templated | Gene Ontology | GO:0045893 | 11 (5.82%) | 691 (3.30%) | 0.0490939035529 | 0.0769137822329 | Tsg101  Smarcc2  Nfia  Foxk2  Esr1  Fubp3  Deaf1  Wac  Nsd1  Smarca4  Mms19 | web link |
| regulation of gene expression | Gene Ontology | GO:0010468 | 7 (3.70%) | 376 (1.79%) | 0.054653500032 | 0.082861758113 | Tsku  Fas  Cggbp1  Pcbp2  Nf1  Fubp3  Tardbp | web link |
| lipid metabolic process | Gene Ontology | GO:0006629 | 9 (4.76%) | 544 (2.60%) | 0.0587257555748 | 0.0862534535005 | Acer2  Adipor2  Sgms2  Enpp2  Cept1  Pnpla6  Crls1  Mvk  Cdipt | web link |
| positive regulation of protein phosphorylation | Gene Ontology | GO:0001934 | 5 (2.65%) | 235 (1.12%) | 0.06209234499 | 0.0884345519554 | Kras  Fnip1  Dtnbp1  Adipor2  Fas | web link |
| positive regulation of transcription by RNA polymerase II | Gene Ontology | GO:0045944 | 15 (7.94%) | 1201 (5.73%) | 0.126284388663 | 0.174569596093 | Sbno2  Nfia  Gabpb1  Foxk2  Zbtb7c  Esr1  Fubp3  Zfp467  Deaf1  Hnrnpu  Zfp131  Pcbp2  Nlrc5  Smarca4  Zfp62 | web link |
| negative regulation of apoptotic process | Gene Ontology | GO:0043066 | 8 (4.23%) | 570 (2.72%) | 0.144561631276 | 0.194125619142 | Dnaja3  Ndufaf4  Fas  Higd1a  Cflar  Birc6  Tmbim4  Smarca4 | web link |
| positive regulation of cell population proliferation | Gene Ontology | GO:0008284 | 8 (4.23%) | 590 (2.81%) | 0.165103823412 | 0.215552213898 | Acer2  Birc6  Enpp2  Ndufaf4  Lifr  Smarca4  Fgfr3  Kras | web link |
| cell cycle | Gene Ontology | GO:0007049 | 8 (4.23%) | 595 (2.84%) | 0.170442494424 | 0.216508033457 | Tsg101  Csnk1a1  Ppp1ca  Birc6  Stk11  Timeless  Hnrnpu  Nup37 | web link |
| protein phosphorylation | Gene Ontology | GO:0006468 | 8 (4.23%) | 616 (2.94%) | 0.193699112761 | 0.239575218415 | Csnk1a1  Cops2  Stk11  Cdk16  Pkdcc  Gak  Clk4  Fgfr3 | web link |
| phosphorylation | Gene Ontology | GO:0016310 | 8 (4.23%) | 641 (3.06%) | 0.223003680021 | 0.260994483327 | Csnk1a1  Stk11  Cdk16  Pkdcc  Sgms2  Mvk  Clk4  Fgfr3 | web link |
| oxidation-reduction process | Gene Ontology | GO:0055114 | 7 (3.70%) | 546 (2.60%) | 0.223874240671 | 0.260994483327 | Cyp4f13  P4ha1  Rdh11  Hsd3b2  Cyp3a59  Ctbp1  Cyp2r1 | web link |
| multicellular organism development | Gene Ontology | GO:0007275 | 12 (6.35%) | 1040 (4.96%) | 0.229876264636 | 0.260994483327 | Tsku  Ngef  Timeless  Deaf1  Hnrnpu  Poglut1  Pkdcc  Zc3h13  Sema4a  Ndrg2  Sema4g  Cadm1 | web link |
| spermatogenesis | Gene Ontology | GO:0007283 | 6 (3.17%) | 458 (2.19%) | 0.23322911276 | 0.260994483327 | Stk11  Sun1  Cdk16  Sfmbt1  Plekha1  Cadm1 | web link |
| biological\_process | Gene Ontology | GO:0008150 | 28 (14.81%) | 2834 (13.52%) | 0.331099896061 | 0.361899886392 | Gpank1  9430038I01Rik  Ccdc97  Cmbl  Atp5mpl  1700066M21Rik  Tmem219  Fam169b  Apol9b  Snx29  0610010K14Rik  Golga1  Gucd1  Phf7  Proser2  Mbd6  Fpgt  Arl14ep  Kctd18  Fam47e  D11Wsu47e  Mettl9  Qser1  Fam214a  Fam214b  Wdr27  Srsf11  Sap18 | web link |
| protein transport | Gene Ontology | GO:0015031 | 7 (3.70%) | 631 (3.01%) | 0.342945628772 | 0.366328285279 | Tsg101  Sec22c  Exoc7  Nup37  Pkdcc  Mtx3  Yif1b | web link |
| proteolysis | Gene Ontology | GO:0006508 | 6 (3.17%) | 664 (3.17%) | 0.555519337844 | 0.580209086193 | Desi1  Pgpep1l  Ncln  Cflar  Habp2  Josd2 | web link |
| innate immune response | Gene Ontology | GO:0045087 | 5 (2.65%) | 560 (2.67%) | 0.571415095959 | 0.583837163263 | Nlrc5  Pja2  Pcbp2  Cfh  G3bp2 | web link |
| signal transduction | Gene Ontology | GO:0007165 | 11 (5.82%) | 2811 (13.41%) | 0.999781431689 | 0.999781431689 | Adra1a  Nf1  Adgre5  Fas  Rin2  Gm45713  Ccl27a  Magi1  Ndrg2  Gm45844  Kras | web link |
