# Supplementary material for: Regulation of Alternative Splicing of Lipid Metabolism Genes in Sepsis-Induced Liver Damage by RNA-Binding Proteins
Source: Inflammation. 2024 May 9;47(6):1952–68. doi: 10.1007/s10753-024-02017-2 (PMC11607010; doi:10.1007/s10753-024-02017-2)
Supplement: Supplementary file 1 — Supplementary file1 (ZIP 38655 KB) [file 10753_2024_2017_MOESM1_ESM.zip › Sup5_AS_GO.zip!/Sup5_AS_GO/ras/di_RAS/GO_KEGG/CLP_vs_Sham_and_CLPDCA_vs_Sham_di_RAS/CLPDCA_vs_Sham_diDown_nir_GO_analysis/CLPDCA_vs_Sham_diDown_nir_GO_enrichment_web_F.html]

GO enrichment analyze


# GO enrichment analyze result


| Term | Database | ID | Input number (235) | Background number (20698) | P-Value | Corrected P-Value | Input | Hyperlink |
| --- | --- | --- | --- | --- | --- | --- | --- | --- |
| chromatin binding | Gene Ontology | GO:0003682 | 18 (7.66%) | 499 (2.41%) | 1.72620975765e-05 | 0.000690483903059 | Smarcc2  Meiob  Nfia  Set  Zfp932  Esr1  Setdb1  Wac  Hnrnpu  Bahd1  Sfmbt1  Mphosph8  Mbd6  Ctbp1  Eed  Nudt21  Smarca4  Hp1bp3 | web link |
| protein binding | Gene Ontology | GO:0005515 | 105 (44.68%) | 6748 (32.60%) | 6.82293693746e-05 | 0.00136458738749 | Gpank1  Gas2l1  Sbno2  Rubcn  Nf2  Nf1  Eed  Ctbp1  Asap3  Esr1  Fas  Irf9  Sun1  Cdk16  Dcaf11  Nup37  Smarca4  Tardbp  Hccs  Abcg8  Hnrnpu  Sfmbt1  Pcbp2  Cfh  Cops2  Stra6l  Slmap  Pja1  Pja2  Fnip1  Gabpb1  Magi1  Zbtb7c  Neb  Zfp932  Flnb  Immt  Smarcc2  Trafd1  Foxk2  Setd4  Ube2d3  Golga1  Lifr  Baiap2  Rnf123  Setdb1  Ghr  Porcn  Timeless  Mycbp2  Ngef  Ppp1ca  Eloc  Kras  Tsg101  Meiob  Gigyf2  Wac  Kif13a  Mphosph8  Ubap2l  Wdr59  Rnf149  Poglut1  Dtnb  Nlrc5  Nudt21  Ctdsp1  Rnf146  Emsy  Exoc7  Eif4g3  Deaf1  Nsmf  P4ha1  Sema4a  Sema4g  Ahcyl1  Desi1  Fbxo3  Fbxo6  Srsf2  Tle2  Stk11  Igfbp4  Dtnbp1  Gsta3  Cadm1  Wdr27  Uba52  Anp32a  Nsd1  Tsc1  Carmil1  Adipor2  Dnaja3  Tsku  Zfp467  Birc6  Zfp131  Cflar  Swi5  Tut4  Cdc42 | web link |
| transferase activity | Gene Ontology | GO:0016740 | 36 (15.32%) | 1771 (8.56%) | 0.000446560669375 | 0.00595414225833 | Pcmtd1  Rnf181  Stk11  Cept1  Pkdcc  Naa60  Clk4  Porcn  Csnk1a1  Ube2j2  Cdk16  Ube2d3  Rnf149  Poglut1  Fpgt  Mycbp2  Nsd1  Rnf216  Rnf123  Fgfr3  Tut4  Sgms2  Osgepl1  Alas2  Etfbkmt  Crls1  Mvk  Cdipt  Setdb1  Pja2  Birc6  Gsta3  Mgst1  Pja1  Rnf146  Setd4 | web link |
| single-stranded DNA binding | Gene Ontology | GO:0003697 | 6 (2.55%) | 103 (0.50%) | 0.00114888264264 | 0.0114888264264 | Meiob  Fubp3  Hnrnpa1  Hnrnpu  Pcbp2  Nabp2 | web link |
| ubiquitin protein ligase activity | Gene Ontology | GO:0061630 | 10 (4.26%) | 294 (1.42%) | 0.00203354048451 | 0.0162683238761 | Rnf181  Ube2d3  Rnf149  Pja1  Mycbp2  Fbxo6  Cdc42  Rnf215  Rnf216  Rnf146 | web link |
| transcription corepressor activity | Gene Ontology | GO:0003714 | 7 (2.98%) | 168 (0.81%) | 0.00313701954041 | 0.0209134636027 | Tsg101  Cops2  Tle2  Hnrnpu  Sfmbt1  Ctbp1  Smarca4 | web link |
| protein-containing complex binding | Gene Ontology | GO:0044877 | 15 (6.38%) | 609 (2.94%) | 0.00424432627882 | 0.0242532930218 | Tsg101  Dnaja3  Carmil1  Cflar  Esr1  Fas  Stk11  Hnrnpu  Ppp1ca  Atp5j  Derl3  Eloc  Tsc1  Smarca4  Kras | web link |
| metal ion binding | Gene Ontology | GO:0046872 | 56 (23.83%) | 3545 (17.13%) | 0.00522683297393 | 0.0261341648697 | Acer2  Tdo2  Bpnt1  Zbtb7c  Cyp4f13  Rnf44  Cept1  Entpd8  Rnf146  Deaf1  Adipor2  Zfp131  Rsl1  Zkscan1  Asap3  Trafd1  2010315B03Rik  Esr1  Rnf181  Rnf149  Zc3h13  Dtnb  Mycbp2  Rnf215  Phf7  Cyp2r1  Rnf123  Ctdsp1  Tut4  Gm14403  Ido2  Hccs  Stk11  Zfp932  Jmjd4  Abcg8  Cyp3a59  Osgepl1  Nsd1  P4ha1  Zfp235  Mvk  Repin1  Foxk2  Dnaja3  Rnf216  Zfp467  Enpp4  Ppp1ca  Enpp2  Zfp995  Pja1  2610044O15Rik8  Pja2  Zfp62  Nt5dc1 | web link |
| RNA polymerase II cis-regulatory region sequence-specific DNA binding | Gene Ontology | GO:0000978 | 22 (9.36%) | 1101 (5.32%) | 0.00735037708924 | 0.029807463562 | Zbtb7c  Hnrnpu  Zkscan1  Rsl1  Foxk2  Esr1  Irf9  Gm14403  Nsd1  Nlrc5  Smarca4  Zfp995  Tardbp  Nfia  Zfp932  2010315B03Rik  Zfp235  Eed  Zfp467  Zfp131  2610044O15Rik8  Zfp62 | web link |
| histone binding | Gene Ontology | GO:0042393 | 6 (2.55%) | 152 (0.73%) | 0.00788895760863 | 0.029807463562 | Smarcc2  Sbno2  Set  Sfmbt1  Anp32a  Smarca4 | web link |
| nucleic acid binding | Gene Ontology | GO:0003676 | 14 (5.96%) | 594 (2.87%) | 0.00819705247956 | 0.029807463562 | Gpank1  Rnase4  Srsf2  Fubp3  Hnrnpa1  Enpp2  Snhg4  Pcbp2  G3bp2  Srsf11  Tardbp  Nabp2  Tut4  Ssb | web link |
| mRNA binding | Gene Ontology | GO:0003729 | 7 (2.98%) | 211 (1.02%) | 0.0105778760251 | 0.0352595867504 | Fubp3  Gm45713  Pcbp2  G3bp2  Nudt21  Mvk  Luc7l2 | web link |
| heme binding | Gene Ontology | GO:0020037 | 6 (2.55%) | 183 (0.88%) | 0.018355185232 | 0.0564774930216 | Ido2  Hccs  Tdo2  Cyp4f13  Cyp3a59  Cyp2r1 | web link |
| identical protein binding | Gene Ontology | GO:0042802 | 29 (12.34%) | 1735 (8.38%) | 0.0229908787289 | 0.0656882249398 | Tdo2  Hnrnpu  Ctbp1  Flnb  Esr1  Fas  0610010K14Rik  Mgst1  Mycbp2  Baiap2  Nudt21  Fgfr3  Tardbp  Emsy  Adipor2  Ghr  P4ha1  Eed  Mvk  Psmc6  Ahcyl1  Cfh  Desi1  Cggbp1  Mrap  Mtss1  Srsf11  Cdc42  Kras | web link |
| RNA binding | Gene Ontology | GO:0003723 | 17 (7.23%) | 915 (4.42%) | 0.0323136778536 | 0.0861698076097 | Ahcyl1  Srsf2  Eif4g3  Fubp3  Hnrnpa1  Mars1  Hnrnpu  Mrps17  Snhg4  Pcbp2  Scaf1  G3bp2  Srsf11  Nudt21  Tardbp  Smarca4  Ssb | web link |
| transferase activity, transferring acyl groups | Gene Ontology | GO:0016746 | 5 (2.13%) | 170 (0.82%) | 0.0448683838419 | 0.112170959605 | Porcn  Alas2  Osgepl1  Taz  Naa60 | web link |
| ubiquitin-protein transferase activity | Gene Ontology | GO:0004842 | 6 (2.55%) | 238 (1.15%) | 0.05478908529 | 0.1289154948 | Birc6  Rnf181  Ube2d3  Pja2  Rnf123  Rnf146 | web link |
| iron ion binding | Gene Ontology | GO:0005506 | 5 (2.13%) | 185 (0.89%) | 0.0602489478938 | 0.131579642899 | Cyp4f13  P4ha1  Cyp2r1  Slc11a2  Cyp3a59 | web link |
| methyltransferase activity | Gene Ontology | GO:0008168 | 5 (2.13%) | 187 (0.90%) | 0.0625003303771 | 0.131579642899 | Nsd1  Pcmtd1  Setd4  Etfbkmt  Setdb1 | web link |
| hydrolase activity | Gene Ontology | GO:0016787 | 26 (11.06%) | 1696 (8.19%) | 0.0721002683562 | 0.144200536712 | Acer2  Meiob  Cmbl  Bpnt1  Entpd8  Bphl  Nudt21  Josd2  Smarca4  Ctdsp1  Rnase4  Mgam  Cdc42  Psmc6  Wdyhv1  Ces3a  Ppp1ca  Enpp4  Desi1  Enpp2  Nudt6  Pnpla6  Habp2  Nt5dc1  Pgpep1l  Kras | web link |
| magnesium ion binding | Gene Ontology | GO:0000287 | 5 (2.13%) | 217 (1.05%) | 0.101819981456 | 0.193942821821 | Stk11  Bpnt1  Foxk2  Mvk  Csnk1a1 | web link |
| ubiquitin protein ligase binding | Gene Ontology | GO:0031625 | 6 (2.55%) | 307 (1.48%) | 0.137847613143 | 0.250632023897 | Tsg101  Washc1  Slc22a18  Ube2j2  Uba52  Pcbp2 | web link |
| actin binding | Gene Ontology | GO:0003779 | 7 (2.98%) | 384 (1.86%) | 0.148383101878 | 0.258057568484 | Washc1  Neb  Hnrnpu  Mtss1  Nf2  Flnb  Inf2 | web link |
| oxidoreductase activity | Gene Ontology | GO:0016491 | 11 (4.68%) | 684 (3.30%) | 0.156848995127 | 0.261414991878 | Ido2  Tdo2  Jmjd4  Cyp3a59  Prodh2  P4ha1  Rdh11  Hsd3b2  Cyp4f13  Ctbp1  Cyp2r1 | web link |
| ATP binding | Gene Ontology | GO:0005524 | 21 (8.94%) | 1484 (7.17%) | 0.17497514125 | 0.275108150059 | Ube2d3  Hnrnpu  Csnk1a1  Ube2j2  Dnaja3  Abcg8  Smarca4  Stk11  Cdk16  Mars1  Entpd8  Kif13a  Pkdcc  Gak  Kifc2  Magi1  Nlrc5  Mvk  Clk4  Fgfr3  Psmc6 | web link |
| catalytic activity | Gene Ontology | GO:0003824 | 7 (2.98%) | 405 (1.96%) | 0.178820297538 | 0.275108150059 | Fahd2a  Auh  Enpp4  Enpp2  Alas2  Hsd3b2  Mgam | web link |
| DNA binding | Gene Ontology | GO:0003677 | 24 (10.21%) | 1779 (8.60%) | 0.215961750245 | 0.319943333696 | Meiob  Deaf1  Hnrnpu  Zkscan1  Foxk2  Esr1  Irf9  0610010K14Rik  Mbd6  Dtnb  Smarca4  Setdb1  Tardbp  Nfia  Mterf3  Timeless  Pcbp2  Nabp2  Repin1  Set  Hp1bp3  Zfp467  Cggbp1  Zfp131 | web link |
| nucleotide binding | Gene Ontology | GO:0000166 | 23 (9.79%) | 1795 (8.67%) | 0.302305061635 | 0.431864373764 | Stk11  Hnrnpu  Entpd8  Kif13a  Pkdcc  Magi1  Csnk1a1  Ube2j2  Cdk16  Ube2d3  Fpgt  Nlrc5  Smarca4  Fgfr3  Abcg8  Mars1  Mvk  Psmc6  Clk4  Mtif2  Cdc42  Kifc2  Kras | web link |
| zinc ion binding | Gene Ontology | GO:0008270 | 10 (4.26%) | 730 (3.53%) | 0.3175013471 | 0.437932892552 | Trafd1  Esr1  Enpp2  Tut4  Dtnb  Mycbp2  Nsd1  Slc11a2  Setdb1  Rnf146 | web link |
| transcription factor binding | Gene Ontology | GO:0008134 | 5 (2.13%) | 349 (1.69%) | 0.363698644055 | 0.484931525407 | Ctbp1  Nfia  Dnaja3  Smarca4  Esr1 | web link |
| protein kinase activity | Gene Ontology | GO:0004672 | 7 (2.98%) | 533 (2.58%) | 0.402098907131 | 0.518837299524 | Csnk1a1  Stk11  Cdk16  Pkdcc  Gak  Clk4  Fgfr3 | web link |
| kinase activity | Gene Ontology | GO:0016301 | 8 (3.40%) | 631 (3.05%) | 0.426223244594 | 0.532779055743 | Csnk1a1  Stk11  Cdk16  Pkdcc  Sgms2  Mvk  Clk4  Fgfr3 | web link |
| DNA-binding transcription activator activity, RNA polymerase II-specific | Gene Ontology | GO:0001228 | 6 (2.55%) | 491 (2.37%) | 0.485553275238 | 0.58854942453 | Nfia  Foxk2  Esr1  Zfp467  Zfp131  Zfp62 | web link |
| DNA-binding transcription factor activity, RNA polymerase II-specific | Gene Ontology | GO:0000981 | 12 (5.11%) | 1055 (5.10%) | 0.539473807008 | 0.617241488099 | Nfia  Zfp932  Foxk2  Zbtb7c  Irf9  Deaf1  Zfp131  Zkscan1  Zfp235  Gm14403  Rsl1  Zfp995 | web link |
| molecular\_function | Gene Ontology | GO:0003674 | 40 (17.02%) | 3542 (17.11%) | 0.542218663992 | 0.617241488099 | Cmbl  Coa8  9430038I01Rik  Ccdc97  Ncln  Atp5mpl  Wdr59  Tmem219  Tmbim4  Fam149b  Cyren  Fam169b  Ubap2l  Higd1a  Nup37  Rin2  Ndufaf4  Dcaf11  Gucd1  Yif1b  Pigx  Mtx3  Proser2  Fpgt  Slc66a2  Mettl9  Arl14ep  Bbip1  Kctd18  Fam47e  Dcun1d3  Trappc13  D11Wsu47e  Snx29  Qser1  Fam214a  Fam214b  Wdr27  Etfrf1  1700066M21Rik | web link |
| enzyme binding | Gene Ontology | GO:0019899 | 5 (2.13%) | 437 (2.11%) | 0.555517339289 | 0.617241488099 | Fnip1  Esr1  Pcbp2  Mms19  Luc7l2 | web link |
| sequence-specific DNA binding | Gene Ontology | GO:0043565 | 5 (2.13%) | 462 (2.23%) | 0.604982242929 | 0.654034857221 | Foxk2  Esr1  Rsl1  Hnrnpu  Zfp131 | web link |
| protein homodimerization activity | Gene Ontology | GO:0042803 | 7 (2.98%) | 676 (3.27%) | 0.650290416642 | 0.684516228044 | Tsg101  Ghr  Naa60  Ctbp1  Nudt21  Cyp2r1  Cadm1 | web link |
| DNA-binding transcription factor activity | Gene Ontology | GO:0003700 | 5 (2.13%) | 616 (2.98%) | 0.832120174586 | 0.853456589319 | Nfia  Irf9  Foxk2  2010315B03Rik  Esr1 | web link |
| calcium ion binding | Gene Ontology | GO:0005509 | 5 (2.13%) | 709 (3.43%) | 0.908343893077 | 0.908343893077 | Adgre5  Ppp2r3a  Enpp2  Habp2  Ppp2r3d | web link |
