# Supplementary material for: Regulation of Alternative Splicing of Lipid Metabolism Genes in Sepsis-Induced Liver Damage by RNA-Binding Proteins
Source: Inflammation. 2024 May 9;47(6):1952–68. doi: 10.1007/s10753-024-02017-2 (PMC11607010; doi:10.1007/s10753-024-02017-2)
Supplement: Supplementary file 1 — Supplementary file1 (ZIP 38655 KB) [file 10753_2024_2017_MOESM1_ESM.zip › Sup5_AS_GO.zip!/Sup5_AS_GO/ras/di_RAS/GO_KEGG/CLP_vs_Sham_and_CLPDCA_vs_Sham_di_RAS/CLPDCA_vs_Sham_diDown_nir_GO_analysis/CLPDCA_vs_Sham_diDown_nir_GO_enrichment_C_top.pdf]

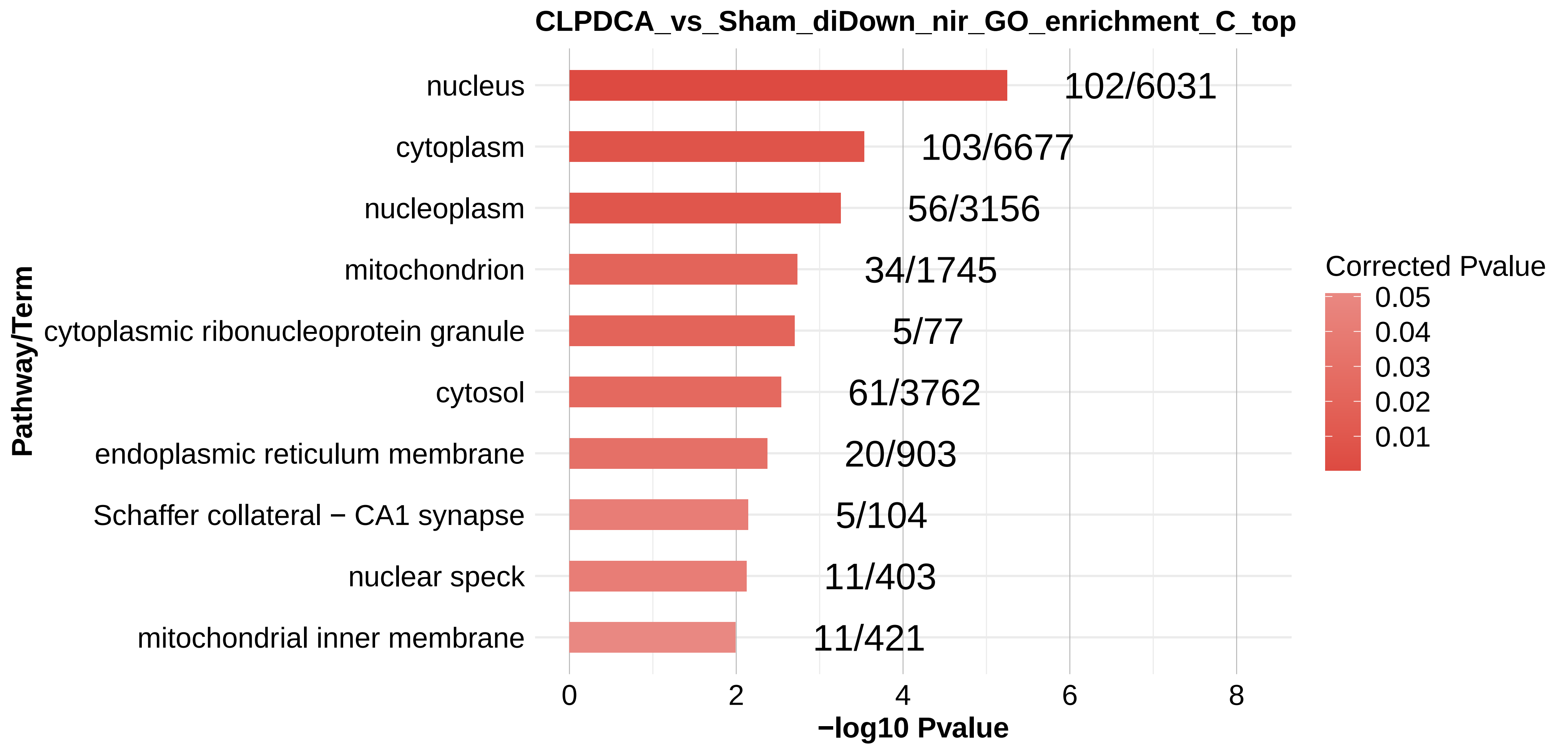

CLPDCA_vs_Sham_diDown_nir_GO_enrichment_C_top
nucleus
cytoplasm
nucleoplasm
mitochondrion
cytoplasmic ribonucleoprotein granule
cytosol
endoplasmic reticulum membrane
Schaffer collateral − CA1 synapse
nuclear speck
mitochondrial inner membrane
102/6031
103/6677
56/3156
34/1745
5/77
61/3762
20/903
5/104
11/403
11/421
Pathway/Term
0
2
4
6
8
−log10 Pvalue
Corrected Pvalue
0.05
0.04
0.03
0.02
0.01
