# Supplementary material for: Regulation of Alternative Splicing of Lipid Metabolism Genes in Sepsis-Induced Liver Damage by RNA-Binding Proteins
Source: Inflammation. 2024 May 9;47(6):1952–68. doi: 10.1007/s10753-024-02017-2 (PMC11607010; doi:10.1007/s10753-024-02017-2)
Supplement: Supplementary file 1 — Supplementary file1 (ZIP 38655 KB) [file 10753_2024_2017_MOESM1_ESM.zip › Sup5_AS_GO.zip!/Sup5_AS_GO/ras/di_RAS/GO_KEGG/CLP_vs_Sham_and_CLPDCA_vs_Sham_di_RAS/CLPDCA_vs_Sham_diDown_nir_GO_analysis/CLPDCA_vs_Sham_diDown_nir_GO_enrichment_F_top.pdf]

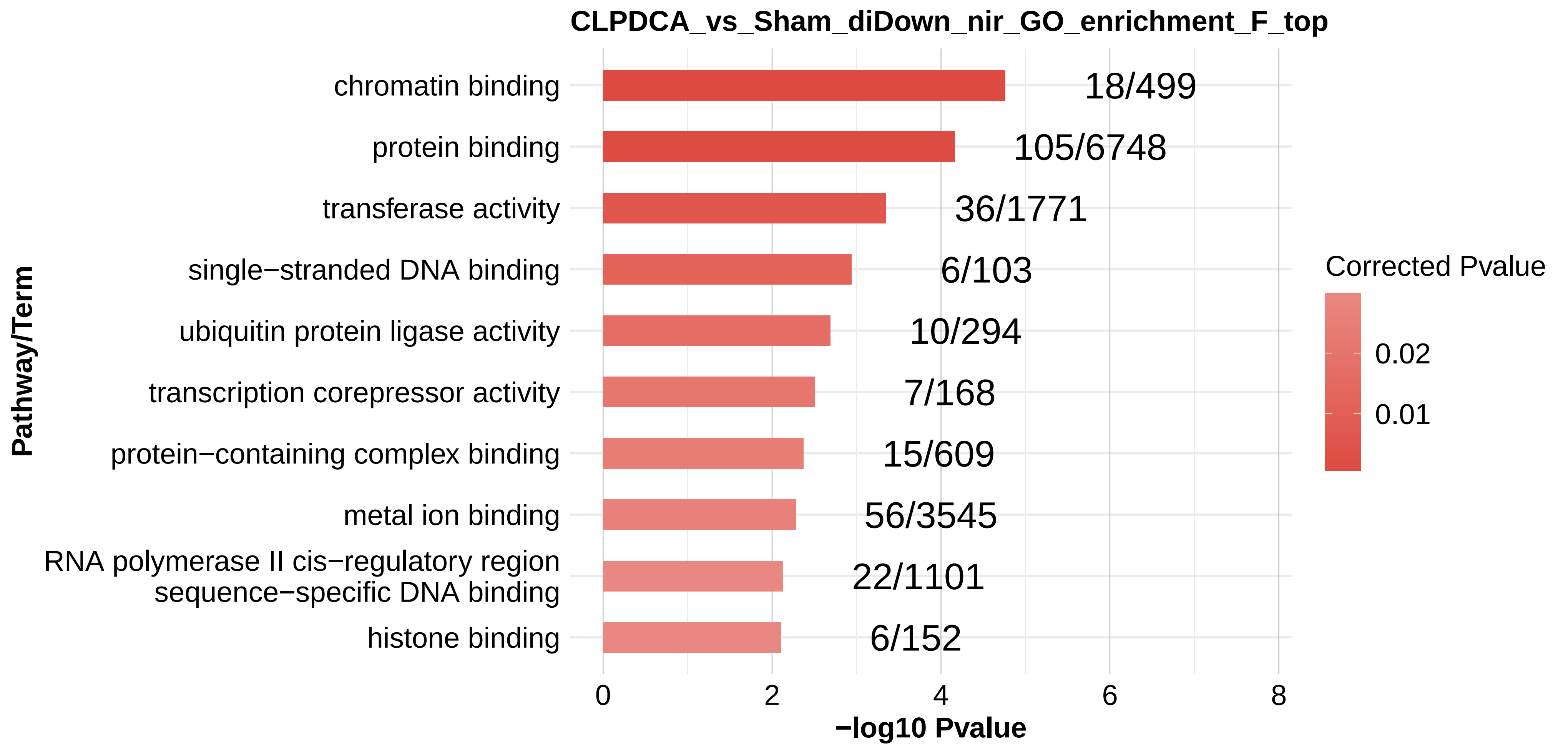
CLPDCA_vs_Sham_diDown_nir_GO_enrichment_F_top
Pathway/Term
chromatin binding
protein binding
transferase activity
single−stranded DNA binding
ubiquitin protein ligase activity
transcription corepressor activity
protein−containing complex binding
metal ion binding
RNA polymerase II cis−regulatory region sequence−specific DNA binding
histone binding
18/499
105/6748
36/1771
6/103
10/294
7/168
15/609
56/3545
22/1101
6/152
0
2
4
6
8
−log10 Pvalue
Corrected Pvalue
0.02
0.01
