# Supplementary material for: Regulation of Alternative Splicing of Lipid Metabolism Genes in Sepsis-Induced Liver Damage by RNA-Binding Proteins
Source: Inflammation. 2024 May 9;47(6):1952–68. doi: 10.1007/s10753-024-02017-2 (PMC11607010; doi:10.1007/s10753-024-02017-2)
Supplement: Supplementary file 1 — Supplementary file1 (ZIP 38655 KB) [file 10753_2024_2017_MOESM1_ESM.zip › Sup5_AS_GO.zip!/Sup5_AS_GO/ras/di_RAS/GO_KEGG/CLP_vs_Sham_and_CLPDCA_vs_Sham_di_RAS/CLPDCA_vs_Sham_diUp_nir_GO_analysis/CLPDCA_vs_Sham_diUp_nir_GO_enrichment_F_top.pdf]

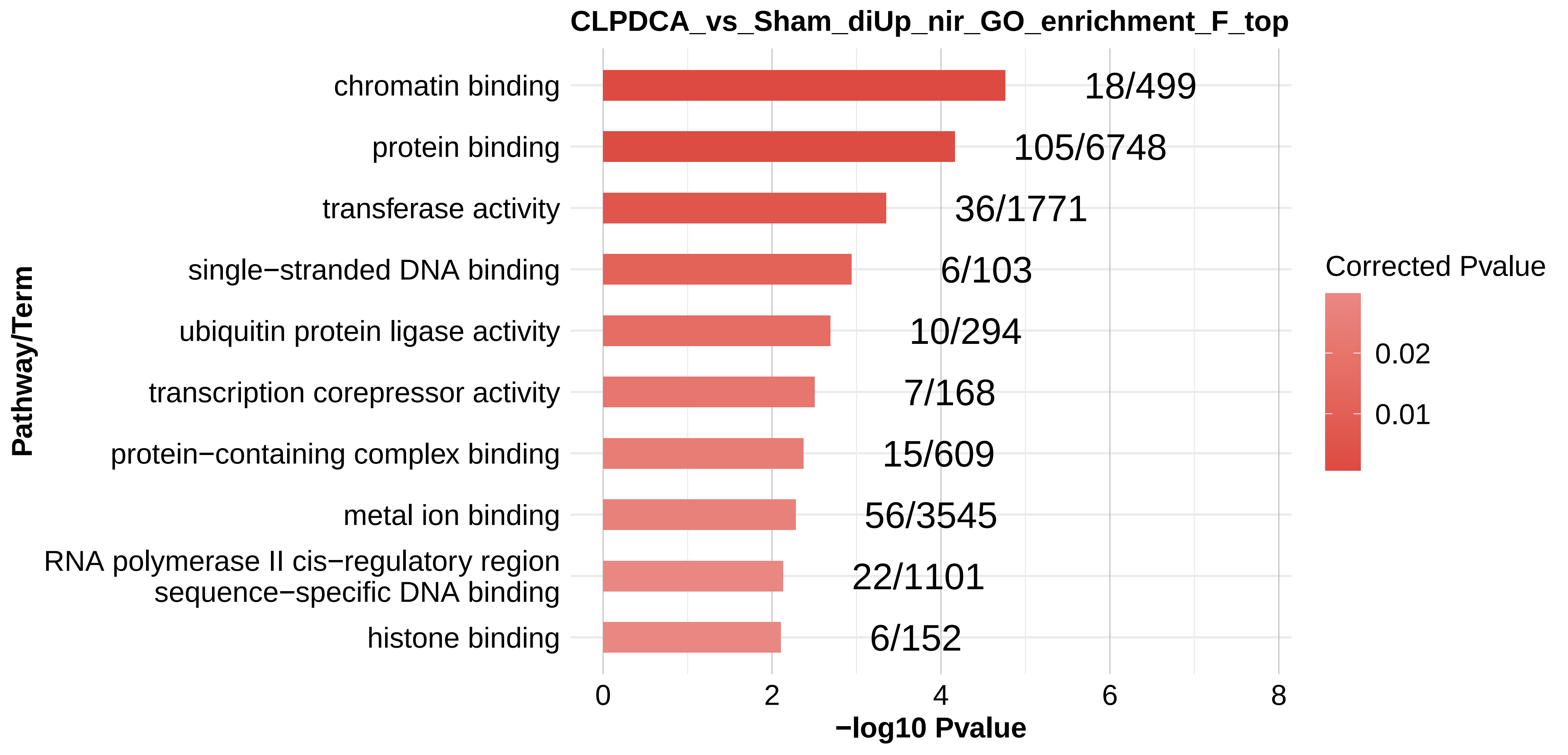

CLPDCA_vs_Sham_diUp_nir_GO_enrichment_F_top
chromatin binding
18/499
protein binding
105/6748
transferase activity
36/1771
single−stranded DNA binding
6/103
ubiquitin protein ligase activity
10/294
transcription corepressor activity
7/168
protein−containing complex binding
15/609
metal ion binding
56/3545
RNA polymerase II cis−regulatory region sequence−specific DNA binding
22/1101
histone binding
6/152
Pathway/Term
0
2
4
6
8
−log10 Pvalue
Corrected Pvalue
0.02
0.01
