# Supplementary material for: Regulation of Alternative Splicing of Lipid Metabolism Genes in Sepsis-Induced Liver Damage by RNA-Binding Proteins
Source: Inflammation. 2024 May 9;47(6):1952–68. doi: 10.1007/s10753-024-02017-2 (PMC11607010; doi:10.1007/s10753-024-02017-2)
Supplement: Supplementary file 1 — Supplementary file1 (ZIP 38655 KB) [file 10753_2024_2017_MOESM1_ESM.zip › Sup5_AS_GO.zip!/Sup5_AS_GO/ras/di_RAS/GO_KEGG/CLP_vs_Sham_and_CLPDCA_vs_Sham_di_RAS/CLPDCA_vs_Sham_diUp_nir_GO_analysis/CLPDCA_vs_Sham_diUp_nir_GO_enrichment_P_top.pdf]

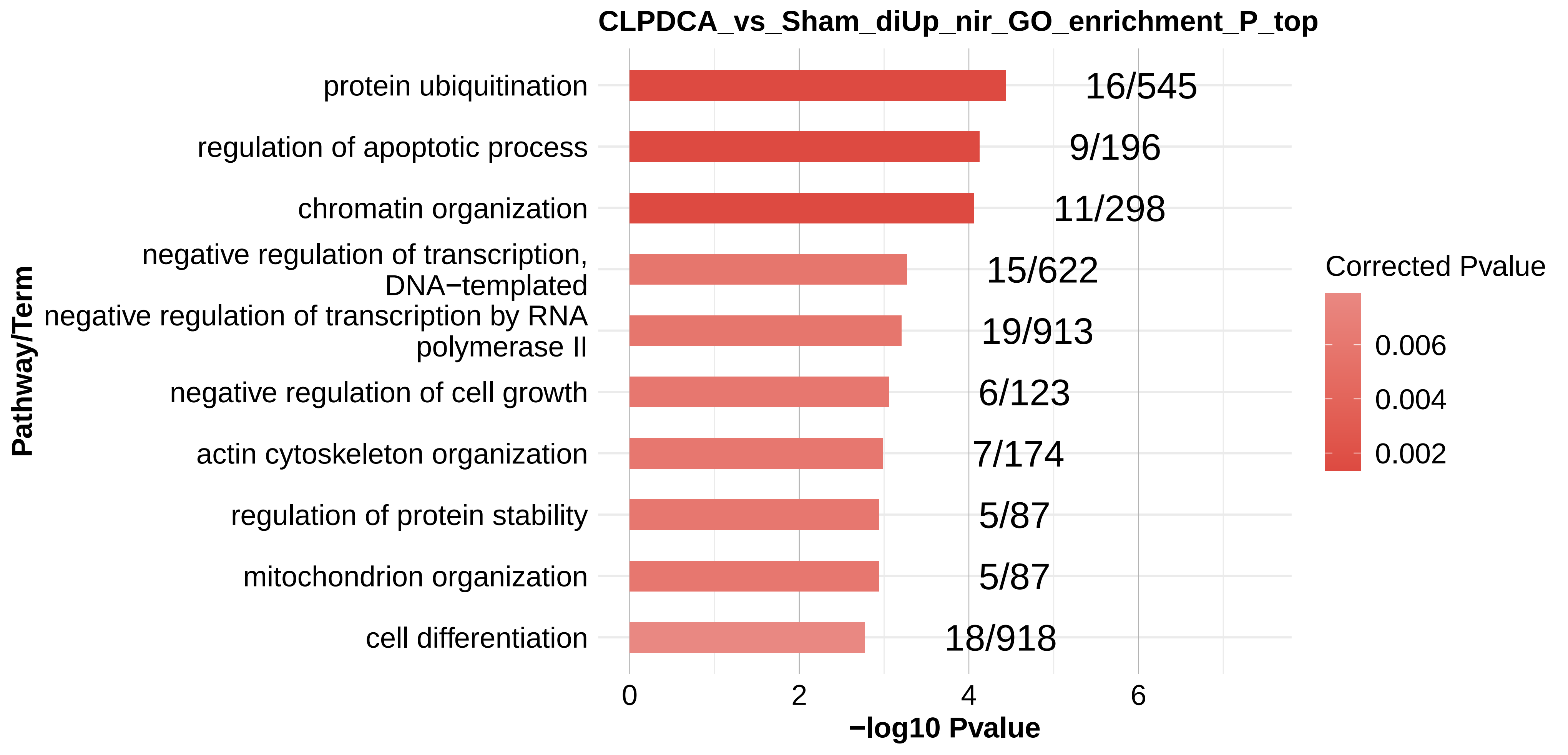
CLPDCA_vs_Sham_diUp_nir_GO_enrichment_P_top
protein ubiquitination
regulation of apoptotic process
chromatin organization
negative regulation of transcription, DNA−templated
negative regulation of transcription by RNA polymerase II
negative regulation of cell growth
actin cytoskeleton organization
regulation of protein stability
mitochondrion organization
cell differentiation
16/545
9/196
11/298
15/622
19/913
6/123
7/174
5/87
5/87
18/918
Pathway/Term
0
2
4
6
−log10 Pvalue
Corrected Pvalue
0.006
0.004
0.002
