# Supplementary material for: Regulation of Alternative Splicing of Lipid Metabolism Genes in Sepsis-Induced Liver Damage by RNA-Binding Proteins
Source: Inflammation. 2024 May 9;47(6):1952–68. doi: 10.1007/s10753-024-02017-2 (PMC11607010; doi:10.1007/s10753-024-02017-2)
Supplement: Supplementary file 1 — Supplementary file1 (ZIP 38655 KB) [file 10753_2024_2017_MOESM1_ESM.zip › Sup5_AS_GO.zip!/Sup5_AS_GO/ras/co_RAS/GO_KEGG/CLP_vs_Sham_and_CLPDCA_vs_Sham_co_RAS/coDown_nir_GO_analysis/coDown_nir_GO_enrichment_web_C.html]

GO enrichment analyze


# GO enrichment analyze result


| Term | Database | ID | Input number (95) | Background number (20939) | P-Value | Corrected P-Value | Input | Hyperlink |
| --- | --- | --- | --- | --- | --- | --- | --- | --- |
| mitochondrion | Gene Ontology | GO:0005739 | 21 (22.11%) | 1745 (8.33%) | 2.87814070987e-05 | 0.000604409549073 | Mrpl36  Smurf1  Card19  Ociad2  Bri3bp  Mff  Mthfs  Esr1  Ccdc58  Apoo  Snap23  Rpl35a  Taz  Ssbp1  Adhfe1  Mpv17l2  Clu  Mmaa  Slc25a44  Mtfr1l  Cln8 | web link |
| mitochondrial membrane | Gene Ontology | GO:0031966 | 5 (5.26%) | 174 (0.83%) | 0.0011858805835 | 0.0124517461268 | Clu  Card19  Slc25a44  Mff  Taz | web link |
| endoplasmic reticulum membrane | Gene Ontology | GO:0005789 | 10 (10.53%) | 903 (4.31%) | 0.00781361061086 | 0.0441110483958 | Card19  Apoo  Asph  Calu  Sc5d  Elovl5  Tmem208  Gba2  Eif5a  Cln8 | web link |
| endoplasmic reticulum | Gene Ontology | GO:0005783 | 14 (14.74%) | 1518 (7.25%) | 0.00840210445635 | 0.0441110483958 | Card19  Ces3a  Tmem258  Apoo  Asph  Calu  Sc5d  Elovl5  Tmem208  Clu  Gba2  Cln8  Proc  Eif5a | web link |
| mitochondrial inner membrane | Gene Ontology | GO:0005743 | 6 (6.32%) | 421 (2.01%) | 0.0123543451425 | 0.0518882495987 | Ociad2  Slc25a44  Apoo  Taz  Mpv17l2  Clu | web link |
| Golgi apparatus | Gene Ontology | GO:0005794 | 12 (12.63%) | 1309 (6.25%) | 0.0151998416582 | 0.0531994458036 | Pmf1  Esr1  Apoo  Paqr3  St3gal4  Calu  Uhmk1  Tcp1  Gak  Gfra1  Gba2  Proc | web link |
| cytosol | Gene Ontology | GO:0005829 | 23 (24.21%) | 3762 (17.97%) | 0.0765229233662 | 0.201404104643 | Apoo  Ifi30  Akr1e1  Coro1b  Raph1  Fbp1  Clu  Lcp1  Bin1  Exoc7  Tcp1  Gak  Thoc7  Madd  Slc40a1  Mthfs  Gstm4  Akr1c6  Smurf1  Fam160a2  Rpl35a  Gba2  Ncor1 | web link |
| synapse | Gene Ontology | GO:0045202 | 7 (7.37%) | 814 (3.89%) | 0.0767253731975 | 0.201404104643 | Mff  Snap23  Slc12a7  Prkar1a  Clu  Bin1  Eif5a | web link |
| perinuclear region of cytoplasm | Gene Ontology | GO:0048471 | 6 (6.32%) | 755 (3.61%) | 0.128490770494 | 0.299811797819 | Mtmr14  Esr1  Cep43  Coro1b  Clu  Lcp1 | web link |
| cytoplasm | Gene Ontology | GO:0005737 | 35 (36.84%) | 6677 (31.89%) | 0.17616161216 | 0.361042719523 | Snap23  Asph  Akr1e1  Coro1b  Vegfa  Fbp1  Mmaa  Clu  Lcp1  Bin1  Mpv17l2  Mtmr14  Esr1  Paox  Tcp1  Mthfs  Thoc7  Madd  Exoc7  Gstm4  Gmppa  Uhmk1  Cep43  Ablim2  Eapp  Dido1  Smurf1  Uxt  Ints7  Rps6kb2  Cflar  Prkar1a  Sult2a8  Akr1c6  Eif5a | web link |
| protein-containing complex | Gene Ontology | GO:0032991 | 6 (6.32%) | 869 (4.15%) | 0.202072104392 | 0.361042719523 | Mff  Tmem258  Esr1  Slc12a7  Prkar1a  Clu | web link |
| intracellular membrane-bounded organelle | Gene Ontology | GO:0043231 | 7 (7.37%) | 1061 (5.07%) | 0.206310125442 | 0.361042719523 | Pmf1  Calu  Ifi30  Sc5d  Gak  Tmem208  Clu | web link |
| cytoskeleton | Gene Ontology | GO:0005856 | 8 (8.42%) | 1285 (6.14%) | 0.227525565294 | 0.367541297782 | Uxt  Cep43  Coro1b  Tcp1  Clu  Lcp1  Bin1  Dido1 | web link |
| extracellular region | Gene Ontology | GO:0005576 | 10 (10.53%) | 1829 (8.73%) | 0.316455817043 | 0.474683725565 | Apoo  Calu  Ifi30  Angptl4  Vegfa  Lifr  AI182371  Clu  Proc  Fetub | web link |
| cellular\_component | Gene Ontology | GO:0005575 | 12 (12.63%) | 2346 (11.20%) | 0.375424311843 | 0.525594036581 | L3hypdh  Plekhg3  Szrd1  Gmppa  Haghl  Fam135a  Smim10l1  Wbp1l  Zfand4  Tmem222  Cyb561d1  Btf3l4 | web link |
| membrane | Gene Ontology | GO:0016020 | 41 (43.16%) | 8771 (41.89%) | 0.439387608316 | 0.576696235915 | Slc25a44  Snap23  Apoo  Asph  Dhrs3  Slc17a2  Cyb561d1  Sc5d  Vegfa  Tmem176b  Clu  Lcp1  Bin1  Exoc7  Card19  Tmem258  Lifr  Madd  Bri3bp  Slc40a1  St3gal4  Mthfs  Tmem222  Phkb  Slc12a7  Wbp1l  Tmem208  Smurf1  Mff  Calu  Paqr3  Esr1  Taz  Elovl5  Prkar1a  Mpv17l2  AI182371  Gfra1  Gba2  Eif5a  Cln8 | web link |
| extracellular space | Gene Ontology | GO:0005615 | 7 (7.37%) | 1732 (8.27%) | 0.679698225915 | 0.816844134421 | Ces3a  Angptl4  Gfra1  Fetub  Vegfa  Clu  Proc | web link |
| integral component of membrane | Gene Ontology | GO:0016021 | 28 (29.47%) | 6684 (31.92%) | 0.730632297434 | 0.816844134421 | Slc25a44  Apoo  Asph  Dhrs3  Slc17a2  Sc5d  Tmem176b  Cyb561d1  Card19  Tmem258  Lifr  Madd  Bri3bp  Slc40a1  St3gal4  Mthfs  Tmem222  Slc12a7  Wbp1l  Tmem208  Mff  Paqr3  Taz  Elovl5  Mpv17l2  AI182371  Gba2  Cln8 | web link |
| nucleus | Gene Ontology | GO:0005634 | 25 (26.32%) | 6031 (28.80%) | 0.739049454952 | 0.816844134421 | Rtca  Vegfa  Fbp1  Tmem176b  Ptbp3  Clu  Bin1  Kdm5a  Pmf1  Esr1  Safb  Thoc7  Emsy  Uhmk1  Cep43  Eapp  Dido1  Uxt  Ints7  Ddx56  Rps6kb2  Akr1c6  Ncor2  Ncor1  Eif5a | web link |
| nucleoplasm | Gene Ontology | GO:0005654 | 11 (11.58%) | 3156 (15.07%) | 0.865870926379 | 0.909164472697 | Emsy  Slc40a1  Pmf1  Snap23  Rtca  Safb  Uhmk1  Srsf11  Ncor1  Ncor2  Dido1 | web link |
| plasma membrane | Gene Ontology | GO:0005886 | 17 (17.89%) | 5547 (26.49%) | 0.981665016529 | 0.981665016529 | Smurf1  Madd  Exoc7  Esr1  Slc40a1  Snap23  Asph  Phkb  Coro1b  Raph1  Slc12a7  Vegfa  Prkar1a  Gfra1  Lcp1  Bin1  Lifr | web link |
