# Supplementary material for: Regulation of Alternative Splicing of Lipid Metabolism Genes in Sepsis-Induced Liver Damage by RNA-Binding Proteins
Source: Inflammation. 2024 May 9;47(6):1952–68. doi: 10.1007/s10753-024-02017-2 (PMC11607010; doi:10.1007/s10753-024-02017-2)
Supplement: Supplementary file 1 — Supplementary file1 (ZIP 38655 KB) [file 10753_2024_2017_MOESM1_ESM.zip › Sup5_AS_GO.zip!/Sup5_AS_GO/ras/co_RAS/GO_KEGG/CLP_vs_Sham_and_CLPDCA_vs_Sham_co_RAS/coDown_nir_GO_analysis/coDown_nir_GO_enrichment_web_F.html]

GO enrichment analyze


# GO enrichment analyze result


| Term | Database | ID | Input number (87) | Background number (20698) | P-Value | Corrected P-Value | Input | Hyperlink |
| --- | --- | --- | --- | --- | --- | --- | --- | --- |
| oxidoreductase activity | Gene Ontology | GO:0016491 | 11 (12.64%) | 684 (3.30%) | 0.000135469657311 | 0.00203204485967 | Paox  Asph  Dhrs3  Ifi30  Akr1e1  Sc5d  Adhfe1  Aldh16a1  Cyb561d1  Akr1c6  Kdm5a | web link |
| identical protein binding | Gene Ontology | GO:0042802 | 14 (16.09%) | 1735 (8.38%) | 0.0131301805331 | 0.0699331478088 | Emsy  Slc40a1  Mff  Esr1  Tysnd1  Coro1b  Mmaa  Angptl4  Vegfa  Fbp1  Ssbp1  Srsf11  Lcp1  Bin1 | web link |
| protein-containing complex binding | Gene Ontology | GO:0044877 | 7 (8.05%) | 609 (2.94%) | 0.0141989720206 | 0.0699331478088 | Mff  Esr1  Coro1b  Cflar  Clu  Bin1  Ncor2 | web link |
| chromatin binding | Gene Ontology | GO:0003682 | 6 (6.90%) | 499 (2.41%) | 0.0186488394157 | 0.0699331478088 | Uxt  Esr1  Safb  Ncor2  Ssbp1  Kdm5a | web link |
| hydrolase activity | Gene Ontology | GO:0016787 | 12 (13.79%) | 1696 (8.19%) | 0.0511042519638 | 0.153312755891 | Ntpcr  Mtmr14  Ces3a  Hagh  Tysnd1  Ddx56  Haghl  Rnpepl1  Fbp1  Mmaa  Gba2  Proc | web link |
| RNA binding | Gene Ontology | GO:0003723 | 7 (8.05%) | 915 (4.42%) | 0.0901191004467 | 0.225049805402 | Safb  Ddx56  Uhmk1  Srsf11  Ptbp3  Thoc7  Eif5a | web link |
| nucleic acid binding | Gene Ontology | GO:0003676 | 5 (5.75%) | 594 (2.87%) | 0.105023242521 | 0.225049805402 | Ptbp3  Ddx56  Safb  Uhmk1  Srsf11 | web link |
| molecular\_function | Gene Ontology | GO:0003674 | 19 (21.84%) | 3542 (17.11%) | 0.151484058226 | 0.284032609174 | Ociad2  Trappc8  Fam160a2  Bri3bp  Tmem258  Ccdc58  Wbp1l  Szrd1  Gmppa  Card19  Haghl  Smim10l1  Tmem222  Fance  Zfand4  Mpv17l2  Tmem208  Eapp  Mtfr1l | web link |
| calcium ion binding | Gene Ontology | GO:0005509 | 5 (5.75%) | 709 (3.43%) | 0.178029217737 | 0.296715362895 | Asph  Lcp1  Proc  Calu  Ppp2r3d | web link |
| transferase activity | Gene Ontology | GO:0016740 | 10 (11.49%) | 1771 (8.56%) | 0.208731546877 | 0.313097320315 | Smurf1  Pi4ka  St3gal4  Gstm4  Gmppa  Uhmk1  Rps6kb2  Elovl5  Sult2a8  Gba2 | web link |
| ATP binding | Gene Ontology | GO:0005524 | 8 (9.20%) | 1484 (7.17%) | 0.284899082675 | 0.38598401843 | Ntpcr  Mthfs  Rtca  Ddx56  Uhmk1  Rps6kb2  Tcp1  Gak | web link |
| protein binding | Gene Ontology | GO:0005515 | 31 (35.63%) | 6748 (32.60%) | 0.308787214744 | 0.38598401843 | Trappc8  Snap23  Asph  Ppfibp1  Coro1b  Raph1  Zfand4  Vegfa  Tmem176b  Clu  Lcp1  Bin1  Kdm5a  Exoc7  Pmf1  Esr1  Safb  Tcp1  Lifr  Thoc7  Emsy  Slc40a1  Gstm4  Uhmk1  Cep43  Smurf1  Mff  Cflar  Prkar1a  Ncor2  Cln8 | web link |
| nucleotide binding | Gene Ontology | GO:0000166 | 9 (10.34%) | 1795 (8.67%) | 0.341040160145 | 0.39350787709 | Ntpcr  Mthfs  Rtca  Ddx56  Uhmk1  Rps6kb2  Tcp1  Prkar1a  Mmaa | web link |
| metal ion binding | Gene Ontology | GO:0046872 | 15 (17.24%) | 3545 (17.13%) | 0.533144843522 | 0.571226618059 | Esr1  Hagh  Mthfs  Asph  Haghl  Rnpepl1  Calu  Zfand4  Adhfe1  Ablim2  Cyb561d1  Lcp1  Kdm5a  Dido1  Fbp1 | web link |
| DNA binding | Gene Ontology | GO:0003677 | 6 (6.90%) | 1779 (8.60%) | 0.768913433266 | 0.768913433266 | Esr1  Safb  Ncor2  Ssbp1  Kdm5a  Ncor1 | web link |
