# Supplementary material for: Regulation of Alternative Splicing of Lipid Metabolism Genes in Sepsis-Induced Liver Damage by RNA-Binding Proteins
Source: Inflammation. 2024 May 9;47(6):1952–68. doi: 10.1007/s10753-024-02017-2 (PMC11607010; doi:10.1007/s10753-024-02017-2)
Supplement: Supplementary file 1 — Supplementary file1 (ZIP 38655 KB) [file 10753_2024_2017_MOESM1_ESM.zip › Sup5_AS_GO.zip!/Sup5_AS_GO/ras/co_RAS/GO_KEGG/CLP_vs_Sham_and_CLPDCA_vs_Sham_co_RAS/coDown_nir_GO_analysis/coDown_nir_GO_enrichment_web_P.html]

GO enrichment analyze


# GO enrichment analyze result


| Term | Database | ID | Input number (55) | Background number (20961) | P-Value | Corrected P-Value | Input | Hyperlink |
| --- | --- | --- | --- | --- | --- | --- | --- | --- |
| negative regulation of apoptotic process | Gene Ontology | GO:0043066 | 8 (14.55%) | 570 (2.72%) | 0.000112469447008 | 0.000889355946286 | Slc40a1  Cflar  Angptl4  Vegfa  Clu  Cln8  Proc  Eif5a | web link |
| regulation of apoptotic process | Gene Ontology | GO:0042981 | 5 (9.09%) | 196 (0.94%) | 0.000161701081143 | 0.000889355946286 | Clu  Card19  Cflar  Madd  Esr1 | web link |
| lipid metabolic process | Gene Ontology | GO:0006629 | 7 (12.73%) | 544 (2.60%) | 0.000526458241589 | 0.00147967476053 | Angptl4  St3gal4  Gstm4  Sc5d  Elovl5  Akr1c6  Gba2 | web link |
| oxidation-reduction process | Gene Ontology | GO:0055114 | 7 (12.73%) | 546 (2.60%) | 0.000538063549284 | 0.00147967476053 | Paox  Dhrs3  Akr1e1  Sc5d  Adhfe1  Aldh16a1  Akr1c6 | web link |
| positive regulation of cell population proliferation | Gene Ontology | GO:0008284 | 6 (10.91%) | 590 (2.81%) | 0.00436466732813 | 0.00960226812189 | Cep43  Vegfa  Lifr  Clu  Eapp  Eif5a | web link |
| negative regulation of transcription by RNA polymerase II | Gene Ontology | GO:0000122 | 7 (12.73%) | 913 (4.36%) | 0.00954523489136 | 0.0174995973008 | Uxt  Esr1  Kdm5a  Vegfa  Fbp1  Ncor2  Ncor1 | web link |
| biological\_process | Gene Ontology | GO:0008150 | 14 (25.45%) | 2834 (13.52%) | 0.0128354258759 | 0.0201699549478 | Ntpcr  Card19  Bri3bp  Ccdc58  Wbp1l  Szrd1  Haghl  Smim10l1  Mmaa  Fance  Zfand4  Srsf11  Tmem222  Btf3l4 | web link |
| apoptotic process | Gene Ontology | GO:0006915 | 5 (9.09%) | 567 (2.71%) | 0.0162963477682 | 0.0224074781813 | Cflar  Madd  Uxt  Dido1  Eif5a | web link |
| protein phosphorylation | Gene Ontology | GO:0006468 | 5 (9.09%) | 616 (2.94%) | 0.0224383675509 | 0.0274246714511 | Prkar1a  Phkb  Gak  Uhmk1  Rps6kb2 | web link |
| cell differentiation | Gene Ontology | GO:0030154 | 5 (9.09%) | 918 (4.38%) | 0.0921669042141 | 0.101383594636 | Ptbp3  Smurf1  Vegfa  Bin1  Tmem176b | web link |
| positive regulation of transcription by RNA polymerase II | Gene Ontology | GO:0045944 | 5 (9.09%) | 1201 (5.73%) | 0.206036646583 | 0.206036646583 | Safb  Ablim2  Vegfa  Slc40a1  Esr1 | web link |
