# Supplementary material for: Regulation of Alternative Splicing of Lipid Metabolism Genes in Sepsis-Induced Liver Damage by RNA-Binding Proteins
Source: Inflammation. 2024 May 9;47(6):1952–68. doi: 10.1007/s10753-024-02017-2 (PMC11607010; doi:10.1007/s10753-024-02017-2)
Supplement: Supplementary file 1 — Supplementary file1 (ZIP 38655 KB) [file 10753_2024_2017_MOESM1_ESM.zip › Sup5_AS_GO.zip!/Sup5_AS_GO/ras/co_RAS/GO_KEGG/CLP_vs_Sham_and_CLPDCA_vs_Sham_co_RAS/coDown_nir_GO_analysis/coDown_nir_GO_enrichment_P_top.pdf]

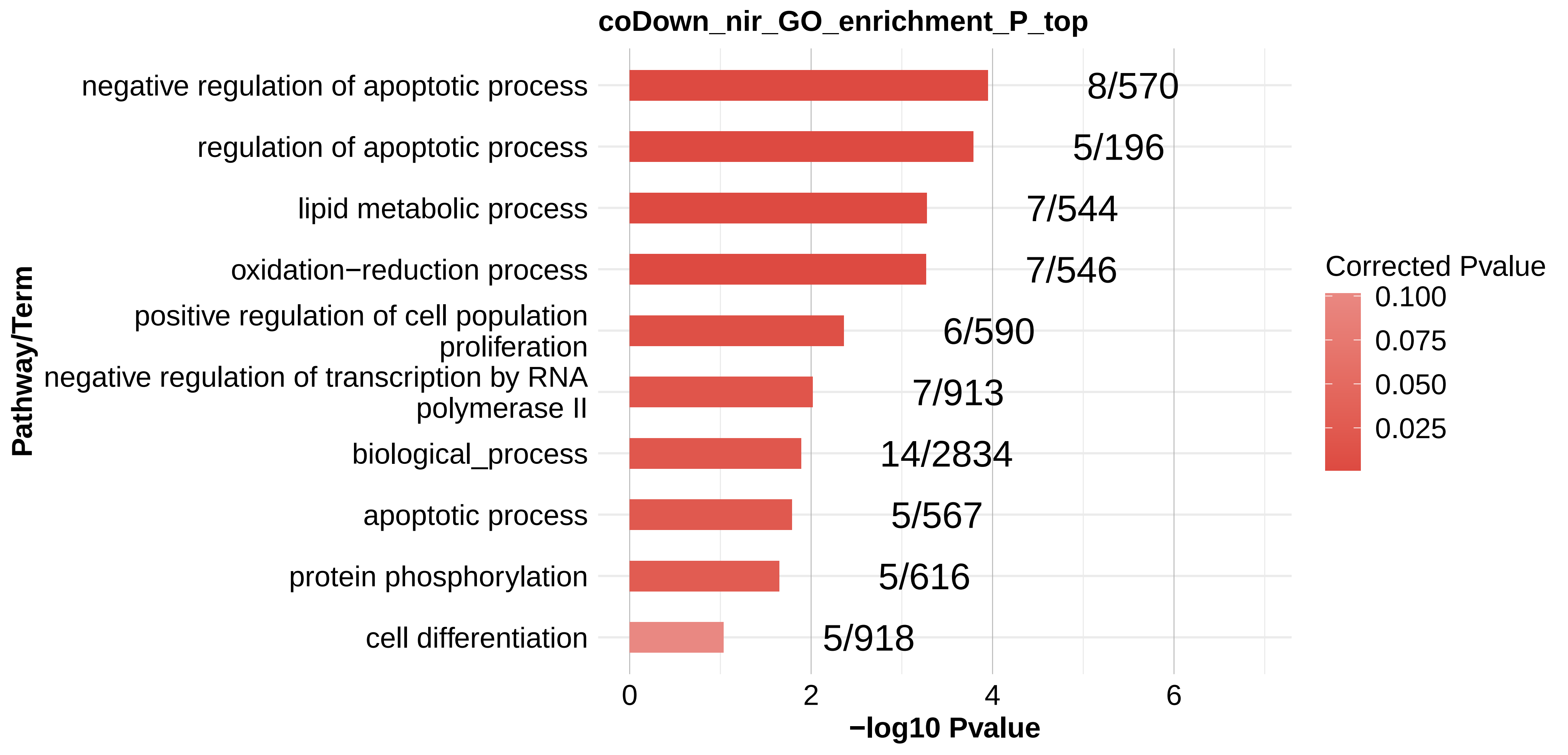
coDown_nir_GO_enrichment_P_top
Pathway/Term
negative regulation of apoptotic process
regulation of apoptotic process
lipid metabolic process
oxidation−reduction process
positive regulation of cell population proliferation
negative regulation of transcription by RNA polymerase II
biological_process
apoptotic process
protein phosphorylation
cell differentiation
8/570
5/196
7/544
7/546
6/590
7/913
14/2834
5/567
5/616
5/918
0
2
4
6
−log10 Pvalue
Corrected Pvalue
0.100
0.075
0.050
0.025
