# Supplementary material for: Regulation of Alternative Splicing of Lipid Metabolism Genes in Sepsis-Induced Liver Damage by RNA-Binding Proteins
Source: Inflammation. 2024 May 9;47(6):1952–68. doi: 10.1007/s10753-024-02017-2 (PMC11607010; doi:10.1007/s10753-024-02017-2)
Supplement: Supplementary file 1 — Supplementary file1 (ZIP 38655 KB) [file 10753_2024_2017_MOESM1_ESM.zip › Sup5_AS_GO.zip!/Sup5_AS_GO/ras/co_RAS/GO_KEGG/CLP_vs_Sham_and_CLPDCA_vs_Sham_co_RAS/coDown_nir_GO_analysis/coDown_nir_GO_enrichment_F_top.pdf]

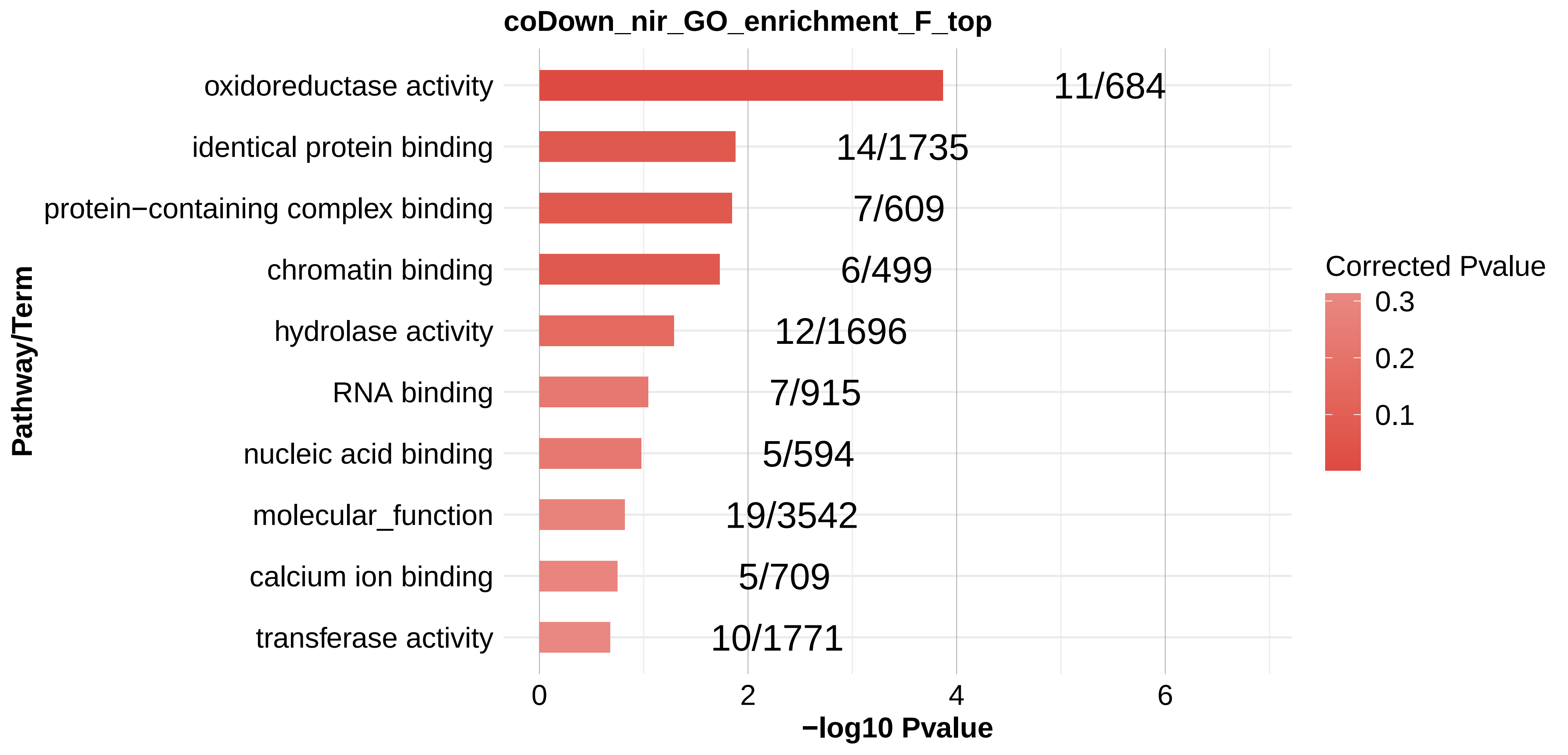
coDown_nir_GO_enrichment_F_top
Pathway/Term
oxidoreductase activity
identical protein binding
protein−containing complex binding
chromatin binding
hydrolase activity
RNA binding
nucleic acid binding
molecular_function
calcium ion binding
transferase activity
11/684
14/1735
7/609
6/499
12/1696
7/915
5/594
19/3542
5/709
10/1771
0
2
4
6
−log10 Pvalue
Corrected Pvalue
0.3
0.2
0.1
