# Supplementary material for: Regulation of Alternative Splicing of Lipid Metabolism Genes in Sepsis-Induced Liver Damage by RNA-Binding Proteins
Source: Inflammation. 2024 May 9;47(6):1952–68. doi: 10.1007/s10753-024-02017-2 (PMC11607010; doi:10.1007/s10753-024-02017-2)
Supplement: Supplementary file 1 — Supplementary file1 (ZIP 38655 KB) [file 10753_2024_2017_MOESM1_ESM.zip › Sup5_AS_GO.zip!/Sup5_AS_GO/ras/co_RAS/GO_KEGG/CLP_vs_Sham_and_CLPDCA_vs_Sham_co_RAS/coUp_nir_GO_analysis/coUp_nir_GO_enrichment_web_C.html]

GO enrichment analyze


# GO enrichment analyze result


| Term | Database | ID | Input number (58) | Background number (20939) | P-Value | Corrected P-Value | Input | Hyperlink |
| --- | --- | --- | --- | --- | --- | --- | --- | --- |
| Golgi apparatus | Gene Ontology | GO:0005794 | 11 (18.97%) | 1309 (6.25%) | 0.000819435964277 | 0.0114721034999 | Slc39a11  St3gal5  St3gal4  Enpp2  Calu  Retreg1  Slc38a10  Tcp1  Gak  Zdhhc20  Arv1 | web link |
| mitochondrion | Gene Ontology | GO:0005739 | 11 (18.97%) | 1745 (8.33%) | 0.00769702623711 | 0.0538791836598 | Hoga1  Mterf3  Trak1  Higd1a  Comt  Taz  Agxt2  Apex1  Ndufs2  Sugct  Idh1 | web link |
| endoplasmic reticulum | Gene Ontology | GO:0005783 | 9 (15.52%) | 1518 (7.25%) | 0.0229533117221 | 0.107115454703 | Calu  Ero1b  Retreg1  Cyp4b1  Apex1  Dnajb12  Zdhhc20  Arhgap5  Arv1 | web link |
| endoplasmic reticulum membrane | Gene Ontology | GO:0005789 | 6 (10.34%) | 903 (4.31%) | 0.0382272931383 | 0.133795525984 | Retreg1  Ero1b  Calu  Cyp4b1  Dnajb12  Arv1 | web link |
| intracellular membrane-bounded organelle | Gene Ontology | GO:0043231 | 6 (10.34%) | 1061 (5.07%) | 0.072409239898 | 0.202745871714 | Comt  Calu  Cyp4b1  Gak  Zdhhc20  Cyp4a31 | web link |
| cytoplasm | Gene Ontology | GO:0005737 | 23 (39.66%) | 6677 (31.89%) | 0.130004444222 | 0.271946530145 | Ube2i  Slc39a11  Idh1  Apex1  Ankhd1  Ankib1  Mtmr4  Mapkapk2  Cnot10  Comt  Tcp1  Zdhhc20  Cyp4a31  Gtf2i  Upp2  Arhgef12  Ints7  Ankrd13a  Trak1  Enpp2  Sult2a8  Cpsf7  Itih4 | web link |
| nucleus | Gene Ontology | GO:0005634 | 21 (36.21%) | 6031 (28.80%) | 0.135973265073 | 0.271946530145 | Ube2i  Ube2d3  Slc39a11  Ints7  Zbtb14  Higd1a  Trak1  Cnot10  Safb  Snrpa1  Mapkapk2  Apex1  Dnajb12  Zfp62  Zfp874a  Gtf2a2  Tmem176b  Cpsf7  Gtf2i  Hnrnpll  Thoc2l | web link |
| membrane | Gene Ontology | GO:0016020 | 27 (46.55%) | 8771 (41.89%) | 0.277004935638 | 0.484758637367 | Slc39a11  Ero1b  Calu  Comt  Slc38a10  Tmem176b  Mtmr4  Arv1  Higd1a  Ube2d3  Tmprss6  Dnajb12  Ndufs2  St3gal5  St3gal4  Retreg1  Susd1  Zdhhc20  Cyp4a31  Arhgef12  Fxyd1  Trak1  Ankrd13a  Enpp2  Cyp4b1  Taz  AI182371 | web link |
| nucleoplasm | Gene Ontology | GO:0005654 | 10 (17.24%) | 3156 (15.07%) | 0.375371628816 | 0.554509052155 | Ube2i  Zbtb14  Higd1a  Safb  Snrpa1  Apex1  Ndufs2  Gtf2a2  Gtf2i  Mapkapk2 | web link |
| extracellular region | Gene Ontology | GO:0005576 | 6 (10.34%) | 1829 (8.73%) | 0.396077894397 | 0.554509052155 | Selenop  C4bp  Enpp2  Calu  AI182371  Itih4 | web link |
| integral component of membrane | Gene Ontology | GO:0016021 | 19 (32.76%) | 6684 (31.92%) | 0.494808825397 | 0.618067163334 | Slc39a11  Tmem176b  Comt  St3gal5  St3gal4  Zdhhc20  Higd1a  Fxyd1  Enpp2  Retreg1  Taz  Cyp4b1  Slc38a10  Tmprss6  Susd1  AI182371  Arv1  Cyp4a31  Dnajb12 | web link |
| extracellular space | Gene Ontology | GO:0005615 | 5 (8.62%) | 1732 (8.27%) | 0.529771854287 | 0.618067163334 | Selenop  Enpp2  Serpina3n  Tmprss6  Itih4 | web link |
| cytosol | Gene Ontology | GO:0005829 | 9 (15.52%) | 3762 (17.97%) | 0.737632326608 | 0.794373274809 | Ube2i  Upp2  Zbtb14  Mterf3  Idh1  Comt  Tcp1  Gak  Arhgap5 | web link |
| plasma membrane | Gene Ontology | GO:0005886 | 8 (13.79%) | 5547 (26.49%) | 0.993640301456 | 0.993640301456 | Slc39a11  Ankrd13a  Fxyd1  Ube2d3  Tmprss6  Comt  Zdhhc20  Itih4 | web link |
