# Supplementary material for: Regulation of Alternative Splicing of Lipid Metabolism Genes in Sepsis-Induced Liver Damage by RNA-Binding Proteins
Source: Inflammation. 2024 May 9;47(6):1952–68. doi: 10.1007/s10753-024-02017-2 (PMC11607010; doi:10.1007/s10753-024-02017-2)
Supplement: Supplementary file 1 — Supplementary file1 (ZIP 38655 KB) [file 10753_2024_2017_MOESM1_ESM.zip › Sup5_AS_GO.zip!/Sup5_AS_GO/ras/co_RAS/GO_KEGG/CLP_vs_Sham_and_CLPDCA_vs_Sham_co_RAS/coUp_nir_GO_analysis/coUp_nir_GO_enrichment_web_F.html]

GO enrichment analyze


# GO enrichment analyze result


| Term | Database | ID | Input number (50) | Background number (20698) | P-Value | Corrected P-Value | Input | Hyperlink |
| --- | --- | --- | --- | --- | --- | --- | --- | --- |
| transferase activity | Gene Ontology | GO:0016740 | 13 (26.00%) | 1771 (8.56%) | 0.000220051112662 | 0.00242056223928 | Ube2i  Upp2  Comt  St3gal5  St3gal4  Pcyt2  Ube2d3  Sugct  Zdhhc20  Sult2a8  Ankib1  Agxt2  Mapkapk2 | web link |
| catalytic activity | Gene Ontology | GO:0003824 | 5 (10.00%) | 405 (1.96%) | 0.00287711004688 | 0.0158241052578 | Hoga1  Pcyt2  Enpp2  Upp2  Agxt2 | web link |
| oxidoreductase activity | Gene Ontology | GO:0016491 | 6 (12.00%) | 684 (3.30%) | 0.00588222929025 | 0.0215681740643 | Idh1  Ero1b  Cyp4b1  Apex1  Ndufs2  Cyp4a31 | web link |
| nucleic acid binding | Gene Ontology | GO:0003676 | 5 (10.00%) | 594 (2.87%) | 0.0140110205537 | 0.0385303065227 | Cpsf7  Safb  Enpp2  Hnrnpll  Ankhd1 | web link |
| RNA binding | Gene Ontology | GO:0003723 | 6 (12.00%) | 915 (4.42%) | 0.0222704394468 | 0.048994966783 | Safb  Snrpa1  Apex1  Ankhd1  Cpsf7  Hnrnpll | web link |
| metal ion binding | Gene Ontology | GO:0046872 | 14 (28.00%) | 3545 (17.13%) | 0.0376111122414 | 0.0689537057759 | Zbtb14  Calu  Idh1  Enpp2  Cyp4b1  Comt  Apex1  Ankib1  Ndufs2  Zfp874a  Zdhhc20  Cyp4a31  Mtmr4  Zfp62 | web link |
| protein binding | Gene Ontology | GO:0005515 | 20 (40.00%) | 6748 (32.60%) | 0.166588536002 | 0.261781985145 | Ube2i  Mapkapk2  Arhgef12  Tcp1  Ankrd13a  Ankhd1  Zbtb14  Cnot10  Safb  Ndufs2  Tmprss6  Ube2d3  Plekha6  C4bp  Snrpa1  Tmem176b  Ankib1  Gtf2i  Selenop  Itih4 | web link |
| DNA binding | Gene Ontology | GO:0003677 | 6 (12.00%) | 1779 (8.60%) | 0.257273460238 | 0.351336113964 | Hmces  Zbtb14  Mterf3  Safb  Apex1  Gtf2i | web link |
| ATP binding | Gene Ontology | GO:0005524 | 5 (10.00%) | 1484 (7.17%) | 0.287456820516 | 0.351336113964 | Ube2i  Tcp1  Gak  Mapkapk2  Ube2d3 | web link |
| hydrolase activity | Gene Ontology | GO:0016787 | 5 (10.00%) | 1696 (8.19%) | 0.39082339773 | 0.429905737504 | Apex1  Mtmr4  Enpp2  Hmces  Tmprss6 | web link |
| molecular\_function | Gene Ontology | GO:0003674 | 7 (14.00%) | 3542 (17.11%) | 0.775152157051 | 0.775152157051 | Cnot10  Higd1a  Retreg1  Plekha6  Susd1  2700062C07Rik  Arv1 | web link |
