# Supplementary material for: Regulation of Alternative Splicing of Lipid Metabolism Genes in Sepsis-Induced Liver Damage by RNA-Binding Proteins
Source: Inflammation. 2024 May 9;47(6):1952–68. doi: 10.1007/s10753-024-02017-2 (PMC11607010; doi:10.1007/s10753-024-02017-2)
Supplement: Supplementary file 1 — Supplementary file1 (ZIP 38655 KB) [file 10753_2024_2017_MOESM1_ESM.zip › Sup5_AS_GO.zip!/Sup5_AS_GO/ras/co_RAS/GO_KEGG/CLP_vs_Sham_and_CLPDCA_vs_Sham_co_RAS/coUp_nir_GO_analysis/coUp_nir_GO_enrichment_C_top.pdf]

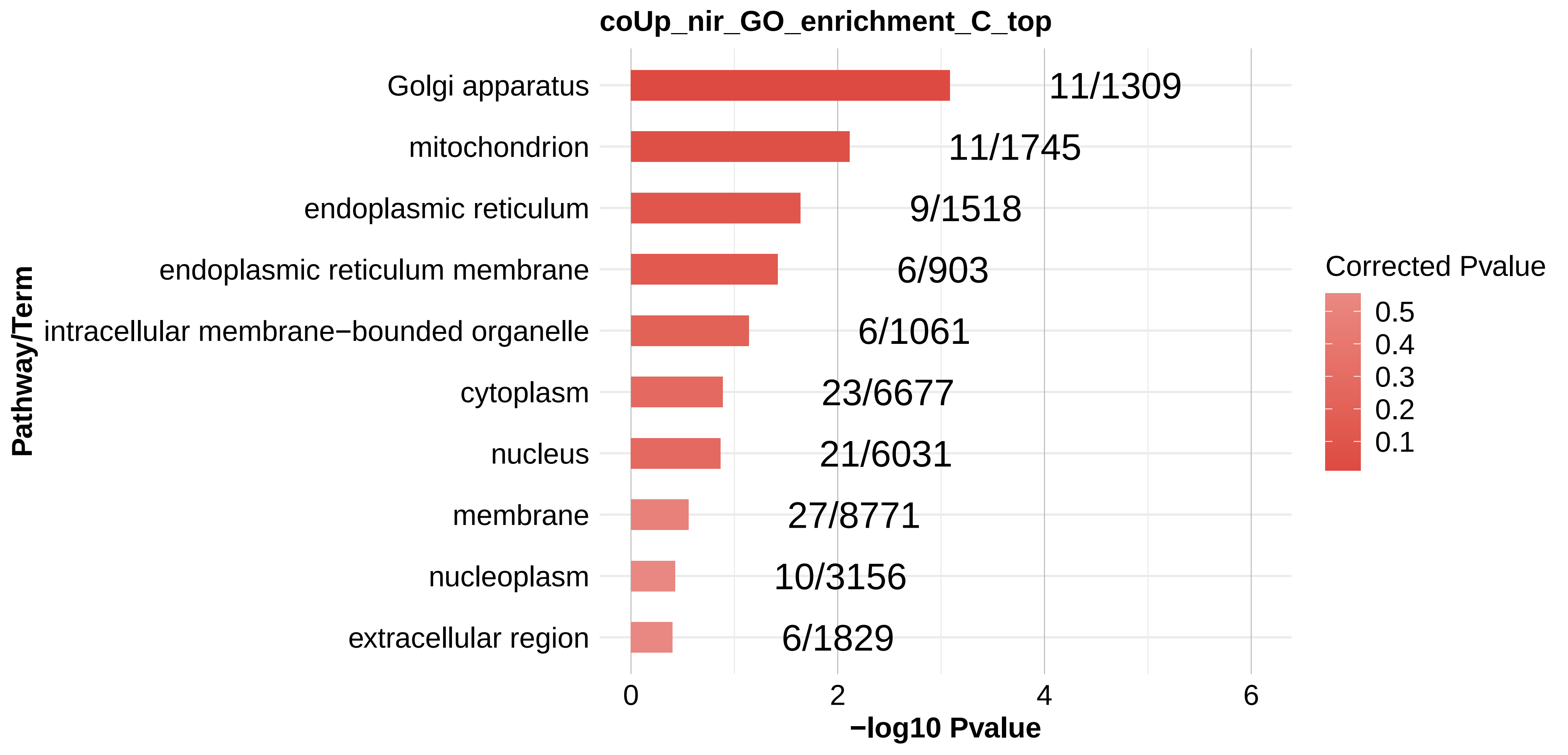
coUp_nir_GO_enrichment_C_top
Pathway/Term
Golgi apparatus
mitochondrion
endoplasmic reticulum
endoplasmic reticulum membrane
intracellular membrane−bounded organelle
cytoplasm
nucleus
membrane
nucleoplasm
extracellular region
11/1309
11/1745
9/1518
6/903
6/1061
23/6677
21/6031
27/8771
10/3156
6/1829
0
2
4
6
−log10 Pvalue
Corrected Pvalue
0.5
0.4
0.3
0.2
0.1
