# Supplementary material for: Regulation of Alternative Splicing of Lipid Metabolism Genes in Sepsis-Induced Liver Damage by RNA-Binding Proteins
Source: Inflammation. 2024 May 9;47(6):1952–68. doi: 10.1007/s10753-024-02017-2 (PMC11607010; doi:10.1007/s10753-024-02017-2)
Supplement: Supplementary file 1 — Supplementary file1 (ZIP 38655 KB) [file 10753_2024_2017_MOESM1_ESM.zip › Sup5_AS_GO.zip!/Sup5_AS_GO/ras/co_RAS/GO_KEGG/CLP_vs_Sham_and_CLPDCA_vs_Sham_co_RAS/coUp_nir_GO_analysis/coUp_nir_GO_enrichment_F_top.pdf]

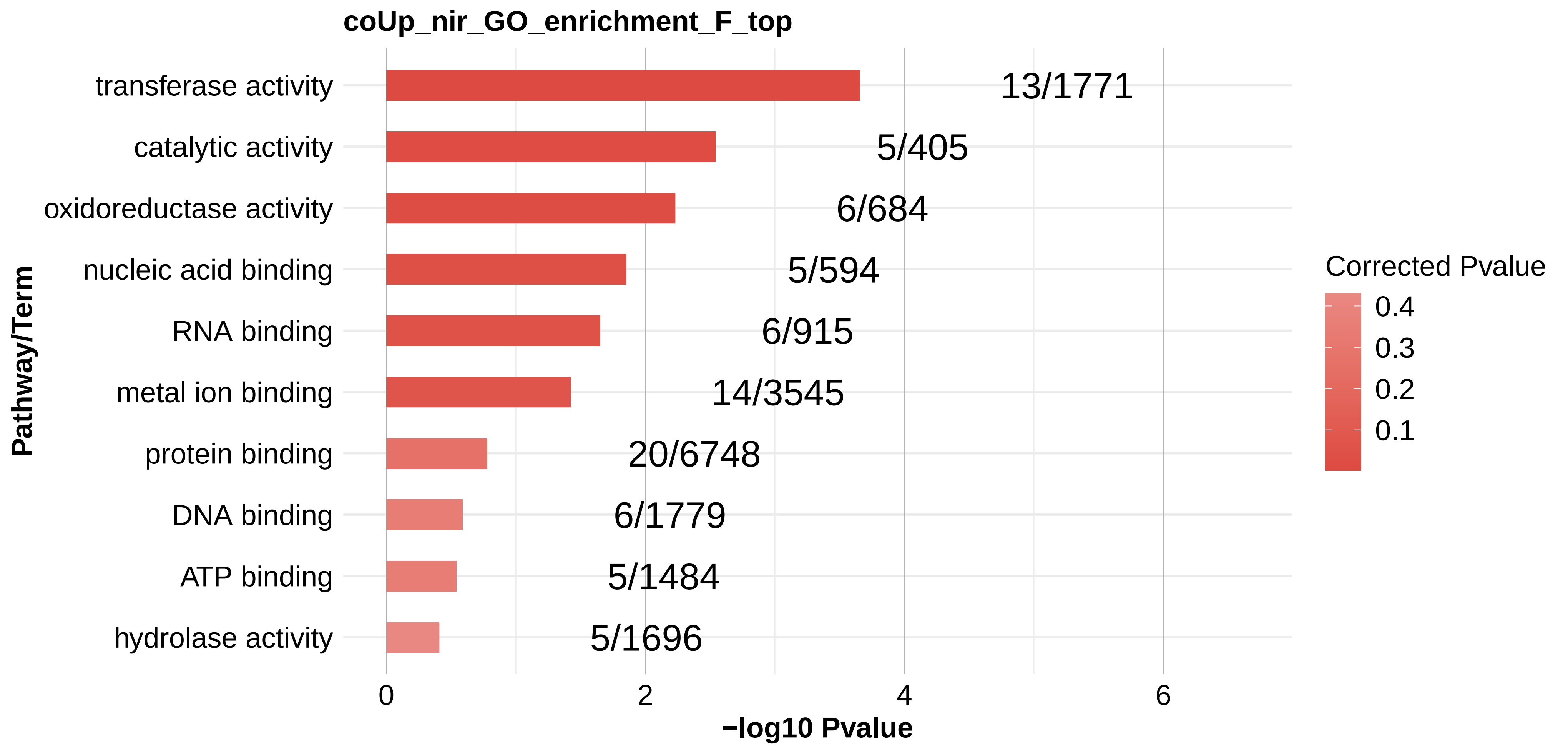

coUp_nir_GO_enrichment_F_top
transferase activity
catalytic activity
oxidoreductase activity
nucleic acid binding
RNA binding
metal ion binding
protein binding
DNA binding
ATP binding
hydrolase activity
13/1771
5/405
6/684
5/594
6/915
14/3545
20/6748
6/1779
5/1484
5/1696
Pathway/Term
0
2
4
6
−log10 Pvalue
Corrected Pvalue
0.4
0.3
0.2
0.1
