# Supplementary material for: Regulation of Alternative Splicing of Lipid Metabolism Genes in Sepsis-Induced Liver Damage by RNA-Binding Proteins
Source: Inflammation. 2024 May 9;47(6):1952–68. doi: 10.1007/s10753-024-02017-2 (PMC11607010; doi:10.1007/s10753-024-02017-2)
Supplement: Supplementary file 1 — Supplementary file1 (ZIP 38655 KB) [file 10753_2024_2017_MOESM1_ESM.zip › Sup5_AS_GO.zip!/Sup5_AS_GO/ras/co_RAS/GO_KEGG/CLP_vs_Sham_and_CLPDCA_vs_Sham_co_RAS/coUp_nir_GO_analysis/coUp_nir_GO_enrichment_web_P.html]

GO enrichment analyze


# GO enrichment analyze result


| Term | Database | ID | Input number (11) | Background number (20961) | P-Value | Corrected P-Value | Input | Hyperlink |
| --- | --- | --- | --- | --- | --- | --- | --- | --- |
| oxidation-reduction process | Gene Ontology | GO:0055114 | 5 (45.45%) | 546 (2.60%) | 4.77738812221e-06 | 9.55477624442e-06 | Cyp4a31  Ero1b  Idh1  Cyp4b1  Ndufs2 | web link |
| positive regulation of transcription by RNA polymerase II | Gene Ontology | GO:0045944 | 6 (54.55%) | 1201 (5.73%) | 1.25809779064e-05 | 1.25809779064e-05 | Safb  Tmprss6  Apex1  Gtf2a2  Gtf2i  Zfp62 | web link |
