# Supplementary material for: Regulation of Alternative Splicing of Lipid Metabolism Genes in Sepsis-Induced Liver Damage by RNA-Binding Proteins
Source: Inflammation. 2024 May 9;47(6):1952–68. doi: 10.1007/s10753-024-02017-2 (PMC11607010; doi:10.1007/s10753-024-02017-2)
Supplement: Supplementary file 1 — Supplementary file1 (ZIP 38655 KB) [file 10753_2024_2017_MOESM1_ESM.zip › Sup5_AS_GO.zip!/Sup5_AS_GO/rasg/CLP_vs_Sham/RASG_DEG_overlap_GO_analysis/RASG_DEG_overlap_GO_enrichment_web_C.html]

GO enrichment analyze


# GO enrichment analyze result


| Term | Database | ID | Input number (53) | Background number (20939) | P-Value | Corrected P-Value | Input | Hyperlink |
| --- | --- | --- | --- | --- | --- | --- | --- | --- |
| extracellular region | Gene Ontology | GO:0005576 | 13 (24.53%) | 1829 (8.73%) | 0.000499906521227 | 0.00586234025796 | Lipc  Wfdc2  Serpina3m  Fn1  St6gal1  Cnmd  Hp  Angptl4  S100a9  Lifr  Manf  Cp  Il18bp | web link |
| extracellular space | Gene Ontology | GO:0005615 | 12 (22.64%) | 1732 (8.27%) | 0.00107713414779 | 0.00586234025796 | Lipc  Wfdc2  Serpina3n  Fn1  Ces3a  Hp  Angptl4  S100a9  Manf  Cp  Il18bp  Serpina3m | web link |
| endoplasmic reticulum | Gene Ontology | GO:0005783 | 11 (20.75%) | 1518 (7.25%) | 0.00125621576956 | 0.00586234025796 | Cyp2c40  Ces3a  Fmo5  Tmem131l  Hp  St6gal1  Rhbdf2  Srebf1  Insig2  Ugt1a9  Manf | web link |
| endoplasmic reticulum membrane | Gene Ontology | GO:0005789 | 7 (13.21%) | 903 (4.31%) | 0.00741251573794 | 0.0259438050828 | Fmo5  Cyp3a59  Rhbdf2  Srebf1  Insig2  Ugt1a9  Cyp2c40 | web link |
| intracellular membrane-bounded organelle | Gene Ontology | GO:0043231 | 7 (13.21%) | 1061 (5.07%) | 0.0169479881002 | 0.0474543666806 | Fmo5  Cyp3a59  Srebf1  Cyp2c40  Ugt2b36  Cyp4a31  D130043K22Rik | web link |
| integral component of membrane | Gene Ontology | GO:0016021 | 21 (39.62%) | 6684 (31.92%) | 0.145619616298 | 0.339779104695 | Slc22a30  Gm5150  St3gal5  St6gal1  Cnmd  Cyp3a59  Fmo5  Tmem131l  Clcn2  Fndc3b  Maob  Rhbdf2  Srebf1  Insig2  Lifr  Slc25a25  Ugt2b36  Cyp4a31  Cp  D130043K22Rik  Ugt1a9 | web link |
| cytosol | Gene Ontology | GO:0005829 | 12 (22.64%) | 3762 (17.97%) | 0.233437243228 | 0.412071004751 | Upp2  Fmo5  Mlxipl  Bdh2  Tacc2  Uox  Gsta2  Raph1  S100a9  Tprkb  Setd4  Mt2 | web link |
| Golgi apparatus | Gene Ontology | GO:0005794 | 5 (9.43%) | 1309 (6.25%) | 0.235469145572 | 0.412071004751 | Mapk15  Hp  Srebf1  St3gal5  St6gal1 | web link |
| mitochondrion | Gene Ontology | GO:0005739 | 5 (9.43%) | 1745 (8.33%) | 0.455568739891 | 0.649370647296 | Ociad2  Maob  Uox  Gls2  Slc25a25 | web link |
| membrane | Gene Ontology | GO:0016020 | 23 (43.40%) | 8771 (41.89%) | 0.46383617664 | 0.649370647296 | Fmo5  Slc22a30  Maob  Cyp2c40  Ugt2b36  Cnmd  Tmem131l  Rhbdf2  Lifr  Fndc3b  St3gal5  St6gal1  Cyp3a59  Insig2  Cyp4a31  D130043K22Rik  Gm5150  Clcn2  Srebf1  Ugt1a9  Slc25a25  S100a9  Cp | web link |
| cytoplasm | Gene Ontology | GO:0005737 | 17 (32.08%) | 6677 (31.89%) | 0.539959871291 | 0.68722165437 | Upp2  Paox  Cyp2c40  Tmem131l  Bdh2  Tacc2  Pfkfb2  Mapk15  Srebf1  S100a9  Sult2a8  Ablim2  Cyp4a31  Tprkb  Setd4  Mt2  Mlxipl | web link |
| nucleoplasm | Gene Ontology | GO:0005654 | 5 (9.43%) | 3156 (15.07%) | 0.917753776834 | 0.930139952728 | Mlxipl  Tacc2  Srebf1  S100a9  Pfkfb2 | web link |
| plasma membrane | Gene Ontology | GO:0005886 | 10 (18.87%) | 5547 (26.49%) | 0.925904265509 | 0.930139952728 | Gm5150  Tmem131l  Clcn2  Tacc2  Raph1  Rhbdf2  S100a9  Lifr  Cp  D130043K22Rik | web link |
| nucleus | Gene Ontology | GO:0005634 | 11 (20.75%) | 6031 (28.80%) | 0.930139952728 | 0.930139952728 | Sbno2  Mlxipl  Tprkb  Tacc2  Tef  Mapk15  Srebf1  S100a9  Shld2  Setd4  Mt2 | web link |
