# Supplementary material for: Regulation of Alternative Splicing of Lipid Metabolism Genes in Sepsis-Induced Liver Damage by RNA-Binding Proteins
Source: Inflammation. 2024 May 9;47(6):1952–68. doi: 10.1007/s10753-024-02017-2 (PMC11607010; doi:10.1007/s10753-024-02017-2)
Supplement: Supplementary file 1 — Supplementary file1 (ZIP 38655 KB) [file 10753_2024_2017_MOESM1_ESM.zip › Sup5_AS_GO.zip!/Sup5_AS_GO/rasg/CLP_vs_Sham/RASG_DEG_overlap_GO_analysis/RASG_DEG_overlap_GO_enrichment_web_F.html]

GO enrichment analyze


# GO enrichment analyze result


| Term | Database | ID | Input number (43) | Background number (20698) | P-Value | Corrected P-Value | Input | Hyperlink |
| --- | --- | --- | --- | --- | --- | --- | --- | --- |
| oxidoreductase activity | Gene Ontology | GO:0016491 | 9 (20.93%) | 684 (3.30%) | 9.18637361158e-06 | 6.43046152811e-05 | Fmo5  Paox  Cyp3a59  Uox  Maob  Cyp2c40  Cyp4a31  Cp  Bdh2 | web link |
| transferase activity, transferring glycosyl groups | Gene Ontology | GO:0016757 | 5 (11.63%) | 230 (1.11%) | 0.000110677588873 | 0.000387371561057 | Ugt2b36  Upp2  Ugt1a9  St3gal5  St6gal1 | web link |
| transferase activity | Gene Ontology | GO:0016740 | 11 (25.58%) | 1771 (8.56%) | 0.000768271731926 | 0.00179263404116 | Upp2  Papss2  St3gal5  St6gal1  Pfkfb2  Gsta2  Mapk15  Ugt1a9  Sult2a8  Ugt2b36  Setd4 | web link |
| identical protein binding | Gene Ontology | GO:0042802 | 7 (16.28%) | 1735 (8.38%) | 0.0647482667179 | 0.113309466756 | Upp2  Lipc  Fn1  Hp  Tef  Maob  Angptl4 | web link |
| metal ion binding | Gene Ontology | GO:0046872 | 9 (20.93%) | 3545 (17.13%) | 0.310494303404 | 0.434692024765 | Lipc  Mt2  Cyp3a59  Slc25a25  S100a9  Cyp2c40  Ablim2  Cyp4a31  Cp | web link |
| protein binding | Gene Ontology | GO:0005515 | 14 (32.56%) | 6748 (32.60%) | 0.559662341094 | 0.652939397943 | Sbno2  Lipc  Tef  Fn1  Raph1  Mlxipl  Gsta2  Mapk15  Rhbdf2  Srebf1  Lifr  Fndc3b  Setd4  Gls2 | web link |
| molecular\_function | Gene Ontology | GO:0003674 | 6 (13.95%) | 3542 (17.11%) | 0.768247799655 | 0.768247799655 | Ociad2  Gm5150  Tmem131l  Manf  Shld2  D130043K22Rik | web link |
