# Supplementary material for: Regulation of Alternative Splicing of Lipid Metabolism Genes in Sepsis-Induced Liver Damage by RNA-Binding Proteins
Source: Inflammation. 2024 May 9;47(6):1952–68. doi: 10.1007/s10753-024-02017-2 (PMC11607010; doi:10.1007/s10753-024-02017-2)
Supplement: Supplementary file 1 — Supplementary file1 (ZIP 38655 KB) [file 10753_2024_2017_MOESM1_ESM.zip › Sup5_AS_GO.zip!/Sup5_AS_GO/rasg/CLP_vs_Sham/RASG_DEG_overlap_GO_analysis/RASG_DEG_overlap_GO_enrichment_web_P.html]

GO enrichment analyze


# GO enrichment analyze result


| Term | Database | ID | Input number (17) | Background number (20961) | P-Value | Corrected P-Value | Input | Hyperlink |
| --- | --- | --- | --- | --- | --- | --- | --- | --- |
| oxidation-reduction process | Gene Ontology | GO:0055114 | 9 (52.94%) | 546 (2.60%) | 1.04411146395e-10 | 3.13233439186e-10 | Fmo5  Paox  Cyp3a59  Uox  Maob  Cyp2c40  Cyp4a31  Cp  Bdh2 | web link |
| lipid metabolic process | Gene Ontology | GO:0006629 | 5 (29.41%) | 544 (2.60%) | 5.52258547564e-05 | 8.28387821346e-05 | Insig2  Lipc  Angptl4  Srebf1  Cyp2c40 | web link |
| positive regulation of transcription by RNA polymerase II | Gene Ontology | GO:0045944 | 5 (29.41%) | 1201 (5.73%) | 0.00212274046042 | 0.00212274046042 | Mlxipl  Sbno2  Srebf1  Tef  Ablim2 | web link |
