# Supplementary material for: Regulation of Alternative Splicing of Lipid Metabolism Genes in Sepsis-Induced Liver Damage by RNA-Binding Proteins
Source: Inflammation. 2024 May 9;47(6):1952–68. doi: 10.1007/s10753-024-02017-2 (PMC11607010; doi:10.1007/s10753-024-02017-2)
Supplement: Supplementary file 1 — Supplementary file1 (ZIP 38655 KB) [file 10753_2024_2017_MOESM1_ESM.zip › Sup5_AS_GO.zip!/Sup5_AS_GO/rasg/CLP_vs_Sham/RASG_DEG_overlap_GO_analysis/RASG_DEG_overlap_GO_enrichment_C_top.pdf]

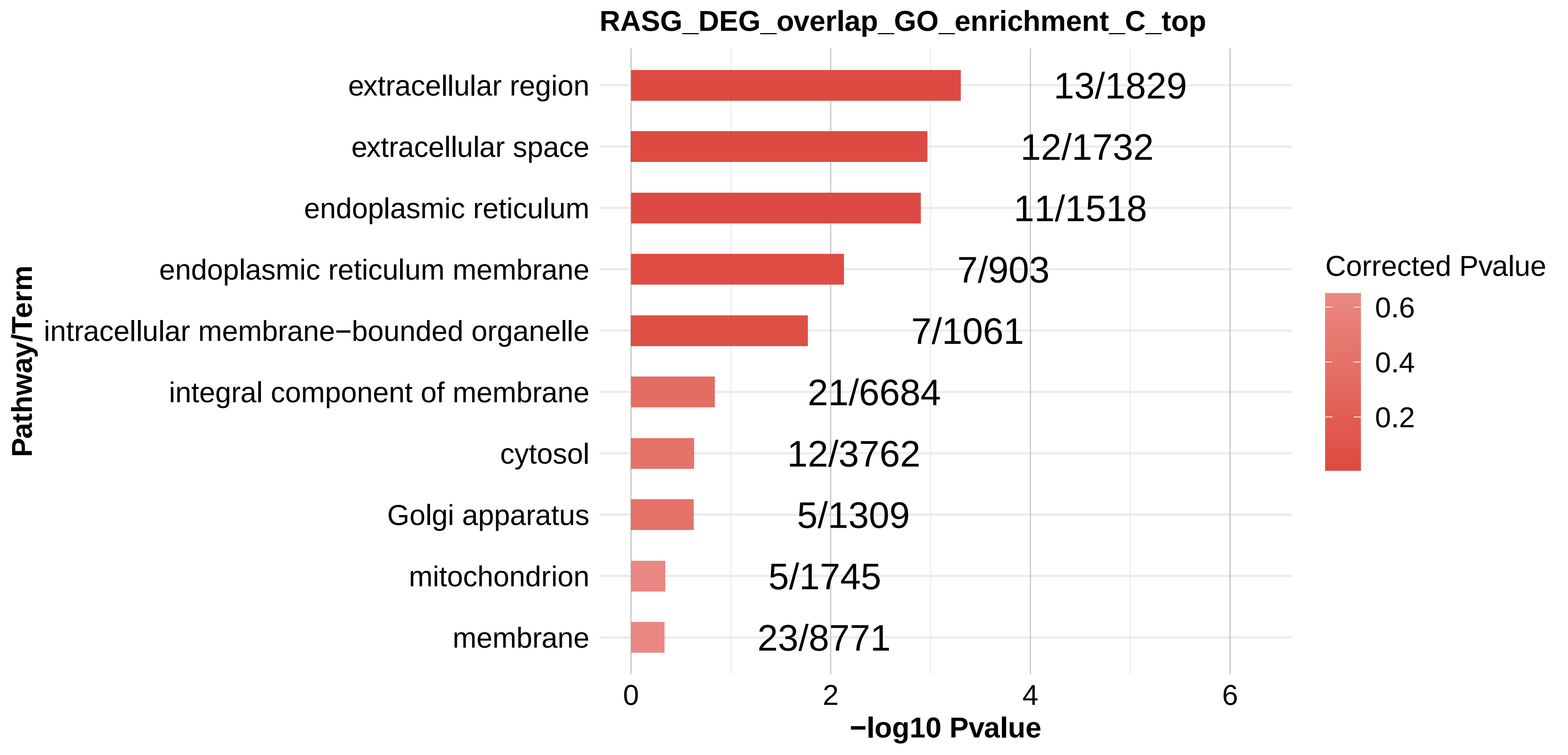

RASG_DEG_overlap_GO_enrichment_C_top
Pathway/Term
extracellular region
extracellular space
endoplasmic reticulum
endoplasmic reticulum membrane
intracellular membrane−bounded organelle
integral component of membrane
cytosol
Golgi apparatus
mitochondrion
membrane
13/1829
12/1732
11/1518
7/903
7/1061
21/6684
12/3762
5/1309
5/1745
23/8771
0
2
4
6
−log10 Pvalue
Corrected Pvalue
0.6
0.4
0.2
