# Supplementary material for: Regulation of Alternative Splicing of Lipid Metabolism Genes in Sepsis-Induced Liver Damage by RNA-Binding Proteins
Source: Inflammation. 2024 May 9;47(6):1952–68. doi: 10.1007/s10753-024-02017-2 (PMC11607010; doi:10.1007/s10753-024-02017-2)
Supplement: Supplementary file 1 — Supplementary file1 (ZIP 38655 KB) [file 10753_2024_2017_MOESM1_ESM.zip › Sup5_AS_GO.zip!/Sup5_AS_GO/rasg/CLP_vs_Sham/RASG_DEG_overlap_GO_analysis/RASG_DEG_overlap_GO_enrichment_F_top.pdf]

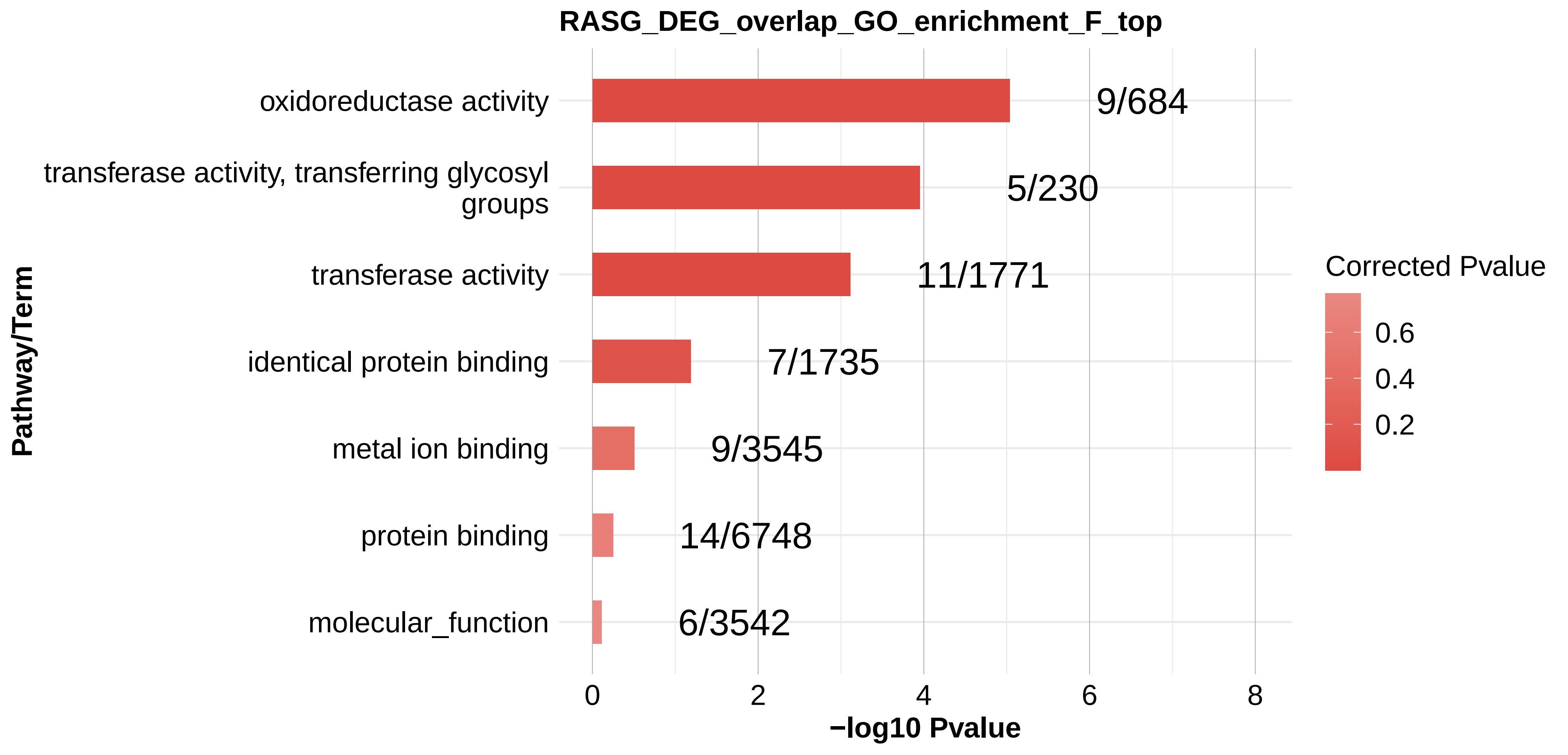
RASG_DEG_overlap_GO_enrichment_F_top
Pathway/Term
oxidoreductase activity
transferase activity, transferring glycosyl groups
transferase activity
identical protein binding
metal ion binding
protein binding
molecular_function
9/684
5/230
11/1771
7/1735
9/3545
14/6748
6/3542
0
2
4
6
8
−log10 Pvalue
Corrected Pvalue
0.6
0.4
0.2
