# Supplementary material for: Regulation of Alternative Splicing of Lipid Metabolism Genes in Sepsis-Induced Liver Damage by RNA-Binding Proteins
Source: Inflammation. 2024 May 9;47(6):1952–68. doi: 10.1007/s10753-024-02017-2 (PMC11607010; doi:10.1007/s10753-024-02017-2)
Supplement: Supplementary file 1 — Supplementary file1 (ZIP 38655 KB) [file 10753_2024_2017_MOESM1_ESM.zip › Sup5_AS_GO.zip!/Sup5_AS_GO/rasg/CLP_vs_Sham/RASG_DEG_overlap_GO_analysis/RASG_DEG_overlap_GO_enrichment_P_top.pdf]

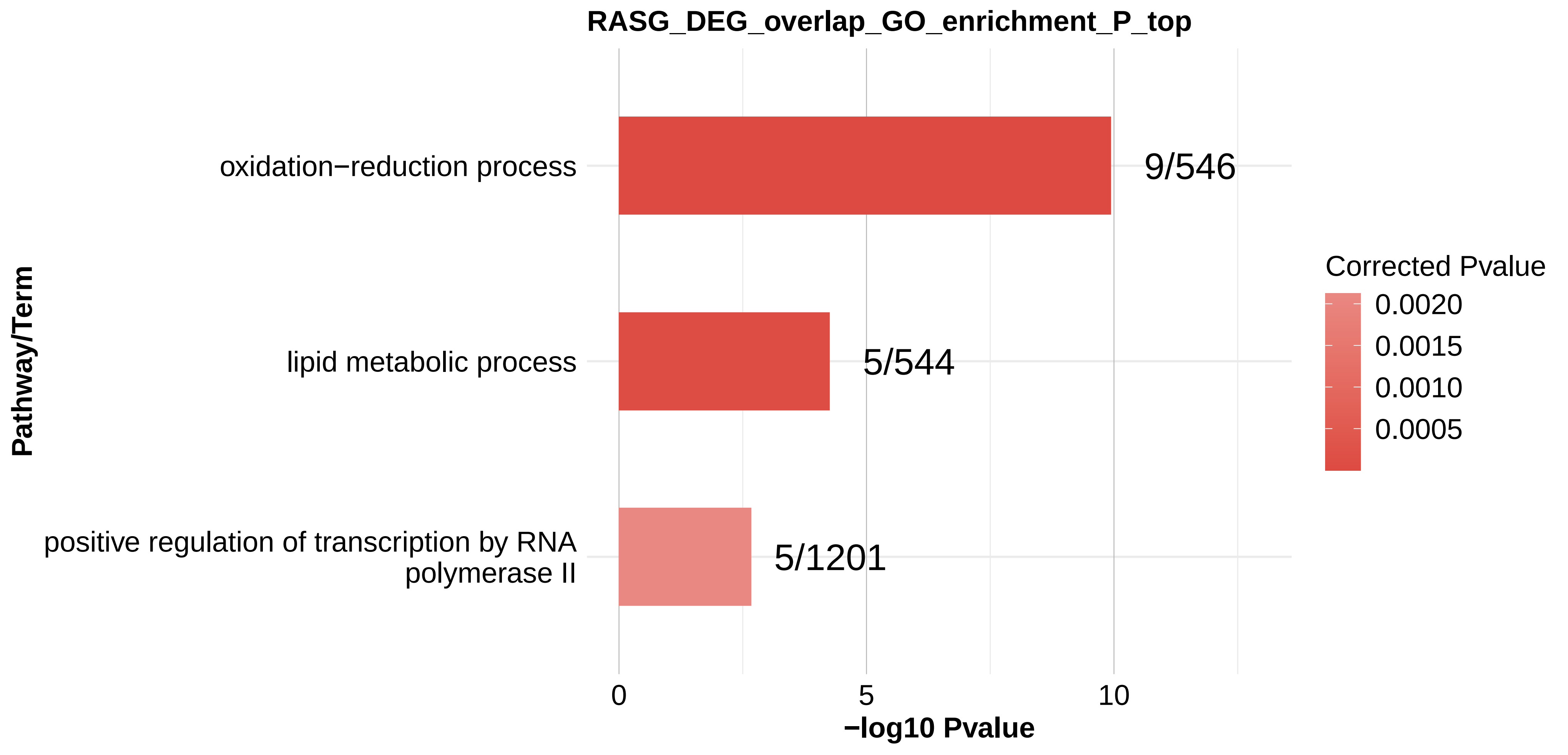
RASG_DEG_overlap_GO_enrichment_P_top
Pathway/Term
oxidation−reduction process
lipid metabolic process
positive regulation of transcription by RNA polymerase II
9/546
5/544
5/1201
0
5
10
−log10 Pvalue
Corrected Pvalue
0.0020
0.0015
0.0010
0.0005
