# Supplementary material for: Regulation of Alternative Splicing of Lipid Metabolism Genes in Sepsis-Induced Liver Damage by RNA-Binding Proteins
Source: Inflammation. 2024 May 9;47(6):1952–68. doi: 10.1007/s10753-024-02017-2 (PMC11607010; doi:10.1007/s10753-024-02017-2)
Supplement: Supplementary file 1 — Supplementary file1 (ZIP 38655 KB) [file 10753_2024_2017_MOESM1_ESM.zip › Sup5_AS_GO.zip!/Sup5_AS_GO/rasg/CLPDCA_vs_Sham/RASG_DEG_overlap_GO_analysis/RASG_DEG_overlap_GO_enrichment_web_P.html]

GO enrichment analyze


# GO enrichment analyze result


| Term | Database | ID | Input number (0) | Background number (20961) | P-Value | Corrected P-Value | Input | Hyperlink |
| --- | --- | --- | --- | --- | --- | --- | --- | --- |
