# Supplementary material for: Regulation of Alternative Splicing of Lipid Metabolism Genes in Sepsis-Induced Liver Damage by RNA-Binding Proteins
Source: Inflammation. 2024 May 9;47(6):1952–68. doi: 10.1007/s10753-024-02017-2 (PMC11607010; doi:10.1007/s10753-024-02017-2)
Supplement: Supplementary file 1 — Supplementary file1 (ZIP 38655 KB) [file 10753_2024_2017_MOESM1_ESM.zip › Sup6_AS_KEGG.zip!/Sup6_AS_KEGG/ras/CLP_vs_Sham/Ras_KEGG_analysis/Ras_KEGG_pathway.html]

KEGG pathway enrichment analyze result


# KEGG pathway enrichment analyze result


| Term | Database | ID | Input number (363) | Background number (8608) | P-Value | Corrected P-Value | Input | Hyperlink | img |
| --- | --- | --- | --- | --- | --- | --- | --- | --- | --- |
| Metabolic pathways | KEGG PATHWAY | mmu01100 | 110 (30.30%) | 1494 (17.36%) | 4.21494839031e-10 | 1.22654998158e-07 | Cers2  Uox  Maob  Aox3  Nme6  Mtmr14  Tm7sf2  Pip5k1c  Ndufs2  Inpp4a  Pigp  Pigv  Cyp2r1  Ptdss1  Nudt9  Mthfs  Mpst  Idnk  Gne  Blvrb  Hsd17b2  Hsd17b6  Ganc  Upp2  Papss2  Ugt1a6b  Extl2  Pcyt2  Enpp3  Atp5j  Khk  Ugt1a9  Nmnat3  Nnt  Gk  Pfkfb2  Bpnt1  Idh1  Dhrs3  Sc5d  St3gal4  Mgat1  Uqcr11  Adk  Echdc1  Kynu  Gstm4  Ugt1a7c  Xylb  Aspdh  Lipc  Mgst1  Haao  Smpd4  Gba2  Entpd5  Ndufa5  Acox2  Acox1  Glyctk  Csad  Tyms  Ugt2b36  Mtmr4  Bdh2  Mtmr2  Cbr4  Man1a  Comt  Agxt2  Mri1  Mccc2  Acp5  Dpagt1  Acp1  St3gal5  St6gal1  Hagh  Pomgnt2  Vkorc1l1  P4ha1  Ggt6  Cyp4a31  Flad1  Cbs  Elovl5  Cox7a2  Grhpr  Uckl1  Baat  Fbp1  Cyp2c40  Pi4ka  Pnpla2  Gba  Mocs2  Mocs1  Ndufs6  Gls2  Hoga1  Acacb  Hyal1  Gmppa  Ndufb2  Alas1  Aldoa  Ntpcr  Mgll  Gsta2  Aprt | web link | no local link |
| Riboflavin metabolism | KEGG PATHWAY | mmu00740 | 5 (1.38%) | 8 (0.09%) | 6.54276123369e-06 | 0.000951971759501 | Blvrb  Enpp3  Flad1  Acp1  Acp5 | web link | no local link |
| Porphyrin and chlorophyll metabolism | KEGG PATHWAY | mmu00860 | 8 (2.20%) | 41 (0.48%) | 0.000260451685847 | 0.0252638135271 | Alas1  Fxn  Ugt1a6b  Ugt1a7c  Ugt1a9  Ugt2b36  Blvrb  Cp | web link | no local link |
| Drug metabolism - cytochrome P450 | KEGG PATHWAY | mmu00982 | 10 (2.75%) | 68 (0.79%) | 0.000513454983297 | 0.0373538500348 | Aox3  Ugt1a6b  Mgst1  Fmo5  Gstm4  Gsta2  Maob  Ugt1a7c  Ugt1a9  Ugt2b36 | web link | no local link |
| Drug metabolism - other enzymes | KEGG PATHWAY | mmu00983 | 11 (3.03%) | 87 (1.01%) | 0.00101392941172 | 0.0590106917622 | Upp2  Nme6  Uckl1  Gstm4  Gsta2  Ugt1a6b  Mgst1  Ugt1a9  Ugt1a7c  Ugt2b36  Ces1f | web link | no local link |
| Protein processing in endoplasmic reticulum | KEGG PATHWAY | mmu04141 | 16 (4.41%) | 163 (1.89%) | 0.00138744509999 | 0.0672910873497 | Fbxo6  Sec23b  Derl1  Dnajc5  Man1a  Ube2d3  Sec31b  Dnajb12  Derl2  Ube2g2  Sec24c  Sec24b  Mapk9  Atxn3  Sec61a2  Uggt1 | web link | no local link |
| Ubiquitin mediated proteolysis | KEGG PATHWAY | mmu04120 | 14 (3.86%) | 139 (1.61%) | 0.00213336132512 | 0.0886868779443 | Smurf1  Ubox5  Huwe1  Btrc  Anapc10  Ube2d3  Nedd4l  Xiap  Ube2g2  Rhobtb1  Ddb2  Ube2i  Ddb1  Anapc5 | web link | no local link |
| Autophagy - animal | KEGG PATHWAY | mmu04140 | 13 (3.58%) | 130 (1.51%) | 0.00323463532392 | 0.112492175867 | Bcl2l1  Mtmr14  Mapk9  Rragc  Atg16l1  Atg16l2  Stk11  Rps6kb2  Cflar  Ulk1  Akt1s1  Atg4d  Mtmr4 | web link | no local link |
| PPAR signaling pathway | KEGG PATHWAY | mmu03320 | 10 (2.75%) | 87 (1.01%) | 0.00347913945979 | 0.112492175867 | Dbi  Acox2  Acox1  Angptl4  Apoc3  Ubc  Sorbs1  Cyp4a31  Gk  Nr1h3 | web link | no local link |
| Glycine, serine and threonine metabolism | KEGG PATHWAY | mmu00260 | 6 (1.65%) | 40 (0.46%) | 0.00614628180648 | 0.178856800569 | Grhpr  Cbs  Glyctk  Agxt2  Maob  Alas1 | web link | no local link |
| Ferroptosis | KEGG PATHWAY | mmu04216 | 6 (1.65%) | 41 (0.48%) | 0.00695048433781 | 0.183871903846 | Slc40a1  Trf  Slc39a8  Pcbp2  Lpcat3  Cp | web link | no local link |
| Taurine and hypotaurine metabolism | KEGG PATHWAY | mmu00430 | 3 (0.83%) | 11 (0.13%) | 0.00953050460843 | 0.204083531986 | Ggt6  Baat  Csad | web link | no local link |
| Sulfur metabolism | KEGG PATHWAY | mmu00920 | 3 (0.83%) | 11 (0.13%) | 0.00953050460843 | 0.204083531986 | Mpst  Papss2  Bpnt1 | web link | no local link |
| Hedgehog signaling pathway | KEGG PATHWAY | mmu04340 | 6 (1.65%) | 44 (0.51%) | 0.00981845171068 | 0.204083531986 | Smurf1  Csnk1a1  Btrc  Csnk1g2  Csnk1g3  Arrb2 | web link | no local link |
| Steroid hormone biosynthesis | KEGG PATHWAY | mmu00140 | 9 (2.48%) | 89 (1.03%) | 0.0124073312352 | 0.23964212427 | Cyp2d10  Comt  Ugt1a6b  Ugt1a7c  Ugt1a9  Cyp2c40  Ugt2b36  Hsd17b2  Hsd17b6 | web link | no local link |
| Pentose and glucuronate interconversions | KEGG PATHWAY | mmu00040 | 5 (1.38%) | 34 (0.39%) | 0.0131761992726 | 0.23964212427 | Ugt2b36  Ugt1a6b  Xylb  Ugt1a9  Ugt1a7c | web link | no local link |
| Retinol metabolism | KEGG PATHWAY | mmu00830 | 9 (2.48%) | 91 (1.06%) | 0.0142214279912 | 0.240160407988 | Aox3  Dhrs3  Ugt1a6b  Ugt1a7c  Ugt1a9  Cyp2c40  Ugt2b36  Cyp4a31  Hsd17b6 | web link | no local link |
| Fructose and mannose metabolism | KEGG PATHWAY | mmu00051 | 5 (1.38%) | 35 (0.41%) | 0.0148552829683 | 0.240160407988 | Aldoa  Pfkfb2  Khk  Gmppa  Fbp1 | web link | no local link |
| Nicotinate and nicotinamide metabolism | KEGG PATHWAY | mmu00760 | 5 (1.38%) | 36 (0.42%) | 0.016671858412 | 0.249808902671 | Nmnat3  Aox3  Enpp3  Nnt  Aspdh | web link | no local link |
| AMPK signaling pathway | KEGG PATHWAY | mmu04152 | 11 (3.03%) | 126 (1.46%) | 0.0171689967472 | 0.249808902671 | Ulk1  Acacb  Ppp2r2d  Stk11  Ppp2r3d  Rps6kb2  Akt1s1  Fbp1  Pfkfb2  Srebf1  Ppp2r5c | web link | no local link |
| mRNA surveillance pathway | KEGG PATHWAY | mmu03015 | 9 (2.48%) | 96 (1.12%) | 0.0196065812051 | 0.271691196699 | Dazap1  Smg7  Ppp2r2d  Ppp2r3d  Upf3b  Cpsf7  Cpsf6  Cpsf1  Ppp2r5c | web link | no local link |
| Metabolism of xenobiotics by cytochrome P450 | KEGG PATHWAY | mmu00980 | 7 (1.93%) | 66 (0.77%) | 0.0205693095781 | 0.272075867602 | Mgst1  Gstm4  Gsta2  Ugt1a6b  Ugt1a7c  Ugt1a9  Ugt2b36 | web link | no local link |
| Peroxisome | KEGG PATHWAY | mmu04146 | 8 (2.20%) | 84 (0.98%) | 0.0248290689582 | 0.307156563443 | Idh1  Acox2  Crot  Acox1  Baat  Paox  Mpv17l2  Abcd4 | web link | no local link |
| Ascorbate and aldarate metabolism | KEGG PATHWAY | mmu00053 | 4 (1.10%) | 27 (0.31%) | 0.0253325000778 | 0.307156563443 | Ugt2b36  Ugt1a6b  Ugt1a7c  Ugt1a9 | web link | no local link |
| Pyrimidine metabolism | KEGG PATHWAY | mmu00240 | 6 (1.65%) | 58 (0.67%) | 0.034535451903 | 0.401992660151 | Upp2  Nme6  Uckl1  Entpd5  Enpp3  Tyms | web link | no local link |
| Other glycan degradation | KEGG PATHWAY | mmu00511 | 3 (0.83%) | 18 (0.21%) | 0.0379083922789 | 0.41096198942 | Gba2  Man2c1  Gba | web link | no local link |
| Pancreatic cancer | KEGG PATHWAY | mmu05212 | 7 (1.93%) | 75 (0.87%) | 0.0381304938637 | 0.41096198942 | Bcl2l1  Ralbp1  Stat1  Rps6kb2  Vegfa  Ddb2  Mapk9 | web link | no local link |
| Sulfur relay system | KEGG PATHWAY | mmu04122 | 2 (0.55%) | 8 (0.09%) | 0.0419540313832 | 0.416104710011 | Mocs2  Mpst | web link | no local link |
| Steroid biosynthesis | KEGG PATHWAY | mmu00100 | 3 (0.83%) | 19 (0.22%) | 0.0436499564852 | 0.416104710011 | Sc5d  Cyp2r1  Tm7sf2 | web link | no local link |
| Pentose phosphate pathway | KEGG PATHWAY | mmu00030 | 4 (1.10%) | 32 (0.37%) | 0.0440835933657 | 0.416104710011 | Aldoa  Glyctk  Fbp1  Idnk | web link | no local link |
| Chemical carcinogenesis | KEGG PATHWAY | mmu05204 | 8 (2.20%) | 94 (1.09%) | 0.0443273058775 | 0.416104710011 | Mgst1  Gstm4  Gsta2  Ugt1a6b  Ugt1a7c  Ugt1a9  Cyp2c40  Ugt2b36 | web link | no local link |
| Thermogenesis | KEGG PATHWAY | mmu04714 | 15 (4.13%) | 232 (2.70%) | 0.0656862510691 | 0.59733434566 | Ndufa5  Cox19  Uqcr11  Cox7a2  Pnpla2  Ndufb2  Rps6kb2  Atp5j  Ndufs2  Akt1s1  Rps6ka3  Ndufs6  Smarca2  Mgll  Atf2 | web link | no local link |
| Adherens junction | KEGG PATHWAY | mmu04520 | 6 (1.65%) | 72 (0.84%) | 0.0823737954521 | 0.72638710535 | Actn1  Pard3  Acp1  Tcf7l2  Nectin3  Sorbs1 | web link | no local link |
| Inositol phosphate metabolism | KEGG PATHWAY | mmu00562 | 6 (1.65%) | 73 (0.85%) | 0.0867426168205 | 0.742414749846 | Mtmr14  Pi4ka  Pip5k1c  Inpp4a  Mtmr4  Mtmr2 | web link | no local link |
| Tight junction | KEGG PATHWAY | mmu04530 | 11 (3.03%) | 167 (1.94%) | 0.0948515407776 | 0.788622810466 | Actn1  Cttn  Igsf5  Stk11  Pard6a  Myl6  Nedd4l  Pard3  Ppp2r2d  Mapk9  Mpdz | web link | no local link |
| Non-alcoholic fatty liver disease (NAFLD) | KEGG PATHWAY | mmu04932 | 10 (2.75%) | 151 (1.75%) | 0.105011298256 | 0.797446119474 | Ndufa5  Mlxipl  Uqcr11  Ndufb2  Ndufs2  Srebf1  Ndufs6  Cox7a2  Mapk9  Nr1h3 | web link | no local link |
| Nucleotide excision repair | KEGG PATHWAY | mmu03420 | 4 (1.10%) | 43 (0.50%) | 0.106158134381 | 0.797446119474 | Ddb2  Ddb1  Ccnh  Ercc5 | web link | no local link |
| Glycerolipid metabolism | KEGG PATHWAY | mmu00561 | 5 (1.38%) | 61 (0.71%) | 0.113640517601 | 0.797446119474 | Gk  Pnpla2  Lipc  Mgll  Glyctk | web link | no local link |
| Dopaminergic synapse | KEGG PATHWAY | mmu04728 | 9 (2.48%) | 135 (1.57%) | 0.116418686911 | 0.797446119474 | Mapk9  Ppp2r2d  Comt  Ppp2r3d  Maob  Arrb2  Gngt1  Atf2  Ppp2r5c | web link | no local link |
| Phosphatidylinositol signaling system | KEGG PATHWAY | mmu04070 | 7 (1.93%) | 98 (1.14%) | 0.119240101809 | 0.797446119474 | Mtmr14  Pi4ka  Pip5k1c  Pip4p2  Inpp4a  Mtmr4  Mtmr2 | web link | no local link |
| Purine metabolism | KEGG PATHWAY | mmu00230 | 9 (2.48%) | 136 (1.58%) | 0.120332519133 | 0.797446119474 | Ntpcr  Nudt9  Nme6  Papss2  Uox  Entpd5  Enpp3  Aprt  Adk | web link | no local link |
| Thiamine metabolism | KEGG PATHWAY | mmu00730 | 2 (0.55%) | 15 (0.17%) | 0.129840199966 | 0.797446119474 | Ntpcr  Acp1 | web link | no local link |
| Circadian rhythm | KEGG PATHWAY | mmu04710 | 3 (0.83%) | 30 (0.35%) | 0.130960964139 | 0.797446119474 | Per1  Btrc  Rora | web link | no local link |
| Glutathione metabolism | KEGG PATHWAY | mmu00480 | 5 (1.38%) | 64 (0.74%) | 0.131954810798 | 0.797446119474 | Gstm4  Idh1  Mgst1  Ggt6  Gsta2 | web link | no local link |
| Mineral absorption | KEGG PATHWAY | mmu04978 | 4 (1.10%) | 47 (0.55%) | 0.135132370844 | 0.797446119474 | Mt2  Clcn2  Slc40a1  Trf | web link | no local link |
| Insulin signaling pathway | KEGG PATHWAY | mmu04910 | 9 (2.48%) | 140 (1.63%) | 0.136690280876 | 0.797446119474 | Exoc7  Acacb  Rps6kb2  Srebf1  Fbp1  Phkb  Sorbs1  Mapk9  Prkar1a | web link | no local link |
| RNA degradation | KEGG PATHWAY | mmu03018 | 6 (1.65%) | 83 (0.96%) | 0.137091719616 | 0.797446119474 | Pan3  Exosc10  Cnot10  Edc4  Cnot2  Zcchc7 | web link | no local link |
| Glyoxylate and dicarboxylate metabolism | KEGG PATHWAY | mmu00630 | 3 (0.83%) | 31 (0.36%) | 0.140722888485 | 0.797446119474 | Hoga1  Grhpr  Glyctk | web link | no local link |
| Sphingolipid metabolism | KEGG PATHWAY | mmu00600 | 4 (1.10%) | 48 (0.56%) | 0.142832901117 | 0.797446119474 | Smpd4  Gba2  Cers2  Gba | web link | no local link |
| Tryptophan metabolism | KEGG PATHWAY | mmu00380 | 4 (1.10%) | 48 (0.56%) | 0.142832901117 | 0.797446119474 | Aox3  Maob  Kynu  Haao | web link | no local link |
| Primary bile acid biosynthesis | KEGG PATHWAY | mmu00120 | 2 (0.55%) | 16 (0.19%) | 0.144435114845 | 0.797446119474 | Acox2  Baat | web link | no local link |
| Amino sugar and nucleotide sugar metabolism | KEGG PATHWAY | mmu00520 | 4 (1.10%) | 49 (0.57%) | 0.150702315294 | 0.797446119474 | Gmppa  Cmah  Cyb5r3  Gne | web link | no local link |
| Cholesterol metabolism | KEGG PATHWAY | mmu04979 | 4 (1.10%) | 49 (0.57%) | 0.150702315294 | 0.797446119474 | Gm44805  Angptl4  Lipc  Apoc3 | web link | no local link |
| Apoptosis - multiple species | KEGG PATHWAY | mmu04215 | 3 (0.83%) | 32 (0.37%) | 0.150720056945 | 0.797446119474 | Bcl2l1  Xiap  Mapk9 | web link | no local link |
| Biosynthesis of unsaturated fatty acids | KEGG PATHWAY | mmu01040 | 3 (0.83%) | 32 (0.37%) | 0.150720056945 | 0.797446119474 | Acox1  Elovl5  Baat | web link | no local link |
| N-Glycan biosynthesis | KEGG PATHWAY | mmu00510 | 4 (1.10%) | 50 (0.58%) | 0.158733349538 | 0.807455104281 | Mgat1  Dpagt1  Man1a  St6gal1 | web link | no local link |
| Arginine and proline metabolism | KEGG PATHWAY | mmu00330 | 4 (1.10%) | 50 (0.58%) | 0.158733349538 | 0.807455104281 | Maob  Hoga1  P4ha1  L3hypdh | web link | no local link |
| Propanoate metabolism | KEGG PATHWAY | mmu00640 | 3 (0.83%) | 33 (0.38%) | 0.160936068895 | 0.807455104281 | Acox1  Echdc1  Acacb | web link | no local link |
| Pantothenate and CoA biosynthesis | KEGG PATHWAY | mmu00770 | 2 (0.55%) | 18 (0.21%) | 0.174528992089 | 0.849965274025 | Enpp3  Aasdhppt | web link | no local link |
| Cysteine and methionine metabolism | KEGG PATHWAY | mmu00270 | 4 (1.10%) | 52 (0.60%) | 0.175250571964 | 0.849965274025 | Cbs  Mpst  Mri1  Agxt2 | web link | no local link |
| Insulin resistance | KEGG PATHWAY | mmu04931 | 7 (1.93%) | 110 (1.28%) | 0.181920731421 | 0.85028644578 | Rps6ka3  Acacb  Mlxipl  Rps6kb2  Srebf1  Mapk9  Nr1h3 | web link | no local link |
| DNA replication | KEGG PATHWAY | mmu03030 | 3 (0.83%) | 35 (0.41%) | 0.181959530087 | 0.85028644578 | Ssbp1  Dna2  Rnaseh2a | web link | no local link |
| Ribosome | KEGG PATHWAY | mmu03010 | 10 (2.75%) | 171 (1.99%) | 0.18536119834 | 0.85028644578 | Mrpl36  Rpl22l1  Rps10  Mrpl30  Mrpl24  Rps19  Rpl35a  Rpl15  Uba52  Rpl18 | web link | no local link |
| Fatty acid biosynthesis | KEGG PATHWAY | mmu00061 | 2 (0.55%) | 19 (0.22%) | 0.18992652569 | 0.85028644578 | Cbr4  Acacb | web link | no local link |
| One carbon pool by folate | KEGG PATHWAY | mmu00670 | 2 (0.55%) | 19 (0.22%) | 0.18992652569 | 0.85028644578 | Tyms  Mthfs | web link | no local link |
| TGF-beta signaling pathway | KEGG PATHWAY | mmu04350 | 6 (1.65%) | 95 (1.10%) | 0.211507523911 | 0.932555900881 | Smurf1  Dcn  Acvr2b  Rps6kb2  Chrd  Tfdp1 | web link | no local link |
| Pyruvate metabolism | KEGG PATHWAY | mmu00620 | 3 (0.83%) | 38 (0.44%) | 0.214734136588 | 0.932651249957 | Hagh  Grhpr  Acacb | web link | no local link |
| Platinum drug resistance | KEGG PATHWAY | mmu01524 | 5 (1.38%) | 77 (0.89%) | 0.22418464168 | 0.93289223511 | Bcl2l1  Xiap  Mgst1  Gstm4  Gsta2 | web link | no local link |
| Glycerophospholipid metabolism | KEGG PATHWAY | mmu00564 | 6 (1.65%) | 97 (1.13%) | 0.225105292033 | 0.93289223511 | Ptdss1  Pcyt2  Taz  Lpcat3  Gpcpd1  Pnpla6 | web link | no local link |
| Phosphonate and phosphinate metabolism | KEGG PATHWAY | mmu00440 | 1 (0.28%) | 6 (0.07%) | 0.227858176954 | 0.93289223511 | Pcyt2 | web link | no local link |
| Caffeine metabolism | KEGG PATHWAY | mmu00232 | 1 (0.28%) | 6 (0.07%) | 0.227858176954 | 0.93289223511 | Uox | web link | no local link |
| Oocyte meiosis | KEGG PATHWAY | mmu04114 | 7 (1.93%) | 119 (1.38%) | 0.236262870437 | 0.93289223511 | Ywhaz  Rps6ka3  Btrc  Anapc10  Cpeb3  Anapc5  Ppp2r5c | web link | no local link |
| Proximal tubule bicarbonate reclamation | KEGG PATHWAY | mmu04964 | 2 (0.55%) | 22 (0.26%) | 0.236989059137 | 0.93289223511 | Gls2  Slc38a3 | web link | no local link |
| Tyrosine metabolism | KEGG PATHWAY | mmu00350 | 3 (0.83%) | 40 (0.46%) | 0.237230327829 | 0.93289223511 | Maob  Aox3  Comt | web link | no local link |
| Neurotrophin signaling pathway | KEGG PATHWAY | mmu04722 | 7 (1.93%) | 121 (1.41%) | 0.249016057299 | 0.966182302321 | Irak1  Kidins220  Rps6ka3  Arhgdib  Abl1  Mapk9  Mapkapk2 | web link | no local link |
| Cell cycle | KEGG PATHWAY | mmu04110 | 7 (1.93%) | 123 (1.43%) | 0.261975136067 | 0.990382225387 | Ywhaz  Ccnh  Orc4  Anapc10  Abl1  Anapc5  Tfdp1 | web link | no local link |
| Parkinson disease | KEGG PATHWAY | mmu05012 | 8 (2.20%) | 144 (1.67%) | 0.262059901563 | 0.990382225387 | Ndufa5  Uqcr11  Ndufb2  Atp5j  Ndufs2  Ube2g2  Ndufs6  Cox7a2 | web link | no local link |
| Mitophagy - animal | KEGG PATHWAY | mmu04137 | 4 (1.10%) | 63 (0.73%) | 0.274393403707 | 1.0 | Bcl2l1  Ulk1  Mapk9  Tbc1d17 | web link | no local link |
| Basal transcription factors | KEGG PATHWAY | mmu03022 | 3 (0.83%) | 44 (0.51%) | 0.283253397929 | 1.0 | Gtf2i  Ccnh  Gtf2a2 | web link | no local link |
| Glycosylphosphatidylinositol (GPI)-anchor biosynthesis | KEGG PATHWAY | mmu00563 | 2 (0.55%) | 25 (0.29%) | 0.284576175014 | 1.0 | Pigp  Pigv | web link | no local link |
| Hepatocellular carcinoma | KEGG PATHWAY | mmu05225 | 9 (2.48%) | 171 (1.99%) | 0.295347080352 | 1.0 | Bcl2l1  Csnk1a1  Gstm4  Gsta2  Rps6kb2  Mgst1  Tcf7l2  Ddb2  Smarca2 | web link | no local link |
| Folate biosynthesis | KEGG PATHWAY | mmu00790 | 2 (0.55%) | 26 (0.30%) | 0.300408103301 | 1.0 | Mocs2  Mocs1 | web link | no local link |
| Vitamin B6 metabolism | KEGG PATHWAY | mmu00750 | 1 (0.28%) | 9 (0.10%) | 0.321553516204 | 1.0 | Aox3 | web link | no local link |
| Hippo signaling pathway | KEGG PATHWAY | mmu04390 | 8 (2.20%) | 154 (1.79%) | 0.324014842819 | 1.0 | Ywhaz  Pard3  Ppp2r2d  Btrc  Tcf7l2  Pard6a  Fgf1  Yap1 | web link | no local link |
| Spliceosome | KEGG PATHWAY | mmu03040 | 7 (1.93%) | 133 (1.55%) | 0.329180242701 | 1.0 | Srsf3  U2af1  Snrpa1  Hnrnpk  Hnrnpm  Txnl4a  Snrnp70 | web link | no local link |
| Progesterone-mediated oocyte maturation | KEGG PATHWAY | mmu04914 | 5 (1.38%) | 90 (1.05%) | 0.330086799429 | 1.0 | Mapk9  Rps6ka3  Anapc5  Anapc10  Cpeb3 | web link | no local link |
| Longevity regulating pathway | KEGG PATHWAY | mmu04211 | 5 (1.38%) | 90 (1.05%) | 0.330086799429 | 1.0 | Rps6kb2  Akt1s1  Stk11  Ulk1  Atf2 | web link | no local link |
| Focal adhesion | KEGG PATHWAY | mmu04510 | 10 (2.75%) | 199 (2.31%) | 0.330288587463 | 1.0 | Actn1  Fn1  Pip5k1c  Itgav  Xiap  Vegfa  Mylpf  Arhgap5  Mapk9  Zyx | web link | no local link |
| mTOR signaling pathway | KEGG PATHWAY | mmu04150 | 8 (2.20%) | 155 (1.80%) | 0.330368222025 | 1.0 | Rps6ka3  Sgk1  Rragc  Stk11  Rps6kb2  Ulk1  Akt1s1  Lamtor2 | web link | no local link |
| Oxidative phosphorylation | KEGG PATHWAY | mmu00190 | 7 (1.93%) | 134 (1.56%) | 0.336063578861 | 1.0 | Ndufa5  Uqcr11  Ndufb2  Atp5j  Ndufs2  Ndufs6  Cox7a2 | web link | no local link |
| IL-17 signaling pathway | KEGG PATHWAY | mmu04657 | 5 (1.38%) | 91 (1.06%) | 0.338507982331 | 1.0 | S100a9  Mapk15  Mapk9  Mapk6  Anapc5 | web link | no local link |
| PI3K-Akt signaling pathway | KEGG PATHWAY | mmu04151 | 17 (4.68%) | 358 (4.16%) | 0.341283148316 | 1.0 | Bcl2l1  Ywhaz  Fn1  Sgk1  Ghr  Prlr  Ppp2r2d  Stk11  Itgav  Rps6kb2  Vegfa  Ppp2r3d  Gngt1  Ppp2r5c  Fgfr2  Fgf1  Atf2 | web link | no local link |
| Small cell lung cancer | KEGG PATHWAY | mmu05222 | 5 (1.38%) | 92 (1.07%) | 0.346946098997 | 1.0 | Ddb2  Bcl2l1  Xiap  Fn1  Itgav | web link | no local link |
| Apoptosis | KEGG PATHWAY | mmu04210 | 7 (1.93%) | 136 (1.58%) | 0.349887649363 | 1.0 | Bcl2l1  Dffa  Parp3  Cflar  Xiap  Lmna  Mapk9 | web link | no local link |
| Endocytosis | KEGG PATHWAY | mmu04144 | 13 (3.58%) | 270 (3.14%) | 0.351437968316 | 1.0 | Smurf1  Washc3  Pard3  Arap1  Pip5k1c  Fgfr2  Pard6a  Nedd4l  Agap3  Arrb2  Arpc5  Chmp3  Bin1 | web link | no local link |
| MAPK signaling pathway | KEGG PATHWAY | mmu04010 | 14 (3.86%) | 294 (3.42%) | 0.358388847225 | 1.0 | Mapk9  Irak1  Map4k2  Ppp5c  Rps6ka3  Vegfa  Fgf1  Arrb2  Mapkapk5  Dusp16  Fgfr2  Mapkapk2  Dusp3  Atf2 | web link | no local link |
| Prolactin signaling pathway | KEGG PATHWAY | mmu04917 | 4 (1.10%) | 72 (0.84%) | 0.361023629529 | 1.0 | Prlr  Mapk9  Stat1  Esr1 | web link | no local link |
| Ubiquinone and other terpenoid-quinone biosynthesis | KEGG PATHWAY | mmu00130 | 1 (0.28%) | 11 (0.13%) | 0.377627854076 | 1.0 | Vkorc1l1 | web link | no local link |
| Synthesis and degradation of ketone bodies | KEGG PATHWAY | mmu00072 | 1 (0.28%) | 11 (0.13%) | 0.377627854076 | 1.0 | Bdh2 | web link | no local link |
| Thyroid hormone signaling pathway | KEGG PATHWAY | mmu04919 | 6 (1.65%) | 118 (1.37%) | 0.379636888608 | 1.0 | Stat1  Esr1  Pfkfb2  Itgav  Dio1  Ncor1 | web link | no local link |
| Bacterial invasion of epithelial cells | KEGG PATHWAY | mmu05100 | 4 (1.10%) | 74 (0.86%) | 0.380392801195 | 1.0 | Cttn  Arpc5  Fn1  Elmo3 | web link | no local link |
| Autophagy - other | KEGG PATHWAY | mmu04136 | 2 (0.55%) | 32 (0.37%) | 0.393414013945 | 1.0 | Atg16l1  Atg4d | web link | no local link |
| Fluid shear stress and atherosclerosis | KEGG PATHWAY | mmu05418 | 7 (1.93%) | 143 (1.66%) | 0.398628761052 | 1.0 | Acvr2b  Gstm4  Gsta2  Mgst1  Vegfa  Itgav  Mapk9 | web link | no local link |
| Carbon metabolism | KEGG PATHWAY | mmu01200 | 6 (1.65%) | 121 (1.41%) | 0.402471510445 | 1.0 | Idh1  Acox1  Glyctk  Fbp1  Idnk  Aldoa | web link | no local link |
| Starch and sucrose metabolism | KEGG PATHWAY | mmu00500 | 2 (0.55%) | 33 (0.38%) | 0.408412591045 | 1.0 | Ganc  Enpp3 | web link | no local link |
| SNARE interactions in vesicular transport | KEGG PATHWAY | mmu04130 | 2 (0.55%) | 33 (0.38%) | 0.408412591045 | 1.0 | Bet1  Snap23 | web link | no local link |
| Yersinia infection | KEGG PATHWAY | mmu05135 | 6 (1.65%) | 123 (1.43%) | 0.417669845986 | 1.0 | Arhgef12  Mapk9  Fn1  Pip5k1c  Rps6ka3  Irak1 | web link | no local link |
| Valine, leucine and isoleucine degradation | KEGG PATHWAY | mmu00280 | 3 (0.83%) | 56 (0.65%) | 0.422520368906 | 1.0 | Aox3  Mccc2  Agxt2 | web link | no local link |
| Sphingolipid signaling pathway | KEGG PATHWAY | mmu04071 | 6 (1.65%) | 124 (1.44%) | 0.425253437782 | 1.0 | Cers2  Ppp2r2d  Ppp2r3d  Gna13  Mapk9  Ppp2r5c | web link | no local link |
| Non-homologous end-joining | KEGG PATHWAY | mmu03450 | 1 (0.28%) | 13 (0.15%) | 0.42907929657 | 1.0 | Dclre1c | web link | no local link |
| Regulation of actin cytoskeleton | KEGG PATHWAY | mmu04810 | 10 (2.75%) | 217 (2.52%) | 0.433059858594 | 1.0 | Arhgef12  Arpc5  Fn1  Pip5k1c  Actn1  Itgav  Gna13  Fgfr2  Fgf1  Mylpf | web link | no local link |
| Base excision repair | KEGG PATHWAY | mmu03410 | 2 (0.55%) | 35 (0.41%) | 0.437851089428 | 1.0 | Apex1  Parp3 | web link | no local link |
| EGFR tyrosine kinase inhibitor resistance | KEGG PATHWAY | mmu01521 | 4 (1.10%) | 80 (0.93%) | 0.437962947807 | 1.0 | Bcl2l1  Fgfr2  Vegfa  Rps6kb2 | web link | no local link |
| Retrograde endocannabinoid signaling | KEGG PATHWAY | mmu04723 | 7 (1.93%) | 150 (1.74%) | 0.44734116982 | 1.0 | Ndufa5  Ndufs6  Mgll  Ndufb2  Ndufs2  Gngt1  Mapk9 | web link | no local link |
| Primary immunodeficiency | KEGG PATHWAY | mmu05340 | 2 (0.55%) | 36 (0.42%) | 0.452269691506 | 1.0 | Dclre1c  Rfxap | web link | no local link |
| Renin-angiotensin system | KEGG PATHWAY | mmu04614 | 2 (0.55%) | 36 (0.42%) | 0.452269691506 | 1.0 | Ctsa  Nln | web link | no local link |
| Glucagon signaling pathway | KEGG PATHWAY | mmu04922 | 5 (1.38%) | 105 (1.22%) | 0.456366613692 | 1.0 | Ppp4r3a  Acacb  Fbp1  Phkb  Atf2 | web link | no local link |
| Thyroid cancer | KEGG PATHWAY | mmu05216 | 2 (0.55%) | 37 (0.43%) | 0.466475681438 | 1.0 | Ddb2  Tcf7l2 | web link | no local link |
| Glycosphingolipid biosynthesis - ganglio series | KEGG PATHWAY | mmu00604 | 1 (0.28%) | 15 (0.17%) | 0.476287999848 | 1.0 | St3gal5 | web link | no local link |
| Fatty acid metabolism | KEGG PATHWAY | mmu01212 | 3 (0.83%) | 61 (0.71%) | 0.478212453305 | 1.0 | Acox1  Cbr4  Elovl5 | web link | no local link |
| Aldosterone-regulated sodium reabsorption | KEGG PATHWAY | mmu04960 | 2 (0.55%) | 38 (0.44%) | 0.480461322819 | 1.0 | Nedd4l  Sgk1 | web link | no local link |
| Alanine, aspartate and glutamate metabolism | KEGG PATHWAY | mmu00250 | 2 (0.55%) | 38 (0.44%) | 0.480461322819 | 1.0 | Gls2  Agxt2 | web link | no local link |
| NF-kappa B signaling pathway | KEGG PATHWAY | mmu04064 | 5 (1.38%) | 108 (1.25%) | 0.48105603171 | 1.0 | Bcl2l1  Cflar  Xiap  Irak1  Ube2i | web link | no local link |
| Toxoplasmosis | KEGG PATHWAY | mmu05145 | 5 (1.38%) | 108 (1.25%) | 0.48105603171 | 1.0 | Bcl2l1  Mapk9  Irak1  Xiap  Stat1 | web link | no local link |
| Longevity regulating pathway - multiple species | KEGG PATHWAY | mmu04213 | 3 (0.83%) | 62 (0.72%) | 0.489056150869 | 1.0 | Clpb  Akt1s1  Rps6kb2 | web link | no local link |
| Axon guidance | KEGG PATHWAY | mmu04360 | 8 (2.20%) | 180 (2.09%) | 0.491303964107 | 1.0 | Plxnb2  Arhgef12  Pard3  Ablim2  Pard6a  Rgs3  Abl1  Sema4g | web link | no local link |
| Colorectal cancer | KEGG PATHWAY | mmu05210 | 4 (1.10%) | 88 (1.02%) | 0.512057334106 | 1.0 | Ddb2  Mapk9  Tcf7l2  Rps6kb2 | web link | no local link |
| Selenocompound metabolism | KEGG PATHWAY | mmu00450 | 1 (0.28%) | 17 (0.20%) | 0.519602938019 | 1.0 | Papss2 | web link | no local link |
| Hepatitis B | KEGG PATHWAY | mmu05161 | 7 (1.93%) | 163 (1.89%) | 0.535399825876 | 1.0 | Ywhaz  Irak1  Stat1  Ddb1  Ddb2  Mapk9  Atf2 | web link | no local link |
| Fc gamma R-mediated phagocytosis | KEGG PATHWAY | mmu04666 | 4 (1.10%) | 92 (1.07%) | 0.547411733633 | 1.0 | Pip5k1c  Bin1  Arpc5  Rps6kb2 | web link | no local link |
| Jak-STAT signaling pathway | KEGG PATHWAY | mmu04630 | 7 (1.93%) | 165 (1.92%) | 0.548481733271 | 1.0 | Bcl2l1  Aox3  Ghr  Stat1  Prlr  Thpo  Lifr | web link | no local link |
| Endocrine resistance | KEGG PATHWAY | mmu01522 | 4 (1.10%) | 93 (1.08%) | 0.55604161305 | 1.0 | Mapk9  Rps6kb2  Ncor1  Esr1 | web link | no local link |
| Arginine biosynthesis | KEGG PATHWAY | mmu00220 | 1 (0.28%) | 19 (0.22%) | 0.559344454301 | 1.0 | Gls2 | web link | no local link |
| 2-Oxocarboxylic acid metabolism | KEGG PATHWAY | mmu01210 | 1 (0.28%) | 19 (0.22%) | 0.559344454301 | 1.0 | Idh1 | web link | no local link |
| RNA transport | KEGG PATHWAY | mmu03013 | 7 (1.93%) | 167 (1.94%) | 0.561406625713 | 1.0 | Ube2i  Gemin7  Nup98  Eif5  Eif3e  Upf3b  Thoc7 | web link | no local link |
| p53 signaling pathway | KEGG PATHWAY | mmu04115 | 3 (0.83%) | 71 (0.82%) | 0.581198834156 | 1.0 | Ddb2  Bcl2l1  Mdm4 | web link | no local link |
| Adipocytokine signaling pathway | KEGG PATHWAY | mmu04920 | 3 (0.83%) | 71 (0.82%) | 0.581198834156 | 1.0 | Mapk9  Stk11  Acacb | web link | no local link |
| Pathways in cancer | KEGG PATHWAY | mmu05200 | 22 (6.06%) | 535 (6.22%) | 0.582074656927 | 1.0 | Fn1  Vegfa  Bcl2l1  Ralbp1  Esr1  Itgav  Gna13  Fgfr2  Stat1  Rassf5  Gstm4  Tcf7l2  Gngt1  Abl1  Mapk9  Fgf1  Arhgef12  Gsta2  Rps6kb2  Xiap  Mgst1  Ddb2 | web link | no local link |
| Bile secretion | KEGG PATHWAY | mmu04976 | 3 (0.83%) | 72 (0.84%) | 0.590771669139 | 1.0 | Slco1b2  Baat  Slc4a2 | web link | no local link |
| Breast cancer | KEGG PATHWAY | mmu05224 | 6 (1.65%) | 147 (1.71%) | 0.591324041396 | 1.0 | Csnk1a1  Esr1  Tcf7l2  Rps6kb2  Ddb2  Fgf1 | web link | no local link |
| Glycosaminoglycan degradation | KEGG PATHWAY | mmu00531 | 1 (0.28%) | 21 (0.24%) | 0.595806603998 | 1.0 | Hyal1 | web link | no local link |
| Lysosome | KEGG PATHWAY | mmu04142 | 5 (1.38%) | 124 (1.44%) | 0.604642890498 | 1.0 | Acp5  Nagpa  Gba  Hyal1  Ctsa | web link | no local link |
| Choline metabolism in cancer | KEGG PATHWAY | mmu05231 | 4 (1.10%) | 99 (1.15%) | 0.605896811206 | 1.0 | Pip5k1c  Mapk9  Rps6kb2  Gpcpd1 | web link | no local link |
| Cocaine addiction | KEGG PATHWAY | mmu05030 | 2 (0.55%) | 48 (0.56%) | 0.607144028566 | 1.0 | Maob  Atf2 | web link | no local link |
| Gastric cancer | KEGG PATHWAY | mmu05226 | 6 (1.65%) | 150 (1.74%) | 0.61115852131 | 1.0 | Csnk1a1  Tcf7l2  Rps6kb2  Ddb2  Fgfr2  Fgf1 | web link | no local link |
| Alzheimer disease | KEGG PATHWAY | mmu05010 | 7 (1.93%) | 175 (2.03%) | 0.611348319648 | 1.0 | Ndufa5  Uqcr11  Ndufb2  Atp5j  Ndufs2  Ndufs6  Cox7a2 | web link | no local link |
| Other types of O-glycan biosynthesis | KEGG PATHWAY | mmu00514 | 1 (0.28%) | 22 (0.26%) | 0.612893153753 | 1.0 | St6gal1 | web link | no local link |
| Mismatch repair | KEGG PATHWAY | mmu03430 | 1 (0.28%) | 22 (0.26%) | 0.612893153753 | 1.0 | Ssbp1 | web link | no local link |
| AGE-RAGE signaling pathway in diabetic complications | KEGG PATHWAY | mmu04933 | 4 (1.10%) | 101 (1.17%) | 0.621740562782 | 1.0 | Mapk9  Vegfa  Fn1  Stat1 | web link | no local link |
| Inflammatory mediator regulation of TRP channels | KEGG PATHWAY | mmu04750 | 5 (1.38%) | 127 (1.48%) | 0.625892920981 | 1.0 | Asic5  P2ry2  Cyp4a31  Mapk9  Cyp2c40 | web link | no local link |
| Pertussis | KEGG PATHWAY | mmu05133 | 3 (0.83%) | 76 (0.88%) | 0.627630799741 | 1.0 | Mapk9  C4bp  Irak1 | web link | no local link |
| Chronic myeloid leukemia | KEGG PATHWAY | mmu05220 | 3 (0.83%) | 76 (0.88%) | 0.627630799741 | 1.0 | Ddb2  Bcl2l1  Abl1 | web link | no local link |
| Terpenoid backbone biosynthesis | KEGG PATHWAY | mmu00900 | 1 (0.28%) | 23 (0.27%) | 0.629259306233 | 1.0 | Icmt | web link | no local link |
| Phenylalanine metabolism | KEGG PATHWAY | mmu00360 | 1 (0.28%) | 23 (0.27%) | 0.629259306233 | 1.0 | Maob | web link | no local link |
| Mannose type O-glycan biosynthesis | KEGG PATHWAY | mmu00515 | 1 (0.28%) | 23 (0.27%) | 0.629259306233 | 1.0 | Pomgnt2 | web link | no local link |
| Fatty acid degradation | KEGG PATHWAY | mmu00071 | 2 (0.55%) | 50 (0.58%) | 0.629489024466 | 1.0 | Cyp4a31  Acox1 | web link | no local link |
| Arrhythmogenic right ventricular cardiomyopathy (ARVC) | KEGG PATHWAY | mmu05412 | 3 (0.83%) | 77 (0.89%) | 0.636481324293 | 1.0 | Lmna  Tcf7l2  Itgav | web link | no local link |
| Chagas disease (American trypanosomiasis) | KEGG PATHWAY | mmu05142 | 4 (1.10%) | 103 (1.20%) | 0.63717888092 | 1.0 | Ppp2r2d  Mapk9  Irak1  Cflar | web link | no local link |
| Fanconi anemia pathway | KEGG PATHWAY | mmu03460 | 2 (0.55%) | 51 (0.59%) | 0.640285930154 | 1.0 | Fance  Cenpx | web link | no local link |
| Histidine metabolism | KEGG PATHWAY | mmu00340 | 1 (0.28%) | 24 (0.28%) | 0.644935354204 | 1.0 | Maob | web link | no local link |
| Glycosaminoglycan biosynthesis - heparan sulfate / heparin | KEGG PATHWAY | mmu00534 | 1 (0.28%) | 24 (0.28%) | 0.644935354204 | 1.0 | Extl2 | web link | no local link |
| Biosynthesis of amino acids | KEGG PATHWAY | mmu01230 | 3 (0.83%) | 78 (0.91%) | 0.645184419639 | 1.0 | Aldoa  Cbs  Idh1 | web link | no local link |
| Proteoglycans in cancer | KEGG PATHWAY | mmu05205 | 8 (2.20%) | 206 (2.39%) | 0.645299692141 | 1.0 | Dcn  Arhgef12  Cttn  Fn1  Esr1  Itgav  Rps6kb2  Vegfa | web link | no local link |
| Amyotrophic lateral sclerosis (ALS) | KEGG PATHWAY | mmu05014 | 2 (0.55%) | 52 (0.60%) | 0.650834167596 | 1.0 | Bcl2l1  Derl1 | web link | no local link |
| Relaxin signaling pathway | KEGG PATHWAY | mmu04926 | 5 (1.38%) | 131 (1.52%) | 0.653137505896 | 1.0 | Arrb2  Mapk9  Vegfa  Gngt1  Atf2 | web link | no local link |
| Ras signaling pathway | KEGG PATHWAY | mmu04014 | 9 (2.48%) | 233 (2.71%) | 0.654710857208 | 1.0 | Bcl2l1  Ralbp1  Rassf5  Vegfa  Gngt1  Abl1  Mapk9  Fgfr2  Fgf1 | web link | no local link |
| alpha-Linolenic acid metabolism | KEGG PATHWAY | mmu00592 | 1 (0.28%) | 25 (0.29%) | 0.659950320004 | 1.0 | Acox1 | web link | no local link |
| Hepatitis C | KEGG PATHWAY | mmu05160 | 6 (1.65%) | 160 (1.86%) | 0.673238150943 | 1.0 | Ywhaz  Stat1  Ppp2r2d  Eif3e  Cflar  Nr1h3 | web link | no local link |
| Hippo signaling pathway - multiple species | KEGG PATHWAY | mmu04392 | 1 (0.28%) | 26 (0.30%) | 0.674332008672 | 1.0 | Yap1 | web link | no local link |
| Wnt signaling pathway | KEGG PATHWAY | mmu04310 | 6 (1.65%) | 162 (1.88%) | 0.684865991324 | 1.0 | Csnk1a1  Btrc  Tcf7l2  Gpc4  Mapk9  Tbl1xr1 | web link | no local link |
| Glycosphingolipid biosynthesis - lacto and neolacto series | KEGG PATHWAY | mmu00601 | 1 (0.28%) | 27 (0.31%) | 0.688107058876 | 1.0 | St3gal4 | web link | no local link |
| Collecting duct acid secretion | KEGG PATHWAY | mmu04966 | 1 (0.28%) | 27 (0.31%) | 0.688107058876 | 1.0 | Slc12a7 | web link | no local link |
| Phototransduction | KEGG PATHWAY | mmu04744 | 1 (0.28%) | 27 (0.31%) | 0.688107058876 | 1.0 | Gngt1 | web link | no local link |
| Butanoate metabolism | KEGG PATHWAY | mmu00650 | 1 (0.28%) | 27 (0.31%) | 0.688107058876 | 1.0 | Bdh2 | web link | no local link |
| Regulation of lipolysis in adipocytes | KEGG PATHWAY | mmu04923 | 2 (0.55%) | 56 (0.65%) | 0.69057897213 | 1.0 | Pnpla2  Mgll | web link | no local link |
| ErbB signaling pathway | KEGG PATHWAY | mmu04012 | 3 (0.83%) | 84 (0.98%) | 0.694290610035 | 1.0 | Abl1  Mapk9  Rps6kb2 | web link | no local link |
| Protein export | KEGG PATHWAY | mmu03060 | 1 (0.28%) | 28 (0.33%) | 0.701300991708 | 1.0 | Sec61a2 | web link | no local link |
| C-type lectin receptor signaling pathway | KEGG PATHWAY | mmu04625 | 4 (1.10%) | 112 (1.30%) | 0.701467066861 | 1.0 | Arhgef12  Mapk9  Mapkapk2  Stat1 | web link | no local link |
| VEGF signaling pathway | KEGG PATHWAY | mmu04370 | 2 (0.55%) | 58 (0.67%) | 0.709016738877 | 1.0 | Vegfa  Mapkapk2 | web link | no local link |
| Endometrial cancer | KEGG PATHWAY | mmu05213 | 2 (0.55%) | 58 (0.67%) | 0.709016738877 | 1.0 | Ddb2  Tcf7l2 | web link | no local link |
| ECM-receptor interaction | KEGG PATHWAY | mmu04512 | 3 (0.83%) | 86 (1.00%) | 0.70947649924 | 1.0 | Cd47  Fn1  Itgav | web link | no local link |
| Cardiac muscle contraction | KEGG PATHWAY | mmu04260 | 3 (0.83%) | 86 (1.00%) | 0.70947649924 | 1.0 | Uqcr11  Cox7a2  Asph | web link | no local link |
| Huntington disease | KEGG PATHWAY | mmu05016 | 7 (1.93%) | 193 (2.24%) | 0.7115695295 | 1.0 | Ndufa5  Uqcr11  Ndufb2  Atp5j  Ndufs2  Ndufs6  Cox7a2 | web link | no local link |
| Fatty acid elongation | KEGG PATHWAY | mmu00062 | 1 (0.28%) | 29 (0.34%) | 0.713938257443 | 1.0 | Elovl5 | web link | no local link |
| HIF-1 signaling pathway | KEGG PATHWAY | mmu04066 | 4 (1.10%) | 114 (1.32%) | 0.714583078403 | 1.0 | Aldoa  Trf  Vegfa  Rps6kb2 | web link | no local link |
| Inflammatory bowel disease (IBD) | KEGG PATHWAY | mmu05321 | 2 (0.55%) | 59 (0.69%) | 0.717887032754 | 1.0 | Rora  Stat1 | web link | no local link |
| Lysine degradation | KEGG PATHWAY | mmu00310 | 2 (0.55%) | 59 (0.69%) | 0.717887032754 | 1.0 | Kmt5b  Nsd1 | web link | no local link |
| Leukocyte transendothelial migration | KEGG PATHWAY | mmu04670 | 4 (1.10%) | 115 (1.34%) | 0.720981181768 | 1.0 | Arhgap5  Actn1  Rassf5  Mylpf | web link | no local link |
| Complement and coagulation cascades | KEGG PATHWAY | mmu04610 | 3 (0.83%) | 88 (1.02%) | 0.724077791549 | 1.0 | Clu  C4bp  Proc | web link | no local link |
| Antifolate resistance | KEGG PATHWAY | mmu01523 | 1 (0.28%) | 30 (0.35%) | 0.726042280354 | 1.0 | Tyms | web link | no local link |
| GABAergic synapse | KEGG PATHWAY | mmu04727 | 3 (0.83%) | 90 (1.05%) | 0.738101515852 | 1.0 | Gngt1  Slc38a3  Gls2 | web link | no local link |
| Galactose metabolism | KEGG PATHWAY | mmu00052 | 1 (0.28%) | 32 (0.37%) | 0.748739420602 | 1.0 | Ganc | web link | no local link |
| Citrate cycle (TCA cycle) | KEGG PATHWAY | mmu00020 | 1 (0.28%) | 32 (0.37%) | 0.748739420602 | 1.0 | Idh1 | web link | no local link |
| Basal cell carcinoma | KEGG PATHWAY | mmu05217 | 2 (0.55%) | 63 (0.73%) | 0.751121922729 | 1.0 | Ddb2  Tcf7l2 | web link | no local link |
| Necroptosis | KEGG PATHWAY | mmu04217 | 6 (1.65%) | 177 (2.06%) | 0.763296088275 | 1.0 | Stat1  Parp3  Cflar  Xiap  Chmp3  Mapk9 | web link | no local link |
| Viral carcinogenesis | KEGG PATHWAY | mmu05203 | 8 (2.20%) | 231 (2.68%) | 0.764901094771 | 1.0 | Ywhaz  Actn1  Hnrnpk  Gtf2a2  Hdac10  Ddb1  Mapkapk2  Atf2 | web link | no local link |
| beta-Alanine metabolism | KEGG PATHWAY | mmu00410 | 1 (0.28%) | 34 (0.39%) | 0.769560874008 | 1.0 | Acox1 | web link | no local link |
| NOD-like receptor signaling pathway | KEGG PATHWAY | mmu04621 | 7 (1.93%) | 206 (2.39%) | 0.772289897894 | 1.0 | Bcl2l1  Stat1  Erbin  Gsdmd  Xiap  Atg16l1  Mapk9 | web link | no local link |
| Aminoacyl-tRNA biosynthesis | KEGG PATHWAY | mmu00970 | 2 (0.55%) | 66 (0.77%) | 0.77379061566 | 1.0 | Farsa  Aars | web link | no local link |
| Non-small cell lung cancer | KEGG PATHWAY | mmu05223 | 2 (0.55%) | 66 (0.77%) | 0.77379061566 | 1.0 | Ddb2  Rassf5 | web link | no local link |
| Adrenergic signaling in cardiomyocytes | KEGG PATHWAY | mmu04261 | 5 (1.38%) | 152 (1.77%) | 0.774528771238 | 1.0 | Ppp2r2d  Ppp2r5c  Crem  Ppp2r3d  Atf2 | web link | no local link |
| Platelet activation | KEGG PATHWAY | mmu04611 | 4 (1.10%) | 125 (1.45%) | 0.779186602608 | 1.0 | Fermt3  Arhgef12  Gna13  Snap23 | web link | no local link |
| Leishmaniasis | KEGG PATHWAY | mmu05140 | 2 (0.55%) | 67 (0.78%) | 0.780937081717 | 1.0 | Irak1  Stat1 | web link | no local link |
| Central carbon metabolism in cancer | KEGG PATHWAY | mmu05230 | 2 (0.55%) | 67 (0.78%) | 0.780937081717 | 1.0 | Fgfr2  Gls2 | web link | no local link |
| Glycolysis / Gluconeogenesis | KEGG PATHWAY | mmu00010 | 2 (0.55%) | 67 (0.78%) | 0.780937081717 | 1.0 | Aldoa  Fbp1 | web link | no local link |
| Amphetamine addiction | KEGG PATHWAY | mmu05031 | 2 (0.55%) | 68 (0.79%) | 0.7878851933 | 1.0 | Maob  Atf2 | web link | no local link |
| Toll-like receptor signaling pathway | KEGG PATHWAY | mmu04620 | 3 (0.83%) | 99 (1.15%) | 0.794323670719 | 1.0 | Mapk9  Irak1  Stat1 | web link | no local link |
| Acute myeloid leukemia | KEGG PATHWAY | mmu05221 | 2 (0.55%) | 69 (0.80%) | 0.794638916105 | 1.0 | Tcf7l2  Rps6kb2 | web link | no local link |
| Osteoclast differentiation | KEGG PATHWAY | mmu04380 | 4 (1.10%) | 128 (1.49%) | 0.794655734506 | 1.0 | Acp5  Mapk9  Tec  Stat1 | web link | no local link |
| Th17 cell differentiation | KEGG PATHWAY | mmu04659 | 3 (0.83%) | 102 (1.18%) | 0.810687476678 | 1.0 | Mapk9  Rora  Stat1 | web link | no local link |
| Melanoma | KEGG PATHWAY | mmu05218 | 2 (0.55%) | 72 (0.84%) | 0.813773644428 | 1.0 | Ddb2  Fgf1 | web link | no local link |
| Serotonergic synapse | KEGG PATHWAY | mmu04726 | 4 (1.10%) | 132 (1.53%) | 0.813916041761 | 1.0 | Maob  Cyp2d10  Cyp2c40  Gngt1 | web link | no local link |
| Amoebiasis | KEGG PATHWAY | mmu05146 | 3 (0.83%) | 103 (1.20%) | 0.81589273455 | 1.0 | Actn1  Serpinb6a  Fn1 | web link | no local link |
| Homologous recombination | KEGG PATHWAY | mmu03440 | 1 (0.28%) | 41 (0.48%) | 0.829785254205 | 1.0 | Ssbp1 | web link | no local link |
| Bladder cancer | KEGG PATHWAY | mmu05219 | 1 (0.28%) | 41 (0.48%) | 0.829785254205 | 1.0 | Vegfa | web link | no local link |
| Parathyroid hormone synthesis, secretion and action | KEGG PATHWAY | mmu04928 | 3 (0.83%) | 107 (1.24%) | 0.835516398899 | 1.0 | Arrb2  Gna13  Atf2 | web link | no local link |
| Signaling pathways regulating pluripotency of stem cells | KEGG PATHWAY | mmu04550 | 4 (1.10%) | 138 (1.60%) | 0.840014549446 | 1.0 | Tbx3  Acvr2b  Fgfr2  Lifr | web link | no local link |
| Human papillomavirus infection | KEGG PATHWAY | mmu05165 | 12 (3.31%) | 361 (4.19%) | 0.840843198602 | 1.0 | Pard3  Csnk1a1  Stat1  Ppp2r2d  Itgav  Rps6kb2  Tcf7l2  Ppp2r3d  Vegfa  Pard6a  Fn1  Ppp2r5c | web link | no local link |
| Vasopressin-regulated water reabsorption | KEGG PATHWAY | mmu04962 | 1 (0.28%) | 43 (0.50%) | 0.843905102519 | 1.0 | Arhgdib | web link | no local link |
| Salmonella infection | KEGG PATHWAY | mmu05132 | 2 (0.55%) | 78 (0.91%) | 0.847332492999 | 1.0 | Mapk9  Arpc5 | web link | no local link |
| Vascular smooth muscle contraction | KEGG PATHWAY | mmu04270 | 4 (1.10%) | 140 (1.63%) | 0.848005249972 | 1.0 | Arhgef12  Myl6  Cyp4a31  Gna13 | web link | no local link |
| TNF signaling pathway | KEGG PATHWAY | mmu04668 | 3 (0.83%) | 113 (1.31%) | 0.861570149254 | 1.0 | Cflar  Mapk9  Atf2 | web link | no local link |
| Proteasome | KEGG PATHWAY | mmu03050 | 1 (0.28%) | 46 (0.53%) | 0.862924712287 | 1.0 | Psmc6 | web link | no local link |
| Measles | KEGG PATHWAY | mmu05162 | 4 (1.10%) | 144 (1.67%) | 0.862980182072 | 1.0 | Bcl2l1  Mapk9  Irak1  Stat1 | web link | no local link |
| Glutamatergic synapse | KEGG PATHWAY | mmu04724 | 3 (0.83%) | 114 (1.32%) | 0.865542772791 | 1.0 | Gngt1  Slc38a3  Gls2 | web link | no local link |
| Ether lipid metabolism | KEGG PATHWAY | mmu00565 | 1 (0.28%) | 47 (0.55%) | 0.868736243406 | 1.0 | Enpp2 | web link | no local link |
| Ribosome biogenesis in eukaryotes | KEGG PATHWAY | mmu03008 | 3 (0.83%) | 116 (1.35%) | 0.873188639362 | 1.0 | Sbds  Rcl1  Rexo5 | web link | no local link |
| Type II diabetes mellitus | KEGG PATHWAY | mmu04930 | 1 (0.28%) | 48 (0.56%) | 0.874302035211 | 1.0 | Mapk9 | web link | no local link |
| ABC transporters | KEGG PATHWAY | mmu02010 | 1 (0.28%) | 48 (0.56%) | 0.874302035211 | 1.0 | Abcd4 | web link | no local link |
| Rheumatoid arthritis | KEGG PATHWAY | mmu05323 | 2 (0.55%) | 84 (0.98%) | 0.875310827498 | 1.0 | Acp5  Vegfa | web link | no local link |
| Tuberculosis | KEGG PATHWAY | mmu05152 | 5 (1.38%) | 178 (2.07%) | 0.876440756114 | 1.0 | Mapk9  Arhgef12  Irak1  Stat1  Rfxap | web link | no local link |
| Notch signaling pathway | KEGG PATHWAY | mmu04330 | 1 (0.28%) | 49 (0.57%) | 0.879632451241 | 1.0 | Ncor2 | web link | no local link |
| Human immunodeficiency virus 1 infection | KEGG PATHWAY | mmu05170 | 7 (1.93%) | 239 (2.78%) | 0.883485789716 | 1.0 | Bcl2l1  Irak1  Btrc  Rps6kb2  Ddb1  Gngt1  Mapk9 | web link | no local link |
| Linoleic acid metabolism | KEGG PATHWAY | mmu00591 | 1 (0.28%) | 50 (0.58%) | 0.884737419135 | 1.0 | Cyp2c40 | web link | no local link |
| Th1 and Th2 cell differentiation | KEGG PATHWAY | mmu04658 | 2 (0.55%) | 87 (1.01%) | 0.887457947624 | 1.0 | Mapk9  Stat1 | web link | no local link |
| Transcriptional misregulation in cancer | KEGG PATHWAY | mmu05202 | 5 (1.38%) | 183 (2.13%) | 0.890758097197 | 1.0 | Ddb2  Bcl2l1  Klf3  Ncor1  Ss18 | web link | no local link |
| PD-L1 expression and PD-1 checkpoint pathway in cancer | KEGG PATHWAY | mmu05235 | 2 (0.55%) | 88 (1.02%) | 0.891257486137 | 1.0 | Stat1  Rps6kb2 | web link | no local link |
| Rap1 signaling pathway | KEGG PATHWAY | mmu04015 | 6 (1.65%) | 214 (2.49%) | 0.893899148087 | 1.0 | Pard3  Rassf5  Pard6a  Vegfa  Fgfr2  Fgf1 | web link | no local link |
| Arachidonic acid metabolism | KEGG PATHWAY | mmu00590 | 2 (0.55%) | 89 (1.03%) | 0.894937857213 | 1.0 | Cyp4a31  Cyp2c40 | web link | no local link |
| Antigen processing and presentation | KEGG PATHWAY | mmu04612 | 2 (0.55%) | 91 (1.06%) | 0.901954069692 | 1.0 | Ifi30  Rfxap | web link | no local link |
| Hypertrophic cardiomyopathy (HCM) | KEGG PATHWAY | mmu05410 | 2 (0.55%) | 91 (1.06%) | 0.901954069692 | 1.0 | Lmna  Itgav | web link | no local link |
| Morphine addiction | KEGG PATHWAY | mmu05032 | 2 (0.55%) | 92 (1.07%) | 0.905296241782 | 1.0 | Arrb2  Gngt1 | web link | no local link |
| Dilated cardiomyopathy (DCM) | KEGG PATHWAY | mmu05414 | 2 (0.55%) | 95 (1.10%) | 0.914695683251 | 1.0 | Lmna  Itgav | web link | no local link |
| Ovarian steroidogenesis | KEGG PATHWAY | mmu04913 | 1 (0.28%) | 57 (0.66%) | 0.914910133853 | 1.0 | Hsd17b2 | web link | no local link |
| Endocrine and other factor-regulated calcium reabsorption | KEGG PATHWAY | mmu04961 | 1 (0.28%) | 57 (0.66%) | 0.914910133853 | 1.0 | Esr1 | web link | no local link |
| Human cytomegalovirus infection | KEGG PATHWAY | mmu05163 | 7 (1.93%) | 256 (2.97%) | 0.920318669411 | 1.0 | Arhgef12  Itgav  Rps6kb2  Vegfa  Gna13  Gngt1  Atf2 | web link | no local link |
| Prostate cancer | KEGG PATHWAY | mmu05215 | 2 (0.55%) | 97 (1.13%) | 0.920468699826 | 1.0 | Fgfr2  Tcf7l2 | web link | no local link |
| FoxO signaling pathway | KEGG PATHWAY | mmu04068 | 3 (0.83%) | 132 (1.53%) | 0.921700454674 | 1.0 | Mapk9  Stk11  Sgk1 | web link | no local link |
| Circadian entrainment | KEGG PATHWAY | mmu04713 | 2 (0.55%) | 99 (1.15%) | 0.925873244769 | 1.0 | Per1  Gngt1 | web link | no local link |
| Estrogen signaling pathway | KEGG PATHWAY | mmu04915 | 3 (0.83%) | 134 (1.56%) | 0.926397479626 | 1.0 | Fkbp4  Atf2  Esr1 | web link | no local link |
| Epstein-Barr virus infection | KEGG PATHWAY | mmu05169 | 6 (1.65%) | 230 (2.67%) | 0.927697825188 | 1.0 | Irak1  Stat1  Ddb2  Mapk9  Ncor2  Psmc6 | web link | no local link |
| Long-term depression | KEGG PATHWAY | mmu04730 | 1 (0.28%) | 61 (0.71%) | 0.92846673679 | 1.0 | Gna13 | web link | no local link |
| T cell receptor signaling pathway | KEGG PATHWAY | mmu04660 | 2 (0.55%) | 103 (1.20%) | 0.935661062722 | 1.0 | Mapk9  Tec | web link | no local link |
| Apelin signaling pathway | KEGG PATHWAY | mmu04371 | 3 (0.83%) | 139 (1.61%) | 0.937033304276 | 1.0 | Gna13  Gngt1  Rps6kb2 | web link | no local link |
| Long-term potentiation | KEGG PATHWAY | mmu04720 | 1 (0.28%) | 67 (0.78%) | 0.944870288965 | 1.0 | Rps6ka3 | web link | no local link |
| RIG-I-like receptor signaling pathway | KEGG PATHWAY | mmu04622 | 1 (0.28%) | 68 (0.79%) | 0.947213350094 | 1.0 | Mapk9 | web link | no local link |
| Fc epsilon RI signaling pathway | KEGG PATHWAY | mmu04664 | 1 (0.28%) | 68 (0.79%) | 0.947213350094 | 1.0 | Mapk9 | web link | no local link |
| Renal cell carcinoma | KEGG PATHWAY | mmu05211 | 1 (0.28%) | 68 (0.79%) | 0.947213350094 | 1.0 | Vegfa | web link | no local link |
| Cortisol synthesis and secretion | KEGG PATHWAY | mmu04927 | 1 (0.28%) | 69 (0.80%) | 0.949457091771 | 1.0 | Atf2 | web link | no local link |
| Human T-cell leukemia virus 1 infection | KEGG PATHWAY | mmu05166 | 6 (1.65%) | 246 (2.86%) | 0.95165771638 | 1.0 | Bcl2l1  Anapc10  Xiap  Mapk9  Anapc5  Atf2 | web link | no local link |
| Kaposi sarcoma-associated herpesvirus infection | KEGG PATHWAY | mmu05167 | 5 (1.38%) | 217 (2.52%) | 0.955216150161 | 1.0 | Stat1  Mapk9  Vegfa  Mapkapk2  Gngt1 | web link | no local link |
| Thyroid hormone synthesis | KEGG PATHWAY | mmu04918 | 1 (0.28%) | 73 (0.85%) | 0.957520230448 | 1.0 | Atf2 | web link | no local link |
| Gastric acid secretion | KEGG PATHWAY | mmu04971 | 1 (0.28%) | 74 (0.86%) | 0.95932692715 | 1.0 | Slc4a2 | web link | no local link |
| Glioma | KEGG PATHWAY | mmu05214 | 1 (0.28%) | 75 (0.87%) | 0.961056986377 | 1.0 | Ddb2 | web link | no local link |
| Salivary secretion | KEGG PATHWAY | mmu04970 | 1 (0.28%) | 78 (0.91%) | 0.965819083461 | 1.0 | Slc4a2 | web link | no local link |
| Chemokine signaling pathway | KEGG PATHWAY | mmu04062 | 4 (1.10%) | 198 (2.30%) | 0.9705119548 | 1.0 | Pard3  Arrb2  Stat1  Gngt1 | web link | no local link |
| Alcoholism | KEGG PATHWAY | mmu05034 | 4 (1.10%) | 199 (2.31%) | 0.97139652527 | 1.0 | Hdac10  Maob  Gngt1  Atf2 | web link | no local link |
| Insulin secretion | KEGG PATHWAY | mmu04911 | 1 (0.28%) | 86 (1.00%) | 0.975866045034 | 1.0 | Atf2 | web link | no local link |
| Viral myocarditis | KEGG PATHWAY | mmu05416 | 1 (0.28%) | 89 (1.03%) | 0.97882088032 | 1.0 | Abl1 | web link | no local link |
| Herpes simplex virus 1 infection | KEGG PATHWAY | mmu05168 | 11 (3.03%) | 435 (5.05%) | 0.979164620976 | 1.0 | Bcl2l1  Zfp825  Stat1  Zfp780b  Srsf3  Zfp938  Zfp235  Zfp874a  Zfp874b  Zfp58  Irak1 | web link | no local link |
| cGMP-PKG signaling pathway | KEGG PATHWAY | mmu04022 | 3 (0.83%) | 173 (2.01%) | 0.979207012063 | 1.0 | Gtf2i  Gna13  Atf2 | web link | no local link |
| GnRH signaling pathway | KEGG PATHWAY | mmu04912 | 1 (0.28%) | 90 (1.05%) | 0.97972333606 | 1.0 | Mapk9 | web link | no local link |
| MicroRNAs in cancer | KEGG PATHWAY | mmu05206 | 6 (1.65%) | 282 (3.28%) | 0.981610547046 | 1.0 | Ube2i  Hnrnpk  Vegfa  Abl1  Mdm4  Gls2 | web link | no local link |
| Hematopoietic cell lineage | KEGG PATHWAY | mmu04640 | 1 (0.28%) | 95 (1.10%) | 0.983691821798 | 1.0 | Thpo | web link | no local link |
| Cellular senescence | KEGG PATHWAY | mmu04218 | 3 (0.83%) | 186 (2.16%) | 0.986633284789 | 1.0 | Btrc  Rassf5  Mapkapk2 | web link | no local link |
| Melanogenesis | KEGG PATHWAY | mmu04916 | 1 (0.28%) | 100 (1.16%) | 0.986885323125 | 1.0 | Tcf7l2 | web link | no local link |
| Aldosterone synthesis and secretion | KEGG PATHWAY | mmu04925 | 1 (0.28%) | 102 (1.18%) | 0.987980607035 | 1.0 | Atf2 | web link | no local link |
| Phospholipase D signaling pathway | KEGG PATHWAY | mmu04072 | 2 (0.55%) | 149 (1.73%) | 0.988182024381 | 1.0 | Pip5k1c  Gna13 | web link | no local link |
| Pancreatic secretion | KEGG PATHWAY | mmu04972 | 1 (0.28%) | 105 (1.22%) | 0.989454847273 | 1.0 | Slc4a2 | web link | no local link |
| Cushing syndrome | KEGG PATHWAY | mmu04934 | 2 (0.55%) | 159 (1.85%) | 0.991926450697 | 1.0 | Tcf7l2  Atf2 | web link | no local link |
| Cholinergic synapse | KEGG PATHWAY | mmu04725 | 1 (0.28%) | 113 (1.31%) | 0.992562844416 | 1.0 | Gngt1 | web link | no local link |
| Influenza A | KEGG PATHWAY | mmu05164 | 2 (0.55%) | 164 (1.91%) | 0.993335977038 | 1.0 | Stat1  Nup98 | web link | no local link |
| cAMP signaling pathway | KEGG PATHWAY | mmu04024 | 3 (0.83%) | 213 (2.47%) | 0.994797511787 | 1.0 | Acox1  Mapk9  Fxyd1 | web link | no local link |
| Cell adhesion molecules (CAMs) | KEGG PATHWAY | mmu04514 | 2 (0.55%) | 171 (1.99%) | 0.994913007515 | 1.0 | Nectin3  Itgav | web link | no local link |
| Cytokine-cytokine receptor interaction | KEGG PATHWAY | mmu04060 | 5 (1.38%) | 293 (3.40%) | 0.995321974816 | 1.0 | Prlr  Acvr2b  Lifr  Ghr  Thpo | web link | no local link |
| Phagosome | KEGG PATHWAY | mmu04145 | 2 (0.55%) | 181 (2.10%) | 0.996550511443 | 1.0 | Sec61a2  Itgav | web link | no local link |
| Calcium signaling pathway | KEGG PATHWAY | mmu04020 | 2 (0.55%) | 194 (2.25%) | 0.997927284057 | 1.0 | Asph  Phkb | web link | no local link |
| Systemic lupus erythematosus | KEGG PATHWAY | mmu05322 | 1 (0.28%) | 143 (1.66%) | 0.997998150561 | 1.0 | Actn1 | web link | no local link |
| Oxytocin signaling pathway | KEGG PATHWAY | mmu04921 | 1 (0.28%) | 154 (1.79%) | 0.998764271911 | 1.0 | Myl6 | web link | no local link |
| Neuroactive ligand-receptor interaction | KEGG PATHWAY | mmu04080 | 4 (1.10%) | 348 (4.04%) | 0.999817487375 | 1.0 | Prlr  P2ry2  Pard3  Ghr | web link | no local link |
| Olfactory transduction | KEGG PATHWAY | mmu04740 | 1 (0.28%) | 1134 (13.17%) | 1.0 | 1.0 | Arrb2 | web link | no local link |
