# Supplementary material for: Regulation of Alternative Splicing of Lipid Metabolism Genes in Sepsis-Induced Liver Damage by RNA-Binding Proteins
Source: Inflammation. 2024 May 9;47(6):1952–68. doi: 10.1007/s10753-024-02017-2 (PMC11607010; doi:10.1007/s10753-024-02017-2)
Supplement: Supplementary file 1 — Supplementary file1 (ZIP 38655 KB) [file 10753_2024_2017_MOESM1_ESM.zip › Sup6_AS_KEGG.zip!/Sup6_AS_KEGG/ras/CLP_vs_Sham/Ras_KEGG_analysis/Ras_KEGG_top.pdf]

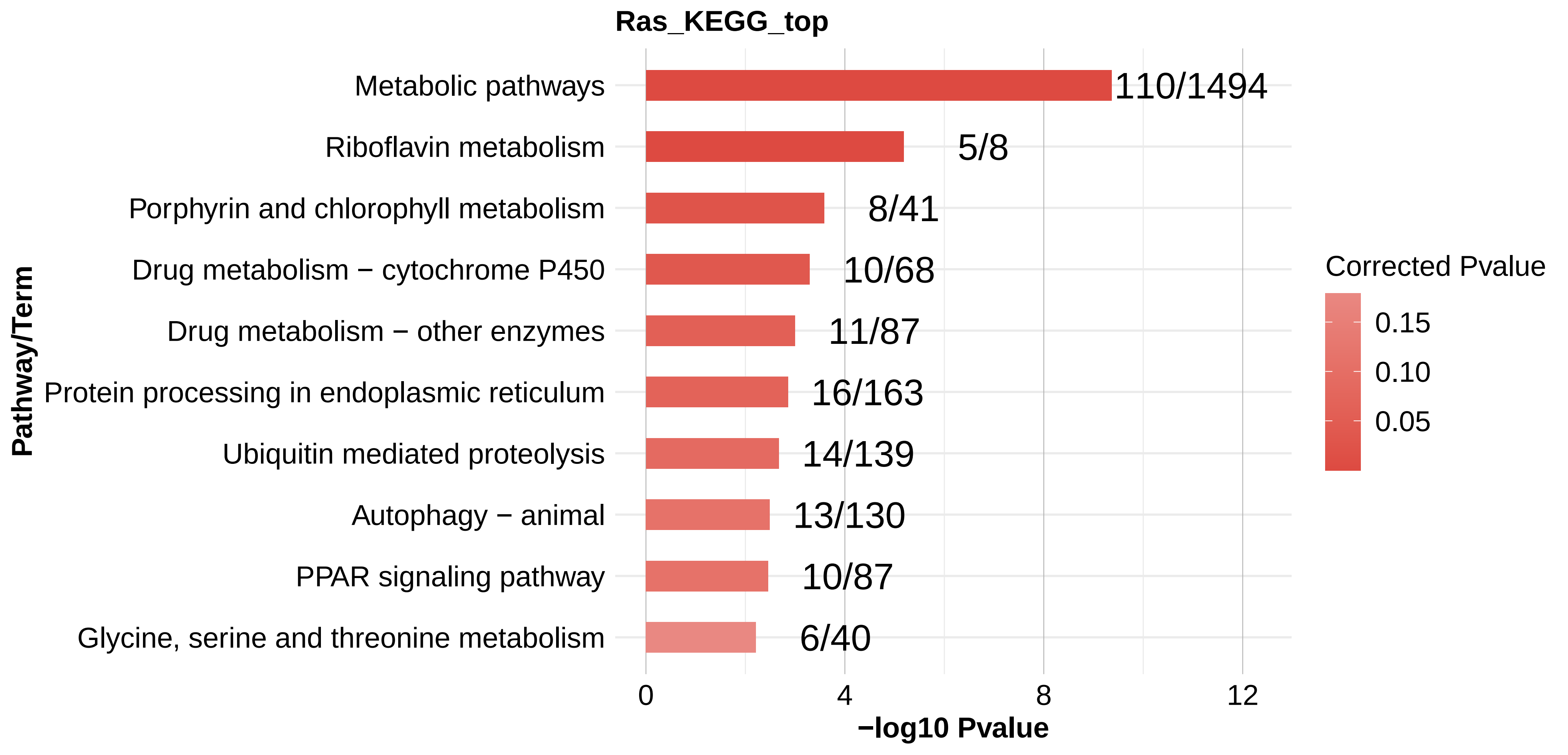

Ras_KEGG_top
Pathway/Term
Metabolic pathways
Riboflavin metabolism
Porphyrin and chlorophyll metabolism
Drug metabolism − cytochrome P450
Drug metabolism − other enzymes
Protein processing in endoplasmic reticulum
Ubiquitin mediated proteolysis
Autophagy − animal
PPAR signaling pathway
Glycine, serine and threonine metabolism
110/1494
5/8
8/41
10/68
11/87
16/163
14/139
13/130
10/87
6/40
0
4
8
12
−log10 Pvalue
Corrected Pvalue
0.15
0.10
0.05
