# Supplementary material for: Regulation of Alternative Splicing of Lipid Metabolism Genes in Sepsis-Induced Liver Damage by RNA-Binding Proteins
Source: Inflammation. 2024 May 9;47(6):1952–68. doi: 10.1007/s10753-024-02017-2 (PMC11607010; doi:10.1007/s10753-024-02017-2)
Supplement: Supplementary file 1 — Supplementary file1 (ZIP 38655 KB) [file 10753_2024_2017_MOESM1_ESM.zip › Sup6_AS_KEGG.zip!/Sup6_AS_KEGG/ras/di_RAS/GO_KEGG/CLP_vs_Sham_and_CLPDCA_vs_Sham_di_RAS/CLPDCA_vs_Sham_diDown_nir_KEGG_analysis/CLPDCA_vs_Sham_diDown_nir_KEGG_pathway.html]

KEGG pathway enrichment analyze result


# KEGG pathway enrichment analyze result


| Term | Database | ID | Input number (104) | Background number (8608) | P-Value | Corrected P-Value | Input | Hyperlink | img |
| --- | --- | --- | --- | --- | --- | --- | --- | --- | --- |
| AMPK signaling pathway | KEGG PATHWAY | mmu04152 | 6 (5.77%) | 126 (1.46%) | 0.00411670736437 | 0.410965160412 | Adra1a  Ppp2r3a  Adipor2  Stk11  Ppp2r3d  Tsc1 | web link | no local link |
| mRNA surveillance pathway | KEGG PATHWAY | mmu03015 | 5 (4.81%) | 96 (1.12%) | 0.00599212801547 | 0.410965160412 | Nudt21  Ppp2r3a  Ppp2r3d  Sap18  Ppp1ca | web link | no local link |
| Glycerophospholipid metabolism | KEGG PATHWAY | mmu00564 | 5 (4.81%) | 97 (1.13%) | 0.00625835269663 | 0.410965160412 | Crls1  Cept1  Pnpla6  Taz  Cdipt | web link | no local link |
| Sphingolipid signaling pathway | KEGG PATHWAY | mmu04071 | 5 (4.81%) | 124 (1.44%) | 0.0169550614599 | 0.565782061588 | Acer2  Ppp2r3a  Sgms2  Ppp2r3d  Kras | web link | no local link |
| Metabolic pathways | KEGG PATHWAY | mmu01100 | 27 (25.96%) | 1494 (17.36%) | 0.0172064918896 | 0.565782061588 | Acer2  Cmbl  Tdo2  Bpnt1  Auh  Cept1  Entpd8  Hsd3b2  Prodh2  Fpgt  Sgms2  Cyp4f13  Pigx  Cyp2r1  Ido2  Mgam  Alas2  P4ha1  Crls1  Mvk  Cdipt  Ahcyl1  Enpp4  Gsta3  Atp5j  Mgst1  Rdh11 | web link | no local link |
| Hepatocellular carcinoma | KEGG PATHWAY | mmu05225 | 6 (5.77%) | 171 (1.99%) | 0.0172319409621 | 0.565782061588 | Smarcc2  Csnk1a1  Gsta3  Mgst1  Smarca4  Kras | web link | no local link |
| Autophagy - animal | KEGG PATHWAY | mmu04140 | 5 (4.81%) | 130 (1.51%) | 0.0203765109315 | 0.573042787234 | Cflar  Tsc1  Stk11  Rubcn  Kras | web link | no local link |
| Longevity regulating pathway | KEGG PATHWAY | mmu04211 | 4 (3.85%) | 90 (1.05%) | 0.0232707730856 | 0.573042787234 | Stk11  Tsc1  Adipor2  Kras | web link | no local link |
| Human papillomavirus infection | KEGG PATHWAY | mmu05165 | 9 (8.65%) | 361 (4.19%) | 0.0301394453627 | 0.659718970718 | Csnk1a1  Fas  Irf9  Ppp2r3a  Ppp2r3d  Kras  Magi1  Cdc42  Tsc1 | web link | no local link |
| Proteoglycans in cancer | KEGG PATHWAY | mmu05205 | 6 (5.77%) | 206 (2.39%) | 0.0382962424217 | 0.702269013608 | Ppp1ca  Fas  Esr1  Flnb  Kras  Cdc42 | web link | no local link |
| mTOR signaling pathway | KEGG PATHWAY | mmu04150 | 5 (4.81%) | 155 (1.80%) | 0.0393748037813 | 0.702269013608 | Fnip1  Tsc1  Stk11  Wdr59  Kras | web link | no local link |
| Renal cell carcinoma | KEGG PATHWAY | mmu05211 | 3 (2.88%) | 68 (0.79%) | 0.0488132274212 | 0.702269013608 | Cdc42  Eloc  Kras | web link | no local link |
| Thermogenesis | KEGG PATHWAY | mmu04714 | 6 (5.77%) | 232 (2.70%) | 0.0615889636088 | 0.702269013608 | Smarcc2  Ndufaf4  Atp5j  Kras  Smarca4  Tsc1 | web link | no local link |
| Platinum drug resistance | KEGG PATHWAY | mmu01524 | 3 (2.88%) | 77 (0.89%) | 0.0659976584571 | 0.702269013608 | Fas  Mgst1  Gsta3 | web link | no local link |
| Axon guidance | KEGG PATHWAY | mmu04360 | 5 (4.81%) | 180 (2.09%) | 0.0665719998558 | 0.702269013608 | Cdc42  Sema4g  Ngef  Sema4a  Kras | web link | no local link |
| PI3K-Akt signaling pathway | KEGG PATHWAY | mmu04151 | 8 (7.69%) | 358 (4.16%) | 0.0674847287264 | 0.702269013608 | Ppp2r3a  Ghr  Stk11  Ppp2r3d  Magi1  Kras  Fgfr3  Tsc1 | web link | no local link |
| Phosphonate and phosphinate metabolism | KEGG PATHWAY | mmu00440 | 1 (0.96%) | 6 (0.07%) | 0.0703559371233 | 0.702269013608 | Cept1 | web link | no local link |
| EGFR tyrosine kinase inhibitor resistance | KEGG PATHWAY | mmu01521 | 3 (2.88%) | 80 (0.93%) | 0.0722660141522 | 0.702269013608 | Fgfr3  Nf1  Kras | web link | no local link |
| African trypanosomiasis | KEGG PATHWAY | mmu05143 | 2 (1.92%) | 39 (0.45%) | 0.0802800876597 | 0.702269013608 | Ido2  Fas | web link | no local link |
| Signaling pathways regulating pluripotency of stem cells | KEGG PATHWAY | mmu04550 | 4 (3.85%) | 138 (1.60%) | 0.0855900143104 | 0.702269013608 | Lifr  Fgfr3  Setdb1  Kras | web link | no local link |
| Ubiquitin mediated proteolysis | KEGG PATHWAY | mmu04120 | 4 (3.85%) | 139 (1.61%) | 0.0873464498424 | 0.702269013608 | Birc6  Eloc  Ube2j2  Ube2d3 | web link | no local link |
| Porphyrin and chlorophyll metabolism | KEGG PATHWAY | mmu00860 | 2 (1.92%) | 41 (0.48%) | 0.0874898952023 | 0.702269013608 | Hccs  Alas2 | web link | no local link |
| Ferroptosis | KEGG PATHWAY | mmu04216 | 2 (1.92%) | 41 (0.48%) | 0.0874898952023 | 0.702269013608 | Pcbp2  Slc11a2 | web link | no local link |
| Bladder cancer | KEGG PATHWAY | mmu05219 | 2 (1.92%) | 41 (0.48%) | 0.0874898952023 | 0.702269013608 | Fgfr3  Kras | web link | no local link |
| Insulin signaling pathway | KEGG PATHWAY | mmu04910 | 4 (3.85%) | 140 (1.63%) | 0.089120433199 | 0.702269013608 | Ppp1ca  Tsc1  Exoc7  Kras | web link | no local link |
| Ether lipid metabolism | KEGG PATHWAY | mmu00565 | 2 (1.92%) | 47 (0.55%) | 0.110182986186 | 0.764714145498 | Enpp2  Cept1 | web link | no local link |
| Adrenergic signaling in cardiomyocytes | KEGG PATHWAY | mmu04261 | 4 (3.85%) | 152 (1.77%) | 0.111734665827 | 0.764714145498 | Adra1a  Ppp2r3a  Ppp2r3d  Ppp1ca | web link | no local link |
| Sphingolipid metabolism | KEGG PATHWAY | mmu00600 | 2 (1.92%) | 48 (0.56%) | 0.114103956099 | 0.764714145498 | Acer2  Sgms2 | web link | no local link |
| Tryptophan metabolism | KEGG PATHWAY | mmu00380 | 2 (1.92%) | 48 (0.56%) | 0.114103956099 | 0.764714145498 | Ido2  Tdo2 | web link | no local link |
| Regulation of actin cytoskeleton | KEGG PATHWAY | mmu04810 | 5 (4.81%) | 217 (2.52%) | 0.121913677495 | 0.764714145498 | Baiap2  Cdc42  Ppp1ca  Fgfr3  Kras | web link | no local link |
| Arginine and proline metabolism | KEGG PATHWAY | mmu00330 | 2 (1.92%) | 50 (0.58%) | 0.122051609479 | 0.764714145498 | P4ha1  Prodh2 | web link | no local link |
| Sulfur metabolism | KEGG PATHWAY | mmu00920 | 1 (0.96%) | 11 (0.13%) | 0.125223793772 | 0.764714145498 | Bpnt1 | web link | no local link |
| Hepatitis C | KEGG PATHWAY | mmu05160 | 4 (3.85%) | 160 (1.86%) | 0.128099323865 | 0.764714145498 | Fas  Irf9  Cflar  Kras | web link | no local link |
| Protein processing in endoplasmic reticulum | KEGG PATHWAY | mmu04141 | 4 (3.85%) | 163 (1.89%) | 0.134484092979 | 0.779216656378 | Derl3  Fbxo6  Ube2j2  Ube2d3 | web link | no local link |
| Tight junction | KEGG PATHWAY | mmu04530 | 4 (3.85%) | 167 (1.94%) | 0.143196970845 | 0.785798182485 | Stk11  Nf2  Magi1  Cdc42 | web link | no local link |
| MAPK signaling pathway | KEGG PATHWAY | mmu04010 | 6 (5.77%) | 294 (3.42%) | 0.144687472956 | 0.785798182485 | Fas  Nf1  Flnb  Kras  Fgfr3  Cdc42 | web link | no local link |
| VEGF signaling pathway | KEGG PATHWAY | mmu04370 | 2 (1.92%) | 58 (0.67%) | 0.15505358257 | 0.785798182485 | Cdc42  Kras | web link | no local link |
| Lysine degradation | KEGG PATHWAY | mmu00310 | 2 (1.92%) | 59 (0.69%) | 0.159294140922 | 0.785798182485 | Nsd1  Setdb1 | web link | no local link |
| Glutathione metabolism | KEGG PATHWAY | mmu00480 | 2 (1.92%) | 64 (0.74%) | 0.180799444869 | 0.785798182485 | Mgst1  Gsta3 | web link | no local link |
| Metabolism of xenobiotics by cytochrome P450 | KEGG PATHWAY | mmu00980 | 2 (1.92%) | 66 (0.77%) | 0.189522688005 | 0.785798182485 | Mgst1  Gsta3 | web link | no local link |
| Long-term potentiation | KEGG PATHWAY | mmu04720 | 2 (1.92%) | 67 (0.78%) | 0.193906169891 | 0.785798182485 | Ppp1ca  Kras | web link | no local link |
| Central carbon metabolism in cancer | KEGG PATHWAY | mmu05230 | 2 (1.92%) | 67 (0.78%) | 0.193906169891 | 0.785798182485 | Fgfr3  Kras | web link | no local link |
| Pathways in cancer | KEGG PATHWAY | mmu05200 | 9 (8.65%) | 535 (6.22%) | 0.197053546701 | 0.785798182485 | Esr1  Fas  Gsta3  Mgst1  Eloc  Ctbp1  Kras  Fgfr3  Cdc42 | web link | no local link |
| Drug metabolism - cytochrome P450 | KEGG PATHWAY | mmu00982 | 2 (1.92%) | 68 (0.79%) | 0.198302925723 | 0.785798182485 | Mgst1  Gsta3 | web link | no local link |
| Cortisol synthesis and secretion | KEGG PATHWAY | mmu04927 | 2 (1.92%) | 69 (0.80%) | 0.202712007041 | 0.785798182485 | Hsd3b2  Mrap | web link | no local link |
| Steroid biosynthesis | KEGG PATHWAY | mmu00100 | 1 (0.96%) | 19 (0.22%) | 0.206415439919 | 0.785798182485 | Cyp2r1 | web link | no local link |
| Adipocytokine signaling pathway | KEGG PATHWAY | mmu04920 | 2 (1.92%) | 71 (0.82%) | 0.211563454747 | 0.785798182485 | Stk11  Adipor2 | web link | no local link |
| Prolactin signaling pathway | KEGG PATHWAY | mmu04917 | 2 (1.92%) | 72 (0.84%) | 0.216004025624 | 0.785798182485 | Esr1  Kras | web link | no local link |
| Adherens junction | KEGG PATHWAY | mmu04520 | 2 (1.92%) | 72 (0.84%) | 0.216004025624 | 0.785798182485 | Baiap2  Cdc42 | web link | no local link |
| Spliceosome | KEGG PATHWAY | mmu03040 | 3 (2.88%) | 133 (1.55%) | 0.216966573983 | 0.785798182485 | Hnrnpa1  Hnrnpu  Srsf2 | web link | no local link |
| Estrogen signaling pathway | KEGG PATHWAY | mmu04915 | 3 (2.88%) | 134 (1.56%) | 0.220132025745 | 0.785798182485 | Esr1  Krt10  Kras | web link | no local link |
| Dopaminergic synapse | KEGG PATHWAY | mmu04728 | 3 (2.88%) | 135 (1.57%) | 0.223307138835 | 0.785798182485 | Ppp2r3a  Ppp2r3d  Ppp1ca | web link | no local link |
| Apoptosis | KEGG PATHWAY | mmu04210 | 3 (2.88%) | 136 (1.58%) | 0.226491580806 | 0.785798182485 | Fas  Cflar  Kras | web link | no local link |
| Endocytosis | KEGG PATHWAY | mmu04144 | 5 (4.81%) | 270 (3.14%) | 0.227509822694 | 0.785798182485 | Tsg101  Cdc42  Fgfr3  Washc1  Asap3 | web link | no local link |
| Pancreatic cancer | KEGG PATHWAY | mmu05212 | 2 (1.92%) | 75 (0.87%) | 0.229374763398 | 0.785798182485 | Cdc42  Kras | web link | no local link |
| Chronic myeloid leukemia | KEGG PATHWAY | mmu05220 | 2 (1.92%) | 76 (0.88%) | 0.233845249913 | 0.785798182485 | Ctbp1  Kras | web link | no local link |
| Other types of O-glycan biosynthesis | KEGG PATHWAY | mmu00514 | 1 (0.96%) | 22 (0.26%) | 0.234898450117 | 0.785798182485 | Poglut1 | web link | no local link |
| Proximal tubule bicarbonate reclamation | KEGG PATHWAY | mmu04964 | 1 (0.96%) | 22 (0.26%) | 0.234898450117 | 0.785798182485 | Slc38a3 | web link | no local link |
| Salmonella infection | KEGG PATHWAY | mmu05132 | 2 (1.92%) | 78 (0.91%) | 0.242801795137 | 0.785798182485 | Flnb  Cdc42 | web link | no local link |
| Terpenoid backbone biosynthesis | KEGG PATHWAY | mmu00900 | 1 (0.96%) | 23 (0.27%) | 0.244165927544 | 0.785798182485 | Mvk | web link | no local link |
| Rap1 signaling pathway | KEGG PATHWAY | mmu04015 | 4 (3.85%) | 214 (2.49%) | 0.259104569986 | 0.785798182485 | Cdc42  Fgfr3  Magi1  Kras | web link | no local link |
| Breast cancer | KEGG PATHWAY | mmu05224 | 3 (2.88%) | 147 (1.71%) | 0.262043365189 | 0.785798182485 | Esr1  Csnk1a1  Kras | web link | no local link |
| Glycosylphosphatidylinositol (GPI)-anchor biosynthesis | KEGG PATHWAY | mmu00563 | 1 (0.96%) | 25 (0.29%) | 0.262368639181 | 0.785798182485 | Pigx | web link | no local link |
| Peroxisome | KEGG PATHWAY | mmu04146 | 2 (1.92%) | 84 (0.98%) | 0.269742345781 | 0.785798182485 | Mvk  Pex16 | web link | no local link |
| Hippo signaling pathway - multiple species | KEGG PATHWAY | mmu04392 | 1 (0.96%) | 26 (0.30%) | 0.271306500247 | 0.785798182485 | Nf2 | web link | no local link |
| Herpes simplex virus 1 infection | KEGG PATHWAY | mmu05168 | 7 (6.73%) | 435 (5.05%) | 0.272280899197 | 0.785798182485 | Srsf2  Fas  Irf9  Ppp1ca  Zfp235  Rsl1  Tsc1 | web link | no local link |
| Non-alcoholic fatty liver disease (NAFLD) | KEGG PATHWAY | mmu04932 | 3 (2.88%) | 151 (1.75%) | 0.275158726736 | 0.785798182485 | Fas  Adipor2  Cdc42 | web link | no local link |
| Drug metabolism - other enzymes | KEGG PATHWAY | mmu00983 | 2 (1.92%) | 87 (1.01%) | 0.283218097977 | 0.785798182485 | Mgst1  Gsta3 | web link | no local link |
| GnRH signaling pathway | KEGG PATHWAY | mmu04912 | 2 (1.92%) | 90 (1.05%) | 0.296674470269 | 0.785798182485 | Cdc42  Kras | web link | no local link |
| Epstein-Barr virus infection | KEGG PATHWAY | mmu05169 | 4 (3.85%) | 230 (2.67%) | 0.302324733311 | 0.785798182485 | Fas  Irf9  Psmc6  Entpd8 | web link | no local link |
| Viral carcinogenesis | KEGG PATHWAY | mmu05203 | 4 (3.85%) | 231 (2.68%) | 0.305058869576 | 0.785798182485 | Dnaja3  Irf9  Cdc42  Kras | web link | no local link |
| Fc gamma R-mediated phagocytosis | KEGG PATHWAY | mmu04666 | 2 (1.92%) | 92 (1.07%) | 0.30562678698 | 0.785798182485 | Cdc42  Asap3 | web link | no local link |
| Endocrine resistance | KEGG PATHWAY | mmu01522 | 2 (1.92%) | 93 (1.08%) | 0.310095699529 | 0.785798182485 | Esr1  Kras | web link | no local link |
| Ras signaling pathway | KEGG PATHWAY | mmu04014 | 4 (3.85%) | 233 (2.71%) | 0.310535708097 | 0.785798182485 | Cdc42  Fgfr3  Nf1  Kras | web link | no local link |
| Wnt signaling pathway | KEGG PATHWAY | mmu04310 | 3 (2.88%) | 162 (1.88%) | 0.311529432866 | 0.785798182485 | Porcn  Csnk1a1  Ctbp1 | web link | no local link |
| Chemical carcinogenesis | KEGG PATHWAY | mmu05204 | 2 (1.92%) | 94 (1.09%) | 0.314559072858 | 0.785798182485 | Mgst1  Gsta3 | web link | no local link |
| Jak-STAT signaling pathway | KEGG PATHWAY | mmu04630 | 3 (2.88%) | 165 (1.92%) | 0.321491104802 | 0.785798182485 | Irf9  Lifr  Ghr | web link | no local link |
| Galactose metabolism | KEGG PATHWAY | mmu00052 | 1 (0.96%) | 32 (0.37%) | 0.322725082566 | 0.785798182485 | Mgam | web link | no local link |
| Apoptosis - multiple species | KEGG PATHWAY | mmu04215 | 1 (0.96%) | 32 (0.37%) | 0.322725082566 | 0.785798182485 | Birc6 | web link | no local link |
| Staphylococcus aureus infection | KEGG PATHWAY | mmu05150 | 2 (1.92%) | 96 (1.12%) | 0.323467155269 | 0.785798182485 | Cfh  Krt10 | web link | no local link |
| RNA transport | KEGG PATHWAY | mmu03013 | 3 (2.88%) | 167 (1.94%) | 0.328135313006 | 0.785798182485 | Eif4g3  Nup37  Sap18 | web link | no local link |
| Starch and sucrose metabolism | KEGG PATHWAY | mmu00500 | 1 (0.96%) | 33 (0.38%) | 0.330938304512 | 0.785798182485 | Mgam | web link | no local link |
| Choline metabolism in cancer | KEGG PATHWAY | mmu05231 | 2 (1.92%) | 99 (1.15%) | 0.336775280056 | 0.785798182485 | Tsc1  Kras | web link | no local link |
| Ribosome | KEGG PATHWAY | mmu03010 | 3 (2.88%) | 171 (1.99%) | 0.341422223426 | 0.785798182485 | Mrps17  Uba52  Mrps2 | web link | no local link |
| AGE-RAGE signaling pathway in diabetic complications | KEGG PATHWAY | mmu04933 | 2 (1.92%) | 101 (1.17%) | 0.345605946026 | 0.785798182485 | Cdc42  Kras | web link | no local link |
| Fructose and mannose metabolism | KEGG PATHWAY | mmu00051 | 1 (0.96%) | 35 (0.41%) | 0.347069958708 | 0.785798182485 | Fpgt | web link | no local link |
| Chagas disease (American trypanosomiasis) | KEGG PATHWAY | mmu05142 | 2 (1.92%) | 103 (1.20%) | 0.354399395019 | 0.785798182485 | Fas  Cflar | web link | no local link |
| T cell receptor signaling pathway | KEGG PATHWAY | mmu04660 | 2 (1.92%) | 103 (1.20%) | 0.354399395019 | 0.785798182485 | Cdc42  Kras | web link | no local link |
| Necroptosis | KEGG PATHWAY | mmu04217 | 3 (2.88%) | 177 (2.06%) | 0.361320358987 | 0.785798182485 | Fas  Irf9  Cflar | web link | no local link |
| Thyroid cancer | KEGG PATHWAY | mmu05216 | 1 (0.96%) | 37 (0.43%) | 0.36281631524 | 0.785798182485 | Kras | web link | no local link |
| Aldosterone-regulated sodium reabsorption | KEGG PATHWAY | mmu04960 | 1 (0.96%) | 38 (0.44%) | 0.370547863859 | 0.785798182485 | Kras | web link | no local link |
| Glycine, serine and threonine metabolism | KEGG PATHWAY | mmu00260 | 1 (0.96%) | 40 (0.46%) | 0.385733298405 | 0.785798182485 | Alas2 | web link | no local link |
| Fat digestion and absorption | KEGG PATHWAY | mmu04975 | 1 (0.96%) | 40 (0.46%) | 0.385733298405 | 0.785798182485 | Abcg8 | web link | no local link |
| Cellular senescence | KEGG PATHWAY | mmu04218 | 3 (2.88%) | 186 (2.16%) | 0.391000125243 | 0.785798182485 | Ppp1ca  Tsc1  Kras | web link | no local link |
| C-type lectin receptor signaling pathway | KEGG PATHWAY | mmu04625 | 2 (1.92%) | 112 (1.30%) | 0.393425124544 | 0.785798182485 | Irf9  Kras | web link | no local link |
| TNF signaling pathway | KEGG PATHWAY | mmu04668 | 2 (1.92%) | 113 (1.31%) | 0.397699169763 | 0.785798182485 | Fas  Cflar | web link | no local link |
| Vasopressin-regulated water reabsorption | KEGG PATHWAY | mmu04962 | 1 (0.96%) | 43 (0.50%) | 0.407833690265 | 0.785798182485 | Dctn4 | web link | no local link |
| Carbohydrate digestion and absorption | KEGG PATHWAY | mmu04973 | 1 (0.96%) | 43 (0.50%) | 0.407833690265 | 0.785798182485 | Mgam | web link | no local link |
| Hedgehog signaling pathway | KEGG PATHWAY | mmu04340 | 1 (0.96%) | 44 (0.51%) | 0.415024034248 | 0.785798182485 | Csnk1a1 | web link | no local link |
| Natural killer cell mediated cytotoxicity | KEGG PATHWAY | mmu04650 | 2 (1.92%) | 118 (1.37%) | 0.418859386307 | 0.785798182485 | Fas  Kras | web link | no local link |
| Thyroid hormone signaling pathway | KEGG PATHWAY | mmu04919 | 2 (1.92%) | 118 (1.37%) | 0.418859386307 | 0.785798182485 | Esr1  Kras | web link | no local link |
| Proteasome | KEGG PATHWAY | mmu03050 | 1 (0.96%) | 46 (0.53%) | 0.42914631558 | 0.785798182485 | Psmc6 | web link | no local link |
| Chemokine signaling pathway | KEGG PATHWAY | mmu04062 | 3 (2.88%) | 198 (2.30%) | 0.430033897864 | 0.785798182485 | Cdc42  Ccl27a  Kras | web link | no local link |
| Neurotrophin signaling pathway | KEGG PATHWAY | mmu04722 | 2 (1.92%) | 121 (1.41%) | 0.431377666475 | 0.785798182485 | Cdc42  Kras | web link | no local link |
| Focal adhesion | KEGG PATHWAY | mmu04510 | 3 (2.88%) | 199 (2.31%) | 0.433250298319 | 0.785798182485 | Flnb  Cdc42  Ppp1ca | web link | no local link |
| Mineral absorption | KEGG PATHWAY | mmu04978 | 1 (0.96%) | 47 (0.55%) | 0.436080301001 | 0.785798182485 | Slc11a2 | web link | no local link |
| Yersinia infection | KEGG PATHWAY | mmu05135 | 2 (1.92%) | 123 (1.43%) | 0.439644911209 | 0.785798182485 | Baiap2  Cdc42 | web link | no local link |
| ABC transporters | KEGG PATHWAY | mmu02010 | 1 (0.96%) | 48 (0.56%) | 0.442930861531 | 0.785798182485 | Abcg8 | web link | no local link |
| Notch signaling pathway | KEGG PATHWAY | mmu04330 | 1 (0.96%) | 49 (0.57%) | 0.449698991251 | 0.785798182485 | Ctbp1 | web link | no local link |
| Amino sugar and nucleotide sugar metabolism | KEGG PATHWAY | mmu00520 | 1 (0.96%) | 49 (0.57%) | 0.449698991251 | 0.785798182485 | Fpgt | web link | no local link |
| Cholesterol metabolism | KEGG PATHWAY | mmu04979 | 1 (0.96%) | 49 (0.57%) | 0.449698991251 | 0.785798182485 | Abcg8 | web link | no local link |
| Cysteine and methionine metabolism | KEGG PATHWAY | mmu00270 | 1 (0.96%) | 52 (0.60%) | 0.469518561227 | 0.785798182485 | Ahcyl1 | web link | no local link |
| Cytokine-cytokine receptor interaction | KEGG PATHWAY | mmu04060 | 4 (3.85%) | 293 (3.40%) | 0.474639834239 | 0.785798182485 | Fas  Ccl27a  Lifr  Ghr | web link | no local link |
| FoxO signaling pathway | KEGG PATHWAY | mmu04068 | 2 (1.92%) | 132 (1.53%) | 0.476023310133 | 0.785798182485 | Stk11  Kras | web link | no local link |
| Kaposi sarcoma-associated herpesvirus infection | KEGG PATHWAY | mmu05167 | 3 (2.88%) | 217 (2.52%) | 0.489916282944 | 0.785798182485 | Fas  Irf9  Kras | web link | no local link |
| Purine metabolism | KEGG PATHWAY | mmu00230 | 2 (1.92%) | 136 (1.58%) | 0.491735001068 | 0.785798182485 | Enpp4  Entpd8 | web link | no local link |
| Valine, leucine and isoleucine degradation | KEGG PATHWAY | mmu00280 | 1 (0.96%) | 56 (0.65%) | 0.494848910249 | 0.785798182485 | Auh | web link | no local link |
| Ovarian steroidogenesis | KEGG PATHWAY | mmu04913 | 1 (0.96%) | 57 (0.66%) | 0.500992001144 | 0.785798182485 | Hsd3b2 | web link | no local link |
| Endocrine and other factor-regulated calcium reabsorption | KEGG PATHWAY | mmu04961 | 1 (0.96%) | 57 (0.66%) | 0.500992001144 | 0.785798182485 | Esr1 | web link | no local link |
| Endometrial cancer | KEGG PATHWAY | mmu05213 | 1 (0.96%) | 58 (0.67%) | 0.507061096206 | 0.785798182485 | Kras | web link | no local link |
| Pyrimidine metabolism | KEGG PATHWAY | mmu00240 | 1 (0.96%) | 58 (0.67%) | 0.507061096206 | 0.785798182485 | Entpd8 | web link | no local link |
| Vascular smooth muscle contraction | KEGG PATHWAY | mmu04270 | 2 (1.92%) | 140 (1.63%) | 0.507152142289 | 0.785798182485 | Adra1a  Ppp1ca | web link | no local link |
| Fluid shear stress and atherosclerosis | KEGG PATHWAY | mmu05418 | 2 (1.92%) | 143 (1.66%) | 0.518517056619 | 0.785798182485 | Mgst1  Gsta3 | web link | no local link |
| Parkinson disease | KEGG PATHWAY | mmu05012 | 2 (1.92%) | 144 (1.67%) | 0.522267102323 | 0.785798182485 | Atp5j  Ube2j2 | web link | no local link |
| Measles | KEGG PATHWAY | mmu05162 | 2 (1.92%) | 144 (1.67%) | 0.522267102323 | 0.785798182485 | Fas  Irf9 | web link | no local link |
| Long-term depression | KEGG PATHWAY | mmu04730 | 1 (0.96%) | 61 (0.71%) | 0.52483318195 | 0.785798182485 | Kras | web link | no local link |
| Longevity regulating pathway - multiple species | KEGG PATHWAY | mmu04213 | 1 (0.96%) | 62 (0.72%) | 0.530615017574 | 0.785798182485 | Kras | web link | no local link |
| Mitophagy - animal | KEGG PATHWAY | mmu04137 | 1 (0.96%) | 63 (0.73%) | 0.536327168074 | 0.785798182485 | Kras | web link | no local link |
| Phospholipase D signaling pathway | KEGG PATHWAY | mmu04072 | 2 (1.92%) | 149 (1.73%) | 0.540726440571 | 0.785798182485 | Tsc1  Kras | web link | no local link |
| Allograft rejection | KEGG PATHWAY | mmu05330 | 1 (0.96%) | 64 (0.74%) | 0.541970465267 | 0.785798182485 | Fas | web link | no local link |
| Gastric cancer | KEGG PATHWAY | mmu05226 | 2 (1.92%) | 150 (1.74%) | 0.544359599955 | 0.785798182485 | Csnk1a1  Kras | web link | no local link |
| Graft-versus-host disease | KEGG PATHWAY | mmu05332 | 1 (0.96%) | 65 (0.76%) | 0.54754573114 | 0.785798182485 | Fas | web link | no local link |
| Non-small cell lung cancer | KEGG PATHWAY | mmu05223 | 1 (0.96%) | 66 (0.77%) | 0.553053777957 | 0.785798182485 | Kras | web link | no local link |
| Human immunodeficiency virus 1 infection | KEGG PATHWAY | mmu05170 | 3 (2.88%) | 239 (2.78%) | 0.55528622404 | 0.785798182485 | Fas  Eloc  Kras | web link | no local link |
| Hippo signaling pathway | KEGG PATHWAY | mmu04390 | 2 (1.92%) | 154 (1.79%) | 0.558694431836 | 0.785798182485 | Nf2  Ppp1ca | web link | no local link |
| Oxytocin signaling pathway | KEGG PATHWAY | mmu04921 | 2 (1.92%) | 154 (1.79%) | 0.558694431836 | 0.785798182485 | Ppp1ca  Kras | web link | no local link |
| Amphetamine addiction | KEGG PATHWAY | mmu05031 | 1 (0.96%) | 68 (0.79%) | 0.563871415586 | 0.785798182485 | Ppp1ca | web link | no local link |
| Fc epsilon RI signaling pathway | KEGG PATHWAY | mmu04664 | 1 (0.96%) | 68 (0.79%) | 0.563871415586 | 0.785798182485 | Kras | web link | no local link |
| Acute myeloid leukemia | KEGG PATHWAY | mmu05221 | 1 (0.96%) | 69 (0.80%) | 0.569182583359 | 0.785798182485 | Kras | web link | no local link |
| Type I diabetes mellitus | KEGG PATHWAY | mmu04940 | 1 (0.96%) | 70 (0.81%) | 0.57442968622 | 0.785798182485 | Fas | web link | no local link |
| Cushing syndrome | KEGG PATHWAY | mmu04934 | 2 (1.92%) | 159 (1.85%) | 0.576164341595 | 0.785798182485 | Hsd3b2  Mrap | web link | no local link |
| p53 signaling pathway | KEGG PATHWAY | mmu04115 | 1 (0.96%) | 71 (0.82%) | 0.579613489527 | 0.785798182485 | Fas | web link | no local link |
| Melanoma | KEGG PATHWAY | mmu05218 | 1 (0.96%) | 72 (0.84%) | 0.584734749582 | 0.785798182485 | Kras | web link | no local link |
| Bile secretion | KEGG PATHWAY | mmu04976 | 1 (0.96%) | 72 (0.84%) | 0.584734749582 | 0.785798182485 | Abcg8 | web link | no local link |
| Hepatitis B | KEGG PATHWAY | mmu05161 | 2 (1.92%) | 163 (1.89%) | 0.589778711367 | 0.785798182485 | Fas  Kras | web link | no local link |
| Thyroid hormone synthesis | KEGG PATHWAY | mmu04918 | 1 (0.96%) | 73 (0.85%) | 0.589794213739 | 0.785798182485 | Asgr2 | web link | no local link |
| Inositol phosphate metabolism | KEGG PATHWAY | mmu00562 | 1 (0.96%) | 73 (0.85%) | 0.589794213739 | 0.785798182485 | Cdipt | web link | no local link |
| Influenza A | KEGG PATHWAY | mmu05164 | 2 (1.92%) | 164 (1.91%) | 0.593131909339 | 0.785798182485 | Fas  Irf9 | web link | no local link |
| Bacterial invasion of epithelial cells | KEGG PATHWAY | mmu05100 | 1 (0.96%) | 74 (0.86%) | 0.594792620507 | 0.785798182485 | Cdc42 | web link | no local link |
| Glioma | KEGG PATHWAY | mmu05214 | 1 (0.96%) | 75 (0.87%) | 0.599730699658 | 0.785798182485 | Kras | web link | no local link |
| Human cytomegalovirus infection | KEGG PATHWAY | mmu05163 | 3 (2.88%) | 256 (2.97%) | 0.602312312463 | 0.785798182485 | Fas  Tsc1  Kras | web link | no local link |
| Salivary secretion | KEGG PATHWAY | mmu04970 | 1 (0.96%) | 78 (0.91%) | 0.614190140131 | 0.791599676838 | Adra1a | web link | no local link |
| Autoimmune thyroid disease | KEGG PATHWAY | mmu05320 | 1 (0.96%) | 79 (0.92%) | 0.618894035257 | 0.791599676838 | Fas | web link | no local link |
| cGMP-PKG signaling pathway | KEGG PATHWAY | mmu04022 | 2 (1.92%) | 173 (2.01%) | 0.622402349509 | 0.791599676838 | Adra1a  Ppp1ca | web link | no local link |
| Alzheimer disease | KEGG PATHWAY | mmu05010 | 2 (1.92%) | 175 (2.03%) | 0.628685111508 | 0.791599676838 | Fas  Atp5j | web link | no local link |
| B cell receptor signaling pathway | KEGG PATHWAY | mmu04662 | 1 (0.96%) | 83 (0.96%) | 0.637148306684 | 0.791599676838 | Kras | web link | no local link |
| ErbB signaling pathway | KEGG PATHWAY | mmu04012 | 1 (0.96%) | 84 (0.98%) | 0.641574884527 | 0.791599676838 | Kras | web link | no local link |
| Gap junction | KEGG PATHWAY | mmu04540 | 1 (0.96%) | 86 (1.00%) | 0.6502682143 | 0.791599676838 | Kras | web link | no local link |
| PD-L1 expression and PD-1 checkpoint pathway in cancer | KEGG PATHWAY | mmu05235 | 1 (0.96%) | 88 (1.02%) | 0.658752672561 | 0.791599676838 | Kras | web link | no local link |
| Colorectal cancer | KEGG PATHWAY | mmu05210 | 1 (0.96%) | 88 (1.02%) | 0.658752672561 | 0.791599676838 | Kras | web link | no local link |
| Complement and coagulation cascades | KEGG PATHWAY | mmu04610 | 1 (0.96%) | 88 (1.02%) | 0.658752672561 | 0.791599676838 | Cfh | web link | no local link |
| Arachidonic acid metabolism | KEGG PATHWAY | mmu00590 | 1 (0.96%) | 89 (1.03%) | 0.662918132896 | 0.791599676838 | Cyp4f13 | web link | no local link |
| Viral myocarditis | KEGG PATHWAY | mmu05416 | 1 (0.96%) | 89 (1.03%) | 0.662918132896 | 0.791599676838 | Eif4g3 | web link | no local link |
| Steroid hormone biosynthesis | KEGG PATHWAY | mmu00140 | 1 (0.96%) | 89 (1.03%) | 0.662918132896 | 0.791599676838 | Hsd3b2 | web link | no local link |
| GABAergic synapse | KEGG PATHWAY | mmu04727 | 1 (0.96%) | 90 (1.05%) | 0.667033230229 | 0.791599676838 | Slc38a3 | web link | no local link |
| Progesterone-mediated oocyte maturation | KEGG PATHWAY | mmu04914 | 1 (0.96%) | 90 (1.05%) | 0.667033230229 | 0.791599676838 | Kras | web link | no local link |
| Retinol metabolism | KEGG PATHWAY | mmu00830 | 1 (0.96%) | 91 (1.06%) | 0.671098567638 | 0.791655196555 | Rdh11 | web link | no local link |
| Huntington disease | KEGG PATHWAY | mmu05016 | 2 (1.92%) | 193 (2.24%) | 0.68164376008 | 0.79930845676 | Atp5j  Dctn4 | web link | no local link |
| Prostate cancer | KEGG PATHWAY | mmu05215 | 1 (0.96%) | 97 (1.13%) | 0.694478515517 | 0.80437269234 | Kras | web link | no local link |
| Alcoholism | KEGG PATHWAY | mmu05034 | 2 (1.92%) | 199 (2.31%) | 0.697888953724 | 0.80437269234 | Ppp1ca  Kras | web link | no local link |
| Phosphatidylinositol signaling system | KEGG PATHWAY | mmu04070 | 1 (0.96%) | 98 (1.14%) | 0.698211829391 | 0.80437269234 | Cdipt | web link | no local link |
| Melanogenesis | KEGG PATHWAY | mmu04916 | 1 (0.96%) | 100 (1.16%) | 0.705543441336 | 0.806905898861 | Kras | web link | no local link |
| Viral protein interaction with cytokine and cytokine receptor | KEGG PATHWAY | mmu04061 | 1 (0.96%) | 101 (1.17%) | 0.709142816289 | 0.806905898861 | Ccl27a | web link | no local link |
| Aldosterone synthesis and secretion | KEGG PATHWAY | mmu04925 | 1 (0.96%) | 102 (1.18%) | 0.712698611177 | 0.806905898861 | Hsd3b2 | web link | no local link |
| NF-kappa B signaling pathway | KEGG PATHWAY | mmu04064 | 1 (0.96%) | 108 (1.25%) | 0.733147033909 | 0.8253140896 | Cflar | web link | no local link |
| Insulin resistance | KEGG PATHWAY | mmu04931 | 1 (0.96%) | 110 (1.28%) | 0.739637513892 | 0.826510962907 | Ppp1ca | web link | no local link |
| Cholinergic synapse | KEGG PATHWAY | mmu04725 | 1 (0.96%) | 113 (1.31%) | 0.749081187085 | 0.826510962907 | Kras | web link | no local link |
| HIF-1 signaling pathway | KEGG PATHWAY | mmu04066 | 1 (0.96%) | 114 (1.32%) | 0.752153059545 | 0.826510962907 | Eloc | web link | no local link |
| Glutamatergic synapse | KEGG PATHWAY | mmu04724 | 1 (0.96%) | 114 (1.32%) | 0.752153059545 | 0.826510962907 | Slc38a3 | web link | no local link |
| Leukocyte transendothelial migration | KEGG PATHWAY | mmu04670 | 1 (0.96%) | 115 (1.34%) | 0.755187681844 | 0.826510962907 | Cdc42 | web link | no local link |
| Oocyte meiosis | KEGG PATHWAY | mmu04114 | 1 (0.96%) | 119 (1.38%) | 0.766962537405 | 0.834760330766 | Ppp1ca | web link | no local link |
| Lysosome | KEGG PATHWAY | mmu04142 | 1 (0.96%) | 124 (1.44%) | 0.780895114165 | 0.842781421682 | Slc11a2 | web link | no local link |
| Platelet activation | KEGG PATHWAY | mmu04611 | 1 (0.96%) | 125 (1.45%) | 0.783580982638 | 0.842781421682 | Ppp1ca | web link | no local link |
| Inflammatory mediator regulation of TRP channels | KEGG PATHWAY | mmu04750 | 1 (0.96%) | 127 (1.48%) | 0.788855276866 | 0.842781421682 | Ppp1ca | web link | no local link |
| Osteoclast differentiation | KEGG PATHWAY | mmu04380 | 1 (0.96%) | 128 (1.49%) | 0.791444482291 | 0.842781421682 | Irf9 | web link | no local link |
| Relaxin signaling pathway | KEGG PATHWAY | mmu04926 | 1 (0.96%) | 131 (1.52%) | 0.7990249129 | 0.844351032492 | Kras | web link | no local link |
| Serotonergic synapse | KEGG PATHWAY | mmu04726 | 1 (0.96%) | 132 (1.53%) | 0.80149057399 | 0.844351032492 | Kras | web link | no local link |
| Oxidative phosphorylation | KEGG PATHWAY | mmu00190 | 1 (0.96%) | 134 (1.56%) | 0.806332368948 | 0.84493338661 | Atp5j | web link | no local link |
| Apelin signaling pathway | KEGG PATHWAY | mmu04371 | 1 (0.96%) | 139 (1.61%) | 0.817931144735 | 0.852552568851 | Kras | web link | no local link |
| Systemic lupus erythematosus | KEGG PATHWAY | mmu05322 | 1 (0.96%) | 143 (1.66%) | 0.826712560362 | 0.857170391533 | Ssb | web link | no local link |
| MicroRNAs in cancer | KEGG PATHWAY | mmu05206 | 2 (1.92%) | 282 (3.28%) | 0.860068680873 | 0.887086545193 | Fgfr3  Kras | web link | no local link |
| Cell adhesion molecules (CAMs) | KEGG PATHWAY | mmu04514 | 1 (0.96%) | 171 (1.99%) | 0.877481790999 | 0.900332879307 | Cadm1 | web link | no local link |
| Calcium signaling pathway | KEGG PATHWAY | mmu04020 | 1 (0.96%) | 194 (2.25%) | 0.907924419617 | 0.926741506034 | Adra1a | web link | no local link |
| NOD-like receptor signaling pathway | KEGG PATHWAY | mmu04621 | 1 (0.96%) | 206 (2.39%) | 0.920698028381 | 0.932294757314 | Irf9 | web link | no local link |
| cAMP signaling pathway | KEGG PATHWAY | mmu04024 | 1 (0.96%) | 213 (2.47%) | 0.927321551696 | 0.932294757314 | Ppp1ca | web link | no local link |
| Neuroactive ligand-receptor interaction | KEGG PATHWAY | mmu04080 | 2 (1.92%) | 348 (4.04%) | 0.927562296617 | 0.932294757314 | Adra1a  Ghr | web link | no local link |
| Human T-cell leukemia virus 1 infection | KEGG PATHWAY | mmu05166 | 1 (0.96%) | 246 (2.86%) | 0.951870958876 | 0.951870958876 | Kras | web link | no local link |
