# Supplementary material for: Regulation of Alternative Splicing of Lipid Metabolism Genes in Sepsis-Induced Liver Damage by RNA-Binding Proteins
Source: Inflammation. 2024 May 9;47(6):1952–68. doi: 10.1007/s10753-024-02017-2 (PMC11607010; doi:10.1007/s10753-024-02017-2)
Supplement: Supplementary file 1 — Supplementary file1 (ZIP 38655 KB) [file 10753_2024_2017_MOESM1_ESM.zip › Sup6_AS_KEGG.zip!/Sup6_AS_KEGG/ras/di_RAS/GO_KEGG/CLP_vs_Sham_and_CLPDCA_vs_Sham_di_RAS/CLPDCA_vs_Sham_diDown_nir_KEGG_analysis/CLPDCA_vs_Sham_diDown_nir_KEGG_top.pdf]

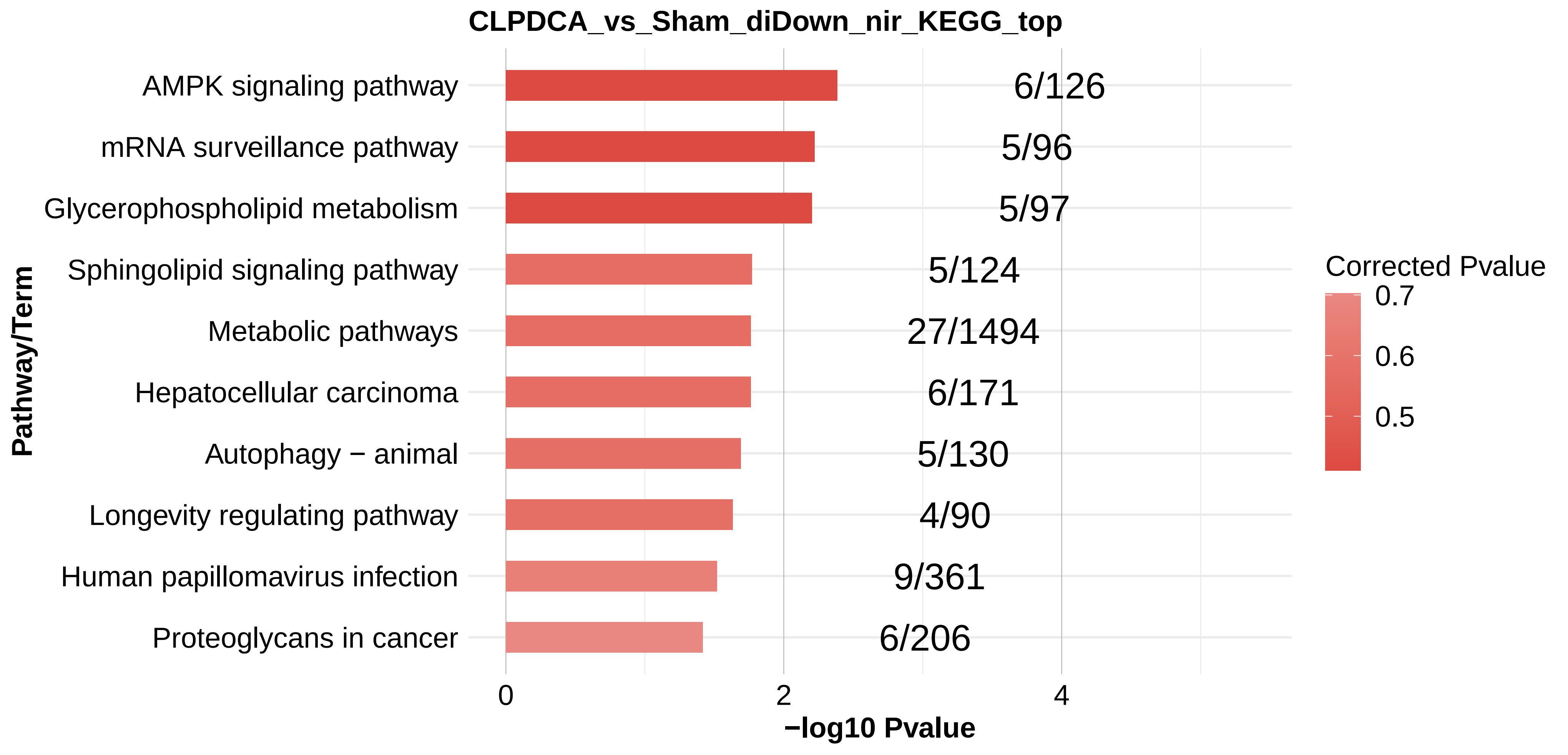
CLPDCA_vs_Sham_diDown_nir_KEGG_top
Pathway/Term
AMPK signaling pathway
mRNA surveillance pathway
Glycerophospholipid metabolism
Sphingolipid signaling pathway
Metabolic pathways
Hepatocellular carcinoma
Autophagy − animal
Longevity regulating pathway
Human papillomavirus infection
Proteoglycans in cancer
6/126
5/96
5/97
5/124
27/1494
6/171
5/130
4/90
9/361
6/206
0
2
4
−log10 Pvalue
Corrected Pvalue
0.7
0.6
0.5
