# Supplementary material for: Regulation of Alternative Splicing of Lipid Metabolism Genes in Sepsis-Induced Liver Damage by RNA-Binding Proteins
Source: Inflammation. 2024 May 9;47(6):1952–68. doi: 10.1007/s10753-024-02017-2 (PMC11607010; doi:10.1007/s10753-024-02017-2)
Supplement: Supplementary file 1 — Supplementary file1 (ZIP 38655 KB) [file 10753_2024_2017_MOESM1_ESM.zip › Sup6_AS_KEGG.zip!/Sup6_AS_KEGG/ras/di_RAS/GO_KEGG/CLP_vs_Sham_and_CLPDCA_vs_Sham_di_RAS/CLP_vs_Sham_diDown_nir_KEGG_analysis/CLP_vs_Sham_diDown_nir_KEGG_pathway.html]

KEGG pathway enrichment analyze result


# KEGG pathway enrichment analyze result


| Term | Database | ID | Input number (166) | Background number (8608) | P-Value | Corrected P-Value | Input | Hyperlink | img |
| --- | --- | --- | --- | --- | --- | --- | --- | --- | --- |
| Metabolic pathways | KEGG PATHWAY | mmu01100 | 47 (28.31%) | 1494 (17.36%) | 0.000278450124561 | 0.0418751986842 | Grhpr  Ndufa5  Cers2  Uckl1  Acox1  Entpd5  Ugt1a6b  Aox3  Ugt2b36  Bdh2  Mtmr2  Tm7sf2  Smpd4  Acacb  Inpp4a  Adk  Pigp  Mocs2  Mocs1  Khk  Ndufs6  Mri1  Cyp2r1  Mccc2  Echdc1  Acp5  Gba  Kynu  Pfkfb2  Aspdh  Mgat1  Gmppa  Uox  Mpst  P4ha1  Ggt6  Hsd17b2  Hsd17b6  Upp2  Lipc  Papss2  Maob  Flad1  Enpp3  Pnpla2  Ugt1a7c  Cox7a2 | web link | no local link |
| Riboflavin metabolism | KEGG PATHWAY | mmu00740 | 3 (1.81%) | 8 (0.09%) | 0.000367326304247 | 0.0418751986842 | Acp5  Flad1  Enpp3 | web link | no local link |
| Drug metabolism - cytochrome P450 | KEGG PATHWAY | mmu00982 | 6 (3.61%) | 68 (0.79%) | 0.00191539245126 | 0.145569826295 | Aox3  Ugt1a6b  Fmo5  Maob  Ugt1a7c  Ugt2b36 | web link | no local link |
| Drug metabolism - other enzymes | KEGG PATHWAY | mmu00983 | 6 (3.61%) | 87 (1.01%) | 0.0065516707723 | 0.294587943759 | Upp2  Uckl1  Ugt1a6b  Ugt1a7c  Ugt2b36  Ces1f | web link | no local link |
| Steroid hormone biosynthesis | KEGG PATHWAY | mmu00140 | 6 (3.61%) | 89 (1.03%) | 0.00730639342713 | 0.294587943759 | Cyp2d10  Ugt1a6b  Ugt1a7c  Ugt2b36  Hsd17b2  Hsd17b6 | web link | no local link |
| Porphyrin and chlorophyll metabolism | KEGG PATHWAY | mmu00860 | 4 (2.41%) | 41 (0.48%) | 0.00775231430944 | 0.294587943759 | Ugt2b36  Ugt1a6b  Cp  Ugt1a7c | web link | no local link |
| Sulfur relay system | KEGG PATHWAY | mmu04122 | 2 (1.20%) | 8 (0.09%) | 0.00958975544667 | 0.312352034549 | Mocs2  Mpst | web link | no local link |
| Autophagy - animal | KEGG PATHWAY | mmu04140 | 7 (4.22%) | 130 (1.51%) | 0.0127360745389 | 0.334460019421 | Ulk1  Rragc  Atg16l1  Atg16l2  Akt1s1  Atg4d  Mapk9 | web link | no local link |
| Protein processing in endoplasmic reticulum | KEGG PATHWAY | mmu04141 | 8 (4.82%) | 163 (1.89%) | 0.0134461044924 | 0.334460019421 | Sec24c  Dnajc5  Derl1  Ube2g2  Fbxo6  Sec24b  Mapk9  Uggt1 | web link | no local link |
| Ascorbate and aldarate metabolism | KEGG PATHWAY | mmu00053 | 3 (1.81%) | 27 (0.31%) | 0.0146692990974 | 0.334460019421 | Ugt2b36  Ugt1a6b  Ugt1a7c | web link | no local link |
| Ubiquitin mediated proteolysis | KEGG PATHWAY | mmu04120 | 7 (4.22%) | 139 (1.61%) | 0.0178355967102 | 0.344578184818 | Btrc  Huwe1  Anapc10  Nedd4l  Ube2g2  Rhobtb1  Anapc5 | web link | no local link |
| Sulfur metabolism | KEGG PATHWAY | mmu00920 | 2 (1.20%) | 11 (0.13%) | 0.0181356939378 | 0.344578184818 | Mpst  Papss2 | web link | no local link |
| Propanoate metabolism | KEGG PATHWAY | mmu00640 | 3 (1.81%) | 33 (0.38%) | 0.0251640840725 | 0.360083400395 | Acox1  Echdc1  Acacb | web link | no local link |
| Pyrimidine metabolism | KEGG PATHWAY | mmu00240 | 4 (2.41%) | 58 (0.67%) | 0.0252675599507 | 0.360083400395 | Upp2  Entpd5  Enpp3  Uckl1 | web link | no local link |
| Non-alcoholic fatty liver disease (NAFLD) | KEGG PATHWAY | mmu04932 | 7 (4.22%) | 151 (1.75%) | 0.026673363301 | 0.360083400395 | Ndufa5  Mlxipl  Srebf1  Ndufs6  Cox7a2  Mapk9  Nr1h3 | web link | no local link |
| Pentose and glucuronate interconversions | KEGG PATHWAY | mmu00040 | 3 (1.81%) | 34 (0.39%) | 0.02721802925 | 0.360083400395 | Ugt2b36  Ugt1a6b  Ugt1a7c | web link | no local link |
| Oocyte meiosis | KEGG PATHWAY | mmu04114 | 6 (3.61%) | 119 (1.38%) | 0.0273250093105 | 0.360083400395 | Ywhaz  Btrc  Anapc10  Cpeb3  Anapc5  Ppp2r5c | web link | no local link |
| Fructose and mannose metabolism | KEGG PATHWAY | mmu00051 | 3 (1.81%) | 35 (0.41%) | 0.0293588580573 | 0.360083400395 | Pfkfb2  Khk  Gmppa | web link | no local link |
| Retinol metabolism | KEGG PATHWAY | mmu00830 | 5 (3.01%) | 91 (1.06%) | 0.0309512906021 | 0.360083400395 | Ugt2b36  Ugt1a6b  Aox3  Hsd17b6  Ugt1a7c | web link | no local link |
| Nicotinate and nicotinamide metabolism | KEGG PATHWAY | mmu00760 | 3 (1.81%) | 36 (0.42%) | 0.0315862631926 | 0.360083400395 | Aspdh  Aox3  Enpp3 | web link | no local link |
| AMPK signaling pathway | KEGG PATHWAY | mmu04152 | 6 (3.61%) | 126 (1.46%) | 0.0347987759081 | 0.377815281288 | Ulk1  Acacb  Pfkfb2  Akt1s1  Srebf1  Ppp2r5c | web link | no local link |
| Ferroptosis | KEGG PATHWAY | mmu04216 | 3 (1.81%) | 41 (0.48%) | 0.0440050330673 | 0.456052160879 | Slc39a8  Cp  Lpcat3 | web link | no local link |
| Ribosome | KEGG PATHWAY | mmu03010 | 7 (4.22%) | 171 (1.99%) | 0.0473148234009 | 0.469033901539 | Rpl22l1  Mrpl24  Rps10  Mrpl30  Rps19  Rpl35a  Rpl18 | web link | no local link |
| Steroid biosynthesis | KEGG PATHWAY | mmu00100 | 2 (1.20%) | 19 (0.22%) | 0.0510048417162 | 0.478410804099 | Cyp2r1  Tm7sf2 | web link | no local link |
| Hedgehog signaling pathway | KEGG PATHWAY | mmu04340 | 3 (1.81%) | 44 (0.51%) | 0.0524573250109 | 0.478410804099 | Csnk1g3  Btrc  Arrb2 | web link | no local link |
| Insulin resistance | KEGG PATHWAY | mmu04931 | 5 (3.01%) | 110 (1.28%) | 0.0611896377032 | 0.528023997649 | Mlxipl  Mapk9  Srebf1  Acacb  Nr1h3 | web link | no local link |
| Sphingolipid metabolism | KEGG PATHWAY | mmu00600 | 3 (1.81%) | 48 (0.56%) | 0.0648450523429 | 0.528023997649 | Smpd4  Cers2  Gba | web link | no local link |
| Tryptophan metabolism | KEGG PATHWAY | mmu00380 | 3 (1.81%) | 48 (0.56%) | 0.0648450523429 | 0.528023997649 | Aox3  Maob  Kynu | web link | no local link |
| Folate biosynthesis | KEGG PATHWAY | mmu00790 | 2 (1.20%) | 26 (0.30%) | 0.0888903011967 | 0.698861678374 | Mocs2  Mocs1 | web link | no local link |
| Progesterone-mediated oocyte maturation | KEGG PATHWAY | mmu04914 | 4 (2.41%) | 90 (1.05%) | 0.0956510105347 | 0.725376191896 | Mapk9  Anapc5  Anapc10  Cpeb3 | web link | no local link |
| IL-17 signaling pathway | KEGG PATHWAY | mmu04657 | 4 (2.41%) | 91 (1.06%) | 0.0986257103017 | 0.725376191896 | Mapk15  Mapk9  Mapk6  Anapc5 | web link | no local link |
| Tight junction | KEGG PATHWAY | mmu04530 | 6 (3.61%) | 167 (1.94%) | 0.103724990409 | 0.739040556668 | Pard3  Igsf5  Myl6  Nedd4l  Mapk9  Mpdz | web link | no local link |
| Caffeine metabolism | KEGG PATHWAY | mmu00232 | 1 (0.60%) | 6 (0.07%) | 0.1102998794 | 0.762071894037 | Uox | web link | no local link |
| Mitophagy - animal | KEGG PATHWAY | mmu04137 | 3 (1.81%) | 63 (0.73%) | 0.121424291801 | 0.796552458043 | Ulk1  Mapk9  Tbc1d17 | web link | no local link |
| Purine metabolism | KEGG PATHWAY | mmu00230 | 5 (3.01%) | 136 (1.58%) | 0.122277789612 | 0.796552458043 | Uox  Entpd5  Papss2  Enpp3  Adk | web link | no local link |
| Autophagy - other | KEGG PATHWAY | mmu04136 | 2 (1.20%) | 32 (0.37%) | 0.126049150002 | 0.798311283345 | Atg16l1  Atg4d | web link | no local link |
| Metabolism of xenobiotics by cytochrome P450 | KEGG PATHWAY | mmu00980 | 3 (1.81%) | 66 (0.77%) | 0.134385558698 | 0.828105604949 | Ugt2b36  Ugt1a6b  Ugt1a7c | web link | no local link |
| Primary immunodeficiency | KEGG PATHWAY | mmu05340 | 2 (1.20%) | 36 (0.42%) | 0.152513270397 | 0.891859440513 | Dclre1c  Rfxap | web link | no local link |
| Vitamin B6 metabolism | KEGG PATHWAY | mmu00750 | 1 (0.60%) | 9 (0.10%) | 0.160825908668 | 0.891859440513 | Aox3 | web link | no local link |
| Aldosterone-regulated sodium reabsorption | KEGG PATHWAY | mmu04960 | 2 (1.20%) | 38 (0.44%) | 0.166124072632 | 0.891859440513 | Nedd4l  Sgk1 | web link | no local link |
| Pyruvate metabolism | KEGG PATHWAY | mmu00620 | 2 (1.20%) | 38 (0.44%) | 0.166124072632 | 0.891859440513 | Grhpr  Acacb | web link | no local link |
| Pancreatic cancer | KEGG PATHWAY | mmu05212 | 3 (1.81%) | 75 (0.87%) | 0.175839001443 | 0.891859440513 | Mapk9  Vegfa  Ralbp1 | web link | no local link |
| Hippo signaling pathway | KEGG PATHWAY | mmu04390 | 5 (3.01%) | 154 (1.79%) | 0.176414917295 | 0.891859440513 | Ywhaz  Btrc  Pard3  Tcf7l2  Yap1 | web link | no local link |
| mTOR signaling pathway | KEGG PATHWAY | mmu04150 | 5 (3.01%) | 155 (1.80%) | 0.179661158065 | 0.891859440513 | Sgk1  Ulk1  Akt1s1  Lamtor2  Rragc | web link | no local link |
| Tyrosine metabolism | KEGG PATHWAY | mmu00350 | 2 (1.20%) | 40 (0.46%) | 0.179936553788 | 0.891859440513 | Maob  Aox3 | web link | no local link |
| Glycine, serine and threonine metabolism | KEGG PATHWAY | mmu00260 | 2 (1.20%) | 40 (0.46%) | 0.179936553788 | 0.891859440513 | Maob  Grhpr | web link | no local link |
| Taurine and hypotaurine metabolism | KEGG PATHWAY | mmu00430 | 1 (0.60%) | 11 (0.13%) | 0.192914814904 | 0.916345370793 | Ggt6 | web link | no local link |
| Synthesis and degradation of ketone bodies | KEGG PATHWAY | mmu00072 | 1 (0.60%) | 11 (0.13%) | 0.192914814904 | 0.916345370793 | Bdh2 | web link | no local link |
| Cell cycle | KEGG PATHWAY | mmu04110 | 4 (2.41%) | 123 (1.43%) | 0.213178980654 | 0.977018966705 | Abl1  Ywhaz  Anapc5  Anapc10 | web link | no local link |
| Lysosome | KEGG PATHWAY | mmu04142 | 4 (2.41%) | 124 (1.44%) | 0.217239938802 | 0.977018966705 | Acp5  Nagpa  Gba  Ctsa | web link | no local link |
| Non-homologous end-joining | KEGG PATHWAY | mmu03450 | 1 (0.60%) | 13 (0.15%) | 0.22378379395 | 0.977018966705 | Dclre1c | web link | no local link |
| ECM-receptor interaction | KEGG PATHWAY | mmu04512 | 3 (1.81%) | 86 (1.00%) | 0.230486113396 | 0.977018966705 | Cd47  Fn1  Itgav | web link | no local link |
| PPAR signaling pathway | KEGG PATHWAY | mmu03320 | 3 (1.81%) | 87 (1.01%) | 0.235610999196 | 0.977018966705 | Dbi  Acox1  Nr1h3 | web link | no local link |
| Cholesterol metabolism | KEGG PATHWAY | mmu04979 | 2 (1.20%) | 49 (0.57%) | 0.243704355512 | 0.977018966705 | Gm44805  Lipc | web link | no local link |
| Arginine and proline metabolism | KEGG PATHWAY | mmu00330 | 2 (1.20%) | 50 (0.58%) | 0.250879594668 | 0.977018966705 | Maob  P4ha1 | web link | no local link |
| Spliceosome | KEGG PATHWAY | mmu03040 | 4 (2.41%) | 133 (1.55%) | 0.254658305207 | 0.977018966705 | U2af1  Txnl4a  Hnrnpk  Snrnp70 | web link | no local link |
| Dopaminergic synapse | KEGG PATHWAY | mmu04728 | 4 (2.41%) | 135 (1.57%) | 0.263155128759 | 0.977018966705 | Maob  Mapk9  Arrb2  Ppp2r5c | web link | no local link |
| Cysteine and methionine metabolism | KEGG PATHWAY | mmu00270 | 2 (1.20%) | 52 (0.60%) | 0.26524556371 | 0.977018966705 | Mpst  Mri1 | web link | no local link |
| Chemical carcinogenesis | KEGG PATHWAY | mmu05204 | 3 (1.81%) | 94 (1.09%) | 0.271979689014 | 0.977018966705 | Ugt2b36  Ugt1a6b  Ugt1a7c | web link | no local link |
| Selenocompound metabolism | KEGG PATHWAY | mmu00450 | 1 (0.60%) | 17 (0.20%) | 0.282044668699 | 0.977018966705 | Papss2 | web link | no local link |
| Glycerophospholipid metabolism | KEGG PATHWAY | mmu00564 | 3 (1.81%) | 97 (1.13%) | 0.287762256803 | 0.977018966705 | Pnpla6  Taz  Lpcat3 | web link | no local link |
| Thermogenesis | KEGG PATHWAY | mmu04714 | 6 (3.61%) | 232 (2.70%) | 0.290449465891 | 0.977018966705 | Ndufa5  Pnpla2  Akt1s1  Ndufs6  Smarca2  Cox7a2 | web link | no local link |
| Phosphatidylinositol signaling system | KEGG PATHWAY | mmu04070 | 3 (1.81%) | 98 (1.14%) | 0.293040445163 | 0.977018966705 | Pip4p2  Mtmr2  Inpp4a | web link | no local link |
| Valine, leucine and isoleucine degradation | KEGG PATHWAY | mmu00280 | 2 (1.20%) | 56 (0.65%) | 0.293962359204 | 0.977018966705 | Aox3  Mccc2 | web link | no local link |
| Other glycan degradation | KEGG PATHWAY | mmu00511 | 1 (0.60%) | 18 (0.21%) | 0.295917394225 | 0.977018966705 | Gba | web link | no local link |
| Pantothenate and CoA biosynthesis | KEGG PATHWAY | mmu00770 | 1 (0.60%) | 18 (0.21%) | 0.295917394225 | 0.977018966705 | Enpp3 | web link | no local link |
| Parkinson disease | KEGG PATHWAY | mmu05012 | 4 (2.41%) | 144 (1.67%) | 0.301981372423 | 0.977018966705 | Cox7a2  Ndufa5  Ube2g2  Ndufs6 | web link | no local link |
| AGE-RAGE signaling pathway in diabetic complications | KEGG PATHWAY | mmu04933 | 3 (1.81%) | 101 (1.17%) | 0.308911109611 | 0.977018966705 | Mapk9  Vegfa  Fn1 | web link | no local link |
| Fatty acid biosynthesis | KEGG PATHWAY | mmu00061 | 1 (0.60%) | 19 (0.22%) | 0.309523647143 | 0.977018966705 | Acacb | web link | no local link |
| Lysine degradation | KEGG PATHWAY | mmu00310 | 2 (1.20%) | 59 (0.69%) | 0.31540793354 | 0.977018966705 | Kmt5b  Nsd1 | web link | no local link |
| Glycerolipid metabolism | KEGG PATHWAY | mmu00561 | 2 (1.20%) | 61 (0.71%) | 0.329627548607 | 0.977018966705 | Pnpla2  Lipc | web link | no local link |
| Focal adhesion | KEGG PATHWAY | mmu04510 | 5 (3.01%) | 199 (2.31%) | 0.338724876457 | 0.977018966705 | Fn1  Mapk9  Vegfa  Itgav  Zyx | web link | no local link |
| Proximal tubule bicarbonate reclamation | KEGG PATHWAY | mmu04964 | 1 (0.60%) | 22 (0.26%) | 0.348793969991 | 0.977018966705 | Slc38a3 | web link | no local link |
| Phenylalanine metabolism | KEGG PATHWAY | mmu00360 | 1 (0.60%) | 23 (0.27%) | 0.361384256618 | 0.977018966705 | Maob | web link | no local link |
| Histidine metabolism | KEGG PATHWAY | mmu00340 | 1 (0.60%) | 24 (0.28%) | 0.373732563362 | 0.977018966705 | Maob | web link | no local link |
| Wnt signaling pathway | KEGG PATHWAY | mmu04310 | 4 (2.41%) | 162 (1.88%) | 0.381025816329 | 0.977018966705 | Mapk9  Btrc  Tbl1xr1  Tcf7l2 | web link | no local link |
| Glycosylphosphatidylinositol (GPI)-anchor biosynthesis | KEGG PATHWAY | mmu00563 | 1 (0.60%) | 25 (0.29%) | 0.385843513325 | 0.977018966705 | Pigp | web link | no local link |
| alpha-Linolenic acid metabolism | KEGG PATHWAY | mmu00592 | 1 (0.60%) | 25 (0.29%) | 0.385843513325 | 0.977018966705 | Acox1 | web link | no local link |
| PI3K-Akt signaling pathway | KEGG PATHWAY | mmu04151 | 8 (4.82%) | 358 (4.16%) | 0.386417219945 | 0.977018966705 | Ywhaz  Ghr  Sgk1  Prlr  Itgav  Vegfa  Fn1  Ppp2r5c | web link | no local link |
| Jak-STAT signaling pathway | KEGG PATHWAY | mmu04630 | 4 (2.41%) | 165 (1.92%) | 0.394193625741 | 0.977018966705 | Prlr  Aox3  Lifr  Ghr | web link | no local link |
| Hippo signaling pathway - multiple species | KEGG PATHWAY | mmu04392 | 1 (0.60%) | 26 (0.30%) | 0.39772164181 | 0.977018966705 | Yap1 | web link | no local link |
| Adipocytokine signaling pathway | KEGG PATHWAY | mmu04920 | 2 (1.20%) | 71 (0.82%) | 0.399243346334 | 0.977018966705 | Mapk9  Acacb | web link | no local link |
| RNA transport | KEGG PATHWAY | mmu03013 | 4 (2.41%) | 167 (1.94%) | 0.402950553328 | 0.977018966705 | Nup98  Thoc7  Eif5  Eif3e | web link | no local link |
| Prolactin signaling pathway | KEGG PATHWAY | mmu04917 | 2 (1.20%) | 72 (0.84%) | 0.406032196884 | 0.977018966705 | Prlr  Mapk9 | web link | no local link |
| Adherens junction | KEGG PATHWAY | mmu04520 | 2 (1.20%) | 72 (0.84%) | 0.406032196884 | 0.977018966705 | Pard3  Tcf7l2 | web link | no local link |
| Bile secretion | KEGG PATHWAY | mmu04976 | 2 (1.20%) | 72 (0.84%) | 0.406032196884 | 0.977018966705 | Slco1b2  Slc4a2 | web link | no local link |
| Butanoate metabolism | KEGG PATHWAY | mmu00650 | 1 (0.60%) | 27 (0.31%) | 0.409371397981 | 0.977018966705 | Bdh2 | web link | no local link |
| Inositol phosphate metabolism | KEGG PATHWAY | mmu00562 | 2 (1.20%) | 73 (0.85%) | 0.412784395509 | 0.977018966705 | Mtmr2  Inpp4a | web link | no local link |
| Bacterial invasion of epithelial cells | KEGG PATHWAY | mmu05100 | 2 (1.20%) | 74 (0.86%) | 0.419498829201 | 0.977018966705 | Arpc5  Fn1 | web link | no local link |
| Endocytosis | KEGG PATHWAY | mmu04144 | 6 (3.61%) | 270 (3.14%) | 0.421138504216 | 0.977018966705 | Pard3  Arpc5  Nedd4l  Agap3  Arrb2  Chmp3 | web link | no local link |
| Sphingolipid signaling pathway | KEGG PATHWAY | mmu04071 | 3 (1.81%) | 124 (1.44%) | 0.429713518868 | 0.977018966705 | Mapk9  Cers2  Ppp2r5c | web link | no local link |
| Arrhythmogenic right ventricular cardiomyopathy (ARVC) | KEGG PATHWAY | mmu05412 | 2 (1.20%) | 77 (0.89%) | 0.439405239841 | 0.977018966705 | Tcf7l2  Itgav | web link | no local link |
| Circadian rhythm | KEGG PATHWAY | mmu04710 | 1 (0.60%) | 30 (0.35%) | 0.442993666082 | 0.977018966705 | Btrc | web link | no local link |
| Salmonella infection | KEGG PATHWAY | mmu05132 | 2 (1.20%) | 78 (0.91%) | 0.445958577537 | 0.977018966705 | Mapk9  Arpc5 | web link | no local link |
| Glyoxylate and dicarboxylate metabolism | KEGG PATHWAY | mmu00630 | 1 (0.60%) | 31 (0.36%) | 0.453772758112 | 0.977018966705 | Grhpr | web link | no local link |
| Axon guidance | KEGG PATHWAY | mmu04360 | 4 (2.41%) | 180 (2.09%) | 0.459173732031 | 0.977018966705 | Abl1  Plxnb2  Pard3  Sema4g | web link | no local link |
| Biosynthesis of unsaturated fatty acids | KEGG PATHWAY | mmu01040 | 1 (0.60%) | 32 (0.37%) | 0.464344487405 | 0.977018966705 | Acox1 | web link | no local link |
| Apoptosis - multiple species | KEGG PATHWAY | mmu04215 | 1 (0.60%) | 32 (0.37%) | 0.464344487405 | 0.977018966705 | Mapk9 | web link | no local link |
| Relaxin signaling pathway | KEGG PATHWAY | mmu04926 | 3 (1.81%) | 131 (1.52%) | 0.465211686692 | 0.977018966705 | Arrb2  Mapk9  Vegfa | web link | no local link |
| Ras signaling pathway | KEGG PATHWAY | mmu04014 | 5 (3.01%) | 233 (2.71%) | 0.468773539526 | 0.977018966705 | Abl1  Rassf5  Mapk9  Vegfa  Ralbp1 | web link | no local link |
| Starch and sucrose metabolism | KEGG PATHWAY | mmu00500 | 1 (0.60%) | 33 (0.38%) | 0.474712819389 | 0.977018966705 | Enpp3 | web link | no local link |
| RNA degradation | KEGG PATHWAY | mmu03018 | 2 (1.20%) | 83 (0.96%) | 0.478072045757 | 0.977018966705 | Cnot2  Edc4 | web link | no local link |
| Oxidative phosphorylation | KEGG PATHWAY | mmu00190 | 3 (1.81%) | 134 (1.56%) | 0.480133759906 | 0.977018966705 | Cox7a2  Ndufa5  Ndufs6 | web link | no local link |
| Rheumatoid arthritis | KEGG PATHWAY | mmu05323 | 2 (1.20%) | 84 (0.98%) | 0.484359110201 | 0.977018966705 | Acp5  Vegfa | web link | no local link |
| ErbB signaling pathway | KEGG PATHWAY | mmu04012 | 2 (1.20%) | 84 (0.98%) | 0.484359110201 | 0.977018966705 | Abl1  Mapk9 | web link | no local link |
| beta-Alanine metabolism | KEGG PATHWAY | mmu00410 | 1 (0.60%) | 34 (0.39%) | 0.48488164411 | 0.977018966705 | Acox1 | web link | no local link |
| DNA replication | KEGG PATHWAY | mmu03030 | 1 (0.60%) | 35 (0.41%) | 0.494854777662 | 0.977018966705 | Dna2 | web link | no local link |
| Base excision repair | KEGG PATHWAY | mmu03410 | 1 (0.60%) | 35 (0.41%) | 0.494854777662 | 0.977018966705 | Parp3 | web link | no local link |
| MAPK signaling pathway | KEGG PATHWAY | mmu04010 | 6 (3.61%) | 294 (3.42%) | 0.502927323783 | 0.977018966705 | Map4k2  Arrb2  Vegfa  Mapkapk5  Mapk9  Dusp3 | web link | no local link |
| Renin-angiotensin system | KEGG PATHWAY | mmu04614 | 1 (0.60%) | 36 (0.42%) | 0.504635963584 | 0.977018966705 | Ctsa | web link | no local link |
| Colorectal cancer | KEGG PATHWAY | mmu05210 | 2 (1.20%) | 88 (1.02%) | 0.509035386309 | 0.977018966705 | Mapk9  Tcf7l2 | web link | no local link |
| Insulin signaling pathway | KEGG PATHWAY | mmu04910 | 3 (1.81%) | 140 (1.63%) | 0.509380863692 | 0.977018966705 | Mapk9  Srebf1  Acacb | web link | no local link |
| Thyroid cancer | KEGG PATHWAY | mmu05216 | 1 (0.60%) | 37 (0.43%) | 0.514228874229 | 0.977018966705 | Tcf7l2 | web link | no local link |
| Longevity regulating pathway | KEGG PATHWAY | mmu04211 | 2 (1.20%) | 90 (1.05%) | 0.521083605293 | 0.977018966705 | Ulk1  Akt1s1 | web link | no local link |
| Fluid shear stress and atherosclerosis | KEGG PATHWAY | mmu05418 | 3 (1.81%) | 143 (1.66%) | 0.523682665917 | 0.977018966705 | Mapk9  Vegfa  Itgav | web link | no local link |
| Small cell lung cancer | KEGG PATHWAY | mmu05222 | 2 (1.20%) | 92 (1.07%) | 0.532934260499 | 0.977018966705 | Fn1  Itgav | web link | no local link |
| Bladder cancer | KEGG PATHWAY | mmu05219 | 1 (0.60%) | 41 (0.48%) | 0.550788794269 | 0.977018966705 | Vegfa | web link | no local link |
| mRNA surveillance pathway | KEGG PATHWAY | mmu03015 | 2 (1.20%) | 96 (1.12%) | 0.556033114737 | 0.977018966705 | Smg7  Ppp2r5c | web link | no local link |
| Retrograde endocannabinoid signaling | KEGG PATHWAY | mmu04723 | 3 (1.81%) | 150 (1.74%) | 0.556151770641 | 0.977018966705 | Mapk9  Ndufa5  Ndufs6 | web link | no local link |
| Basal transcription factors | KEGG PATHWAY | mmu03022 | 1 (0.60%) | 44 (0.51%) | 0.576401679899 | 0.977018966705 | Gtf2a2 | web link | no local link |
| Proteasome | KEGG PATHWAY | mmu03050 | 1 (0.60%) | 46 (0.53%) | 0.592665073347 | 0.977018966705 | Psmc6 | web link | no local link |
| Amoebiasis | KEGG PATHWAY | mmu05146 | 2 (1.20%) | 103 (1.20%) | 0.594489970468 | 0.977018966705 | Serpinb6a  Fn1 | web link | no local link |
| Hepatitis C | KEGG PATHWAY | mmu05160 | 3 (1.81%) | 160 (1.86%) | 0.600189169834 | 0.977018966705 | Ywhaz  Eif3e  Nr1h3 | web link | no local link |
| Mineral absorption | KEGG PATHWAY | mmu04978 | 1 (0.60%) | 47 (0.55%) | 0.600562480241 | 0.977018966705 | Clcn2 | web link | no local link |
| Glucagon signaling pathway | KEGG PATHWAY | mmu04922 | 2 (1.20%) | 105 (1.22%) | 0.605013907833 | 0.977018966705 | Ppp4r3a  Acacb | web link | no local link |
| Type II diabetes mellitus | KEGG PATHWAY | mmu04930 | 1 (0.60%) | 48 (0.56%) | 0.608307676862 | 0.977018966705 | Mapk9 | web link | no local link |
| Cocaine addiction | KEGG PATHWAY | mmu05030 | 1 (0.60%) | 48 (0.56%) | 0.608307676862 | 0.977018966705 | Maob | web link | no local link |
| Amino sugar and nucleotide sugar metabolism | KEGG PATHWAY | mmu00520 | 1 (0.60%) | 49 (0.57%) | 0.615903579391 | 0.977018966705 | Gmppa | web link | no local link |
| N-Glycan biosynthesis | KEGG PATHWAY | mmu00510 | 1 (0.60%) | 50 (0.58%) | 0.623353048467 | 0.977018966705 | Mgat1 | web link | no local link |
| Fatty acid degradation | KEGG PATHWAY | mmu00071 | 1 (0.60%) | 50 (0.58%) | 0.623353048467 | 0.977018966705 | Acox1 | web link | no local link |
| Fanconi anemia pathway | KEGG PATHWAY | mmu03460 | 1 (0.60%) | 51 (0.59%) | 0.630658890247 | 0.977018966705 | Cenpx | web link | no local link |
| Amyotrophic lateral sclerosis (ALS) | KEGG PATHWAY | mmu05014 | 1 (0.60%) | 52 (0.60%) | 0.637823857434 | 0.977018966705 | Derl1 | web link | no local link |
| Viral carcinogenesis | KEGG PATHWAY | mmu05203 | 4 (2.41%) | 231 (2.68%) | 0.655803156683 | 0.977018966705 | Ywhaz  Hdac10  Hnrnpk  Gtf2a2 | web link | no local link |
| Ribosome biogenesis in eukaryotes | KEGG PATHWAY | mmu03008 | 2 (1.20%) | 116 (1.35%) | 0.659220246722 | 0.977018966705 | Sbds  Rexo5 | web link | no local link |
| Alzheimer disease | KEGG PATHWAY | mmu05010 | 3 (1.81%) | 175 (2.03%) | 0.660712427186 | 0.977018966705 | Cox7a2  Ndufa5  Ndufs6 | web link | no local link |
| Regulation of lipolysis in adipocytes | KEGG PATHWAY | mmu04923 | 1 (0.60%) | 56 (0.65%) | 0.66512821957 | 0.977018966705 | Pnpla2 | web link | no local link |
| Necroptosis | KEGG PATHWAY | mmu04217 | 3 (1.81%) | 177 (2.06%) | 0.668266149839 | 0.977018966705 | Chmp3  Mapk9  Parp3 | web link | no local link |
| Thyroid hormone signaling pathway | KEGG PATHWAY | mmu04919 | 2 (1.20%) | 118 (1.37%) | 0.668414947228 | 0.977018966705 | Pfkfb2  Itgav | web link | no local link |
| Ovarian steroidogenesis | KEGG PATHWAY | mmu04913 | 1 (0.60%) | 57 (0.66%) | 0.67162830324 | 0.977018966705 | Hsd17b2 | web link | no local link |
| Endometrial cancer | KEGG PATHWAY | mmu05213 | 1 (0.60%) | 58 (0.67%) | 0.678002961369 | 0.977018966705 | Tcf7l2 | web link | no local link |
| VEGF signaling pathway | KEGG PATHWAY | mmu04370 | 1 (0.60%) | 58 (0.67%) | 0.678002961369 | 0.977018966705 | Vegfa | web link | no local link |
| Neurotrophin signaling pathway | KEGG PATHWAY | mmu04722 | 2 (1.20%) | 121 (1.41%) | 0.681832582792 | 0.977018966705 | Abl1  Mapk9 | web link | no local link |
| Yersinia infection | KEGG PATHWAY | mmu05135 | 2 (1.20%) | 123 (1.43%) | 0.690530689364 | 0.977018966705 | Mapk9  Fn1 | web link | no local link |
| Fatty acid metabolism | KEGG PATHWAY | mmu01212 | 1 (0.60%) | 61 (0.71%) | 0.696398212255 | 0.977018966705 | Acox1 | web link | no local link |
| Human papillomavirus infection | KEGG PATHWAY | mmu05165 | 6 (3.61%) | 361 (4.19%) | 0.702252775466 | 0.977018966705 | Pard3  Fn1  Tcf7l2  Vegfa  Itgav  Ppp2r5c | web link | no local link |
| Longevity regulating pathway - multiple species | KEGG PATHWAY | mmu04213 | 1 (0.60%) | 62 (0.72%) | 0.702294772074 | 0.977018966705 | Akt1s1 | web link | no local link |
| Inflammatory mediator regulation of TRP channels | KEGG PATHWAY | mmu04750 | 2 (1.20%) | 127 (1.48%) | 0.707343173367 | 0.977018966705 | P2ry2  Mapk9 | web link | no local link |
| Basal cell carcinoma | KEGG PATHWAY | mmu05217 | 1 (0.60%) | 63 (0.73%) | 0.708077485371 | 0.977018966705 | Tcf7l2 | web link | no local link |
| Osteoclast differentiation | KEGG PATHWAY | mmu04380 | 2 (1.20%) | 128 (1.49%) | 0.711426269109 | 0.977018966705 | Acp5  Mapk9 | web link | no local link |
| Glutathione metabolism | KEGG PATHWAY | mmu00480 | 1 (0.60%) | 64 (0.74%) | 0.713748537147 | 0.977018966705 | Ggt6 | web link | no local link |
| Huntington disease | KEGG PATHWAY | mmu05016 | 3 (1.81%) | 193 (2.24%) | 0.724310828428 | 0.977018966705 | Cox7a2  Ndufa5  Ndufs6 | web link | no local link |
| Aminoacyl-tRNA biosynthesis | KEGG PATHWAY | mmu00970 | 1 (0.60%) | 66 (0.77%) | 0.724764188506 | 0.977018966705 | Farsa | web link | no local link |
| Non-small cell lung cancer | KEGG PATHWAY | mmu05223 | 1 (0.60%) | 66 (0.77%) | 0.724764188506 | 0.977018966705 | Rassf5 | web link | no local link |
| Serotonergic synapse | KEGG PATHWAY | mmu04726 | 2 (1.20%) | 132 (1.53%) | 0.72728729695 | 0.977018966705 | Maob  Cyp2d10 | web link | no local link |
| FoxO signaling pathway | KEGG PATHWAY | mmu04068 | 2 (1.20%) | 132 (1.53%) | 0.72728729695 | 0.977018966705 | Mapk9  Sgk1 | web link | no local link |
| Amphetamine addiction | KEGG PATHWAY | mmu05031 | 1 (0.60%) | 68 (0.79%) | 0.735358386583 | 0.977018966705 | Maob | web link | no local link |
| RIG-I-like receptor signaling pathway | KEGG PATHWAY | mmu04622 | 1 (0.60%) | 68 (0.79%) | 0.735358386583 | 0.977018966705 | Mapk9 | web link | no local link |
| Renal cell carcinoma | KEGG PATHWAY | mmu05211 | 1 (0.60%) | 68 (0.79%) | 0.735358386583 | 0.977018966705 | Vegfa | web link | no local link |
| Fc epsilon RI signaling pathway | KEGG PATHWAY | mmu04664 | 1 (0.60%) | 68 (0.79%) | 0.735358386583 | 0.977018966705 | Mapk9 | web link | no local link |
| Acute myeloid leukemia | KEGG PATHWAY | mmu05221 | 1 (0.60%) | 69 (0.80%) | 0.740502474151 | 0.977018966705 | Tcf7l2 | web link | no local link |
| Apoptosis | KEGG PATHWAY | mmu04210 | 2 (1.20%) | 136 (1.58%) | 0.742409103716 | 0.977018966705 | Mapk9  Parp3 | web link | no local link |
| Signaling pathways regulating pluripotency of stem cells | KEGG PATHWAY | mmu04550 | 2 (1.20%) | 138 (1.60%) | 0.749699308427 | 0.977018966705 | Tbx3  Lifr | web link | no local link |
| NOD-like receptor signaling pathway | KEGG PATHWAY | mmu04621 | 3 (1.81%) | 206 (2.39%) | 0.764225103612 | 0.977018966705 | Erbin  Mapk9  Atg16l1 | web link | no local link |
| Proteoglycans in cancer | KEGG PATHWAY | mmu05205 | 3 (1.81%) | 206 (2.39%) | 0.764225103612 | 0.977018966705 | Vegfa  Fn1  Itgav | web link | no local link |
| Gastric acid secretion | KEGG PATHWAY | mmu04971 | 1 (0.60%) | 74 (0.86%) | 0.764769558978 | 0.977018966705 | Slc4a2 | web link | no local link |
| Pertussis | KEGG PATHWAY | mmu05133 | 1 (0.60%) | 76 (0.88%) | 0.773832301226 | 0.977018966705 | Mapk9 | web link | no local link |
| Chronic myeloid leukemia | KEGG PATHWAY | mmu05220 | 1 (0.60%) | 76 (0.88%) | 0.773832301226 | 0.977018966705 | Abl1 | web link | no local link |
| Salivary secretion | KEGG PATHWAY | mmu04970 | 1 (0.60%) | 78 (0.91%) | 0.782547905086 | 0.977018966705 | Slc4a2 | web link | no local link |
| Rap1 signaling pathway | KEGG PATHWAY | mmu04015 | 3 (1.81%) | 214 (2.49%) | 0.786390292062 | 0.977018966705 | Rassf5  Pard3  Vegfa | web link | no local link |
| EGFR tyrosine kinase inhibitor resistance | KEGG PATHWAY | mmu01521 | 1 (0.60%) | 80 (0.93%) | 0.790929589803 | 0.977018966705 | Vegfa | web link | no local link |
| Regulation of actin cytoskeleton | KEGG PATHWAY | mmu04810 | 3 (1.81%) | 217 (2.52%) | 0.794249536435 | 0.977018966705 | Arpc5  Fn1  Itgav | web link | no local link |
| Adrenergic signaling in cardiomyocytes | KEGG PATHWAY | mmu04261 | 2 (1.20%) | 152 (1.77%) | 0.795937227184 | 0.977018966705 | Crem  Ppp2r5c | web link | no local link |
| Peroxisome | KEGG PATHWAY | mmu04146 | 1 (0.60%) | 84 (0.98%) | 0.806741595398 | 0.977018966705 | Acox1 | web link | no local link |
| Cardiac muscle contraction | KEGG PATHWAY | mmu04260 | 1 (0.60%) | 86 (1.00%) | 0.814195927176 | 0.977018966705 | Cox7a2 | web link | no local link |
| Th1 and Th2 cell differentiation | KEGG PATHWAY | mmu04658 | 1 (0.60%) | 87 (1.01%) | 0.817815203882 | 0.977018966705 | Mapk9 | web link | no local link |
| Pathways in cancer | KEGG PATHWAY | mmu05200 | 8 (4.82%) | 535 (6.22%) | 0.818243424215 | 0.977018966705 | Ralbp1  Fn1  Rassf5  Tcf7l2  Vegfa  Itgav  Abl1  Mapk9 | web link | no local link |
| Viral myocarditis | KEGG PATHWAY | mmu05416 | 1 (0.60%) | 89 (1.03%) | 0.824844856115 | 0.977018966705 | Abl1 | web link | no local link |
| Hepatitis B | KEGG PATHWAY | mmu05161 | 2 (1.20%) | 163 (1.89%) | 0.826831873126 | 0.977018966705 | Ywhaz  Mapk9 | web link | no local link |
| GABAergic synapse | KEGG PATHWAY | mmu04727 | 1 (0.60%) | 90 (1.05%) | 0.828257903877 | 0.977018966705 | Slc38a3 | web link | no local link |
| GnRH signaling pathway | KEGG PATHWAY | mmu04912 | 1 (0.60%) | 90 (1.05%) | 0.828257903877 | 0.977018966705 | Mapk9 | web link | no local link |
| Antigen processing and presentation | KEGG PATHWAY | mmu04612 | 1 (0.60%) | 91 (1.06%) | 0.831604838364 | 0.977018966705 | Rfxap | web link | no local link |
| Hypertrophic cardiomyopathy (HCM) | KEGG PATHWAY | mmu05410 | 1 (0.60%) | 91 (1.06%) | 0.831604838364 | 0.977018966705 | Itgav | web link | no local link |
| Fc gamma R-mediated phagocytosis | KEGG PATHWAY | mmu04666 | 1 (0.60%) | 92 (1.07%) | 0.834886932626 | 0.977018966705 | Arpc5 | web link | no local link |
| Morphine addiction | KEGG PATHWAY | mmu05032 | 1 (0.60%) | 92 (1.07%) | 0.834886932626 | 0.977018966705 | Arrb2 | web link | no local link |
| Endocrine resistance | KEGG PATHWAY | mmu01522 | 1 (0.60%) | 93 (1.08%) | 0.838105435349 | 0.977018966705 | Mapk9 | web link | no local link |
| Dilated cardiomyopathy (DCM) | KEGG PATHWAY | mmu05414 | 1 (0.60%) | 95 (1.10%) | 0.844356541851 | 0.977018966705 | Itgav | web link | no local link |
| Hepatocellular carcinoma | KEGG PATHWAY | mmu05225 | 2 (1.20%) | 171 (1.99%) | 0.846604891382 | 0.977018966705 | Smarca2  Tcf7l2 | web link | no local link |
| Prostate cancer | KEGG PATHWAY | mmu05215 | 1 (0.60%) | 97 (1.13%) | 0.850367677409 | 0.977018966705 | Tcf7l2 | web link | no local link |
| Choline metabolism in cancer | KEGG PATHWAY | mmu05231 | 1 (0.60%) | 99 (1.15%) | 0.856148000465 | 0.977018966705 | Mapk9 | web link | no local link |
| Toll-like receptor signaling pathway | KEGG PATHWAY | mmu04620 | 1 (0.60%) | 99 (1.15%) | 0.856148000465 | 0.977018966705 | Mapk9 | web link | no local link |
| Human T-cell leukemia virus 1 infection | KEGG PATHWAY | mmu05166 | 3 (1.81%) | 246 (2.86%) | 0.858508669861 | 0.977018966705 | Mapk9  Anapc5  Anapc10 | web link | no local link |
| Melanogenesis | KEGG PATHWAY | mmu04916 | 1 (0.60%) | 100 (1.16%) | 0.858954373943 | 0.977018966705 | Tcf7l2 | web link | no local link |
| Tuberculosis | KEGG PATHWAY | mmu05152 | 2 (1.20%) | 178 (2.07%) | 0.862209383769 | 0.977018966705 | Mapk9  Rfxap | web link | no local link |
| Th17 cell differentiation | KEGG PATHWAY | mmu04659 | 1 (0.60%) | 102 (1.18%) | 0.864404893812 | 0.977018966705 | Mapk9 | web link | no local link |
| Chagas disease (American trypanosomiasis) | KEGG PATHWAY | mmu05142 | 1 (0.60%) | 103 (1.20%) | 0.867051118551 | 0.977018966705 | Mapk9 | web link | no local link |
| T cell receptor signaling pathway | KEGG PATHWAY | mmu04660 | 1 (0.60%) | 103 (1.20%) | 0.867051118551 | 0.977018966705 | Mapk9 | web link | no local link |
| Pancreatic secretion | KEGG PATHWAY | mmu04972 | 1 (0.60%) | 105 (1.22%) | 0.872190544998 | 0.977018966705 | Slc4a2 | web link | no local link |
| Parathyroid hormone synthesis, secretion and action | KEGG PATHWAY | mmu04928 | 1 (0.60%) | 107 (1.24%) | 0.877132446535 | 0.977018966705 | Arrb2 | web link | no local link |
| Cellular senescence | KEGG PATHWAY | mmu04218 | 2 (1.20%) | 186 (2.16%) | 0.878265100361 | 0.977018966705 | Btrc  Rassf5 | web link | no local link |
| Toxoplasmosis | KEGG PATHWAY | mmu05145 | 1 (0.60%) | 108 (1.25%) | 0.879531695315 | 0.977018966705 | Mapk9 | web link | no local link |
| C-type lectin receptor signaling pathway | KEGG PATHWAY | mmu04625 | 1 (0.60%) | 112 (1.30%) | 0.888671855076 | 0.977018966705 | Mapk9 | web link | no local link |
| TNF signaling pathway | KEGG PATHWAY | mmu04668 | 1 (0.60%) | 113 (1.31%) | 0.890847051882 | 0.977018966705 | Mapk9 | web link | no local link |
| HIF-1 signaling pathway | KEGG PATHWAY | mmu04066 | 1 (0.60%) | 114 (1.32%) | 0.892979999426 | 0.977018966705 | Vegfa | web link | no local link |
| Glutamatergic synapse | KEGG PATHWAY | mmu04724 | 1 (0.60%) | 114 (1.32%) | 0.892979999426 | 0.977018966705 | Slc38a3 | web link | no local link |
| Leukocyte transendothelial migration | KEGG PATHWAY | mmu04670 | 1 (0.60%) | 115 (1.34%) | 0.895071513447 | 0.977018966705 | Rassf5 | web link | no local link |
| Chemokine signaling pathway | KEGG PATHWAY | mmu04062 | 2 (1.20%) | 198 (2.30%) | 0.899151922245 | 0.977018966705 | Pard3  Arrb2 | web link | no local link |
| Alcoholism | KEGG PATHWAY | mmu05034 | 2 (1.20%) | 199 (2.31%) | 0.900733715368 | 0.977018966705 | Maob  Hdac10 | web link | no local link |
| Carbon metabolism | KEGG PATHWAY | mmu01200 | 1 (0.60%) | 121 (1.41%) | 0.906794239439 | 0.977018966705 | Acox1 | web link | no local link |
| Neuroactive ligand-receptor interaction | KEGG PATHWAY | mmu04080 | 4 (2.41%) | 348 (4.04%) | 0.908652643999 | 0.977018966705 | Prlr  P2ry2  Pard3  Ghr | web link | no local link |
| MicroRNAs in cancer | KEGG PATHWAY | mmu05206 | 3 (1.81%) | 282 (3.28%) | 0.913460099288 | 0.977018966705 | Abl1  Hnrnpk  Vegfa | web link | no local link |
| cAMP signaling pathway | KEGG PATHWAY | mmu04024 | 2 (1.20%) | 213 (2.47%) | 0.920591548909 | 0.977018966705 | Acox1  Mapk9 | web link | no local link |
| Kaposi sarcoma-associated herpesvirus infection | KEGG PATHWAY | mmu05167 | 2 (1.20%) | 217 (2.52%) | 0.92554330712 | 0.977018966705 | Mapk9  Vegfa | web link | no local link |
| Cytokine-cytokine receptor interaction | KEGG PATHWAY | mmu04060 | 3 (1.81%) | 293 (3.40%) | 0.925916024542 | 0.977018966705 | Prlr  Lifr  Ghr | web link | no local link |
| Vascular smooth muscle contraction | KEGG PATHWAY | mmu04270 | 1 (0.60%) | 140 (1.63%) | 0.935986288354 | 0.977018966705 | Myl6 | web link | no local link |
| Epstein-Barr virus infection | KEGG PATHWAY | mmu05169 | 2 (1.20%) | 230 (2.67%) | 0.939708472223 | 0.977018966705 | Mapk9  Psmc6 | web link | no local link |
| Measles | KEGG PATHWAY | mmu05162 | 1 (0.60%) | 144 (1.67%) | 0.940860945975 | 0.977018966705 | Mapk9 | web link | no local link |
| Breast cancer | KEGG PATHWAY | mmu05224 | 1 (0.60%) | 147 (1.71%) | 0.944273133854 | 0.977018966705 | Tcf7l2 | web link | no local link |
| Gastric cancer | KEGG PATHWAY | mmu05226 | 1 (0.60%) | 150 (1.74%) | 0.947489563976 | 0.977018966705 | Tcf7l2 | web link | no local link |
| Human immunodeficiency virus 1 infection | KEGG PATHWAY | mmu05170 | 2 (1.20%) | 239 (2.78%) | 0.947981776446 | 0.977018966705 | Mapk9  Btrc | web link | no local link |
| Oxytocin signaling pathway | KEGG PATHWAY | mmu04921 | 1 (0.60%) | 154 (1.79%) | 0.951492833595 | 0.977018966705 | Myl6 | web link | no local link |
| Cushing syndrome | KEGG PATHWAY | mmu04934 | 1 (0.60%) | 159 (1.85%) | 0.956072841888 | 0.977018966705 | Tcf7l2 | web link | no local link |
| Influenza A | KEGG PATHWAY | mmu05164 | 1 (0.60%) | 164 (1.91%) | 0.960222767999 | 0.977018966705 | Nup98 | web link | no local link |
| Human cytomegalovirus infection | KEGG PATHWAY | mmu05163 | 2 (1.20%) | 256 (2.97%) | 0.960760349971 | 0.977018966705 | Vegfa  Itgav | web link | no local link |
| Cell adhesion molecules (CAMs) | KEGG PATHWAY | mmu04514 | 1 (0.60%) | 171 (1.99%) | 0.965385881977 | 0.977018966705 | Itgav | web link | no local link |
| Herpes simplex virus 1 infection | KEGG PATHWAY | mmu05168 | 4 (2.41%) | 435 (5.05%) | 0.971597869457 | 0.977018966705 | Zfp58  Zfp235  Zfp938  Zfp874b | web link | no local link |
| Phagosome | KEGG PATHWAY | mmu04145 | 1 (0.60%) | 181 (2.10%) | 0.971626921336 | 0.977018966705 | Itgav | web link | no local link |
| Transcriptional misregulation in cancer | KEGG PATHWAY | mmu05202 | 1 (0.60%) | 183 (2.13%) | 0.972733795798 | 0.977018966705 | Ss18 | web link | no local link |
| Olfactory transduction | KEGG PATHWAY | mmu04740 | 1 (0.60%) | 1134 (13.17%) | 0.999999999949 | 0.999999999949 | Arrb2 | web link | no local link |
