# Supplementary material for: Regulation of Alternative Splicing of Lipid Metabolism Genes in Sepsis-Induced Liver Damage by RNA-Binding Proteins
Source: Inflammation. 2024 May 9;47(6):1952–68. doi: 10.1007/s10753-024-02017-2 (PMC11607010; doi:10.1007/s10753-024-02017-2)
Supplement: Supplementary file 1 — Supplementary file1 (ZIP 38655 KB) [file 10753_2024_2017_MOESM1_ESM.zip › Sup6_AS_KEGG.zip!/Sup6_AS_KEGG/ras/di_RAS/GO_KEGG/CLP_vs_Sham_and_CLPDCA_vs_Sham_di_RAS/CLP_vs_Sham_diDown_nir_KEGG_analysis/CLP_vs_Sham_diDown_nir_KEGG_top.pdf]

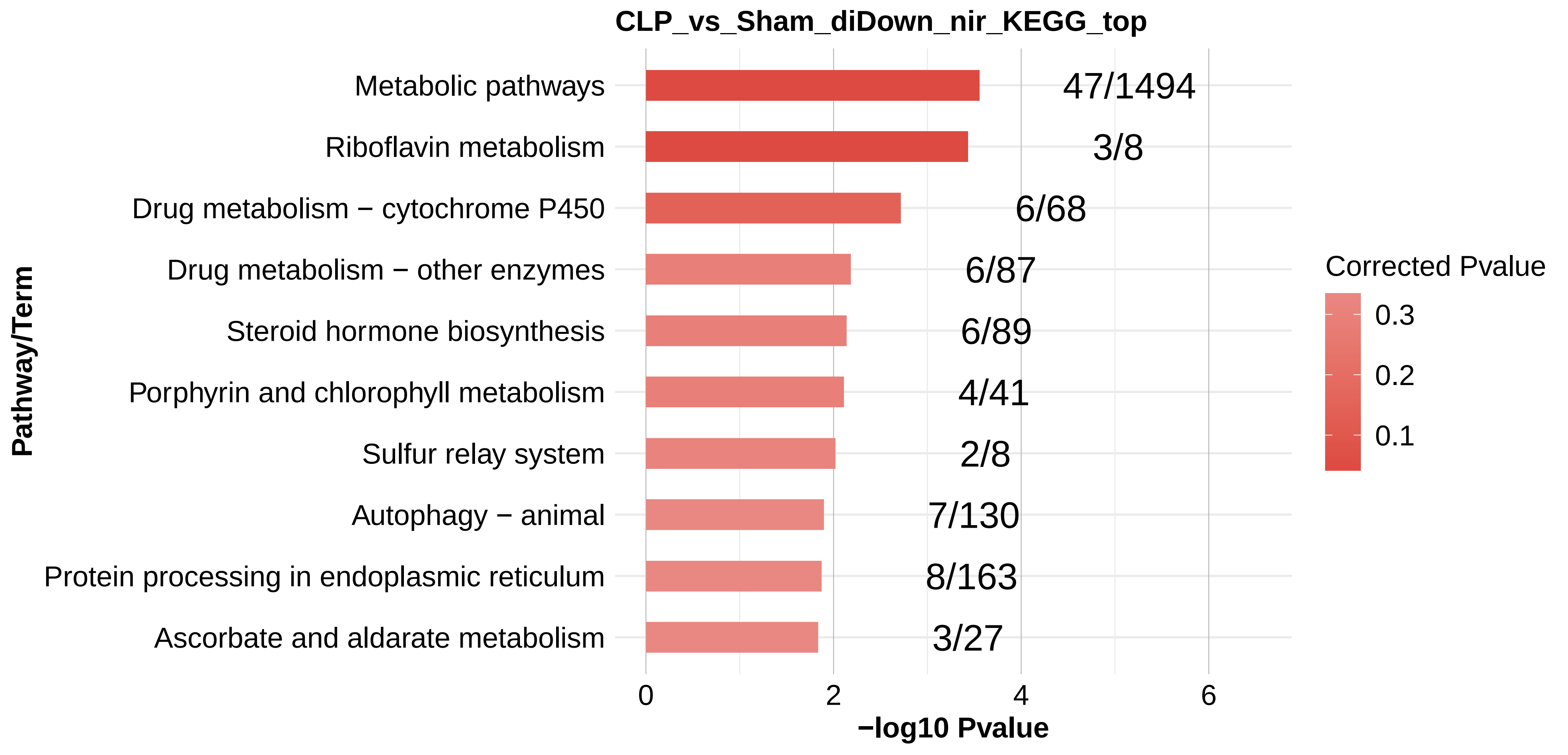
CLP_vs_Sham_diDown_nir_KEGG_top
Pathway/Term
Metabolic pathways
Riboflavin metabolism
Drug metabolism − cytochrome P450
Drug metabolism − other enzymes
Steroid hormone biosynthesis
Porphyrin and chlorophyll metabolism
Sulfur relay system
Autophagy − animal
Protein processing in endoplasmic reticulum
Ascorbate and aldarate metabolism
47/1494
3/8
6/68
6/87
6/89
4/41
2/8
7/130
8/163
3/27
0
2
4
6
−log10 Pvalue
Corrected Pvalue
0.3
0.2
0.1
