# Supplementary material for: Regulation of Alternative Splicing of Lipid Metabolism Genes in Sepsis-Induced Liver Damage by RNA-Binding Proteins
Source: Inflammation. 2024 May 9;47(6):1952–68. doi: 10.1007/s10753-024-02017-2 (PMC11607010; doi:10.1007/s10753-024-02017-2)
Supplement: Supplementary file 1 — Supplementary file1 (ZIP 38655 KB) [file 10753_2024_2017_MOESM1_ESM.zip › Sup6_AS_KEGG.zip!/Sup6_AS_KEGG/ras/co_RAS/GO_KEGG/CLP_vs_Sham_and_CLPDCA_vs_Sham_co_RAS/coUp_nir_KEGG_analysis/coUp_nir_KEGG_pathway.html]

KEGG pathway enrichment analyze result


# KEGG pathway enrichment analyze result


| Term | Database | ID | Input number (28) | Background number (8608) | P-Value | Corrected P-Value | Input | Hyperlink | img |
| --- | --- | --- | --- | --- | --- | --- | --- | --- | --- |
| Metabolic pathways | KEGG PATHWAY | mmu01100 | 11 (39.29%) | 1494 (17.36%) | 0.00497910628159 | 0.314948007952 | Upp2  St3gal5  St3gal4  Idh1  Pcyt2  Comt  Agxt2  Hoga1  Cyp4a31  Mtmr4  Ndufs2 | web link | no local link |
| Basal transcription factors | KEGG PATHWAY | mmu03022 | 2 (7.14%) | 44 (0.51%) | 0.00887177487189 | 0.314948007952 | Gtf2i  Gtf2a2 | web link | no local link |
| Phosphonate and phosphinate metabolism | KEGG PATHWAY | mmu00440 | 1 (3.57%) | 6 (0.07%) | 0.0193642847917 | 0.458288073404 | Pcyt2 | web link | no local link |
| Glycerophospholipid metabolism | KEGG PATHWAY | mmu00564 | 2 (7.14%) | 97 (1.13%) | 0.039288819628 | 0.49049338757 | Pcyt2  Taz | web link | no local link |
| Glycosphingolipid biosynthesis - ganglio series | KEGG PATHWAY | mmu00604 | 1 (3.57%) | 15 (0.17%) | 0.0477343133133 | 0.49049338757 | St3gal5 | web link | no local link |
| C-type lectin receptor signaling pathway | KEGG PATHWAY | mmu04625 | 2 (7.14%) | 112 (1.30%) | 0.0509175640534 | 0.49049338757 | Arhgef12  Mapkapk2 | web link | no local link |
| 2-Oxocarboxylic acid metabolism | KEGG PATHWAY | mmu01210 | 1 (3.57%) | 19 (0.22%) | 0.0600876285755 | 0.49049338757 | Idh1 | web link | no local link |
| Ubiquitin mediated proteolysis | KEGG PATHWAY | mmu04120 | 2 (7.14%) | 139 (1.61%) | 0.0744893918281 | 0.49049338757 | Ube2i  Ube2d3 | web link | no local link |
| Vascular smooth muscle contraction | KEGG PATHWAY | mmu04270 | 2 (7.14%) | 140 (1.63%) | 0.0754204609412 | 0.49049338757 | Cyp4a31  Arhgef12 | web link | no local link |
| Glycosphingolipid biosynthesis - lacto and neolacto series | KEGG PATHWAY | mmu00601 | 1 (3.57%) | 27 (0.31%) | 0.0843323160828 | 0.49049338757 | St3gal4 | web link | no local link |
| Glyoxylate and dicarboxylate metabolism | KEGG PATHWAY | mmu00630 | 1 (3.57%) | 31 (0.36%) | 0.0962273948269 | 0.49049338757 | Hoga1 | web link | no local link |
| Protein processing in endoplasmic reticulum | KEGG PATHWAY | mmu04141 | 2 (7.14%) | 163 (1.89%) | 0.0978248308573 | 0.49049338757 | Dnajb12  Ube2d3 | web link | no local link |
| Citrate cycle (TCA cycle) | KEGG PATHWAY | mmu00020 | 1 (3.57%) | 32 (0.37%) | 0.0991778009065 | 0.49049338757 | Idh1 | web link | no local link |
| Base excision repair | KEGG PATHWAY | mmu03410 | 1 (3.57%) | 35 (0.41%) | 0.10797339877 | 0.49049338757 | Apex1 | web link | no local link |
| Alanine, aspartate and glutamate metabolism | KEGG PATHWAY | mmu00250 | 1 (3.57%) | 38 (0.44%) | 0.11668615494 | 0.49049338757 | Agxt2 | web link | no local link |
| Tyrosine metabolism | KEGG PATHWAY | mmu00350 | 1 (3.57%) | 40 (0.46%) | 0.122449007328 | 0.49049338757 | Comt | web link | no local link |
| Glycine, serine and threonine metabolism | KEGG PATHWAY | mmu00260 | 1 (3.57%) | 40 (0.46%) | 0.122449007328 | 0.49049338757 | Agxt2 | web link | no local link |
| Ether lipid metabolism | KEGG PATHWAY | mmu00565 | 1 (3.57%) | 47 (0.55%) | 0.142334856672 | 0.49049338757 | Enpp2 | web link | no local link |
| Arginine and proline metabolism | KEGG PATHWAY | mmu00330 | 1 (3.57%) | 50 (0.58%) | 0.150723696872 | 0.49049338757 | Hoga1 | web link | no local link |
| Fatty acid degradation | KEGG PATHWAY | mmu00071 | 1 (3.57%) | 50 (0.58%) | 0.150723696872 | 0.49049338757 | Cyp4a31 | web link | no local link |
| Cysteine and methionine metabolism | KEGG PATHWAY | mmu00270 | 1 (3.57%) | 52 (0.60%) | 0.156272240983 | 0.49049338757 | Agxt2 | web link | no local link |
| Valine, leucine and isoleucine degradation | KEGG PATHWAY | mmu00280 | 1 (3.57%) | 56 (0.65%) | 0.1672646506 | 0.49049338757 | Agxt2 | web link | no local link |
| Viral carcinogenesis | KEGG PATHWAY | mmu05203 | 2 (7.14%) | 231 (2.68%) | 0.172455639998 | 0.49049338757 | Mapkapk2  Gtf2a2 | web link | no local link |
| Pyrimidine metabolism | KEGG PATHWAY | mmu00240 | 1 (3.57%) | 58 (0.67%) | 0.172708939285 | 0.49049338757 | Upp2 | web link | no local link |
| VEGF signaling pathway | KEGG PATHWAY | mmu04370 | 1 (3.57%) | 58 (0.67%) | 0.172708939285 | 0.49049338757 | Mapkapk2 | web link | no local link |
| Glutathione metabolism | KEGG PATHWAY | mmu00480 | 1 (3.57%) | 64 (0.74%) | 0.188836654063 | 0.494573534402 | Idh1 | web link | no local link |
| Inositol phosphate metabolism | KEGG PATHWAY | mmu00562 | 1 (3.57%) | 73 (0.85%) | 0.212461099212 | 0.494573534402 | Mtmr4 | web link | no local link |
| Pertussis | KEGG PATHWAY | mmu05133 | 1 (3.57%) | 76 (0.88%) | 0.220187424171 | 0.494573534402 | C4bp | web link | no local link |
| Biosynthesis of amino acids | KEGG PATHWAY | mmu01230 | 1 (3.57%) | 78 (0.91%) | 0.22529764438 | 0.494573534402 | Idh1 | web link | no local link |
| RNA degradation | KEGG PATHWAY | mmu03018 | 1 (3.57%) | 83 (0.96%) | 0.237932314216 | 0.494573534402 | Cnot10 | web link | no local link |
| Peroxisome | KEGG PATHWAY | mmu04146 | 1 (3.57%) | 84 (0.98%) | 0.240435293125 | 0.494573534402 | Idh1 | web link | no local link |
| Drug metabolism - other enzymes | KEGG PATHWAY | mmu00983 | 1 (3.57%) | 87 (1.01%) | 0.247896757006 | 0.494573534402 | Upp2 | web link | no local link |
| PPAR signaling pathway | KEGG PATHWAY | mmu03320 | 1 (3.57%) | 87 (1.01%) | 0.247896757006 | 0.494573534402 | Cyp4a31 | web link | no local link |
| Complement and coagulation cascades | KEGG PATHWAY | mmu04610 | 1 (3.57%) | 88 (1.02%) | 0.250368167733 | 0.494573534402 | C4bp | web link | no local link |
| Arachidonic acid metabolism | KEGG PATHWAY | mmu00590 | 1 (3.57%) | 89 (1.03%) | 0.252831746524 | 0.494573534402 | Cyp4a31 | web link | no local link |
| Steroid hormone biosynthesis | KEGG PATHWAY | mmu00140 | 1 (3.57%) | 89 (1.03%) | 0.252831746524 | 0.494573534402 | Comt | web link | no local link |
| Retinol metabolism | KEGG PATHWAY | mmu00830 | 1 (3.57%) | 91 (1.06%) | 0.257735503843 | 0.494573534402 | Cyp4a31 | web link | no local link |
| mRNA surveillance pathway | KEGG PATHWAY | mmu03015 | 1 (3.57%) | 96 (1.12%) | 0.269859502917 | 0.500013748838 | Cpsf7 | web link | no local link |
| Phosphatidylinositol signaling system | KEGG PATHWAY | mmu04070 | 1 (3.57%) | 98 (1.14%) | 0.274655439502 | 0.500013748838 | Mtmr4 | web link | no local link |
| NF-kappa B signaling pathway | KEGG PATHWAY | mmu04064 | 1 (3.57%) | 108 (1.25%) | 0.298183051048 | 0.508488024027 | Ube2i | web link | no local link |
| Leukocyte transendothelial migration | KEGG PATHWAY | mmu04670 | 1 (3.57%) | 115 (1.34%) | 0.314212675074 | 0.508488024027 | Arhgap5 | web link | no local link |
| Carbon metabolism | KEGG PATHWAY | mmu01200 | 1 (3.57%) | 121 (1.41%) | 0.327670842785 | 0.508488024027 | Idh1 | web link | no local link |
| Neurotrophin signaling pathway | KEGG PATHWAY | mmu04722 | 1 (3.57%) | 121 (1.41%) | 0.327670842785 | 0.508488024027 | Mapkapk2 | web link | no local link |
| Yersinia infection | KEGG PATHWAY | mmu05135 | 1 (3.57%) | 123 (1.43%) | 0.332100032888 | 0.508488024027 | Arhgef12 | web link | no local link |
| Platelet activation | KEGG PATHWAY | mmu04611 | 1 (3.57%) | 125 (1.45%) | 0.336501079714 | 0.508488024027 | Arhgef12 | web link | no local link |
| Inflammatory mediator regulation of TRP channels | KEGG PATHWAY | mmu04750 | 1 (3.57%) | 127 (1.48%) | 0.340874155504 | 0.508488024027 | Cyp4a31 | web link | no local link |
| Autophagy - animal | KEGG PATHWAY | mmu04140 | 1 (3.57%) | 130 (1.51%) | 0.347381697781 | 0.508488024027 | Mtmr4 | web link | no local link |
| Spliceosome | KEGG PATHWAY | mmu03040 | 1 (3.57%) | 133 (1.55%) | 0.353827263898 | 0.508488024027 | Snrpa1 | web link | no local link |
| Oxidative phosphorylation | KEGG PATHWAY | mmu00190 | 1 (3.57%) | 134 (1.56%) | 0.355962111876 | 0.508488024027 | Ndufs2 | web link | no local link |
| Dopaminergic synapse | KEGG PATHWAY | mmu04728 | 1 (3.57%) | 135 (1.57%) | 0.358090157765 | 0.508488024027 | Comt | web link | no local link |
| Parkinson disease | KEGG PATHWAY | mmu05012 | 1 (3.57%) | 144 (1.67%) | 0.37693990055 | 0.524101440982 | Ndufs2 | web link | no local link |
| Retrograde endocannabinoid signaling | KEGG PATHWAY | mmu04723 | 1 (3.57%) | 150 (1.74%) | 0.389208643434 | 0.524101440982 | Ndufs2 | web link | no local link |
| Non-alcoholic fatty liver disease (NAFLD) | KEGG PATHWAY | mmu04932 | 1 (3.57%) | 151 (1.75%) | 0.391230653127 | 0.524101440982 | Ndufs2 | web link | no local link |
| RNA transport | KEGG PATHWAY | mmu03013 | 1 (3.57%) | 167 (1.94%) | 0.422718217312 | 0.547360775899 | Ube2i | web link | no local link |
| cGMP-PKG signaling pathway | KEGG PATHWAY | mmu04022 | 1 (3.57%) | 173 (2.01%) | 0.434116260408 | 0.547360775899 | Gtf2i | web link | no local link |
| Alzheimer disease | KEGG PATHWAY | mmu05010 | 1 (3.57%) | 175 (2.03%) | 0.437867151361 | 0.547360775899 | Ndufs2 | web link | no local link |
| Tuberculosis | KEGG PATHWAY | mmu05152 | 1 (3.57%) | 178 (2.07%) | 0.443448571359 | 0.547360775899 | Arhgef12 | web link | no local link |
| Axon guidance | KEGG PATHWAY | mmu04360 | 1 (3.57%) | 180 (2.09%) | 0.447139788762 | 0.547360775899 | Arhgef12 | web link | no local link |
| Cellular senescence | KEGG PATHWAY | mmu04218 | 1 (3.57%) | 186 (2.16%) | 0.458072346856 | 0.551239603843 | Mapkapk2 | web link | no local link |
| Huntington disease | KEGG PATHWAY | mmu05016 | 1 (3.57%) | 193 (2.24%) | 0.470563604691 | 0.556833598884 | Ndufs2 | web link | no local link |
| Focal adhesion | KEGG PATHWAY | mmu04510 | 1 (3.57%) | 199 (2.31%) | 0.481049011493 | 0.558516152016 | Arhgap5 | web link | no local link |
| Proteoglycans in cancer | KEGG PATHWAY | mmu05205 | 1 (3.57%) | 206 (2.39%) | 0.493028978832 | 0.558516152016 | Arhgef12 | web link | no local link |
| cAMP signaling pathway | KEGG PATHWAY | mmu04024 | 1 (3.57%) | 213 (2.47%) | 0.504742045562 | 0.558516152016 | Fxyd1 | web link | no local link |
| Kaposi sarcoma-associated herpesvirus infection | KEGG PATHWAY | mmu05167 | 1 (3.57%) | 217 (2.52%) | 0.511317603959 | 0.558516152016 | Mapkapk2 | web link | no local link |
| Regulation of actin cytoskeleton | KEGG PATHWAY | mmu04810 | 1 (3.57%) | 217 (2.52%) | 0.511317603959 | 0.558516152016 | Arhgef12 | web link | no local link |
| Thermogenesis | KEGG PATHWAY | mmu04714 | 1 (3.57%) | 232 (2.70%) | 0.535234252435 | 0.575782301862 | Ndufs2 | web link | no local link |
| Human cytomegalovirus infection | KEGG PATHWAY | mmu05163 | 1 (3.57%) | 256 (2.97%) | 0.571170606287 | 0.605270343976 | Arhgef12 | web link | no local link |
| MicroRNAs in cancer | KEGG PATHWAY | mmu05206 | 1 (3.57%) | 282 (3.28%) | 0.607075764958 | 0.633858519294 | Ube2i | web link | no local link |
| MAPK signaling pathway | KEGG PATHWAY | mmu04010 | 1 (3.57%) | 294 (3.42%) | 0.622652500574 | 0.640700399142 | Mapkapk2 | web link | no local link |
| Herpes simplex virus 1 infection | KEGG PATHWAY | mmu05168 | 1 (3.57%) | 435 (5.05%) | 0.766438381829 | 0.77738750157 | Zfp874a | web link | no local link |
| Pathways in cancer | KEGG PATHWAY | mmu05200 | 1 (3.57%) | 535 (6.22%) | 0.834633015517 | 0.834633015517 | Arhgef12 | web link | no local link |
