# Supplementary material for: Regulation of Alternative Splicing of Lipid Metabolism Genes in Sepsis-Induced Liver Damage by RNA-Binding Proteins
Source: Inflammation. 2024 May 9;47(6):1952–68. doi: 10.1007/s10753-024-02017-2 (PMC11607010; doi:10.1007/s10753-024-02017-2)
Supplement: Supplementary file 1 — Supplementary file1 (ZIP 38655 KB) [file 10753_2024_2017_MOESM1_ESM.zip › Sup6_AS_KEGG.zip!/Sup6_AS_KEGG/ras/co_RAS/GO_KEGG/CLP_vs_Sham_and_CLPDCA_vs_Sham_co_RAS/coUp_nir_KEGG_analysis/coUp_nir_KEGG_top.pdf]

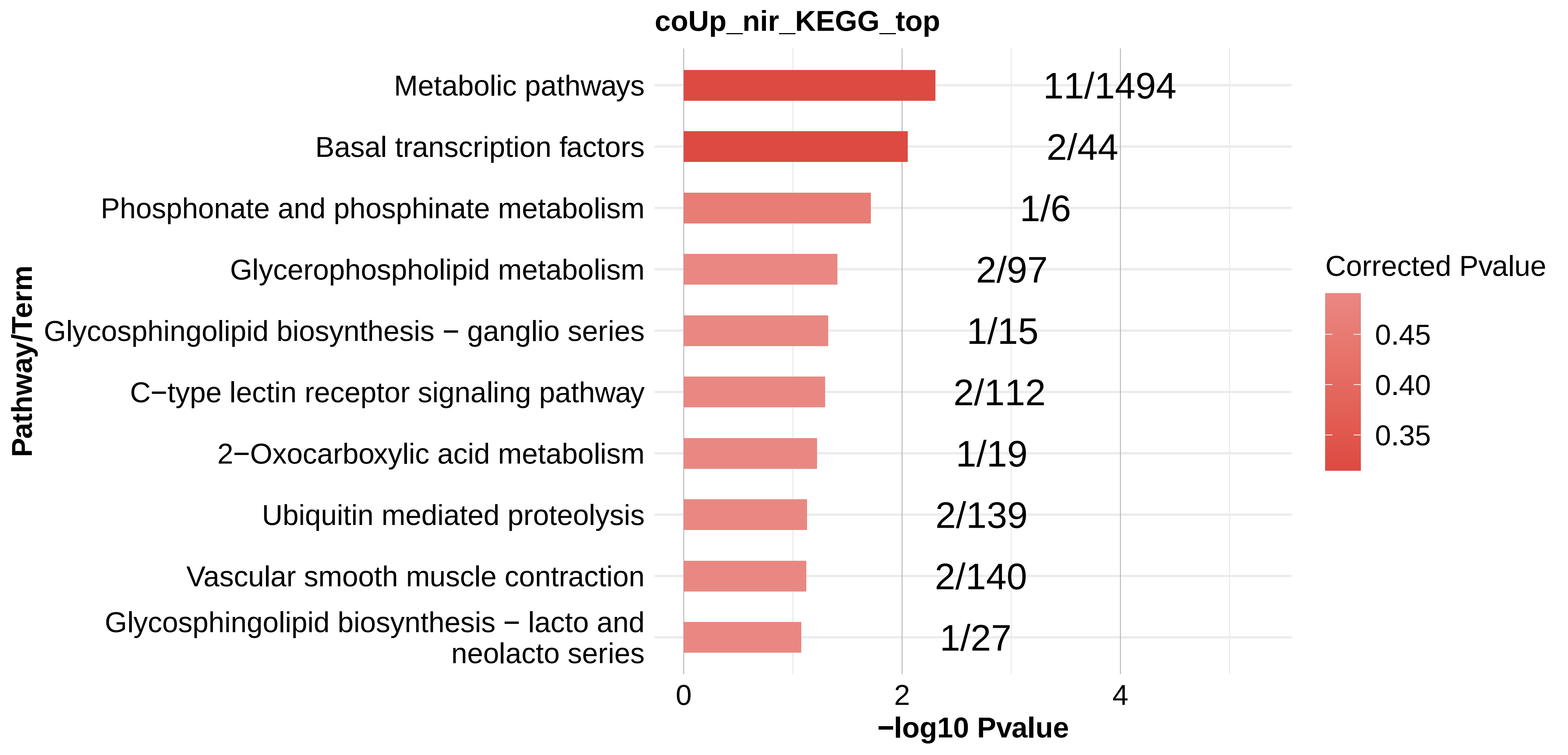

coUp_nir_KEGG_top
Metabolic pathways
Basal transcription factors
Phosphonate and phosphinate metabolism
Glycerophospholipid metabolism
Glycosphingolipid biosynthesis − ganglio series
C−type lectin receptor signaling pathway
2−Oxocarboxylic acid metabolism
Ubiquitin mediated proteolysis
Vascular smooth muscle contraction
Glycosphingolipid biosynthesis − lacto and neolacto series
11/1494
2/44
1/6
2/97
1/15
2/112
1/19
2/139
2/140
1/27
Pathway/Term
0
2
4
−log10 Pvalue
Corrected Pvalue
0.45
0.40
0.35
