# Supplementary material for: Regulation of Alternative Splicing of Lipid Metabolism Genes in Sepsis-Induced Liver Damage by RNA-Binding Proteins
Source: Inflammation. 2024 May 9;47(6):1952–68. doi: 10.1007/s10753-024-02017-2 (PMC11607010; doi:10.1007/s10753-024-02017-2)
Supplement: Supplementary file 1 — Supplementary file1 (ZIP 38655 KB) [file 10753_2024_2017_MOESM1_ESM.zip › Sup6_AS_KEGG.zip!/Sup6_AS_KEGG/ras/co_RAS/GO_KEGG/CLP_vs_Sham_and_CLPDCA_vs_Sham_co_RAS/coDown_nir_KEGG_analysis/coDown_nir_KEGG_pathway.html]

KEGG pathway enrichment analyze result


# KEGG pathway enrichment analyze result


| Term | Database | ID | Input number (44) | Background number (8608) | P-Value | Corrected P-Value | Input | Hyperlink | img |
| --- | --- | --- | --- | --- | --- | --- | --- | --- | --- |
| Insulin signaling pathway | KEGG PATHWAY | mmu04910 | 5 (11.36%) | 140 (1.63%) | 0.000690895068516 | 0.0780711427423 | Fbp1  Phkb  Prkar1a  Exoc7  Rps6kb2 | web link | no local link |
| Endocrine resistance | KEGG PATHWAY | mmu01522 | 3 (6.82%) | 93 (1.08%) | 0.0117439648445 | 0.514256559042 | Esr1  Ncor1  Rps6kb2 | web link | no local link |
| Fructose and mannose metabolism | KEGG PATHWAY | mmu00051 | 2 (4.55%) | 35 (0.41%) | 0.0136528290011 | 0.514256559042 | Gmppa  Fbp1 | web link | no local link |
| AMPK signaling pathway | KEGG PATHWAY | mmu04152 | 3 (6.82%) | 126 (1.46%) | 0.0262288313006 | 0.552335999815 | Fbp1  Ppp2r3d  Rps6kb2 | web link | no local link |
| Autophagy - animal | KEGG PATHWAY | mmu04140 | 3 (6.82%) | 130 (1.51%) | 0.0284268119766 | 0.552335999815 | Cflar  Mtmr14  Rps6kb2 | web link | no local link |
| Metabolic pathways | KEGG PATHWAY | mmu01100 | 13 (29.55%) | 1494 (17.36%) | 0.0320029738347 | 0.552335999815 | Ntpcr  Mtmr14  Pi4ka  St3gal4  Hagh  Gstm4  Gmppa  Dhrs3  Sc5d  Elovl5  Fbp1  Mthfs  Gba2 | web link | no local link |
| Inositol phosphate metabolism | KEGG PATHWAY | mmu00562 | 2 (4.55%) | 73 (0.85%) | 0.0533913329753 | 0.552335999815 | Mtmr14  Pi4ka | web link | no local link |
| Pancreatic cancer | KEGG PATHWAY | mmu05212 | 2 (4.55%) | 75 (0.87%) | 0.056020352501 | 0.552335999815 | Vegfa  Rps6kb2 | web link | no local link |
| EGFR tyrosine kinase inhibitor resistance | KEGG PATHWAY | mmu01521 | 2 (4.55%) | 80 (0.93%) | 0.0627873657293 | 0.552335999815 | Vegfa  Rps6kb2 | web link | no local link |
| Peroxisome | KEGG PATHWAY | mmu04146 | 2 (4.55%) | 84 (0.98%) | 0.0683917042289 | 0.552335999815 | Paox  Mpv17l2 | web link | no local link |
| Thiamine metabolism | KEGG PATHWAY | mmu00730 | 1 (2.27%) | 15 (0.17%) | 0.0740473981134 | 0.552335999815 | Ntpcr | web link | no local link |
| Complement and coagulation cascades | KEGG PATHWAY | mmu04610 | 2 (4.55%) | 88 (1.02%) | 0.0741560988345 | 0.552335999815 | Clu  Proc | web link | no local link |
| Fc gamma R-mediated phagocytosis | KEGG PATHWAY | mmu04666 | 2 (4.55%) | 92 (1.07%) | 0.080072086876 | 0.552335999815 | Bin1  Rps6kb2 | web link | no local link |
| TGF-beta signaling pathway | KEGG PATHWAY | mmu04350 | 2 (4.55%) | 95 (1.10%) | 0.084603619161 | 0.552335999815 | Smurf1  Rps6kb2 | web link | no local link |
| Proteoglycans in cancer | KEGG PATHWAY | mmu05205 | 3 (6.82%) | 206 (2.39%) | 0.0875789163406 | 0.552335999815 | Esr1  Vegfa  Rps6kb2 | web link | no local link |
| Other glycan degradation | KEGG PATHWAY | mmu00511 | 1 (2.27%) | 18 (0.21%) | 0.0882002018537 | 0.552335999815 | Gba2 | web link | no local link |
| Phosphatidylinositol signaling system | KEGG PATHWAY | mmu04070 | 2 (4.55%) | 98 (1.14%) | 0.0892124417014 | 0.552335999815 | Mtmr14  Pi4ka | web link | no local link |
| Steroid biosynthesis | KEGG PATHWAY | mmu00100 | 1 (2.27%) | 19 (0.22%) | 0.0928706548361 | 0.552335999815 | Sc5d | web link | no local link |
| One carbon pool by folate | KEGG PATHWAY | mmu00670 | 1 (2.27%) | 19 (0.22%) | 0.0928706548361 | 0.552335999815 | Mthfs | web link | no local link |
| Glucagon signaling pathway | KEGG PATHWAY | mmu04922 | 2 (4.55%) | 105 (1.22%) | 0.100248632168 | 0.562799768578 | Fbp1  Phkb | web link | no local link |
| Mismatch repair | KEGG PATHWAY | mmu03430 | 1 (2.27%) | 22 (0.26%) | 0.106742177609 | 0.562799768578 | Ssbp1 | web link | no local link |
| HIF-1 signaling pathway | KEGG PATHWAY | mmu04066 | 2 (4.55%) | 114 (1.32%) | 0.114966040259 | 0.562799768578 | Vegfa  Rps6kb2 | web link | no local link |
| Thyroid hormone signaling pathway | KEGG PATHWAY | mmu04919 | 2 (4.55%) | 118 (1.37%) | 0.121678682581 | 0.562799768578 | Ncor1  Esr1 | web link | no local link |
| Collecting duct acid secretion | KEGG PATHWAY | mmu04966 | 1 (2.27%) | 27 (0.31%) | 0.129402057547 | 0.562799768578 | Slc12a7 | web link | no local link |
| Glycosphingolipid biosynthesis - lacto and neolacto series | KEGG PATHWAY | mmu00601 | 1 (2.27%) | 27 (0.31%) | 0.129402057547 | 0.562799768578 | St3gal4 | web link | no local link |
| Fatty acid elongation | KEGG PATHWAY | mmu00062 | 1 (2.27%) | 29 (0.34%) | 0.138307854182 | 0.562799768578 | Elovl5 | web link | no local link |
| Pentose phosphate pathway | KEGG PATHWAY | mmu00030 | 1 (2.27%) | 32 (0.37%) | 0.151499848415 | 0.562799768578 | Fbp1 | web link | no local link |
| Biosynthesis of unsaturated fatty acids | KEGG PATHWAY | mmu01040 | 1 (2.27%) | 32 (0.37%) | 0.151499848415 | 0.562799768578 | Elovl5 | web link | no local link |
| SNARE interactions in vesicular transport | KEGG PATHWAY | mmu04130 | 1 (2.27%) | 33 (0.38%) | 0.15585316076 | 0.562799768578 | Snap23 | web link | no local link |
| DNA replication | KEGG PATHWAY | mmu03030 | 1 (2.27%) | 35 (0.41%) | 0.164494402322 | 0.562799768578 | Ssbp1 | web link | no local link |
| Fluid shear stress and atherosclerosis | KEGG PATHWAY | mmu05418 | 2 (4.55%) | 143 (1.66%) | 0.165563153764 | 0.562799768578 | Vegfa  Gstm4 | web link | no local link |
| Breast cancer | KEGG PATHWAY | mmu05224 | 2 (4.55%) | 147 (1.71%) | 0.172834785464 | 0.562799768578 | Esr1  Rps6kb2 | web link | no local link |
| Pyruvate metabolism | KEGG PATHWAY | mmu00620 | 1 (2.27%) | 38 (0.44%) | 0.177294403212 | 0.562799768578 | Hagh | web link | no local link |
| Ferroptosis | KEGG PATHWAY | mmu04216 | 1 (2.27%) | 41 (0.48%) | 0.189902697164 | 0.562799768578 | Slc40a1 | web link | no local link |
| Homologous recombination | KEGG PATHWAY | mmu03440 | 1 (2.27%) | 41 (0.48%) | 0.189902697164 | 0.562799768578 | Ssbp1 | web link | no local link |
| Bladder cancer | KEGG PATHWAY | mmu05219 | 1 (2.27%) | 41 (0.48%) | 0.189902697164 | 0.562799768578 | Vegfa | web link | no local link |
| Hedgehog signaling pathway | KEGG PATHWAY | mmu04340 | 1 (2.27%) | 44 (0.51%) | 0.202322089598 | 0.562799768578 | Smurf1 | web link | no local link |
| Mineral absorption | KEGG PATHWAY | mmu04978 | 1 (2.27%) | 47 (0.55%) | 0.214555345845 | 0.562799768578 | Slc40a1 | web link | no local link |
| Hepatocellular carcinoma | KEGG PATHWAY | mmu05225 | 2 (4.55%) | 171 (1.99%) | 0.217429124964 | 0.562799768578 | Gstm4  Rps6kb2 | web link | no local link |
| Ribosome | KEGG PATHWAY | mmu03010 | 2 (4.55%) | 171 (1.99%) | 0.217429124964 | 0.562799768578 | Mrpl36  Rpl35a | web link | no local link |
| Sphingolipid metabolism | KEGG PATHWAY | mmu00600 | 1 (2.27%) | 48 (0.56%) | 0.218592206583 | 0.562799768578 | Gba2 | web link | no local link |
| Notch signaling pathway | KEGG PATHWAY | mmu04330 | 1 (2.27%) | 49 (0.57%) | 0.222608788699 | 0.562799768578 | Ncor2 | web link | no local link |
| Amino sugar and nucleotide sugar metabolism | KEGG PATHWAY | mmu00520 | 1 (2.27%) | 49 (0.57%) | 0.222608788699 | 0.562799768578 | Gmppa | web link | no local link |
| Cholesterol metabolism | KEGG PATHWAY | mmu04979 | 1 (2.27%) | 49 (0.57%) | 0.222608788699 | 0.562799768578 | Angptl4 | web link | no local link |
| Arginine and proline metabolism | KEGG PATHWAY | mmu00330 | 1 (2.27%) | 50 (0.58%) | 0.226605191701 | 0.562799768578 | L3hypdh | web link | no local link |
| Fanconi anemia pathway | KEGG PATHWAY | mmu03460 | 1 (2.27%) | 51 (0.59%) | 0.230581514623 | 0.562799768578 | Fance | web link | no local link |
| Endocrine and other factor-regulated calcium reabsorption | KEGG PATHWAY | mmu04961 | 1 (2.27%) | 57 (0.66%) | 0.254023256791 | 0.562799768578 | Esr1 | web link | no local link |
| VEGF signaling pathway | KEGG PATHWAY | mmu04370 | 1 (2.27%) | 58 (0.67%) | 0.257861752488 | 0.562799768578 | Vegfa | web link | no local link |
| Calcium signaling pathway | KEGG PATHWAY | mmu04020 | 2 (4.55%) | 194 (2.25%) | 0.261082482256 | 0.562799768578 | Asph  Phkb | web link | no local link |
| Fatty acid metabolism | KEGG PATHWAY | mmu01212 | 1 (2.27%) | 61 (0.71%) | 0.269261790196 | 0.562799768578 | Elovl5 | web link | no local link |
| Longevity regulating pathway - multiple species | KEGG PATHWAY | mmu04213 | 1 (2.27%) | 62 (0.72%) | 0.273023634262 | 0.562799768578 | Rps6kb2 | web link | no local link |
| PI3K-Akt signaling pathway | KEGG PATHWAY | mmu04151 | 3 (6.82%) | 358 (4.16%) | 0.276230881792 | 0.562799768578 | Vegfa  Ppp2r3d  Rps6kb2 | web link | no local link |
| Human papillomavirus infection | KEGG PATHWAY | mmu05165 | 3 (6.82%) | 361 (4.19%) | 0.280441959832 | 0.562799768578 | Vegfa  Ppp2r3d  Rps6kb2 | web link | no local link |
| Glutathione metabolism | KEGG PATHWAY | mmu00480 | 1 (2.27%) | 64 (0.74%) | 0.280490631216 | 0.562799768578 | Gstm4 | web link | no local link |
| Metabolism of xenobiotics by cytochrome P450 | KEGG PATHWAY | mmu00980 | 1 (2.27%) | 66 (0.77%) | 0.28788265793 | 0.562799768578 | Gstm4 | web link | no local link |
| Pathways in cancer | KEGG PATHWAY | mmu05200 | 4 (9.09%) | 535 (6.22%) | 0.291375067638 | 0.562799768578 | Vegfa  Esr1  Gstm4  Rps6kb2 | web link | no local link |
| Glycolysis / Gluconeogenesis | KEGG PATHWAY | mmu00010 | 1 (2.27%) | 67 (0.78%) | 0.291550787531 | 0.562799768578 | Fbp1 | web link | no local link |
| Renal cell carcinoma | KEGG PATHWAY | mmu05211 | 1 (2.27%) | 68 (0.79%) | 0.295200449789 | 0.562799768578 | Vegfa | web link | no local link |
| Drug metabolism - cytochrome P450 | KEGG PATHWAY | mmu00982 | 1 (2.27%) | 68 (0.79%) | 0.295200449789 | 0.562799768578 | Gstm4 | web link | no local link |
| Acute myeloid leukemia | KEGG PATHWAY | mmu05221 | 1 (2.27%) | 69 (0.80%) | 0.298831735528 | 0.562799768578 | Rps6kb2 | web link | no local link |
| Prolactin signaling pathway | KEGG PATHWAY | mmu04917 | 1 (2.27%) | 72 (0.84%) | 0.309616235328 | 0.573551386755 | Esr1 | web link | no local link |
| Platinum drug resistance | KEGG PATHWAY | mmu01524 | 1 (2.27%) | 77 (0.89%) | 0.327231222524 | 0.580521589196 | Gstm4 | web link | no local link |
| Rheumatoid arthritis | KEGG PATHWAY | mmu05323 | 1 (2.27%) | 84 (0.98%) | 0.351156281807 | 0.580521589196 | Vegfa | web link | no local link |
| ErbB signaling pathway | KEGG PATHWAY | mmu04012 | 1 (2.27%) | 84 (0.98%) | 0.351156281807 | 0.580521589196 | Rps6kb2 | web link | no local link |
| Cardiac muscle contraction | KEGG PATHWAY | mmu04260 | 1 (2.27%) | 86 (1.00%) | 0.357837911554 | 0.580521589196 | Asph | web link | no local link |
| PPAR signaling pathway | KEGG PATHWAY | mmu03320 | 1 (2.27%) | 87 (1.01%) | 0.36115346329 | 0.580521589196 | Angptl4 | web link | no local link |
| Drug metabolism - other enzymes | KEGG PATHWAY | mmu00983 | 1 (2.27%) | 87 (1.01%) | 0.36115346329 | 0.580521589196 | Gstm4 | web link | no local link |
| PD-L1 expression and PD-1 checkpoint pathway in cancer | KEGG PATHWAY | mmu05235 | 1 (2.27%) | 88 (1.02%) | 0.364452283571 | 0.580521589196 | Rps6kb2 | web link | no local link |
| Colorectal cancer | KEGG PATHWAY | mmu05210 | 1 (2.27%) | 88 (1.02%) | 0.364452283571 | 0.580521589196 | Rps6kb2 | web link | no local link |
| Longevity regulating pathway | KEGG PATHWAY | mmu04211 | 1 (2.27%) | 90 (1.05%) | 0.371000059289 | 0.580521589196 | Rps6kb2 | web link | no local link |
| Antigen processing and presentation | KEGG PATHWAY | mmu04612 | 1 (2.27%) | 91 (1.06%) | 0.374249178494 | 0.580521589196 | Ifi30 | web link | no local link |
| Retinol metabolism | KEGG PATHWAY | mmu00830 | 1 (2.27%) | 91 (1.06%) | 0.374249178494 | 0.580521589196 | Dhrs3 | web link | no local link |
| Human cytomegalovirus infection | KEGG PATHWAY | mmu05163 | 2 (4.55%) | 256 (2.97%) | 0.378096946096 | 0.580521589196 | Vegfa  Rps6kb2 | web link | no local link |
| Chemical carcinogenesis | KEGG PATHWAY | mmu05204 | 1 (2.27%) | 94 (1.09%) | 0.383898435853 | 0.580521589196 | Gstm4 | web link | no local link |
| mRNA surveillance pathway | KEGG PATHWAY | mmu03015 | 1 (2.27%) | 96 (1.12%) | 0.390250328042 | 0.580521589196 | Ppp2r3d | web link | no local link |
| Glycerophospholipid metabolism | KEGG PATHWAY | mmu00564 | 1 (2.27%) | 97 (1.13%) | 0.393402229542 | 0.580521589196 | Taz | web link | no local link |
| Choline metabolism in cancer | KEGG PATHWAY | mmu05231 | 1 (2.27%) | 99 (1.15%) | 0.399658338218 | 0.580521589196 | Rps6kb2 | web link | no local link |
| Endocytosis | KEGG PATHWAY | mmu04144 | 2 (4.55%) | 270 (3.14%) | 0.403692669102 | 0.580521589196 | Smurf1  Bin1 | web link | no local link |
| AGE-RAGE signaling pathway in diabetic complications | KEGG PATHWAY | mmu04933 | 1 (2.27%) | 101 (1.17%) | 0.405851376518 | 0.580521589196 | Vegfa | web link | no local link |
| Chagas disease (American trypanosomiasis) | KEGG PATHWAY | mmu05142 | 1 (2.27%) | 103 (1.20%) | 0.41198196564 | 0.581924526466 | Cflar | web link | no local link |
| NF-kappa B signaling pathway | KEGG PATHWAY | mmu04064 | 1 (2.27%) | 108 (1.25%) | 0.427039246139 | 0.595746108812 | Cflar | web link | no local link |
| Insulin resistance | KEGG PATHWAY | mmu04931 | 1 (2.27%) | 110 (1.28%) | 0.432956069317 | 0.596634583327 | Rps6kb2 | web link | no local link |
| TNF signaling pathway | KEGG PATHWAY | mmu04668 | 1 (2.27%) | 113 (1.31%) | 0.441719500408 | 0.601377151158 | Cflar | web link | no local link |
| Carbon metabolism | KEGG PATHWAY | mmu01200 | 1 (2.27%) | 121 (1.41%) | 0.464446638978 | 0.615882798379 | Fbp1 | web link | no local link |
| Sphingolipid signaling pathway | KEGG PATHWAY | mmu04071 | 1 (2.27%) | 124 (1.44%) | 0.472734069291 | 0.615882798379 | Ppp2r3d | web link | no local link |
| Platelet activation | KEGG PATHWAY | mmu04611 | 1 (2.27%) | 125 (1.45%) | 0.475468593212 | 0.615882798379 | Snap23 | web link | no local link |
| Relaxin signaling pathway | KEGG PATHWAY | mmu04926 | 1 (2.27%) | 131 (1.52%) | 0.491587042297 | 0.615882798379 | Vegfa | web link | no local link |
| Estrogen signaling pathway | KEGG PATHWAY | mmu04915 | 1 (2.27%) | 134 (1.56%) | 0.4994637217 | 0.615882798379 | Esr1 | web link | no local link |
| Dopaminergic synapse | KEGG PATHWAY | mmu04728 | 1 (2.27%) | 135 (1.57%) | 0.502062682786 | 0.615882798379 | Ppp2r3d | web link | no local link |
| Apoptosis | KEGG PATHWAY | mmu04210 | 1 (2.27%) | 136 (1.58%) | 0.504648454291 | 0.615882798379 | Cflar | web link | no local link |
| Purine metabolism | KEGG PATHWAY | mmu00230 | 1 (2.27%) | 136 (1.58%) | 0.504648454291 | 0.615882798379 | Ntpcr | web link | no local link |
| Signaling pathways regulating pluripotency of stem cells | KEGG PATHWAY | mmu04550 | 1 (2.27%) | 138 (1.60%) | 0.509780689788 | 0.615882798379 | Lifr | web link | no local link |
| Apelin signaling pathway | KEGG PATHWAY | mmu04371 | 1 (2.27%) | 139 (1.61%) | 0.512327283607 | 0.615882798379 | Rps6kb2 | web link | no local link |
| Ubiquitin mediated proteolysis | KEGG PATHWAY | mmu04120 | 1 (2.27%) | 139 (1.61%) | 0.512327283607 | 0.615882798379 | Smurf1 | web link | no local link |
| Gastric cancer | KEGG PATHWAY | mmu05226 | 1 (2.27%) | 150 (1.74%) | 0.539500396716 | 0.640662228835 | Rps6kb2 | web link | no local link |
| Adrenergic signaling in cardiomyocytes | KEGG PATHWAY | mmu04261 | 1 (2.27%) | 152 (1.77%) | 0.544279415647 | 0.640662228835 | Ppp2r3d | web link | no local link |
| mTOR signaling pathway | KEGG PATHWAY | mmu04150 | 1 (2.27%) | 155 (1.80%) | 0.551357193556 | 0.642302710019 | Rps6kb2 | web link | no local link |
| Hepatitis C | KEGG PATHWAY | mmu05160 | 1 (2.27%) | 160 (1.86%) | 0.562915468615 | 0.649075999525 | Cflar | web link | no local link |
| Jak-STAT signaling pathway | KEGG PATHWAY | mmu04630 | 1 (2.27%) | 165 (1.92%) | 0.574182567376 | 0.653828722792 | Lifr | web link | no local link |
| RNA transport | KEGG PATHWAY | mmu03013 | 1 (2.27%) | 167 (1.94%) | 0.578609489196 | 0.653828722792 | Thoc7 | web link | no local link |
| Necroptosis | KEGG PATHWAY | mmu04217 | 1 (2.27%) | 177 (2.06%) | 0.600078143642 | 0.671374556748 | Cflar | web link | no local link |
| Axon guidance | KEGG PATHWAY | mmu04360 | 1 (2.27%) | 180 (2.09%) | 0.606307637303 | 0.671693755051 | Ablim2 | web link | no local link |
| Transcriptional misregulation in cancer | KEGG PATHWAY | mmu05202 | 1 (2.27%) | 183 (2.13%) | 0.612442267256 | 0.671902681553 | Ncor1 | web link | no local link |
| Focal adhesion | KEGG PATHWAY | mmu04510 | 1 (2.27%) | 199 (2.31%) | 0.643615514904 | 0.699313011386 | Vegfa | web link | no local link |
| Rap1 signaling pathway | KEGG PATHWAY | mmu04015 | 1 (2.27%) | 214 (2.49%) | 0.670607183191 | 0.720386259573 | Vegfa | web link | no local link |
| Kaposi sarcoma-associated herpesvirus infection | KEGG PATHWAY | mmu05167 | 1 (2.27%) | 217 (2.52%) | 0.675760562077 | 0.720386259573 | Vegfa | web link | no local link |
| Epstein-Barr virus infection | KEGG PATHWAY | mmu05169 | 1 (2.27%) | 230 (2.67%) | 0.69719604924 | 0.727701799546 | Ncor2 | web link | no local link |
| Thermogenesis | KEGG PATHWAY | mmu04714 | 1 (2.27%) | 232 (2.70%) | 0.700368448073 | 0.727701799546 | Rps6kb2 | web link | no local link |
| Ras signaling pathway | KEGG PATHWAY | mmu04014 | 1 (2.27%) | 233 (2.71%) | 0.701942443809 | 0.727701799546 | Vegfa | web link | no local link |
| Human immunodeficiency virus 1 infection | KEGG PATHWAY | mmu05170 | 1 (2.27%) | 239 (2.78%) | 0.711218120307 | 0.730614978133 | Rps6kb2 | web link | no local link |
| MicroRNAs in cancer | KEGG PATHWAY | mmu05206 | 1 (2.27%) | 282 (3.28%) | 0.769919175802 | 0.783791593384 | Vegfa | web link | no local link |
| Cytokine-cytokine receptor interaction | KEGG PATHWAY | mmu04060 | 1 (2.27%) | 293 (3.40%) | 0.782953800432 | 0.784102331133 | Lifr | web link | no local link |
| MAPK signaling pathway | KEGG PATHWAY | mmu04010 | 1 (2.27%) | 294 (3.42%) | 0.784102331133 | 0.784102331133 | Vegfa | web link | no local link |
