# Supplementary material for: Regulation of Alternative Splicing of Lipid Metabolism Genes in Sepsis-Induced Liver Damage by RNA-Binding Proteins
Source: Inflammation. 2024 May 9;47(6):1952–68. doi: 10.1007/s10753-024-02017-2 (PMC11607010; doi:10.1007/s10753-024-02017-2)
Supplement: Supplementary file 1 — Supplementary file1 (ZIP 38655 KB) [file 10753_2024_2017_MOESM1_ESM.zip › Sup6_AS_KEGG.zip!/Sup6_AS_KEGG/ras/co_RAS/GO_KEGG/CLP_vs_Sham_and_CLPDCA_vs_Sham_co_RAS/coDown_nir_KEGG_analysis/coDown_nir_KEGG_top.pdf]

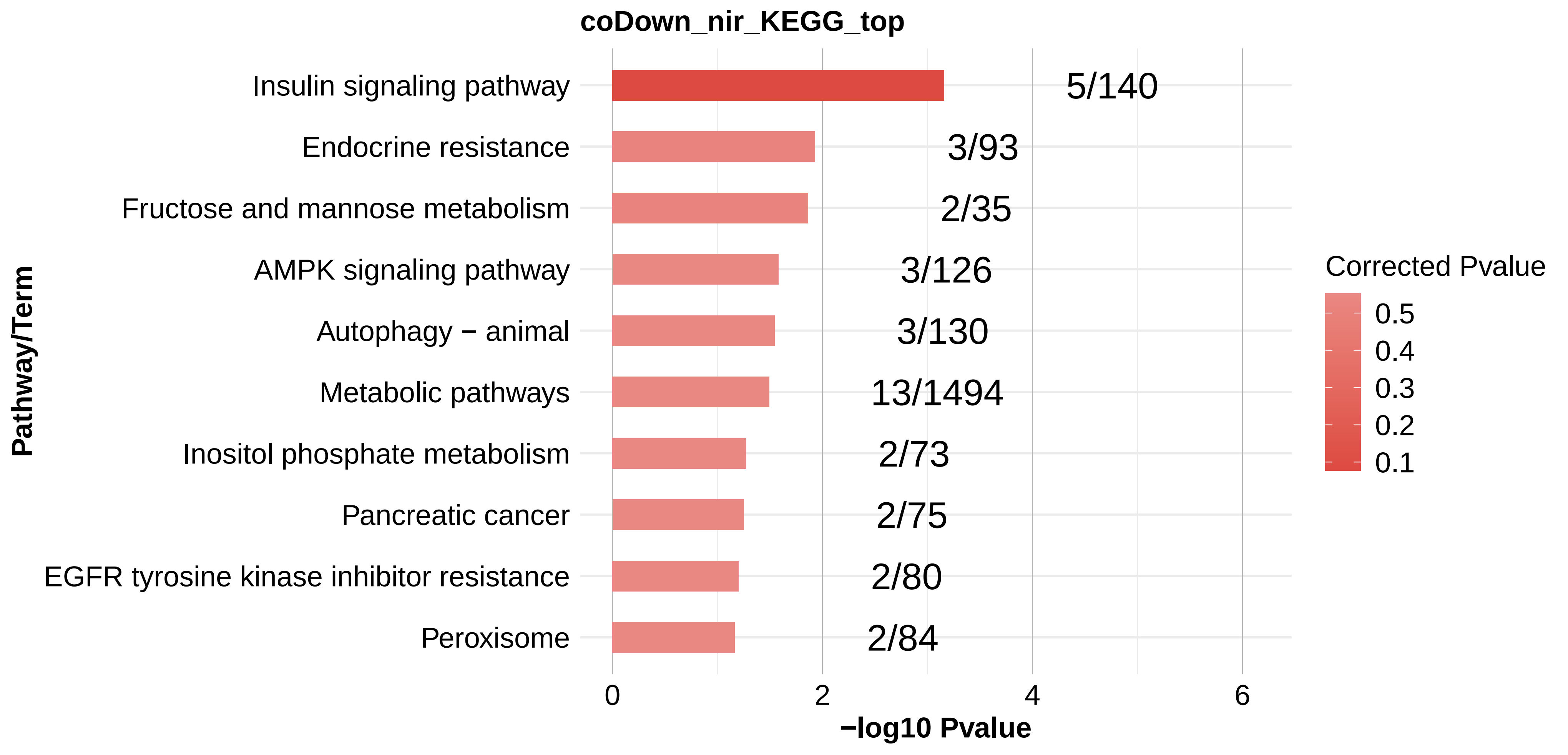
coDown_nir_KEGG_top
Pathway/Term
Insulin signaling pathway
Endocrine resistance
Fructose and mannose metabolism
AMPK signaling pathway
Autophagy − animal
Metabolic pathways
Inositol phosphate metabolism
Pancreatic cancer
EGFR tyrosine kinase inhibitor resistance
Peroxisome
5/140
3/93
2/35
3/126
3/130
13/1494
2/73
2/75
2/80
2/84
0
2
4
6
−log10 Pvalue
Corrected Pvalue
0.5
0.4
0.3
0.2
0.1
