# Supplementary material for: Regulation of Alternative Splicing of Lipid Metabolism Genes in Sepsis-Induced Liver Damage by RNA-Binding Proteins
Source: Inflammation. 2024 May 9;47(6):1952–68. doi: 10.1007/s10753-024-02017-2 (PMC11607010; doi:10.1007/s10753-024-02017-2)
Supplement: Supplementary file 1 — Supplementary file1 (ZIP 38655 KB) [file 10753_2024_2017_MOESM1_ESM.zip › Sup6_AS_KEGG.zip!/Sup6_AS_KEGG/ras/CLPDCA_vs_Sham/Ras_KEGG_analysis/Ras_KEGG_pathway.html]

KEGG pathway enrichment analyze result


# KEGG pathway enrichment analyze result


| Term | Database | ID | Input number (253) | Background number (8608) | P-Value | Corrected P-Value | Input | Hyperlink | img |
| --- | --- | --- | --- | --- | --- | --- | --- | --- | --- |
| Metabolic pathways | KEGG PATHWAY | mmu01100 | 70 (27.67%) | 1494 (17.36%) | 2.32591777888e-05 | 0.00614042293624 | Entpd8  Acer2  Cmbl  Tdo2  Bpnt1  Hoga1  Cyp4f13  Idh1  Cept1  Csad  Mtr  Nudt5  Dhtkd1  Sc5d  Nadsyn1  Fbp1  Hsd3b2  Mtmr4  Mcee  Mthfs  Nme4  Ggcx  Dglucy  Pi4ka  Mgst1  Prodh2  Comt  Agxt2  Idh3g  Hagh  Ndufs2  Fpgt  Agpat3  Sgms2  Auh  Pigx  Cyp2r1  Ido2  Gns  Gss  St3gal5  St3gal4  Aldh3a2  Gstm4  Mgam  Gmppa  Dhrs3  Alas2  P4ha1  Bckdhb  Crls1  Cyp4a31  Mvk  Gba2  Hsd17b6  Cdipt  Ntpcr  Upp2  Ahcyl1  Mtmr14  Ampd2  Enpp4  Pcyt2  Mat1a  Gsta3  Atp5j  Elovl5  Rdh11  Xylb  Cox7a2 | web link | no local link |
| Autophagy - animal | KEGG PATHWAY | mmu04140 | 12 (4.74%) | 130 (1.51%) | 0.000425560358019 | 0.0561739672584 | Bcl2l1  Mtmr14  Kras  Stk11  Nras  Rubcn  Rps6kb2  Atg4c  Cflar  Atg13  Mtmr4  Tsc1 | web link | no local link |
| Ferroptosis | KEGG PATHWAY | mmu04216 | 6 (2.37%) | 41 (0.48%) | 0.00115124563443 | 0.10130961583 | Gss  Slc11a2  Slc40a1  Slc39a14  Pcbp2  Steap3 | web link | no local link |
| EGFR tyrosine kinase inhibitor resistance | KEGG PATHWAY | mmu01521 | 8 (3.16%) | 80 (0.93%) | 0.00232411524293 | 0.153391606033 | Bcl2l1  Nras  Egfr  Rps6kb2  Vegfa  Nf1  Fgfr3  Kras | web link | no local link |
| Peroxisome | KEGG PATHWAY | mmu04146 | 8 (3.16%) | 84 (0.98%) | 0.0031612780371 | 0.166915480359 | Paox  Idh1  Mpv17l2  Gnpat  Pxmp2  Mvk  Mpv17  Pex16 | web link | no local link |
| Bladder cancer | KEGG PATHWAY | mmu05219 | 5 (1.98%) | 41 (0.48%) | 0.00665638674817 | 0.249774827822 | Vegfa  Egfr  Fgfr3  Nras  Kras | web link | no local link |
| Lysine degradation | KEGG PATHWAY | mmu00310 | 6 (2.37%) | 59 (0.69%) | 0.00743773624194 | 0.249774827822 | Kmt2c  Aldh3a2  Dhtkd1  Ezh2  Nsd1  Setdb1 | web link | no local link |
| Glycerophospholipid metabolism | KEGG PATHWAY | mmu00564 | 8 (3.16%) | 97 (1.13%) | 0.00756893417642 | 0.249774827822 | Pcyt2  Cept1  Taz  Agpat3  Pnpla6  Gnpat  Crls1  Cdipt | web link | no local link |
| Phosphonate and phosphinate metabolism | KEGG PATHWAY | mmu00440 | 2 (0.79%) | 6 (0.07%) | 0.0119363678883 | 0.298897798044 | Pcyt2  Cept1 | web link | no local link |
| Hepatocellular carcinoma | KEGG PATHWAY | mmu05225 | 11 (4.35%) | 171 (1.99%) | 0.0119747259555 | 0.298897798044 | Bcl2l1  Smarcc2  Gstm4  Csnk1a1  Nras  Egfr  Gsta3  Mgst1  Rps6kb2  Smarca4  Kras | web link | no local link |
| Complement and coagulation cascades | KEGG PATHWAY | mmu04610 | 7 (2.77%) | 88 (1.02%) | 0.0146691170054 | 0.298897798044 | Cfh  C4bp  Masp1  Serpina1a  Clu  F10  Proc | web link | no local link |
| Arginine and proline metabolism | KEGG PATHWAY | mmu00330 | 5 (1.98%) | 50 (0.58%) | 0.0152121796672 | 0.298897798044 | Aldh3a2  Hoga1  P4ha1  Prodh2  L3hypdh | web link | no local link |
| Longevity regulating pathway | KEGG PATHWAY | mmu04211 | 7 (2.77%) | 90 (1.05%) | 0.0164380419443 | 0.298897798044 | Adipor2  Stk11  Nras  Rps6kb2  Atg13  Kras  Tsc1 | web link | no local link |
| Axon guidance | KEGG PATHWAY | mmu04360 | 11 (4.35%) | 180 (2.09%) | 0.0170232269572 | 0.298897798044 | Arhgef12  Ngef  Cdc42  Rgs3  Nras  Pard6a  Neo1  Ablim2  Sema4a  Kras  Sema4g | web link | no local link |
| Cysteine and methionine metabolism | KEGG PATHWAY | mmu00270 | 5 (1.98%) | 52 (0.60%) | 0.0178017841518 | 0.298897798044 | Mtr  Gss  Ahcyl1  Mat1a  Agxt2 | web link | no local link |
| Proteoglycans in cancer | KEGG PATHWAY | mmu05205 | 12 (4.74%) | 206 (2.39%) | 0.0183289604677 | 0.298897798044 | Arhgef12  Esr1  Sdc1  Ppp1ca  Egfr  Rps6kb2  Vegfa  Fas  Flnb  Cdc42  Nras  Kras | web link | no local link |
| Ubiquitin mediated proteolysis | KEGG PATHWAY | mmu04120 | 9 (3.56%) | 139 (1.61%) | 0.0211836809413 | 0.298897798044 | Smurf1  Ube2i  Klhl13  Ube2j2  Birc6  Btrc  Ube2d3  Xiap  Eloc | web link | no local link |
| Insulin signaling pathway | KEGG PATHWAY | mmu04910 | 9 (3.56%) | 140 (1.63%) | 0.0220798542282 | 0.298897798044 | Exoc7  Ppp1ca  Nras  Prkar1a  Rps6kb2  Fbp1  Phkb  Kras  Tsc1 | web link | no local link |
| Pancreatic cancer | KEGG PATHWAY | mmu05212 | 6 (2.37%) | 75 (0.87%) | 0.0225907008554 | 0.298897798044 | Bcl2l1  Egfr  Rps6kb2  Vegfa  Cdc42  Kras | web link | no local link |
| mRNA surveillance pathway | KEGG PATHWAY | mmu03015 | 7 (2.77%) | 96 (1.12%) | 0.0226437725791 | 0.298897798044 | Ppp1ca  Srrm1  Ppp2r3a  Ppp2r3d  Sap18  Cpsf7  Nudt21 | web link | no local link |
| Valine, leucine and isoleucine degradation | KEGG PATHWAY | mmu00280 | 5 (1.98%) | 56 (0.65%) | 0.0238186568503 | 0.29943454326 | Aldh3a2  Auh  Mcee  Bckdhb  Agxt2 | web link | no local link |
| Platinum drug resistance | KEGG PATHWAY | mmu01524 | 6 (2.37%) | 77 (0.89%) | 0.0253533837908 | 0.304240605489 | Bcl2l1  Fas  Gstm4  Gsta3  Xiap  Mgst1 | web link | no local link |
| VEGF signaling pathway | KEGG PATHWAY | mmu04370 | 5 (1.98%) | 58 (0.67%) | 0.0272645486487 | 0.31294960188 | Nras  Cdc42  Vegfa  Mapkapk2  Kras | web link | no local link |
| AMPK signaling pathway | KEGG PATHWAY | mmu04152 | 8 (3.16%) | 126 (1.46%) | 0.032029490232 | 0.352324392552 | Adra1a  Stk11  Adipor2  Ppp2r3a  Ppp2r3d  Rps6kb2  Fbp1  Tsc1 | web link | no local link |
| Glutathione metabolism | KEGG PATHWAY | mmu00480 | 5 (1.98%) | 64 (0.74%) | 0.0394392878749 | 0.39928470363 | Idh1  Mgst1  Gstm4  Gss  Gsta3 | web link | no local link |
| Hedgehog signaling pathway | KEGG PATHWAY | mmu04340 | 4 (1.58%) | 44 (0.51%) | 0.0394797757727 | 0.39928470363 | Smurf1  Spop  Btrc  Csnk1a1 | web link | no local link |
| Thermogenesis | KEGG PATHWAY | mmu04714 | 12 (4.74%) | 232 (2.70%) | 0.0408359355985 | 0.39928470363 | Smarcc2  Ndufaf4  Nras  Rps6kb2  Atp5j  Ndufs2  Ndufaf1  Ndufaf2  Cox7a2  Kras  Smarca4  Tsc1 | web link | no local link |
| Renal cell carcinoma | KEGG PATHWAY | mmu05211 | 5 (1.98%) | 68 (0.79%) | 0.0491353833231 | 0.463276471332 | Nras  Cdc42  Vegfa  Eloc  Kras | web link | no local link |
| Tryptophan metabolism | KEGG PATHWAY | mmu00380 | 4 (1.58%) | 48 (0.56%) | 0.0517316364319 | 0.470936276483 | Aldh3a2  Dhtkd1  Tdo2  Ido2 | web link | no local link |
| Endocrine resistance | KEGG PATHWAY | mmu01522 | 6 (2.37%) | 93 (1.08%) | 0.0556576275266 | 0.489787122234 | Esr1  Egfr  Nras  Rps6kb2  Ncor1  Kras | web link | no local link |
| Apoptosis - multiple species | KEGG PATHWAY | mmu04215 | 3 (1.19%) | 32 (0.37%) | 0.0665895188634 | 0.567084934837 | Bcl2l1  Birc6  Xiap | web link | no local link |
| Fructose and mannose metabolism | KEGG PATHWAY | mmu00051 | 3 (1.19%) | 35 (0.41%) | 0.0824979270372 | 0.680607898057 | Fpgt  Gmppa  Fbp1 | web link | no local link |
| Spliceosome | KEGG PATHWAY | mmu03040 | 7 (2.77%) | 133 (1.55%) | 0.0966736435411 | 0.773389148329 | Srsf5  Srsf2  Hnrnpa1  Snrpa1  Hnrnpu  Rbm25  Sf3a2 | web link | no local link |
| 2-Oxocarboxylic acid metabolism | KEGG PATHWAY | mmu01210 | 2 (0.79%) | 19 (0.22%) | 0.106085785943 | 0.777962430251 | Idh1  Idh3g | web link | no local link |
| Steroid biosynthesis | KEGG PATHWAY | mmu00100 | 2 (0.79%) | 19 (0.22%) | 0.106085785943 | 0.777962430251 | Sc5d  Cyp2r1 | web link | no local link |
| One carbon pool by folate | KEGG PATHWAY | mmu00670 | 2 (0.79%) | 19 (0.22%) | 0.106085785943 | 0.777962430251 | Mtr  Mthfs | web link | no local link |
| Drug metabolism - other enzymes | KEGG PATHWAY | mmu00983 | 5 (1.98%) | 87 (1.01%) | 0.112546126736 | 0.803031823195 | Nme4  Upp2  Mgst1  Gstm4  Gsta3 | web link | no local link |
| Central carbon metabolism in cancer | KEGG PATHWAY | mmu05230 | 4 (1.58%) | 67 (0.78%) | 0.133863740914 | 0.855446505434 | Nras  Egfr  Fgfr3  Kras | web link | no local link |
| Basal transcription factors | KEGG PATHWAY | mmu03022 | 3 (1.19%) | 44 (0.51%) | 0.138434153962 | 0.855446505434 | Taf13  Gtf2i  Gtf2a2 | web link | no local link |
| Neurotrophin signaling pathway | KEGG PATHWAY | mmu04722 | 6 (2.37%) | 121 (1.41%) | 0.145566557445 | 0.855446505434 | Nras  Map2k5  Sh2b1  Kras  Mapkapk2  Cdc42 | web link | no local link |
| Terpenoid backbone biosynthesis | KEGG PATHWAY | mmu00900 | 2 (0.79%) | 23 (0.27%) | 0.145630502899 | 0.855446505434 | Pcyox1  Mvk | web link | no local link |
| Sphingolipid signaling pathway | KEGG PATHWAY | mmu04071 | 6 (2.37%) | 124 (1.44%) | 0.157789440635 | 0.855446505434 | Acer2  Ppp2r3a  Nras  Ppp2r3d  Sgms2  Kras | web link | no local link |
| Prolactin signaling pathway | KEGG PATHWAY | mmu04917 | 4 (1.58%) | 72 (0.84%) | 0.16114625487 | 0.855446505434 | Prlr  Kras  Nras  Esr1 | web link | no local link |
| Adherens junction | KEGG PATHWAY | mmu04520 | 4 (1.58%) | 72 (0.84%) | 0.16114625487 | 0.855446505434 | Baiap2  Afdn  Egfr  Cdc42 | web link | no local link |
| Phosphatidylinositol signaling system | KEGG PATHWAY | mmu04070 | 5 (1.98%) | 98 (1.14%) | 0.161263950624 | 0.855446505434 | Ppip5k2  Mtmr14  Mtmr4  Pi4ka  Cdipt | web link | no local link |
| Choline metabolism in cancer | KEGG PATHWAY | mmu05231 | 5 (1.98%) | 99 (1.15%) | 0.166065534988 | 0.855446505434 | Kras  Nras  Tsc1  Egfr  Rps6kb2 | web link | no local link |
| Sphingolipid metabolism | KEGG PATHWAY | mmu00600 | 3 (1.19%) | 48 (0.56%) | 0.166516814685 | 0.855446505434 | Acer2  Gba2  Sgms2 | web link | no local link |
| Inositol phosphate metabolism | KEGG PATHWAY | mmu00562 | 4 (1.58%) | 73 (0.85%) | 0.166832106721 | 0.855446505434 | Mtmr14  Mtmr4  Pi4ka  Cdipt | web link | no local link |
| PI3K-Akt signaling pathway | KEGG PATHWAY | mmu04151 | 14 (5.53%) | 358 (4.16%) | 0.168868301573 | 0.855446505434 | Bcl2l1  Stk11  Ghr  Prlr  Nras  Ppp2r3a  Egfr  Ppp2r3d  Rps6kb2  Vegfa  Magi1  Kras  Fgfr3  Tsc1 | web link | no local link |
| Endocytosis | KEGG PATHWAY | mmu04144 | 11 (4.35%) | 270 (3.14%) | 0.171459851637 | 0.855446505434 | Smurf1  Tsg101  Washc1  Arap2  Egfr  Sh3glb2  Pard6a  Cdc42  Bin1  Fgfr3  Asap3 | web link | no local link |
| mTOR signaling pathway | KEGG PATHWAY | mmu04150 | 7 (2.77%) | 155 (1.80%) | 0.171545869719 | 0.855446505434 | Fnip1  Stk11  Nras  Wdr59  Rps6kb2  Kras  Tsc1 | web link | no local link |
| Cholesterol metabolism | KEGG PATHWAY | mmu04979 | 3 (1.19%) | 49 (0.57%) | 0.173784171689 | 0.855446505434 | Abcg8  Angptl4  Apoc3 | web link | no local link |
| Human papillomavirus infection | KEGG PATHWAY | mmu05165 | 14 (5.53%) | 361 (4.19%) | 0.176408043463 | 0.855446505434 | Csnk1a1  Cdc42  Nras  Irf9  Ppp2r3a  Egfr  Pard6a  Rps6kb2  Vegfa  Ppp2r3d  Magi1  Fas  Kras  Tsc1 | web link | no local link |
| Rap1 signaling pathway | KEGG PATHWAY | mmu04015 | 9 (3.56%) | 214 (2.49%) | 0.1786818349 | 0.855446505434 | Nras  Afdn  Egfr  Pard6a  Vegfa  Magi1  Cdc42  Fgfr3  Kras | web link | no local link |
| Chronic myeloid leukemia | KEGG PATHWAY | mmu05220 | 4 (1.58%) | 76 (0.88%) | 0.184303733075 | 0.855446505434 | Bcl2l1  Ctbp1  Nras  Kras | web link | no local link |
| Biosynthesis of amino acids | KEGG PATHWAY | mmu01230 | 4 (1.58%) | 78 (0.91%) | 0.196271203559 | 0.855446505434 | Mtr  Idh1  Mat1a  Idh3g | web link | no local link |
| Protein processing in endoplasmic reticulum | KEGG PATHWAY | mmu04141 | 7 (2.77%) | 163 (1.89%) | 0.203691252014 | 0.855446505434 | Sil1  Ube2j2  Ube2d3  Dnajb12  Derl3  Fbxo6  Sec31a | web link | no local link |
| Apoptosis | KEGG PATHWAY | mmu04210 | 6 (2.37%) | 136 (1.58%) | 0.210767801617 | 0.855446505434 | Bcl2l1  Fas  Nras  Cflar  Xiap  Kras | web link | no local link |
| Purine metabolism | KEGG PATHWAY | mmu00230 | 6 (2.37%) | 136 (1.58%) | 0.210767801617 | 0.855446505434 | Nme4  Ntpcr  Ampd2  Enpp4  Nudt5  Entpd8 | web link | no local link |
| RNA transport | KEGG PATHWAY | mmu03013 | 7 (2.77%) | 167 (1.94%) | 0.220578765068 | 0.855446505434 | Ube2i  Srrm1  Gemin6  Nup37  Eif4g3  Sap18  Thoc7 | web link | no local link |
| Tight junction | KEGG PATHWAY | mmu04530 | 7 (2.77%) | 167 (1.94%) | 0.220578765068 | 0.855446505434 | Stk11  Afdn  Pard6a  Myl6  Nf2  Magi1  Cdc42 | web link | no local link |
| Glyoxylate and dicarboxylate metabolism | KEGG PATHWAY | mmu00630 | 2 (0.79%) | 31 (0.36%) | 0.230993495296 | 0.855446505434 | Hoga1  Mcee | web link | no local link |
| Focal adhesion | KEGG PATHWAY | mmu04510 | 8 (3.16%) | 199 (2.31%) | 0.231280035596 | 0.855446505434 | Ppp1ca  Egfr  Xiap  Vegfa  Arhgap5  Flnb  Zyx  Cdc42 | web link | no local link |
| C-type lectin receptor signaling pathway | KEGG PATHWAY | mmu04625 | 5 (1.98%) | 112 (1.30%) | 0.233076435837 | 0.855446505434 | Arhgef12  Irf9  Nras  Mapkapk2  Kras | web link | no local link |
| ErbB signaling pathway | KEGG PATHWAY | mmu04012 | 4 (1.58%) | 84 (0.98%) | 0.2334831513 | 0.855446505434 | Nras  Kras  Egfr  Rps6kb2 | web link | no local link |
| Ribosome | KEGG PATHWAY | mmu03010 | 7 (2.77%) | 171 (1.99%) | 0.237946803184 | 0.855446505434 | Mrpl36  Rpl38  Rpl35a  Mrps2  Mrps17  Rpl15  Uba52 | web link | no local link |
| Autophagy - other | KEGG PATHWAY | mmu04136 | 2 (0.79%) | 32 (0.37%) | 0.241953760391 | 0.855446505434 | Atg4c  Atg13 | web link | no local link |
| Citrate cycle (TCA cycle) | KEGG PATHWAY | mmu00020 | 2 (0.79%) | 32 (0.37%) | 0.241953760391 | 0.855446505434 | Idh1  Idh3g | web link | no local link |
| Endometrial cancer | KEGG PATHWAY | mmu05213 | 3 (1.19%) | 58 (0.67%) | 0.242525773489 | 0.855446505434 | Nras  Egfr  Kras | web link | no local link |
| Pyrimidine metabolism | KEGG PATHWAY | mmu00240 | 3 (1.19%) | 58 (0.67%) | 0.242525773489 | 0.855446505434 | Nme4  Upp2  Entpd8 | web link | no local link |
| Fluid shear stress and atherosclerosis | KEGG PATHWAY | mmu05418 | 6 (2.37%) | 143 (1.66%) | 0.244217985152 | 0.855446505434 | Sdc1  Gstm4  Gsta3  Mgst1  Vegfa  Map2k5 | web link | no local link |
| Gap junction | KEGG PATHWAY | mmu04540 | 4 (1.58%) | 86 (1.00%) | 0.246255474608 | 0.855446505434 | Nras  Egfr  Map2k5  Kras | web link | no local link |
| Ras signaling pathway | KEGG PATHWAY | mmu04014 | 9 (3.56%) | 233 (2.71%) | 0.247021296336 | 0.855446505434 | Bcl2l1  Nras  Afdn  Egfr  Vegfa  Nf1  Cdc42  Fgfr3  Kras | web link | no local link |
| MAPK signaling pathway | KEGG PATHWAY | mmu04010 | 11 (4.35%) | 294 (3.42%) | 0.247295641785 | 0.855446505434 | Nf1  Nras  Egfr  Fgfr3  Vegfa  Map2k5  Fas  Flnb  Cdc42  Mapkapk2  Kras | web link | no local link |
| PPAR signaling pathway | KEGG PATHWAY | mmu03320 | 4 (1.58%) | 87 (1.01%) | 0.252699298601 | 0.855446505434 | Cyp4a31  Angptl4  Apoc3  Ubc | web link | no local link |
| Propanoate metabolism | KEGG PATHWAY | mmu00640 | 2 (0.79%) | 33 (0.38%) | 0.252936452606 | 0.855446505434 | Mcee  Bckdhb | web link | no local link |
| PD-L1 expression and PD-1 checkpoint pathway in cancer | KEGG PATHWAY | mmu05235 | 4 (1.58%) | 88 (1.02%) | 0.259178233209 | 0.855446505434 | Nras  Kras  Egfr  Rps6kb2 | web link | no local link |
| Colorectal cancer | KEGG PATHWAY | mmu05210 | 4 (1.58%) | 88 (1.02%) | 0.259178233209 | 0.855446505434 | Nras  Kras  Egfr  Rps6kb2 | web link | no local link |
| Breast cancer | KEGG PATHWAY | mmu05224 | 6 (2.37%) | 147 (1.71%) | 0.263998882234 | 0.855446505434 | Csnk1a1  Esr1  Egfr  Nras  Rps6kb2  Kras | web link | no local link |
| Thyroid hormone signaling pathway | KEGG PATHWAY | mmu04919 | 5 (1.98%) | 118 (1.37%) | 0.266299426462 | 0.855446505434 | Nras  Thra  Kras  Ncor1  Esr1 | web link | no local link |
| GnRH signaling pathway | KEGG PATHWAY | mmu04912 | 4 (1.58%) | 90 (1.05%) | 0.27223155815 | 0.855446505434 | Nras  Cdc42  Egfr  Kras | web link | no local link |
| Longevity regulating pathway - multiple species | KEGG PATHWAY | mmu04213 | 3 (1.19%) | 62 (0.72%) | 0.274402356887 | 0.855446505434 | Kras  Nras  Rps6kb2 | web link | no local link |
| Retinol metabolism | KEGG PATHWAY | mmu00830 | 4 (1.58%) | 91 (1.06%) | 0.278801065892 | 0.855446505434 | Cyp4a31  Rdh11  Hsd17b6  Dhrs3 | web link | no local link |
| Ubiquinone and other terpenoid-quinone biosynthesis | KEGG PATHWAY | mmu00130 | 1 (0.40%) | 11 (0.13%) | 0.279884666408 | 0.855446505434 | Ggcx | web link | no local link |
| Taurine and hypotaurine metabolism | KEGG PATHWAY | mmu00430 | 1 (0.40%) | 11 (0.13%) | 0.279884666408 | 0.855446505434 | Csad | web link | no local link |
| Sulfur metabolism | KEGG PATHWAY | mmu00920 | 1 (0.40%) | 11 (0.13%) | 0.279884666408 | 0.855446505434 | Bpnt1 | web link | no local link |
| Mitophagy - animal | KEGG PATHWAY | mmu04137 | 3 (1.19%) | 63 (0.73%) | 0.282444653802 | 0.855446505434 | Bcl2l1  Nras  Kras | web link | no local link |
| Fc gamma R-mediated phagocytosis | KEGG PATHWAY | mmu04666 | 4 (1.58%) | 92 (1.07%) | 0.285395922692 | 0.855446505434 | Cdc42  Asap3  Bin1  Rps6kb2 | web link | no local link |
| Thyroid cancer | KEGG PATHWAY | mmu05216 | 2 (0.79%) | 37 (0.43%) | 0.29688564422 | 0.855446505434 | Nras  Kras | web link | no local link |
| Oxytocin signaling pathway | KEGG PATHWAY | mmu04921 | 6 (2.37%) | 154 (1.79%) | 0.299531276835 | 0.855446505434 | Ppp1ca  Egfr  Nras  Myl6  Map2k5  Kras | web link | no local link |
| TGF-beta signaling pathway | KEGG PATHWAY | mmu04350 | 4 (1.58%) | 95 (1.10%) | 0.30530895167 | 0.855446505434 | Smurf1  Neo1  Tfdp1  Rps6kb2 | web link | no local link |
| Metabolism of xenobiotics by cytochrome P450 | KEGG PATHWAY | mmu00980 | 3 (1.19%) | 66 (0.77%) | 0.306679239944 | 0.855446505434 | Mgst1  Gstm4  Gsta3 | web link | no local link |
| Non-small cell lung cancer | KEGG PATHWAY | mmu05223 | 3 (1.19%) | 66 (0.77%) | 0.306679239944 | 0.855446505434 | Nras  Egfr  Kras | web link | no local link |
| Regulation of actin cytoskeleton | KEGG PATHWAY | mmu04810 | 8 (3.16%) | 217 (2.52%) | 0.306880288708 | 0.855446505434 | Arhgef12  Ppp1ca  Egfr  Nras  Baiap2  Cdc42  Fgfr3  Kras | web link | no local link |
| Pyruvate metabolism | KEGG PATHWAY | mmu00620 | 2 (0.79%) | 38 (0.44%) | 0.307831128849 | 0.855446505434 | Aldh3a2  Hagh | web link | no local link |
| Long-term potentiation | KEGG PATHWAY | mmu04720 | 3 (1.19%) | 67 (0.78%) | 0.314779966461 | 0.865167884271 | Kras  Nras  Ppp1ca | web link | no local link |
| African trypanosomiasis | KEGG PATHWAY | mmu05143 | 2 (0.79%) | 39 (0.45%) | 0.31874319953 | 0.865167884271 | Ido2  Fas | web link | no local link |
| Drug metabolism - cytochrome P450 | KEGG PATHWAY | mmu00982 | 3 (1.19%) | 68 (0.79%) | 0.322885537378 | 0.865167884271 | Mgst1  Gstm4  Gsta3 | web link | no local link |
| Glycine, serine and threonine metabolism | KEGG PATHWAY | mmu00260 | 2 (0.79%) | 40 (0.46%) | 0.329614386221 | 0.865167884271 | Alas2  Agxt2 | web link | no local link |
| Hepatitis C | KEGG PATHWAY | mmu05160 | 6 (2.37%) | 160 (1.86%) | 0.330675146341 | 0.865167884271 | Fas  Irf9  Egfr  Cflar  Nras  Kras | web link | no local link |
| Acute myeloid leukemia | KEGG PATHWAY | mmu05221 | 3 (1.19%) | 69 (0.80%) | 0.330992258755 | 0.865167884271 | Kras  Nras  Rps6kb2 | web link | no local link |
| Porphyrin and chlorophyll metabolism | KEGG PATHWAY | mmu00860 | 2 (0.79%) | 41 (0.48%) | 0.340437683369 | 0.874343341893 | Hccs  Alas2 | web link | no local link |
| AGE-RAGE signaling pathway in diabetic complications | KEGG PATHWAY | mmu04933 | 4 (1.58%) | 101 (1.17%) | 0.345507955548 | 0.874343341893 | Nras  Cdc42  Vegfa  Kras | web link | no local link |
| p53 signaling pathway | KEGG PATHWAY | mmu04115 | 3 (1.19%) | 71 (0.82%) | 0.34719490855 | 0.874343341893 | Bcl2l1  Fas  Steap3 | web link | no local link |
| Melanoma | KEGG PATHWAY | mmu05218 | 3 (1.19%) | 72 (0.84%) | 0.35528398045 | 0.874343341893 | Nras  Egfr  Kras | web link | no local link |
| Jak-STAT signaling pathway | KEGG PATHWAY | mmu04630 | 6 (2.37%) | 165 (1.92%) | 0.356936861202 | 0.874343341893 | Bcl2l1  Ghr  Prlr  Irf9  Egfr  Lifr | web link | no local link |
| Estrogen signaling pathway | KEGG PATHWAY | mmu04915 | 5 (1.98%) | 134 (1.56%) | 0.358794889582 | 0.874343341893 | Nras  Krt10  Kras  Egfr  Esr1 | web link | no local link |
| Glycosphingolipid biosynthesis - ganglio series | KEGG PATHWAY | mmu00604 | 1 (0.40%) | 15 (0.17%) | 0.360997819191 | 0.874343341893 | St3gal5 | web link | no local link |
| Thiamine metabolism | KEGG PATHWAY | mmu00730 | 1 (0.40%) | 15 (0.17%) | 0.360997819191 | 0.874343341893 | Ntpcr | web link | no local link |
| Glioma | KEGG PATHWAY | mmu05214 | 3 (1.19%) | 75 (0.87%) | 0.37946326719 | 0.90923009991 | Nras  Egfr  Kras | web link | no local link |
| Signaling pathways regulating pluripotency of stem cells | KEGG PATHWAY | mmu04550 | 5 (1.98%) | 138 (1.60%) | 0.382289928371 | 0.90923009991 | Fgfr3  Nras  Lifr  Setdb1  Kras | web link | no local link |
| NF-kappa B signaling pathway | KEGG PATHWAY | mmu04064 | 4 (1.58%) | 108 (1.25%) | 0.392518094405 | 0.914429013399 | Bcl2l1  Cflar  Ube2i  Xiap | web link | no local link |
| Vascular smooth muscle contraction | KEGG PATHWAY | mmu04270 | 5 (1.98%) | 140 (1.63%) | 0.39403203611 | 0.914429013399 | Adra1a  Myl6  Cyp4a31  Arhgef12  Ppp1ca | web link | no local link |
| Selenocompound metabolism | KEGG PATHWAY | mmu00450 | 1 (0.40%) | 17 (0.20%) | 0.398073748135 | 0.914429013399 | Mtr | web link | no local link |
| Mineral absorption | KEGG PATHWAY | mmu04978 | 2 (0.79%) | 47 (0.55%) | 0.404032957934 | 0.914429013399 | Slc11a2  Slc40a1 | web link | no local link |
| Ether lipid metabolism | KEGG PATHWAY | mmu00565 | 2 (0.79%) | 47 (0.55%) | 0.404032957934 | 0.914429013399 | Enpp2  Cept1 | web link | no local link |
| Pathways in cancer | KEGG PATHWAY | mmu05200 | 17 (6.72%) | 535 (6.22%) | 0.405258312756 | 0.914429013399 | Bcl2l1  Arhgef12  Gstm4  Cdc42  Esr1  Nras  Egfr  Gsta3  Xiap  Mgst1  Vegfa  Eloc  Fas  Ctbp1  Rps6kb2  Fgfr3  Kras | web link | no local link |
| ABC transporters | KEGG PATHWAY | mmu02010 | 2 (0.79%) | 48 (0.56%) | 0.41435974677 | 0.918800077009 | Abcg8  Abcc6 | web link | no local link |
| Other glycan degradation | KEGG PATHWAY | mmu00511 | 1 (0.40%) | 18 (0.21%) | 0.415800129432 | 0.918800077009 | Gba2 | web link | no local link |
| Necroptosis | KEGG PATHWAY | mmu04217 | 6 (2.37%) | 177 (2.06%) | 0.420314108152 | 0.918800077009 | Fas  Irf9  Cflar  Xiap  Spata2l  Sharpin | web link | no local link |
| Notch signaling pathway | KEGG PATHWAY | mmu04330 | 2 (0.79%) | 49 (0.57%) | 0.424597005285 | 0.918800077009 | Ctbp1  Ncor2 | web link | no local link |
| Amino sugar and nucleotide sugar metabolism | KEGG PATHWAY | mmu00520 | 2 (0.79%) | 49 (0.57%) | 0.424597005285 | 0.918800077009 | Fpgt  Gmppa | web link | no local link |
| HIF-1 signaling pathway | KEGG PATHWAY | mmu04066 | 4 (1.58%) | 114 (1.32%) | 0.432433783755 | 0.92557776011 | Vegfa  Egfr  Eloc  Rps6kb2 | web link | no local link |
| Fatty acid degradation | KEGG PATHWAY | mmu00071 | 2 (0.79%) | 50 (0.58%) | 0.434741069143 | 0.92557776011 | Aldh3a2  Cyp4a31 | web link | no local link |
| Phospholipase D signaling pathway | KEGG PATHWAY | mmu04072 | 5 (1.98%) | 149 (1.73%) | 0.446518937366 | 0.934977618754 | Agpat3  Kras  Egfr  Nras  Tsc1 | web link | no local link |
| Gastric cancer | KEGG PATHWAY | mmu05226 | 5 (1.98%) | 150 (1.74%) | 0.452292018157 | 0.934977618754 | Nras  Kras  Egfr  Csnk1a1  Rps6kb2 | web link | no local link |
| Non-alcoholic fatty liver disease (NAFLD) | KEGG PATHWAY | mmu04932 | 5 (1.98%) | 151 (1.75%) | 0.45804935335 | 0.934977618754 | Fas  Ndufs2  Adipor2  Cox7a2  Cdc42 | web link | no local link |
| Adrenergic signaling in cardiomyocytes | KEGG PATHWAY | mmu04261 | 5 (1.98%) | 152 (1.77%) | 0.463789923818 | 0.934977618754 | Adra1a  Ppp2r3a  Ppp2r3d  Tpm1  Ppp1ca | web link | no local link |
| Cardiac muscle contraction | KEGG PATHWAY | mmu04260 | 3 (1.19%) | 86 (1.00%) | 0.465912546909 | 0.934977618754 | Cox7a2  Asph  Tpm1 | web link | no local link |
| Glycosaminoglycan degradation | KEGG PATHWAY | mmu00531 | 1 (0.40%) | 21 (0.24%) | 0.465919445747 | 0.934977618754 | Gns | web link | no local link |
| Cellular senescence | KEGG PATHWAY | mmu04218 | 6 (2.37%) | 186 (2.16%) | 0.467404078722 | 0.934977618754 | Ppp1ca  Btrc  Nras  Tsc1  Mapkapk2  Kras | web link | no local link |
| Carbon metabolism | KEGG PATHWAY | mmu01200 | 4 (1.58%) | 121 (1.41%) | 0.478059193585 | 0.934977618754 | Mcee  Idh1  Fbp1  Idh3g | web link | no local link |
| Human cytomegalovirus infection | KEGG PATHWAY | mmu05163 | 8 (3.16%) | 256 (2.97%) | 0.48114676023 | 0.934977618754 | Arhgef12  Nras  Egfr  Rps6kb2  Vegfa  Fas  Tsc1  Kras | web link | no local link |
| Other types of O-glycan biosynthesis | KEGG PATHWAY | mmu00514 | 1 (0.40%) | 22 (0.26%) | 0.481655136934 | 0.934977618754 | Poglut1 | web link | no local link |
| Proximal tubule bicarbonate reclamation | KEGG PATHWAY | mmu04964 | 1 (0.40%) | 22 (0.26%) | 0.481655136934 | 0.934977618754 | Slc38a3 | web link | no local link |
| Mismatch repair | KEGG PATHWAY | mmu03430 | 1 (0.40%) | 22 (0.26%) | 0.481655136934 | 0.934977618754 | Ssbp1 | web link | no local link |
| Steroid hormone biosynthesis | KEGG PATHWAY | mmu00140 | 3 (1.19%) | 89 (1.03%) | 0.488606617038 | 0.941548517504 | Hsd17b6  Comt  Hsd3b2 | web link | no local link |
| Huntington disease | KEGG PATHWAY | mmu05016 | 6 (2.37%) | 193 (2.24%) | 0.503331494429 | 0.949148843085 | Atp5j  Ndufs2  Cox7a2  Polr2h  Dctn4  Nrf1 | web link | no local link |
| Histidine metabolism | KEGG PATHWAY | mmu00340 | 1 (0.40%) | 24 (0.28%) | 0.511754489709 | 0.949148843085 | Aldh3a2 | web link | no local link |
| Herpes simplex virus 1 infection | KEGG PATHWAY | mmu05168 | 13 (5.14%) | 435 (5.05%) | 0.516630012099 | 0.949148843085 | Bcl2l1  Srsf5  Srsf2  Fas  Irf9  Ppp1ca  Zfp235  Zfp946  Zfp874a  Zfp141  Zfp950  Rsl1  Tsc1 | web link | no local link |
| Viral carcinogenesis | KEGG PATHWAY | mmu05203 | 7 (2.77%) | 231 (2.68%) | 0.521872352432 | 0.949148843085 | Dnaja3  Irf9  Nras  Gtf2a2  Kras  Mapkapk2  Cdc42 | web link | no local link |
| Chemical carcinogenesis | KEGG PATHWAY | mmu05204 | 3 (1.19%) | 94 (1.09%) | 0.525354524982 | 0.949148843085 | Mgst1  Gstm4  Gsta3 | web link | no local link |
| Glycosylphosphatidylinositol (GPI)-anchor biosynthesis | KEGG PATHWAY | mmu00563 | 1 (0.40%) | 25 (0.29%) | 0.526144763952 | 0.949148843085 | Pigx | web link | no local link |
| Glycerolipid metabolism | KEGG PATHWAY | mmu00561 | 2 (0.79%) | 61 (0.71%) | 0.539391957574 | 0.949148843085 | Aldh3a2  Agpat3 | web link | no local link |
| Long-term depression | KEGG PATHWAY | mmu04730 | 2 (0.79%) | 61 (0.71%) | 0.539391957574 | 0.949148843085 | Nras  Kras | web link | no local link |
| Staphylococcus aureus infection | KEGG PATHWAY | mmu05150 | 3 (1.19%) | 96 (1.12%) | 0.539644382437 | 0.949148843085 | Masp1  Cfh  Krt10 | web link | no local link |
| Hippo signaling pathway - multiple species | KEGG PATHWAY | mmu04392 | 1 (0.40%) | 26 (0.30%) | 0.540112534513 | 0.949148843085 | Nf2 | web link | no local link |
| Relaxin signaling pathway | KEGG PATHWAY | mmu04926 | 4 (1.58%) | 131 (1.52%) | 0.540605266704 | 0.949148843085 | Vegfa  Egfr  Nras  Kras | web link | no local link |
| FoxO signaling pathway | KEGG PATHWAY | mmu04068 | 4 (1.58%) | 132 (1.53%) | 0.546653455449 | 0.949148843085 | Nras  Stk11  Egfr  Kras | web link | no local link |
| Prostate cancer | KEGG PATHWAY | mmu05215 | 3 (1.19%) | 97 (1.13%) | 0.54669681261 | 0.949148843085 | Nras  Egfr  Kras | web link | no local link |
| Glycosphingolipid biosynthesis - lacto and neolacto series | KEGG PATHWAY | mmu00601 | 1 (0.40%) | 27 (0.31%) | 0.553670158466 | 0.949148843085 | St3gal4 | web link | no local link |
| Collecting duct acid secretion | KEGG PATHWAY | mmu04966 | 1 (0.40%) | 27 (0.31%) | 0.553670158466 | 0.949148843085 | Slc12a7 | web link | no local link |
| Maturity onset diabetes of the young | KEGG PATHWAY | mmu04950 | 1 (0.40%) | 27 (0.31%) | 0.553670158466 | 0.949148843085 | Hnf1b | web link | no local link |
| Ascorbate and aldarate metabolism | KEGG PATHWAY | mmu00053 | 1 (0.40%) | 27 (0.31%) | 0.553670158466 | 0.949148843085 | Aldh3a2 | web link | no local link |
| Human immunodeficiency virus 1 infection | KEGG PATHWAY | mmu05170 | 7 (2.77%) | 239 (2.78%) | 0.558323312101 | 0.950950673514 | Bcl2l1  Fas  Btrc  Nras  Rps6kb2  Eloc  Kras | web link | no local link |
| Dopaminergic synapse | KEGG PATHWAY | mmu04728 | 4 (1.58%) | 135 (1.57%) | 0.56454799332 | 0.955388911773 | Ppp2r3a  Comt  Ppp2r3d  Ppp1ca | web link | no local link |
| RNA polymerase | KEGG PATHWAY | mmu03020 | 1 (0.40%) | 29 (0.34%) | 0.57960260524 | 0.956886692822 | Polr2h | web link | no local link |
| Fatty acid elongation | KEGG PATHWAY | mmu00062 | 1 (0.40%) | 29 (0.34%) | 0.57960260524 | 0.956886692822 | Elovl5 | web link | no local link |
| T cell receptor signaling pathway | KEGG PATHWAY | mmu04660 | 3 (1.19%) | 103 (1.20%) | 0.587656527709 | 0.956886692822 | Cdc42  Nras  Kras | web link | no local link |
| Apelin signaling pathway | KEGG PATHWAY | mmu04371 | 4 (1.58%) | 139 (1.61%) | 0.587797019059 | 0.956886692822 | Nrf1  Kras  Nras  Rps6kb2 | web link | no local link |
| Glycolysis / Gluconeogenesis | KEGG PATHWAY | mmu00010 | 2 (0.79%) | 67 (0.78%) | 0.59059542941 | 0.956886692822 | Aldh3a2  Fbp1 | web link | no local link |
| MicroRNAs in cancer | KEGG PATHWAY | mmu05206 | 8 (3.16%) | 282 (3.28%) | 0.591952849544 | 0.956886692822 | Ube2i  Egfr  Nras  Tpm1  Ezh2  Vegfa  Fgfr3  Kras | web link | no local link |
| Circadian rhythm | KEGG PATHWAY | mmu04710 | 1 (0.40%) | 30 (0.35%) | 0.592000383637 | 0.956886692822 | Btrc | web link | no local link |
| Fc epsilon RI signaling pathway | KEGG PATHWAY | mmu04664 | 2 (0.79%) | 68 (0.79%) | 0.598707850381 | 0.956886692822 | Nras  Kras | web link | no local link |
| Cortisol synthesis and secretion | KEGG PATHWAY | mmu04927 | 2 (0.79%) | 69 (0.80%) | 0.606699360579 | 0.956886692822 | Hsd3b2  Mrap | web link | no local link |
| Pentose phosphate pathway | KEGG PATHWAY | mmu00030 | 1 (0.40%) | 32 (0.37%) | 0.615713952588 | 0.956886692822 | Fbp1 | web link | no local link |
| Biosynthesis of unsaturated fatty acids | KEGG PATHWAY | mmu01040 | 1 (0.40%) | 32 (0.37%) | 0.615713952588 | 0.956886692822 | Elovl5 | web link | no local link |
| Galactose metabolism | KEGG PATHWAY | mmu00052 | 1 (0.40%) | 32 (0.37%) | 0.615713952588 | 0.956886692822 | Mgam | web link | no local link |
| Parkinson disease | KEGG PATHWAY | mmu05012 | 4 (1.58%) | 144 (1.67%) | 0.615822388007 | 0.956886692822 | Atp5j  Ndufs2  Cox7a2  Ube2j2 | web link | no local link |
| Kaposi sarcoma-associated herpesvirus infection | KEGG PATHWAY | mmu05167 | 6 (2.37%) | 217 (2.52%) | 0.6186511192 | 0.956886692822 | Fas  Irf9  Nras  Vegfa  Mapkapk2  Kras | web link | no local link |
| Adipocytokine signaling pathway | KEGG PATHWAY | mmu04920 | 2 (0.79%) | 71 (0.82%) | 0.622320035255 | 0.956886692822 | Stk11  Adipor2 | web link | no local link |
| SNARE interactions in vesicular transport | KEGG PATHWAY | mmu04130 | 1 (0.40%) | 33 (0.38%) | 0.627050749463 | 0.956886692822 | Snap23 | web link | no local link |
| Starch and sucrose metabolism | KEGG PATHWAY | mmu00500 | 1 (0.40%) | 33 (0.38%) | 0.627050749463 | 0.956886692822 | Mgam | web link | no local link |
| Thyroid hormone synthesis | KEGG PATHWAY | mmu04918 | 2 (0.79%) | 73 (0.85%) | 0.637459190272 | 0.95708157499 | Asgr2  Serpina7 | web link | no local link |
| beta-Alanine metabolism | KEGG PATHWAY | mmu00410 | 1 (0.40%) | 34 (0.39%) | 0.638054383327 | 0.95708157499 | Aldh3a2 | web link | no local link |
| Pentose and glucuronate interconversions | KEGG PATHWAY | mmu00040 | 1 (0.40%) | 34 (0.39%) | 0.638054383327 | 0.95708157499 | Xylb | web link | no local link |
| Base excision repair | KEGG PATHWAY | mmu03410 | 1 (0.40%) | 35 (0.41%) | 0.648734607378 | 0.96216818173 | Apex1 | web link | no local link |
| DNA replication | KEGG PATHWAY | mmu03030 | 1 (0.40%) | 35 (0.41%) | 0.648734607378 | 0.96216818173 | Ssbp1 | web link | no local link |
| Nicotinate and nicotinamide metabolism | KEGG PATHWAY | mmu00760 | 1 (0.40%) | 36 (0.42%) | 0.659100890399 | 0.966452991093 | Nadsyn1 | web link | no local link |
| Leukocyte transendothelial migration | KEGG PATHWAY | mmu04670 | 3 (1.19%) | 115 (1.34%) | 0.662177955288 | 0.966452991093 | Arhgap5  Afdn  Cdc42 | web link | no local link |
| Hippo signaling pathway | KEGG PATHWAY | mmu04390 | 4 (1.58%) | 154 (1.79%) | 0.668212187781 | 0.966452991093 | Btrc  Nf2  Pard6a  Ppp1ca | web link | no local link |
| Salmonella infection | KEGG PATHWAY | mmu05132 | 2 (0.79%) | 78 (0.91%) | 0.673224148103 | 0.966452991093 | Flnb  Cdc42 | web link | no local link |
| Aldosterone-regulated sodium reabsorption | KEGG PATHWAY | mmu04960 | 1 (0.40%) | 38 (0.44%) | 0.678928135722 | 0.966452991093 | Kras | web link | no local link |
| Alanine, aspartate and glutamate metabolism | KEGG PATHWAY | mmu00250 | 1 (0.40%) | 38 (0.44%) | 0.678928135722 | 0.966452991093 | Agxt2 | web link | no local link |
| Natural killer cell mediated cytotoxicity | KEGG PATHWAY | mmu04650 | 3 (1.19%) | 118 (1.37%) | 0.679213987499 | 0.966452991093 | Fas  Nras  Kras | web link | no local link |
| Oocyte meiosis | KEGG PATHWAY | mmu04114 | 3 (1.19%) | 119 (1.38%) | 0.68475002718 | 0.966452991093 | Btrc  Cpeb3  Ppp1ca | web link | no local link |
| Tyrosine metabolism | KEGG PATHWAY | mmu00350 | 1 (0.40%) | 40 (0.46%) | 0.69760648925 | 0.966452991093 | Comt | web link | no local link |
| Fat digestion and absorption | KEGG PATHWAY | mmu04975 | 1 (0.40%) | 40 (0.46%) | 0.69760648925 | 0.966452991093 | Abcg8 | web link | no local link |
| B cell receptor signaling pathway | KEGG PATHWAY | mmu04662 | 2 (0.79%) | 83 (0.96%) | 0.70608301497 | 0.966452991093 | Nras  Kras | web link | no local link |
| RNA degradation | KEGG PATHWAY | mmu03018 | 2 (0.79%) | 83 (0.96%) | 0.70608301497 | 0.966452991093 | Cnot10  Xrn1 | web link | no local link |
| Yersinia infection | KEGG PATHWAY | mmu05135 | 3 (1.19%) | 123 (1.43%) | 0.706183726067 | 0.966452991093 | Baiap2  Arhgef12  Cdc42 | web link | no local link |
| Wnt signaling pathway | KEGG PATHWAY | mmu04310 | 4 (1.58%) | 162 (1.88%) | 0.70647161006 | 0.966452991093 | Porcn  Btrc  Csnk1a1  Ctbp1 | web link | no local link |
| Homologous recombination | KEGG PATHWAY | mmu03440 | 1 (0.40%) | 41 (0.48%) | 0.706535709397 | 0.966452991093 | Ssbp1 | web link | no local link |
| Platelet activation | KEGG PATHWAY | mmu04611 | 3 (1.19%) | 125 (1.45%) | 0.716476838653 | 0.97466359568 | Arhgef12  Ppp1ca  Snap23 | web link | no local link |
| Vasopressin-regulated water reabsorption | KEGG PATHWAY | mmu04962 | 1 (0.40%) | 43 (0.50%) | 0.723613881641 | 0.97466359568 | Dctn4 | web link | no local link |
| Carbohydrate digestion and absorption | KEGG PATHWAY | mmu04973 | 1 (0.40%) | 43 (0.50%) | 0.723613881641 | 0.97466359568 | Mgam | web link | no local link |
| Arachidonic acid metabolism | KEGG PATHWAY | mmu00590 | 2 (0.79%) | 89 (1.03%) | 0.741841831583 | 0.988919775806 | Cyp4f13  Cyp4a31 | web link | no local link |
| Proteasome | KEGG PATHWAY | mmu03050 | 1 (0.40%) | 46 (0.53%) | 0.747392583457 | 0.988919775806 | Psmc6 | web link | no local link |
| Progesterone-mediated oocyte maturation | KEGG PATHWAY | mmu04914 | 2 (0.79%) | 90 (1.05%) | 0.747427604438 | 0.988919775806 | Cpeb3  Kras | web link | no local link |
| Small cell lung cancer | KEGG PATHWAY | mmu05222 | 2 (0.79%) | 92 (1.07%) | 0.758289339535 | 0.988919775806 | Bcl2l1  Xiap | web link | no local link |
| Oxidative phosphorylation | KEGG PATHWAY | mmu00190 | 3 (1.19%) | 134 (1.56%) | 0.759371535621 | 0.988919775806 | Atp5j  Ndufs2  Cox7a2 | web link | no local link |
| Alzheimer disease | KEGG PATHWAY | mmu05010 | 4 (1.58%) | 175 (2.03%) | 0.761657804261 | 0.988919775806 | Atp5j  Ndufs2  Cox7a2  Fas | web link | no local link |
| Fanconi anemia pathway | KEGG PATHWAY | mmu03460 | 1 (0.40%) | 51 (0.59%) | 0.782580584244 | 0.988919775806 | Fance | web link | no local link |
| Amyotrophic lateral sclerosis (ALS) | KEGG PATHWAY | mmu05014 | 1 (0.40%) | 52 (0.60%) | 0.789008901667 | 0.988919775806 | Bcl2l1 | web link | no local link |
| Melanogenesis | KEGG PATHWAY | mmu04916 | 2 (0.79%) | 100 (1.16%) | 0.797792165177 | 0.988919775806 | Nras  Kras | web link | no local link |
| Measles | KEGG PATHWAY | mmu05162 | 3 (1.19%) | 144 (1.67%) | 0.800740544803 | 0.988919775806 | Bcl2l1  Fas  Irf9 | web link | no local link |
| Malaria | KEGG PATHWAY | mmu05144 | 1 (0.40%) | 55 (0.64%) | 0.807179911425 | 0.988919775806 | Sdc1 | web link | no local link |
| Chagas disease (American trypanosomiasis) | KEGG PATHWAY | mmu05142 | 2 (0.79%) | 103 (1.20%) | 0.811072246525 | 0.988919775806 | Cflar  Fas | web link | no local link |
| Epstein-Barr virus infection | KEGG PATHWAY | mmu05169 | 5 (1.98%) | 230 (2.67%) | 0.811840359854 | 0.988919775806 | Fas  Irf9  Ncor2  Psmc6  Entpd8 | web link | no local link |
| Regulation of lipolysis in adipocytes | KEGG PATHWAY | mmu04923 | 1 (0.40%) | 56 (0.65%) | 0.812883580594 | 0.988919775806 | Abhd5 | web link | no local link |
| Ovarian steroidogenesis | KEGG PATHWAY | mmu04913 | 1 (0.40%) | 57 (0.66%) | 0.818419180935 | 0.988919775806 | Hsd3b2 | web link | no local link |
| Endocrine and other factor-regulated calcium reabsorption | KEGG PATHWAY | mmu04961 | 1 (0.40%) | 57 (0.66%) | 0.818419180935 | 0.988919775806 | Esr1 | web link | no local link |
| Glucagon signaling pathway | KEGG PATHWAY | mmu04922 | 2 (0.79%) | 105 (1.22%) | 0.819489655174 | 0.988919775806 | Fbp1  Phkb | web link | no local link |
| Legionellosis | KEGG PATHWAY | mmu05134 | 1 (0.40%) | 58 (0.67%) | 0.823791645819 | 0.988919775806 | Hsf1 | web link | no local link |
| Calcium signaling pathway | KEGG PATHWAY | mmu04020 | 4 (1.58%) | 194 (2.25%) | 0.827430281635 | 0.988919775806 | Adra1a  Egfr  Asph  Phkb | web link | no local link |
| Toxoplasmosis | KEGG PATHWAY | mmu05145 | 2 (0.79%) | 108 (1.25%) | 0.831488304166 | 0.988919775806 | Bcl2l1  Xiap | web link | no local link |
| Fatty acid metabolism | KEGG PATHWAY | mmu01212 | 1 (0.40%) | 61 (0.71%) | 0.838977422795 | 0.988919775806 | Elovl5 | web link | no local link |
| Insulin resistance | KEGG PATHWAY | mmu04931 | 2 (0.79%) | 110 (1.28%) | 0.839084441959 | 0.988919775806 | Ppp1ca  Rps6kb2 | web link | no local link |
| Chemokine signaling pathway | KEGG PATHWAY | mmu04062 | 4 (1.58%) | 198 (2.30%) | 0.839185538549 | 0.988919775806 | Cdc42  Nras  Ccl27a  Kras | web link | no local link |
| Alcoholism | KEGG PATHWAY | mmu05034 | 4 (1.58%) | 199 (2.31%) | 0.842017896697 | 0.988919775806 | Kras  Nras  Slc29a1  Ppp1ca | web link | no local link |
| Cytosolic DNA-sensing pathway | KEGG PATHWAY | mmu04623 | 1 (0.40%) | 63 (0.73%) | 0.848369740816 | 0.988919775806 | Polr2h | web link | no local link |
| Cholinergic synapse | KEGG PATHWAY | mmu04725 | 2 (0.79%) | 113 (1.31%) | 0.849900210157 | 0.988919775806 | Nras  Kras | web link | no local link |
| TNF signaling pathway | KEGG PATHWAY | mmu04668 | 2 (0.79%) | 113 (1.31%) | 0.849900210157 | 0.988919775806 | Cflar  Fas | web link | no local link |
| Cushing syndrome | KEGG PATHWAY | mmu04934 | 3 (1.19%) | 159 (1.85%) | 0.851535456414 | 0.988919775806 | Egfr  Hsd3b2  Mrap | web link | no local link |
| Allograft rejection | KEGG PATHWAY | mmu05330 | 1 (0.40%) | 64 (0.74%) | 0.852859203142 | 0.988919775806 | Fas | web link | no local link |
| Graft-versus-host disease | KEGG PATHWAY | mmu05332 | 1 (0.40%) | 65 (0.76%) | 0.857216251551 | 0.988919775806 | Fas | web link | no local link |
| Ribosome biogenesis in eukaryotes | KEGG PATHWAY | mmu03008 | 2 (0.79%) | 116 (1.35%) | 0.860052527088 | 0.988919775806 | Tcof1  Xrn1 | web link | no local link |
| NOD-like receptor signaling pathway | KEGG PATHWAY | mmu04621 | 4 (1.58%) | 206 (2.39%) | 0.860696372307 | 0.988919775806 | Bcl2l1  Irf9  Xiap  Sharpin | web link | no local link |
| Hepatitis B | KEGG PATHWAY | mmu05161 | 3 (1.19%) | 163 (1.89%) | 0.863027003019 | 0.988919775806 | Fas  Nras  Kras | web link | no local link |
| Amphetamine addiction | KEGG PATHWAY | mmu05031 | 1 (0.40%) | 68 (0.79%) | 0.869531251528 | 0.988919775806 | Ppp1ca | web link | no local link |
| Type I diabetes mellitus | KEGG PATHWAY | mmu04940 | 1 (0.40%) | 70 (0.81%) | 0.877147533565 | 0.988919775806 | Fas | web link | no local link |
| Cell adhesion molecules (CAMs) | KEGG PATHWAY | mmu04514 | 3 (1.19%) | 171 (1.99%) | 0.883694745975 | 0.988919775806 | Sdc1  Neo1  Cadm1 | web link | no local link |
| Lysosome | KEGG PATHWAY | mmu04142 | 2 (0.79%) | 124 (1.44%) | 0.884135902101 | 0.988919775806 | Gns  Slc11a2 | web link | no local link |
| Bile secretion | KEGG PATHWAY | mmu04976 | 1 (0.40%) | 72 (0.84%) | 0.884320860054 | 0.988919775806 | Abcg8 | web link | no local link |
| cGMP-PKG signaling pathway | KEGG PATHWAY | mmu04022 | 3 (1.19%) | 173 (2.01%) | 0.88840925284 | 0.988919775806 | Adra1a  Gtf2i  Ppp1ca | web link | no local link |
| Bacterial invasion of epithelial cells | KEGG PATHWAY | mmu05100 | 1 (0.40%) | 74 (0.86%) | 0.891076896574 | 0.988919775806 | Cdc42 | web link | no local link |
| Inflammatory mediator regulation of TRP channels | KEGG PATHWAY | mmu04750 | 2 (0.79%) | 127 (1.48%) | 0.892137521846 | 0.988919775806 | Cyp4a31  Ppp1ca | web link | no local link |
| Pertussis | KEGG PATHWAY | mmu05133 | 1 (0.40%) | 76 (0.88%) | 0.89743982718 | 0.988919775806 | C4bp | web link | no local link |
| Salivary secretion | KEGG PATHWAY | mmu04970 | 1 (0.40%) | 78 (0.91%) | 0.903432439597 | 0.988919775806 | Adra1a | web link | no local link |
| Serotonergic synapse | KEGG PATHWAY | mmu04726 | 2 (0.79%) | 132 (1.53%) | 0.904345452701 | 0.988919775806 | Nras  Kras | web link | no local link |
| Autoimmune thyroid disease | KEGG PATHWAY | mmu05320 | 1 (0.40%) | 79 (0.92%) | 0.906296635703 | 0.988919775806 | Fas | web link | no local link |
| Rheumatoid arthritis | KEGG PATHWAY | mmu05323 | 1 (0.40%) | 84 (0.98%) | 0.919396954778 | 0.988919775806 | Vegfa | web link | no local link |
| ECM-receptor interaction | KEGG PATHWAY | mmu04512 | 1 (0.40%) | 86 (1.00%) | 0.924110961424 | 0.988919775806 | Sdc1 | web link | no local link |
| Viral myocarditis | KEGG PATHWAY | mmu05416 | 1 (0.40%) | 89 (1.03%) | 0.930671986152 | 0.988919775806 | Eif4g3 | web link | no local link |
| GABAergic synapse | KEGG PATHWAY | mmu04727 | 1 (0.40%) | 90 (1.05%) | 0.932730911789 | 0.988919775806 | Slc38a3 | web link | no local link |
| Antigen processing and presentation | KEGG PATHWAY | mmu04612 | 1 (0.40%) | 91 (1.06%) | 0.934728925327 | 0.988919775806 | Ifi30 | web link | no local link |
| Hypertrophic cardiomyopathy (HCM) | KEGG PATHWAY | mmu05410 | 1 (0.40%) | 91 (1.06%) | 0.934728925327 | 0.988919775806 | Tpm1 | web link | no local link |
| IL-17 signaling pathway | KEGG PATHWAY | mmu04657 | 1 (0.40%) | 91 (1.06%) | 0.934728925327 | 0.988919775806 | Mapk15 | web link | no local link |
| Human T-cell leukemia virus 1 infection | KEGG PATHWAY | mmu05166 | 4 (1.58%) | 246 (2.86%) | 0.935023671824 | 0.988919775806 | Bcl2l1  Xiap  Nras  Kras | web link | no local link |
| Cytokine-cytokine receptor interaction | KEGG PATHWAY | mmu04060 | 5 (1.98%) | 293 (3.40%) | 0.936477060422 | 0.988919775806 | Prlr  Fas  Ccl27a  Lifr  Ghr | web link | no local link |
| Dilated cardiomyopathy (DCM) | KEGG PATHWAY | mmu05414 | 1 (0.40%) | 95 (1.10%) | 0.942146979382 | 0.989720591716 | Tpm1 | web link | no local link |
| Viral protein interaction with cytokine and cytokine receptor | KEGG PATHWAY | mmu04061 | 1 (0.40%) | 101 (1.17%) | 0.951728913534 | 0.989720591716 | Ccl27a | web link | no local link |
| cAMP signaling pathway | KEGG PATHWAY | mmu04024 | 3 (1.19%) | 213 (2.47%) | 0.952950326064 | 0.989720591716 | Ppp1ca  Afdn  Fxyd1 | web link | no local link |
| Aldosterone synthesis and secretion | KEGG PATHWAY | mmu04925 | 1 (0.40%) | 102 (1.18%) | 0.953164505973 | 0.989720591716 | Hsd3b2 | web link | no local link |
| Influenza A | KEGG PATHWAY | mmu05164 | 2 (0.79%) | 164 (1.91%) | 0.95661013399 | 0.989720591716 | Fas  Irf9 | web link | no local link |
| Parathyroid hormone synthesis, secretion and action | KEGG PATHWAY | mmu04928 | 1 (0.40%) | 107 (1.24%) | 0.959729058634 | 0.989720591716 | Egfr | web link | no local link |
| Glutamatergic synapse | KEGG PATHWAY | mmu04724 | 1 (0.40%) | 114 (1.32%) | 0.967408127773 | 0.993720343494 | Slc38a3 | web link | no local link |
| Transcriptional misregulation in cancer | KEGG PATHWAY | mmu05202 | 2 (0.79%) | 183 (2.13%) | 0.973265824849 | 0.993720343494 | Bcl2l1  Ncor1 | web link | no local link |
| Cell cycle | KEGG PATHWAY | mmu04110 | 1 (0.40%) | 123 (1.43%) | 0.975176576339 | 0.993720343494 | Tfdp1 | web link | no local link |
| Osteoclast differentiation | KEGG PATHWAY | mmu04380 | 1 (0.40%) | 128 (1.49%) | 0.978663974653 | 0.993720343494 | Irf9 | web link | no local link |
| Systemic lupus erythematosus | KEGG PATHWAY | mmu05322 | 1 (0.40%) | 143 (1.66%) | 0.986459564503 | 0.995328473716 | Ssb | web link | no local link |
| Retrograde endocannabinoid signaling | KEGG PATHWAY | mmu04723 | 1 (0.40%) | 150 (1.74%) | 0.9890515341 | 0.995328473716 | Ndufs2 | web link | no local link |
| Neuroactive ligand-receptor interaction | KEGG PATHWAY | mmu04080 | 4 (1.58%) | 348 (4.04%) | 0.99280001208 | 0.995328473716 | Prlr  Adra1a  Ghr  Thra | web link | no local link |
| Tuberculosis | KEGG PATHWAY | mmu05152 | 1 (0.40%) | 178 (2.07%) | 0.995328473716 | 0.995328473716 | Arhgef12 | web link | no local link |
