# Supplementary material for: Regulation of Alternative Splicing of Lipid Metabolism Genes in Sepsis-Induced Liver Damage by RNA-Binding Proteins
Source: Inflammation. 2024 May 9;47(6):1952–68. doi: 10.1007/s10753-024-02017-2 (PMC11607010; doi:10.1007/s10753-024-02017-2)
Supplement: Supplementary file 1 — Supplementary file1 (ZIP 38655 KB) [file 10753_2024_2017_MOESM1_ESM.zip › Sup6_AS_KEGG.zip!/Sup6_AS_KEGG/ras/CLPDCA_vs_Sham/Ras_KEGG_analysis/Ras_KEGG_top.pdf]

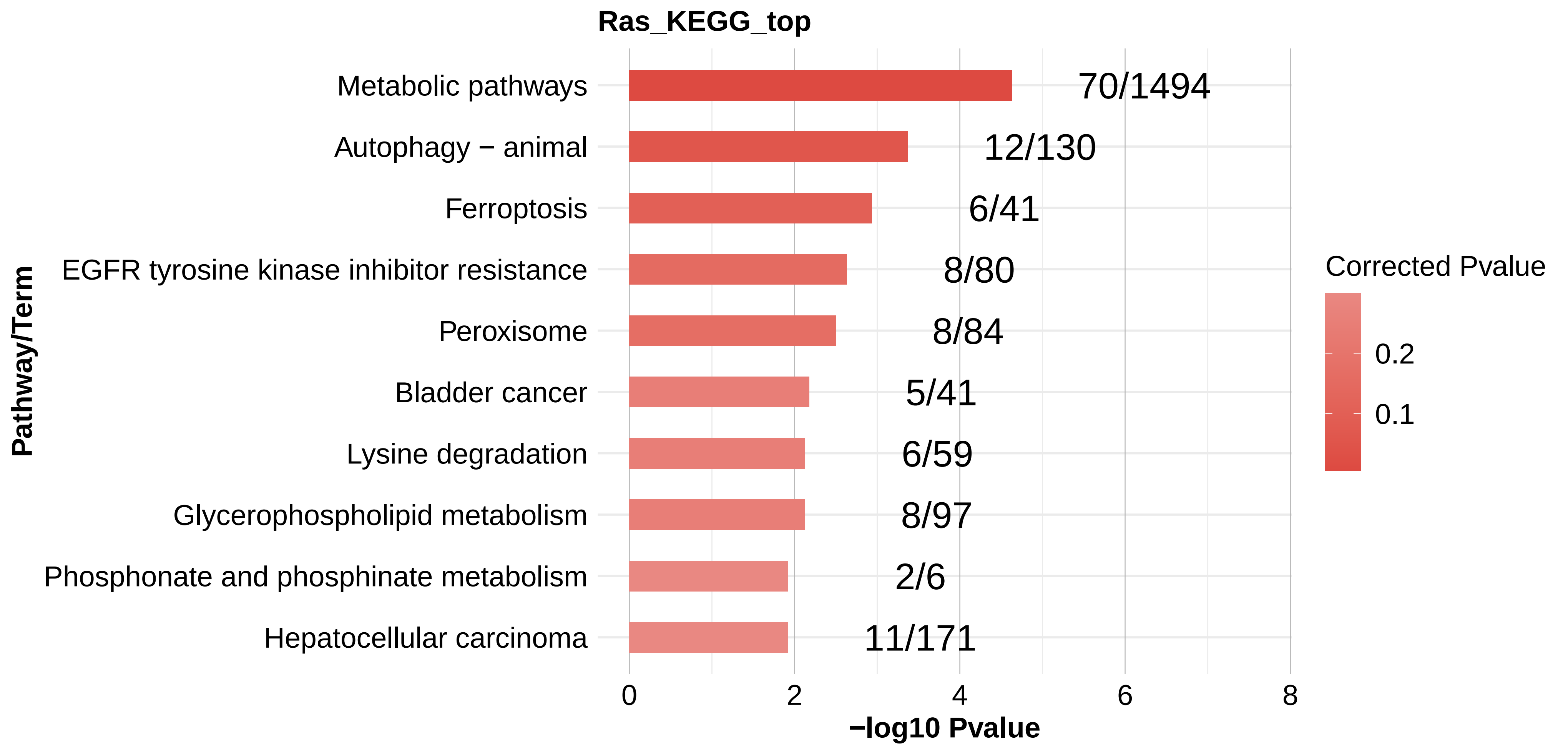
Ras_KEGG_top
Pathway/Term
Metabolic pathways
Autophagy − animal
Ferroptosis
EGFR tyrosine kinase inhibitor resistance
Peroxisome
Bladder cancer
Lysine degradation
Glycerophospholipid metabolism
Phosphonate and phosphinate metabolism
Hepatocellular carcinoma
70/1494
12/130
6/41
8/80
8/84
5/41
6/59
8/97
2/6
11/171
0
2
4
6
8
−log10 Pvalue
Corrected Pvalue
0.2
0.1
