# Supplementary material for: Regulation of Alternative Splicing of Lipid Metabolism Genes in Sepsis-Induced Liver Damage by RNA-Binding Proteins
Source: Inflammation. 2024 May 9;47(6):1952–68. doi: 10.1007/s10753-024-02017-2 (PMC11607010; doi:10.1007/s10753-024-02017-2)
Supplement: Supplementary file 1 — Supplementary file1 (ZIP 38655 KB) [file 10753_2024_2017_MOESM1_ESM.zip › Sup6_AS_KEGG.zip!/Sup6_AS_KEGG/rasg/CLP_vs_Sham/RASG_DEG_overlap_KEGG_analysis/RASG_DEG_overlap_KEGG_pathway.html]

KEGG pathway enrichment analyze result


# KEGG pathway enrichment analyze result


| Term | Database | ID | Input number (28) | Background number (8608) | P-Value | Corrected P-Value | Input | Hyperlink | img |
| --- | --- | --- | --- | --- | --- | --- | --- | --- | --- |
| Drug metabolism - cytochrome P450 | KEGG PATHWAY | mmu00982 | 5 (17.86%) | 68 (0.79%) | 2.2624998488e-06 | 0.000171949988509 | Ugt2b36  Maob  Ugt1a9  Gsta2  Fmo5 | web link | no local link |
| Metabolic pathways | KEGG PATHWAY | mmu01100 | 15 (53.57%) | 1494 (17.36%) | 1.41531023159e-05 | 0.000537817888004 | Upp2  Lipc  Papss2  St3gal5  St6gal1  Pfkfb2  Gsta2  Uox  Maob  Ugt1a9  Cyp2c40  Ugt2b36  Cyp4a31  Bdh2  Gls2 | web link | no local link |
| Drug metabolism - other enzymes | KEGG PATHWAY | mmu00983 | 4 (14.29%) | 87 (1.01%) | 0.000165615071878 | 0.00339528103817 | Ugt2b36  Upp2  Ugt1a9  Gsta2 | web link | no local link |
| Retinol metabolism | KEGG PATHWAY | mmu00830 | 4 (14.29%) | 91 (1.06%) | 0.000197087674033 | 0.00339528103817 | Ugt2b36  Cyp4a31  Ugt1a9  Cyp2c40 | web link | no local link |
| Chemical carcinogenesis | KEGG PATHWAY | mmu05204 | 4 (14.29%) | 94 (1.09%) | 0.000223373752511 | 0.00339528103817 | Ugt2b36  Ugt1a9  Gsta2  Cyp2c40 | web link | no local link |
| Porphyrin and chlorophyll metabolism | KEGG PATHWAY | mmu00860 | 3 (10.71%) | 41 (0.48%) | 0.000302505940873 | 0.00383174191772 | Ugt2b36  Cp  Ugt1a9 | web link | no local link |
| Metabolism of xenobiotics by cytochrome P450 | KEGG PATHWAY | mmu00980 | 3 (10.71%) | 66 (0.77%) | 0.00122983014738 | 0.0133524416002 | Ugt2b36  Ugt1a9  Gsta2 | web link | no local link |
| Steroid hormone biosynthesis | KEGG PATHWAY | mmu00140 | 3 (10.71%) | 89 (1.03%) | 0.00290337504704 | 0.0275820629469 | Ugt2b36  Ugt1a9  Cyp2c40 | web link | no local link |
| Ascorbate and aldarate metabolism | KEGG PATHWAY | mmu00053 | 2 (7.14%) | 27 (0.31%) | 0.00340587593304 | 0.0287607301012 | Ugt2b36  Ugt1a9 | web link | no local link |
| Pentose and glucuronate interconversions | KEGG PATHWAY | mmu00040 | 2 (7.14%) | 34 (0.39%) | 0.00536765056697 | 0.040794144309 | Ugt2b36  Ugt1a9 | web link | no local link |
| Mineral absorption | KEGG PATHWAY | mmu04978 | 2 (7.14%) | 47 (0.55%) | 0.0100771429828 | 0.0691534294225 | Mt2  Clcn2 | web link | no local link |
| Cholesterol metabolism | KEGG PATHWAY | mmu04979 | 2 (7.14%) | 49 (0.57%) | 0.0109189625404 | 0.0691534294225 | Lipc  Angptl4 | web link | no local link |
| Caffeine metabolism | KEGG PATHWAY | mmu00232 | 1 (3.57%) | 6 (0.07%) | 0.0193642847917 | 0.113206588013 | Uox | web link | no local link |
| PPAR signaling pathway | KEGG PATHWAY | mmu03320 | 2 (7.14%) | 87 (1.01%) | 0.0322005596979 | 0.148725707909 | Cyp4a31  Angptl4 | web link | no local link |
| Arachidonic acid metabolism | KEGG PATHWAY | mmu00590 | 2 (7.14%) | 89 (1.03%) | 0.0335732340817 | 0.148725707909 | Cyp4a31  Cyp2c40 | web link | no local link |
| IL-17 signaling pathway | KEGG PATHWAY | mmu04657 | 2 (7.14%) | 91 (1.06%) | 0.0349687269047 | 0.148725707909 | Mapk15  S100a9 | web link | no local link |
| Synthesis and degradation of ketone bodies | KEGG PATHWAY | mmu00072 | 1 (3.57%) | 11 (0.13%) | 0.035224509768 | 0.148725707909 | Bdh2 | web link | no local link |
| Sulfur metabolism | KEGG PATHWAY | mmu00920 | 1 (3.57%) | 11 (0.13%) | 0.035224509768 | 0.148725707909 | Papss2 | web link | no local link |
| Glycosphingolipid biosynthesis - ganglio series | KEGG PATHWAY | mmu00604 | 1 (3.57%) | 15 (0.17%) | 0.0477343133133 | 0.187347200849 | St3gal5 | web link | no local link |
| Insulin resistance | KEGG PATHWAY | mmu04931 | 2 (7.14%) | 110 (1.28%) | 0.0493018949602 | 0.187347200849 | Mlxipl  Srebf1 | web link | no local link |
| Selenocompound metabolism | KEGG PATHWAY | mmu00450 | 1 (3.57%) | 17 (0.20%) | 0.0539304135942 | 0.189440067323 | Papss2 | web link | no local link |
| Arginine biosynthesis | KEGG PATHWAY | mmu00220 | 1 (3.57%) | 19 (0.22%) | 0.0600876285755 | 0.189440067323 | Gls2 | web link | no local link |
| AMPK signaling pathway | KEGG PATHWAY | mmu04152 | 2 (7.14%) | 126 (1.46%) | 0.0627481360618 | 0.189440067323 | Pfkfb2  Srebf1 | web link | no local link |
| Inflammatory mediator regulation of TRP channels | KEGG PATHWAY | mmu04750 | 2 (7.14%) | 127 (1.48%) | 0.0636265208902 | 0.189440067323 | Cyp4a31  Cyp2c40 | web link | no local link |
| Serotonergic synapse | KEGG PATHWAY | mmu04726 | 2 (7.14%) | 132 (1.53%) | 0.0680816103376 | 0.189440067323 | Maob  Cyp2c40 | web link | no local link |
| Proximal tubule bicarbonate reclamation | KEGG PATHWAY | mmu04964 | 1 (3.57%) | 22 (0.26%) | 0.0692510549119 | 0.189440067323 | Gls2 | web link | no local link |
| Other types of O-glycan biosynthesis | KEGG PATHWAY | mmu00514 | 1 (3.57%) | 22 (0.26%) | 0.0692510549119 | 0.189440067323 | St6gal1 | web link | no local link |
| Purine metabolism | KEGG PATHWAY | mmu00230 | 2 (7.14%) | 136 (1.58%) | 0.0717194267788 | 0.189440067323 | Uox  Papss2 | web link | no local link |
| Phenylalanine metabolism | KEGG PATHWAY | mmu00360 | 1 (3.57%) | 23 (0.27%) | 0.0722863414787 | 0.189440067323 | Maob | web link | no local link |
| Histidine metabolism | KEGG PATHWAY | mmu00340 | 1 (3.57%) | 24 (0.28%) | 0.0753120820074 | 0.190790607752 | Maob | web link | no local link |
| Butanoate metabolism | KEGG PATHWAY | mmu00650 | 1 (3.57%) | 27 (0.31%) | 0.0843323160828 | 0.204029575006 | Bdh2 | web link | no local link |
| Non-alcoholic fatty liver disease (NAFLD) | KEGG PATHWAY | mmu04932 | 2 (7.14%) | 151 (1.75%) | 0.0859071894764 | 0.204029575006 | Mlxipl  Srebf1 | web link | no local link |
| Fructose and mannose metabolism | KEGG PATHWAY | mmu00051 | 1 (3.57%) | 35 (0.41%) | 0.10797339877 | 0.248666009289 | Pfkfb2 | web link | no local link |
| Alanine, aspartate and glutamate metabolism | KEGG PATHWAY | mmu00250 | 1 (3.57%) | 38 (0.44%) | 0.11668615494 | 0.257407524277 | Gls2 | web link | no local link |
| Tyrosine metabolism | KEGG PATHWAY | mmu00350 | 1 (3.57%) | 40 (0.46%) | 0.122449007328 | 0.257407524277 | Maob | web link | no local link |
| Glycine, serine and threonine metabolism | KEGG PATHWAY | mmu00260 | 1 (3.57%) | 40 (0.46%) | 0.122449007328 | 0.257407524277 | Maob | web link | no local link |
| Ferroptosis | KEGG PATHWAY | mmu04216 | 1 (3.57%) | 41 (0.48%) | 0.12531682103 | 0.257407524277 | Cp | web link | no local link |
| Tryptophan metabolism | KEGG PATHWAY | mmu00380 | 1 (3.57%) | 48 (0.56%) | 0.145139975701 | 0.266395371215 | Maob | web link | no local link |
| Cocaine addiction | KEGG PATHWAY | mmu05030 | 1 (3.57%) | 48 (0.56%) | 0.145139975701 | 0.266395371215 | Maob | web link | no local link |
| N-Glycan biosynthesis | KEGG PATHWAY | mmu00510 | 1 (3.57%) | 50 (0.58%) | 0.150723696872 | 0.266395371215 | St6gal1 | web link | no local link |
| Linoleic acid metabolism | KEGG PATHWAY | mmu00591 | 1 (3.57%) | 50 (0.58%) | 0.150723696872 | 0.266395371215 | Cyp2c40 | web link | no local link |
| Arginine and proline metabolism | KEGG PATHWAY | mmu00330 | 1 (3.57%) | 50 (0.58%) | 0.150723696872 | 0.266395371215 | Maob | web link | no local link |
| Fatty acid degradation | KEGG PATHWAY | mmu00071 | 1 (3.57%) | 50 (0.58%) | 0.150723696872 | 0.266395371215 | Cyp4a31 | web link | no local link |
| Pyrimidine metabolism | KEGG PATHWAY | mmu00240 | 1 (3.57%) | 58 (0.67%) | 0.172708939285 | 0.298315440584 | Upp2 | web link | no local link |
| Glycerolipid metabolism | KEGG PATHWAY | mmu00561 | 1 (3.57%) | 61 (0.71%) | 0.180811067486 | 0.305369802865 | Lipc | web link | no local link |
| Glutathione metabolism | KEGG PATHWAY | mmu00480 | 1 (3.57%) | 64 (0.74%) | 0.188836654063 | 0.31199099367 | Gsta2 | web link | no local link |
| Central carbon metabolism in cancer | KEGG PATHWAY | mmu05230 | 1 (3.57%) | 67 (0.78%) | 0.196786395577 | 0.315747659577 | Gls2 | web link | no local link |
| Amphetamine addiction | KEGG PATHWAY | mmu05031 | 1 (3.57%) | 68 (0.79%) | 0.199419574469 | 0.315747659577 | Maob | web link | no local link |
| Bacterial invasion of epithelial cells | KEGG PATHWAY | mmu05100 | 1 (3.57%) | 74 (0.86%) | 0.21504470662 | 0.333538728634 | Fn1 | web link | no local link |
| Platinum drug resistance | KEGG PATHWAY | mmu01524 | 1 (3.57%) | 77 (0.89%) | 0.222746584054 | 0.338574807762 | Gsta2 | web link | no local link |
| Peroxisome | KEGG PATHWAY | mmu04146 | 1 (3.57%) | 84 (0.98%) | 0.240435293125 | 0.358295730932 | Paox | web link | no local link |
| ECM-receptor interaction | KEGG PATHWAY | mmu04512 | 1 (3.57%) | 86 (1.00%) | 0.24541749037 | 0.35868710131 | Fn1 | web link | no local link |
| GABAergic synapse | KEGG PATHWAY | mmu04727 | 1 (3.57%) | 90 (1.05%) | 0.255287517283 | 0.366072666293 | Gls2 | web link | no local link |
| Small cell lung cancer | KEGG PATHWAY | mmu05222 | 1 (3.57%) | 92 (1.07%) | 0.260175729966 | 0.366173249582 | Fn1 | web link | no local link |
| AGE-RAGE signaling pathway in diabetic complications | KEGG PATHWAY | mmu04933 | 1 (3.57%) | 101 (1.17%) | 0.28179243195 | 0.388838739321 | Fn1 | web link | no local link |
| Amoebiasis | KEGG PATHWAY | mmu05146 | 1 (3.57%) | 103 (1.20%) | 0.286512755289 | 0.388838739321 | Fn1 | web link | no local link |
| Glutamatergic synapse | KEGG PATHWAY | mmu04724 | 1 (3.57%) | 114 (1.32%) | 0.311944538398 | 0.415926051197 | Gls2 | web link | no local link |
| Thyroid hormone signaling pathway | KEGG PATHWAY | mmu04919 | 1 (3.57%) | 118 (1.37%) | 0.320973909698 | 0.420586502363 | Pfkfb2 | web link | no local link |
| Yersinia infection | KEGG PATHWAY | mmu05135 | 1 (3.57%) | 123 (1.43%) | 0.332100032888 | 0.427789872872 | Fn1 | web link | no local link |
| Dopaminergic synapse | KEGG PATHWAY | mmu04728 | 1 (3.57%) | 135 (1.57%) | 0.358090157765 | 0.44469540326 | Maob | web link | no local link |
| Signaling pathways regulating pluripotency of stem cells | KEGG PATHWAY | mmu04550 | 1 (3.57%) | 138 (1.60%) | 0.364433691324 | 0.44469540326 | Lifr | web link | no local link |
| Vascular smooth muscle contraction | KEGG PATHWAY | mmu04270 | 1 (3.57%) | 140 (1.63%) | 0.368629084282 | 0.44469540326 | Cyp4a31 | web link | no local link |
| Insulin signaling pathway | KEGG PATHWAY | mmu04910 | 1 (3.57%) | 140 (1.63%) | 0.368629084282 | 0.44469540326 | Srebf1 | web link | no local link |
| Fluid shear stress and atherosclerosis | KEGG PATHWAY | mmu05418 | 1 (3.57%) | 143 (1.66%) | 0.374872141538 | 0.445160668076 | Gsta2 | web link | no local link |
| Jak-STAT signaling pathway | KEGG PATHWAY | mmu04630 | 1 (3.57%) | 165 (1.92%) | 0.4188699118 | 0.489755589182 | Lifr | web link | no local link |
| Hepatocellular carcinoma | KEGG PATHWAY | mmu05225 | 1 (3.57%) | 171 (1.99%) | 0.43034124085 | 0.49554445916 | Gsta2 | web link | no local link |
| Axon guidance | KEGG PATHWAY | mmu04360 | 1 (3.57%) | 180 (2.09%) | 0.447139788762 | 0.507203342477 | Ablim2 | web link | no local link |
| Alcoholism | KEGG PATHWAY | mmu05034 | 1 (3.57%) | 199 (2.31%) | 0.481049011493 | 0.529851085122 | Maob | web link | no local link |
| Focal adhesion | KEGG PATHWAY | mmu04510 | 1 (3.57%) | 199 (2.31%) | 0.481049011493 | 0.529851085122 | Fn1 | web link | no local link |
| Proteoglycans in cancer | KEGG PATHWAY | mmu05205 | 1 (3.57%) | 206 (2.39%) | 0.493028978832 | 0.535288605589 | Fn1 | web link | no local link |
| Regulation of actin cytoskeleton | KEGG PATHWAY | mmu04810 | 1 (3.57%) | 217 (2.52%) | 0.511317603959 | 0.547325885928 | Fn1 | web link | no local link |
| Pathways in cancer | KEGG PATHWAY | mmu05200 | 2 (7.14%) | 535 (6.22%) | 0.526753643463 | 0.556017734766 | Fn1  Gsta2 | web link | no local link |
| MicroRNAs in cancer | KEGG PATHWAY | mmu05206 | 1 (3.57%) | 282 (3.28%) | 0.607075764958 | 0.632024084066 | Gls2 | web link | no local link |
| Cytokine-cytokine receptor interaction | KEGG PATHWAY | mmu04060 | 1 (3.57%) | 293 (3.40%) | 0.621377524107 | 0.638171511245 | Lifr | web link | no local link |
| PI3K-Akt signaling pathway | KEGG PATHWAY | mmu04151 | 1 (3.57%) | 358 (4.16%) | 0.696178884039 | 0.699262220791 | Fn1 | web link | no local link |
| Human papillomavirus infection | KEGG PATHWAY | mmu05165 | 1 (3.57%) | 361 (4.19%) | 0.699262220791 | 0.699262220791 | Fn1 | web link | no local link |
