# Supplementary material for: Regulation of Alternative Splicing of Lipid Metabolism Genes in Sepsis-Induced Liver Damage by RNA-Binding Proteins
Source: Inflammation. 2024 May 9;47(6):1952–68. doi: 10.1007/s10753-024-02017-2 (PMC11607010; doi:10.1007/s10753-024-02017-2)
Supplement: Supplementary file 1 — Supplementary file1 (ZIP 38655 KB) [file 10753_2024_2017_MOESM1_ESM.zip › Sup6_AS_KEGG.zip!/Sup6_AS_KEGG/rasg/CLP_vs_Sham/RASG_DEG_overlap_KEGG_analysis/RASG_DEG_overlap_KEGG_top.pdf]

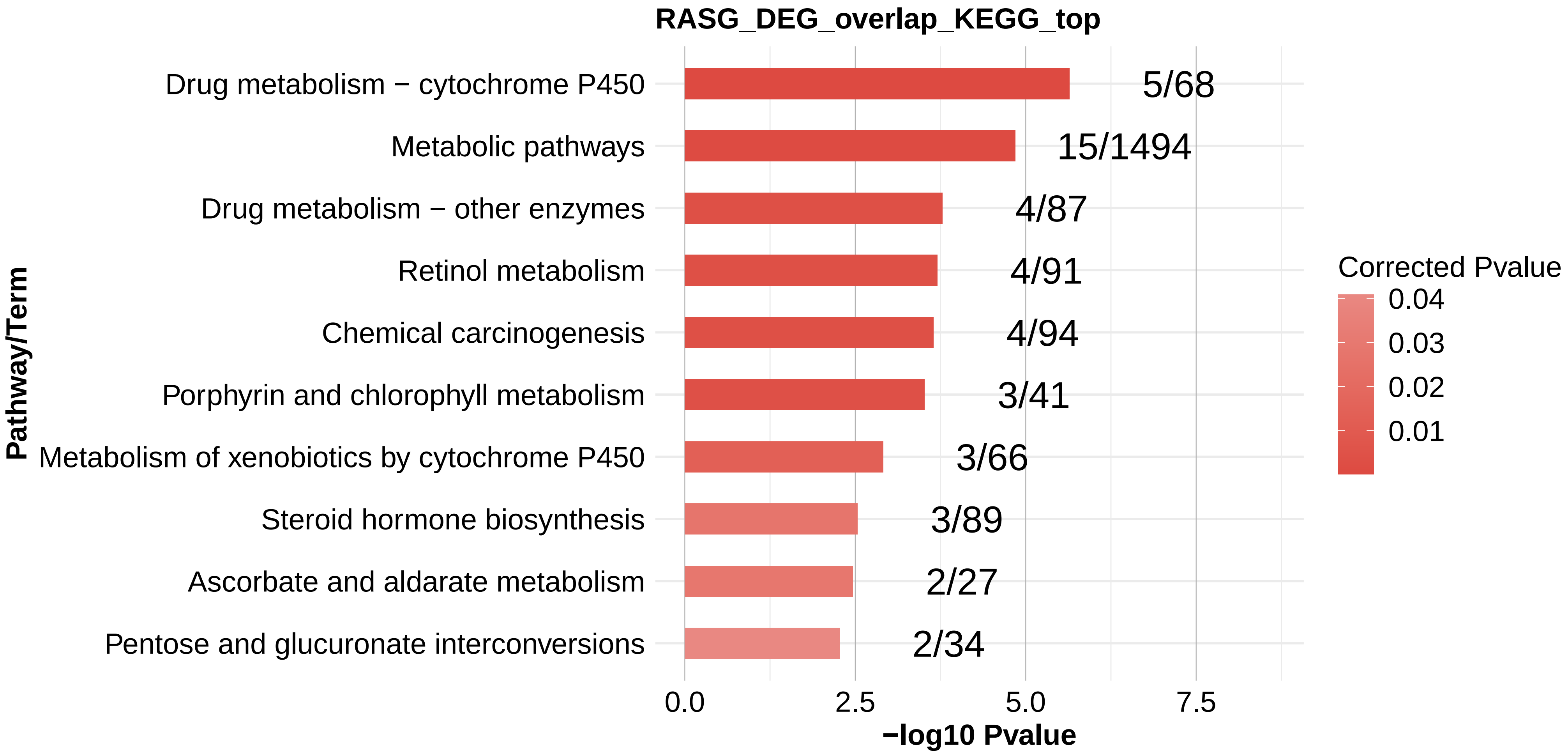
RASG_DEG_overlap_KEGG_top
Drug metabolism − cytochrome P450
Metabolic pathways
Drug metabolism − other enzymes
Retinol metabolism
Chemical carcinogenesis
Porphyrin and chlorophyll metabolism
Metabolism of xenobiotics by cytochrome P450
Steroid hormone biosynthesis
Ascorbate and aldarate metabolism
Pentose and glucuronate interconversions
5/68
15/1494
4/87
4/91
4/94
3/41
3/66
3/89
2/27
2/34
Pathway/Term
0.0
2.5
5.0
7.5
−log10 Pvalue
Corrected Pvalue
0.04
0.03
0.02
0.01
