# Supplementary material for: Regulation of Alternative Splicing of Lipid Metabolism Genes in Sepsis-Induced Liver Damage by RNA-Binding Proteins
Source: Inflammation. 2024 May 9;47(6):1952–68. doi: 10.1007/s10753-024-02017-2 (PMC11607010; doi:10.1007/s10753-024-02017-2)
Supplement: Supplementary file 1 — Supplementary file1 (ZIP 38655 KB) [file 10753_2024_2017_MOESM1_ESM.zip › Sup6_AS_KEGG.zip!/Sup6_AS_KEGG/rasg/CLPDCA_vs_Sham/RASG_DEG_overlap_KEGG_analysis/RASG_DEG_overlap_KEGG_top.pdf]

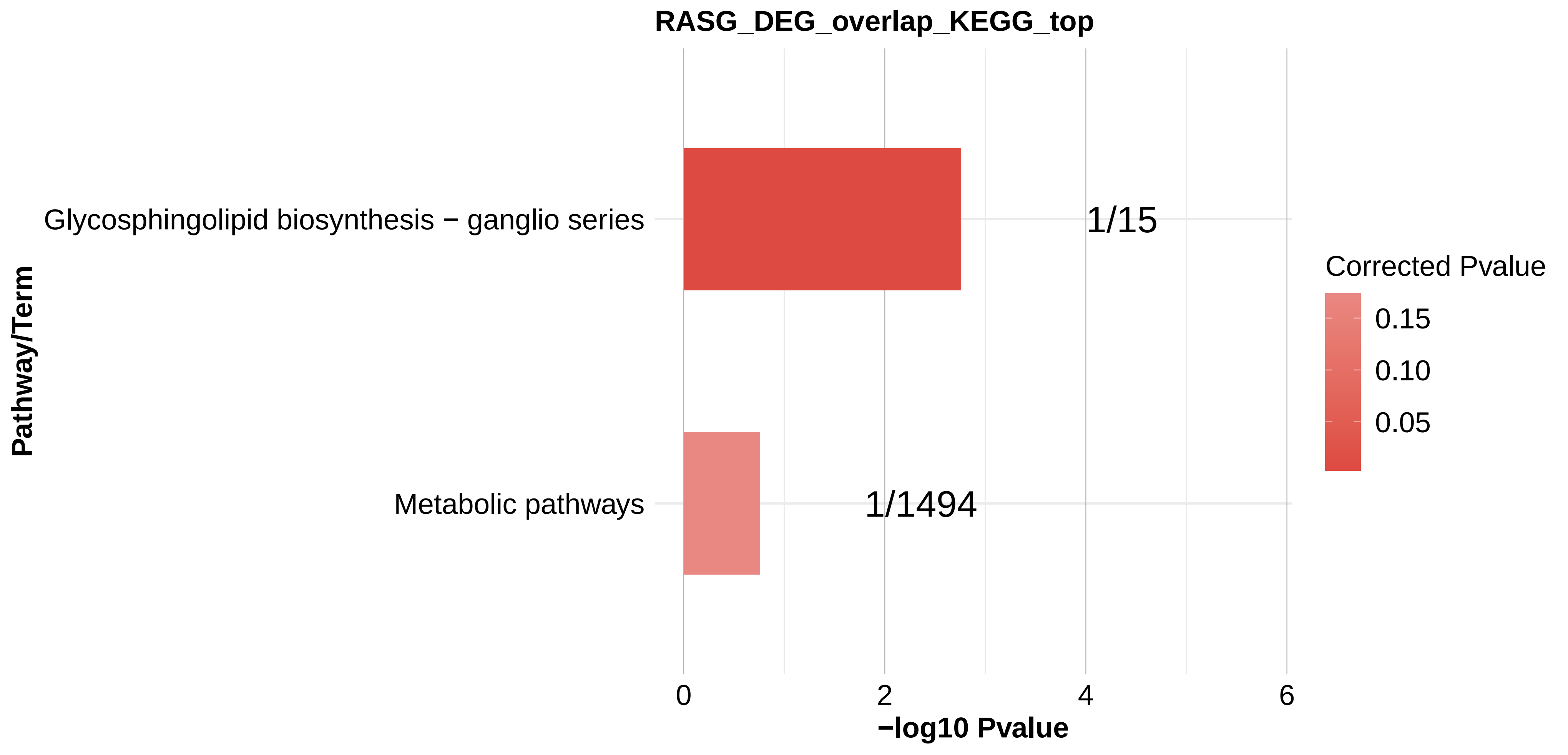
RASG_DEG_overlap_KEGG_top
Pathway/Term
Glycosphingolipid biosynthesis − ganglio series
Metabolic pathways
1/15
1/1494
0
2
4
6
−log10 Pvalue
Corrected Pvalue
0.15
0.10
0.05
