# Supplementary material for: Regulation of Alternative Splicing of Lipid Metabolism Genes in Sepsis-Induced Liver Damage by RNA-Binding Proteins
Source: Inflammation. 2024 May 9;47(6):1952–68. doi: 10.1007/s10753-024-02017-2 (PMC11607010; doi:10.1007/s10753-024-02017-2)
Supplement: Supplementary file 1 — Supplementary file1 (ZIP 38655 KB) [file 10753_2024_2017_MOESM1_ESM.zip › Sup6_AS_KEGG.zip!/Sup6_AS_KEGG/rasg/CLPDCA_vs_Sham/RASG_DEG_overlap_KEGG_analysis/RASG_DEG_overlap_KEGG_pathway.html]

KEGG pathway enrichment analyze result


# KEGG pathway enrichment analyze result


| Term | Database | ID | Input number (1) | Background number (8608) | P-Value | Corrected P-Value | Input | Hyperlink | img |
| --- | --- | --- | --- | --- | --- | --- | --- | --- | --- |
| Glycosphingolipid biosynthesis - ganglio series | KEGG PATHWAY | mmu00604 | 1 (100.00%) | 15 (0.17%) | 0.00174256505576 | 0.00348513011152 | St3gal5 | web link | no local link |
| Metabolic pathways | KEGG PATHWAY | mmu01100 | 1 (100.00%) | 1494 (17.36%) | 0.173559479554 | 0.173559479554 | St3gal5 | web link | no local link |
